# Amino Acid-Derived Ionic Chiral Catalysts Enable Desymmetrizing Cross-Coupling to Remote Acyclic Quaternary Stereocenters

**DOI:** 10.1021/jacs.3c04877

**Published:** 2023-07-20

**Authors:** Junqiang Wei, Vincent Gandon, Ye Zhu

**Affiliations:** † Department of Chemistry, Faculty of Science, National University of Singapore, 3 Science Drive 3, Singapore 117543, Singapore; ‡ Institut de Chimie Moléculaire et des Matériaux d’Orsay (UMR CNRS 8182), Paris-Saclay University, bâtiment Hesnri Moissan, 17 avenue des sciences, 91400 Orsay, France

## Abstract

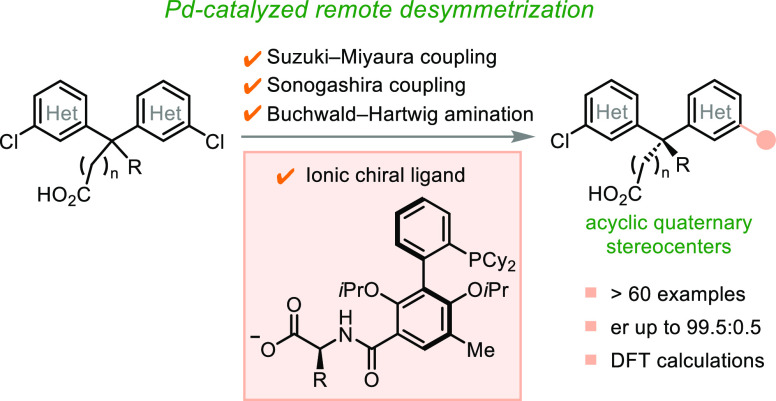

Synthetic application of asymmetric catalysis relies on strategic alignment of bond construction to creation of chirality of a target molecule. Remote desymmetrization offers distinctive advantages of spatial decoupling of catalytic transformation and generation of a stereogenic element. However, such spatial separation presents substantial difficulties for the chiral catalyst to discriminate distant enantiotopic sites through a reaction three or more bonds away from a prochirality center. Here, we report a strategy that establishes acyclic quaternary carbon stereocenters through cross-coupling reactions at distal positions of aryl substituents. The new class of amino acid-derived ionic chiral catalysts enables desymmetrizing (enantiotopic-group-selective) Suzuki–Miyaura reaction, Sonogashira reaction, and Buchwald–Hartwig amination between diverse diarylmethane scaffolds and aryl, alkynyl, and amino coupling partners, providing rapid access to enantioenriched molecules that project substituents to widely spaced positions in the three-dimensional space. Experimental and computational investigations reveal electrostatic steering of substrates by the C-terminus of chiral ligands through ionic interactions. Cooperative ion-dipole interactions between the catalyst’s amide group and potassium cation aid in the preorganization that transmits asymmetry to the product. This study demonstrates that it is practical to achieve precise long-range stereocontrol through engineering the spatial arrangements of the ionic catalysts’ substrate-recognizing groups and metal centers.

## Introduction

**Scheme 1:**
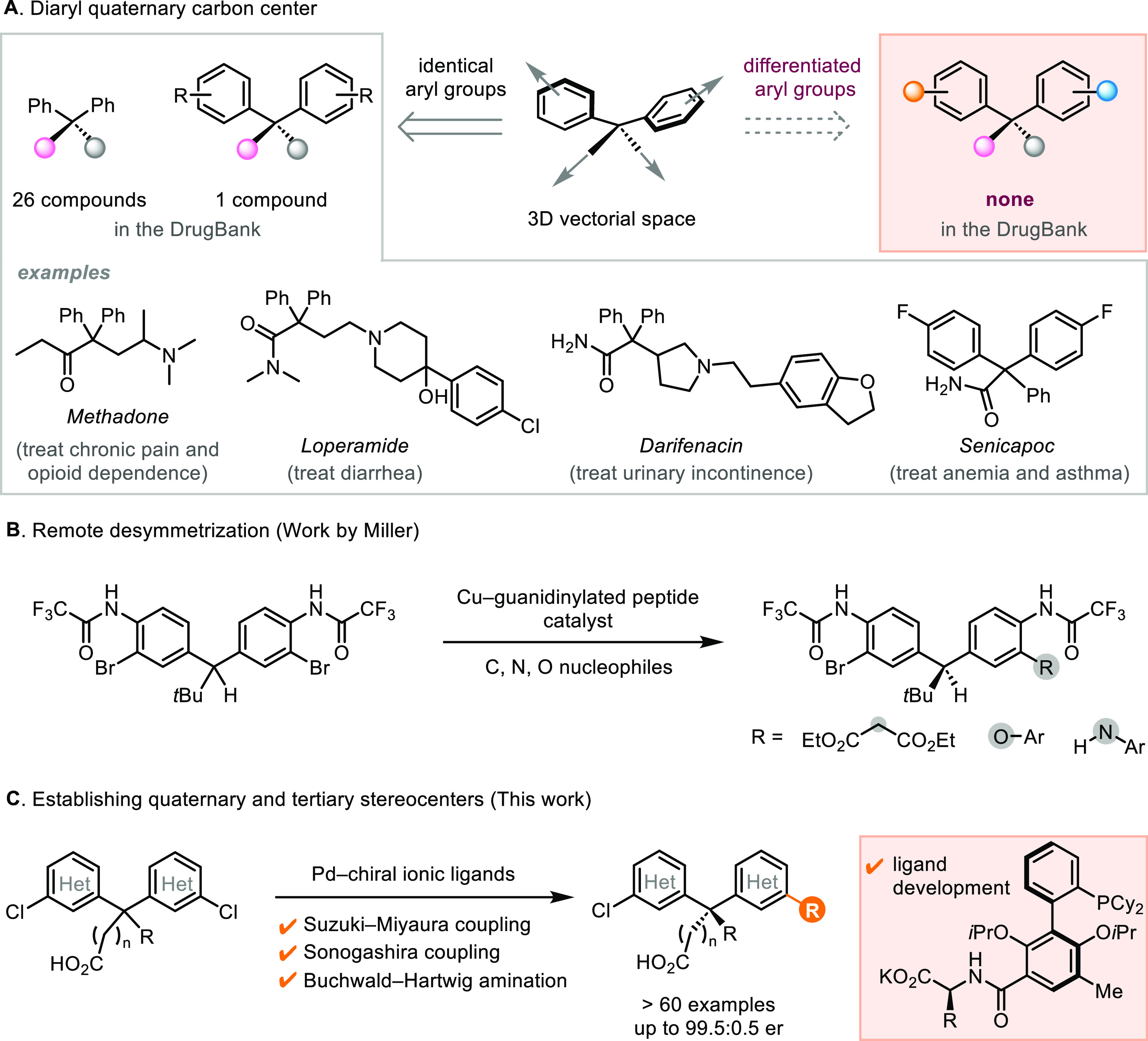
Diaryl Quaternary Carbon Centers and Desymmetrization Strategy

The property and function of a molecule are encoded in the atomic connectivity and stereochemistry of its core scaffold and peripheral substituents. Incorporation of quaternary carbon centers has emerged as the essential approach to access three-dimensional (3D) chemical space for the design of functional materials and bioactive molecules.^1^ In drug discovery, quaternization of secondary or tertiary carbons is mostly performed at peripheral positions of lead compounds and commonly through replacing hydrogen atoms by methyl groups. By contrast, applications of core scaffolds centered at quaternary carbon atoms are rare, especially at the early stages of drug discovery programs.^2^ Because binding interactions are an emergent property that is not fully divisible among fragments of a molecule,^3^ it is often desirable to explore molecular structures that direct peripheral substituents toward widely spaced binding regions in order to address challenging biological targets.^4^ Molecular scaffolds built on quaternary carbon centers bearing two aryl groups are especially appealing (Scheme 1A).^5,6^ The four vertices of the tetrahedral scaffolds warrant 3D molecular structures and project peripheral substituents to distant loci, and structural diversity is readily attainable through cross-coupling reactions at the two aryl groups. Although a number of experimental, investigational, and approved drug molecules feature nonstereogenic quaternary carbons bearing two identical aryl (mostly unsubstituted phenyl) groups, the vast pool of stereogenic quaternary carbons bearing two differentiated aryl groups has remained untapped.^7^

A compelling synthetic strategy toward this class of stereogenic centers is catalytic desymmetrization of symmetrical diarylmethane derivatives via enantiotopic-group-selective reactions. To date, desymmetrization of two enantiotopic arenes of a trisubstituted carbon through transformations at the distant *meta*-positions—most relevant to the vectorial derivatization—has been made possible by very few ingenious catalysts.^8,9^ Miller and co-workers pioneered long-range asymmetric Ullmann coupling to establish tertiary stereocenters (Scheme 1B).^10^ Yu and co-workers,^11^ and Phipps and co-workers^12^ accomplished enantioselective arene *meta* C–H functionalization to access enantioenriched (diarylmethyl)amines.^13^ Employing enzymes and organocatalysts, Lewis and co-workers^14^ and Yeung and co-workers^15^ have achieved desymmetrizing halogenation reactions of *tert*-Bu-substituted diarylmethane derivatives. These elegant studies require judicious choices of benzylic substituents (e.g., *tert*-Bu group and protected amino groups) at trisubstituted carbons, which are pivotal to the long-range enantioinduction.^16^ Recently, catalytic desymmetrization of 1,3-dicarbonyl and malononitrile derivatives to access quaternary stereocenters has gained increasing traction.^17,18^ By contrast, it has remained a formidable challenge to establish stereogenic quaternary carbons through desymmetrization of remote reaction sites because their spatial distancing necessitates long-range stereocontrol, which is exacerbated by the requirement to discern nonhydrogen groups attached to the sterically congested prochirality center.^19−21^ To date, catalytic desymmetrization has been achieved only at the *ortho* positions of the geminal arenes in the context of diaryl quaternary carbon stereocenters.^22^

We have recently disclosed an ionic catalyst-enabled desymmetrizing Suzuki–Miyaura reaction to establish remote cyclic quaternary stereocenters within fluorene and xanthene skeletons.^23,24^ Within a given substrate, the enantiotopic reaction sites are distant from the ionic catalyst-binding group.^25^ This key feature differs from previous catalyst systems that employ ligands containing chiral ionic groups, where enantiocontrol is invariably exerted directly at the counterionic sites of reaction intermediates.^26^ We hypothesize that the long-range ionic stereocontrol strategy could be applicable to the creation of chiral elements that are difficult to establish using conventional methods.^27^ Here, we report the evolution of a new family of ionic chiral ligands—amino-acid-functionalized dialkylbiaryl phosphines,^28,29^ which allows rapid access to acyclic quaternary^30^ and tertiary stereocenters through desymmetrizing Suzuki–Miyaura, Sonogashira, and Buchwald–Hartwig coupling reactions (Scheme 1C). In this study, we propose that the ionic chiral ligands can be engineered to achieve optimal geometrical arrangements of metal-binding phosphorous atoms and substrate-binding ionic groups. The diversification of ionic chiral catalysts holds the key to overcoming the challenges posed by distal quaternary prostereogenic carbon centers and to significantly expanding the scope of both pro-stereogenic scaffolds and coupling partners. We also aim to further elucidate the underlying mode of ionic stereocontrol over this unique symmetry-breaking process through both experimental and computational investigations, which reveal the intriguing electrostatic interactions engendered by the amide group and the terminal carboxylate of the ligand.

## Results and Discussion

### Initial Investigation of Cyclic and Acyclic Substrates

**Scheme 2:**
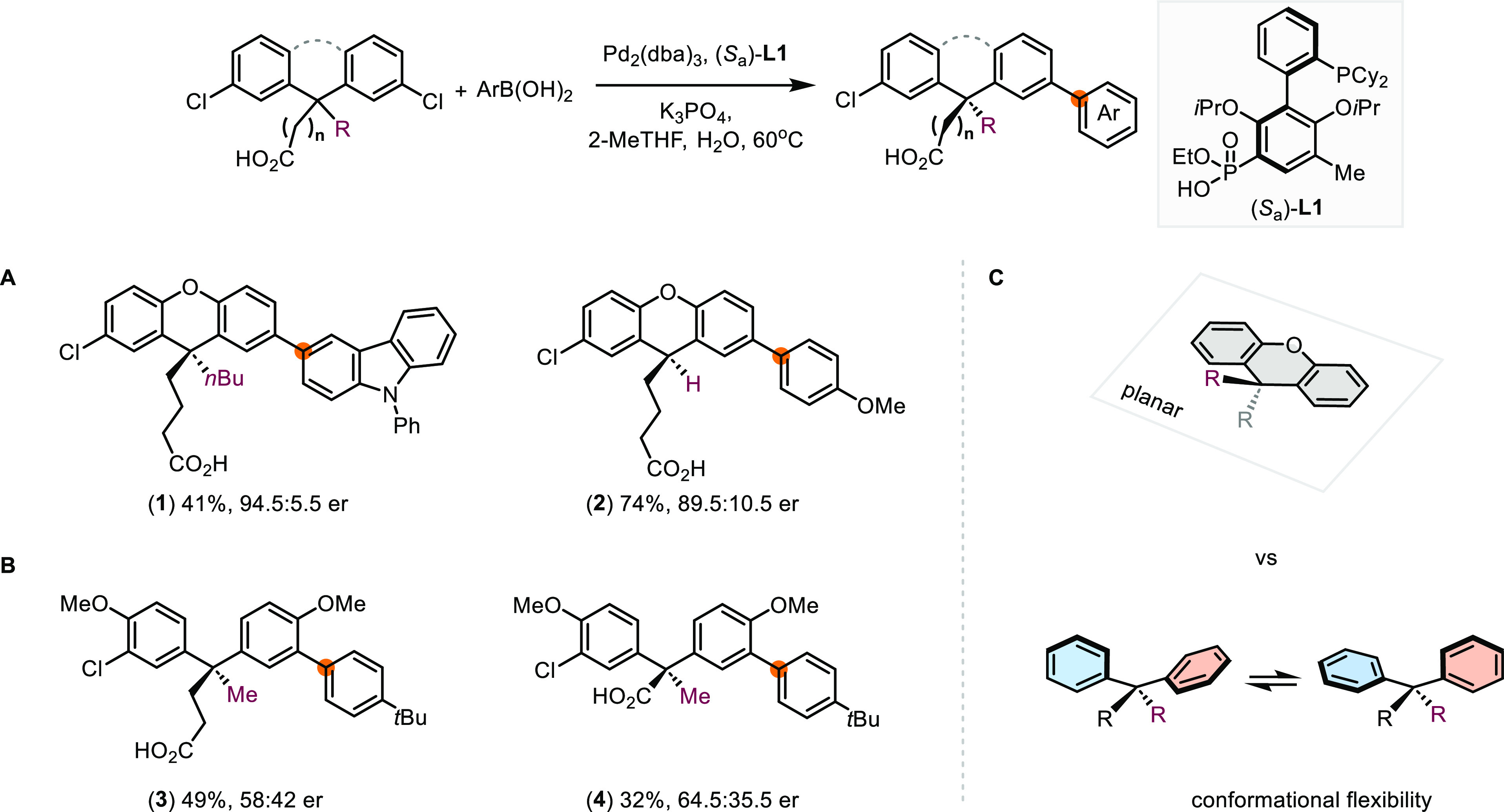
Comparison between Desymmetrization of Cyclic and Acyclic Substrates Reaction conditions: Bis(chloroaryl)carboxylic acid (0.25 mmol), arylboronic acid (0.30 mmol), Pd_2_(dba)_3_ (1.0 mol %), (*S*_a_)-**L1** (2.2 mol %), K_3_PO_4_ (2.5 mmol), 2-MeTHF (20 mL/mmol), H_2_O (1.6 mL/mmol), 60 °C, 18 h. Isolated yields reported. Enantiomeric ratio (er) was determined using high-performance liquid chromatography with the chiral stationary phase (HPLC–CSP). The absolute configurations of xanthene products were assigned according to ref (23). dba: dibenzylideneacetone.

At the start, we evaluated the performance of our first-generation phosphonate ligand (*S*_a_)-**L1** (a: axial chirality) in desymmetrizing Suzuki–Miyaura reactions of both cyclic and acyclic substrates. As an expansion of our previous study using cyclic substrates,^23^ we targeted xanthene-carbazole scaffold, which has been broadly utilized in organic light-emitting diodes (OLEDs). Coupling product **1** was synthesized with high enantiopurity (94.5:5.5 er), albeit in modest isolated yield (41%) as a result of incomplete conversion and the difficulty in chromatographic purification of the free carboxylic acid product from unconsumed starting material (Scheme 2A). Moreover, a substrate containing a prostereogenic tertiary carbon underwent the desymmetrization reaction with slightly reduced enantioselectivity (**2**, 74% yield, 89.5:10.5 er).

Meanwhile, our attempt to expand the scope to acyclic substrates revealed their differences from the cyclic counterparts (Scheme 2B). Under the optimized reaction conditions for xanthene substrates, the desymmetrization of a 4,4-diaryl pentanoic acid derivative afforded coupling product **3** in merely 58:42 er. The reaction of a 2,2-diaryl propanoic acid derivative gave marginally improved stereoselectivity (**4**, 64.5:35.5 er). Plausibly, the contrast between the effectiveness of the phosphonate ligand (*S*_a_)-**L1** toward cyclic and acyclic substrates was attributable to the different conformations of the diarylmethane moiety. While xanthene is planar and rigid,^31^ the acyclic diarylmethane scaffold adopts nonplanar, flexible conformations that raise the entropic penalty of the preorganization between the substrate and catalyst (Scheme 2C). The preliminary investigation presented the opportunity to address the challenging stereocontrol over acyclic quaternary centers by evolving the ionic chiral phosphine ligands.

### Development of Ionic Chiral Ligands

**Scheme 3:**
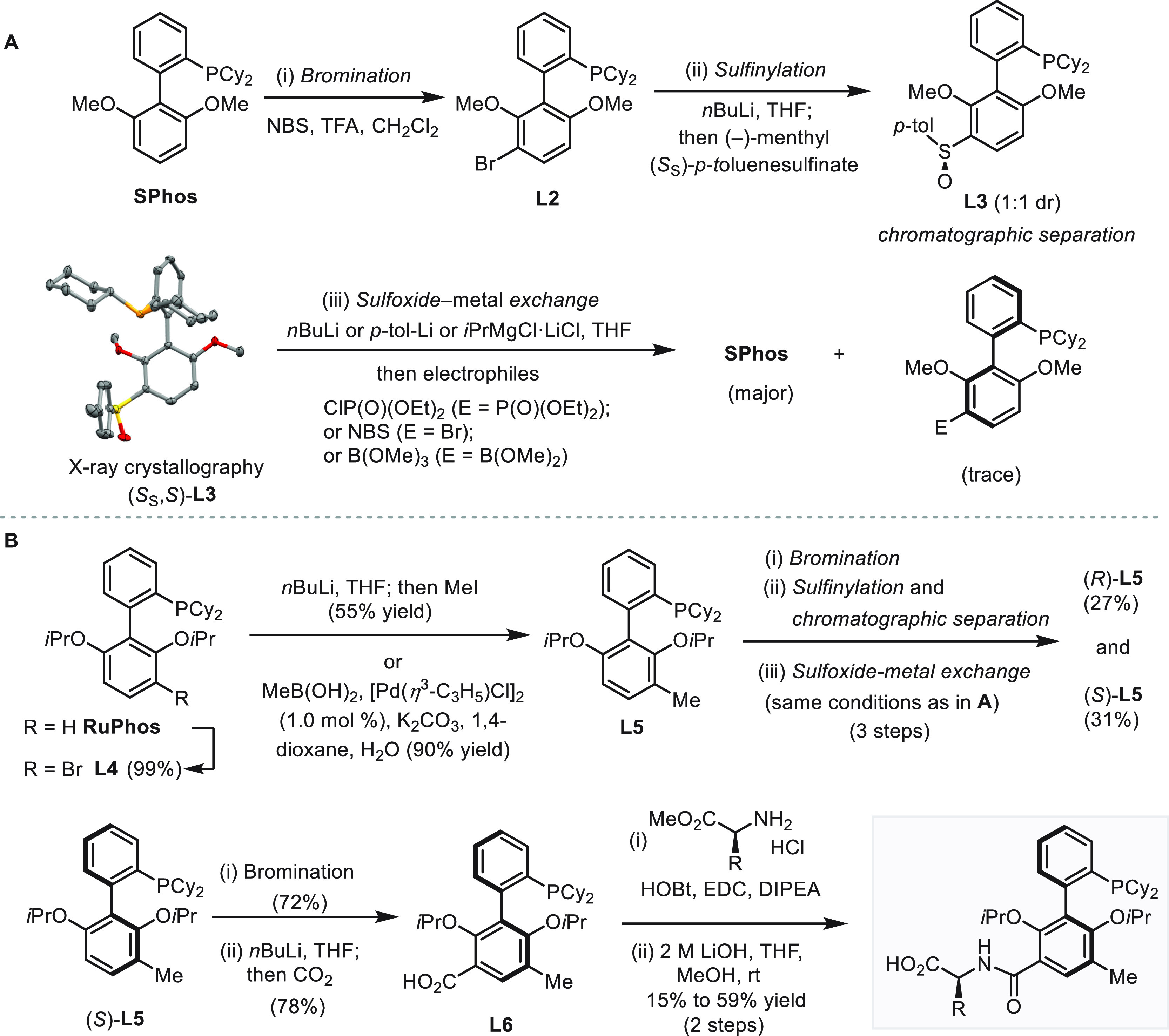
Synthesis of Amino Acid-Derived Chiral Phosphine Ligands NBS: *N*-bromosuccinimide, TFA: trifluoroacetic acid, HOBt: hydroxybenzotriazole, DIPEA: *N*,*N*-diisopropylethylamine, EDC: 1-ethyl-3-(3-dimethylaminopropyl)carbodiimide.

Initially, we devised a three-step synthesis toward 3′-functionalized, axially chiral dialkylbiaryl phosphine ligands starting from commercially available SPhos and RuPhos.^32^ The preparation and separation of atropo-diastereomers of **L3** were successful (Scheme 3A). Despite multiple attempts, we only observed the formation of 3′-functionalized ligands in trace amounts via sulfoxide–metal exchange using organolithium and organomagnesium reagents followed by the addition of electrophilic agents. Instead, SPhos was obtained even when the reaction was quenched prematurely with various electrophiles. Presumably, the 3′-lithio intermediate underwent rapid protonation in the presence of sulfoxide byproducts from the sulfoxide–metal exchange step.^33^

Therefore, we adjusted our plan by incorporating a methyl group as an “atropo-tag” (Scheme 3B). Upon bromination of the RuPhos at 3′-position, the methyl group can be readily installed through lithium–bromide exchange of **L4** followed by methylation using iodomethane. Alternatively, **L5** can be prepared through the Pd-catalyzed Suzuki–Miyaura reaction.^34^ Following the three-step synthesis we initially devised, both enantiomers of **L5** are accessible in enantioenriched forms. Subsequently, bromination of (*S*_a_)-**L5** followed by carboxylation^35^ using CO_2_ yielded axially chiral 3′-carboxyl ligand **L6**.

In addition to acting as a substrate-interacting group, the carboxyl group of **L6** could serve as a handle for further structural diversification of the ionic catalysts. We next explored a new class of amino acid-derived axially chiral biaryl phosphine ligands. Using peptide coupling reagents, an array of amino acid methyl esters was coupled to **L6** through formation of an amide linker. Subsequent saponification unveiled the terminal carboxyl group, which could serve as the anionic motif of chiral catalysts upon deprotonation under the basic conditions of Pd-catalyzed desymmetrization reactions. In addition, the added chirality center of the amino acid moiety could direct the spatial arrangement of the terminal carboxyl group in respect with the phosphorous atom.

Alternatively, we have attempted to separate the diastereomers upon amide coupling between racemic **L6** and enantiopure amino-acid esters. Although peak resolution could be achieved using analytical HPLC, we were unable to identify suitable conditions for preparative separation using normal-phase chromatography on silica gel columns. Besides, chromatographic separation of the diastereomeric esters derived from racemic **L6** and (−)-menthol was unsatisfactory. Gratifyingly, we have streamlined the experimental operations of the synthetic route in Scheme 3B to enable gram-scale preparation of ligands.

### Establishing Acyclic Quaternary Stereocenters

**Scheme 4:**
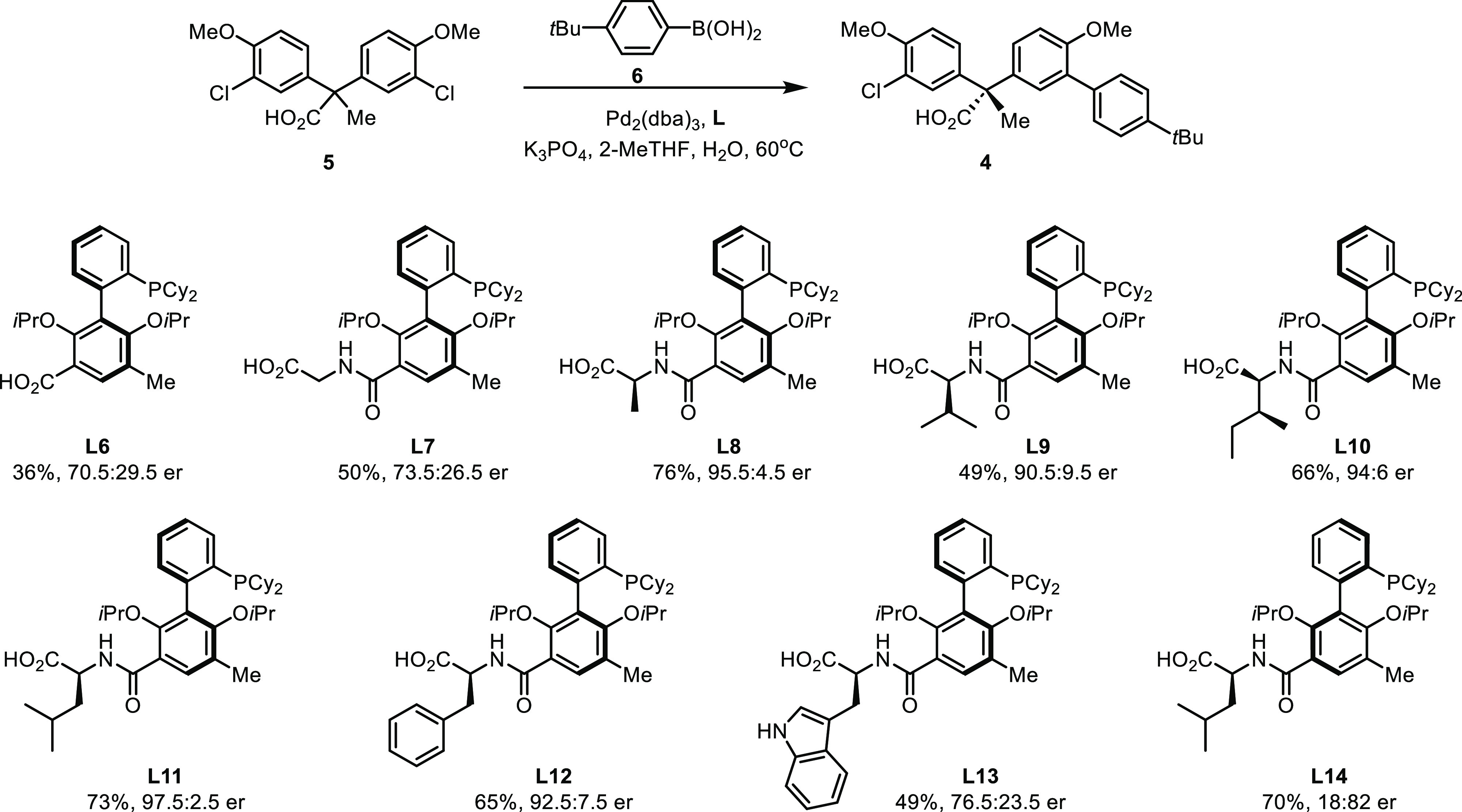
Optimization of Desymmetrization to Establish Acyclic Quaternary Stereocenters^,^ Reaction conditions: **5** (0.1 mmol), **6** (0.12 mmol), Pd_2_(dba)_3_ (1.0 mol %), ligand (2.0 mol %), K_3_PO_4_ (0.5 mmol), 2-MeTHF (20 mL/mmol), H_2_O (2 mL/mmol), 60 °C, 18 h. Er was determined using HPLC–CSP. The major enantiomer of reaction product **4** using (*R*_a_, *S*)-**L6** to **L13** is opposite to that obtained using (*S*_a_)-**L1** and (*S*_a_, *S*)-**L14** (Table S1 in the Supporting Information, SI). The absolute configuration of **4** was assigned by analogy to **39** (Scheme 6) and **70** (Scheme 8).

With the new family of ionic chiral phosphine ligands in hands, we reinvestigated the desymmetrization Suzuki–Miyaura coupling between 2,2-diaryl propanoic acid **5** and aryl boronic acid **6** (Scheme 4). In comparison with the reaction using phosphonate ligand (*S*_a_)-**L1** (35.5:64.5 er), similar levels of enantioselectivity were obtained using 3′-carboxylate (*R*_a_)-**L6** (70.5:29.5 er) and glycine-derived (*R*_a_)-**L7** (73.5:26.5 er). The results suggest that extending the distance between the ligand’s nonligating anionic group and the phosphine through an amide linker does not lead to significant change in the mode of stereocontrol.

Encouragingly, the model reaction afforded the desymmetrization product **4** in 76% yield with 95.5:4.5 er when alanine-derived (*R*_a_, *S*)-**L8** was used as the supporting ligand. Presumably, the additional chirality center of the amino acid moiety enhances the stereoselectivity engendered by the ligand’s axially chiral biaryls backbone. The increased steric bulkiness of valine-derived (*R*_a_, *S*)-**L9** and isoleucine-derived (*R*_a_, *S*, *S*)-**L10** resulted in slightly reduced er (90.5:9.5 and 94:6, respectively). High enantioselectivity was achieved when leucine-derived (*R*_a_, *S*)-**L11** was used as the ligand (97.5:2.5 er). Interestingly, while reaction using phenylalanine-derived (*R*_a_, *S*)-**L12** gave comparable enantioselectivity (92.5:7.5 er) to the ligands that consist of aliphatic amino acids, noticeable decrease in er (76.5:23.5) was observed when tryptophan-derived (*R*_a_, *S*)-**L13** was utilized. Possibly, the indolyl group plays a detrimental role through hydrogen bonding interactions.

Furthermore, in contrast to (*R*_a_, *S*)-**L11**, the diastereomeric (*S*_a_, *S*)-**L14** gave relatively low selectivity (18:82 er) favoring the opposite enantiomer of desymmetrization product **11**. On the basis of the result, we concluded that the axial chirality of the ligands is determinant in stereocontrol, while the chirality of amino acid moiety plays a secondary role. In addition, the chirality of ligand’s biaryls backbone and chirality of amino acid moiety match in the case of (*R*_a_, *S*)-**L11**, thus transmitting the asymmetry to the substrate synergistically.

Subsequently, we evaluated the effect of alkali and alkaline earth metal cations (Table S2 in SI). The reactions using of KOH, NaOH, and CsOH exhibited similar levels of stereocontrol (95:5–97.5:2.5 er), while diminished enantioselectivity was observed using LiOH, Ca(OH)_2_, and Ba(OH)_2_ (84.5:15.5–91.5:8.5 er). The results confirm the involvement of cations in the enantio-determining step and show that stereocontrol is attainable with both monovalent and bivalent cations. Ultimately, we chose (*R*_a_, *S*)-**L11** as the premier ligand and K_3_PO_4_ in water/2-MeTHF as the optimal reaction media, given the excellent enantioselectivity and the enhanced functional group compatibility by avoiding metal hydroxide bases.

### Substrate Scope Expansion

**Scheme 5:**
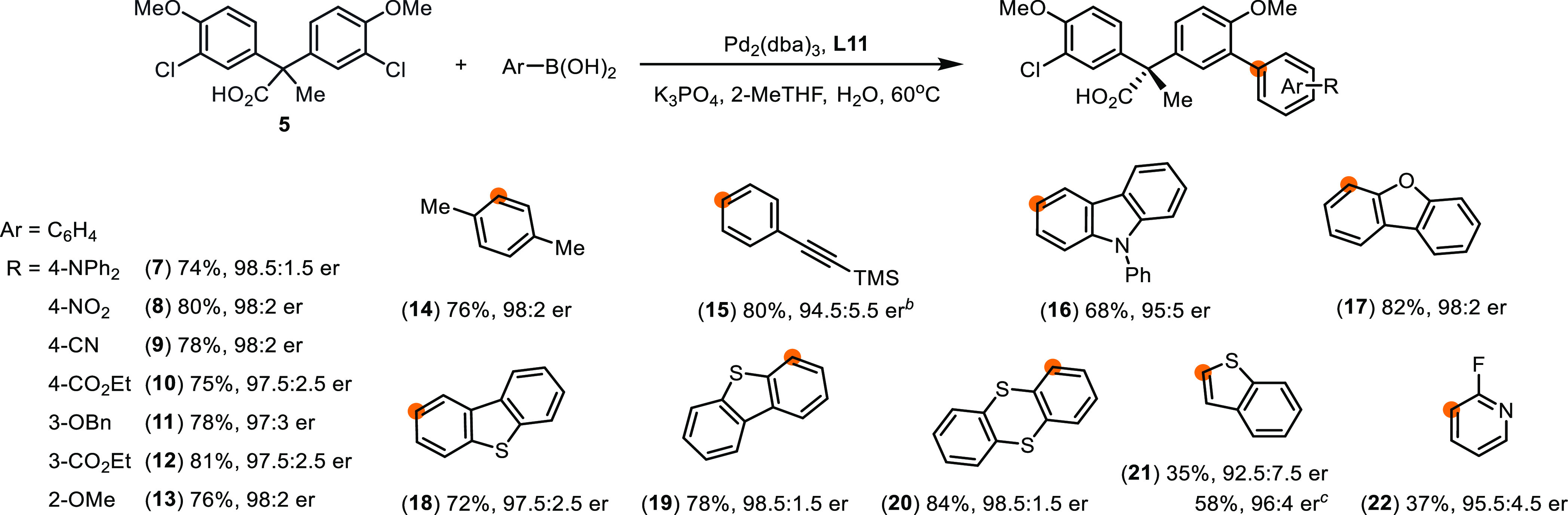
Scope of Aryl Boronic Acids for Desymmetrizing Suzuki–Miyaura Reaction Reaction conditions: **12** (0.25 mmol), arylboronic acid (0.3 mmol), Pd_2_(dba)_3_ (1.0 mol %), **L11** (2.0 mol %), K_3_PO_4_ (1.25 mmol), 2-MeTHF (20 mL/mmol), H_2_O (2 mL/mmol), 60 °C, 18 h. Isolated yields as ethyl esters reported. Er was determined using HPLC–CSP. The absolute configurations of products were assigned by analogy to **39** (Scheme 6) and **70** (Scheme 8). Prepared from aryl boronic pinacol ester. The reaction was carried out using benzothiophene-2-boronic acid (3.6 equiv), Pd_2_(dba)_3_ (3.0 mol %), **L11** (6.0 mol %), K_3_PO_4_ (10 equiv).

With the optimal conditions in hand, we turned our attention to exploring the substrate scope of the remote desymmetrizing cross-coupling. First, a broad spectrum of aryl boronic acids was examined (Scheme 5). Electron-rich (**7**, **11**, **13**), electron-deficient (**8**–**10**, **12**), and electron-neutral (**14**, **15**) functional groups were all well tolerated. The substitution patterns (*para***7**–**10**; *meta***11**, **12**; *ortho***13**, **14**) had insignificant impact on the reactivity and enantioselectivity (74–81% yield, 97:3–98.5:1.5 er). Besides, the reaction is compatible with aryl boronic pinacol ester (**15**, 80% yield, 94.5: 5.5 er). The results suggest that the amino acid moiety of the carboxylate catalyst unlikely reacts with aryl boronic acid or the boric acid byproduct under the reaction conditions.^36^

Moreover, various polycyclic heteroaryl groups that are commonly employed in π-conjugated materials, such as carbazole (**16**), dibenzofuran (**17**), dibenzothiophene (**18**, **19**), and thianthrene (**20**), can be installed through the desymmetrizing Suzuki–Miyaura reaction in high enantioselectivity (68–84% yield, 95:5–98.5:1.5 er). Finally, heteroaryl boronic acids including benzothiophene and fluoropyridine participated in the transformation, affording the coupling products in comparable enantioselectivity (**21**, 92.5:7.5 er; **22**, 95.5:4.5 er). When the reaction was performed using 3.6 equivalents of benzothiophene boronic acid, compound **21** was obtained in increased yield (58%) and improved enantiopurity (96:4 er), along with a small quantity of the doubly coupled product (14%). Plausibly, a kinetic resolution in the secondary coupling enhances the enantiopurity of the primary product (vide infra).

**Scheme 6:**
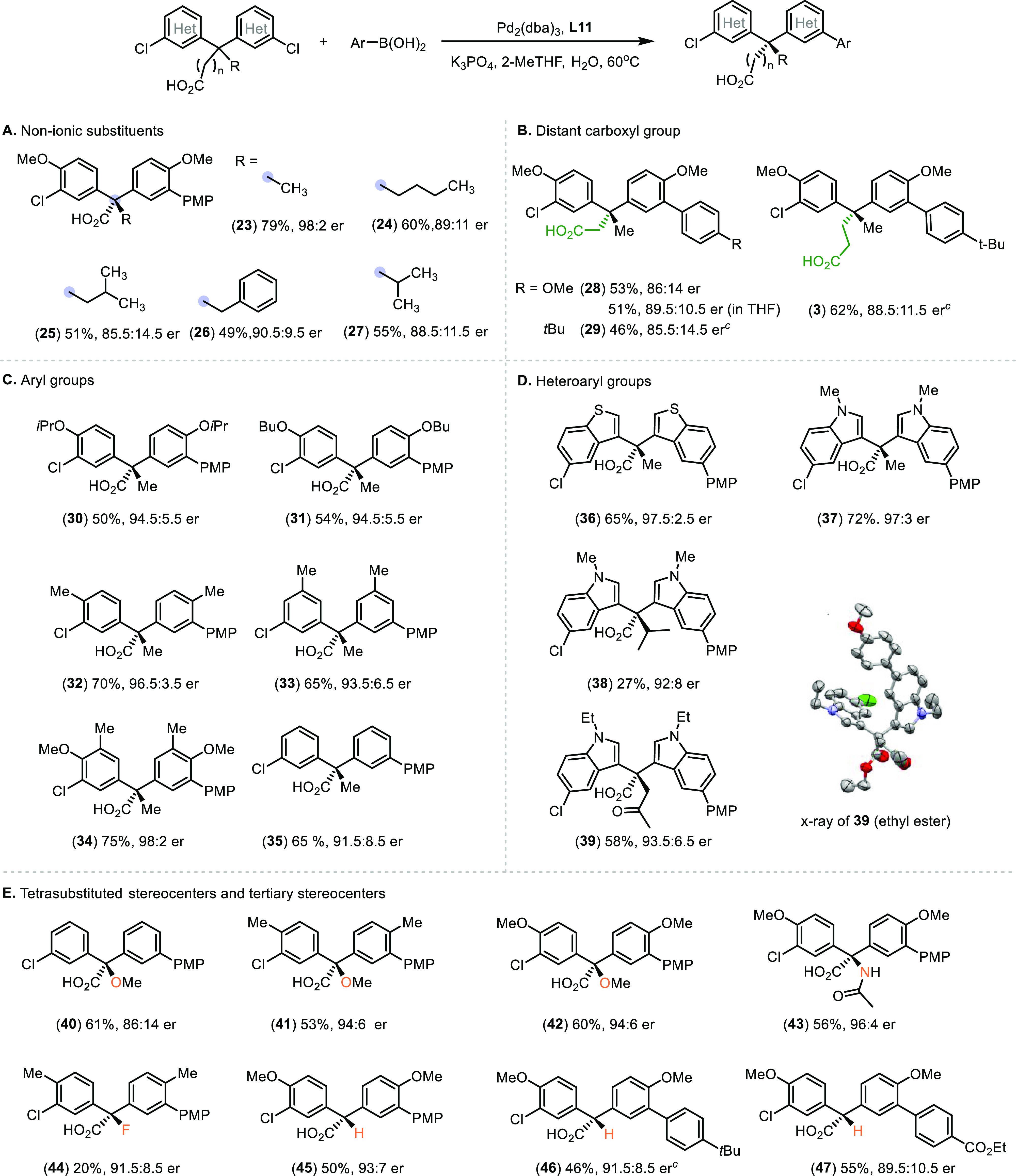
Desymmetrizing Suzuki–Miyaura Reaction of Diverse Prochiral Substrates^,^ Reaction conditions: bis(chloroaryl)carboxylic acid (0.25 mmol), arylboronic acid (0.3 mmol), Pd_2_(dba)_3_ (1.0 mol %), **L11** (2.0 mol %), K_3_PO_4_ (1.25 mmol), 2-MeTHF (20 mL/mmol), H_2_O (2 mL/mmol), 60 °C, 18 h. Isolated yields of ethyl esters reported. Er was determined using HPLC–CSP. The major enantiomer of **3** from reaction using (*R*_a_, *S*)-**L11** is opposite to that obtained using (*S*_a_)-**L1** (Scheme 2B). The absolute configuration of **39** was assigned by X-ray crystallography. The absolute configurations of other products were assigned by analogy to **39** and **70** (Scheme 8). Isolated yields of carboxylic acids reported. PMP: *p*-methoxyphenyl.

Next, we turned our attention to the nonionic substituents of the quaternary centers (Scheme 6A). Among the substrates investigated, the one bearing a methyl group afforded the highest enantioselectivity (**23**, 79% yield, 98:2 er). The steric bulkiness of the non-ionic α-substituents has marginal influence on the enantioselectivity (**24**–**27**, 49–60% yield, 85.5:14.5–90.5:9.5 er). These results show that the asymmetric induction by the amino-derived chiral catalyst differs from known examples of desymmetrization to form quaternary carbons, which commonly rely on steric biasing between a methyl group and a bulky alkyl group or an aryl substituent.^17,24a,31c^ In addition, we investigated the effect of extending the aliphatic linker between the carboxyl group and the pro-stereogenic carbon (Scheme 6B). Clearly, the Pd–**L11** catalyst is adaptable to the pronounced change in spatial arrangements between the substrates’ distal carboxylate and reaction sites. Slightly improved enantioselectivity was observed when the reaction is carried out in THF (**28**, 89.5:10.5 er) than in 2-MeTHF (86:14 er).

Subsequently, we explored various substitution patterns on the enantiotopic chloroarenes to probe their effects on the desymmetrization process (Scheme 6C). Previously, Miller and co-workers discovered that trifluoroacetamide was crucial for the exquisite substrate–catalyst preorganization, leading to high stereoselectivity (Scheme 1B).^10^ Contrary to our initial expectation that the MeO substituents adjacent to the reaction sites could be influential, replacing it for *i*PrO and *n*BuO groups (**30**, **31**, 50–54% yield, 94.5:5.5 er) and for *ortho* Me- and *meta* Me- groups did not affect the stereocontrol significantly (**32**, **33**, **34**, 65–75% yield, 93.5:6.5–98:2 er). On the other hand, slightly reduced enantiopurity was observed using the unsubstituted counterpart (**35**, 65% yield, 91.5:8.5 er). The results demonstrate that the steric and electronic properties of the substituents on the chloroarenes, despite their proximity to the sites of coupling reactions, do not play a determining role in the stereocontrol.

Prevalent in marine organisms, bis(indolyl)methanes have emerged as important scaffolds for bioactive compounds.^37^ By contrast, bis(benzothiophenyl)methanes have remained unexplored. Gratifyingly, the desymmetrization strategy is applicable to the synthesis of enantioenriched bis(indolyl)methane and bis(benzothiophenyl)methane bearing quaternary carbon centers (**36**–**39**) (Scheme 6D). Notably, desymmetrization of highly sterically congested substrates afford satisfactory stereocontrol (**38**, 92:8 er; **39**, 93.5:6.5 er), despite the reduced reactivity. The absolute configuration of ethyl ester **39** was determined by X-ray crystallography, which revealed the characteristic two-blade propeller-like conformation and the extended distance between the reaction sites and the carboxylate group compared to model substrate **5**. Evidently, the ionic chiral catalyst system is capable of adapting to the changes in the substrates’ core scaffolds while retaining high levels of asymmetric induction.

The high compatibility of the catalyst system to substrates bearing various nonionic substituents preluded that non-carbon substituted could be tolerated (Scheme 6E). Indeed, the remote desymmetrizing Suzuki–Miyaura reaction provides unified access to enantioenriched (86:14–96:4 er) α-tertiary alcohol derivatives (**40**–**42**), α-tertiary amide (**43**), and α-tertiary fluorine (**44**).^38^ Furthermore, a collection of diarylmethanes bearing chiral α-tertiary carbon was synthesized in good enantioselectivity (**45**–**47**, 46–55% yield, 89.5:10.5–93:7 er) by desymmetrization. The results further evidence that the ionic chiral catalyst does not rely on simple steric biasing between the geminal substituents to confer stereocontrol.

**Scheme 7:**
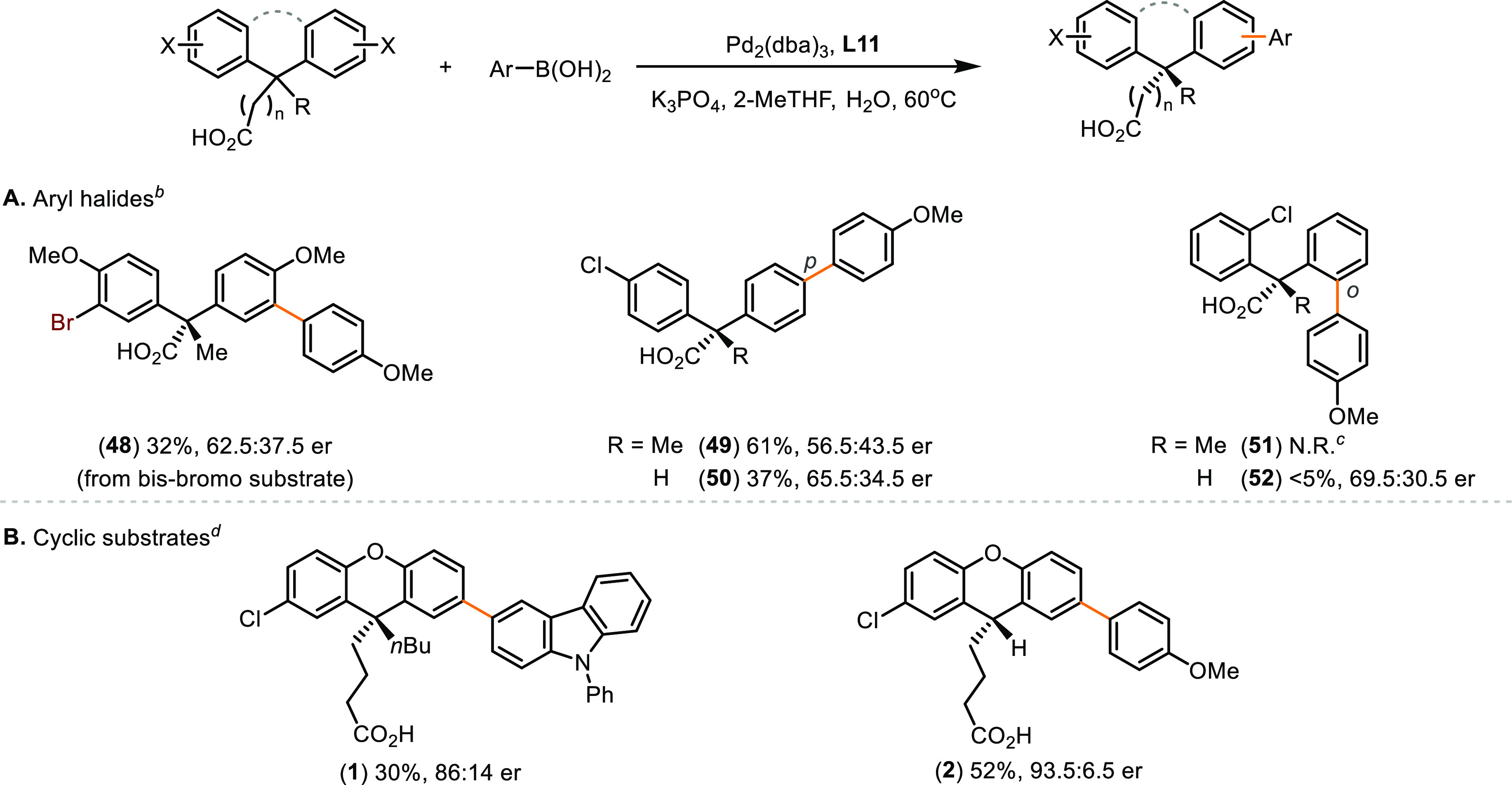
Effects of Aryl Halides and Cyclic Substrates Reaction conditions: bis(haloaryl)carboxylic acid (0.25 mmol), arylboronic acid (0.3 mmol), Pd_2_(dba)_3_ (1.0 mol %), **L11** (2.0 mol %), K_3_PO_4_ (1.25 mmol), 2-MeTHF (20 mL/mmol), H_2_O (2 mL/mmol), 60 °C, 18 h. Er was determined using HPLC–CSP. Isolated yields of ethyl esters reported. The absolute configurations of products were assigned by analogy to **39** (Scheme 6) and **70** (Scheme 8). 1^st^ run: 60 °C; 2^nd^ run: 80 °C. Formation of the coupling product was not observed. The authentic product marker was prepared from the ethyl ester substrate using SPhos. The major enantiomers of **1** and **2** from reactions using (*R*_a_, *S*)-**L11** are opposite to that obtained using (*S*_a_)-**L1** (Scheme 2A).

On the other hand, the identity and positions of halides have a profound impact (Scheme 7A). Replacing the chloroarenes for bromoarenes led to ineffective stereocontrol (**48**, 62.5:37.5 er). Additionally, the enantioselectivity diminished when the chloro groups were placed at the *para* positions with respect to quaternary (**49**, 56.5:43.5 er) and tertiary (**50**, 65.5:34.5 er) prochirality centers. Previous study by Miller and co-workers also highlighted the difficulty in achieving *para* desymmetrization of tertiary diarylmethanes in high enantioselectivity.^10a,39^ The reactions of *ortho* chloro substrates proceeded poorly, presumably due to increased steric hindrance at the reaction sites (**51** and **52**). We were intrigued to revisit the desymmetrization of cyclic substrates using **L11** as the ligand (Scheme 7B). Compared with reactions catalyzed by Pd–**L1** (Scheme 2A), reduced stereoselectivity was obtained for the quaternary substrate (**1**, 86:14 er), while the stereochemical outcome improved in the case of the tertiary substrate (**2**, 93.5:6.5 er).

### Desymmetrizing Sonogashira Reaction and Buchwald–Hartwig Amination

**Scheme 8:**
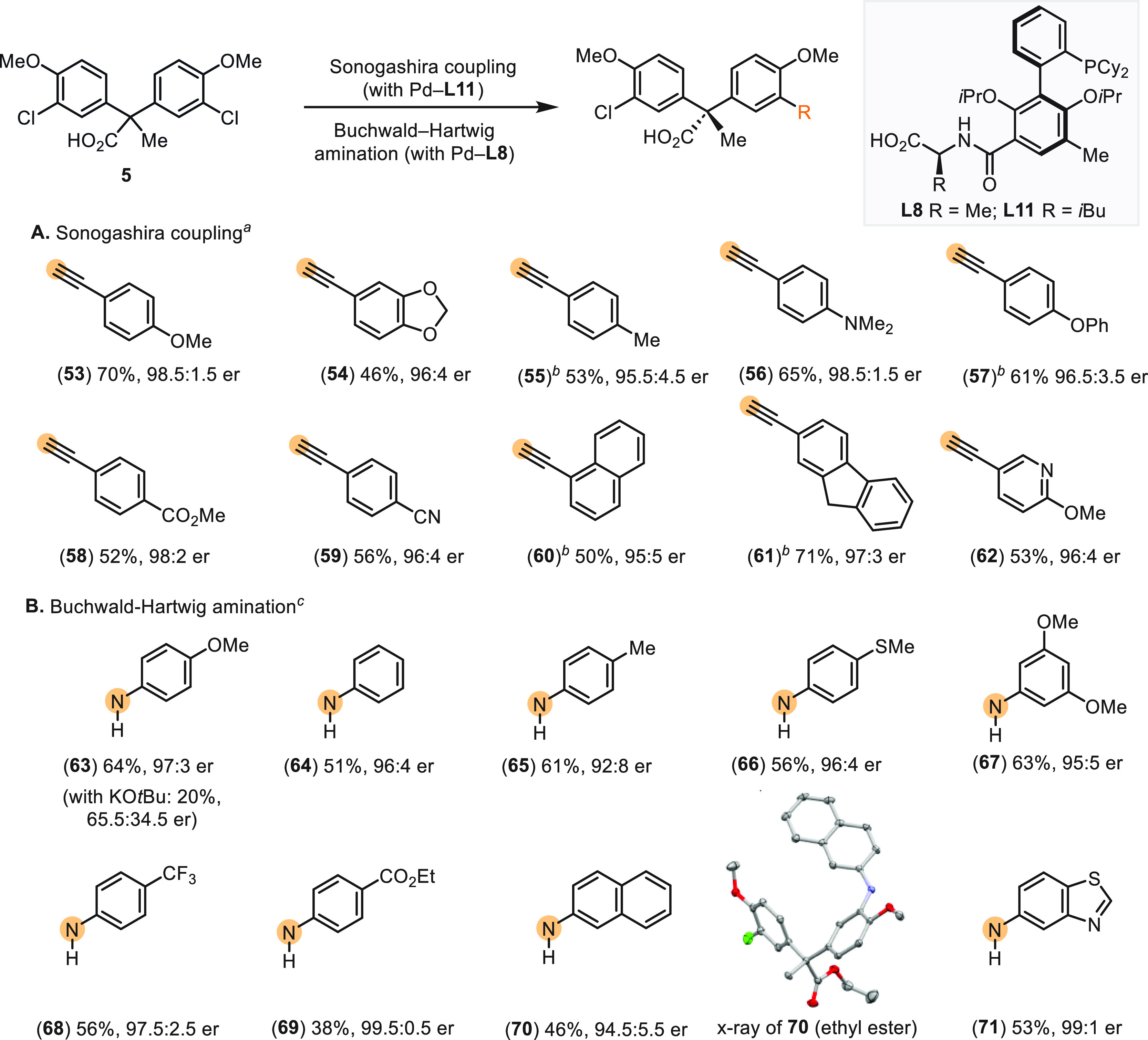
Desymmetrizing Sonogashira Reaction and Buchwald-Hartwig Amination Reaction conditions: **5** (0.25 mmol), alkynes (0.30 mmol), Pd_2_(dba)_3_ (1.0 mol %), **L11** (2.0 mol %), K_3_PO_4_ (1.25 mmol), 2-MeTHF (20 mL/mmol), H_2_O (2 mL/mmol), 60 °C, 24 h. Isolated yields reported. Isolated as ethyl ester. Reaction conditions: **12** (0.25 mmol), anilines (0.375 mmol), Pd_2_(dba)_3_ (1.0 mol %), **L8** (2.0 mol %), K_3_PO_4_ (1.5 mmol), 1,4-dioxane (10 mL/mmol), 110 °C, 18 h. Isolated yields of ethyl esters reported. Er was determined using HPLC–CSP. The absolute configuration of **70** was assigned by X-ray crystallography. The absolute configurations of other products were assigned by analogy to **39** (Scheme 6) and **70**.

In an effort to substantially diversify the coupling partners, we turned our attention to remote desymmetrization through Sonogashira reaction and Buchwald–Hartwig amination. These two reactions, along with Suzuki–Miyaura coupling, are the only ones among the top 20 most utilized chemical transformation in pharmaceutical research that are discovered within the past 50 years.^40^ First, we explored the desymmetrizing Sonogashira coupling^41^ using Pd–**L11** as the catalyst. The C(sp^2^)–C(sp) bond formation proceeds under similar conditions as the Suzuki–Miyaura coupling without the need of additional copper cocatalysts^42^ (Scheme 8A). Alkynyl groups bearing a broad spectrum of substituents can be readily installed with excellent stereocontrol of the quaternary carbon (**53**–**59**, 46–70% yield, 95.5:4.5–98.5:1.5 er). In addition, naphthyl ethynyl (**60**), fluorenyl ethynyl (**61**), and pyridyl ethynyl (**62**) participated in the transformation, affording the coupling products with high enantioselectivity (50–71% yield, 95:5–97:3 er).

Next, we explored desymmetrizing Buchwald-Hartwig amination (Scheme 8B).^43^ Our initial attempts using KO*t*Bu as base led to the formation of coupling product **63** in low enantiopurity (20% yield, 65.5:34.5 er with Pd–**L8**; 15% yield, 65:35 er with Pd–**L11**). Unlike the biphasic 2-MeTHF/water solvent system for Suzuki–Miyaura coupling and Sonogashira reactions, the Buchwald–Hartwig amination requires anhydrous conditions. High ionic strength of the reaction media resulting from the soluble KO*t*Bu presumably interrupts the ionic stereocontrol. Fortunately, the high level of enantioselectivity was restored by using K_3_PO_4_ as the base and 1,4-dioxane as the solvent (**63**, 64% yield, 97:3 er with Pd–**L8**). The limited solubility of K_3_PO_4_ in the organic solvent likely reduces the interference of ionizable reagents (see Tables S3–S5 in the SI). Under the optimal conditions, a broad range of anilines of varying electronic properties (**63**–**69**, 38–64% yield, 92:8–99.5:0.5 er), naphthylamine (**70**, 46% yield, 94.5:5.5 er) and aminobenzothiazole (**71**, 53% yield, 99:1 er), are all suitable coupling partners. Meanwhile, the absolute configuration of **70** was elucidated by X-ray crystallography, which also demonstrates the two-blade propeller-like conformation.

### Elucidating the Ionic Stereocontrol

**Scheme 9:**
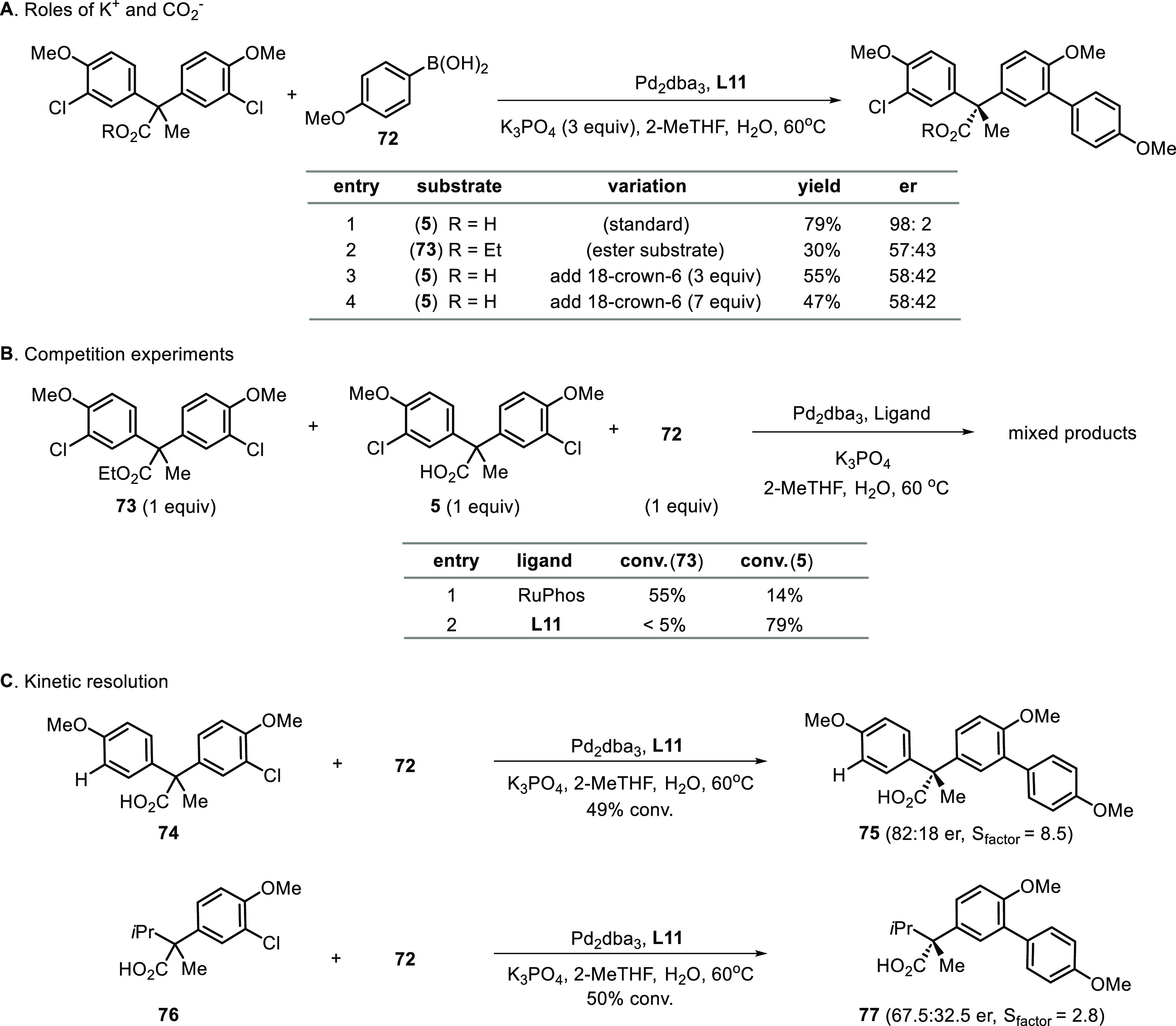
Control Experiments

To elucidate the contributing factors of stereocontrol, a series of control experiments was carried out. The pendent anionic carboxylate of the substrates and the potassium cation of the base are critical to the stereocontrol (Scheme 9A). In comparison with the model reaction between **5** and **72** (98:2 er, entry 1), significant reduction in enantioselectivity was observed using ethyl ester substrate **73** in the absence of the anionic carboxylate group (57:43 er, entry 2). Furthermore, sequestration of potassium by addition of 18-crown-6 into the reaction mixture of carboxylate substrate **5** also led to a sharp decrease in enantioselectivity (58:42 er, entries 3 and 4), which is intriguingly similar to that obtained for ester substrate **73** in the absence of 18-crown-6 (entry 2). The result suggests that without the bridging potassium cation, the electrostatic repulsive interactions between the carboxylate group of the substrate and the carboxylate group of the catalyst is ineffective in exerting effective stereocontrol. Therefore, both the carboxylate anion and potassium cation participated in the selectivity-determining step, presumably through contributing to attractive ionic substrate–catalyst interactions.

Moreover, competition experiments between the neutral substrate (**73**, COOEt) and the ionizable substrate (**5**, COO^–^K^+^ upon in situ deprotonation) unveil the intrinsic electronic effect of substrate’s distal anions on the selectivity-determining step (Scheme 9B). Interestingly, the reactions using RuPhos as ligand demonstrate four-fold preference for the coupling of the ester substrate than the carboxylate substrate (entry 1). Such selectivity favoring the neutral substrate over the anionic substrate likely results from the field effect engendered by the negatively charged carboxylate group rather than from steric effects, given the similar steric properties of carboxylate and ester groups and their distance from the reaction sites. Recent computational studies have also suggested that oriented electric fields have a profound effect on the rate of the oxidative addition step involving Pd–phosphine complexes.^44^

By contrast, when anionic ligand **L11** is utilized, the ionizable substrate’s pendent carboxylate group accelerates the selectivity-determining step, even though its intrinsic electronic effect should have resulted in rate reduction (entry 2). We surmised that the rate acceleration that favors the carboxylate substrate by the ionic catalyst is a result of attractive, not repulsive, substrate–catalyst electrostatic interactions.^45^ This conclusion is corroborated by the fact that enantiocontrol is achievable when the carboxyl group is tethered through flexible aliphatic linkers (**3**, **28** and **29**). Repulsive interactions, either electrostatic or steric, between the substrate’s carboxylate and the ligand’s carboxylate can be avoided without affecting the metal center’s action at the reaction site because of the conformational flexibility of the aliphatic linker.

Next, we investigated the role of diarylmethane scaffolds in the stereocontrol of desymmetrization. To this end, kinetic resolution of mono aryl chloride substrates **74** and **76** were carried out. Replacing the nonreacting aryl group of **74** by an isopropyl group of **76** led to a sharp decrease in the selectivity factor (Scheme 9C). We hypothesized that the distinctive two-blade propeller-like conformation of diarylmethane compounds^46^ is an integral part of the enantio-determining step of the desymmetrization reaction. This result also suggests that in the context of desymmetrization reactions, the presence of a secondary kinetic resolution of the primary products might contribute to the enantiopurity observed.^47^ We have demonstrated the enantiopurity upgrade of desymmetrization products through formation of doubly coupled products in the case of **21** (Scheme 5) and through a separate second coupling step (vide infra).

### Computational Investigations

Because of the distant enantiotopic reaction sites and the lack of directionality of ionic interactions,^48^ it becomes nonintuitive how the chiral catalyst exerts long-range asymmetric induction. In an effort to gain insight into the ionic stereocontrol, we performed computational studies of the desymmetrization reactions of cyclic and acyclic substrates using (*S*_a_)-**L1** and (*R*_a_, *S*)-**L11** based on an electrostatic steering model engaging attractive ionic substrate–catalyst interactions.

Oxidative addition of aryl chloride to palladium is an irreversible process under ambient conditions when electron-rich dicyclohexyl biaryl phosphine ligands are utilized.^49^ Compared with the subsequent transmetalation and reductive elimination steps that lead to C–C and C–N bond formation, reductive elimination to form aryl chloride from aryl palladium chloride is highly unfavorable.^50^ Therefore, we postulated that the oxidative addition step is selectivity–determining. This hypothesis is consistent with the observation that the stereoselectivity is influenced by the identity of halides (**23** vs **48**) but independent from the electronic and steric properties of various types of aryl, alkynyl, and amino coupling partners.

**Scheme 10:**
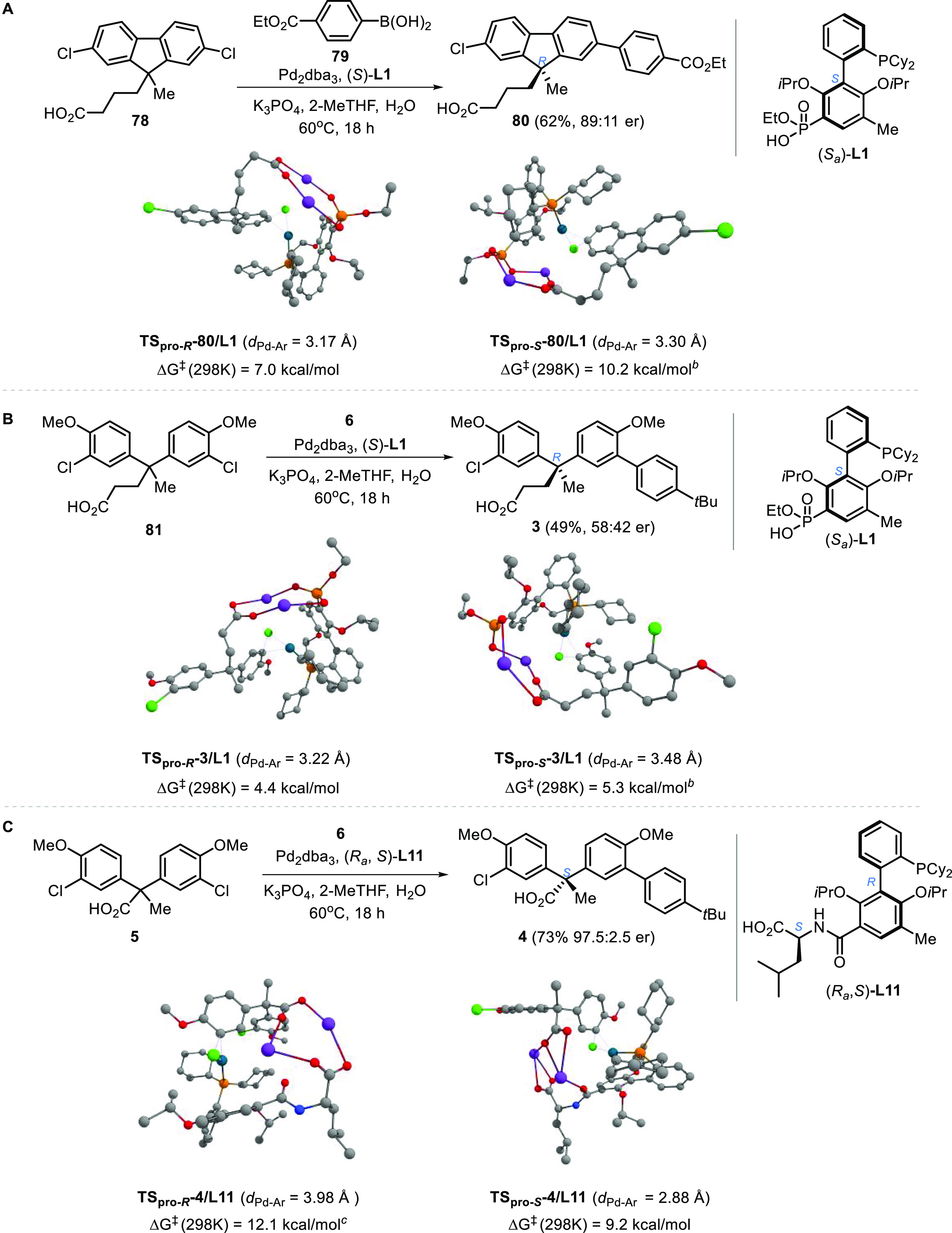
M11L/SDD(Pd)-6–311+G(d,p)/SMD Computed Transition States of Oxidative Addition Steps Carbon, gray; chlorine, green; nitrogen, blue; oxygen, red; potassium, purple; phosphine, orange; palladium, cyan; TS: transition state; *d*_Pd–Ar_: distance between Pd atom and centroid of the distal arene of ligand’s biaryl backbone. Pro-*R* pre-oxidative addition complex as reference. Pro-*S* pre-oxidative addition complex as reference (see Tables S6–S8 in the SI for details).

The Gibbs free energies of diastereomeric transition states were assessed based on DFT calculations for the oxidative addition steps involved in the desymmetrization of **78**, **81**, and **5** (Scheme 10). For Pd–(*S*_a_)-**L1**-catalyzed desymmetrization reactions, the computations revealed a relatively high ΔΔ*G^‡^*_298K_ for fluorene-derived **78** (3.2 kcal/mol) compared to acyclic substrate **81** (0.9 kcal/mol). The computational results are consistent with the experimental outcome that the desymmetrization of **78** is markedly more selective than **81** when (*S*_a_)-**L1** is utilized as the supporting ligand. Furthermore, the DFT calculations suggest that the formation of *R* products is favored, which agrees with the stereochemistry observed. Meanwhile, the computational studies reveal that the desymmetrization of acyclic substrate **5** using Pd–(*R*_a_, *S*)-**L11** as the catalyst proceeds in high selectivity (ΔΔ*G^‡^*_298K_ = 2.9 kcal/mol), favoring the formation of the *S* product, which is consistent with the experimental observations. We were delighted that the computed electrostatic steering models correctly predict the major enantiomers of products in all three cases, and we recognized that the models are primitive without considering the effect of hydration of ions and influence of other ionic species in the reaction media.

The lowest energy-optimized geometries reveal the cause of energetic differentiation between the diastereomeric transition states of oxidative addition steps. In agreement with the literature,^51^ we found that the oxidative addition proceeds through transition states where the Pd center is oriented in proximity to the distal arene of the ligands’ biaryl scaffold. Intriguingly, between each diastereomeric pair, the one with a shorter *d*_Pd–Ar_ (i.e., the distance between the Pd atom and centroid of the distal arene of ligand’s biaryl backbone) possesses a lower Gibbs free energy. This character is consistent with that the ligand’s distal arene functions as a labile coordinating π-donor, which stabilizes the transition state of oxidative addition at the metal center.^51^ The electrostatic steering of substrates is evidenced by the attractive interactions between the substrates’ carboxylate and the ligands’ anionic group via bridging potassium ions. On the basis of the computational results, two oxygen atoms of the phosphonate anion of **L1** and the carboxylate anion of **L11** jointly participate in ionic substrate-catalyst interactions through engaging bridging potassium cations. Importantly, transition state TS_pro-*S*_-**4**/**L11** revealed additional participation of the amide carbonyl through coordinating to one of the potassium ions. This characteristic explains the particularly short *d*_Pd–Ar_ (2.88 Å) of transition state TS_pro*-S*_*-***4**/**L11**. By contrast, the amide carbonyl does not engage the potassium ions through ion-dipole interactions in TS_pro*-R*_-**4**/**L11**, of which the *d*_Pd–Ar_ is much longer (3.98 Å). Plausibly, such synergistic electrostatic effects of terminal carboxylate and the amide group contributes to the effective stereocontrol over desymmetrization of acyclic substrates using Pd–**L11** as the catalyst.

### Derivation from Desymmetrizing Coupling

**Scheme 11:**
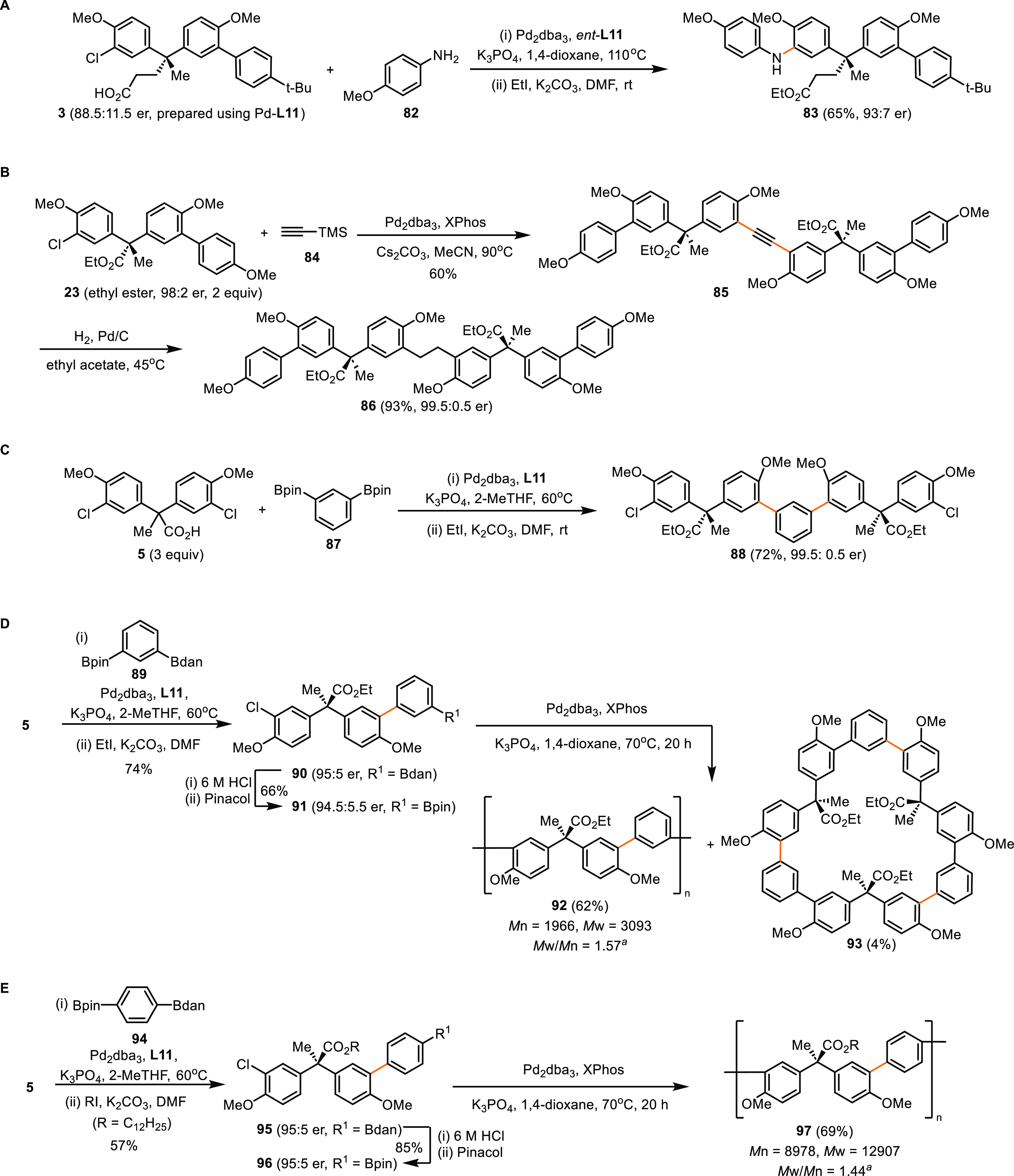
Derivatization of Desymmetrization Products *M*_n_, *M*_w_, and PDI (*M*_w_/*M*_n_) were determined by gel permeation chromatography (GPC). dan:naphthalene-1,8-diaminato.

The desymmetrization reactions decouple the creation of the quaternary chirality from the construction of scaffolds, which maximizes the benefits of cross-coupling reactions through diversifying a myriad of coupling partners. The method provides rapid assembly of molecules that project substituents and functional groups to widely spaced positions in 3D space. To illustrate this aspect, the Buchwald–Hartwig coupling between desymmetrization product **3** (88.5:11.5 er, prepared using Pd–(*R*_a_, *S*)-**L11**) and *p*-anisidine (**82**) was carried out using the enantiomeric catalyst (i.e., Pd–(*S*_a_, *R*)-**L11**), affording the bis-functionalized product **83** with improved enantiopurity (93:7 er, Scheme 11A). The kinetic resolution of **3** by the enantiomeric catalyst likely explains the upgraded enantiopurity of **83**. In addition, Sonogashira coupling of desymmetrization product **23** using trimethylsilylacetylene (**84**) as an acetylene surrogate afforded enantioenriched dimeric product **85** and then **86** upon hydrogenation (99.5:0.5 er), which are chiral analogs of dimeric diarylmethane compounds previously used for crystal engineering (Scheme 11B).^52^

In addition, it is practical to apply the desymmetrization strategy to dimerization of **5** using benzenediboronic acid bis(pinacol) ester (**87**) as the coupling partner, affording enantioenriched precursor (**88**) to dimeric bisphenol derivatives^53^ (Scheme 11C). Besides, the desymmetrization reactions using arenes bearing differentiated boron substituents (**89**: *meta*; **94**: *para*) yielded AB-type monomers **91** and **96**, which were subsequently converted into tactic oligomers **92** and **97**, respectively (Scheme 10D,E). Interestingly, a new type of macrocycle containing three *meta*-terphenyl units (**93**) was produced in the all-*syn* form as a minor product of oligomerization reaction of **91**.

## Conclusions

In summary, we have innovated a desymmetrizing cross-coupling strategy that establishes remote acyclic quaternary stereocenters. The evolution of a new class of ionic chiral ligands—amino acid-functionalized dialkylbiaryl phosphines—allows for designer dispositions of metal-binding phosphorous atom and substrate-binding carboxylate, which transmits the asymmetry precisely across a long distance to the prochirality center. This asymmetry-breaking process is applicable to a broad scope of diarylmethane scaffolds, a distinct characteristic rarely seen among remote desymmetrization reactions. Moreover, the catalyst system is capable of promoting the Suzuki–Miyaura reaction, Sonogashira reaction, and Buchwald–Hartwig amination—all are among the most utilized synthetic methods. A critical attribute of the long-range stereocontrol is the attractive electrostatic interactions engendered from the ionic chiral ligand, which steer the substrate and accelerate the selectivity-determining step. Computational investigations revealed that the ionic interactions of the amino acid moiety is further enhanced by the carbonyl group of the amide linker, thus shortening the distance between the Pd atom and the ligand’s distal arene and lowering the activation barrier of oxidative addition. The exquisite metal–ligand–substrate preorganization enabled by the amino acid-derived ionic ligands permits the effective asymmetric induction for acyclic quaternary stereocenters. We anticipate that the desymmetrization method paves the way for exploring the untapped chemical space of quaternary diarylmethanes. Moreover, the underlying mode of ionic stereocontrol elucidated through this study will guide future application of ionic chiral ligands to the creation of chirality elements that are difficult to construct using existing methods.

## Supporting Information Available

Experimental details and characterization data (PDF)

## References

[ref1] aBrandt J. R.; Salerno F.; Fuchter M. J. The added value of small-molecule chirality in technological applications. Nat. Rev. Chem. 2017, 1, 0045 10.1038/s41570-017-0045.

[ref2] Carreira E. M.; Fessard T. C. Four-Membered Ring-Containing Spirocycles: Synthetic Strategies and Opportunities. Chem. Rev. 2014, 114, 8257–8322. 10.1021/cr500127b. 25003801

[ref3] Barelier S.; Cummings J. A.; Rauwerdink A. M.; Hitchcock D. S.; Farelli J. D.; Almo S. C.; Raushel F. M.; Allen K. N.; Shoichet B. K. Substrate Deconstruction and the Nonadditivity of Enzyme Recognition. J. Am. Chem. Soc. 2014, 136, 7374–7382. 10.1021/ja501354q. 24791931 PMC4046767

[ref4] aDa Silva F.; Bret G.; Teixeira L.; Gonzalez C. F.; Rognan D. Exhaustive Repertoire of Druggable Cavities at Protein–Protein Interfaces of Known Three-Dimensional Structure. J. Med. Chem. 2019, 62, 9732–9742. 10.1021/acs.jmedchem.9b01184. 31603323

[ref5] aOdachowski M.; Bonet A.; Essafi S.; Conti-Ramsden P.; Harvey J. N.; Leonori D.; Aggarwal V. K. Development of Enantiospecific Coupling of Secondary and Tertiary Boronic Esters with Aromatic Compounds. J. Am. Chem. Soc. 2016, 138, 9521–9532. 10.1021/jacs.6b03963. 27384259 PMC5063455

[ref6] aTakeda M.; Takatsu K.; Shintani R.; Hayashi T. Synthesis of Quaternary Carbon Stereocenters by Copper-Catalyzed Asymmetric Allylic Substitution of Allyl Phosphates with Arylboronates. J. Org. Chem. 2014, 79, 2354–2367. 10.1021/jo500068p. 24601661

[ref7] aAmeen D.; Snape T. J. Chiral 1,1-diaryl compounds as important pharmacophores. Med. Chem. Commun. 2013, 4, 893–907. 10.1039/c3md00088e.

[ref8] Lewis C. A.; Chiu A.; Kubryk M.; Balsells J.; Pollard D.; Esser C. K.; Murry J.; Reamer R. A.; Hansen K. B.; Miller S. J. Remote Desymmetrization at Near-Nanometer Group Separation Catalyzed by a Miniaturized Enzyme Mimic. J. Am. Chem. Soc. 2006, 128, 16454–16455. 10.1021/ja067840j. 17177366

[ref9] aMetrano A. J.; Miller S. J. Peptide-Based Catalysts Reach the Outer Sphere through Remote Desymmetrization and Atroposelectivity. Acc. Chem. Res. 2019, 52, 199–215. 10.1021/acs.accounts.8b00473. 30525436 PMC6335614

[ref10] aKim B.; Chinn A. J.; Fandrick D. R.; Senanayake C. H.; Singer R. A.; Miller S. J. Distal Stereocontrol Using Guanidinylated Peptides as Multifunctional Ligands: Desymmetrization of Diarylmethanes via Ullman Cross-Coupling. J. Am. Chem. Soc. 2016, 138, 7939–7945. 10.1021/jacs.6b03444. 27254785 PMC5127171

[ref11] Shi H.; Herron A. N.; Shao Y.; Shao Q.; Yu J.-Q. Enantioselective remote *meta*-C–H arylation and alkylation via a chiral transient mediator. Nature 2018, 558, 581–585. 10.1038/s41586-018-0220-1. 29915312 PMC6026055

[ref12] aGenov G. R.; Douthwaite J. L.; Lahdenperä A. S. K.; Gibson D. C.; Phipps R. J. Enantioselective remote C-H activation directed by a chiral cation. Science 2020, 367, 1246–1251. 10.1126/science.aba1120. 32165586

[ref13] Wang H.; Li H.; Chen X.; Zhou C.; Li S.; Yang Y.-F.; Li G. Asymmetric Remote *meta*-C–H Activation Controlled by a Chiral Ligand. ACS Catal. 2022, 12, 13435–13445. 10.1021/acscatal.2c03187.

[ref14] Payne J. T.; Butkovich P. H.; Gu Y.; Kunze K. N.; Park H. J.; Wang D.-S.; Lewis J. C. Enantioselective Desymmetrization of Methylenedianilines via Enzyme-Catalyzed Remote Halogenation. J. Am. Chem. Soc. 2018, 140, 546–549. 10.1021/jacs.7b09573. 29294291 PMC5898188

[ref15] Xiong X.; Zheng T.; Wang X.; Tse Y.-L. S.; Yeung Y.-Y. Access to Chiral Bisphenol Ligands (BPOL) through Desymmetrizing Asymmetric *Ortho*-Selective Halogenation. Chem 2020, 6, 919–932. 10.1016/j.chempr.2020.01.009.

[ref16] Milo A.; Bess E. N.; Sigman M. S. Interrogating selectivity in catalysis using molecular vibrations. Nature 2014, 507, 210–214. 10.1038/nature13019. 24622199

[ref17] aXu P.; Huang Z. Catalytic reductive desymmetrization of malonic esters. Nat. Chem. 2021, 13, 634–642. 10.1038/s41557-021-00715-0. 34112991

[ref18] aLu Z.; Hu X.-D.; Zhang H.; Zhang X.-W.; Cai J.; Usman M.; Cong H.; Liu W.-B. Enantioselective Assembly of Cycloenones with a Nitrile-Containing All-Carbon Quaternary Center from Malononitriles Enabled by Ni Catalysis. J. Am. Chem. Soc. 2020, 142, 7328–7333. 10.1021/jacs.0c02075. 32255625

[ref19] Zeng X.-P.; Cao Z.-Y.; Wang Y.-H.; Zhou F.; Zhou J. Catalytic Enantioselective Desymmetrization Reactions to All-Carbon Quaternary Stereocenters. Chem. Rev. 2016, 116, 7330–7396. 10.1021/acs.chemrev.6b00094. 27251100

[ref20] aWang Y.; Zhang W.-Y.; Xie J.-H.; Yu Z.-L.; Tan J.-H.; Zheng C.; Hou X.-L.; You S.-L. Enantioselective Desymmetrization of Bisphenol Derivatives via Ir-Catalyzed Allylic Dearomatization. J. Am. Chem. Soc. 2020, 142, 19354–19359. 10.1021/jacs.0c09638. 33140959

[ref21] aGarcía-Urdiales E.; Alfonso I.; Gotor V. Enantioselective Enzymatic Desymmetrizations in Organic Synthesis. Chem. Rev. 2005, 105, 313–354. 10.1021/cr040640a. 15720156

[ref22] Shi B.-F.; Zhang Y.-H.; Lam J. K.; Wang D.-H.; Yu J.-Q. Pd (II)-Catalyzed Enantioselective C–H Olefination of Diphenylacetic Acids. J. Am. Chem. Soc. 2010, 132, 460–461. 10.1021/ja909571z. 20017549 PMC2806936

[ref23] Lou Y.; Wei J.; Li M.; Zhu Y. Distal Ionic Substrate–Catalyst Interactions Enable Long-Range Stereocontrol: Access to Remote Quaternary Stereocenters through a Desymmetrizing Suzuki–Miyaura Reaction. J. Am. Chem. Soc. 2022, 144, 123–129. 10.1021/jacs.1c12345. 34979078 PMC9549467

[ref24] aWillis M. C.; Powell L. H. W.; Claverie C. K.; Watson S. J. Enantioselective Suzuki Reactions: Catalytic Asymmetric Synthesis of Compounds Containing Quaternary Carbon Centers. Angew. Chem., Int. Ed. 2004, 43, 1249–1251. 10.1002/anie.200352648. 14991790

[ref25] aDavis H. J.; Mihai M. T.; Phipps R. J. Ion Pair-Directed Regiocontrol in Transition-Metal Catalysis: A *Meta*-Selective C–H Borylation of Aromatic Quaternary Ammonium Salts. J. Am. Chem. Soc. 2016, 138, 12759–12762. 10.1021/jacs.6b08164. 27626468

[ref26] aOhmatsu K.; Imagawa N.; Ooi T. Ligand-enabled multiple absolute stereocontrol in metal-catalysed cycloaddition for construction of contiguous all-carbon quaternary stereocentres. Nat. Chem. 2014, 6, 47–51. 10.1038/nchem.1796. 24345946

[ref27] aLi M.; Chia X. L.; Tian C.; Zhu Y. Mechanically planar chiral rotaxanes through catalytic desymmetrization. Chem 2022, 8, 2843–2855. 10.1016/j.chempr.2022.08.009.

[ref28] aYu J.; Long J.; Yang Y.; Wu W.; Xue P.; Chung L. W.; Dong X.-Q.; Zhang X. Iridium-Catalyzed Asymmetric Hydrogenation of Ketones with Accessible and Modular Ferrocene-Based Amino-phosphine Acid (f-Ampha) Ligands. Org. Lett. 2017, 19, 690–693. 10.1021/acs.orglett.6b03862. 28093919

[ref29] aNi H.; Chan W. L.; Lu Y. Phosphine-Catalyzed Asymmetric Organic Reactions. Chem. Rev. 2018, 118, 9344–9411. 10.1021/acs.chemrev.8b00261. 30204423

[ref30] Feng J.; Holmes M.; Krische M. J. Acyclic Quaternary Carbon Stereocenters via Enantioselective Transition Metal Catalysis. Chem. Rev. 2017, 117, 12564–12580. 10.1021/acs.chemrev.7b00385. 28910092 PMC5651685

[ref31] aZhu R.-Y.; Chen L.; Hu X.-S.; Zhou F.; Zhou J. Enantioselective synthesis of *P*-chiral tertiary phosphine oxides with an ethynyl group via Cu(I)-catalyzed azide–alkyne cycloaddition. Chem. Sci. 2020, 11, 97–106. 10.1039/C9SC04938J. 32110361 PMC7012078

[ref32] Ruiz-Castillo P.; Buchwald S. L. Applications of Palladium-Catalyzed C–N Cross-Coupling Reactions. Chem. Rev. 2016, 116, 12564–12649. 10.1021/acs.chemrev.6b00512. 27689804 PMC5070552

[ref33] We were unable to secure a source of tert-butyl lithium as a possible solution. In some cases, we were able to access enantioenriched ligands through classical chiral resolution (ref 27b).

[ref34] Li M.; Chia X. L.; Zhu Y. Tethered photocatalyst-directed palladium-catalysed C–H allenylation of *N*-aryl tetrahydroisoquinolines. Chem. Commun. 2022, 58, 4719–4722. 10.1039/D2CC01064J. 35297451

[ref35] On I. K. W.; Hong W.; Zhu Y. Remote control over both site-selectivity and atroposelectivity of Suzuki–Miyaura coupling through distal ionic interactions. Tetrahedron Lett. 2023, 119, 154408 10.1016/j.tetlet.2023.154408.

[ref36] Mohler L. K.; Czarnik A. W. α-Amino acid chelative complexation by an arylboronic acid. J. Am. Chem. Soc. 1993, 115, 7037–7038. 10.1021/ja00068a097.

[ref37] aSun F.-L.; Zheng X.-J.; Gu Q.; He Q.-L.; You S.-L. Enantioselective Synthesis of Unsymmetrical Triarylmethanes by Chiral Brønsted Acids. Eur. J. Org. Chem. 2010, 2010, 47–50. 10.1002/ejoc.200901164.

[ref38] aZheng Y.; Zhang S.; Low K.-H.; Zi W.; Huang Z. A Unified and Desymmetric Approach to Chiral Tertiary Alkyl Halides. J. Am. Chem. Soc. 2022, 144, 1951–1961. 10.1021/jacs.1c12404. 35076212

[ref39] aHsieh S.-Y.; Tang Y.; Crotti S.; Stone E. A.; Miller S. J. Catalytic Enantioselective Pyridine *N*-Oxidation. J. Am. Chem. Soc. 2019, 141, 18624–18629. 10.1021/jacs.9b10414. 31656070 PMC6926419

[ref40] Brown D. G.; Bostrom J. Analysis of Past and Present Synthetic Methodologies on Medicinal Chemistry: Where Have All the New Reactions Gone?. J. Med. Chem. 2016, 59, 4443–4458. 10.1021/acs.jmedchem.5b01409. 26571338

[ref41] Kanda K.; Koike T.; Endo K.; Shibata T. The first asymmetric Sonogashira coupling for the enantioselective generation of planar chirality in paracyclophanes. Chem. Commun. 2009, 1870–1872. 10.1039/b818904h. 19319429

[ref42] Gelman D.; Buchwald S. L. Efficient Palladium-Catalyzed Coupling of Aryl Chlorides and Tosylates with Terminal Alkynes: Use of a Copper Cocatalyst Inhibits the Reaction. Angew. Chem., Int. Ed. 2003, 42, 5993–5996. 10.1002/anie.200353015. 14679552

[ref43] aTakenaka K.; Itoh N.; Sasai H. Enantioselective Synthesis of *C*_2_-Symmetric Spirobilactams via Pd-Catalyzed Intramolecular Double *N*-Arylation. Org. Lett. 2009, 11, 1483–1486. 10.1021/ol900016g. 19254000

[ref44] aJoy J.; Stuyver T.; Shaik S. Oriented External Electric Fields and Ionic Additives Elicit Catalysis and Mechanistic Crossover in Oxidative Addition Reactions. J. Am. Chem. Soc. 2020, 142, 3836–3850. 10.1021/jacs.9b11507. 31994390

[ref45] aFanourakis A.; Docherty P. J.; Chuentragool P.; Phipps R. J. Recent Developments in Enantioselective Transition Metal Catalysis Featuring Attractive Noncovalent Interactions between Ligand and Substrate. ACS Catal. 2020, 10, 10672–10714. 10.1021/acscatal.0c02957. 32983588 PMC7507755

[ref46] aKoshima H.; Ding K.; Chisaka Y.; Matsuura T. Generation of Chirality in a Two-Component Molecular Crystal of Acridine and Diphenylacetic Acid and Its Absolute Asymmetric Photodecarboxylating Condensation. J. Am. Chem. Soc. 1996, 118, 12059–12065. 10.1021/ja961106q.

[ref47] aHayashi T.; Niizuma S.; Kamikawa T.; Suzuki N.; Uozumi Y. Catalytic asymmetric synthesis of axially chiral biaryls by palladium-catalyzed enantioposition-selective cross-coupling. J. Am. Chem. Soc. 1995, 117, 9101–9102. 10.1021/ja00140a041.

[ref48] aYe X.; Tan C.-H. Enantioselective transition metal catalysis directed by chiral cations. Chem. Sci. 2021, 12, 533–539. 10.1039/D0SC05734G. PMC8179005 34163782

[ref49] aShen X.; Hyde A. M.; Buchwald S. L. Palladium-Catalyzed Conversion of Aryl and Vinyl Triflates to Bromides and Chlorides. J. Am. Chem. Soc. 2010, 132, 14076–14078. 10.1021/ja107481a. 20857936 PMC2975586

[ref50] aJones D. J.; Lautens M.; McGlacken G. P. The emergence of Pd-mediated reversible oxidative addition in cross coupling, carbohalogenation and carbonylation reactions. Nat. Catal. 2019, 2, 843–851. 10.1038/s41929-019-0361-0.

[ref51] aBarder T. E.; Buchwald S. L. Insights into Amine Binding to Biaryl Phosphine Palladium Oxidative Addition Complexes and Reductive Elimination from Biaryl Phosphine Arylpalladium Amido Complexes via Density Functional Theory. J. Am. Chem. Soc. 2007, 129, 12003–12010. 10.1021/ja073747z. 17850080

[ref52] Marti T.; Peterson B. R.; Fürer A.; Mordasini-Denti T.; Zarske J.; Jaun B.; Diederich F.; Gramlich V. Macrotricyclic Steroid Receptors by Pd^0^-Catalyzed Cross-Coupling Reactions: Dissolution of cholesterol in aqueous solution and investigations of the principles governing selective molecular recognition of steroidal substrates. Helv. Chim. Acta 1998, 81, 109–144. 10.1002/hlca.19980810112.

[ref53] Chen D.; Kannan K.; Tan H.; Zheng Z.; Feng Y.-L.; Wu Y.; Widelka M. Bisphenol Analogues Other Than BPA: Environmental Occurrence, Human Exposure, and Toxicity—A Review. Environ. Sci. Technol. 2016, 50, 5438–5453. 10.1021/acs.est.5b05387. 27143250

