# Supplementary material for: Amino Acid-Derived Ionic Chiral Catalysts Enable Desymmetrizing Cross-Coupling to Remote Acyclic Quaternary Stereocenters
Source: J Am Chem Soc. 2023 Jul 20;145(30):16796–811. doi: 10.1021/jacs.3c04877 (PMC10401725; doi:10.1021/jacs.3c04877)

Supporting Information

Amino Acid-Derived Ionic Chiral Catalysts Enable Desymmetrizing Cross-Coupling to Remote Acyclic Quaternary Stereocenters

Junqiang Wei,[1] Vincent Gandon*[,2] and Ye Zhu*[,1]

[1]Department of Chemistry, Faculty of Science, National University of Singapore, 3 Science Drive 3, Singapore 117543.

*Correspondence: chmzhu@nus.edu.sg

[2]Institut de Chimie Moléculaire et des Matériaux d'Orsay (UMR CNRS 8182), Paris-Saclay University, bâtiment Henri Moissan, 17 avenue des sciences, 91400 Orsay, France.

*Correspondence: vincent.gandon@universite-paris-saclay.fr

Table of Contents

List of acronyms and abbreviations 3
General information 5
Preparation of ligands 7
Preparation of substrates 15
The general procedure of Suzuki cross-coupling desymmetrization reaction of xanthene substrates 43
Characterization data of desymmetrization products of xanthene substrates 44
Optimization of Suzuki cross-coupling reaction conditions 46
General procedure for Suzuki cross-coupling desymmetrization reactions of acyclic substrates 48
Characterization data of desymmetrization products of Suzuki cross-coupling reactions ... 49
The general procedure of Sonogashira cross-coupling desymmetrization reaction. 82
Characterization data of desymmetrization products of Sonogashira cross-coupling reaction 83
Optimization of reaction conditions for Buchwald–Hartwig cross-coupling 90
The general procedure for Buchwald–Hartwig cross-coupling desymmetrization reactions 93
Characterization data of desymmetrization products of Buchwald–Hartwig cross-coupling reactions 94
Control experiments 100
Competition experiments 101
Kinetic resolution 102
Computational study 104
Derivatization of desymmetrization products 140
References 154
NMR spectra 156
HPLC traces 290
X-ray crystallography data 369

List of acronyms and abbreviations

**Ac**	acetyl
**Bu**	butyl
**Bn**	benzyl
**Cy**	cyclohexyl
**bpin**	boronic acid pinacol
**dan**	naphthalene-1,8-diaminato
**DCM**	dichloromethane
**DIBAL-H**	diisobutylaluminum hydride solution
**DIPEA**	*N*,*N*-diisopropylethylamine
**DMF**	*N*,*N*-dimethylformamide
**DMSO**	dimethyl sulfoxide
**EA**	ethyl acetate
**EDC**	*N*-(3-dimethylaminopropyl)-*N′*-ethylcarbodiimide
**Et**	ethyl
**HCl**	hydrochloride
**HOBt**	1-hydroxybenzotriazole
**I_2**	iodine
**LDA**	lithium diisopropylamide
**Me**	methyl
**MeCN**	acetonitrile

**2-MeTHF**	2-methyltetrahydrofuran
**NFSI**	*N*-fluorobenzenesulfonimide
**NCS**	*N*-chlorosuccinimide
**Ph**	phenyl
**PIFA**	[bis(trifluoroacetoxy)iodo]benzene
**pin**	pinacolato
**PLC**	preparative thin layer chromatography
**Pr**	propyl
**RuPhos**	2-dicyclohexylphosphino-2′,6′-diisopropoxybiphenyl
**TFA**	trifluoroacetic acid
**THF**	tetrahydrofuran
**Tf**	trifluoromethylsulfonyl
**TMS**	trimethylsilyl
**Tol**	methylphenyl
**XPhos**	2-dicyclohexylphosphino-2′,4′,6′-triisopropylbiphenyl

General information

Materials: Commercially available reagents and solvents were used as received (Sigma-Aldrich, Strem Chemicals, and BLDpharm). Commercial dry solvents (Aldrich Sure/Seal™) were sparged with nitrogen before being used in catalytic reactions. Solvents used for column chromatography were analytical grade.

Methods: Unless otherwise noted, all experiments were set up under an atmosphere of nitrogen in a glovebox or using standard Schlenk techniques. Reactions were monitored by thin-layer chromatography (TLC), gas chromatography (GC), or nuclear magnetic resonance (NMR) analysis. Flash column chromatography was performed using Tsingdao silica gel (60, particle size 300-400 mesh). Yields refer to isolated yields after flash column chromatography purification.

Characterization: Products were characterized utilizing nuclear magnetic resonance (NMR), mass spectrometry (MS), high-performance liquid chromatography (HPLC), and optical rotation. NMR spectra were recorded on a Bruker DPX 400 spectrometer at 300 MHz for ^{1}H NMR, 101 MHz for ^{13}C NMR, and 162 MHz for ^{31}P NMR or on a Bruker DPX 500 spectrometer at 500 MHz for ^{1}H NMR, 126 MHz for ^{13}C NMR, 202 MHz for ^{31}P NMR and 471 MHz for ^{19}F NMR in $CDCl_3$ with tetramethylsilane as internal standard. Chemical shifts were reported relative to tetramethylsilane (0 ppm) for ^{1}H NMR and relative to $CDCl_3$ (77.0 ppm) for ^{13}C NMR. ^{19}F spectra were calibrated from the external standard ($CFCl_3$: 0 ppm). ^{31}P spectra were calibrated from the external standard (85 wt% phosphoric acid: 0 ppm). NMR data are reported as chemical shift (parts per million, ppm),

multiplicity (s = singlet, d = doublet, t = triplet, q = quartet, m = multiplet), coupling constant (Hz), and integration. HPLC analysis was performed on a Shimadzu i-series HPLC system equipped with a photodiode array (PDA) detector and Chiralcel and Chiralpak columns (0.46 cm∅× 25 cm). The wavelength that is an apex in the spectrum was selected for analysis of the enantiomeric ratio (er) of each compound. Optical rotation ($[\alpha]_D^T$, deg•cm^3•g^{-1}•dm^{-1}) was measured on a Jasco DIP-1000 Digital Polarimeter at λ=589 nm in the given solvent at the indicated concentration (c, g/100 mL) and temperature (T, °C). X-ray diffraction was performed on Bruker D8 Venture single crystal X-ray diffractometer.

Preparation of ligands

1. General Remarks

L6 (*R*)-2'-(dicyclohexylphosphaneyl)-2,6-diisopropoxy-5-methyl-[1,1'-biphenyl]-3-carbo-xylic acid was synthesized from RuPhos following procedures previously reported.[1,2,3]

2. The general procedure for the synthesis of amino acid-drived ligands[4]

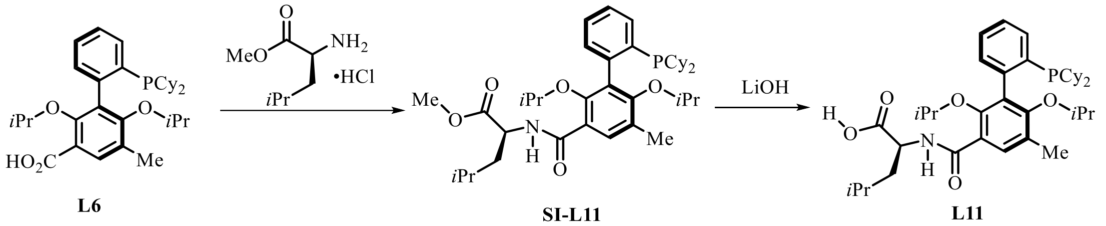

L11: (*R*)2'-(dicyclohexylphosphaneyl)-2,6-diisopropoxy-5-methyl-[1,1'-biphenyl]-3-carbonyl-L-leucine

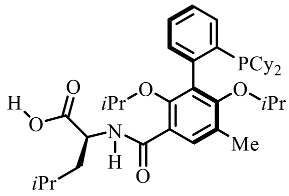

To **L6**-(*R*)-2'-(dicyclohexylphosphaneyl)-2,6-diisopropoxy-5-meth-yl-[1,1'-biphenyl]-3-carboxylic acid (52.4 mg, 0.1 mmol), DIPEA (51.6 mg, 0.4 mmol), HOBt·H_2O (27 mg, 0.2 mmol), and methyl L-leucinate hydrochloride (22 mg, 0.1 mmol) were added DCM (1 mL). The mixture was cooled to 0 °C and EDC·HCl (38.4 mg, 0.2 mmol) was then added to the solution. After that, the resulting reaction mixture was stirred at room temperature for 24 h. The reaction was quenched with H_2O, then the mixture was extracted with EA (5 mL X 3) three times. The combined organic phases were washed with brine, dried over Na_2SO_4, and concentrated in vacuo. The crude product **SI-L11** was used directly in the next step without further purification.

Under the N_2 atmosphere, **SI-L11** (crude material) was dissolved in 0.5 mL THF and 0.5 mL MeOH, followed by the addition of 2 M LiOH $_{(aq.)}$ (0.5 mL, 1 mmol). The resulting reaction mixture was stirred at room temperature for 24 h. After the reaction was completed, removal of the organic phase was performed under reduced pressure, and water was added and extracted with DCM (5 mL X 3) three times. The combined organic phases were washed with brine, dried over Na_2SO_4, and concentrated in vacuo. The crude materials were purified by flash column chromatography with hexane and acetone (5:1 – 2:1) to give the desired product **L11**, 32 mg in 50% yield over two steps as a white solid. ^{1}H NMR (500 MHz, Acetone-d_6) δ 8.27 (d, J = 8.1 Hz, 1H), 7.89 (s, 1H), 7.78 – 7.75 (m, 1H), 7.52 – 7.42 (m, 3H), 4.87– 4.83 (m, 1H), 3.88 – 3.81 (m, 2H), 2.30 (s, 3H), 2.19 – 2.07 (m, 3H), 1.92 (d, J = 12.3 Hz, 1H), 1.82 – 1.61 (m, 10H), 1.50 – 1.43 (m, 1H), 1.40 – 1.14 (m, 10H), 1.01 – 0.95 (m, 15H), 0.85 (d, J = 6.1 Hz, 3H). ^{13}C NMR (101 MHz, Acetone-d_6) δ 174.4, 166.0, 165.9, 158.4, 153.7 (d, J = 1.7 Hz), 143.2 (d, J = 32.6 Hz), 139.3 (d, J = 19.4 Hz), 133.2(d, J = 3.3 Hz), 133.1 (d, J = 5.9 Hz), 132.7, 132.4 (d, J = 7.3 Hz), 128.7, 128.1 (d, J = 6.2 Hz), 123.7 (d, J = 3.7 Hz), 77.6, 75.5, 43.2, 35.3, 35.1, 34.4 (d, J = 15.3 Hz), 33.5 (d, J = 21.4 Hz), 31.8 (d, J = 15.6 Hz), 30.80 (d, J = 15.4 Hz), 29.20, 28.8 (d, J = 13.9 Hz), 28.1, 27.8, 27.3 (d, J = 9.8 Hz), 25.9, 25.7, 23.3, 22.8, 22.4, 21.0, 17.3. ^{31}P NMR (202 MHz, Acetone-d_6) δ -8.93. HRMS (m/z, ESI): Calcd. for Chemical Formula: $C_{38}H_{57}NO_5P^+$ $[M+H]^+$: 638.3969, Found: 638.3974.

3. Characterization and Spectra of amino acid-drived ligands L6-L14

L6-(*R*)-2'-(dicyclohexylphosphaneyl)-2,6-diisopropoxy-5-methyl-[1,1'-biphenyl]-3-carboxylic acid

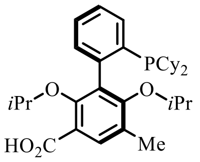

White solid, 250 mg, 75%. ^{1}H NMR (500 MHz, Acetone-d_6) δ 11.09 (s, 1H), 7.79 (s, 1H), 7.72 – 7.68 (m, 1H), 7.46 – 7.40 (m, 3H), 3.95 – 3.86 (m, 2H), 2.27 (s, 3H), 2.14 – 2.04 (m, 2H), 1.98 – 1.87 (m, 2H), 1.77 – 1.68 (m, 2H), 1.65 – 1.51 (m, 6H), 1.28 – 1.20 (m, 6H), 1.18 – 1.09 (m, 2H), 1.05 – 1.00 (m, 2H), 0.94 (d, *J* = 6.2 Hz, 3H), 0.90 (dd, *J* = 6.2, 2.6 Hz, 6H), 0.87 (d, *J* = 6.1 Hz, 3H). ^{31}P NMR (162 MHz, Acetone-d_6) δ -8.76. ^{13}C NMR (126 MHz, Acetone-d_6) δ 167.0, 160.4, 155.2, 143.1 (d, *J* = 32.6 Hz), 140.0 (d, *J* = 19.3 Hz), 133.9, 133.7 (d, *J* = 3.1 Hz), 133.4 (d, *J* = 5.7 Hz), 129.3, 129.1, 128.8, 120.6, 78.9, 76.5, 35.1 (d, *J* = 7.9 Hz), 35.0 (d, *J* = 7.9 Hz), 33.1 (d, *J* = 4.2 Hz), 33.0 (d, *J* = 3.9 Hz), 28.8 (d, *J* = 4.9 Hz), 28.7 (d, *J* = 5.1 Hz), 28.5 (d, *J* = 4.0 Hz), 28.4 (d, *J* = 4.2 Hz). 27.8, 23.5, 23.3, 22.7, 22.5, 17.7.

L7-(*R*)-(2'-(dicyclohexylphosphaneyl)-2,6-diisopropoxy-5-methyl-[1,1'-biphenyl]-3-carbonyl)-glycine

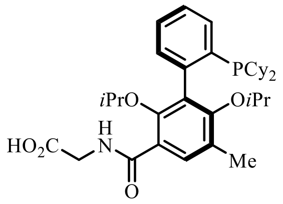

Prepared from methyl glycine hydrochloride and **L6**, two steps, 25 mg in 43% yield as a white solid. ^{1}H NMR (400 MHz, Acetone-d_6) δ 8.33 (s, 1H), 7.85 (d, *J* = 0.9 Hz, 1H), 7.73 – 7.69 (m, 1H), 7.44 – 7.41 (m, 3H), 4.28 – 4.09 (m, 2H), 3.88 – 3.76 (m, 2H), 2.27 (s, 3H), 2.20 – 2.10 (m, 2H), 1.96 (d, *J* = 7.5 Hz, 2H), 1.79 – 1.69 (m, 2H), 1.68 – 1.52 (m, 6H), 1.32 – 1.18 (m, 7H), 1.18 – 1.12 (m, 1H), 1.09 – 0.98 (m, 2H),0.93 – 0.85 (m, 12H). ^{13}C NMR (101 MHz, Acetone-d_6) δ 171.5, 166.1, 158.4, 153.5, 143.0 (d, J = 32.7 Hz), 139.3 (d, *J* = 19.4 Hz), 133.0 (d, *J* = 6.2 Hz), 132.9, 132.4, 132.2 (d, *J* = 7.3

Hz), 128.4, 127.9 (d, J = 4.3 Hz), 123.5, 77.4, 75.5, 41.8, 34.5 (d, J = 2.6 Hz), 34.3 (d, J = 2.5 Hz), 32.6 (d, J = 2.4 Hz), 32.4 (d, J = 2.5 Hz), 29.7, 28.2 (d, J = 5.0 Hz), 28.0 (d, J = 4.7 Hz), 27.9 (d, J = 5.9 Hz), 27.8 (d, J = 6.1 Hz). 27.1, 22.8, 22.6, 21.7, 21.4, 17.1. ^{31}P NMR (202 MHz, Acetone-d_6) δ -8.89. HRMS (m/z, ESI): Calcd. for Chemical Formula: $C_{34}H_{49}NO_5P^+$ $[M+H]^+$: 582.3343, Found: 582.3349.

L8-(*R*)-(2'-(dicyclohexylphosphaneyl)-2,6-diisopropoxy-5-methyl-[1,1'-biphenyl]-3-carbonyl)-L-alanine

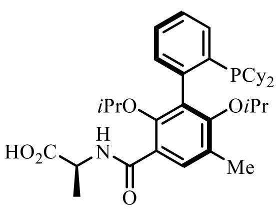

Prepared from methyl L-alanine hydrochloride and **L6**, two steps, 35 mg in 59% yield as a white solid. ^{1}H NMR (500 MHz, Acetone-d_6) δ 8.38 (d, J = 6.6 Hz, 1H), 7.87 (s, 1H), 7.76 – 7.73 (m, 1H), 7.49 – 7.40 (m, 3H), 4.68 – 4.62 (m, 1H), 3.89 – 3.72 (m, 2H), 2.29 (s, 3H), 2.20 – 2.14 (m, 2H), 2.04 – 1.96 (m, 2H), 1.78 (d, J = 10.6 Hz, 2H), 1.70 – 1.57 (m, 6H), 1.49 (d, J = 7.1 Hz, 3H), 1.31 – 1.25 (m, 6H), 1.21 – 1.14 (m, 2H), 1.13 – 1.02 (m, 2H), 0.95 – 0.92 (m, 9H), 0.87 (d, J = 6.1 Hz, 3H). ^{13}C NMR (126 MHz, Acetone-d_6) δ 174.7, 164.8, 157.6, 152.4, 142.2 (d, J = 32.7 Hz), 138.5 (d, J = 19.6 Hz), 132.2 (d, J = 5.9 Hz), 132.1 (d, J = 3.1 Hz), 131.6, 131.4 (d, J = 7.3 Hz), 127.7, 127.0 (d, J = 23.5 Hz). 123.1, 76.2, 74.8, 48.8, 33.8 (d, J = 15.8 Hz), 33.5 (d, J = 15.5 Hz), 32.1 (d, J = 20.4 Hz), 31.5 (d, J = 17.9 Hz), 29.4, 29.2, 27.5 (d, J = 8.4 Hz), 27.4 (d, J = 7.3 Hz), 27.2 (d, J = 7.0 Hz), 27.0 (d, J = 8.5 Hz), 26.3, 21.9 (d, J = 19.5 Hz), 20.9 (d, J = 23.5 Hz), 18.1, 16.3. ^{31}P NMR (202 MHz, Acetone-d_6) δ -9.04. HRMS (m/z, ESI): Calcd. for Chemical Formula: $C_{35}H_{51}NO_5P^+$ $[M+H]^+$: 596.3499, Found: 596.3507.

L9-(*R*)-2'-(dicyclohexylphosphaneyl)-2,6-diisopropoxy-5-methyl-[1,1'-biphenyl]-3-carbonyl)-L-valine

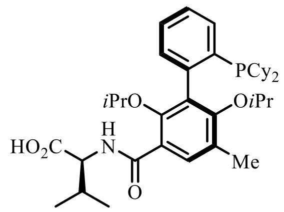

Prepared from methyl L-valine hydrochloride and **L6**, two steps, 14 mg in 22% yield as a white solid. ^{1}H NMR (500 MHz, Acetone-d_6) δ 8.35 (d, J = 7.9 Hz, 1H), 7.91 (s, 1H), 7.76 – 7.70 (m, 1H), 7.48 – 7.38 (m, 3H), 4.47 – 4.44 (m, 1H), 3.93 – 3.77 (m, 2H), 2.26 (s, 3H), 2.24 – 2.10 (m, 2H), 2.05 – 1.99 (m, 2H), 1.91 (d, J = 11.7 Hz, 1H), 1.78 – 1.70 (m, 3H), 1.65 (d, J = 12.0 Hz, 4H), 1.54 – 1.47 (m, 1H), 1.20 – 1.12 (m, 10H), 0.98 (d, J = 6.6 Hz, 9H), 0.95 – 0.93 (m, 6H), 0.89 (d, J = 6.1 Hz, 3H). ^{13}C NMR (126 MHz, Acetone-d_6) δ 175.9, 165.6, 165.5, 157.2, 152.8,142.5 (d, J = 32.7 Hz), 138.4 (d, J = 19.8 Hz), 132.3, 132.2 (d, J = 5.9 Hz), 131.3 (d, J = 7.3 Hz), 127.6, 126.8 (d, J = 28.0 Hz), 123.4, 76.6, 74.5, 60.0, 33.9 (t, J = 17.0 Hz), 31.9 (t, J = 17.4 Hz), 31.1 (d, J = 14.3 Hz), 29.6 (d, J = 14.8 Hz), 27.5 (d, J = 12.9 Hz), 27.2 (d, J = 10.7 Hz), 27.0 (t, J = 7.9 Hz). 26.4 (d, J = 8.0 Hz), 21.9 (d, J = 8.6 Hz), 21.4, 20.3, 19.4, 18.4, 16.5. ^{31}P NMR (202 MHz, Acetone-d_6) δ -8.88. HRMS (m/z, ESI): Calcd. for Chemical Formula: $C_{37}H_{55}NO_5P^+$ $[M+H]^+$: 624.3812, Found: 624.3821.

L10-(*R*)-2'-(dicyclohexylphosphaneyl)-2,6-diisopropoxy-5-methyl-[1,1'-biphenyl]-3-carbonyl-L-isoleucine

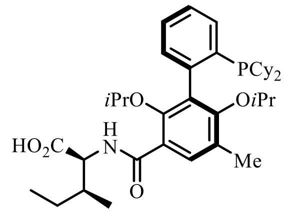

Prepared from methyl L-isoleucine hydrochloride and **L6**, two steps, 11 mg in 17% yield as a white solid. ^{1}H NMR (500 MHz, Acetone-d_6) δ 11.42 (s, 1H), 8.31 (d, J = 8.2 Hz, 1H), 7.88 (s, 1H), 7.77 – 7.75 (m, 1H), 7.51 – 7.39 (m, 3H), 4.77 (dd, J = 8.2, 5.2 Hz, 1H), 3.93 – 3.84 (m, 2H), 2.30 (s, 3H), 2.20 – 2.12 (m, 1H), 2.05 – 1.96 (m, 2H), 1.89 (d, J = 12.7 Hz, 1H), 1.83 – 1.71 (m, 3H), 1.70 – 1.58 (m, 5H),

1.48 (d, *J* = 13.5 Hz, 1H), 1.41 – 1.31 (m, 4H), 1.30 – 1.14 (m, 8H), 1.02 – 0.95 (m, 12H), 0.92 (d, *J* = 6.3 Hz, 3H), 0.89 (d, *J* = 6.2 Hz, 3H). ^{13}C NMR (126 MHz, Acetone-d_6) δ 172.4, 165.1, 157.4, 152.8, 142.4 (d, *J* = 32.8 Hz), 138.3 (d, *J* = 19.6 Hz), 132.4 (d, *J* = 3.4 Hz), 132.1 (d, *J* = 5.9 Hz), 131.8, 127.7, 127.1 (d, *J* = 12.3 Hz), 123.0, 76.8, 74.5, 56.4, 38.1, 34.1 (d, *J* = 15.9 Hz), 33.7 (d, *J* = 15.6 Hz), 32.2 (d, *J* = 19.9 Hz), 30.7 (d, *J* = 15.1 Hz), 29.8 (d, *J* = 15.1 Hz), 29.5, 27.6 (d, *J* = 13.3 Hz), 27.1 (d, *J* = 5.0 Hz), 26.9 (d, *J* = 9.7 Hz), 26.3 (d, *J* = 9.8 Hz), 25.3, 21.9 (d, *J* = 10.5 Hz), 21.3, 19.9, 16.4, 15.1, 11.1. ^{31}P NMR (202 MHz, Acetone-d_6) δ -8.76. HRMS (m/z, ESI): Calcd. for Chemical Formula: $C_{38}H_{57}NO_5P^+$ $[M+H]^+$: 638.3969, Found: 638.3973.

L12-(*R*)-2'-(dicyclohexylphosphaneyl)-2,6-diisopropoxy-5-methyl-[1,1'-biphenyl]-3-carbonyl-L-phenylalanine

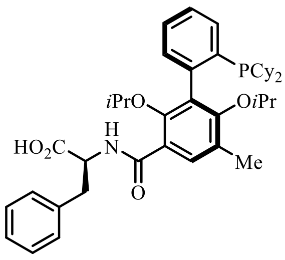

Prepared from methyl L-phenylalanine hydrochloride and **L6**, two steps, 10 mg in 15% yield as a white solid. ^{1}H NMR (500 MHz, Acetone-d_6) δ 8.34 (d, *J* = 6.9 Hz, 1H), 7.86 (s, 1H), 7.77 – 7.69 (m, 1H), 7.46 – 7.41 (m, 3H), 7.32 – 7.18 (m, 5H), 4.98 (q, *J* = 6.4 Hz, 1H), 3.89 – 3.84 (m, 1H), 3.82 – 3.77 (m, 1H), 3.33 (dd, *J* = 13.8, 6.3 Hz, 1H), 3.17 (dd, *J* = 13.9, 6.2 Hz, 1H), 2.30 (s, 3H), 2.15 – 2.09 (m, 2H), 2.02 – 1.97 (m, 1H), 1.94 (d, *J* = 10.9 Hz, 1H), 1.74 (d, *J* = 11.6 Hz, 2H), 1.69 – 1.63 (m, 2H), 1.62 – 1.60 (m, 2H), 1.55 – 1.50 (m, 2H), 1.30 – 1.18 (m, 7H), 1.15 – 1.08 (m, 2H), 1.04 – 1.00 (m, 1H), 0.93 (d, *J* = 6.1 Hz, 3H), 0.88 (dd, *J* = 8.2, 6.2 Hz, 6H), 0.79 (d, *J* = 6.2 Hz, 3H). ^{13}C NMR (126 MHz, Acetone-d_6) δ 172.3, 165.1, 157.5, 152.8, 142.3 (d, *J* = 32.6 Hz), 138.5 (d, *J* = 19.7 Hz), 137.3, 132.2 (d, *J* = 4.3 Hz), 132.1, 131.6, 131.4 (d, *J* = 7.3 Hz), 129.4, 128.2, 127.6, 127.0, 126.5, 122.9, 76.7, 74.6, 54.0, 53.9, 33.9 (d, *J* = 15.8 Hz), 33.5 (d, *J* = 15.6 Hz), 32.0

(d, J = 19.8 Hz), 31.2 (d, J = 17.2 Hz), 29.5, 27.3 (t, J = 12.0 Hz), 27.1 (d, J = 7.3 Hz), 27.0 (d, J = 8.9 Hz), 26.3 (d, J = 3.1 Hz), 22.0, 21.8, 21.1, 20.2, 16.4. ^{31}P NMR (202 MHz, Acetone-d_6) δ -8.89. HRMS (m/z, ESI): Calcd. for Chemical Formula: $C_{41}H_{55}NO_5P^+$ $[M+H]^+$: 672.3812, Found: 672.3811.

L13-(*R*)-2'-(dicyclohexylphosphaneyl)-2,6-diisopropoxy-5-methyl-[1,1'-biphenyl]-3-carbonyl)-L-tryptophan

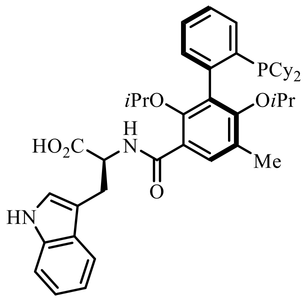

Prepared from methyl L-tryptophan hydrochloride and **L6**, two steps, 28 mg in 39% yield as a white solid. ^{1}H NMR (500 MHz, Acetone-d_6) δ 10.03 (s, 1H), 8.35 (d, J = 6.9 Hz, 1H), 7.86 (s, 1H), 7.75 – 7.62 (m, 2H), 7.47 – 7.31 (m, 4H), 7.18 (s, 1H), 7.06 (t, J = 7.7 Hz, 1H), 6.98 (t, J = 7.5 Hz, 1H), 5.08 – 5.04 (m, 1H), 3.85 – 3.74 (m, 2H), 3.45 (dd, J = 14.7, 6.4 Hz, 1H), 3.32 (dd, J = 14.7, 6.1 Hz, 1H), 2.28 (s, 3H), 2.14 – 2.09 (m, 2H), 1.97 – 1.88 (m, 2H), 1.76 – 1.69 (m, 2H), 1.64 (d, J = 12.1 Hz, 2H), 1.59 – 1.46 (m, 4H), 1.28 – 1.16 (m, 8H), 1.09 – 1.00 (m, 2H), 0.89 (dd, J = 19.5, 6.2 Hz, 6H), 0.82 (d, J = 6.1 Hz, 3H), 0.76 (d, J = 6.2 Hz, 3H). ^{13}C NMR (126 MHz, Chloroform-d) δ 167.1, 158.1, 152.8, 141.9 (d, J = 31.3 Hz), 136.1, 132.3, 132.0, 131.1, 127.8, 127.5, 127.1, 123.2, 121.8, 119.4, 118.7, 111.1, 110.1, 75.1, 53.4, 34.0 (d, J = 15.1 Hz), 33.6 (d, J = 13.9 Hz), 32.1 (d, J = 17.6 Hz), 29.7, 29.1 (d, J = 10.0 Hz), 28.5 (d, J = 6.3 Hz), 27.8, 27.6, 27.5, 27.3, 26.4, 22.5 (d, J = 14.5 Hz), 21.7, 21.0, 17.0, 14.1. ^{31}P NMR (202 MHz, Acetone-d_6) δ -9.07. HRMS (m/z, ESI): Calcd. for Chemical Formula: $C_{43}H_{56}N_2O_5P^+$ $[M+H]^+$: 711.3921, Found: 711.3923.

L14-(*S*)-2'-(dicyclohexylphosphaneyl)-2,6-diisopropoxy-5-methyl-[1,1'-biphenyl]-3-carbonyl-L-leucine

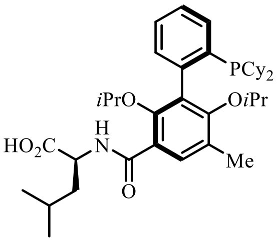

Prepared from methyl L-leucine hydrochloride and **(*S*)-L6**, two steps, 32 mg in 50% yield as a white solid. ^{1}H NMR (500 MHz, Acetone-d_6) δ 11.33 (s, 1H), 8.27 (d, *J* = 8.2 Hz, 1H), 7.87 (s, 1H), 7.77 – 7.75 (m, 1H), 7.47 – 7.45 (m, 3H), 4.89 – 4.76 (m, 1H), 3.87 – 3.82 (m, 2H), 2.28 (s, 3H), 2.23 – 2.05 (m, 3H), 1.93 – 1.87 (m, 1H), 1.81 – 1.61 (m, 10H), 1.47 – 1.43 (m, 1H), 1.40 – 1.13 (m, 10H), 1.00 – 0.92 (m, 15H), 0.84 (d, *J* = 6.2 Hz, 3H). ^{13}C NMR (126 MHz, Acetone-d_6) δ 174.2, 158.1, 153.5, 143.0 (d, *J* = 31.8 Hz), 133.0 (d, *J* = 3.1 Hz), 132.9, 132.5, 128.6, 128.0, 123.6, 77.4, 75.3, 51.2, 51.1, 43.0 (d, *J* = 3.6 Hz), 34.9 (d, *J* = 15.1 Hz), 33.9 (d, *J* = 16.4 Hz), 33.2 (d, *J* = 25.2 Hz), 31.4 (d, *J* = 12.6 Hz), 30.5 (d, *J* = 15.1 Hz), 28.9 (d, *J* = 6.3 Hz), 28.6 (d, *J* = 15.1 Hz), 27.9 (d, *J* = 6.3 Hz), 27.8 (d, *J* = 11.3 Hz), 27.6 (d, *J* = 7.6 Hz), 27.1(d, *J* = 11.9 Hz), 25.5, 23.1, 22.6 (d, *J* = 9.6 Hz), 22.2 (d, *J* = 11.3 Hz), 20.8, 17.1. ^{31}P NMR (202 MHz, Acetone-d_6) δ -8.89. HRMS (m/z, ESI): Calcd. for Chemical Formula: $C_{38}H_{57}NO_5P^+$ $[M+H]^+$: 638.3969, Found: 638.3970.

Preparation of substrates

1. The general procedure A for the synthesis of substrates[5]

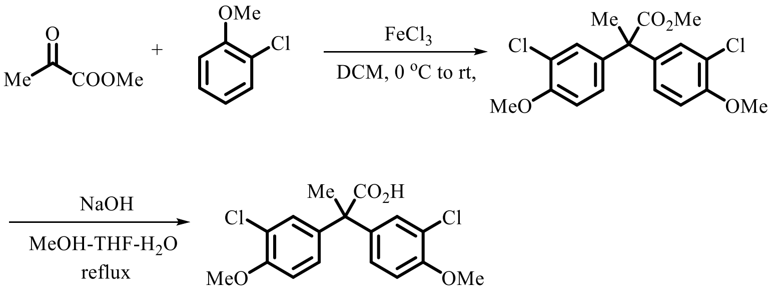

Methyl 2,2-bis(3-chloro-4-methoxyphenyl)-propanoate

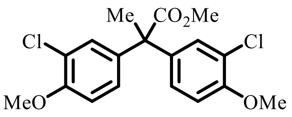

Under N_2 atmosphere, methyl pyruvate (5.61 g, 55 mmol) and $FeCl_3$ (8.1 g, 50 mmol) were dissolved in anhydrous 30 mL DCM, then 2-Chloroanisole (7.1, 50 mmol) was added dropwise at 0°C. After addition, the resulting reaction mixture was stirred for 18 h at room temperature. The reaction was quenched with H_2O and extracted with DCM (40 mL X 3) three times. The combined organic phases were washed with brine, dried over Na_2SO_4, and concentrated in vacuo. The crude materials were purified by flash column chromatography to give the desired product, 8.44 g in 91% yield as a light-yellow oil.

2,2-bis(3-chloro-4-methoxyphenyl)-propanoic acid

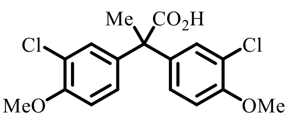

Under the N_2 atmosphere, methyl 2,2-bis(3-chloro-4-methoxyphenyl)-propanoate (2.54 g, 6.9 mmol) was dissolved in 10 mL THF, 10 mL MeOH, and 10 mL H_2O followed by the addition of NaOH (2.76 g, 40 mmol). The resulting reaction mixture was heated to reflux and stirred at this temperature for 24 h. Then, the reaction was cooled

to room temperature, the mixture was neutralized with 1 M HCl $_{(aq.)}$ to pH 2 – 3 and extracted with EA (20 mL X 3) three times. The combined organic phases were washed with brine, dried over Na_2SO_4, and concentrated in vacuo. The crude materials were purified by flash column chromatography with hexane and acetone (5:1 – 2:1) to give the desired product, 2.25 g in 92% yield as a white solid. ^1H NMR (500 MHz, Chloroform-*d*) δ 7.27 (d, *J* = 2.4 Hz, 2H), 7.11 (dd, *J* = 8.7, 2.4 Hz, 2H), 6.88 (d, *J* = 8.7 Hz, 2H), 3.90 (s, 6H), 1.88 (s, 3H). ^{13}C NMR (126 MHz, Chloroform-*d*) δ 179.9, 154.2, 136.6, 129.9, 127.5, 122.4, 111.8, 56.3, 54.9, 27.0.

2,2-bis(3-chloro-4-isopropoxyphenyl)-propanoic acid

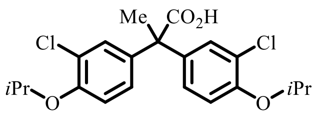

Prepared from 1-chloro-2-(propan-2-yloxy)-benzene and methyl pyruvate, two steps, 310 mg in 23% yield as a white solid. ^1H NMR (500 MHz, Chloroform-*d*) δ 7.26 (d, *J* = 2.7 Hz, 2H), 7.08 – 7.06 (m, 2H), 6.87 (d, *J* = 8.7 Hz, 2H), 4.58 – 4.51 (m, 2H), 1.86 (s, 3H), 1.38 (d, *J* = 6.1 Hz, 12H). ^{13}C NMR (101 MHz, Chloroform-*d*) δ 180.5, 152.7, 136.4, 129.9, 127.3, 123.7, 114.9, 72.0, 54.8, 26.9, 22.1. HRMS (m/z, ESI): Calcd. for Chemical Formula: $C_{21}H_{23}Cl_2O_4^-$ [M-H]$^-$: 409.0979, Found: 409.0976.

2,2-bis(4-butoxy-3-chlorophenyl)-propanoic acid

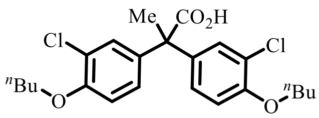

Prepared from 4-(2-chlorophenoxy)-1-butanol and methyl pyruvate, two steps, 303 mg in 20% yield as a yellowish oil. ^1H NMR (500 MHz, Chloroform-*d*) δ 7.25 (d, *J* = 2.4 Hz, 2H), 7.08 (dd, *J* = 8.7, 2.4 Hz, 2H), 6.85 (d, *J* = 8.7 Hz, 2H), 4.02 (t, *J* = 6.4 Hz, 4H), 1.87 (s, 3H), 1.85 – 1.78 (m, 4H), 1.57 – 1.48 (m, 4H), 0.98 (t, *J* = 7.4 Hz, 6H). ^{13}C NMR (101 MHz, Chloroform-*d*) δ 179.4, 153.7, 136.2, 129.7, 127.3, 122.6, 112.7, 68.8,

54.7, 31.2, 26.9, 19.2, 13.8. HRMS (m/z, ESI): Calcd. for Chemical Formula: $C_{23}H_{27}Cl_2O_4^-$ [M-H]$^-$: 437.1292, Found: 437.1286.

2,2-bis(3-chloro-4-methoxy-5-methylphenyl)-propanoic acid

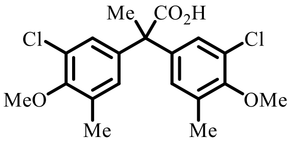

Prepared from 1-chloro-2-methoxy-3-methylbenzene and methyl pyruvate, two steps, 190 mg in 42% yield as a white solid. ^{1}H NMR (500 MHz, Chloroform-*d*) δ 7.09 (d, *J* = 2.4 Hz, 2H), 6.95 (dd, *J* = 2.4, 2H), 3.83 (s, 6H), 2.28 (s, 6H), 1.86 (s, 3H). ^{13}C NMR (126 MHz, Chloroform-*d*) δ 178.7, 153.4, 139.6, 132.9, 129.2, 127.6, 127.5, 60.2, 55.1, 27.0, 16.6. HRMS (m/z, ESI): Calcd. for Chemical Formula: $C_{19}H_{19}Cl_2O_4^-$ [M-H]$^-$: 381.0666, Found: 381.0667.

2,2-bis(5-chlorobenzo[b]thiophen-3-yl)-propanoic acid

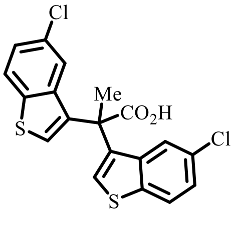

Prepared from 5-chlorobenzothiophene and methyl pyruvate, two steps, 232 mg in 82% yield over two steps as a white solid. ^{1}H NMR (400 MHz, Acetone-d_6) δ7.98 (d, *J* = 0.5 Hz, 1H), 7.96 (d, *J* = 0.5 Hz, 1H), 7.76 (d, *J* = 0.5 Hz, 2H), 7.56 (dd, *J* = 2.1, 0.5 Hz, 2H), 7.31 (dd, *J* = 2.1, 0.5 Hz, 1H), 7.29 (dd, *J* = 2.1, 0.5 Hz, 1H), 2.24 (s, 3H). ^{13}C NMR (101 MHz, Acetone-d_6) δ 178.3, 144.5, 144.2, 141.7, 134.7, 132.4, 129.6, 129.5, 128.8, 56.5, 30.3. HRMS (m/z, ESI): Calcd. for Chemical Formula: $C_{19}H_{11}Cl_2O_2S_2^-$ [M-H]$^-$: 404.9583, Found: 404.9583.

2,2-bis(3-bromo-4-methoxyphenyl)propanoic acid

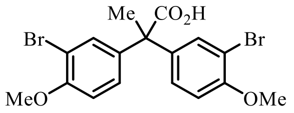

Prepared from 2-bromoanisole and methyl pyruvate, two steps, 310 mg in 59% yield as a white solid. ^{1}H NMR (500 MHz, Chloroform-*d*) δ 7.44 (dd, *J* = 2.4, 1.0 Hz, 2H), 7.16 (ddd, *J* = 8.7, 2.5, 1.0 Hz, 2H), 6.85 (dd, *J* = 8.7, 0.9 Hz, 2H), 3.89 (s, 6H), 1.88 (s, 3H). ^{13}C NMR (126 MHz, Chloroform-*d*) δ 179.2, 155.0, 136.9, 132.7, 130.6, 128.2, 111.4, 56.3, 54.6, 27.0. HRMS (m/z, ESI): Calcd. for Chemical Formula: $C_{17}H_{15}Br_2O_4^-$ [M-H]$^-$: 440.9343, Found: 440.9339.

2. The general procedure B for the synthesis of substrates[5,6,7]

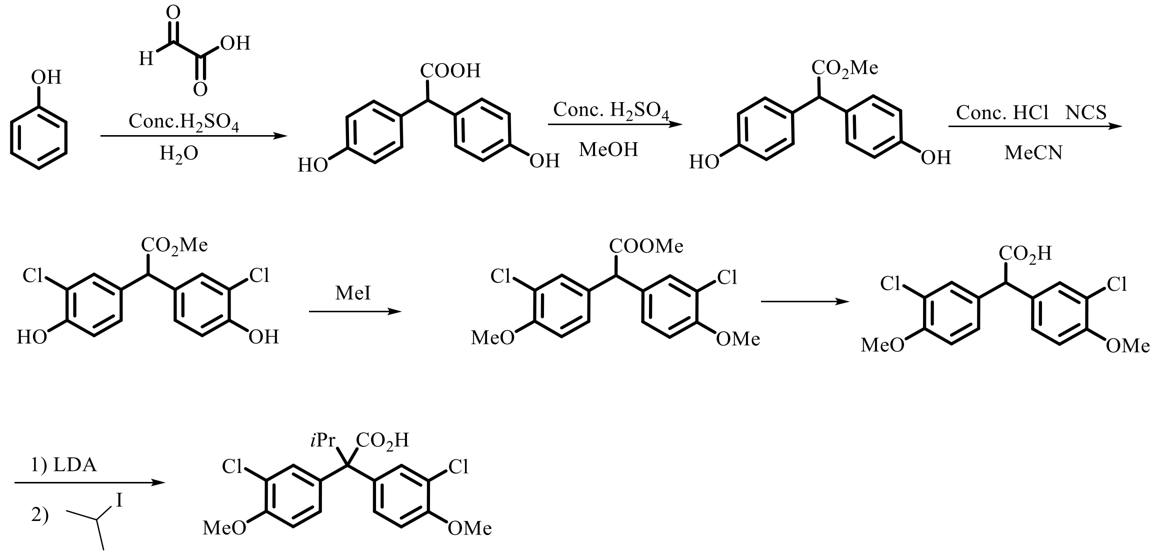

Methyl 2,2-bis(4-hydroxyphenyl)-acetate

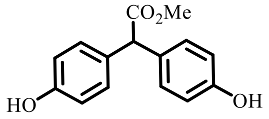

Under the N_2 atmosphere, phenol (5.7 g, 60.64 mmol) and 2-oxoacetic acid (50% in H_2O, 4.48 g, 30.32 mmol) were added into 3 mL H_2O, then Sulfuric acid (95%, 2.7 mL) was added dropwise at 0°C. After addition, the resulting mixture was stirred for 5 h at 45°C. The reaction was quenched with H_2O and extracted with EA (40 mL X 3)

three times. The combined organic phases were washed with brine, dried over Na_2SO_4, and concentrated in vacuo. The crude material was used directly in the next step without further purification.

The above crude product was dissolved into 80 mL MeOH, then Sulfuric acid (95%, 0.5 mL) was added dropwise at 0°C. The reaction solution was then heated to reflux and stirred for 24 h at this temperature. Finally, the reaction was cooled to room temperature, quenched with H_2O and extracted with EA (40 mL X 3) three times. The combined organic phases were washed with brine, dried over Na_2SO_4, and concentrated in vacuo. The crude materials were purified by flash column chromatography with hexane and acetone (30:1 – 5:1) to give the desired product, 2.14 g in 27% yield over two steps as a light-yellow oil.

Methyl 2,2-bis(3-chloro-4-hydroxyphenyl)-acetate

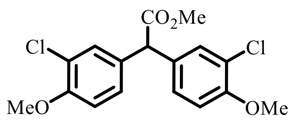

Methyl 2,2-bis(4-hydroxyphenyl)-acetate (2.14 g, 8.3 mmol) was dissolved in 30 mL MeCN, then hydrochloric acid (37%, 5.19 mL, 62.25 mmol) was added dropwise at 0°C. After addition, NCS (2.22 g, 16.6 mmol) was added to the reaction, and the resulting reaction mixture was stirred at room temperature for 24 h. The reaction was quenched with H_2O and extracted with EA (40 mL X 3) three times. The combined organic phases were washed with brine, dried over Na_2SO_4, and concentrated in vacuo. The crude material was used directly in the next step without further purification.

The above crude ester and K_2CO_3 (4.58 g, 33.2 mmol) were added into 30 mL DMF, then MeI (3.53 g, 24.9 mmol) was added dropwise at 0°C. After addition, the resulting reaction mixture was stirred for 18 h at 60°C. The reaction was cooled to room temperature and quenched with H_2O, then

extracted with EA (40 mL X 3) three times. The combined organic phases were washed with brine, dried over Na_2SO_4, and concentrated in vacuo. The crude materials were purified by flash column chromatography with hexane and acetone (100:1 – 20:1) to give the desired product, 1.21 g in 41% yield over two steps as a colorless oil.

2,2-bis(3-chloro-4-methoxyphenyl)-acetic acid

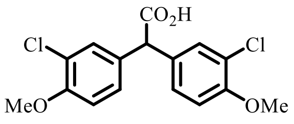

Methyl 2,2-bis(3-chloro-4-hydroxyphenyl)-acetate (1.21 g, 3.4 mmol) was dissolved in 15 mL THF, 10 mL H_2O and 15 mL MeOH, followed by the addition of NaOH (1.36 g, 34 mmol). The resulting reaction mixture was heated to reflux and stirred at this temperature for 24 h. After the reaction was completed, the mixture was neutralized with 1.0 M HCl $_{(aq.)}$ to pH 2 – 3 and extracted with EA (20 mL X 3) three times. The combined organic phases were washed with brine, dried over Na_2SO_4, and concentrated in vacuo. The crude materials were purified by flash column chromatography with hexane and acetone (5:1 – 2:1) to give the desired product, 2,2-bis(3-chloro-4-methoxyphenyl)-acetic acid, 1.04 g in 90% yield as a white solid. ^{1}H NMR (400 MHz, Chloroform-*d*) δ 7.32 (d, *J* = 2.3 Hz, 2H), 7.17 (dd, *J* = 8.6, 2.3 Hz, 2H), 6.89 (d, *J* = 8.6 Hz, 2H), 4.88 (s, 1H), 3.89 (s, 6H). ^{13}C NMR (101 MHz, Chloroform-*d*) δ 177.3, 154.5, 130.6, 130.3, 127.8, 122.7, 112.1, 56.2, 54.5.

2,2-bis(3-chloro-4-methoxyphenyl)-3-methylbutanoic acid

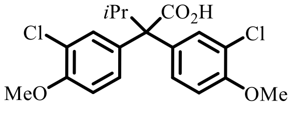

2,2-Bis(3-chloro-4-methoxyphenyl)-acetic acid (304 mg, 0.89 mmol) was dissolved in 8 mL THF under N_2 atmosphere, and LDA (0.98 mL, 2.0 M in Hexanes, 1.96 mmol) was added dropwise at -78°C. The resulting reaction mixture was

stirred for 30 minutes at 45°C. After that, 2-iodopropane (197 mg, 1.16 mmol) was added dropwise into the reaction mixture, the mixture was then stirred for 15 h at 45°C. The reaction was cooled to room temperature and quenched with H_2O, then extracted with EA (40 mL X 3) three times. The combined organic phases were washed with brine, dried over Na_2SO_4, and concentrated in vacuo. The crude materials were purified by flash column chromatography with hexane and acetone (5:1–2:1) to give the desired product, 191 mg in 56% yield as a white solid. ^1H NMR (400 MHz, Chloroform-*d*) δ 7.31 (d, *J* = 2.4 Hz, 2H), 7.15 (dd, *J* = 8.7, 2.4 Hz, 2H), 6.87 (d, *J* = 8.7 Hz, 2H), 3.91 (s, 6H), 3.19 (p, *J* = 6.6 Hz, 1H), 0.81 (d, *J* = 6.6 Hz, 6H). ^{13}C NMR (101 MHz, Chloroform-*d*) δ 177.9, 154.0, 132.5, 132.0, 129.9, 121.5, 110.9, 63.5, 56.1, 30.7, 18.7. HRMS (m/z, ESI): Calcd. for Chemical Formula: $C_{19}H_{19}Cl_2O_4^-$ [M-H]$^-$: 381.0666, Found: 381.0664.

2,2-bis(3-chloro-4-methoxyphenyl) hexanoic acid

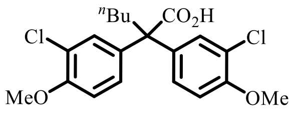

Prepared from 2,2-bis(3-chloro-4-methoxyphenyl)-acetic acid, 208 mg in 59% yield as a white solid. ^1H NMR (500 MHz, Chloroform-*d*) δ 7.30 (d, *J* = 2.4 Hz, 2H), 7.14 (dd, *J* = 8.7, 2.4 Hz, 2H), 6.86 (d, *J* = 8.7 Hz, 2H), 3.90 (s, 6H), 2.30 – 2.22 (m, 2H), 1.32 – 1.27 (m, 2H), 1.06 – 1.00 (m, 2H), 0.84 (t, *J* = 7.3 Hz, 3H). ^{13}C NMR (101 MHz, Chloroform-*d*) δ 179.3, 153.9, 135.4, 130.5, 128.4, 121.9, 111.4, 58.6, 56.1, 37.9, 27.4, 23.1, 13.9. HRMS (m/z, ESI): Calcd. for Chemical Formula: $C_{20}H_{21}Cl_2O_4^-$ [M-H]$^-$: 395.0822, Found: 395.0822.

2,2-bis(3-chloro-4-methoxyphenyl)-4-methylpentanoic acid

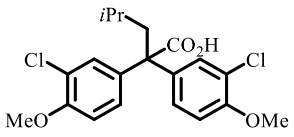

Prepared from 2,2-bis(3-chloro-4-methoxyphenyl)-acetic acid, 150 mg in 54% yield as a white solid. ^1H NMR (500 MHz, Chloroform-*d*) δ

7.34 (d, *J* = 2.4 Hz, 2H), 7.19 (dd, *J* = 8.7, 2.4 Hz, 2H), 6.85 (d, *J* = 8.7 Hz, 2H), 3.90 (s, 6H), 2.25 (d, *J* = 5.6 Hz, 2H), 1.41 – 1.33 (m, 1H), 0.71 (d, *J* = 6.6 Hz, 6H). ^{13}C NMR (126 MHz, Chloroform-*d*) δ 178.4, 153.9, 135.9, 130.6, 128.4, 121.9, 111.4, 58.0, 56.1, 46.5, 25.2, 23.9. HRMS (m/z, ESI): Calcd. for Chemical Formula: $C_{20}H_{21}Cl_2O_4^-$ [M-H]$^-$: 395.0822, Found: 395.0822.

2,2-bis(3-chloro-4-methoxyphenyl)-3-phenylpropanoic acid

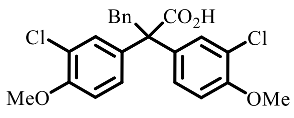

Prepared from 2,2-bis(3-chloro-4-methoxyphenyl)-acetic acid, 180 mg in 60% yield as a white solid. ^{1}H NMR (500 MHz, Chloroform-*d*) δ 7.20 – 7.13 (m, 3H), 7.11 – 7.05 (m, 2H), 7.03 – 7.01 (m, 2H), 6.80 (dd, *J* = 8.8, 1.1 Hz, 2H), 6.74 – 6.69 (m, 2H), 3.90 (s, 6H), 3.62 (s, 2H). ^{13}C NMR (126 MHz, Chloroform-*d*) δ 177.2, 154.1, 136.3, 134.9, 130.9, 130.8, 128.7, 127.8, 126.8, 121.8, 111.1, 60.2, 56.2, 44.3. HRMS (m/z, ESI): Calcd. for Chemical Formula: $C_{23}H_{19}Cl_2O_4^-$ [M-H]$^-$: 429.0666, Found: 429.0664.

3. The general procedure C for the synthesis of substrates[8]

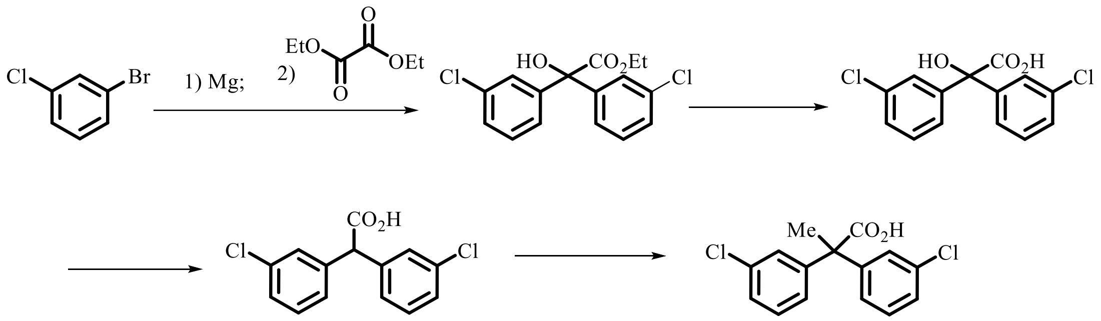

2,2-bis(3-chlorophenyl)-2-hydroxyacetic acid

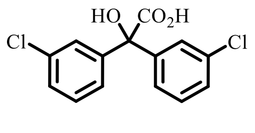

Under N_2 atmosphere, magnesium (332 mg, 13.86 mmol) and I_2 (15 mg, 0.12 mmol) were added in 5 mL THF, 1-bromo-3-chlorobenzene (2.52 g,

13.2 mmol) in 10 mL THF was then added dropwise at room temperature. The resulting reaction mixture was stirred for 8 h at this temperature. After that, diethyl oxalate (876 mg, 6.6 mmol) in 10 mL THF was added dropwise into the above reaction mixture at -78°C, and the resulting solution was slowly warmed to room temperature. After stirring overnight, the reaction was quenched with saturated NH_4Cl $_{(aq.)}$ at 0°C and stirred for another 30 minutes. The mixture was extracted with EA (35 mL X 3) three times. The combined organic phases were washed with brine, dried over Na_2SO_4, and concentrated in vacuo. The crude material was used directly in the next step without further purification.

The above crude mixture was dissolved in 20 mL THF, 10 mL H_2O and 20 mL MeOH, followed by the addition of NaOH (2.64 g, 66.0 mmol). The resulting reaction mixture was heated to reflux and stirred at this temperature for 24 h. After the reaction was completed, the mixture was neutralized with 1 M HCl $_{(aq.)}$ to pH 2 – 3 and extracted with EA (35 mL X 3) three times. The combined organic phases were washed with water and brine, dried over Na_2SO_4, and concentrated in vacuo. The crude materials were purified by flash column chromatography with hexane and acetone (15:1 – 2:1) to give the desired product, 1.49 g in 76% yield over two steps as an off-white solid.

2,2-bis(3-chlorophenyl)-propanoic acid

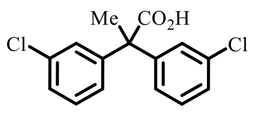

The 2,2-bis(3-chlorophenyl)-2-hydroxyacetic acid (1.0 g, 3.37 mmol) was dissolved in TFA (11 mL), and heated to reflux in an oil bath, then 57% HI (aq., 4.9 mL) was added dropwise. The solution was cooled to room temperature and stirred for 5 h. After that, most of the volatile was evaporated. The residue was diluted with water, added 1.0 M NaOH $_{(aq.)}$ to adjust pH < 3 in an ice bath, and extracted with EA (15 mL X 3) three times. The

combined organic phase was washed with brine, dried over anhydrous Na_2SO_4, filtered, and concentrated. The crude acid was purified by flash column chromatography (hexane and acetone (15:1 – 2:1) to give 2,2-bis(3-chlorophenyl)-acetic acid 798 mg in 85% yield as a white solid.

The 2,2-bis(3-chlorophenyl)-acetic acid (798 mg, 2.85 mmol) was dissolved in dried THF (10 mL) under N_2 atmosphere, and then LDA (3.14 mL, 2.0 M in Hexanes, 6.27 mmol) was added at -78°C. The resulting reaction mixture was stirred for 30 minutes at 45°C. After that, iodomethane (657 mg, 3.42 mmol) was added dropwise into the reaction mixture at -78°C, then the mixture was stirred for 15 h at 45°C. The reaction was cooled to room temperature and quenched with H_2O, then extracted with EA (40 mL X 3) three times. The combined organic phase was washed with brine, dried over Na_2SO_4, and concentrated in vacuo. The crude materials were purified by flash column chromatography with hexane and acetone (15:1 – 2:1) to give the desired product acid, 690 mg in 82% yield as a white solid. ^{1}H NMR (400 MHz, Chloroform-*d*) δ 7.29 – 7.22 (m, 6H), 7.15 – 7.12 (m, 2H), 1.91 (s, 3H). ^{13}C NMR (101 MHz, Chloroform-*d*) δ 179.9, 145.0, 134.3, 129.6, 128.2, 127.7, 126.4, 56.1, 26.7. HRMS (m/z, ESI): Calcd. for Chemical Formula: $C_{15}H_{11}Cl_2O_2^-$ $[M-H]^-$: 293.0142, Found: 293.0143.

2,2-bis(2-chlorophenyl)acetic acid

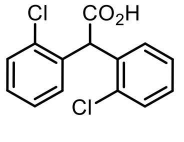

Prepared from 2-bromochlorobenzene, three steps, 350 mg in 50% yield as a white solid. ^{1}H NMR (400 MHz, Chloroform-*d*) δ 7.46 – 7.41 (m, 2H), 7.30 – 7.23 (m, 4H), 7.17 – 7.12 (m, 2H), 5.90 (s, 1H). ^{13}C NMR (101 MHz, Chloroform-*d*) δ 176.8, 134.8, 134.6, 129.9, 129.8, 129.1, 127.0, 51.3.

2,2-bis(3-chloro-4-methylphenyl)-propanoic acid

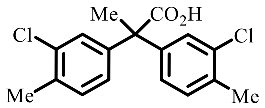

Prepared from 4-bromo-2-chloro-1-methylbenzene, four steps, 320 mg in 35% yield as a white solid. ^{1}H NMR (400 MHz, Chloroform-*d*) δ 7.24 (d, *J* = 2.1 Hz, 2H), 7.18 (d, *J* = 8.1 Hz, 2H), 7.04 (dd, *J* = 8.1, 2.1 Hz, 2H), 2.36 (s, 6H), 1.89 (s, 3H). ^{13}C NMR (101 MHz, Chloroform-*d*) δ 179.8, 142.5, 135.1, 134.3, 130.7, 128.5, 126.4, 55.4, 26.7, 19.7. HRMS (m/z, ESI): Calcd. for Chemical Formula: $C_{17}H_{15}Cl_2O_2^-$ $[M\text{-}H]^-$: 321.0455, Found: 321.0450.

2,2-bis(3-chloro-5-methylphenyl)-propanoic acid

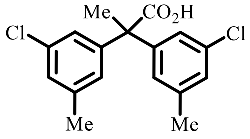

Prepared from 1-bromo-3-chloro-5-methylbenzene, four steps, 150 mg in 25% yield as a white solid. ^{1}H NMR (500 MHz, Chloroform-*d*) δ 7.10 (s, 2H), 7.04 (s, 2H), 6.94 (s, 2H), 2.31 (s, 6H), 1.89 (s, 3H). ^{13}C NMR (126 MHz, Chloroform-*d*) δ 179.8, 144.9, 139.7, 133.9, 128.2, 127.1, 125.3, 56.0, 26.8, 21.4. HRMS (m/z, ESI): Calcd. for Chemical Formula: $C_{17}H_{15}Cl_2O_2^-$ $[M\text{-}H]^-$: 321.0455, Found: 321.0452.

2,2-bis(2-chlorophenyl)propanoic acid

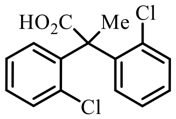

Prepared from 2,2-bis(2-chlorophenyl)acetic acid, 142 mg in 45% yield as a white solid. ^{1}H NMR (400 MHz, Chloroform-*d*) δ 7.47 – 7.42 (m, 2H), 7.40 – 7.36 (m, 2H), 7.30 – 7.23 (m, 4H), 2.30 (s, 3H). ^{13}C NMR (126 MHz, Chloroform-*d*) δ 179.2, 139.1, 134.0, 131.7, 130.1, 128.6, 128.4, 126.7, 57.2, 25.0. HRMS (m/z, ESI): Calcd. for Chemical Formula: $C_{15}H_{11}Cl_2O_2^-$ $[M\text{-}H]^-$: 293.0142, Found: 293.0138.

2,2-bis(4-chlorophenyl)propanoic acid

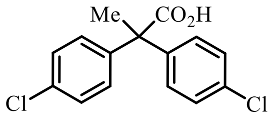

Prepared from 2,2-bis(4-chlorophenyl)acetic acid, 662 mg in 75% yield as a white solid. ^{1}H NMR (400 MHz, Chloroform-*d*) δ 7.33 – 7.27 (m, 4H), 7.21 – 7.16 (m, 4H), 1.90 (s, 3H). ^{13}C NMR (101 MHz, Chloroform-*d*) δ 180.5, 141.8, 133.3, 129.4, 128.5, 55.6, 26.8. HRMS (m/z, ESI): Calcd. for Chemical Formula: $C_{15}H_{11}Cl_2O_2^-$ $[M-H]^-$: 293.0142, Found: 293.0136.

4. The general procedure D for the synthesis of substrates[9]

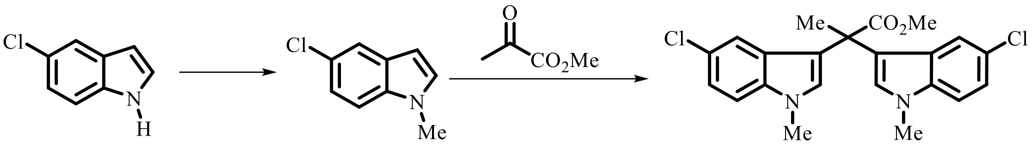

Methyl 2,2-bis(5-chloro-1-methyl-1H-indol-3-yl)-propanoate

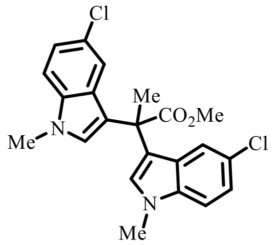

Under the N_2 atmosphere, 5-chloroindole (1.51 g, 10.0 mmol) was added in 20 mL DMF, then NaH (60 % dispersion in mineral oil, 420 mg, 10.5 mmol) was added in three portions at 0°C. The resulting reaction mixture was stirred for 1.5 h at room temperature. After that, MeI (1.56 g, 11.0 mmol) was added dropwise into the reaction mixture at 0°C, and then warmed to room temperature. After stirring overnight, the reaction was quenched with saturated NH_4Cl (aq.) at 0°C. The mixture was extracted with EA (35 mL X 3) three times. The combined organic phase was washed with brine, dried over Na_2SO_4, and concentrated in vacuo. The crude materials were purified by flash column chromatography with hexane and acetone (50:1 – 20:1) to give the desired product, 5-chloro-1-methyl-1H-indole, 1.47 g in 89% yield as a yellow oil.

Under the N_2 atmosphere, 5-chloro-1-methyl-1H-indole (1.0 g, 6.06 mmol) and methyl pyruvate (680 mg, 6.66 mmol) were added in 10 mL AcOH. The resulting reaction mixture was heated to reflux and stirred for 12 h at this temperature. After that, the reaction was cooled to room temperature and most of the volatile was evaporated. The mixture was extracted with EA (15 mL X 3) three times. The combined organic phase was washed with H_2O, and brine, dried over Na_2SO_4, and concentrated in vacuo. The crude materials were purified by flash column chromatography with hexane and acetone (30:1 – 10:1) to give the desired product, 540 mg in 89% yield as a yellow solid. ^{1}H NMR (500 MHz, Chloroform-*d*) δ 7.43 (dd, *J* = 1.9, 0.5 Hz, 2H), 7.21 (dd, *J* = 8.7, 0.6 Hz, 2H), 7.15 (dd, *J* = 8.7, 2.0 Hz, 2H), 6.86 (s, 2H), 3.72 (s, 6H), 3.71 (s, 3H), 2.06 (s, 3H). ^{13}C NMR (101 MHz, Chloroform-*d*) δ 175.4, 136.0, 128.8, 127.3, 124.8, 121.9, 120.5, 117.0, 110.4, 52.4, 45.9, 33.0, 26.1. HRMS (m/z, ESI): Calcd. for Chemical Formula: $C_{22}H_{20}Cl_2N_2NaO_2^+$ $[M+Na]^+$: 437.0797, Found: 437.0796.

Ethyl 2,2-bis(5-chloro-1-ethyl-1H-indol-3-yl)-4-oxopentanoate

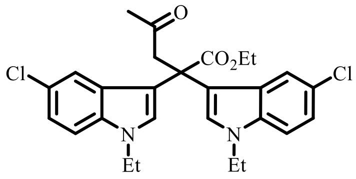

Prepared from 5-chloroindole and ethyl 2,4-dioxovalerate, two steps, 705 mg in 53% yield as a yellow solid. ^{1}H NMR (400 MHz, Chloroform-*d*) δ 7.35 (s, 2H), 7.20 – 7.15 (m, 4H), 7.02 (dd, *J* = 8.6, 2.1 Hz, 2H), 4.19 – 4.11 (m, 6H), 3.78 (s, 2H), 1.93 (s, 3H), 1.45 (t, *J* = 7.2 Hz, 6H), 1.18 (t, *J* = 7.1 Hz, 3H). ^{13}C NMR (101 MHz, Chloroform-*d*) δ 205.6, 172.8, 134.6, 128.7, 127.5, 124.4, 121.4, 120.4, 113.8, 110.4, 61.2, 50.4, 47.7, 41.3, 30.8, 15.5, 14.0. HRMS (m/z, ESI): Calcd. for Chemical Formula: $C_{27}H_{28}Cl_2N_2NaO_3^+$ $[M+Na]^+$: 521.1369, Found: 521.1366.

Methyl 2,2-bis(5-chloro-1-methyl-1H-indol-3-yl)-3-methylbutanoate[10]

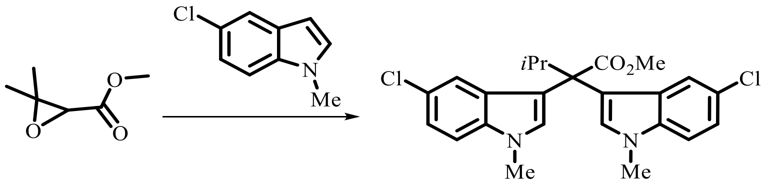

Under the N_2 atmosphere, 5-chloro-1-methyl-1H-indole (713 g, 4.32 mmol) and methyl 3,3-dimethyloxirane-2-carboxylate (281 mg, 2.16 mmol) were added in 10 mL DCM, then zinc iodide (689 mg, 2.16 mmol) was added to the reaction mixture. The resulting reaction mixture was stirred for 16 h at room temperature. After that, the reaction was quenched with H_2O. The mixture was extracted with DCM (15 mL X 3) three times. The combined organic phase was washed with H_2O, and brine, dried over Na_2SO_4, and concentrated in vacuo. The crude materials were purified by flash column chromatography with hexane and acetone (30:1 – 10:1) to give the desired product, 200 mg in 58% yield as a white solid. ^{1}H NMR (500 Mz, Chloroform-*d*) δ 7.18 (s, 1H), 7.16 (s, 1H), 7.11 (s, 2H), 7.07 – 7.04 (m, 4H), 3.78 (s, 6H), 3.61 (s, 3H), 3.23 (p, J = 6.7 Hz, 1H), 0.94 (d, J = 6.7 Hz, 6H). ^{13}C NMR (126 MHz, Chloroform-*d*) δ 174.5, 135.4, 130.7, 128.6, 124.5, 121.4, 121.3, 113.3, 110.1, 55.4, 52.0, 33.5, 33.2, 19.4. HRMS (m/z, ESI): Calcd. for Chemical Formula: $C_{24}H_{24}Cl_2N_2NaO_2^+$ $[M+Na]^+$: 465.1107, Found: 465.1110.

5. The general procedure E for the synthesis of substrates[8]

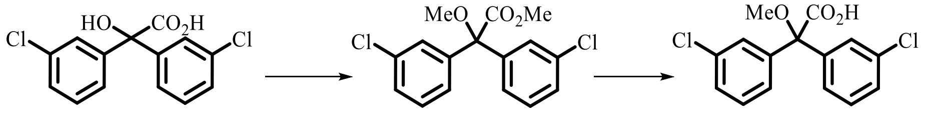

2,2-bis(3-chlorophenyl)-2-methoxyacetic acid

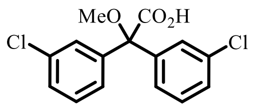

Under the N_2 atmosphere, 2,2-bis(3-chlorophenyl)-2-hydroxyacetic acid (490 mg, 1.66 mmol) was added in 10 mL DMF, then NaH (60 % dispersion in mineral oil, 199 mg, 4.98 mmol) was added in three portions at 0°C. The resulting reaction mixture was stirred for 1.5 h at room temperature. After that, MeI (1.12g, 11.0 mmol) was added dropwise into the reaction mixture at 0°C, and then warmed to room temperature. After stirring overnight, the reaction was quenched with saturated NH_4Cl (aq.) at 0°C. The mixture was extracted with EA (30 mL X 3) three times. The combined organic phase was washed with brine, dried over Na_2SO_4, and concentrated in vacuo. The crude materials were purified by flash column chromatography with hexane and acetone (50:1 – 20:1) to give the desired product, methyl 2,2-bis(3-chlorophenyl)-2-methoxyacetate, 480 mg in 89% yield as a white solid.

Under the N_2 atmosphere, methyl 2,2-bis(3-chlorophenyl)-2-methoxyacetate (480 mg, 1.48 mmol) was dissolved in 3 mL THF, 3 mL MeOH, and 3 mL 5 M NaOH $_{(aq.)}$. The resulting reaction mixture was heated to reflux and stirred at this temperature for 24 h. After that, the reaction was cooled to room temperature, the reaction mixture was neutralized with 1.0 M HCl $_{(aq.)}$ to pH 2 – 3 and extracted with EA (15 mL X 3) three times. The combined organic phase was washed with water and brine, dried over Na_2SO_4, and concentrated in vacuo. The crude materials were purified by flash column chromatography with hexane and acetone (5:1 – 2:1) to give the desired product, 593 mg in 86% yield as a white solid. ^{1}H NMR (400 MHz, Chloroform-*d*) δ 9.66 (s, 1H), 7.50 – 7.49 (m, 2H), 7.37 – 7.27 (m, 6H), 3.20 (s, 3H). ^{13}C NMR (101 MHz, Chloroform-*d*) δ 175.2, 140.7, 134.4, 129.6, 128.9, 128.5, 126.8, 86.0, 53.7. HRMS (m/z, ESI): Calcd. for Chemical Formula: $C_{15}H_{11}Cl_2O_3^-$ [M-H]$^-$: 309.0091, Found: 309.0087.

2,2-bis(3-chloro-4-methylphenyl)-2-methoxy-acetic acid

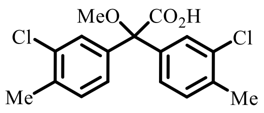

Prepared from 2,2-bis(3-chloro-4-methylphenyl)-2-hydroxy-acetic acid, two steps, 205 mg in 60% yield as a white solid. ^{1}H NMR (500 MHz, Chloroform-*d*) δ 7.45 (d, *J* = 1.7 Hz, 2H), 7.24 – 7.19 (m, 4H), 3.17 (s, 3H), 2.38 (s, 6H). ^{13}C NMR (126 MHz, Chloroform-*d*) δ 173.0, 137.1, 136.9, 134.4, 130.8, 128.9, 126.9, 53.5, 19.8. HRMS (m/z, ESI): Calcd. for Chemical Formula: $C_{17}H_{15}Cl_2O_3^-$ [M-H]$^-$: 337.0404, Found: 337.0403.

2,2-bis(3-chloro-4-methoxyphenyl)-2-methoxy-acetic acid

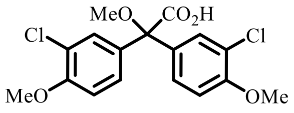

Prepared from 2,2-bis(3-chloro-4-methoxyphenyl)-2-hydroxy-acetic acid, two steps, 245 mg in 63% yield as a white solid. ^{1}H NMR (500 MHz, Chloroform-*d*) δ 7.46 (d, *J* = 2.3 Hz, 2H), 7.30 (dd, *J* = 8.6, 2.3 Hz, 2H), 6.91 (d, *J* = 8.6 Hz, 2H), 3.92 (s, 6H), 3.15 (s, 3H). ^{13}C NMR (126 MHz, Chloroform-*d*) δ 173.7, 155.2, 131.1, 130.3, 128.3, 122.4, 111.5, 85.7, 56.2, 53.4. HRMS (m/z, ESI): Calcd. for Chemical Formula: $C_{17}H_{15}Cl_2O_5^-$ [M-H]$^-$: 369.0302, Found: 369.0299.

6. The procedure for the synthesis of 2,2-bis(3-chloro-4-methylphenyl)-2-fluoroacetic acid.[8]

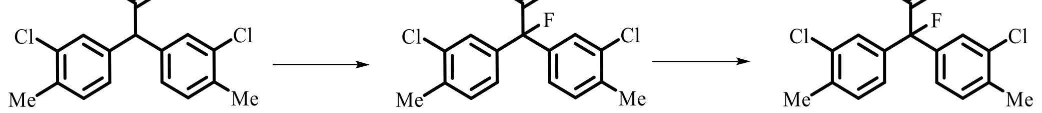

Under the N_2 atmosphere, ethyl 2,2-bis(3-chloro-4-methylphenyl)-acetate (720 mg, 2.14 mmol) was added in 8 mL THF, then LDA (2.57 mL, 1.0 M in Hexanes, 2.57 mmol) was added dropwise at -78°C. The reaction mixture was stirred for 30 minutes at 45°C. After that, NFSI (810 mg, 2.57 mmol)

in THF (5 mL) was added dropwise into the reaction mixture at -78°C, and then warmed to room temperature. After stirring for 24 h, the reaction was quenched with saturated $NH_4Cl_{(aq.)}$ at 0°C. The mixture was extracted with EA (10 mL X 3) three times. The combined organic phase was washed with brine, dried over Na_2SO_4, and concentrated in vacuo. The crude materials were purified by flash column chromatography with hexane and acetone (50:1 – 20:1) to give the desired product, ethyl 2,2-bis(3-chloro-4-methylphenyl)-2-fluoroacetate, 222 mg in 29% yield as a white solid.

Under the N_2 atmosphere, ethyl 2,2-bis(3-chloro-4-methylphenyl)-2-fluoroacetate (222 mg, 0.63 mmol) was dissolved in 2 mL THF, and 2 mL H_2O, followed by the addition of NaOH (126 mg, 3.15 mmol). The resulting reaction mixture was stirred at room temperature for 24 h. After that, the reaction mixture was neutralized with 1 M $HCl_{(aq.)}$ to pH 2 – 3 and extracted with EA (8 mL X 3) three times. The combined organic phases were washed with water and brine, dried over Na_2SO_4, and concentrated in vacuo. The crude materials were purified by flash column chromatography with hexane and acetone (15:1 – 5:1) to give the desired product, 167 mg in 81% yield as a white solid.

^{1}H NMR (500 MHz, Chloroform-*d*) δ 7.45 (d, *J* = 1.5 Hz, 2H), 7.26 (d, *J* = 1.5 Hz, 4H), 2.40 (s, 6H). ^{19}F NMR (471 MHz, Chloroform-*d*) δ -138.95 (s, 1F). ^{13}C NMR (126 MHz, Chloroform-*d*) δ 173.7 (d, *J* = 28.6 Hz), 137.7, 136.3 (d, *J* = 23.1 Hz), 134.7, 131.0, 127.5 (d, *J* = 7.3 Hz), 125.1 (d, *J* = 6.5 Hz), 95.9 (d, *J* = 191.4 Hz), 19.9. HRMS (m/z, ESI): Calcd. for Chemical Formula: $C_{16}H_{12}Cl_2FO_2^-$ [M-H]$^-$: 325.0204, Found: 325.0201.

7. The procedure for the synthesis of 2-acetamido-2,2-bis-(3-chloro-4-methoxy-phenyl)-acetic acid.[11]

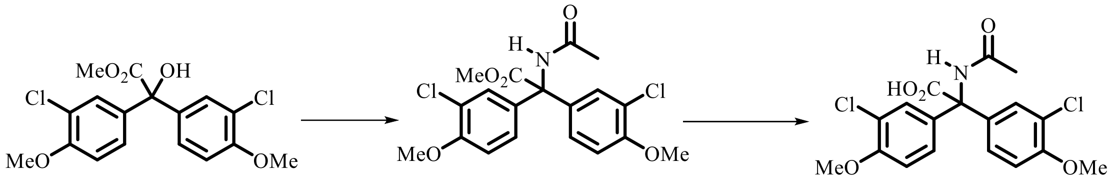

Under the N_2 atmosphere, methyl 2,2-bis(3-chloro-4-methoxyphenyl)-2-hydroxyacetate (370 mg, 1.0 mmol) was added in 2.0 mL MeCN, then Conc.H_2SO_4 (1.0 mL) was added dropwise at 0°C. The reaction mixture was stirred for 30 minutes at this temperature. After that, the reaction was poured into saturated NH_4Cl $_{(aq.)}$ at 0°C. The mixture was extracted with EA (10 mL X 3) three times. The combined organic phases were washed with brine, dried over Na_2SO_4, and concentrated in vacuo. The crude materials were purified by flash column chromatography with hexane and acetone (30:1 – 10:1) to give the desired product, methyl 2-acetamido-2,2-bis(3-chloro-4-methoxyphenyl)-acetate, 380 mg in 92% yield as a white solid.

Under the N_2 atmosphere, methyl 2-acetamido-2,2-bis(3-chloro-4-methoxyphenyl)-acetate (380 mg, 0.92 mmol) was dissolved in 6 mL THF, 6 mLMeOH, and 2.0 M LiOH $_{(aq.)}$ (2 mL). The resulting reaction mixture was stirred at 55°C for 16 h. After that, the reaction mixture was neutralized with 1.0 M HCl $_{(aq.)}$ to pH 4 – 6 and extracted with EA (10 mL X 3) three times. The combined organic phase was washed with water and brine, dried over Na_2SO_4, and concentrated in vacuo. The crude materials were purified by flash column chromatography with hexane and acetone (5:1 – 2:1) to give the desired product, 310 mg in 85 % yield as a white solid. ^{1}H NMR (400 MHz, Methanol-d_4) δ 7.32 (d, J = 2.4 Hz, 2H), 7.19 (dd, J = 8.8, 2.4 Hz, 2H), 6.91 (d, J = 8.8 Hz, 2H), 3.79 (s, 6H), 1.94 (s, 3H). ^{13}C NMR (101 MHz, Methanol-d_4) δ 173.6, 172.8, 156.0, 134.8, 131.5, 129.5, 122.8, 112.6, 69.1, 56.8, 23.0. HRMS (m/z, ESI): Calcd. for Chemical Formula: $C_{18}H_{16}Cl_2NO_5^-$ [M-H]$^-$: 396.0411,

Found: 396.0413.

8. The procedure for the synthesis of 3,3-bis(3-chloro-4-methoxyphenyl)-butanoic acid.[1]

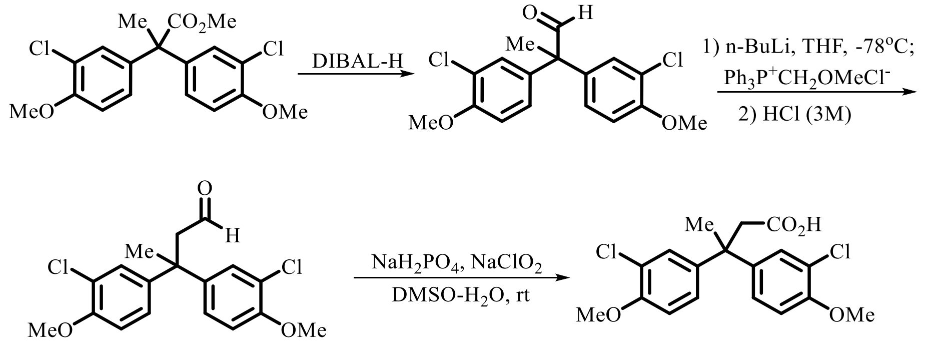

Under the N_2 atmosphere, methyl 2,2-bis(3-chloro-4-methoxyphenyl)-propanoate (715 mg, 1.94 mmol) was added in 10 mL DCM, then DIBAL-H (2.0 mL, 1.0 M in cyclohexane, 2.0 mmol) was added dropwise at -78°C. The reaction mixture was stirred for 60 minutes at this temperature. After that, the reaction was quenched with saturated $NH_4Cl_{(aq.)}$ at -78°C and stirred for 8 h at room temperature. The mixture was extracted with DCM (10 mL X 3) three times. The combined organic phase was washed with brine, dried over Na_2SO_4, and concentrated in vacuo. The crude materials were purified by flash column chromatography with hexane and acetone (40:1 – 10:1) to give the desired product, 2,2-bis(3-chloro-4-methoxyphenyl)-propanal, 534 mg in 71% yield as a white solid. Under the N_2 atmosphere, (Methoxymethyl)triphenyl-phosphonium chloride (1.18 g, 3.45 mmol) was dissolved in 15 mL THF, then n-BuLi (1.58 mL, 2.0 M in Hexanes, 3.17 mmol) was added dropwise at -78°C. The reaction mixture was stirred for 60 minutes at room temperature. After that, 2,2-bis(3-chloro-4-methoxyphenyl)-propanal (534 mg, 1.38 mmol) in 5 mL THF was added dropwise into the reaction at -78°C. After stirring overnight at room temperature, the reaction was quenched with H_2O at 0°C. Then, 3.0 M $HCl_{(aq.)}$ (10 mL) was added to the mixture, the solution was stirred for 24 h. The mixture was extracted with EA (10 mL X 3) three times. The combined

organic phase was washed with brine, dried over Na_2SO_4, and concentrated in vacuo. The crude materials were purified by flash column chromatography with hexane and acetone (40:1 – 10:1) to give the desired product, 3,3-bis(3-chloro-4-methoxyphenyl)-butanal, 277 mg in 57% yield as a white solid.

Under the N_2 atmosphere, 3,3-bis(3-chloro-4-methoxyphenyl)-butanal (277 mg, 0.79 mmol) and NaH_2PO_4 (379 mg, 3.16 mmol) were added in co-solvents (4 mL DMSO and 2 mL H_2O). Then, $NaClO_2$ (107 mg, 1.19 mmol) in 2 mL H_2O was added dropwise to the reaction mixture and stirred overnight at room temperature. After that, the mixture was extracted with EA (10 mL X 3) three times. The combined organic phase was washed with brine, dried over Na_2SO_4, and concentrated in vacuo. The crude materials were purified by flash column chromatography with hexane and acetone (20:1 – 3:1) to give the desired acid, 256 mg in 88% yield as a white solid. ^1H NMR (500 MHz, Chloroform-*d*) δ 7.17 (d, J = 2.4 Hz, 2H), 7.02 (dd, J = 8.7, 2.4 Hz, 2H), 6.83 (d, J = 8.7 Hz, 2H), 3.88 (s, 6H), 3.08 (s, 2H), 1.81 (s, 3H). ^{13}C NMR (126 MHz, Chloroform-*d*) δ 153.4, 140.8, 128.8, 126.2, 111.6, 56.1, 45.6, 43.9, 28.1. HRMS (m/z, ESI): Calcd. for Chemical Formula: $C_{18}H_{17}Cl_2O_4^-$ $[M-H]^-$: 367.0509, Found: 367.0508.

9. The procedure for the synthesis of 4,4-bis(3-chloro-4-methoxyphenyl)-pentanoic acid.[12]

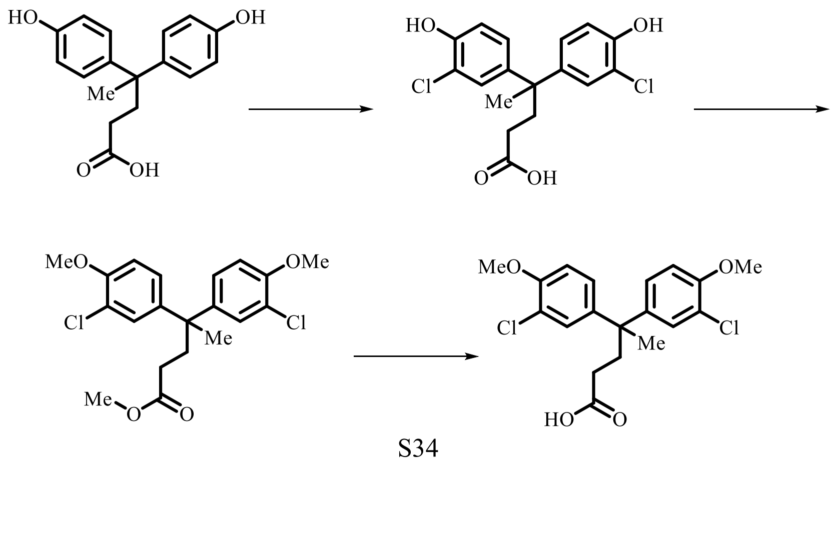

Under the N_2 atmosphere, diphenolic acid (1.43 g, 5.0 mmol) and NCS (1.47 g, 11 mmol) were added in co-solvents (30 mL 1,4-dioxane and 7.5 mL $CHCl_3$). The reaction mixture was heated to reflux and stirred overnight at this temperature. Then, the reaction was cooled to room temperature and quenched with H_2O. The mixture was extracted with EA (30 mL X 3) three times. The combined organic phase was washed with brine, dried over Na_2SO_4, and concentrated in vacuo. The crude material was used directly in the next step without further purification.

Under the N_2 atmosphere, the above crude product was dissolved in 25 mL DMF. Then, NaH (60 % dispersion in mineral oil, 620 mg, 15.5 mmol) was added in three portions at 0°C. After the resulting reaction mixture stirring for 1.5 h at room temperature, MeI (3.55 g, 25 mmol) was added dropwise to the reaction mixture at room temperature. Then, the mixture was stirred for 18 h at 60°C and cooled to room temperature. The mixture was quenched with H_2O and extracted with EA (30 mL X 3) three times. The combined organic phase was washed with brine, dried over Na_2SO_4, and concentrated in vacuo. The crude material was used directly in the next step without further purification.

Under the N_2 atmosphere, the above product was dissolved in 10 mL THF, 10 mL MeOH, and 2 M NaOH $_{(aq.)}$ (10 mL). The resulting reaction mixture was heated to reflux and stirred at this temperature for 24 h. After that, the reaction mixture was neutralized with 1 M HCl $_{(aq.)}$ to pH 2 – 3 and extracted with EA (10 mL X 3) three times. The combined organic phase was washed with water and brine, dried over Na_2SO_4, and concentrated in vacuo. The crude materials were purified by flash column chromatography with hexane and acetone (5:1 – 2:1) to give the desired product, 1.19 g in 62% yield as a white solid over three steps. ^{1}H NMR (500 MHz, Chloroform-*d*) δ 7.18 (d, J = 2.4 Hz, 2H), 7.01 (dd, J = 8.7, 2.4 Hz, 2H), 6.84 (d, J = 8.7 Hz, 2H), 3.88 (s, 6H), 2.41 – 2.34 (m, 2H),

2.18 – 2.11 (m, 2H), 1.56 (s, 3H). ^{13}C NMR (126 MHz, Chloroform-*d*) δ 179.7, 153.4, 141.5, 129.1, 126.5, 122.3, 111.9, 56.3, 44.6, 36.2, 30.0, 27.5. HRMS (m/z, ESI): Calcd. for Chemical Formula: $C_{19}H_{19}Cl_2O_4^-$ [M-H]$^-$: 381.0666, Found: 381.0668.

10. The procedure for the synthesis of 2-(3-chloro-4-methoxyphenyl)-2-(4-methoxyphenyl)-propanoic acid.[13,14]

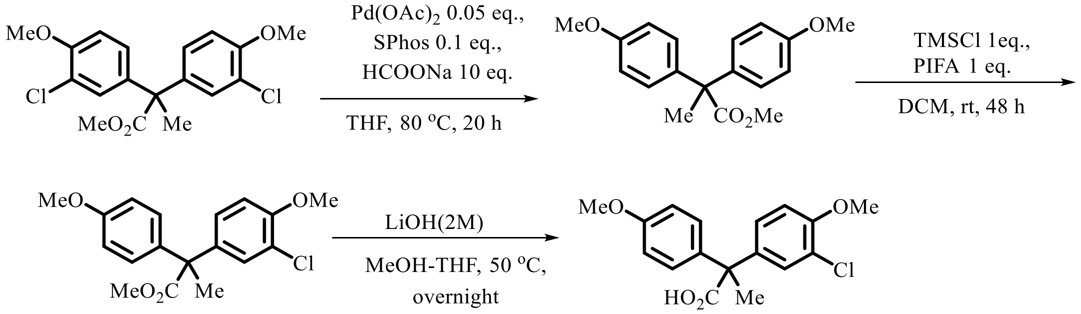

Under the N_2 atmosphere, methyl 2,2-bis(3-chloro-4-methoxyphenyl)-propanoate (360 mg, 0.94 mmol), $Pd(OAc)_2$ (10 mg, 0.047 mmol), SPhos (38 mg, 0.094 mmol) and HCO_2Na (640 mg, 9.4 mmol) were added in THF (6 mL). The reaction mixture was heated to reflux and stirred for 20 h at this temperature. Then, the reaction was cooled to room temperature and quenched with H_2O. The mixture was extracted with EA (30 mL X 3) three times. The combined organic phase was washed with brine, dried over Na_2SO_4, and concentrated in vacuo. The crude materials were purified by flash column chromatography with hexane and acetone (50:1 – 20:1) to give the desired product, 254 mg in 86% yield as a white solid.

Under the N_2 atmosphere, PIFA (336 mg, 0.78 mmol) was dissolved in 3 mL DCM, then TMSCl (86 mg, 0.78 mmol) was added dropwise in the solution, and the mixture was allowed to stir for 10 minutes at room temperature. After that, methyl 2,2-bis(4-methoxyphenyl)-propanoate (254 mg, 0.78 mmol) in 3 mL DCM was added dropwise to the above reaction mixture. Then, the mixture

was stirred for 48 h at this temperature. Finally, the reaction was quenched with H_2O. The mixture was extracted with DCM (10 mL X 3) three times. The combined organic phase was washed with brine, dried over Na_2SO_4, and concentrated in vacuo. The crude material was used directly in the next step without further purification.

Under the N_2 atmosphere, the above product was dissolved in 2 mL THF, 2 mL MeOH, and 2.0 M LiOH $_{(aq.)}$ (2 mL). The resulting reaction mixture was stirred overnight at 50°C. After that, the reaction mixture was neutralized with 1.0 M HCl $_{(aq.)}$ to pH 2 – 3 and extracted with EA (10 mL X 3) three times. The combined organic phase was washed with water and brine, dried over Na_2SO_4, and concentrated in vacuo. The crude materials were purified by flash column chromatography with hexane and acetone (5:1 – 2:1) to give the desired product, 224 mg in 90% yield as a white solid over two steps. ^{1}H NMR (500 MHz, Chloroform-*d*) δ 7.28 (d, *J* = 2.4 Hz, 1H), 7.21 – 7.16 (m, 2H), 7.12 (dd, *J* = 8.7, 2.4 Hz, 1H), 6.89 – 6.83 (m, 3H), 3.89 (s, 3H), 3.81 (s, 3H), 1.88 (s, 3H). ^{13}C NMR (126 MHz, Chloroform-*d*) δ 180.1, 158.6, 153.9, 137.2, 135.3, 129.9, 129.0, 127.5, 122.0, 113.6, 111.5, 56.2, 55.3, 54.9, 27.0. HRMS (m/z, ESI): Calcd. for Chemical Formula: $C_{17}H_{16}ClO_4^-$ [M-H]$^-$: 319.0743, Found: 319.0743.

11. The procedure for the synthesis of 2-(3-chloro-4-methoxyphenyl)-2,3-dimethyl- butanoic acid.[14]

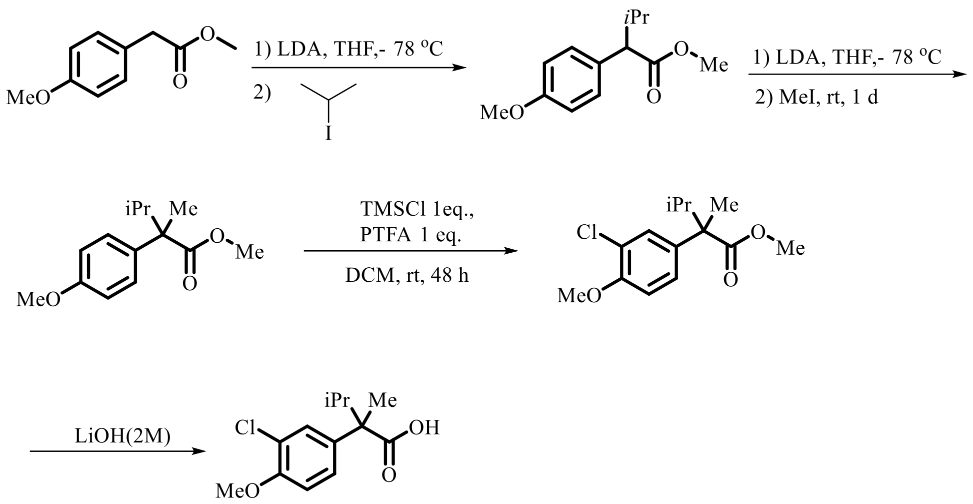

Under the N_2 atmosphere, methyl 2-(4-methoxyphenyl)-acetate (1.08 g, 6.0 mmol) was added in 10 mL THF, LDA (7.2 mL, 1.0 M in Hexanes, 7.2 mmol) was then added dropwise at -78°C. The reaction mixture was stirred for 60 minutes at this temperature. 2-iodopropane (1.22 g, 7.2 mmol) was added dropwise into the reaction mixture at -78°C, then it was allowed to slowly warm to room temperature. After stirring for 24 h, the reaction was quenched with saturated $NH_4Cl_{(aq.)}$ at 0°C. The mixture was extracted with EA (40 mL X 3) three times. The combined organic phase was washed with brine, dried over Na_2SO_4, and concentrated in vacuo. The crude material was used directly in the next step without further purification.

Under the N_2 atmosphere, the above crude product was dissolved in 10 mL THF, LDA (7.2 mL, 1.0 M in Hexanes, 7.2 mmol) was then added dropwise at -78°C. The reaction mixture was stirred for 45 minutes at room temperature. MeI (1.02 g, 7.2 mmol) was added dropwise into the reaction mixture at -78°C, then the reaction mixture was warmed to room temperature. After stirring overnight, the reaction was quenched with saturated $NH_4Cl_{(aq.)}$ at 0°C. The mixture was extracted with EA (30 mL X 3) three times. The combined organic phase was washed with brine, dried over

Na_2SO_4, and concentrated in vacuo. The crude materials were purified by flash column chromatography with hexane and acetone (50:1 – 20:1) to give the desired product, 730 mg in 50% yield as a white solid over two steps.

Under the N_2 atmosphere, PIFA (778 mg, 1.81 mmol) was dissolved in 12 mL DCM, then TMSCl (196 mg, 1.81 mmol) was added dropwise in the solution and the mixture was needed to stir for 10 minutes. After that, methyl 2-(4-methoxyphenyl)-2,3-dimethylbutanoate (428 mg, 1.81 mmol) in 4 mL DCM was added dropwise to the reaction mixture. Then, the mixture was stirred for 48 h at room temperature. Finally, the reaction was quenched with H_2O. The mixture was extracted with DCM (10 mL X 3) three times. The combined organic phase was washed with brine, dried over Na_2SO_4, and concentrated in vacuo. The crude material was used directly in the next step without further purification.

Under the N_2 atmosphere, the above product was dissolved in 4 mL THF, 2 mL MeOH, and 2.0 M NaOH (2 mL). The resulting reaction mixture was heated to reflux and stirred overnight. After cooling to room temperature, the reaction mixture was neutralized with 1.0 M HCl $_{(aq.)}$ to pH 2 – 3 and extracted with EA (10 mL X 3) three times. The combined organic phase was washed with water and brine, dried over Na_2SO_4, and concentrated in vacuo. The crude materials were purified by flash column chromatography with hexane and acetone (5:1 – 2:1) to give the desired product, 200 mg in 43% yield as a white solid over four steps. ^{1}H NMR (400 MHz, Chloroform-*d*) δ 7.47 (d, J = 2.4 Hz, 1H), 7.32 (dd, J = 8.7, 2.4 Hz, 1H), 6.88 (d, J = 8.7 Hz, 1H), 3.88 (s, 3H), 2.67 – 2.60 (m, 1H), 1.44 (s, 3H), 0.99 (d, J = 6.6 Hz, 3H), 0.62 (d, J = 6.9 Hz, 3H).^{13}C NMR (101 MHz, Chloroform-*d*) δ 181.6, 153.9, 135.2, 128.8, 126.2, 122.2, 111.6, 56.1, 52.8, 34.7, 18.9, 16.9, 14.5. HRMS (m/z, ESI): Calcd. for Chemical Formula: $C_{13}H_{16}ClO_3^-$ [M-H]$^-$: 255.0793, Found: 255.0795.

12. The procedure for the synthesis of 4-(2,7-dichloro-9H-xanthen-9-yl)butanoic acid.[1]

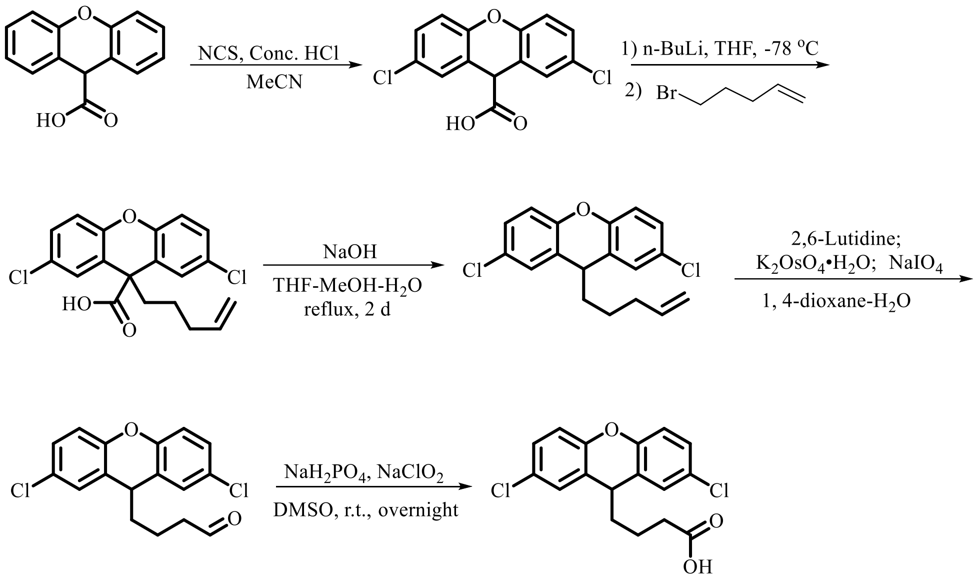

Under the N_2 atmosphere, 9H-xanthene-9-carboxylic acid (2.26 g, 10.0 mmol) was dissolved in 60 mL MeCN, and 10 mL DCM. Then, Conc. HCl (6.25 mL, 75 mmol) was added dropwise to the solution at 0°C. After that, NCS (2.95 g, 22.0 mmol) was added to the reaction mixture at this temperature, and then the mixture was warmed to room temperature. After stirring for 24 h, the reaction was quenched with saturated $NH_4Cl_{(aq.)}$ at 0°C. The mixture was extracted with EA (40 mL X 3) three times. The combined organic phase was washed with brine, dried over Na_2SO_4, and concentrated in vacuo. The crude material was used directly in the next step without further purification.

Under the N_2 atmosphere, the above crude product was dissolved in 40 mL THF, and n-BuLi (11 mL, 2.0 M in Hexanes, 22.0 mmol) was added dropwise at -78°C. The reaction mixture was stirred for 45 minutes at room temperature. After that, 5-bromopent-1-ene (1.94 g, 13.0 mmol) was added dropwise into the reaction mixture at -78°C, and then the mixture was warmed to room temperature. After stirring overnight, the reaction was quenched with saturated $NH_4Cl_{(aq.)}$ at 0°C. The mixture

was extracted with EA (30 mL X 3) three times. The combined organic phases were washed with brine, dried over Na_2SO_4, and concentrated in vacuo. The crude materials were purified by flash column chromatography with hexane and acetone (20:1 – 4:1) to give the desired product, 2.72 g in 75 % yield as a white solid.

Under the N_2 atmosphere, 2,7-dichloro-9-(pent-4-en-1-yl)-9H-xanthene-9-carboxylic acid (1.75 g, 4.82 mmol) was dissolved in 20 mL THF, 20 mL MeOH, and 2 M NaOH $_{(aq.)}$ (10 mL). After that, the reaction mixture was heated to reflux and stirred for 24 h at this temperature. Finally, the reaction was cooled to room temperature and neutralized with 1.0 M HCl $_{(aq.)}$ to pH 5 – 6 and extracted with EA (30 mL X 3) three times. The combined organic phase was washed with water and brine, dried over Na_2SO_4, and concentrated in vacuo. The crude materials were purified by flash column chromatography with hexane and acetone (100:1 – 50:1) to give the desired product, 1.23 g in 80% yield as a white solid.

Under the N_2 atmosphere, 2,7-dichloro-9-(pent-4-en-1-yl)-9H-xanthene (600 mg, 1.88 mmol), $K_2OsO_4{\cdot}2H_2O$ (15 mg, 0.038 mmol), $NaIO_4$ (3.23 g, 7.52 mmol), and 2,6-lutidine (414 mg, 3.76 mmol) were added in 12 mL 1,4-dioxane and H_2O (3 mL). After that, the reaction mixture was stirred overnight at room temperature. Finally, the reaction was diluted with H_2O. The mixture was extracted with EA (15 mL X 3) three times. The combined organic phase was washed with brine, dried over Na_2SO_4, and concentrated in vacuo. The crude materials were purified by flash column chromatography with hexane and acetone (50:1 – 10:1) to give the desired product, 470 mg in 78% yield as a white solid.

Under the N_2 atmosphere, 4-(2,7-dichloro-9H-xanthen-9-yl)-butanal (321 mg, 1.0 mmol), and NaH_2PO_4 (480 mg, 4.0 mmol) were dissolved in 10 mL DMSO and H_2O (3 mL). $NaClO_2$ (158 mg,

1.4 mmol) was dissolved in H_2O (2 mL) then the mixture was added dropwise into the above reaction solution. After that, the reaction mixture was stirred overnight at room temperature. The reaction was diluted with H_2O and extracted with EA (15 mL X 3) three times. The combined organic phase was washed with brine, dried over Na_2SO_4, and concentrated in vacuo. The crude materials were purified by flash column chromatography with hexane and acetone (10:1 – 5:1) to give the desired product, 212 mg in 63% yield as a white solid. ^{1}H NMR (500 MHz, Chloroform-*d*) δ 7.20 – 7.14 (m, 4H), 7.01 (d, *J* = 8.5 Hz, 2H), 3.96 (t, *J* = 5.8 Hz, 1H), 2.23 (t, *J* = 7.4 Hz, 2H), 1.80 – 1.73 (m, 2H), 1.50 – 1.42 (m, 2H). ^{13}C NMR (126 MHz, Chloroform-*d*) δ 178.8, 150.5, 128.3, 128.1, 128.0, 125.7, 117.9, 39.4, 38.6, 33.6, 20.3. HRMS (m/z, ESI): Calcd. for Chemical Formula: $C_{17}H_{13}Cl_2O_3^-$ [M-H]$^-$: 335.0247, Found: 335.0243.

The general procedure of Suzuki cross-coupling desymmetrization reaction of xanthene substrates

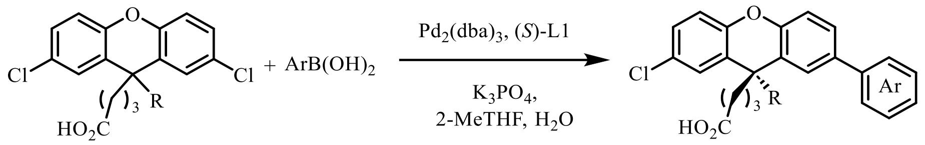

Under N_2 atmosphere, a mixture of Pd_2dba_3 (2.3 mg, 0.0025 mmol) and (*S*)-**L1**(3.2 mg, 0.0055 mmol) was added 0.5 mL 2-MeTHF, and the mixture was stirred at room temperature for 20 min. The resulting metal-ligand complex solution was added to a reaction flask containing 4.5 mL 2-MeTHF solution of Xanthene acid (0.25 mmol) and arylboronic acid (0.3 mmol), and K_3PO_4 (530.7 mg, 2.5 mmol), followed by addition of 0.41 mL H_2O. Then the resulting reaction mixture was stirred at 60°C for 18 h. The reaction was then quenched with water, neutralized to pH 3-5 with 1.0 M HCl $_{(aq.)}$, and extracted with ethyl acetate (4 mL X 3) three times. The combined organic phase was washed with brine, dried over Na_2SO_4, and concentrated in vacuo. The crude materials were purified by flash column chromatography with hexane and ethyl acetate as the eluents to give the desired products.

Characterization data of desymmetrization products of xanthene substrates

(*R*)-4-(9-butyl-2-chloro-7-(9-phenyl-9H-carbazol-3-yl)-9H-xanthen-9-yl)-butanoic acid (1)

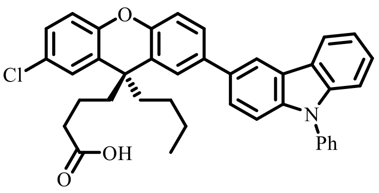

White solid, 61 mg, 41% yield, $[\alpha]_D^{20}$ = -1.32 (*c* 0.400 $CHCl_3$) for 94.5:5.5 er. ^{1}H NMR (500 MHz, Chloroform-*d*) δ 8.30 (d, *J* = 1.9 Hz, 1H), 8.21 (d, *J* = 7.8 Hz, 1H), 7.64 – 7.51 (m, 7H), 7.50 – 7.40 (m, 4H), 7.32 – 7.31 (m, 1H), 7.28 – 7.25 (m, 1H), 7.17 (dd, *J* = 8.7, 2.4 Hz, 1H), 7.09 (d, *J* = 8.2 Hz, 1H), 6.99 (d, *J* = 8.5 Hz, 1H), 2.20 – 2.09 (m, 3H), 2.06 – 1.96 (m, 1H), 1.95 – 1.83 (m, 2H), 1.35 – 1.28 (m, 2H), 1.18 – 1.12 (m, 2H), 0.96 – 0.87 (m, 2H), 0.74 (t, *J* = 7.3 Hz, 3H). ^{13}C NMR (101 MHz, Chloroform-*d*) δ 178.9, 150.6, 141.5, 140.4, 137.8, 137.6, 133.3, 130.0, 128.1, 127.8, 127.6, 127.2, 126.9, 126.3, 126.2, 125.5, 125.0, 124.5, 124.0, 123.6, 120.6, 120.2, 118.6, 117.9, 116.7, 110.2, 110.1, 45.6, 44.1, 42.8, 33.8, 27.0, 23.0, 20.2, 14.0. HRMS (m/z, ESI): Calcd. for Chemical Formula: $C_{39}H_{33}ClNO_3^-$ [M-H]$^-$: 598.2154, Found: 598.2137. A sample of the acid was esterification to provide the Me-ester for the HPLC analysis of the reaction product: Daicel Chiralpak IA, hexane/*iso*-propanol = 99: 1, 1.0 mL/min, λ= 295 nm, retention time: 9.09 min (major) and 11.91 min (minor).

(*R*)-4-(2-chloro-7-(4-methoxyphenyl)-9H-xanthen-9-yl)-butanoic acid (2)

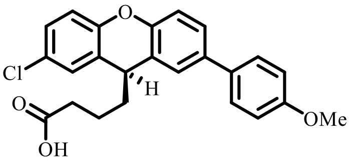

White solid, 76 mg, 74% yield, $[\alpha]_D^{20}$ = +9.50 (*c* 1.00 $CHCl_3$) for 89.5:10.5 er. ^{1}H NMR (500 MHz, Chloroform-*d*) δ 7.51 – 7.47 (m, 2H), 7.40 (dd, *J* = 8.4, 2.3 Hz, 1H), 7.34 (d, *J* = 2.2 Hz, 1H), 7.20 – 7.16 (m, 2H), 7.11 (d, *J* = 8.4 Hz, 1H), 7.05 – 7.02 (m, 1H), 6.99 – 6.95 (m, 2H), 4.04 (t, *J* = 5.8 Hz, 1H), 3.85 (s, 3H), 2.23 (t, *J* = 7.4 Hz, 2H), 1.85 – 1.77 (m, 2H), 1.55 – 1.48 (m, 2H). ^{13}C NMR

(126 MHz, Chloroform-*d*) δ 178.1, 159.0, 151.0, 150.9, 136.4, 133.1, 128.2, 127.9, 127.9, 127.8, 126.6, 126.5, 126.3, 124.3, 117.9, 116.8, 114.3, 55.4, 39.6, 38.9, 33.6, 20.5. HRMS (m/z, ESI): Calcd. for Chemical Formula: $C_{24}H_{20}ClO_4^-$ $[M-H]^-$: 407.1056, Found: 407.1051. HPLC analysis of the reaction product: Daicel Chiralpak ID, hexane/*iso*-propanol = 95: 5, 1.0 mL/min, λ= 272 nm, retention time:13.92 min (major) and 11.77 min (minor).

Optimization of Suzuki cross-coupling reaction conditions

Table S1 Ligand[a]

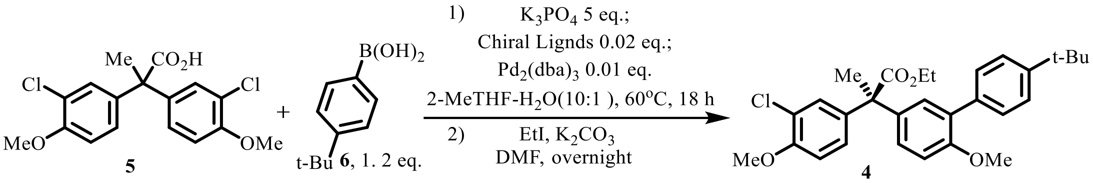

Entry	Ligand sources	Yield[b]	er[c]	HPLC[d]	HPLC[e]
1	**(*S*a)-L1**	32%	35.5:64.5		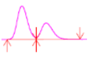
2	**(*R*a)-L6**	36%	70.5:29.5	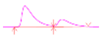	
3	**(*R*a)-L7**	50%	73.5:26.5	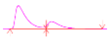	
4	**(*R*a, *S*)-L8**	76%	95.5:4.5	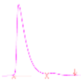	
5	**(*R*a, *S*)-L9**	49%	90.5:9.5	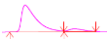	
6	**(*R*a, *S*, *S*)-L10**	66%	94:6	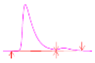	
7	**(*R*a, *S*)-L11**	73%	97.5:2.5	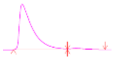	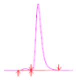
8	**(*R*a, *S*)-L12**	65%	92.5:7.5	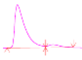	
9	**(*R*a, *S*)-L13**	49%	76.5:23.5	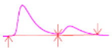	
10	**(*S*a, *S*)-L14**	70%	18:82	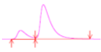	

[a]Unless otherwise stated, the reactions were performed with the substrate **5** (0.1 mmol), 4-tert-butylphenylboronic acid (0.12 mmol), $Pd_2(dba)_3$ (1.0 mol%), Ligand sources (2.0 mol%), K_3PO_4 (0.5 mmol) in 2.0 mL THF and 0.2 mL H_2O at 60°C for 18 h. [b]The yield of product **4** was isolated yield by column chromatography. [c]Determined using chiral chromatography. [d]HPLC trace for acid product. [e]HPLC trace for ethyl ester product.

Table S2 Bases[a]

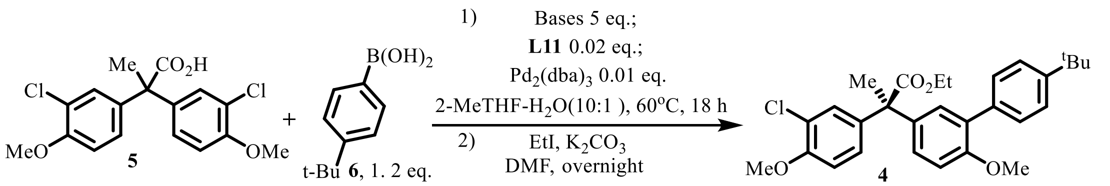

Entry	Bases	Yield[b]	er[c]
1	K_3PO_4	73%	97.5:2.5
3	KOH	58%	97:3
4	NaOH	65%	95:5
5	CsOH (50% in H_2O)	63%	96:4
6	LiOH	55%	85.5:14.5
7	Li_2CO_3	trace	
8	$Ca(OH)_2$	29%	84.5:15.5
9	$Ba(OH)_2$	39%	91.5:8.5

[a]Unless otherwise stated, the reactions were performed with the substrate **5** (0.1 mmol), 4-tert-butylphenylboronic acid (0.12 mmol), $Pd_2(dba)_3$ (1.0 mol%), **L11** (2.0 mol%), Bases (0.5 mmol) in 2.0 mL THF and 0.2 mL H_2O at 60°C for 18 h. [b]The yield of product **4** was isolated yield by column chromatography. [c]Determined using chiral chromatography.

General procedure for Suzuki cross-coupling desymmetrization reactions of acyclic substrates

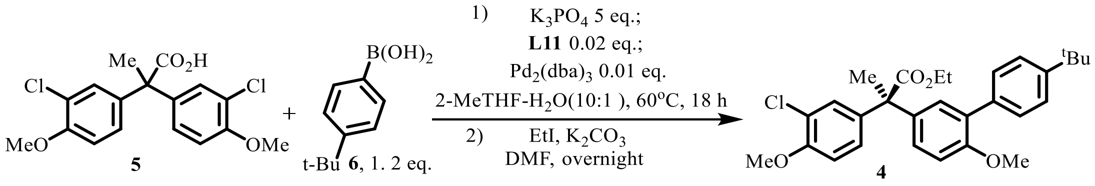

Under N_2 atmosphere, a mixture of $Pd_2(dba)_3$ (2.3 mg, 0.0025 mmol) and **L11** (3.2 mg, 0.005 mmol) was added to 0.5 mL 2-MeTHF, the mixture was then stirred at room temperature for 20 min. The resulting metal-ligand complex solution was added to a reaction flask containing 4.5 mL 2-MeTHF solution of 2,2-bis(3-chloro-4-methoxyphenyl)-propanoic acid **5** (88.5 mg, 0.25 mmol) and 4-tert-butylphenylboronic acid **6** (53.4 mg, 0.3 mmol), and K_3PO_4 (265 mg, 1.25 mmol), followed by addition of 0.50 mL H_2O. Then the resulting reaction mixture was stirred at 60°C for 18 h. The reaction was then quenched with water, neutralized to pH 3 – 5 with 1.0 M HCl $_{(aq.)}$, and extracted with ethyl acetate (4.0 mL X 3) three times. The combined organic phase was washed with brine, dried over Na_2SO_4, and concentrated in vacuo. After that, the obtained crude product was dissolved in DMF (1.0 mL), and treated with EtI (78 mg, 0.5 mmol) and K_2CO_3 (138 mg, 1.0 mmol). The reaction mixture was stirred overnight at room temperature. Then the mixture was diluted with ethyl acetate (20.0 mL) and washed with water, brine, and dried over Na_2SO_4. The organic layer was concentrated in vacuo and purified by flash column chromatography with hexane and acetone as the eluents to give the desired product.

Characterization data of desymmetrization products of Suzuki cross-coupling reactions

Ethyl (*S*)-2-(4'-(tert-butyl)-6-methoxy-[1,1'-biphenyl]-3-yl)-2-(3-chloro-4-methoxyphenyl)-propanoate (4)

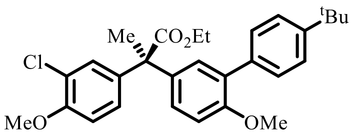

Colorless oil, 88 mg, 73%, $[\alpha]_D^{20}$ = -1.20 (*c* 1.000 $CHCl_3$) for 97.5:2.5 er. ^{1}H NMR (500 MHz, Chloroform-*d*) δ 7.45 – 7.40 (m, 4H), 7.30 (d, *J* = 2.4 Hz, 1H), 7.19 (d, *J* = 2.5 Hz, 1H), 7.19 – 7.11 (m, 2H), 6.90 (d, *J* = 8.6 Hz, 1H), 6.85 (d, *J* = 8.7 Hz, 1H), 4.22 (q, *J* = 7.1 Hz, 2H), 3.89 (s, 3H), 3.82 (s, 3H), 1.89 (s, 3H), 1.35 (s, 9H), 1.24 (t, *J* = 7.1 Hz, 3H). ^{13}C NMR (126 MHz, Chloroform-*d*) δ 174.9, 155.4, 153.7, 149.8, 138.0, 136.3, 135.4, 130.6, 130.02, 129.98, 129.1, 127.6, 127.5, 125.0, 121.9, 111.4, 110.6, 61.4, 56.1, 55.6, 55.1, 34.5, 31.4, 27.2, 14.1. HRMS (m/z, ESI): Calcd. for Chemical Formula: $C_{29}H_{33}ClNaO_4^+$ $[M+Na]^+$: 503.1960, Found: 530.1955. HPLC analysis of the reaction product: Daicel Chiralpak IB, hexane/*iso*-propanol = 99: 1, 0.6 mL/min, λ= 287 nm, retention time: 11.67 min (major) and 11.17 min (minor).

Ethyl (*S*)-2-(3-chloro-4-methoxyphenyl)-2-(4'-(diphenylamino)-6-methoxy-[1,1'-bipheny-l]-3-yl)-propanoate (7)

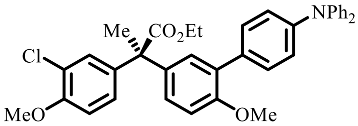

White solid, 110 mg, 74%, $[\alpha]_D^{20}$ = +9.81 (*c* 1.000 $CHCl_3$) for 98.5:1.5 er. ^{1}H NMR (400 MHz, Chloroform-*d*) δ 7.35 – 7.31 (m, 2H), 7.26 – 7.24 (m, 1H), 7.24 – 7.20 (m, 4H), 7.16 (d, *J* = 2.6 Hz, 1H), 7.13 – 7.08 (m, 5H), 7.07 (dd, *J* = 2.5, 1.3 Hz, 1H), 7.06 – 7.02 (m, 2H), 7.01 – 6.97 (m, 2H), 6.87 (d, *J* = 8.7 Hz, 1H), 6.82

(d, J = 8.7 Hz, 1H), 4.18 (q, J = 7.1 Hz, 2H), 3.86 (s, 3H), 3.80 (s, 3H), 1.86 (s, 3H), 1.20 (t, J = 7.1 Hz, 3H). ^{13}C NMR (101 MHz, Chloroform-*d*) δ 174.9, 155.3, 153.7, 147.8, 146.7, 138.0, 136.3, 132.2, 130.29, 130.25, 130.0, 129.7, 129.2, 127.52, 127.48, 124.5, 123.0, 122.9, 121.9, 111.4, 110.7, 61.4, 56.1, 55.6, 55.1, 27.2, 14.1. HRMS (m/z, ESI): Calcd. for Chemical Formula: $C_{37}H_{35}ClNO_4^+$ $[M+H]^+$: 592.2249, Found: 592.2252. HPLC analysis of the reaction product: Daicel Chiralpak IA, hexane/*iso*-propanol = 99: 1, 1.0 mL/min, λ= 313 nm, retention time: 14.37 min (major) and 19.62 min (minor).

Ethyl (*S*)-2-(3-chloro-4-methoxyphenyl)-2-(6-methoxy-4'-nitro-[1,1'-biphenyl]-3-yl)-propanoate (8)

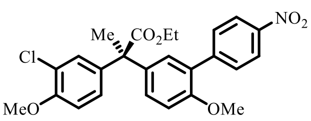

Yellow solid, 94 mg, 80%, $[\alpha]_D^{20}$ = +6.38 (*c* 1.000 $CHCl_3$) for 98:2 er. ^{1}H NMR (400 MHz, Chloroform-*d*) δ 8.26 – 8.18 (m, 2H), 7.67 – 7.59 (m, 2H), 7.27 (d, J = 2.4 Hz, 1H), 7.23 (dd, J = 8.7, 2.6 Hz, 1H), 7.18 (d, J = 2.5 Hz, 1H), 7.12 (dd, J = 8.7, 2.4 Hz, 1H), 6.95 (d, J = 8.7 Hz, 1H), 6.87 (d, J = 8.7 Hz, 1H), 4.22 (q, J = 7.1 Hz, 2H), 3.90 (s, 3H), 3.84 (s, 3H), 1.90 (s, 3H), 1.23 (t, J = 7.1 Hz, 3H). ^{13}C NMR (101 MHz, Chloroform-*d*) δ 174.7, 155.3, 153.8, 146.7, 145.3, 137.6, 136.8, 130.4, 130.3, 129.9, 129.6, 127.8, 127.3, 123.2, 122.1, 111.5, 111.0, 61.5, 56.2, 55.7, 55.0, 27.1, 14.1. HRMS (m/z, ESI): Calcd. for Chemical Formula: $C_{25}H_{24}ClNNaO_6^+$ $[M+Na]^+$: 492.1184, Found: 492.1185. HPLC analysis of the reaction product: Daicel Chiralpak IA, hexane/*iso*-propanol = 99: 1, 1.0 mL/min, λ= 319 nm, retention time: 26.09 min (major) and 30.25 min (minor).

Ethyl (*S*)-2-(3-chloro-4-methoxyphenyl)-2-(4'-cyano-6-methoxy-[1,1'-biphenyl]-3-yl)-propanoate (9)

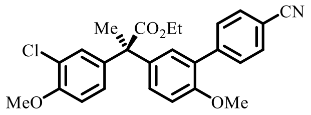

Colorless oil, 87 mg, 78%, $[\alpha]_D^{20}$ = +2.73 (*c* 1.000 $CHCl_3$) for 98:2 er. ^1H NMR (400 MHz, Chloroform-*d*) δ 7.67 – 7.62 (m, 2H), 7.59 – 7.54 (m, 2H), 7.25 (d, *J* = 1.9 Hz, 1H), 7.20 (dd, *J* = 8.6, 2.6 Hz, 1H), 7.13 (d, *J* = 2.5 Hz, 1H), 7.10 (dd, *J* = 8.7, 2.4 Hz, 1H), 6.93 (d, *J* = 8.7 Hz, 1H), 6.85 (d, *J* = 8.7 Hz, 1H), 4.20 (q, *J* = 7.1 Hz, 2H), 3.88 (s, 3H), 3.81 (s, 3H), 1.88 (s, 3H), 1.22 (t, *J* = 7.1 Hz, 3H). ^{13}C NMR (101 MHz, Chloroform-*d*) δ 174.7, 155.2, 153.8, 143.2, 137.6, 136.8, 131.8, 130.3, 129.9, 129.3, 128.2, 127.3, 122.0, 119.1, 111.5, 111.0, 110.5, 61.5, 56.2, 55.6, 55.0, 27.2, 14.1. HRMS (m/z, ESI): Calcd. for Chemical Formula: $C_{26}H_{24}ClNNaO_4^+$ $[M+Na]^+$: 472.1286, Found: 472.1285. HPLC analysis of the reaction product: Daicel Chiralpak IA, hexane/*iso*-propanol = 99: 1, 1.0 mL/min, λ= 297 nm, retention time: 35.27 min (major) and 37.31 min (minor).

Ethyl (*S*)-5'-(2-(3-chloro-4-methoxyphenyl)-1-ethoxy-1-oxopropan-2-yl)-2'-methoxy-[1,1'-biphenyl]-4-carboxylate (10)

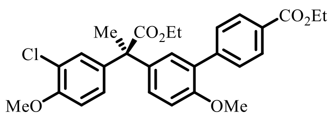

Colorless oil, 93 mg, 75%, $[\alpha]_D^{20}$ = -3.00 (*c* 1.000 $CHCl_3$) for 97.5:2.5 er. ^1H NMR (400 MHz, Chloroform-*d*) δ 8.09 – 8.02 (m, 2H), 7.57 – 7.52 (m, 2H), 7.29 (d, *J* = 2.4 Hz, 1H), 7.20 – 7.15 (m, 2H), 7.12 (dd, *J* = 8.6, 2.4 Hz, 1H), 6.93 (d, *J* = 9.4 Hz, 1H), 6.86 (d, *J* = 8.7 Hz, 1H), 4.39 (q, *J* = 7.1 Hz, 2H), 4.21 (q, *J* = 7.1 Hz, 2H), 3.89 (s, 3H), 3.82 (s, 3H), 1.90 (s, 3H), 1.40 (t, *J* = 7.1 Hz, 3H), 1.23 (t, *J* = 7.1 Hz, 3H). ^{13}C NMR (101 MHz, Chloroform-*d*) δ 174.8, 166.6, 155.3, 153.8, 143.1, 137.7, 136.6, 130.4, 129.9, 129.5, 129.23, 129.17, 128.9, 128.7, 127.4, 122.0, 111.5, 110.9, 61.5, 60.9, 56.1, 55.6, 55.1, 27.2,

14.4, 14.1. HRMS (m/z, ESI): Calcd. for Chemical Formula: $C_{28}H_{30}ClO_6^+$ $[M+H]^+$: 497.1725, Found: 497.1725. HPLC analysis of the reaction product: Daicel Chiralpak IA, hexane/*iso*-propanol = 99: 1, 1.0 mL/min, λ= 268 nm, retention time: 30.47 min (major) and 34.58 min (minor).

Ethyl (*S*)-2-(3'-(benzyloxy)-6-methoxy-[1,1'-biphenyl]-3-yl)-2-(3-chloro-4-methoxyphenyl)-propanoate (11)

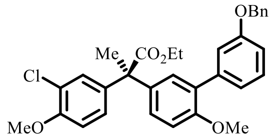

White solid, 103 mg, 78%, $[\alpha]_D^{20}$ = +2.32 (*c* 1.000 $CHCl_3$) for 97:3 er. ^{1}H NMR (400 MHz, Chloroform-*d*) δ 7.48 – 7.42 (m, 2H), 7.41 – 7.37 (m, 2H), 7.36 – 7.28 (m, 3H), 7.18 (d, *J* = 2.6 Hz, 1H), 7.16 – 7.10 (m, 3H), 7.09 – 7.07 (m, 1H), 6.96 – 6.96 (m, 1H), 6.91 (d, *J* = 8.6 Hz, 1H), 6.86 (d, *J* = 8.7 Hz, 1H), 5.09 (s, 2H), 4.22 (q, *J* = 7.1 Hz, 2H), 3.89 (s, 3H), 3.79 (s, 3H), 1.90 (s, 3H), 1.24 (t, *J* = 7.1 Hz, 3H). ^{13}C NMR (101 MHz, Chloroform-*d*) δ 174.9, 158.5, 155.3, 153.7, 139.8, 137.9, 137.2, 136.4, 130.4, 130.0, 129.0, 128.6, 128.1, 127.9, 127.5, 127.4, 122.3, 121.9, 116.3, 113.5, 111.5, 110.8, 70.0, 61.4, 56.1, 55.6, 55.1, 27.2, 14.1. HRMS (m/z, ESI): Calcd. for Chemical Formula: $C_{32}H_{32}ClO_5^+$ $[M+H]^+$: 531.1933, Found: 531.1931. HPLC analysis of the reaction product: Daicel Chiralpak IA, hexane/*iso*-propanol = 99: 1, 1.0 mL/min, λ= 287 nm, retention time: 20.71 min (major) and 24.20 min (minor).

Ethyl (*S*)-5'-(2-(3-chloro-4-methoxyphenyl)-1-ethoxy-1-oxopropan-2-yl)-2'-methoxy-[1,1'-bip-henyl]-3-carboxylate (12)

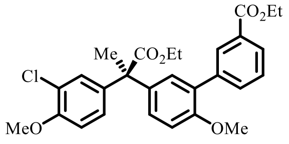

Colorless oil, 100 mg, 81%, $[\alpha]_D^{20}$ = -14.50 (*c* 1.000 $CHCl_3$) for 97.5:2.5 er. ^{1}H NMR (500 MHz, Chloroform-*d*) δ 8.14 (t, *J* = 1.8 Hz, 1H), 8.00 – 7.98 (m, 1H), 7.69 – 7.63 (m, 1H), 7.45 (t, *J* = 7.7 Hz, 1H), 7.29 (dd, *J* = 2.4, 0.8 Hz, 1H), 7.17

(d, J = 8.3 Hz, 2H), 7.14 – 7.08 (m, 1H), 6.92 (d, J = 8.3 Hz, 1H), 6.86 (d, J = 8.7 Hz, 1H), 4.38 (q, J = 7.1 Hz, 2H), 4.22 (q, J = 7.1 Hz, 2H), 3.89 (s, 3H), 3.82 (s, 3H), 1.90 (s, 3H), 1.39 (t, J = 7.1 Hz, 3H), 1.24 (t, J = 7.1 Hz, 3H). ^{13}C NMR (126 MHz, Chloroform-*d*) δ 174.8, 166.7, 155.3, 153.7, 138.7, 137.8, 136.5, 134.0, 130.6, 130.4, 130.4, 129.9, 129.3, 128.5, 128.1, 128.0, 127.4, 121.9, 111.4, 110.8, 61.5, 60.9, 56.1, 55.7, 55.1, 27.2, 14.4, 14.1. HRMS (m/z, ESI): Calcd. for Chemical Formula: $C_{28}H_{29}ClNaO_6^+$ $[M+Na]^+$: 519.1545, Found: 519.1546. HPLC analysis of the reaction product: Daicel Chiralpak IA, hexane/*iso*-propanol = 99: 1, 1.0 mL/min, λ= 287 nm, retention time: 22.32 min (major) and 26.91 min (minor).

Ethyl (*S*)-2-(3-chloro-4-methoxyphenyl)-2-(2',6-dimethoxy-[1,1'-biphenyl]-3-yl)-propanoate (13)

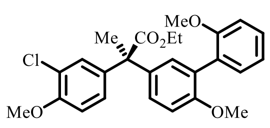

White solid, 86 mg, 76%, $[\alpha]_D^{20}$ = -4.43 (*c* 0.500 $CHCl_3$) for 98:2 er. ^{1}H NMR (400 MHz, Chloroform-*d*) δ 7.34 – 7.28 (m, 2H), 7.23 (dd, J = 7.5, 1.8 Hz, 1H), 7.17 – 7.13 (m, 2H), 7.11 (d, J = 2.6 Hz, 1H), 7.02 – 6.94 (m, 2H), 6.91 (d, J = 8.6 Hz, 1H), 6.85 (d, J = 8.7 Hz, 1H), 4.20 (q, J = 7.1 Hz, 2H), 3.89 (s, 3H), 3.78 (s, 3H), 3.76 (s, 3H), 1.89 (s, 3H), 1.22 (t, J = 7.1 Hz, 3H). ^{13}C NMR (101 MHz, Chloroform-*d*) δ 175.0, 157.0, 155.8, 153.6, 138.1, 135.6, 131.6, 131.5, 130.1, 128.7, 127.7, 127.6, 127.5, 127.2, 121.8, 120.3, 111.4, 111.3, 110.5, 61.3, 56.1, 55.7, 55.7, 55.1, 27.2, 14.1. HRMS (m/z, ESI): Calcd. for Chemical Formula: $C_{26}H_{27}ClNaO_5^+$ $[M+Na]^+$: 477.1439, Found: 477.1438. A sample of the ester was hydrolyzed to provide the parent carboxylic acid for the HPLC analysis of the reaction product: Daicel Chiralpak IA, hexane/*iso*-propanol = 95: 5, 1.0 mL/min, λ= 283 nm, retention time: 18.11 min (major) and 16.24 min (minor).

Ethyl (*S*)-2-(3-chloro-4-methoxyphenyl)-2-(2',5'-dimethyl)-[1,1'-biphenyl]-3-yl)-propanoate (14)

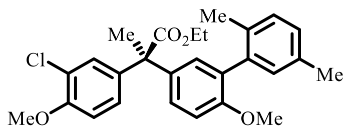

White solid, 86 mg, 76%, $[\alpha]_D^{20}$ = -6.31 (*c* 0.500 $CHCl_3$) for 98:2 er. ^{1}H NMR (500 MHz, Chloroform-*d*) δ 7.27 (d, *J* = 5.7 Hz, 1H), 7.18 – 7.10 (m, 3H), 7.07 – 6.96 (m, 3H), 6.87 (dd, *J* = 18.3, 8.7 Hz, 2H), 4.20 (q, *J* = 7.1 Hz, 2H), 3.89 (s, 3H), 3.77 (s, 3H), 2.32 (s, 3H), 2.08 (s, 3H), 1.88 (s, 3H), 1.22 (t, *J* = 7.1 Hz, 3H). ^{13}C NMR (126 MHz, Chloroform-*d*) δ 174.9, 155.5, 153.6, 138.2, 134.8, 133.6, 130.9, 130.7, 130.5, 130.1, 129.6, 128.2, 127.6, 127.4, 121.9, 111.4, 110.1, 61.4, 56.1, 55.5, 55.1, 27.2, 21.0, 19.5, 14.1. HRMS (m/z, ESI): Calcd. for Chemical Formula: $C_{27}H_{29}ClNaO_4^+$ $[M+Na]^+$: 475.1647, Found: 475.1650. A sample of the ester was hydrolyzed to provide the parent carboxylic acid for the HPLC analysis of the reaction product: Daicel Chiralpak IA, hexane/*iso*-propanol = 95: 5, 1.0 mL/min, λ= 279 nm, retention time: 12.09 min (major) and 11.00 min (minor).

(*S*)-2-(3-chloro-4-methoxyphenyl)-2-(6-methoxy-4'-((trimethylsilyl)ethynyl)-[1,1'-biphenyl]-3-yl)-propanoic acid (15)

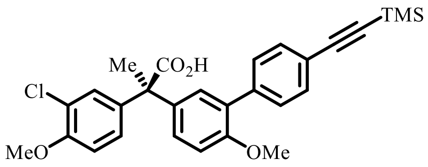

White solid, 394 mg, 80%, (1.0 mmol). ^{1}H NMR (500 MHz, Chloroform-*d*) δ 7.49 – 7.45 (m, 2H), 7.44 – 7.40 (m, 2H), 7.31 (d, *J* = 2.4 Hz, 1H), 7.20 (d, *J* = 8.0 Hz, 2H), 7.15 (dd, *J* = 8.6, 2.5 Hz, 1H), 6.92 (dd, *J* = 8.0, 1.0 Hz, 1H), 6.87 (d, *J* = 8.8 Hz, 1H), 3.89 (s, 3H), 3.80 (s, 3H), 1.91 (s, 3H), 0.26 (s, 9H). ^{13}C NMR (126 MHz, Chloroform-*d*) δ 180.4, 155.5, 153.9, 138.5, 137.1, 135.6, 131.6, 130.2, 129.9, 129.6, 129.4, 128.5, 127.5, 122.1, 121.8, 111.5, 111.0, 105.2, 94.4, 56.2, 55.7, 55.0, 27.0, 24.8. HRMS (m/z, ESI): Calcd. for Chemical Formula: $C_{28}H_{28}ClO_4Si^-$ $[M-H]^-$: 491.1451, Found: 491.1449.

Dodecyl (*S*)-2-(3-chloro-4-methoxyphenyl)-2-(4'-ethynyl-6-methoxy-[1,1'-biphenyl]-3-yl)-propanoate (SI-15)

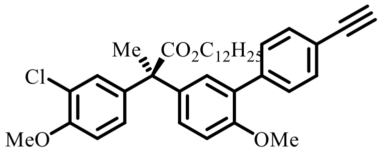

The obtained product **15** was dissolved in MeOH (2.0 mL), and treated with K_2CO_3 (552 mg, 4.0 mmol). The reaction mixture was stirred for 1.0 h at room temperature. Then, the reaction was quenched with H_2O, and diluted with ethyl acetate (20.0 mL). The mixture was washed with water, brine, and dried over Na_2SO_4. The organic layer was concentrated in vacuo and directly gone to next step. The above obtained crude product was dissolved in DMF (3.0 mL), and treated with 1-iodododecane (888 mg, 3.0 mmol) and K_2CO_3 (552 mg, 4.0 mmol). The reaction mixture was stirred overnight at room temperature. Then the mixture was diluted with ethyl acetate (20.0 mL) and washed with water, brine, and dried over Na_2SO_4. The organic layer was concentrated in vacuo and purified by flash column chromatography with hexane and acetone (50:1 – 10:1) to give the desired product **SI-15**, Colorless oil, 182 mg, 39%, two steps. $[\alpha]_D^{20}$ = -9.71 (*c* 2.200 $CHCl_3$) for 94.5:5.5 er. ^1H NMR (400 MHz, Chloroform-*d*) δ 7.53 – 7.48 (m, 2H), 7.46 – 7.42 (m, 2H), 7.29 (d, *J* = 2.4 Hz, 1H), 7.15 (dd, *J* = 6.8, 2.5 Hz, 2H), 7.11 (dd, *J* = 8.7, 2.4 Hz, 1H), 6.93 – 6.89 (m, 1H), 6.85 (d, *J* = 8.7 Hz, 1H), 4.14 (t, *J* = 6.5 Hz, 2H), 3.89 (s, 3H), 3.81 (s, 3H), 3.09 (s, 1H), 1.89 (s, 3H), 1.32 – 1.16 (m, 20H), 0.91 – 0.86 (m, 3H). ^{13}C NMR (101 MHz, Chloroform-*d*) δ 174.9, 155.3, 153.7, 139.0, 137.8, 136.6, 131.8, 130.3, 130.0, 129.5, 129.3, 128.4, 127.4, 121.9, 120.7, 111.4, 110.8, 83.8, 65.6, 56.1, 55.6, 55.2, 31.9, 29.7, 29.7, 29.6, 29.5, 29.4, 29.2, 28.5, 27.2, 25.9, 22.7, 14.1. HRMS (m/z, ESI): Calcd. for Chemical Formula: $C_{37}H_{46}ClO_4^+$ $[M+H]^+$: 589.3079, Found: 589.3084. HPLC analysis of the

reaction product: Daicel Chiralpak IA, hexane/*iso*-propanol = 99: 1, 1.0 mL/min, λ= 306 nm, retention time: 8.99 min (major) and 9.72 min (minor).

Ethyl (*S*)-2-(3-chloro-4-methoxyphenyl)-2-(4-methoxy-3-(9-phenyl-9H-carbazol-1-yl)-phenyl) -propanoate (16)

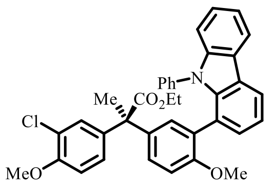

White solid, 100 mg, 68%, $[\alpha]_D^{20}$ = +1.01 (*c* 0.500 $CHCl_3$) for 95:5 er. ^1H NMR (400 MHz, Chloroform-*d*) δ 8.24 (dd, *J* = 1.7, 0.6 Hz, 1H), 8.16 – 8.14 (m, 1H), 7.64 – 7.61 (m, 1H), 7.61 – 7.57 (m, 3H), 7.55 (dd, *J* = 8.5, 1.7 Hz, 1H), 7.50 – 7.39 (m, 4H), 7.36 (d, *J* = 2.4 Hz, 1H), 7.32 – 7.26 (m, 2H), 7.18 – 7.15 (m, 2H), 6.96 (d, *J* = 8.7 Hz, 1H), 6.87 (d, *J* = 8.7 Hz, 1H), 4.28 – 4.21 (m, 2H), 3.90 (s, 3H), 3.85 (s, 3H), 1.94 (s, 3H), 1.27 (t, *J* = 7.1 Hz, 3H).^{13}C NMR (101 MHz, Chloroform-*d*) δ 175.0, 155.5, 153.7, 141.2, 140.1, 138.0, 137.8, 136.4, 131.0, 130.9, 130.3, 130.1, 129.9, 127.8, 127.5, 127.5, 127.1, 125.9, 123.5, 123.3, 121.9, 121.3, 120.4, 119.9, 111.4, 110.8, 109.8, 109.3, 61.4, 56.2, 55.7, 55.2, 27.3, 14.1. HRMS (m/z, ESI): Calcd. for Chemical Formula: $C_{37}H_{33}ClNO_4^+$ $[M+H]^+$: 590.2093, Found: 590.2092. HPLC analysis of the reaction product: Daicel Chiralpak IA, hexane/*iso*-propanol = 99: 1, 1.0 mL/min, λ= 294 nm, retention time: 28.88 min (major) and 41.79 min (minor).

Ethyl (*S*)-2-(3-chloro-4-methoxyphenyl)-2-(3-(dibenzo-[b,d]furan-4-yl)-4-methoxyphenyl) propanoate (17)

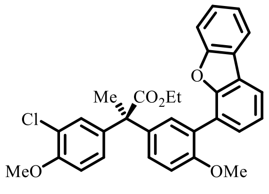

White solid, 105 mg, 82%, $[\alpha]_D^{20}$ = -7.10 (*c* 1.000 $CHCl_3$) for 98:2 er. ^1H NMR (400 MHz, Chloroform-*d*) δ 7.99 – 7.90 (m, 2H), 7.53 – 7.47 (m, 2H), 7.46 – 7.31 (m, 5H), 7.28 – 7.24 (m, 1H), 7.19 (dd, *J* = 8.7, 2.4 Hz,

1H), 7.02 (d, J = 8.7 Hz, 1H), 6.87 (d, J = 8.7 Hz, 1H), 4.23 (q, J = 7.1 Hz, 2H), 3.89 (s, 3H), 3.82 (s, 3H), 1.93 (s, 3H), 1.24 (t, J = 7.1 Hz, 3H). ^{13}C NMR (101 MHz, Chloroform-*d*) δ 174.9, 156.2, 155.9, 153.9, 153.7, 137.9, 136.2, 131.4, 130.1, 128.9, 128.6, 127.5, 127.0, 125.1, 124.4, 124.3, 123.0, 122.6, 122.5, 121.9, 120.6, 119.7, 111.7, 111.4, 110.9, 61.4, 56.1, 55.8, 55.1, 27.3, 14.1. HRMS (m/z, ESI): Calcd. for Chemical Formula: $C_{31}H_{27}ClNaO_5^+$ [M+Na]$^+$: 537.1439, Found: 537.1439. HPLC analysis of the reaction product: Daicel Chiralpak IA, hexane/*iso*-propanol = 99: 1, 1.0 mL/min, λ= 287 nm, retention time: 14.76 min (major) and 16.34 min (minor).

Ethyl (*S*)-2-(3-chloro-4-methoxyphenyl)-2-(3-(dibenzo[b,d]thiophen-2-yl)-4-methoxyphenyl) -propanoate (18)

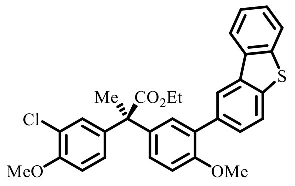

White solid, 95 mg, 72%, $[\alpha]_D^{20}$ = -8.27 (*c* 0.500 $CHCl_3$) for 97.5:2.5 er. ^{1}H NMR (400 MHz, Chloroform-*d*) δ 8.24 (dd, J = 1.8, 0.6 Hz, 1H), 8.19 – 8.12 (m, 1H), 7.91 – 7.81 (m, 2H), 7.59 (dd, J = 8.3, 1.7 Hz, 1H), 7.49 – 7.42 (m, 2H), 7.34 (d, J = 2.4 Hz, 1H), 7.29 (d, J = 2.5 Hz, 1H), 7.21 – 7.15 (m, 2H), 6.97 (d, J = 8.7 Hz, 1H), 6.87 (d, J = 8.7 Hz, 1H), 4.24 (q, J = 7.1 Hz, 2H), 3.90 (s, 3H), 3.85 (s, 3H), 1.94 (s, 3H), 1.27 (t, J = 7.1 Hz, 3H). ^{13}C NMR (101 MHz, Chloroform-*d*) δ 174.9, 155.4, 153.8, 139.8, 138.3, 137.9, 136.6, 135.6, 135.5, 134.9, 130.7, 130.1, 130.0, 128.5, 128.1, 127.4, 126.7, 124.3, 122.9, 122.5, 122.2, 122.0, 121.6, 111.5, 110.9, 61.5, 56.2, 55.7, 55.1, 27.3, 14.1. HRMS (m/z, ESI): Calcd. for Chemical Formula: $C_{31}H_{27}ClNaO_4S^+$ [M+Na]$^+$: 553.1211, Found: 553.1208. HPLC analysis of the reaction product: Daicel Chiralpak IA, hexane/*iso*-propanol = 99: 1, 1.0 mL/min, λ= 289 nm, retention time: 21.69 min (major) and 23.30 min (minor).

Ethyl (*S*)-2-(3-chloro-4-methoxyphenyl)-2-(3-(dibenzo[b,d]thiophen-4-yl)-4-(oxidaneyl)-phenyl)-propanoate (19)

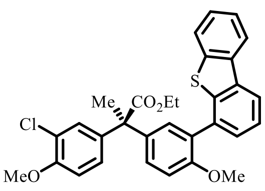

White solid, 103 mg, 78%, $[\alpha]_D^{20}$ = +1.70 (*c* 0.500 $CHCl_3$) for 98.5:1.5 er. ^{1}H NMR (400 MHz, Chloroform-*d*) δ 8.18 – 8.11 (m, 2H), 7.84 – 7.78 (m, 1H), 7.54 – 7.40 (m, 4H), 7.37 (dd, *J* = 2.5, 0.6 Hz, 2H), 7.29 – 7.26 (m, 1H), 7.17 (dd, *J* = 8.7, 2.4 Hz, 1H), 7.00 (d, *J* = 8.7 Hz, 1H), 6.86 (d, *J* = 8.7 Hz, 1H), 4.22 (q, *J* = 7.1 Hz, 2H), 3.88 (s, 3H), 3.79 (s, 3H), 1.92 (s, 3H), 1.23 (t, *J* = 7.1 Hz, 3H). ^{13}C NMR (101 MHz, Chloroform-*d*) δ 174.8, 155.4, 153.7, 140.2, 139.7, 137.8, 136.4, 135.9, 135.8, 133.4, 130.6, 130.3, 128.72, 128.65, 128.2, 127.3, 126.6, 124.4, 124.2, 122.6, 122.0, 121.6, 120.4, 111.4, 111.0, 61.5, 56.1, 55.7, 55.2, 27.3, 14.1. HRMS (m/z, ESI): Calcd. for Chemical Formula: $C_{31}H_{27}ClNaO_4S^+$ $[M+Na]^+$: 553.1211, Found: 553.1207. A sample of the ester was hydrolyzed to provide the parent carboxylic acid for the HPLC analysis of the reaction product: Daicel Chiralpak IA, hexane/*iso*-propanol = 95: 5, 1.0 mL/min, λ= 286 nm, retention time: 19.53 min (major) and 24.12 min (minor).

Ethyl (*S*)-2-(3-chloro-4-methoxyphenyl)-2-(4-methoxy-3-(thianthren-1-yl)-phenyl)-propanoate (20)

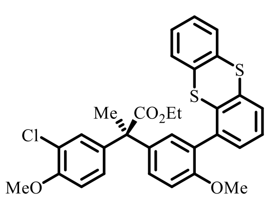

White solid, 118 mg, 84%, $[\alpha]_D^{20}$ = +5.00 (*c* 0.500 $CHCl_3$) for 98.5:1.5 er. ^{1}H NMR (400 MHz, Chloroform-*d*) δ 7.50 – 7.47 (m, 2H), 7.38 – 7.26 (m, 3H), 7.25 – 7.11 (m, 5H), 7.06 (d, *J* = 2.5 Hz, 1H), 6.96 (dd, *J* = 8.8, 3.7 Hz, 1H), 6.85 (dd, *J* = 8.8, 4.8 Hz, 1H), 4.21 (q, *J* = 7.1 Hz, 2H), 3.88 (s, 3H), 3.81 (s, 3H), 1.90 (s, 3H), 1.21 (t, *J* = 7.1 Hz, 3H). ^{13}C NMR (126 MHz, Chloroform-*d*) δ 174.8, 153.7, 138.9, 136.5, 136.3, 136.2, 135.3, 131.1, 130.7, 130.1, 129.8, 128.9, 128.8, 128.5, 128.2, 127.5, 127.4, 127.3,

127.0, 121.9, 111.4, 110.2, 61.4, 56.1, 55.6, 27.2, 14.1. HRMS (m/z, ESI): Calcd. for Chemical Formula: $C_{31}H_{27}ClNaO_4S_2^+$ $[M+Na]^+$: 585.0931, Found: 585.0926. HPLC analysis of the reaction product: Daicel Chiralpak ODH, hexane/*iso*-propanol = 99: 1, 1.0 mL/min, λ= 261 nm, retention time: 25.02 min (major) and 51.09 min (minor).

Ethyl (*S*)-2-(3-(benzo[b]thiophen-2-yl)-4-methoxyphenyl)-2-(3-chloro-4-methoxyphenyl)-Propanoate (21)

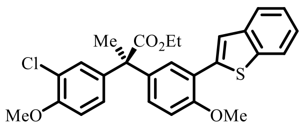

White solid, 42 mg, 35%, $[\alpha]_D^{20}$ = -8.27 (*c* 0.500 $CHCl_3$) for 92.5:7.5 er. ^{1}H NMR (500 MHz, Chloroform-*d*) δ 7.82 – 7.79 (m, 1H), 7.77 (dd, *J* = 7.4, 1.4 Hz, 1H), 7.68 (s, 1H), 7.57 (d, *J* = 2.5 Hz, 1H), 7.36 – 7.27 (m, 3H), 7.15 (dd, *J* = 8.7, 2.5 Hz, 1H), 7.12 (dd, *J* = 8.7, 2.4 Hz, 1H), 6.96 (d, *J* = 8.7 Hz, 1H), 6.87 (d, *J* = 8.8 Hz, 1H), 4.24 (q, *J* = 7.1, 2H), 3.97 (s, 3H), 3.90 (s, 3H), 1.92 (s, 3H), 1.25 (t, *J* = 7.1 Hz, 3H). 13C NMR (126 MHz, Chloroform-*d*) δ 174.8, 155.2, 153.8, 140.1, 139.9, 139.8, 137.7, 136.5, 129.9, 129.2, 128.8, 127.5, 124.2, 124.1, 123.5, 122.8, 122.7, 122.0, 121.8, 111.5, 111.3, 61.6, 56.2, 55.8, 55.1, 27.2, 14.1. HRMS (m/z, ESI): Calcd. for Chemical Formula: $C_{27}H_{25}ClNaO_4S^+$ $[M+Na]^+$: 503.1054, Found: 503.1056. HPLC analysis of the reaction product: Daicel Chiralpak IA, hexane/*iso*-propanol = 99: 1, 1.0 mL/min, λ= 317 nm, retention time: 18.65 min (major) and 19.85 min (minor).

When the reaction was carried out in 2-MeTHF (3 mL), H_2O (0.3 mL), boronic acid (3.6 eq.), K_3PO_4 (10 eq.), Pd_2dba_3 (3 mol%), (*R*, *S*)-**L11**(6 mol%), and 2,2-bis(3-chloro-4-methoxyphenyl)-propanoic acid **5** (0.083 mmol), 23 mg product was obtained, 58% yield, 96:4 er.

Ethyl 2,2-bis(3-(benzo[b]thiophen-3-yl)-4-methoxyphenyl)propanoate

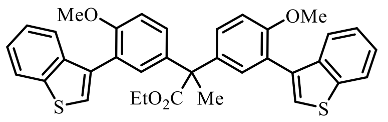

Colorless oil, 6.7 mg, 14%. ^{1}H NMR (400 MHz, Chloroform-*d*) δ 7.80 (dt, *J* = 7.9, 1.0 Hz, 2H), 7.78 – 7.74 (m, 2H), 7.70 (d, *J* = 0.8 Hz, 2H), 7.65 (d, *J* = 2.5 Hz, 2H), 7.35 – 7.27 (m, 4H), 7.21 (dd, *J* = 8.7, 2.5 Hz, 2H), 6.98 – 6.95 (m, 2H), 4.27 (q, *J* = 7.1 Hz, 2H), 3.97 (s, 6H), 1.99 (s, 3H), 1.28 (t, *J* = 7.1 Hz, 3H). ^{13}C NMR (101 MHz, Chloroform-*d*) δ 174.0, 154.1, 139.1, 139.0, 138.8, 135.9, 128.3, 127.9, 123.1, 123.0, 122.4, 121.7, 121.6, 120.8, 110.3, 60.5, 54.7, 54.2, 26.3, 13.2. HRMS (m/z, ESI): Calcd. for Chemical Formula: $C_{35}H_{30}NaO_4S_2^+$ $[M+Na]^+$: 601.1478, Found: 601.1481.

Ethyl (*S*)-2-(3-chloro-4-methoxyphenyl)-2-(3-(2-fluoropyridin-3-yl)-propanoate (22)

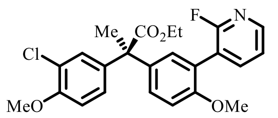

Yellow oil, 41 mg, 37%, $[\alpha]_D^{20}$ = -2.85 (*c* 1.000 $CHCl_3$) for 95.5:4.5 er. ^{1}H NMR (500 MHz, Chloroform-*d*) δ 8.18 – 8.17 (m, 1H), 7.78 – 7.74 (m, 1H), 7.28 (d, *J* = 2.4 Hz, 1H), 7.24 – 7.19 (m, 2H), 7.15 – 7.09 (m, 2H), 6.93 (d, *J* = 8.7 Hz, 1H), 6.86 (d, *J* = 8.7 Hz, 1H), 4.21 (q, *J* = 7.1 Hz, 2H), 3.89 (s, 3H), 3.81 (s, 3H), 1.88 (s, 3H), 1.22 (t, *J* = 7.1 Hz, 3H). ^{13}C NMR (126 MHz, Chloroform-*d*) δ 174.7, 161.7, 159.8, 155.7, 153.8, 146.4 (d, *J* = 14.6 Hz), 142.3 (d, *J* = 4.6 Hz), 137.6, 136.3, 130.8, 129.9, 129.4, 127.4, 122.6 (d, *J* = 4.4 Hz), 121.4 (J = 76.5 Hz), 121.1(d, *J* = 4.3 Hz), 111.5, 110.7, 61.5, 56.1, 55.7, 55.0, 27.2, 14.0. ^{19}F NMR (471 MHz, Chloroform-*d*) δ -68.73 (s, 1F). HRMS (m/z, ESI): Calcd. for Chemical Formula: $C_{24}H_{23}ClFNNaO_4^+$ $[M+Na]^+$: 466.1192, Found: 466.1194. A sample of the ester was hydrolyzed to provide the parent carboxylic acid for the HPLC analysis of the reaction product: Daicel Chiralpak IA, hexane/*iso*-propanol = 95: 5, 1.0 mL/min, λ= 272 nm, retention time: 29.44 min (major) and 36.02 min (minor).

Ethyl (*S*)-2-(3-chloro-4-methoxyphenyl)-2-(4',6-dimethoxy-[1,1'-biphenyl]-3-yl)-propanoate (23)

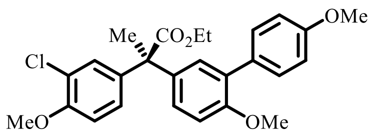

Colorless oil, 90 mg, 79%, $[\alpha]_D^{20}$ = -7.50 (*c* 1.000 $CHCl_3$) for 98:2 er. ^{1}H NMR (400 MHz, Chloroform-*d*) δ 7.44 – 7.40 (m, 2H), 7.30 (d, *J* = 2.4 Hz, 1H), 7.16 (d, *J* = 2.5 Hz, 1H), 7.14 – 7.10 (m, 2H), 6.96 – 6.92 (m, 2H), 6.90 (d, *J* = 8.6 Hz, 1H), 6.86 (d, *J* = 8.7 Hz, 1H), 4.22 (q, *J* = 7.1 Hz, 2H), 3.89 (s, 3H), 3.83 (s, 3H), 3.81 (s, 3H), 1.90 (s, 3H), 1.23 (t, *J* = 7.1 Hz, 3H). ^{13}C NMR (101 MHz, Chloroform-*d*) δ 174.9, 158.7, 155.3, 153.7, 137.9, 136.4, 130.8, 130.6, 130.31, 129.99, 129.8, 127.5, 121.9, 113.5, 111.4, 110.7, 61.4, 56.1, 55.6, 55.3, 55.1, 27.2, 14.1. HRMS (m/z, ESI): Calcd. for Chemical Formula: $C_{26}H_{27}ClNaO_5^+$ $[M+Na]^+$: 477.1439, Found: 477.1438. HPLC analysis of the reaction product: Daicel Chiralpak IA, hexane/*iso*-propanol = 99: 1, 1.0 mL/min, λ= 284 nm, retention time: 18.09 min (major) and 20.00 min (minor).

Ethyl (*S*)-2-(3-chloro-4-methoxyphenyl)-2-(4',6-dimethoxy-[1,1'-biphenyl]-3-yl)-hexanoate (24)

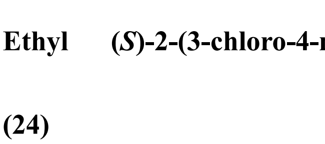

Colorless oil, 74 mg, 60%, $[\alpha]_D^{20}$ = +2.35 (*c* 1.000 $CHCl_3$) for 89:11 er. ^{1}H NMR (400 MHz, Chloroform-*d*) δ 7.45 – 7.40 (m, 2H), 7.34 (d, *J* = 2.4 Hz, 1H), 7.20 – 7.14 (m, 3H), 6.96 – 6.91 (m, 2H), 6.89 (d, *J* = 8.5 Hz, 1H), 6.84 (d, *J* = 8.7 Hz, 1H), 4.17 (q, *J* = 7.1 Hz, 2H), 3.89 (s, 3H), 3.83 (s, 3H), 3.81 (s, 3H), 2.36 – 2.26 (m, 2H), 1.34 – 1.27 (m, 2H), 1.19 (t, *J* = 7.1 Hz, 3H), 1.10 – 1.03 (m, 2H), 0.86 (t, *J* = 7.3 Hz, 3H). ^{13}C NMR (101 MHz, Chloroform-*d*) δ 174.2, 158.7, 155.1, 153.5, 136.7, 135.0, 131.2, 130.9, 130.7, 130.6,

129.5, 128.44, 128.36, 121.6, 113.5, 111.2, 110.4, 61.1, 58.9, 56.1, 55.6, 55.3, 38.0, 27.5, 23.2, 14.1, 14.0. HRMS (m/z, ESI): Calcd. for Chemical Formula: $C_{29}H_{33}ClNaO_5^+$ $[M+Na]^+$: 519.1909, Found: 519.1907. HPLC analysis of the reaction product: Daicel Chiralpak IB, hexane/*iso*-propanol = 98: 2, 0.5 mL/min, λ= 272 nm, retention time: 13.82 min (major) and 13.18 min (minor).

Ethyl (*S*)-2-(3-chloro-4-methoxyphenyl)-2-(4',6-dimethoxy-[1,1'-biphenyl]-3-yl)-4-methylpen-tanoate (25)

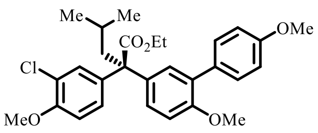

Colorless oil, 63 mg, 51%, $[\alpha]_D^{20}$ = +3.85 (*c* 1.000 $CHCl_3$) for 85.5:14.5 er. ^1H NMR (400 MHz, Chloroform-*d*) δ 7.42 – 7.39 (m, 3H), 7.24 – 7.18 (m, 3H), 6.96 – 6.80 (m, 4H), 4.13 (q, *J* = 7.1 Hz, 2H), 3.89 (s, 3H), 3.83 (s, 3H), 3.81 (s, 3H), 2.31 – 2.26 (m, 2H), 1.44 – 1.35 (m, 1H), 1.17 (t, *J* = 7.1 Hz, 3H), 0.70 (dd, *J* = 6.7, 1.5 Hz, 6H). ^{13}C NMR (101 MHz, Chloroform-*d*) δ 174.2, 158.7, 155.1, 153.5, 137.2, 135.6, 131.2, 130.9, 130.8, 130.6, 129.5, 128.6, 128.5, 121.6, 113.5, 111.1, 110.4, 61.0, 58.5, 56.1, 55.6, 55.3, 46.7, 25.3, 24.1, 14.0. HRMS (m/z, ESI): Calcd. for Chemical Formula: $C_{29}H_{33}ClNaO_5^+$ $[M+Na]^+$: 519.1909, Found: 519.1909. HPLC analysis of the reaction product: Daicel Chiralpak IE, hexane/*iso*-propanol = 99: 1, 0.5 mL/min, λ= 272 nm, retention time: 21.50 min (major) and 24.15 min (minor).

Ethyl (*S*)-2-(3-chloro-4-methoxyphenyl)-2-(4',6-dimethoxy-[1,1'-biphenyl]-3-yl)-3-phenylpro-panoate (26)

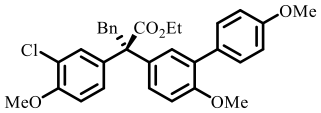

Colorless oil, 65 mg, 49%, $[\alpha]_D^{20}$ = +3.45 (*c* 1.000 $CHCl_3$) for 90.5:9.5 er. ^1H NMR (500 MHz, Chloroform-*d*) δ 7.33 – 7.28 (m,

2H), 7.21 (d, J = 2.3 Hz, 1H), 7.16 – 7.02 (m, 6H), 6.92 – 6.87 (m, 2H), 6.84 (d, J = 8.6 Hz, 1H), 6.77 (d, J = 8.7 Hz, 1H), 6.75 – 6.70 (m, 2H), 4.20 – 4.14 (m, 2H), 3.89 (s, 3H), 3.82 (s, 3H), 3.81 (s, 3H), 3.72 – 3.61 (m, 2H), 1.17 (t, J = 7.1 Hz, 3H). ^{13}C NMR (126 MHz, Chloroform-*d*) δ 173.4, 158.6, 155.3, 153.6, 137.3, 136.2, 134.6, 131.6, 131.2, 131.0, 130.7, 130.6, 129.3, 128.8, 128.6, 127.6, 126.4, 121.4, 113.4, 110.9, 110.3, 61.3, 60.7, 56.1, 55.6, 55.3, 44.5, 14.0. HRMS (m/z, ESI): Calcd. for Chemical Formula: $C_{32}H_{31}ClNaO_5^+$ $[M+Na]^+$: 553.1752, Found: 553.1750. HPLC analysis of the reaction product: Daicel Chiralpak IA, hexane/*iso*-propanol = 99: 1, 1.0 mL/min, λ= 272 nm, retention time: 23.89 min (major) and 32.05 min (minor).

Ethyl (S)-2-(3-chloro-4-methoxyphenyl)-2-(4',6-dimethoxy-[1,1'-biphenyl]-3-yl)-3-methylbutanoate (27)

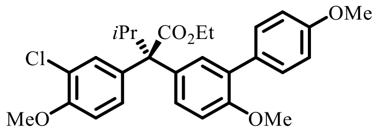

Colorless oil, 66 mg, 55%, $[\alpha]_D^{20}$ = -1.45 (*c* 1.000 $CHCl_3$) for 88.5:11.5 er. ^{1}H NMR (400 MHz, Chloroform-*d*) δ 7.45 – 7.34 (m, 3H), 7.22 – 7.15 (m, 3H), 6.96 – 6.82 (m, 4H), 4.10 (q, J = 7.1 Hz, 2H), 3.91 (s, 3H), 3.83 – 3.82 (m, 6H), 3.28 (p, J = 6.6 Hz, 1H), 1.10 (t, J = 7.1 Hz, 3H), 0.82 (d, J = 6.6 Hz, 6H). ^{13}C NMR (101 MHz, Chloroform-*d*) δ 174.1, 158.6, 155.2, 153.5, 133.6, 132.7, 132.2, 132.0, 130.9, 130.7, 130.1, 129.9, 128.9, 121.1, 113.5, 110.7, 110.0, 64.0, 60.9, 56.1, 55.5, 55.3, 30.6, 18.8, 13.9. HRMS (m/z, ESI): Calcd. for Chemical Formula: $C_{28}H_{31}ClNaO_5^+$ $[M+Na]^+$: 505.1752, Found: 505.1752. HPLC analysis of the reaction product: Daicel Chiralpak IA, hexane/*iso*-propanol = 99: 1, 1.0 mL/min, λ= 272 nm, retention time: 12.02 min (major) and 16.67 min (minor).

Ethyl (*S*)-3-(3-chloro-4-methoxyphenyl)-3-(4',6-dimethoxy-[1,1'-biphenyl]-3-yl)-butanoate (28)

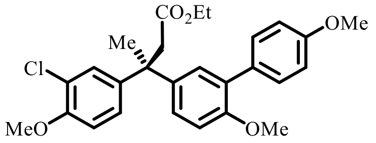

White solid, 60 mg, 51%, $[\alpha]_D^{20}$ = -1.43 (*c* 0.350 $CHCl_3$) for 89.5:10.5 er (THF was solvent). ^1H NMR (500 MHz, Chloroform-*d*) δ 7.43 – 7.39 (m, 2H), 7.25 (d, *J* = 2.4 Hz, 1H), 7.11 – 7.08 (m, 2H), 7.07 (d, *J* = 2.4 Hz, 1H), 6.96 – 6.91 (m, 2H), 6.87 (d, *J* = 8.4 Hz, 1H), 6.83 (d, *J* = 8.7 Hz, 1H), 3.92 (q, *J* = 7.1 Hz, 2H), 3.87 (s, 3H), 3.83 (s, 3H), 3.79 (s, 3H), 3.08 (d, *J* = 1.4 Hz, 2H), 1.85 (s, 3H), 1.01 (t, *J* = 7.1 Hz, 3H). ^{13}C NMR (126 MHz, Chloroform-*d*) δ 171.2, 158.7, 154.8, 153.1, 141.7, 140.2, 131.0, 130.6, 129.7, 129.5, 129.1, 126.53, 126.48, 121.8, 113.5, 111.5, 110.6, 60.1, 56.1, 55.6, 55.3, 46.8, 44.4, 28.6, 14.0. HRMS (m/z, ESI): Calcd. for Chemical Formula: $C_{27}H_{29}ClNaO_5^+$ $[M+Na]^+$: 491.1596, Found: 491.1600. HPLC analysis of the reaction product: Daicel Chiralpak ASH, hexane/*iso*-propanol = 99: 1, 0.5 mL/min, λ= 288 nm, retention time: 31.47 min (major) and 26.28 min (minor).

When the reaction was carried out in 2-MeTHF, 63 mg product was obtained, 53% yield, 86:14 er.

(*S*)-3-(4'-(tert-butyl)-6-methoxy-[1,1'-biphenyl]-3-yl)-3-(3-chloro-4-methoxyphenyl)-butanoic acid (29)

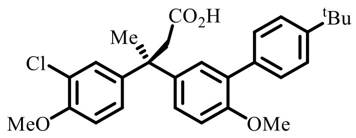

White solid, 54 mg, 46%. $[\alpha]_D^{20}$ = -2.80 (*c* 1.000 $CHCl_3$) for 85.5:14.5 er. ^1H NMR (500 MHz, Chloroform-*d*) δ 7.42 (d, *J* = 0.7 Hz, 4H), 7.23 (d, *J* = 2.4 Hz, 1H), 7.12 (d, *J* = 2.6 Hz, 1H), 7.07 (td, *J* = 8.6, 2.5 Hz, 2H), 6.87 (d, *J* = 8.6 Hz, 1H), 6.82 (d, *J* = 8.7 Hz, 1H), 3.87 (s, 3H), 3.80 (s, 3H), 3.11 (d, *J* = 1.5 Hz, 2H), 1.85 (s, 3H), 1.35 (s, 9H). ^{13}C NMR (126 MHz, Chloroform-*d*) δ 176.2, 154.9, 153.2, 149.8, 141.4,

139.9, 135.5, 130.0, 129.6, 129.2, 129.0, 126.6, 126.4, 125.0, 121.9, 111.6, 110.6, 56.1, 55.5, 46.2, 44.1, 34.5, 31.4, 28.2. HRMS (m/z, ESI): Calcd. for Chemical Formula: $C_{28}H_{31}ClNaO_4^+$ $[M+Na]^+$:489.1803, Found: 489.1801. A sample of the acid was reduced to provide the parent primary alcohol for the HPLC analysis of the reaction product: Daicel Chiralpak IC, hexane/*iso*-propanol = 97: 3, 1.0 mL/min, λ= 289 nm, retention time: 16.52 min (major) and 14.96 min (minor).

(*S*)-4-(4'-(tert-butyl)-6-methoxy-[1,1'-biphenyl]-3-yl)-4-(3-chloro-4-methoxyphenyl)-pentanoic acid (3)

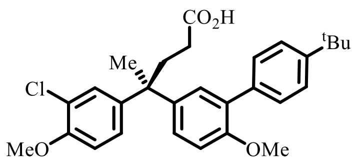

White solid, 74 mg, 62%. $[\alpha]_D^{20}$ = +1.80 (*c* 0.500 $CHCl_3$) for 88.5:11.5 er. ^{1}H NMR (500 MHz, Chloroform-*d*) δ 7.41 (s, 4H), 7.23 (d, *J* = 2.4 Hz, 1H), 7.12 (d, *J* = 2.5 Hz, 1H), 7.09 (dd, *J* = 8.5, 2.5 Hz, 1H), 7.06 (dd, *J* = 8.6, 2.4 Hz, 1H), 6.88 (d, *J* = 8.5 Hz, 1H), 6.83 (d, *J* = 8.6 Hz, 1H), 3.87 (s, 3H), 3.80 (s, 3H), 2.46 – 2.38 (m, 2H), 2.20 – 2.13 (m, 2H), 1.60 (s, 3H), 1.35 (s, 9H). ^{13}C NMR (126 MHz, Chloroform-*d*) δ 154.8, 153.0, 149.8, 142.1, 140.1, 135.6, 130.1, 129.8, 129.2, 129.1, 126.8, 126.5, 125.0, 122.0, 111.7, 110.7, 56.1, 55.6, 44.6, 36.2, 34.5, 31.4, 29.9, 29.7, 27.6. HRMS (m/z, ESI): Calcd. for Chemical Formula: $C_{29}H_{33}ClNaO_4^+$ $[M+Na]^+$: 503.1960, Found: 503.1960. A sample of the acid was esterification to provide the Me-ester for the HPLC analysis of the reaction product: Daicel Chiralpak ODH, hexane/*iso*-propanol = 99: 1, 1.0 mL/min, λ= 288 nm, retention time: 11.71 min (major) and 10.72 min (minor).

Ethyl (*S*)-2-(3-chloro-4-isopropoxyphenyl)-2-(6-isopropoxy-4'-methoxy-[1,1'-biphenyl]-3-yl) -propanoate (30)

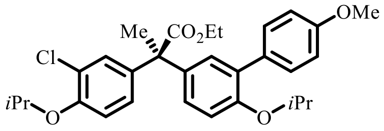

White solid, 64 mg, 50%, $[\alpha]_D^{20}$ = -6.30 (*c* 0.050 $CHCl_3$) for 94.5:5.5 er. ^{1}H NMR (400 MHz, Chloroform-*d*) δ 7.48 – 7.39 (m, 2H), 7.28 (d, *J* = 2.4 Hz, 1H), 7.16 (d, *J* = 2.6 Hz, 1H), 7.08 – 7.05 (m, 2H), 6.94 – 6.82 (m, 4H), 4.53 (p, *J* = 6.1 Hz, 1H), 4.45 (p, *J* = 6.1 Hz, 1H), 4.21 (q, *J* = 7.1 Hz, 2H), 3.84 (s, 3H), 1.88 (s, 3H), 1.38 (d, *J* = 6.1 Hz, 6H), 1.27 (d, *J* = 6.1 Hz, 6H), 1.23 (t, *J* = 7.1 Hz, 3H). ^{13}C NMR (101 MHz, Chloroform-*d*) δ 175.0, 158.5, 153.7, 152.3, 138.0, 136.3, 131.2, 130.8, 130.7, 130.4, 130.1, 127.3, 123.5, 115.0, 114.1, 113.3, 72.0, 70.7, 61.4, 55.2, 55.1, 27.2, 22.1, 14.1. HRMS (m/z, ESI): Calcd. for Chemical Formula: $C_{30}H_{35}ClNaO_5^+$ $[M+Na]^+$: 533.2065, Found: 533.2068. HPLC analysis of the reaction product: Daicel Chiralpak IA, hexane/*iso*-propanol = 99: 1, 1.0 mL/min, λ= 272 nm, retention time: 7.60 min (major) and 8.33 min (minor).

Ethyl (*S*)-2-(4-butoxy-3-chlorophenyl)-2-(6-butoxy-4'-methoxy-[1,1'-biphenyl]-3-yl)-propanoate (31)

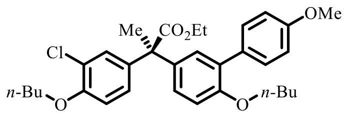

Colorless oil, 72 mg, 54%, $[\alpha]_D^{20}$ = -10.1 (*c* 0.500 $CHCl_3$) for 94.5:5.5 er. ^{1}H NMR (500 MHz, Chloroform-*d*) δ 7.47 – 7.41 (m, 2H), 7.28 (d, *J* = 2.4 Hz, 1H), 7.16 (d, *J* = 2.5 Hz, 1H), 7.09 – 7.06 (m, 2H), 6.94 – 6.90 (m, 2H), 6.87 (d, *J* = 8.7 Hz, 1H), 6.83 (d, *J* = 8.7 Hz, 1H), 4.20 (q, *J* = 7.1 Hz, 2H), 4.02 (t, *J* = 6.5 Hz, 2H), 3.96 (t, *J* = 6.5 Hz, 2H), 3.84 (s, 3H), 1.88 (s, 3H), 1.85 – 1.78 (m, 2H), 1.75 – 1.68 (m, 2H), 1.55 – 1.50 (m, 2H), 1.46 – 1.40 (m, 2H), 1.23 (t, *J* = 7.1 Hz, 3H), 0.98 (t, *J* = 7.4 Hz, 3H), 0.93 (t, *J* = 7.4 Hz, 3H). ^{13}C NMR (126 MHz, Chloroform-*d*) δ 175.0, 158.6, 154.8, 153.3, 137.7, 136.2, 131.0,

130.7, 130.2, 129.93, 129.86, 127.40, 127.36, 122.3, 113.3, 112.5, 111.7, 68.8, 68.1, 61.4, 55.3, 55.1, 31.3, 31.2, 27.2, 19.3, 19.2, 14.1, 13.9, 13.8. HRMS (m/z, ESI): Calcd. for Chemical Formula: $C_{32}H_{39}ClNaO_5^+$ $[M+Na]^+$: 561.2378, Found: 561.2376. A sample of the ester was hydrolyzed to provide the parent carboxylic acid for the HPLC analysis of the reaction product: Daicel Chiralpak IA, hexane/*iso*-propanol = 99: 1, 1.0 mL/min, λ= 285 nm, retention time: 51.18 min (major) and 55.10 min (minor).

Ethyl (*S*)-2-(3-chloro-4-methylphenyl)-2-(4'-methoxy-6-methyl-[1,1'-biphenyl]-3-yl)-propanoate (32)

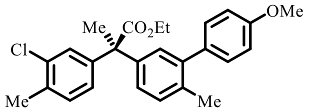

Colorless oil, 74 mg, 70%, $[\alpha]_D^{20}$ = -4.35 (*c* 1.000 $CHCl_3$) for 96.5:3.5 er. ^1H NMR (400 MHz, Chloroform-*d*) δ 7.26 (d, *J* = 1.8 Hz, 1H), 7.25 – 7.21 (m, 2H), 7.20 – 7.18 (m, 1H), 7.15 (dd, *J* = 8.1, 0.9 Hz, 1H), 7.10 – 7.03 (m, 3H), 6.96 – 6.91 (m, 2H), 4.21 (q, *J* = 7.1 Hz, 2H), 3.85 (s, 3H), 2.35 (s, 3H), 2.27 (s, 3H), 1.90 (s, 3H), 1.23 (t, *J* = 7.1 Hz, 3H). ^{13}C NMR (101 MHz, Chloroform-*d*) δ 174.7, 158.6, 143.9, 141.5, 141.2, 134.4, 134.3, 134.2, 134.0, 130.4, 130.3, 130.1, 129.3, 128.6, 126.6, 126.5, 113.5, 61.4, 55.7, 55.3, 27.0, 20.1, 19.6, 14.1. HRMS (m/z, ESI): Calcd. for Chemical Formula: $C_{26}H_{28}ClO_3^+$ $[M+H]^+$: 423.1721, Found: 423.1722. HPLC analysis of the reaction product: Daicel Chiralpak IA, hexane/*iso*-propanol = 99: 1, 1.0 mL/min, λ= 272 nm, retention time: 5.70 min (major) and 6.02 min (minor).

Ethyl (*S*)-2-(3-chloro-5-methylphenyl)-2-(4'-methoxy-5-methyl-[1,1'-biphenyl]-3-yl)-propanoate (33)

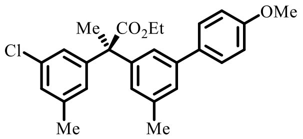

Colorless oil, 69 mg, 65%, $[\alpha]_D^{20}$ = -2.90 (*c* 1.000 $CHCl_3$) for 93.5:6.5 er. ^1H NMR (400 MHz, Chloroform-*d*) δ 7.48 – 7.43 (m, 2H), 7.28 – 7.27 (m, 1H), 7.19 (td, *J* = 1.8, 0.6 Hz, 1H), 7.08 – 7.60 (m, 2H), 6.98 – 6.92 (m, 4H), 4.24 (q, *J* = 7.1 Hz, 2H), 3.84 (s, 3H), 2.38 (s, 3H), 2.30 (s, 3H), 1.92 (s, 3H), 1.24 (t, *J* = 7.1 Hz, 3H). ^{13}C NMR (101 MHz, Chloroform-*d*) δ 174.6, 159.1, 146.4, 144.3, 140.6, 139.3, 138.1, 133.8, 133.6, 128.2, 127.7, 127.3, 127.0, 126.4, 125.5, 123.7, 114.1, 61.5, 56.4, 55.4, 27.1, 21.7, 21.4, 14.1. HRMS (m/z, ESI): Calcd. for Chemical Formula: $C_{26}H_{28}ClO_3^+$ $[M+H]^+$: 423.1721, Found: 423.1720. A sample of the ester was hydrolyzed to provide the parent carboxylic acid for the HPLC analysis of the reaction product: Daicel Chiralpak IA, hexane/*iso*-propanol = 99: 1, 1.0 mL/min, λ= 263 nm, retention time: 36.98 min (major) and 32.74 min (minor).

Ethyl (*S*)-2-(3-chloro-4-methoxy-5-methylphenyl)-2-(4',6-dimethoxy-5-methyl-[1,1'-biphenyl] -3-yl)-propanoate (34)

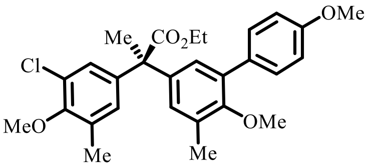

White solid, 90 mg, 75%, $[\alpha]_D^{20}$ = -23.1 (*c* 0.500 $CHCl_3$) for 98:2 er. ^1H NMR (400 MHz, Chloroform-*d*) δ 7.49 – 7.45 (m, 2H), 7.10 (d, *J* = 2.3 Hz, 1H), 7.00 – 6.91 (m, 5H), 4.23 (q, *J* = 7.1 Hz, 2H), 3.84 (s, 3H), 3.82 (s, 3H), 3.39 (s, 3H), 2.30 (s, 3H), 2.27 (s, 3H), 1.87 (s, 3H), 1.24 (t, *J* = 7.1 Hz, 3H). ^{13}C NMR (101 MHz, Chloroform-*d*) δ 174.8, 158.8, 154.7, 153.0, 141.0, 139.2, 133.7, 132.4, 131.13, 131.08, 130.2, 129.34, 129.32, 129.0, 128.4, 128.2, 127.7, 127.2, 113.7, 61.4, 60.1, 59.7, 55.5, 55.3, 27.2, 16.6, 14.1. HRMS (m/z, ESI): Calcd. for Chemical Formula:

$C_{28}H_{31}ClNaO_5^+$ $[M+Na]^+$: 505.1752, Found: 505.1749. A sample of the ester was hydrolyzed to provide the parent carboxylic acid for the HPLC analysis of the reaction product: Daicel Chiralpak IA, hexane/*iso*-propanol = 95: 5, 1.0 mL/min, λ= 272 nm, retention time: 8.99 min (major) and 8.03 min (minor).

Ethyl (*S*)-2-(3-chlorophenyl)-2-(4'-methoxy-[1,1'-biphenyl]-3-yl)-propanoate (35)

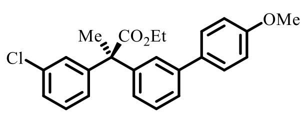

Colorless oil, 64 mg, 65%, $[\alpha]_D^{20}$ = -7.50 (*c* 1.000 $CHCl_3$) for 91.5:8.5 er. ^1H NMR (400 MHz, Chloroform-*d*) δ 7.49 – 7.45 (m, 3H), 7.41 (t, *J* = 1.7 Hz, 1H), 7.37 (td, *J* = 7.7, 0.5 Hz, 1H), 7.29 (dt, *J* = 1.9, 1.0 Hz, 1H), 7.26 – 7.23 (m, 2H), 7.18 – 7.13 (m, 2H), 7.00 – 6.93 (m, 2H), 4.24 (q, *J* = 7.1 Hz, 2H), 3.85 (s, 3H), 1.96 (s, 3H), 1.24 (t, *J* = 7.1 Hz, 3H). ^{13}C NMR (101 MHz, Chloroform-*d*) δ 174.4, 159.3, 146.6, 144.3, 140.8, 134.0, 133.6, 129.2, 128.6, 128.4, 128.2, 127.1, 126.6, 126.4, 126.3, 125.5, 114.2, 61.6, 56.5, 55.4, 27.1, 14.1. HRMS (m/z, ESI): Calcd. for Chemical Formula: $C_{24}H_{24}ClO_3^+$ $[M+H]^+$: 395.1408, Found: 395.1409. HPLC analysis of the reaction product: Daicel Chiralpak OJH, hexane/*iso*-propanol = 97: 3, 1.0 mL/min, λ= 263 nm, retention time: 17.44 min (major) and 11.84 min (minor).

Ethyl (*S*)-2-(5-chlorobenzo[b]thiophen-3-yl)-2-(5-(4-methoxyphenyl)-benzo[b]thiophen-3-yl)-propanoate (36)

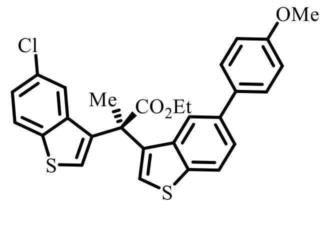

White solid, 82 mg, 65%, $[\alpha]_D^{20}$ = +12.85 (*c* 1.000 $CHCl_3$) for 97.5:2.5 er. ^1H NMR (400 MHz, Chloroform-*d*) δ 7.87 (dd, *J* = 8.3, 0.6 Hz, 1H), 7.75 (dd, *J* = 8.6, 0.5 Hz, 1H), 7.70 (dd, *J* = 1.7, 0.7 Hz, 1H), 7.62 (dd, *J* = 2.0, 0.5 Hz, 1H), 7.50 (dd, *J* = 8.4, 1.7 Hz, 1H), 7.41 – 7.38 (m, 1H), 7.37 – 7.32

(m, 2H), 7.30 (s, 1H), 7.26 – 7.22 (m, 1H), 6.96 – 6.90 (m, 2H), 4.25 – 4.17 (m, 2H), 3.83 (s, 3H), 2.22 (s, 3H), 1.12 (t, *J* = 7.1 Hz, 3H). ^{13}C NMR (101 MHz, Chloroform-*d*) δ 172.8, 159.0, 139.4, 139.0, 138.8, 137.9, 136.8, 136.7, 136.6, 133.8, 130.1, 128.3, 126.5, 125.1, 124.7, 124.3, 123.7, 123.6, 123.1, 121.9, 114.2, 61.9, 55.3, 51.7, 25.7, 14.0. HRMS (m/z, ESI): Calcd. for Chemical Formula: $C_{28}H_{23}ClNaO_3S_2^+$ $[M+Na]^+$: 529.0669, Found: 529.0672. HPLC analysis of the reaction product: Daicel Chiralpak IA, hexane/*iso*-propanol = 99: 1, 1.0 mL/min, λ= 257 nm, retention time: 14.06 min (major) and 12.79 min (minor).

The general procedure for desymmetrization of indole substrates

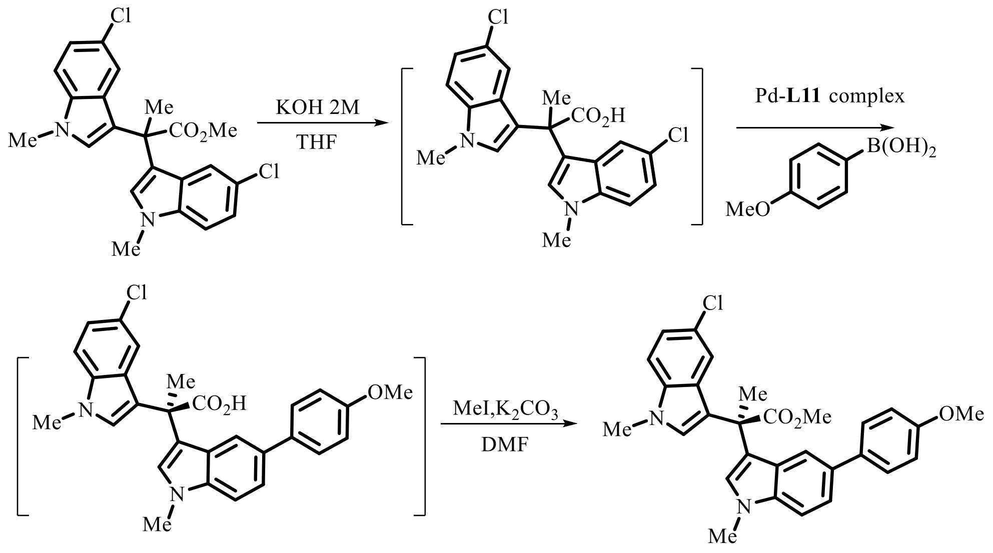

Under the N_2 atmosphere, methyl 2,2-bis(5-chloro-1-methyl-1H-indol-3-yl)-propanoate (103.5 mg, 0.25 mmol) was dissolved in 1.0 mL THF, and 2.0 M KOH $_{(aq.)}$ (0.65 mL). The resulting reaction mixture was heated to reflux and stirred overnight. After cooling to room temperature, the reaction mixture was concentrated and dried in a vacuum. The crude materials were gone to the next step without further purification. Under N_2 atmosphere, a mixture of $Pd_2(dba)_3$ (2.3 mg, 0.0025 mmol) and **L7** (3.2 mg, 0.005 mmol) was added to 0.5 mL 2-MeTHF, and the mixture was stirred at room

temperature for 20 min. The resulting metal-ligand complex solution was added to a reaction flask containing 4.5 mL 2-MeTHF solution of the above crude product mixture and 4-methoxyphenyl-boronic acid (45.6 mg, 0.3 mmol), and K_3PO_4 (265 mg, 1.25 mmol), followed by addition of 0.50 mL H_2O. Then the resulting reaction mixture was stirred at 60°C for 18 h. The reaction was then quenched with water, neutralized to pH 4 – 6 with saturated $KH_2PO_{4\,(aq.)}$, and extracted with ethyl acetate (4 mL X 3) three times. The combined organic phases were washed with brine, dried over Na_2SO_4, and concentrated in vacuo. After that, the obtained crude product and K_2CO_3 (138 mg, 1.0 mmol) were added to DMF (1.0 mL), then treated with MeI (71 mg, 0.5 mmol). The reaction mixture was stirred overnight at room temperature. Then the mixture was diluted with ethyl acetate (20 mL) and washed with water. The organic layer was concentrated in vacuo and purified by flash column chromatography with hexane and acetone as the eluents to give the desired product.

Methyl (*S*)-2-(5-chloro-1-methyl-1H-indol-3-yl)-2-(5-(4-methoxyphenyl)-1-methyl-1H-indol-3-yl)propanoate (37)

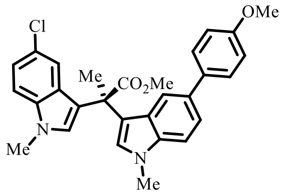

White solid, 88 mg, 72%, $[\alpha]_D^{20}$ = +9.05 (*c* 1.000 $CHCl_3$) for 97:3 er. ^{1}H NMR (400 MHz, Chloroform-*d*) δ 7.61 (dd, *J* = 1.7, 0.7 Hz, 1H), 7.51 (dd, *J* = 2.0, 0.6 Hz, 1H), 7.48 – 7.41 (m, 3H), 7.35 (dd, *J* = 8.5, 0.7 Hz, 1H), 7.21 (dd, *J* = 8.8, 0.6 Hz, 1H), 7.15 (dd, *J* = 8.7, 2.0 Hz, 1H), 6.96 – 6.92 (m, 2H), 6.90 (s, 1H), 6.82 (s, 1H), 3.84 (s, 3H), 3.75 (s, 3H), 3.71 – 3.70 (m, 6H), 2.11 (s, 3H). ^{13}C NMR (101 MHz, Chloroform-*d*) δ 175.7, 158.4, 136.9, 136.1, 135.3, 132.2, 128.9, 128.4, 128.1, 127.4, 126.8, 124.8, 121.8, 121.2, 120.8, 119.0, 117.6, 117.3, 114.1, 110.3, 109.6, 55.4, 52.4, 46.1, 33.0, 32.9, 26.2. HRMS (m/z, ESI): Calcd. for Chemical Formula: $C_{29}H_{27}ClN_2NaO_3^+$ $[M+Na]^+$: 509.1602, Found:

509.1603. HPLC analysis of the reaction product: Daicel Chiralpak IA, hexane/*iso*-propanol = 97: 3, 1.0 mL/min, λ= 257 nm, retention time: 21.86 min (major) and 20.67 min (minor).

Methyl (*S*)-2-(5-chloro-1-methyl-1H-indol-3-yl)-2-(5-(4-methoxyphenyl)-1-methyl-1H-indol-3-yl)-3-methylbutanoate (38)

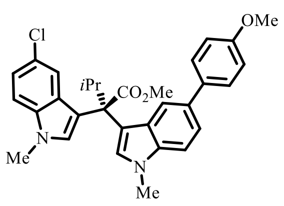

White solid, 35 mg, 27%, $[\alpha]_D^{20}$ = -1.50 (*c* 0.200 $CHCl_3$) for 92:8 er. ^1H NMR (400 MHz, Chloroform-*d*) δ 7.31 (dd, *J* = 2.9, 1.2 Hz, 2H), 7.28 (d, *J* = 2.1 Hz, 1H), 7.27 (d, *J* = 2.1 Hz, 1H), 7.23 (d, *J* = 1.4 Hz, 1H), 7.20 – 7.14 (m, 3H), 7.09 (s, 1H), 7.06 (dd, *J* = 8.6, 2.1 Hz, 1H), 6.92 – 6.87 (m, 2H), 3.82 (s, 3H), 3.81 (s, 3H), 3.77 (s, 3H), 3.63 (s, 3H), 3.29 (p, *J* = 6.6 Hz, 1H), 1.00 (d, *J* = 6.6 Hz, 3H), 0.96 (d, *J* = 6.6 Hz, 3H). ^{13}C NMR (101 MHz, Chloroform-*d*) δ 174.8, 158.3, 136.3, 135.5, 131.7, 130.7, 130.2, 128.8, 128.3, 128.2, 124.5, 121.6, 121.4, 120.7, 120.2, 114.0, 114.0, 113.5, 109.9, 109.2, 55.5, 55.3, 52.0, 33.7, 33.1, 33.0, 19.5, 19.4. HRMS (m/z, ESI): Calcd. for Chemical Formula: $C_{31}H_{31}ClN_2NaO_3^+$ $[M+Na]^+$: 537.1915, Found: 537.1914. HPLC analysis of the reaction product: Daicel Chiralpak IA, hexane/*iso*-propanol = 99: 1, 1.0 mL/min, λ= 255 nm, retention time: 34.15 min (major) and 32.17 min (minor).

Ethyl (*S*)-2-(5-chloro-1-ethyl-1H-indol-3-yl)-2-(1-ethyl-5-(4-methoxyphenyl)-1H-indol-3-yl)-4-oxopentanoate (39)

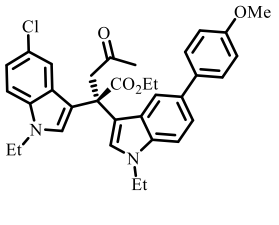

White solid, 83 mg, 58%, (Base was 2 M LiOH $_{(aq.)}$ (0.65 mL).). $[\alpha]_D^{20}$ = -4.05 (*c* 1.000 $CHCl_3$) for 93.5:6.5 er. ^1H NMR (400 MHz, Chloroform-*d*) δ 7.40 (s, 1H), 7.35 (t, *J* = 1.2 Hz, 1H), 7.31 – 7.25 (m, 5H), 7.24 (d, *J*

= 2.0 Hz, 1H), 7.17 (d, *J* = 8.7 Hz, 1H), 7.00 (dd, *J* = 8.7, 2.1 Hz, 1H), 6.89 – 6.85 (m, 2H), 4.21 – 4.10 (m, 6H), 3.88 – 3.78 (m, 5H), 1.91 (s, 3H), 1.48 – 1.43 (m, 6H), 1.17 (t, *J* = 7.1 Hz, 3H). ^{13}C NMR (101 MHz, Chloroform-*d*) δ 205.9, 173.0, 158.3, 135.5, 135.3, 134.6, 131.7, 128.7, 128.1, 128.0, 127.8, 127.0, 124.3, 121.3, 120.7, 120.6, 119.0, 114.3, 114.2, 113.9, 110.2, 109.5, 61.1, 55.3, 50.6, 47.9, 41.2, 41.2, 30.8, 15.7, 15.6, 14.1. HRMS (m/z, ESI): Calcd. for Chemical Formula: $C_{34}H_{35}ClN_2NaO_4^+$ [M+Na]$^+$: 593.2178 Found: 593.2177. HPLC analysis of the reaction product: Daicel Chiralpak IA, hexane/*iso*-propanol = 95: 5, 1.0 mL/min, λ= 259 nm, retention time: 20.29 min (major) and 26.39 min (minor).

Ethyl (*R*)-2-(3-chlorophenyl)-2-methoxy-2-(4'-methoxy-[1,1'-biphenyl]-3-yl)-acetate (40)

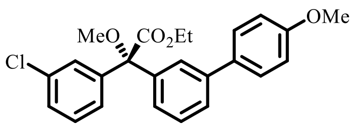

White solid, 63 mg, 61%, $[\alpha]_D^{20}$ = -12.15 (*c* 1.000 $CHCl_3$) for 86:14 er. ^{1}H NMR (400 MHz, Chloroform-*d*) δ 7.62 (t, *J* = 1.8 Hz, 1H), 7.57 (t, *J* = 1.9 Hz, 1H), 7.54 – 7.47 (m, 3H), 7.42 – 7.27 (m, 5H), 6.99 – 6.94 (m, 2H), 4.28 (q, *J* = 7.1 Hz, 2H), 3.84 (s, 3H), 3.23 (s, 3H), 1.24 (d, *J* = 7.1 Hz, 3H). ^{13}C NMR (101 MHz, Chloroform-*d*) δ 171.1, 159.3, 142.7, 140.6, 140.2, 134.0, 133.3, 129.2, 128.5, 128.4, 128.23, 128.19, 126.9, 126.8, 126.7, 126.6, 114.2, 86.7, 61.8, 55.4, 53.4, 14.1. HRMS (m/z, ESI): Calcd. for Chemical Formula: $C_{24}H_{23}ClNaO_4^+$ [M+Na]$^+$: 433.1177, Found: 433.1180. HPLC analysis of the reaction product: Daicel Chiralpak OJH, hexane/*iso*-propanol = 99: 1, 1 mL/min, λ= 263 nm, retention time: 23.12 min (major) and 29.63 min (minor).

Ethyl (*R*)-2-(3-chloro-4-methylphenyl)-2-methoxy-2-(4'-methoxy-6-methyl-[1,1'-biphenyl]-3-yl)-acetate (41)

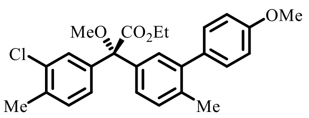

White solid, 58 mg, 53%, $[\alpha]_D^{20}$ = -10.05 (*c* 1.000 $CHCl_3$) for 94:6 er. ^1H NMR (400 MHz, Chloroform-*d*) δ 7.52 (d, *J* = 1.9 Hz, 1H), 7.28 – 7.19 (m, 6H), 7.16 (dd, *J* = 8.1, 0.8 Hz, 1H), 6.96 – 6.90 (m, 2H), 4.25 (q, *J* = 7.1 Hz, 2H), 3.84 (s, 3H), 3.18 (s, 3H), 2.36 (s, 3H), 2.28 (s, 3H), 1.23 (t, *J* = 7.1 Hz, 3H). ^{13}C NMR (101 MHz, Chloroform-*d*) δ 171.4, 158.6, 141.1, 139.9, 137.2, 135.7, 135.5, 134.0, 133.9, 130.3, 130.0, 128.9, 127.1, 126.8, 113.5, 86.4, 61.7, 55.3, 53.2, 20.3, 19.8, 14.1. HRMS (m/z, ESI): Calcd. for Chemical Formula: $C_{26}H_{27}ClNaO_4^+$ $[M+Na]^+$: 461.1490, Found: 461.1487. HPLC analysis of the reaction product: Daicel Chiralpak IA, hexane/*iso*-propanol = 99: 1, 0.5 mL/min, λ= 274 nm, retention time: 13.08 min (major) and 14.51 min (minor).

Ethyl (*R*)-2-(3-chloro-4-methoxyphenyl)-2-(4',6-dimethoxy-[1,1'-biphenyl]-3-yl)-2-methoxy acetate (42)

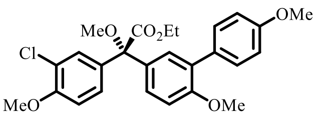

White solid, 70 mg, 60%, $[\alpha]_D^{20}$ = -5.25 (*c* 1.000 $CHCl_3$) for 94:6 er. ^1H NMR (400 MHz, Chloroform-*d*) δ 7.54 (d, *J* = 2.3 Hz, 1H), 7.45 – 7.41 (m, 2H), 7.35 (dd, *J* = 8.7, 2.3 Hz, 1H), 7.33 – 7.30 (m, 2H), 6.95 – 6.90 (m, 3H), 6.87 (d, *J* = 8.7 Hz, 1H), 4.25 (q, *J* = 7.1 Hz, 2H), 3.90 (s, 3H), 3.830 – 3.826 (m, 6H), 3.18 (s, 3H), 1.24 (t, *J* = 7.1 Hz, 3H). ^{13}C NMR (101 MHz, Chloroform-*d*) δ 171.6, 158.8, 156.2, 154.5, 133.9, 132.0, 131.0, 130.7, 130.5, 130.3, 129.7, 128.5, 128.0, 121.9, 113.5, 111.2, 110.4, 86.1, 61.7, 56.1, 55.6, 55.3, 53.1, 14.1. HRMS (m/z, ESI): Calcd. for Chemical Formula: $C_{26}H_{27}ClNaO_6^+$ $[M+Na]^+$: 493.1388, Found: 493.1385. HPLC analysis of the reaction product: Daicel Chiralpak ID, hexane/*iso*-propanol

= 99: 1, 0.5 mL/min, λ= 272 nm, retention time: 43.66 min (major) and 47.94 min (minor).

Ethyl (*R*)-2-acetamido-2-(3-chloro-4-methoxyphenyl)-2-(4',6-dimethoxy-[1,1'-biphenyl]-3-yl) acetate (43)

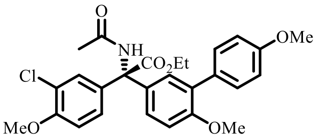

White solid, 70 mg, 56%, $[\alpha]_D^{20}$ = -5.10 (*c* 1.000 $CHCl_3$) for 96:4 er. ^{1}H NMR (500 MHz, Chloroform-*d*) δ 7.41 (d, *J* = 8.4 Hz, 3H), 7.35 – 7.25 (m, 3H), 7.11 (s, 1H), 6.95 – 6.84 (m, 4H), 4.24 (q, *J* = 7.1 Hz, 2H), 3.89 (s, 3H), 3.83 (s, 3H), 3.81 (s, 3H), 2.05 (s, 3H), 1.20 (t, *J* = 7.1 Hz, 3H). ^{13}C NMR (126 MHz, Chloroform-*d*) δ 171.9, 168.5, 158.8, 156.1, 154.3, 132.2, 130.8, 130.7, 130.62, 130.55, 130.5, 129.7, 129.0, 128.3, 126.8, 121.7, 113.5, 112.1, 111.0, 110.4, 68.6, 62.8, 56.1, 55.6, 55.3, 24.0, 13.9. HRMS (m/z, ESI): Calcd. for Chemical Formula: $C_{27}H_{29}ClNO_6^+$ $[M+H]^+$: 498.1678, Found: 498.1673. HPLC analysis of the reaction product: Daicel Chiralpak IA, hexane/*iso*-propanol = 95: 5, 1.0 mL/min, λ= 272 nm, retention time: 40.74 min (major) and 31.11 min (minor).

Ethyl (*R*)-2-(3-chloro-4-methylphenyl)-2-fluoro-2-(4'-methoxy-6-methyl-[1,1'-biphenyl]-3-yl) acetate (44)

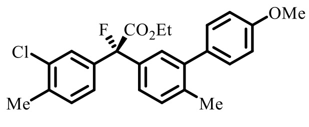

White solid, 21 mg, 20%, $[\alpha]_D^{20}$ = -1.43 (*c* 0.350 $CHCl_3$) for 91.5:8.5 er. ^{1}H NMR (500 MHz, Chloroform-*d*) δ 7.48 (d, *J* = 1.8 Hz, 1H), 7.26 (s, 4H), 7.24 – 7.21 (m, 3H), 6.96 – 6.92 (m, 2H), 4.33 (q, *J* = 7.1 Hz, 2H), 3.85 (s, 3H), 2.38 (s, 3H), 2.29 (s, 3H), 1.30 (t, *J* = 7.1 Hz, 3H). ^{19}F NMR (471 MHz, Chloroform-*d*) δ -140.13 (s, 1F). ^{13}C NMR (126 MHz, Chloroform-*d*) δ 169.4 (d, *J* = 27.8 Hz), 158.7, 141.6, 137.8, 137.6, 136.9 (d, *J* = 6.8 Hz), 135.7, 135.5, 134.4, 133.7, 130.7, 130.4, 130.3, 128.3 (d, *J* = 6.6 Hz), 127.5 (d, *J* = 7.7

Hz), 125.5 (d, J = 6.3 Hz), 125.2 (d, J = 6.7 Hz), 113.6, 96.7 (d, J = 190.7 Hz), 62.5, 55.3, 20.4, 19.8, 14.1. HRMS (m/z, ESI): Calcd. for Chemical Formula: $C_{25}H_{24}ClFNaO_3^+$ $[M+Na]^+$: 449.1290, Found: 449.1291. HPLC analysis of the reaction product: Daicel Chiralpak IA, hexane/*iso*-propanol = 99: 1, 1.0 mL/min, λ= 272 nm, retention time: 7.40 min (major) and 8.41 min (minor).

Ethyl (*S*)-2-(3-chloro-4-methoxyphenyl)-2-(4',6-dimethoxy-[1,1'-biphenyl]-3-yl)acetate (45)

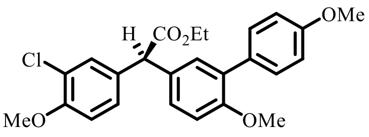

Colorless oil, 55 mg, 50%, $[\alpha]_D^{20}$ = -7.90 (*c* 1.000 $CHCl_3$) for 93:7 er. ^{1}H NMR (400 MHz, Chloroform-*d*) δ 7.47 – 7.42 (m, 2H), 7.36 (d, J = 2.3 Hz, 1H), 7.24 – 7.19 (m, 3H), 6.96 – 6.91 (m, 3H), 6.88 (d, J = 8.6 Hz, 1H), 4.90 (s, 1H), 4.21 (q, J = 7.1 Hz, 2H), 3.88 (s, 3H), 3.84 (s, 3H), 3.80 (s, 3H), 1.28 (t, J = 7.1 Hz, 3H). ^{13}C NMR (101 MHz, Chloroform-*d*) δ 172.5, 158.8, 155.8, 154.1, 132.3, 130.8, 130.7, 130.6, 130.53, 130.47, 130.4, 127.9, 127.8, 122.4, 113.5, 112.0, 111.3, 61.3, 56.2, 55.6, 55.3, 55.2, 14.2. HRMS (m/z, ESI): Calcd. for Chemical Formula: $C_{25}H_{26}ClO_5^+$ $[M+Na]^+$: 441.1463, Found: 441.1464. HPLC analysis of the reaction product: Daicel Chiralpak ID, hexane/*iso*-propanol = 99: 1, 1.0 mL/min, λ= 258 nm, retention time: 23.92 min (major) and 26.65 min (minor).

(*S*)-2-(4'-(tert-butyl)-6-methoxy-[1,1'-biphenyl]-3-yl)-2-(3-chloro-4-methoxyphenyl)-acetic acid (46)

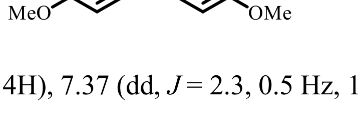

White solid, 50 mg, 46%. $[\alpha]_D^{20}$ = -3.90 (*c* 0.500 $CHCl_3$) for 91.5:8.5 er. ^{1}H NMR (400 MHz, Chloroform-*d*) δ 7.46 – 7.40 (m, 4H), 7.37 (dd, J = 2.3, 0.5 Hz, 1H), 7.26 – 7.20 (m, 3H), 6.94 (dd, J = 8.1, 0.8 Hz, 1H), 6.88 (d, J = 8.5 Hz, 1H), 4.94 (s, 1H), 3.88 (s, 3H), 3.80 (s, 3H), 1.35 (s, 9H). ^{13}C NMR (101 MHz, Chloroform-

d) δ 177.2, 156.0, 154.3, 150.0, 135.0, 131.4, 131.1, 130.8, 130.4, 129.9, 129.1, 128.2, 127.9, 125.0, 122.6, 112.1, 111.3, 56.2, 55.6, 55.0, 34.5, 31.4. HRMS (m/z, ESI): Calcd. for Chemical Formula: $C_{26}H_{27}ClNaO_4^+$ $[M+Na]^+$: 461.1490, Found: 461.1491. HPLC analysis of the reaction product: Daicel Chiralpak IA, hexane/*iso*-propanol = 90: 10, 1.0 mL/min, λ= 286 nm, retention time: 8.18 min (major) and 6.86 min (minor).

Ethyl (*S*)-5'-(1-(3-chloro-4-methoxyphenyl)-2-ethoxy-2-oxoethyl)-2'-methoxy-[1,1'-biphenyl]-4-carboxylate (47)

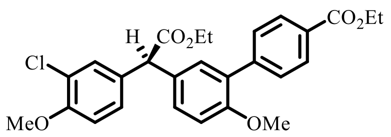

White solid, 66 mg, 55%. $[\alpha]_D^{20}$ = +5.60 (*c* 0.500 $CHCl_3$) for 89.5:10.5 er. ^1H NMR (500 MHz, Chloroform-*d*) δ 8.10 – 8.04 (m, 2H), 7.61 – 7.53 (m, 2H), 7.35 (d, *J* = 2.3 Hz, 1H), 7.29 (dd, *J* = 8.5, 2.4 Hz, 1H), 7.25 (d, *J* = 2.4 Hz, 1H), 7.20 (dd, *J* = 8.5, 2.3 Hz, 1H), 6.96 (d, *J* = 8.5 Hz, 1H), 6.88 (d, *J* = 8.5 Hz, 1H), 4.91 (s, 1H), 4.39 (q, *J* = 7.1 Hz, 2H), 4.24 – 4.20 (m, 2H), 3.88 (s, 3H), 3.80 (s, 3H), 1.40 (t, *J* = 7.1 Hz, 3H), 1.27 (t, *J* = 7.1 Hz, 3H). ^{13}C NMR (126 MHz, Chloroform-*d*) δ 172.4, 166.6, 155.8, 154.2, 142.9, 132.1, 130.9, 130.9, 130.3, 129.8, 129.5, 129.24, 129.19, 129.0, 127.7, 122.5, 112.1, 111.5, 61.4, 60.9, 56.2, 55.7, 55.1, 14.4, 14.2. HRMS (m/z, ESI): Calcd. for Chemical Formula: $C_{27}H_{28}ClO_6^+$ $[M+H]^+$: 483.1569, Found: 483.1563. HPLC analysis of the reaction product: Daicel Chiralpak IA, hexane/*iso*-propanol = 99: 1, 1.0 mL/min, λ= 267 nm, retention time: 36.78 min (major) and 46.19 min (minor).

Ethyl (S)-2-(3-bromo-4-methoxyphenyl)-2-(4',6-dimethoxy-[1,1'-biphenyl]-3-yl)propanoate (48)

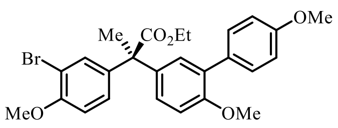

White solid, 40 mg, 32%. $[\alpha]_D^{20}$ = -2.40 (*c* 1.000 $CHCl_3$) for 62.5:37.5 er. ^1H NMR (400 MHz, Chloroform-*d*) δ 7.47 (d, *J* = 2.4 Hz, 1H), 7.44 – 7.39 (m, 2H), 7.18 – 7.08 (m, 3H), 6.95 – 6.87 (m, 3H), 6.81 (s, 1H), 4.21 (q, *J* = 7.1 Hz, 2H), 3.89 (s, 3H), 3.83 (s, 3H), 3.81 (s, 3H), 1.89 (s, 3H), 1.23 (t, *J* = 7.1 Hz, 3H). ^{13}C NMR (101 MHz, Chloroform-*d*) δ 174.9, 158.7, 155.3, 154.6, 138.4, 136.4, 133.0, 130.8, 130.6, 130.3, 129.8, 128.3, 127.5, 113.5, 111.2, 111.1, 110.7, 61.4, 56.2, 55.6, 55.3, 55.0, 27.2, 14.1. HRMS (m/z, ESI): Calcd. for Chemical Formula: $C_{26}H_{28}BrO_5^+$ $[M+H]^+$: 499.1115, Found: 499.1123. HPLC analysis of the reaction product: Daicel Chiralpak IA, hexane/*iso*-propanol = 99: 1, 1.0 mL/min, λ= 272 nm, retention time: 18.22 min (major) and 20.02 min (minor).

Ethyl (S)-2-(4-chlorophenyl)-2-(4',methoxy-[1,1'-biphenyl]-4-yl)propanoate (49)

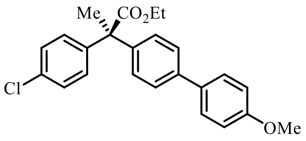

White solid, 60 mg, 61%. $[\alpha]_D^{20}$ = +1.13 (*c* 0.580 $CHCl_3$) for 56.5:43.5 er. ^1H NMR (400 MHz, Chloroform-*d*) δ 7.55 – 7.47 (m, 4H), 7.30 – 7.23 (m, 4H), 7.23 – 7.17 (m, 2H), 7.00 – 6.94 (m, 2H), 4.22 (q, *J* = 7.1 Hz, 2H), 3.85 (s, 3H), 1.93 (s, 3H), 1.23 (t, *J* = 7.1 Hz, 3H). ^{13}C NMR (101 MHz, Chloroform-*d*) δ 174.7, 159.2, 143.1, 142.5, 139.4, 133.0, 132.7, 129.6, 128.3, 128.2, 128.1, 126.4, 114.2, 61.5, 55.9, 55.4, 27.0, 14.0. HRMS (m/z, ESI): Calcd. for Chemical Formula: $C_{24}H_{24}ClO_3^+$ $[M+H]^+$: 395.1408, Found: 395.1412. HPLC analysis of the reaction product: Daicel Chiralpak OJH, hexane/*iso*-propanol = 97: 3, 1.0 mL/min, λ= 267 nm, retention time: 41.65 min (major) and 56.51 min (minor).

Ethyl (*S*)-2-(4-chlorophenyl)-2-(4'-methoxy-[1,1'-biphenyl]-4-yl)-acetate(50)

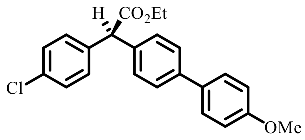

White solid, 35 mg, 37%, $[\alpha]_D^{20}$ = +3.40 (*c* 1.000 $CHCl_3$) for 65.5:34.5 er. ^1H NMR (400 MHz, Chloroform-*d*) δ 7.54 – 7.47 (m, 4H), 7.36 – 7.27 (m, 6H), 6.97 (d, *J* = 8.8 Hz, 2H), 5.00 (s, 1H), 4.23 (q, *J* = 7.1 Hz, 2H), 3.85 (s, 3H), 1.28 (t, *J* = 7.1 Hz, 3H). ^{13}C NMR (101 MHz, Chloroform-*d*) δ 172.2, 159.2, 140.0, 137.3, 136.7, 133.2, 133.1, 130.0, 128.84, 128.77, 128.1, 127.0, 114.2, 61.4, 56.1, 55.4, 14.2. HRMS (m/z, ESI): Calcd. for Chemical Formula: $C_{23}H_{21}ClNaO_3^+$ $[M+Na]^+$: 403.1071, Found: 403.1069. HPLC analysis of the reaction product: Daicel Chiralpak OJH, hexane/*iso*-propanol = 97: 3, 1.0 mL/min, λ= 266 nm, retention time: 45.29 min (major) and 51.40 min (minor).

Ethyl-2-(2-chlorophenyl)-2-(4'-methoxy-[1,1'-biphenyl]-4-yl)propanoate (rac.51)

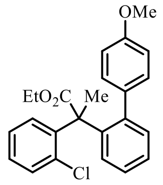

^1H NMR (400 MHz, Chloroform-*d*) δ 7.56 – 7.47 (m, 4H), 7.31 – 7.24 (m, 4H), 7.23 – 7.19 (m, 2H), 7.00 – 6.95 (m, 2H), 4.23 (q, *J* = 7.1 Hz, 2H), 3.85 (s, 3H), 1.93 (s, 3H), 1.24 (t, *J* = 7.1 Hz, 3H). ^{13}C NMR (101 MHz, Chloroform-*d*) δ 174.7, 159.2, 143.1, 142.5, 139.4, 133.0, 132.7, 129.6, 128.3, 128.2, 128.1, 126.4, 114.2, 61.5, 55.9, 55.4, 27.0, 14.0. HRMS (m/z, ESI): Calcd. for Chemical Formula: $C_{24}H_{24}ClO_3^+$ $[M+H]^+$: 395.1408 Found: 395.1410.

The NMR and HRMS data were obtained from racemic reaction between Et-ester of the bis-chloro substrate and aryl boronic acid using SPhos as ligand. Formation of coupling product was not observed using **L11** as the chiral ligand.

Ethyl (S)-2-(2-chlorophenyl)-2-(4'-methoxy-[1,1'-biphenyl]-2-yl)acetate (52)

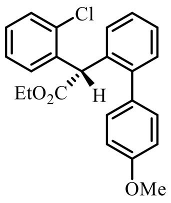

<5% yield, 69.5:30.5 er. ^{1}H NMR (500 MHz, Chloroform-*d*) δ 7.36 – 7.31 (m, 3H), 7.29 – 7.27 (m, 2H), 7.22 – 7.19 (m, 2H), 7.17 – 7.14 (m, 1H), 7.09 – 7.06 (m, 2H), 6.90 – 6.86 (m, 2H), 5.45 (s, 1H), 4.18 – 4.12 (m, 2H), 3.83 (s, 3H), 1.19 (t, *J* = 7.1 Hz, 3H). ^{13}C NMR (126 MHz, Chloroform-*d*) δ 172.4, 158.9, 142.6, 137.0, 134.9, 134.4, 133.2, 130.8, 130.2, 130.1, 129.6, 128.8, 128.4, 127.42, 127.36, 126.7, 113.6, 61.3, 55.3, 51.6, 14.1. HRMS (m/z, ESI): Calcd. for Chemical Formula: $C_{23}H_{21}ClNaO_3^+$ $[M+Na]^+$: 403.1071, Found: 403.1067. HPLC analysis of the reaction product: Daicel Chiralpak IA, hexane/*iso*-propanol = 99: 1, 1.0 mL/min, λ= 245 nm, retention time: 12.34 min (major) and 7.89 min (minor).

(*S*)-4-(9-butyl-2-chloro-7-(9-phenyl-9H-carbazol-3-yl)-9H-xanthen-9-yl)-butanoic acid (1)

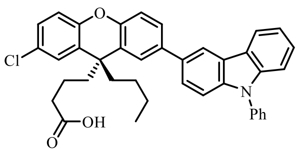

White solid, 45 mg, 30% yield, $[\alpha]_D^{20}$ = +1.25 (*c* 1.830 $CHCl_3$) for 86:14 er. NMR was same with (*R*)-4-(9-butyl-2-chloro-7-(9-phenyl-9H-carbazol-3-yl)-9H-xanthen-9-yl)-butanoic acid (**1**). A sample of the acid was esterification to provide the Me-ester for the HPLC analysis of the reaction product: Daicel Chiralpak IA, hexane/*iso*-propanol = 99: 1, 1.0 mL/min, λ= 295 nm, retention time: 12.89 min (major) and 9.91 min (minor).

(*S*)-4-(2-chloro-7-(4-methoxyphenyl)-9H-xanthen-9-yl)-butanoic acid (2)

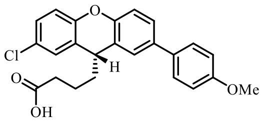

White solid, 53 mg, 52% yield, $[\alpha]_D^{20}$ = -14.5 (*c* 1.000 $CHCl_3$) for 93.5:6.5 er. NMR was same with (*R*)-4-(2-chloro-7-(4-methoxyphenyl)-9H-xanthen-9-yl)-butanoic acid (**2**). HPLC analysis of the reaction product: Daicel Chiralpak ID,

hexane/*iso*-propanol = 95: 5, 1.0 mL/min, λ= 272 nm, retention time: 11.51 min (major) and 14.02 min (minor).

The general procedure of Sonogashira cross-coupling desymmetrization reaction.

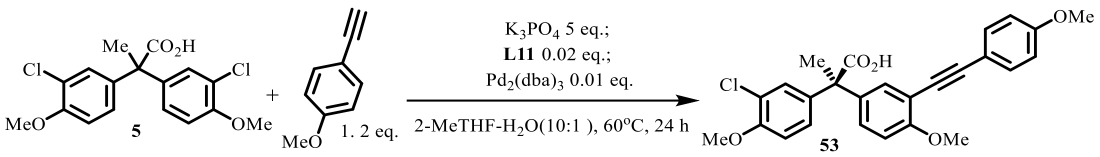

Under N_2 atmosphere, a mixture of $Pd_2(dba)_3$ (2.3 mg, 0.0025 mmol) and **L11**(3.2 mg, 0.005 mmol) was added to 0.5 mL 2-MeTHF, the mixture was then stirred at room temperature for 20 min. The resulting metal-ligand complex solution was added to a reaction flask containing 4.5 mL 2-MeTHF solution of 2,2-bis(3-chloro-4-methoxyphenyl)-propanoic acid **5** (88.5 mg, 0.25 mmol) and 4-1-Ethynyl-4-methoxybenzene (39.6 mg, 0.3 mmol), and K_3PO_4 (265 mg, 1.25 mmol), followed by addition of 0.50 mL H_2O. Then the resulting reaction mixture was stirred at 60°C for 24 h. The reaction was then quenched with water, neutralized to pH 3 – 5 with 1 M HCl $_{(aq.)}$, and extracted with ethyl acetate (4 mL X 3) three times. The combined organic phase was washed with brine and dried over Na_2SO_4. The organic layer was concentrated in vacuo and purified by flash column chromatography with hexane and acetone as the eluents to give the desired products.

The general procedure for esterification of products for isolation and characterization:

The obtained crude product was dissolved in DMF (1.0 mL), then treated with EtI (78 mg, 0.5 mmol) and K_2CO_3 (138 mg, 1.0 mmol). The reaction mixture was stirred overnight at room temperature. Then the mixture was diluted with ethyl acetate (20 mL) and washed with water. The organic layer was concentrated in vacuo and purified by flash column chromatography with hexane and acetone as the eluents to give the desired product.

Characterization data of desymmetrization products of Sonogashira cross-coupling reaction

(*S*)-2-(3-chloro-4-methoxyphenyl)-2-(4-methoxy-3-((4-methoxyphenyl)-ethynyl)phenyl)propa-noic acid (53)

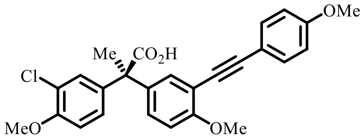

White solid, 79 mg, 70%, $[\alpha]_D^{20}$ = +13.35 (*c* 1.000 $CHCl_3$) for 98.5:1.5 er. ^{1}H NMR (500 MHz, Chloroform-*d*) δ 7.50 – 7.45 (m, 2H), 7.40 (d, *J* = 2.6 Hz, 1H), 7.29 (d, *J* = 2.4 Hz, 1H), 7.18 (dd, *J* = 8.8, 2.6 Hz, 1H), 7.12 (dd, *J* = 8.7, 2.4 Hz, 1H), 6.89 – 6.81 (m, 4H), 3.90 (s, 3H), 3.89 (s, 3H), 3.81 (s, 3H), 1.90 (s, 3H). ^{13}C NMR (126 MHz, Chloroform-*d*) δ 180.6, 159.6, 158.9, 154.0, 137.0, 135.3, 133.2, 132.7, 129.8, 129.2, 127.6, 122.1, 115.5, 113.9, 112.6, 111.6, 110.5, 93.7, 84.2, 56.2, 56.0, 55.3, 54.8, 26.9. HRMS (m/z, ESI): Calcd. for Chemical Formula: $C_{26}H_{24}ClO_5^+$ $[M+H]^+$: 451.1307, Found: 451.1304. HPLC analysis of the reaction product: Daicel Chiralpak IA, hexane/*iso*-propanol = 80: 20, 1.0 mL/min, λ= 312 nm, retention time: 8.27 min (major) and 10.31 min (minor).

(*S*)-2-(3-(benzo[d][1,3]-dioxol-5-ylethynyl)-4-methoxyphenyl)-2-(3-chloro-4-methoxyphenyl)-propanoic acid (54)

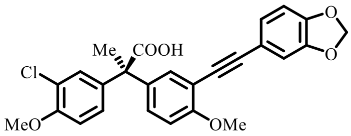

White solid, 53 mg, 46%, $[\alpha]_D^{20}$ = +17.20 (*c* 0.500 $CHCl_3$) for 96:4 er. ^{1}H NMR (500 MHz, Chloroform-*d*) δ 7.38 (d, *J* = 2.5 Hz, 1H), 7.28 (d, *J* = 2.4 Hz, 1H), 7.18 (dd, *J* = 8.7, 2.6 Hz, 1H), 7.12 (dd, *J* = 8.7, 2.4 Hz, 1H), 7.07 (dd, *J* = 8.0, 1.6 Hz, 1H), 6.99 (d, *J* = 1.6 Hz, 1H), 6.85 (dd, *J* = 13.8, 8.8 Hz, 2H), 6.77 (d, *J* = 8.0 Hz, 1H), 5.97 (s, 2H), 3.90 – 3.89 (m, 6H), 1.89 (s, 3H). ^{13}C NMR (126 MHz, Chloroform-*d*) δ

180.1, 158.9, 154.0, 147.9, 147.4, 136.9, 135.3, 132.7, 129.8, 129.3, 127.5, 126.4, 122.1, 116.7, 112.4, 111.7, 111.6, 110.5, 108.4, 101.3, 93.6, 84.0, 56.2, 56.0, 54.8, 26.9. HRMS (m/z, ESI): Calcd. for Chemical Formula: $C_{26}H_{22}ClNO_6^+$ $[M+H]^+$: 465.1099, Found: 465.1097. HPLC analysis of the reaction product: Daicel Chiralpak IA, hexane/*iso*-propanol = 80: 20, 1.0 mL/min, λ= 316 nm, retention time: 9.45 min (major) and 14.67 min (minor).

Ethyl (*S*)-2-(3-chloro-4-methoxyphenyl)-2-(4-methoxy-3-(p-tolylethynyl)-phenyl)-propanoate (55)

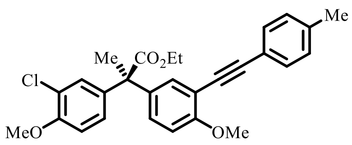

White solid, 61 mg, 53%, $[\alpha]_D^{20}$ = +3.55 (*c* 1.000 $CHCl_3$) for 95.5:4.5 er. ^{1}H NMR (500 MHz, Chloroform-*d*) δ 7.44 (d, *J* = 7.9 Hz, 2H), 7.38 – 7.35 (m, 1H), 7.25 (dd, *J* = 2.4, 0.8 Hz, 1H), 7.17 – 7.11 (m, 3H), 7.08 – 7.06 (m, 1H), 6.84 (dd, *J* = 12.6, 8.7 Hz, 2H), 4.21 (q, *J* = 7.1 Hz, 2H), 3.91 – 3.89 (m, 6H), 2.35 (s, 3H), 1.87 (s, 3H), 1.23 (t, *J* = 7.1 Hz, 3H). ^{13}C NMR (126 MHz, Chloroform-*d*) δ 174.7, 158.8, 153.7, 138.3, 137.8, 136.1, 132.8, 131.6, 129.9, 129.3, 129.0, 127.4, 122.0, 120.3, 112.3, 111.5, 110.4, 93.7, 85.0, 61.5, 56.1, 56.0, 54.9, 27.1, 21.5, 14.1. HRMS (m/z, ESI): Calcd. for Chemical Formula: $C_{28}H_{28}ClO_4^+$ $[M+H]^+$: 463.1671, Found: 463.1669. The ester product was provided for the HPLC analysis of the reaction product: Daicel Chiralpak IA, hexane/*iso*-propanol = 99: 1, 1.0 mL/min, λ= 310 nm, retention time: 33.07 min (major) and 31.38 min (minor).

(*S*)-2-(3-chloro-4-methoxyphenyl)-2-(3-((4-(dimethylamino)-phenyl)-ethynyl)-4-methoxyphenyl)-propanoic acid (56)

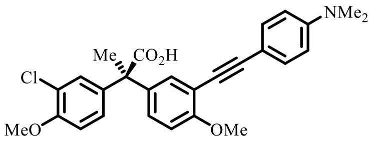

Red solid, 75 mg, 65%, $[\alpha]_D^{20}$ = +1.08 (*c* 1.000 $CHCl_3$) for 98.5:1.5 er. ^1H NMR (500 MHz, Chloroform-*d*) δ 7.47 – 7.38 (m, 3H), 7.28 (d, *J* = 2.4 Hz, 1H), 7.16 – 7.10 (m, 2H), 6.90 – 6.80 (m, 2H), 6.69 – 6.60 (m, 2H), 3.90 – 3.89 (m, 6H), 2.98 (s, 6H), 1.89 (s, 3H). ^{13}C NMR (126 MHz, Chloroform-*d*) δ 179.6, 158.7, 153.9, 150.1, 137.2, 135.3, 132.8, 132.5, 129.9, 128.7, 127.6, 113.1, 111.8, 111.6, 110.4, 110.3, 94.9, 83.5, 56.2, 56.0, 54.8, 40.3, 27.0. HRMS (m/z, ESI): Calcd. for Chemical Formula: $C_{27}H_{27}ClNO_4^+$ $[M+H]^+$: 464.1623, Found: 464.1621. HPLC analysis of the reaction product: Daicel Chiralpak IA, hexane/*iso*-propanol = 80: 20, 1.0 mL/min, λ= 327 nm, retention time: 8.47 min (major) and 10.71 min (minor).

Ethyl (*S*)-2-(3-chloro-4-methoxyphenyl)-2-(4-methoxy-3-((4-phenoxyphenyl)-ethynyl)-phenyl) -propanoate (57)

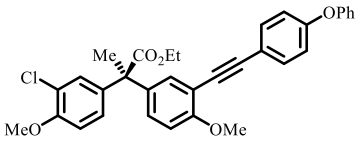

White solid, 82 mg, 61%, $[\alpha]_D^{20}$ = +13.50 (*c* 1.000 $CHCl_3$) for 96.5:3.5 er. ^1H NMR (400 MHz, Chloroform-*d*) δ 7.53 – 7.49 (m, 2H), 7.38 – 7.33 (m, 3H), 7.25 (s, 1H), 7.16 – 7.11 (m, 2H), 7.08 (dd, *J* = 8.6, 2.4 Hz, 1H), 7.05 – 7.01 (m, 2H), 6.97 – 6.93 (m, 2H), 6.85 (t, *J* = 9.0 Hz, 2H), 4.22 (q, *J* = 7.1 Hz, 2H), 3.91 (s, 3H), 3.89 (s, 3H), 1.88 (s, 3H), 1.23 (t, *J* = 7.1 Hz, 3H). ^{13}C NMR (101 MHz, Chloroform-*d*) δ 174.7, 158.8, 157.5, 156.5, 153.8, 137.7, 136.1, 133.3, 132.8, 129.9, 129.3, 127.4, 123.8, 122.0, 119.4, 118.4, 118.1, 112.2, 111.5, 110.4, 93.0, 85.1, 61.5, 56.2, 56.0, 54.9, 27.1, 14.1. HRMS (m/z, ESI): Calcd. for Chemical Formula: $C_{33}H_{33}ClO_5^+$ $[M+H]^+$: 541.1776, Found: 541.1777. HPLC analysis

of the reaction product: Daicel Chiralpak IA, hexane/*iso*-propanol = 99: 1, 1.0 mL/min, λ= 312 nm, retention time: 42.60 min (major) and 45.16 min (minor).

(*S*)-2-(3-chloro-4-methoxyphenyl)-2-(4-methoxy-3-((4-(methoxycarbonyl)-phenyl)-ethynyl)-phenyl)-propanoic acid (58)

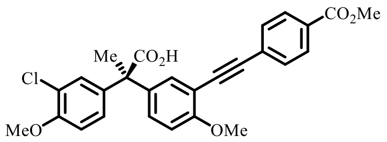

White solid, 62 mg, 52%, $[\alpha]_D^{20}$ = +15.0 (*c* 1.000 $CHCl_3$) for 98:2 er. ^{1}H NMR (500 MHz, Chloroform-*d*) δ 8.00 (dd, *J* = 8.3, 1.6 Hz, 2H), 7.59 (dd, *J* = 8.5, 1.6 Hz, 2H), 7.44 – 7.39 (m, 1H), 7.30 – 7.27 (m, 1H), 7.24 – 7.21 (m, 1H), 7.14 – 7.11 (m, 1H), 6.89 – 6.85 (m, 2H), 3.94 – 3.88 (m, 9H), 1.90 (s, 3H). ^{13}C NMR (126 MHz, Chloroform-*d*) δ 179.9, 166.7, 159.2, 154.0, 136.8, 135.4, 133.0, 131.6, 130.1, 129.8, 129.4, 129.4, 128.1, 127.5, 122.2, 111.8, 111.6, 110.5, 92.8, 88.7, 56.2, 56.0, 54.8, 52.2, 26.9. HRMS (m/z, ESI): Calcd. for Chemical Formula: $C_{27}H_{23}ClNaO_6^+$ $[M+Na]^+$: 501.1075, Found: 501.1072. HPLC analysis of the reaction product: Daicel Chiralpak IA, hexane/*iso*-propanol = 80: 20, 1.0 mL/min, λ= 324 nm, retention time: 9.07 min (major) and 10.76 min (minor).

(*S*)-2-(3-chloro-4-methoxyphenyl)-2-(3-((4-cyanophenyl)-ethynyl)-4-methoxyphenyl)-propanoic acid (59)

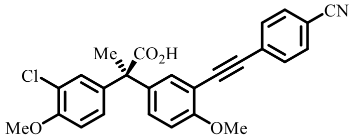

Yellow solid, 62 mg, 56%, $[\alpha]_D^{20}$ = +15.15 (*c* 1.000 $CHCl_3$) for 96:4 er. ^{1}H NMR (500 MHz, Chloroform-*d*) δ 7.61 (d, *J* = 1.0 Hz, 4H), 7.40 (dd, *J* = 2.6, 0.9 Hz, 1H), 7.28 (dd, *J* = 2.4, 1.0 Hz, 1H), 7.26 – 7.22 (m, 1H), 7.14 – 7.11 (m, 1H), 6.91 – 6.84 (m, 2H), 3.91 (s, 3H), 3.89 (s, 3H), 1.90 (s, 3H). ^{13}C NMR (126 MHz, Chloroform-*d*) δ 179.6, 159.3, 154.1, 136.7, 135.5, 133.0, 132.1, 132.0, 130.4, 129.8, 128.4, 127.4,

122.2, 118.6, 111.6, 111.34, 111.29, 110.6, 91.9, 90.2, 56.2, 56.0, 54.8, 26.9. HRMS (m/z, ESI): Calcd. for Chemical Formula: $C_{26}H_{20}ClNNaO_4^+$ $[M+Na]^+$: 468.0973, Found: 468.0980. HPLC analysis of the reaction product: Daicel Chiralpak IA, hexane/*iso*-propanol = 80: 20, 1.0 mL/min, λ= 326 nm, retention time: 9.91 min (major) and 17.76 min (minor).

Ethyl (*S*)-2-(3-chloro-4-methoxyphenyl)-2-(4-methoxy-3-(naphthalen-1-ylethynyl)-phenyl) propanoate (60)

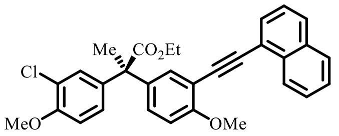

White solid, 62 mg, 50%, $[\alpha]_D^{20}$ = +1.03 (*c* 1.000 $CHCl_3$) for 95:5 er. ^{1}H NMR (400 MHz, Chloroform-*d*) δ 8.56 – 8.53 (m, 1H), 7.87 – 7.81 (m, 2H), 7.76 (dd, *J* = 7.2, 1.2 Hz, 1H), 7.67 – 7.51 (m, 1H), 7.55 – 7.50 (m, 1H), 7.48 – 7.41 (m, 2H), 7.28 (d, *J* = 2.4 Hz, 1H), 7.18 (dd, *J* = 8.8, 2.6 Hz, 1H), 7.10 (dd, *J* = 8.7, 2.4 Hz, 1H), 6.88 (t, *J* = 8.4 Hz, 2H), 4.24 (q, *J* = 7.1 Hz, 2H), 3.98 (s, 3H), 3.90 (s, 3H), 1.91 (s, 3H), 1.25 (t, *J* = 7.1 Hz, 3H). ^{13}C NMR (101 MHz, Chloroform-*d*) δ 174.7, 159.1, 153.8, 137.7, 136.2, 133.4, 133.2, 132.5, 130.1, 129.9, 129.6, 128.7, 128.2, 127.5, 126.7, 126.6, 126.4, 125.3, 122.0, 121.2, 112.4, 111.5, 110.4, 91.8, 90.8, 61.5, 56.2, 56.0, 54.9, 27.1, 14.1. HRMS (m/z, ESI): Calcd. for Chemical Formula: $C_{31}H_{28}ClO_4^+$ $[M+H]^+$: 499.1671, Found: 499.1673. The ester product was provided for the HPLC analysis of the reaction product: Daicel Chiralpak IA, hexane/*iso*-propanol = 99: 1, 1.0 mL/min, λ= 329 nm, retention time: 31.63 min (major) and 34.98 min (minor).

Ethyl (*S*)-2-(3-((9H-fluoren-2-yl)-ethynyl)-4-methoxyphenyl)-2-(3-chloro-4-methoxyphenyl)-propanoate (61)

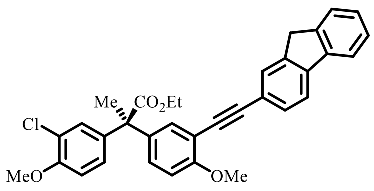

White solid, 95 mg, 71%, $[\alpha]_D^{20}$ = +17.05 (*c* 1.000 $CHCl_3$) for 97:3 er. ^{1}H NMR (400 MHz, Chloroform-*d*) δ 7.81 – 7.70 (m, 3H), 7.60 – 7.53 (m, 2H), 7.41 – 7.36 (m, 2H), 7.34 – 7.30 (m, 1H), 7.26 (d, *J* = 2.8 Hz, 1H), 7.15 (dd, *J* = 8.8, 2.6 Hz, 1H), 7.09 (dd, *J* = 8.7, 2.4 Hz, 1H), 6.86 (dd, *J* = 8.8, 6.5 Hz, 2H), 4.23 (q, *J* = 7.1 Hz, 2H), 3.93 (s, 3H), 3.91 – 3.90 (m, 5H), 1.89 (s, 3H), 1.24 (t, *J* = 7.1 Hz, 3H). ^{13}C NMR (101 MHz, Chloroform-*d*) δ 174.7, 158.8, 153.8, 143.6, 143.1, 141.8, 141.2, 137.7, 136.1, 132.8, 130.6, 129.9, 129.3, 128.3, 127.5, 127.1, 126.9, 125.1, 122.0, 121.5, 120.2, 119.7, 112.3, 111.5, 110.4, 94.3, 85.7, 61.5, 56.2, 56.0, 54.9, 36.7, 27.1, 14.1. HRMS (m/z, ESI): Calcd. for Chemical Formula: $C_{34}H_{30}ClNO_4^+$ $[M+H]^+$: 537.1827, Found: 537.1830. The ester product was provided for the HPLC analysis of the reaction product: Daicel Chiralpak IA, hexane/*iso*-propanol = 99: 1, 1.0 mL/min, λ= 325nm, retention time: 35.16 min (major) and 38.35 min (minor).

(*S*)-2-(3-chloro-4-methoxyphenyl)-2-(4-methoxy-3-((6-methoxypyridin-3-yl)-ethynyl)-phenyl) -propanoic acid (62)

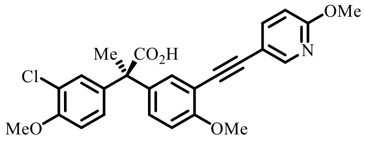

White solid, 60 mg, 53%, $[\alpha]_D^{20}$ = +28.60 (*c* 0.500 $CHCl_3$) for 96:4 er. ^{1}H NMR (400 MHz, Chloroform-*d*) δ 8.36 (dd, *J* = 2.3, 0.8 Hz, 1H), 7.70 (dd, *J* = 8.6, 2.3 Hz, 1H), 7.40 (d, *J* = 2.5 Hz, 1H), 7.28 (t, *J* = 2.5 Hz, 1H), 7.20 (dd, *J* = 8.8, 2.6 Hz, 1H), 7.12 (dd, *J* = 8.6, 2.5 Hz, 1H), 6.89 – 6.83 (m, 2H), 6.71 (dd, *J* = 8.6, 0.8 Hz, 1H), 3.95 (s, 3H), 3.91 (s, 3H), 3.89 (s, 3H), 1.90 (s, 3H). ^{13}C NMR (101 MHz, Chloroform-*d*)

δ 179.4, 163.3, 158.9, 154.0, 150.1, 141.4, 136.9, 135.4, 132.8, 129.8, 129.6, 127.5, 122.2, 113.3, 112.1, 111.6, 110.6, 110.5, 90.4, 86.9, 56.2, 56.0, 54.8, 53.7, 27.0. HRMS (m/z, ESI): Calcd. for Chemical Formula: $C_{25}H_{23}ClNO_5^+$ $[M+H]^+$: 452.1259, Found: 452.1260. HPLC analysis of the reaction product: Daicel Chiralpak IA, hexane/*iso*-propanol = 80: 20, 1.0 mL/min, λ= 311 nm, retention time: 8.21 min (major) and 10.65 min (minor).

Optimization of reaction conditions for Buchwald–Hartwig cross-coupling

Table S3 ligand sources[a]

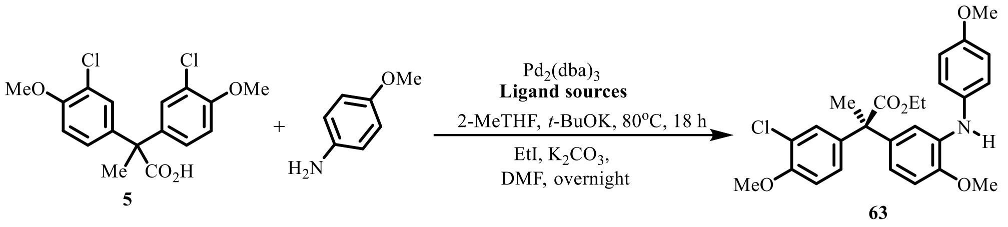

Entry	Ligand sources	Yield[b]	er[c]
1	**(*R*a, *S*)-L8**	20%	65.5:34.5
2	**(*R*a, *S*)-L11**	15%	65:35
3	**(*R*a, *S*)-L12**	trace	
4	**(*R*a, *S*)-L13**	trace	

[a]Unless otherwise stated, the reactions were performed with the substrate (0.1 mmol), *p*-anisidine (0.12 mmol), $Pd_2(dba)_3$ (1.0 mol%), Ligand sources (2.0 mol%), *t*-BuOK (0.3 mmol) in 1.0 mL 2-MeTHF at 80°C for 18 h. [b]The yield of the product was isolated yield by column chromatography. [c]Determined using chiral chromatography.

Table S4 Bases[a]

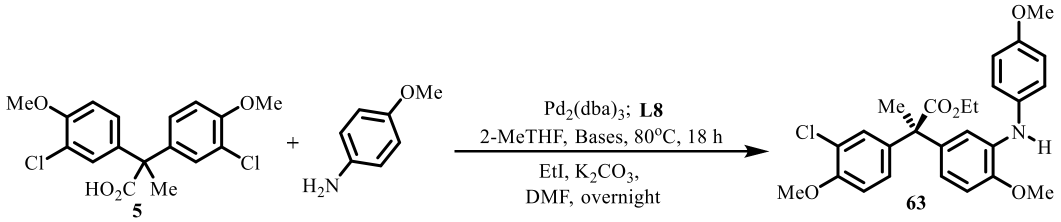

Entry	Bases	Yield[b]	er[c]
1	*t*-BuOK	20%	65.5:34.5
2	*t*-BuONa	53%	70:30
3	LiHMDS	trace	
4	K_3PO_4	43%	87:13

[a]Unless otherwise stated, the reactions were performed with the substrate (0.1 mmol), *p*-anisidine (0.12 mmol), $Pd_2(dba)_3$ (1.0 mol%), **L 8** (2.0 mol%), Bases (0.3 mmol) in 1.0 mL 2-MeTHF at 80ºC for 18 h. [b]The yield of the product was isolated yield by column chromatography. [c]Determined using chiral chromatography.

Table S5 Solvents and temperature[a]

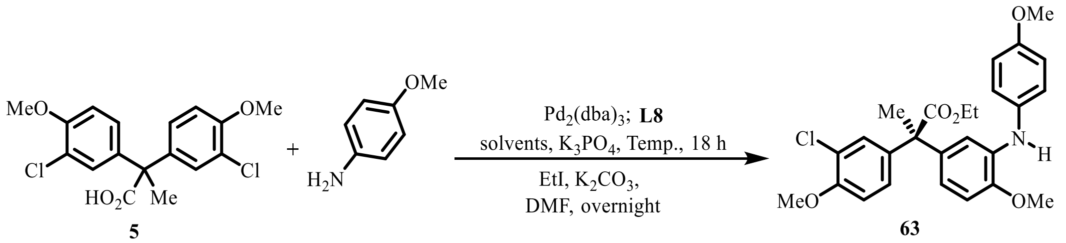

Entry	Solvents	Temp.	*p*-anisidine	K_3PO_4	Yield[b]	er[c]
1	2-MeTHF	80°C	0.12 mmol	0.3 mmol	43%	87:13
2	toluene	80°C	0.12 mmol	0.3 mmol	32%	84:16
3	1,4-dioxane	110°C	0.15 mmol	0.6 mmol	64%	97:3

[a]Unless otherwise stated, the reactions were performed with the substrate (0.1 mmol), *p*-anisidine, $Pd_2(dba)_3$ (1.0 mol%), **L 8** (2.0 mol%), K_3PO_4 in 1.0 mL solvents at temperature for 18 h. [b]The yield of the product was isolated yield by column chromatography. [c]Determined using chiral chromatography.

The general procedure for Buchwald–Hartwig cross-coupling desymmetrization reactions

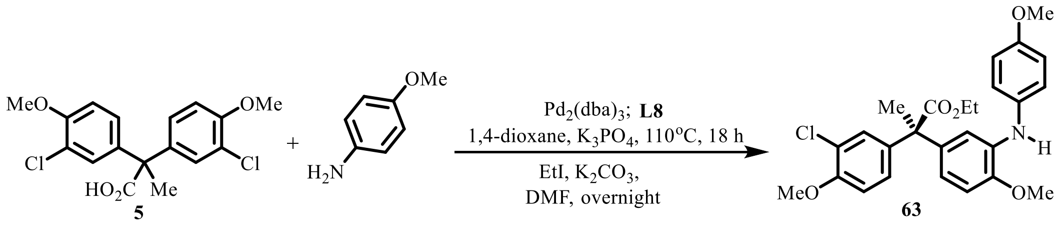

Under N_2 atmosphere, a mixture of $Pd_2(dba)_3$ (2.3 mg, 0.0025 mmol) and **L8** (3.2 mg, 0.005 mmol) was added to 0.5 mL 1,4-dioxane, the mixture was then stirred at room temperature for 20 min. The resulting metal-ligand complex solution was added to a reaction flask containing 2.0 mL 1,4-dioxane solution of 2,2-bis(3-chloro-4-methoxyphenyl)-propanoic acid **5** (88.5 mg, 0.25 mmol), *p*-anisidine (46.1 mg, 0.375 mmol), and K_3PO_4 (318 mg, 1.25 mmol). Then the resulting reaction mixture was stirred at 110°C for 18 h. The reaction was then quenched with water, neutralized to pH 4 – 6 with saturated $KH_2PO_{4\,(aq.)}$, and extracted with ethyl acetate (4 mL X 3) three times. The combined organic phase was washed with brine, dried over Na_2SO_4, and concentrated in vacuo. After that, the obtained crude product was dissolved in DMF (1.0 mL), and treated with EtI (78 mg, 0.5 mmol) and K_2CO_3 (138 mg, 1.0 mmol). The reaction mixture was stirred overnight at room temperature. Then the mixture was diluted with ethyl acetate (20 mL) and washed with water. The organic layer was concentrated in vacuo and purified by flash column chromatography with hexane and acetone as the eluents to give the desired product.

Characterization data of desymmetrization products of Buchwald–Hartwig cross-coupling reactions

Ethyl (*S*)-2-(3-chloro-4-methoxyphenyl)-2-(4-methoxy-3-((4-methoxyphenyl)-amino)-phenyl)-propanoate (63)

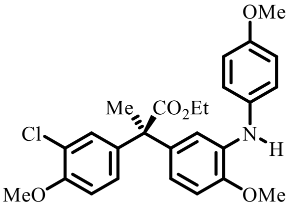

Reddish solid, 75 mg, 64%, $[\alpha]_D^{20}$ = -38.10 (*c* 0.500 $CHCl_3$) for 97:3 er. ^1H NMR (400 MHz, Chloroform-*d*) δ 7.27 (d, *J* = 2.4 Hz, 1H), 7.09 (dd, *J* = 8.6, 2.4 Hz, 1H), 7.04 – 6.99 (m, 2H), 6.92 (d, *J* = 2.3 Hz, 1H), 6.85 – 6.75 (m, 4H), 6.59 (dd, *J* = 8.4, 2.3 Hz, 1H), 5.95 (s, 1H), 4.19 – 4.13 (m, 2H), 3.89 (s, 3H), 3.88 (s, 3H), 3.78 (s, 3H), 1.81 (s, 3H), 1.18 (t, *J* = 7.1 Hz, 3H). ^{13}C NMR (101 MHz, Chloroform-*d*) δ 175.0, 155.0, 153.6, 146.4, 138.0, 136.8, 135.4, 134.1, 130.2, 127.4, 121.8, 121.7, 117.8, 114.6, 113.2, 111.3, 109.7, 61.3, 56.1, 55.6, 55.6, 55.3, 27.1, 14.0. HRMS (m/z, ESI): Calcd. for Chemical Formula: $C_{26}H_{29}ClNO_5^+$ $[M+H]^+$: 470.1729, Found: 470.1730. HPLC analysis of the reaction product: Daicel Chiralpak IB, hexane/*iso*-propanol = 99: 1, 1.0 mL/min, λ= 281 nm, retention time: 20.23 min (major) and 24.03 min (minor).

Ethyl (*S*)-2-(3-chloro-4-methoxyphenyl)-2-(4-methoxy-3-(phenylamino)-phenyl)-propanoate (64)

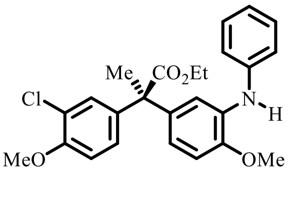

Yellowish oil, 56 mg, 51%, $[\alpha]_D^{20}$ = -47.01 (*c* 0.500 $CHCl_3$) for 96:4 er. ^1H NMR (500 MHz, Chloroform-*d*) δ 7.30 (d, *J* = 2.4 Hz, 1H), 7.24 – 7.19 (m, 2H), 7.15 (d, *J* = 2.3 Hz, 1H), 7.11 (dd, *J* = 8.6, 2.4 Hz, 1H), 7.06 – 7.02 (m, 2H), 6.91 – 6.86 (m, 1H), 6.84 (d, *J* = 8.7 Hz, 1H), 6.81 (d, *J* = 8.4 Hz, 1H), 6.68

(dd, J = 8.5, 2.4 Hz, 1H), 4.23 – 4.15 (m, 2H), 3.89 (s, 3H), 3.88 (s, 3H), 1.84 (s, 3H), 1.21 (t, J = 7.1 Hz, 3H). ^{13}C NMR (126 MHz, Chloroform-*d*) δ 175.0, 153.6, 147.3, 142.7, 137.9, 136.7, 132.1, 130.2, 129.3, 127.4, 121.8, 121.0, 119.1, 117.9, 115.4, 111.3, 109.9, 61.4, 56.2, 55.6, 55.3, 27.2, 14.1. HRMS (m/z, ESI): Calcd. for Chemical Formula: $C_{25}H_{27}ClNO_4^+$ $[M+H]^+$: 440.1623, Found: 440.1624. HPLC analysis of the reaction product: Daicel Chiralpak IB, hexane/*iso*-propanol = 99: 1, 1.0 mL/min, λ= 292 nm, retention time: 21.04 min (major) and 32.67 min (minor).

Ethyl (*S*)-2-(3-chloro-4-methoxyphenyl)-2-(4-methoxy-3-(p-tolylamino)-phenyl)-propanoate (65)

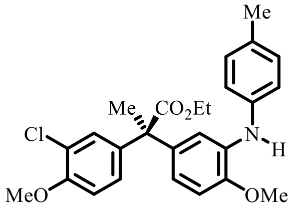

Yellowish oil, 69 mg, 61%, $[\alpha]_D^{20}$ = -44.70 (c 0.500 $CHCl_3$) for 92:8 er. ^{1}H NMR (500 MHz, Chloroform-*d*) δ 7.29 (d, J = 2.4 Hz, 1H), 7.12 – 7.07 (m, 2H), 7.06 – 7.02 (m, 2H), 6.98 – 6.94 (m, 2H), 6.83 (d, J = 8.7 Hz, 1H), 6.79 (d, J = 8.4 Hz, 1H), 6.64 (dd, J = 8.4, 2.4 Hz, 1H), 4.23 – 4.13 (m, 2H), 3.89 (s, 3H), 3.88 (s, 3H), 2.28 (s, 3H), 1.83 (s, 3H), 1.20 (t, J = 7.1 Hz, 3H). ^{13}C NMR (126 MHz, Chloroform-*d*) δ 175.0, 153.6, 146.9, 139.9, 138.0, 136.7, 132.9, 130.7, 130.1, 129.8, 127.4, 121.8, 118.7, 118.4, 114.4, 111.3, 109.8, 61.4, 56.1, 55.6, 55.3, 27.2, 20.7, 14.0. HRMS (m/z, ESI): Calcd. for Chemical Formula: $C_{26}H_{29}ClNO_4^+$ $[M+H]^+$: 454.1780, Found: 454.1782. HPLC analysis of the reaction product: Daicel Chiralpak IB, hexane/*iso*-propanol = 99: 1, 1.0 mL/min, λ= 282 nm, retention time: 14.09 min (major) and 17.03 min (minor).

Ethyl (*S*)-2-(3-chloro-4-methoxyphenyl)-2-(4-methoxy-3-((4-(methylthio)-phenyl)-amino)-phenyl)-propanoate (66)

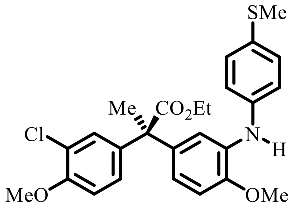

Reddish solid, 68 mg, 56%, $[\alpha]_D^{20}$ = -29.80 (*c* 0.500 $CHCl_3$) for 96:4 er. ^{1}H NMR (500 MHz, Chloroform-*d*) δ 7.28 (d, *J* = 2.4 Hz, 1H), 7.21 – 7.15 (m, 2H), 7.12 – 7.06 (m, 2H), 6.97 (d, *J* = 8.3 Hz, 2H), 6.84 (d, *J* = 8.7 Hz, 1H), 6.80 (d, *J* = 8.5 Hz, 1H), 6.69 (dd, *J* = 8.5, 2.3 Hz, 1H), 4.22 – 4.15 (m, 2H), 3.89 (s, 3H), 3.88 (s, 3H), 2.44 (s, 3H), 1.83 (s, 3H), 1.21 (t, *J* = 7.1 Hz, 3H). ^{13}C NMR (126 MHz, Chloroform-*d*) δ 174.9, 153.7, 147.3, 140.9, 137.9, 136.8, 132.0, 130.1, 129.8, 129.0, 127.3, 121.9, 119.2, 118.6, 115.4, 111.3, 109.9, 61.4, 56.2, 55.6, 55.2, 27.1, 17.8, 14.1. HRMS (m/z, ESI): Calcd. for Chemical Formula: $C_{26}H_{29}ClNO_4S^+$ $[M+H]^+$: 486.1500, Found: 486.1501. A sample of the ester was reduced to provide the parent primary alcohol for the HPLC analysis of the reaction product: Daicel Chiralpak IA, hexane/*iso*-propanol = 80: 20, 1.0 mL/min, λ= 310 nm, retention time: 25.03 min (major) and 13.52 min (minor).

Ethyl (*S*)-2-(3-chloro-4-methoxyphenyl)-2-(3-((3,5-dimethoxyphenyl)-amino)-4-methoxyphen-yl)-propanoate (67)

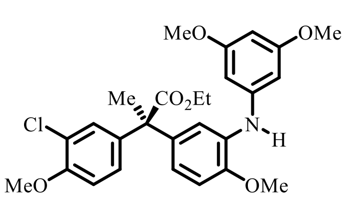

White solid, 79 mg, 63%, $[\alpha]_D^{20}$ = -27.03 (*c* 0.500 $CHCl_3$) for 95:5 er. ^{1}H NMR (500 MHz, Chloroform-*d*) δ 7.27 – 7.24 (m, 2H), 7.11 (dd, *J* = 8.7, 2.4 Hz, 1H), 6.84 (d, *J* = 8.7 Hz, 1H), 6.80 (d, *J* = 8.4 Hz, 1H), 6.68 (dd, *J* = 8.5, 2.4 Hz, 1H), 6.22 (d, *J* = 2.2 Hz, 2H), 6.03 (t, *J* = 2.2 Hz, 1H), 4.23 – 4.16 (m, 2H), 3.88 (s, 3H), 3.87 (s, 3H), 3.70 (s, 6H), 1.85 (s, 3H), 1.20 (t, *J* = 7.1 Hz, 3H). ^{13}C NMR (126 MHz, Chloroform-*d*) δ 174.9, 161.6, 153.6, 147.5, 144.6, 138.0, 136.7, 131.7, 129.9, 127.5,

121.8, 119.3, 116.2, 111.3, 109.9, 95.8, 93.5, 61.4, 56.1, 55.6, 55.2, 55.2, 27.1, 14.0. HRMS (m/z, ESI): Calcd. for Chemical Formula: $C_{27}H_{31}ClNO_6^+$ $[M+H]^+$: 500.1834, Found: 500.1833. HPLC analysis of the reaction product: Daicel Chiralpak IB, hexane/*iso*-propanol = 99: 1, 1.0 mL/min, λ= 307 nm, retention time: 33.77 min (major) and 44.78 min (minor).

Ethyl (*S*)-2-(3-chloro-4-methoxyphenyl)-2-(4-methoxy-3-((4-(trifluoromethyl)-phenyl)-amino) -phenyl)-propanoate (68)

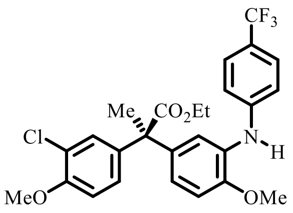

Yellowish oil, 71 mg, 56%, $[\alpha]_D^{20}$ = -11.90 (*c* 0.500 $CHCl_3$) for 97.5:2.5 er. ^1H NMR (400 MHz, Chloroform-*d*) δ 7.45 – 7.40 (m, 2H), 7.28 (d, *J* = 2.4 Hz, 1H), 7.16 (d, *J* = 2.1 Hz, 1H), 7.12 (dd, *J* = 8.6, 2.4 Hz, 1H), 7.04 – 6.99 (m, 2H), 6.88 – 6.79 (m, 3H), 4.25 – 4.16 (m, 2H), 3.89 (s, 3H), 3.88 (s, 3H), 1.86 (s, 3H), 1.23 (t, *J* = 7.1 Hz, 3H). ^{13}C NMR (101 MHz, Chloroform-*d*) δ 174.8, 153.8, 148.3, 146.2, 137.8, 136.8, 130.1 (d, *J* = 9.8 Hz), 127.2, 126.6 (q, *J* = 3.7 Hz), 124.6 (q, *J* = 270.7 Hz), 122.0, 121.9, 121.8 (q, *J* = 32.7 Hz), 121.0, 117.8, 115.7, 111.4, 110.2, 61.5, 56.2, 55.7, 55.2, 27.1, 14.1. ^{19}F NMR (377 MHz, Chloroform-*d*) δ - 61.47 (s, 3F). HRMS (m/z, ESI): Calcd. for Chemical Formula: $C_{26}H_{26}ClF_3NO_4^+$ $[M+H]^+$: 508.1497, Found: 508.1497. HPLC analysis of the reaction product: Daicel Chiralpak IB, hexane/*iso*-propanol = 99: 1, 1.0 mL/min, λ= 307 nm, retention time: 16.60 min (major) and 19.00 min (minor).

Ethyl (*S*)-4-((5-(2-(3-chloro-4-methoxyphenyl)-1-ethoxy-1-oxopropan-2-yl)-2-methoxyphenyl) -amino)-benzoate (69)

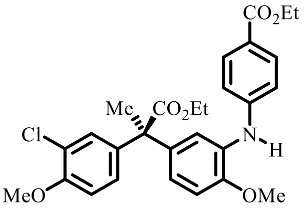

White solid, 49 mg, 38%, $[\alpha]_D^{20}$ = -14.70 (*c* 0.500 $CHCl_3$) for 99.5:0.5 er. ^{1}H NMR (400 MHz, Chloroform-*d*) δ 7.91 – 7.84 (m, 2H), 7.27 (d, *J* = 2.4 Hz, 1H), 7.22 (d, *J* = 2.2 Hz, 1H), 7.12 (dd, *J* = 8.7, 2.4 Hz, 1H), 7.00 – 6.94 (m, 2H), 6.88 – 6.78 (m, 3H), 4.33 (q, *J* = 7.1 Hz, 2H), 4.25 – 4.17 (m, 2H), 3.90 (s, 3H), 3.88 (s, 3H), 1.86 (s, 3H), 1.37 (t, *J* = 7.1 Hz, 3H), 1.23 (t, *J* = 7.1 Hz, 3H). ^{13}C NMR (126 MHz, Chloroform-*d*) δ 174.8, 166.5, 153.7, 148.4, 147.3, 137.8, 136.7, 131.3, 130.0, 127.3, 122.0, 121.6, 121.1, 118.1, 115.0, 111.4, 110.2, 61.5, 60.4, 56.2, 55.7, 55.2, 27.1, 14.4, 14.1. HRMS (m/z, ESI): Calcd. for Chemical Formula: $C_{28}H_{31}ClNO_6^+$ $[M+H]^+$: 512.1834, Found: 512.1834. HPLC analysis of the reaction product: Daicel Chiralpak IF, hexane/*iso*-propanol = 97: 3, 1.0 mL/min, λ= 326 nm, retention time: 39.16 min (major) and 33.91 min (minor).

Ethyl (*S*)-2-(3-chloro-4-methoxyphenyl)-2-(4-methoxy-3-(naphthalen-1-ylamino)-phenyl)-propanoate (70)

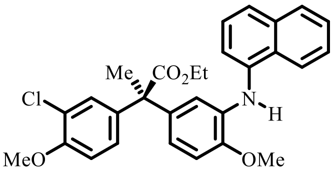

Reddish solid, 56 mg, 46%, $[\alpha]_D^{20}$ = -28.10 (*c* 0.500 $CHCl_3$) for 94.5:5.5 er. ^{1}H NMR (500 MHz, Chloroform-*d*) δ 7.71 (dd, *J* = 8.5, 5.6 Hz, 2H), 7.56 (d, *J* = 8.3 Hz, 1H), 7.41 – 7.33 (m, 3H), 7.31 – 7.27 (m, 1H), 7.26 (d, *J* = 2.0 Hz, 1H), 7.20 (dd, *J* = 8.8, 2.3 Hz, 1H), 7.16 (dd, *J* = 8.7, 2.4 Hz, 1H), 6.85 (dd, *J* = 8.6, 2.9 Hz, 2H), 6.77 – 6.72 (m, 1H), 4.24 – 4.17 (m, 2H), 3.91 (s, 3H), 3.89 (s, 3H), 1.87 (s, 3H), 1.24 – 1.18 (m, 3H). ^{13}C NMR (126 MHz, Chloroform-*d*) δ 174.9, 153.7, 147.4, 140.2, 137.9, 137.0, 134.6, 132.0, 130.2, 129.2, 129.0, 127.6, 127.4, 126.5, 126.3, 123.4, 121.9, 120.3,

119.3, 115.7, 111.6, 111.3, 109.9, 61.4, 56.1, 55.7, 55.3, 27.1, 14.1. HRMS (m/z, ESI): Calcd. for Chemical Formula: $C_{29}H_{29}ClNO_4^+$ [M+H]$^+$: 490.1780, Found: 490.1780. A sample of the ester was reduced to provide the parent primary alcohol for the HPLC analysis of the reaction product: Daicel Chiralpak IA, hexane/*iso*-propanol = 80: 20, 1.0 mL/min, λ= 266 nm, retention time: 12.02 min (major) and 19.19 min (minor).

Ethyl (*S*)-2-(3-(benzo[d]thiazol-5-ylamino)-4-methoxyphenyl)-2-(3-chloro-4-methoxyphenyl) -propanoate (71)

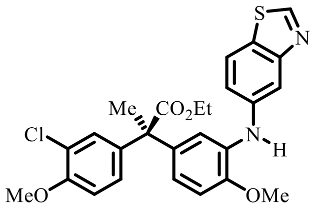

Yellowish oil, 66 mg, 53%, $[\alpha]_D^{20}$ = -53.5 (*c* 0.500 $CHCl_3$) for 99:1 er. ^{1}H NMR (400 MHz, Chloroform-*d*) δ 8.87 (s, 1H), 7.72 – 7.66 (m, 2H), 7.21 (d, *J* = 2.4 Hz, 1H), 7.13 (dd, *J* = 10.1, 2.3 Hz, 2H), 7.06 (dd, *J* = 8.6, 2.4 Hz, 1H), 6.78 (dd, *J* = 9.5, 8.6 Hz, 2H), 6.66 (dd, *J* = 8.5, 2.3 Hz, 1H), 4.16 – 4.10 (m, 2H), 3.84 (s, 3H), 3.82 (s, 3H), 1.78 (s, 3H), 1.13 (t, *J* = 7.1 Hz, 3H). ^{13}C NMR (101 MHz, Chloroform-*d*) δ 174.9, 154.7, 153.6, 147.6, 142.0, 137.8, 136.9, 131.9, 130.0, 127.5, 122.1, 121.8, 119.8, 118.2, 115.7, 111.5, 111.4, 110.0, 61.5, 56.1, 55.7, 55.2, 27.2, 14.1. HRMS (m/z, ESI): Calcd. for Chemical Formula: $C_{26}H_{26}ClN_2O_5S^+$ [M+H]$^+$: 497.1296, Found: 497.1297. HPLC analysis of the reaction product: Daicel Chiralpak IB, hexane/*iso*-propanol = 97: 3, 1.0 mL/min, λ= 308 nm, retention time: 47.01 min (major) and 41.60 min (minor).

Control experiments

1. The importance of K^+

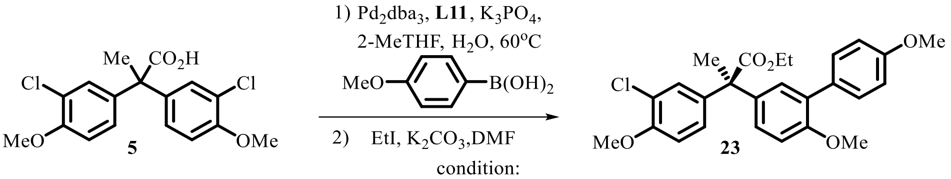

condition:
1. no additive : 79% yield, 98:2 er
2. 3.0 equiv 18-crown-6: 55% conv., 58:42 er
3. 7.0 equiv 18-crown-6: 47% conv., 58:42 er

To the mixture of 2,2-bis(3-chloro-4-methoxyphenyl)-propanoic acid **5** (0.1 mmol, 1.0 equivalent), K_3PO_4 (0.3 mmol) and 18-crown-6 were added 1.5 mL 2-MeTHF and the resulting reaction mixture was stirring for 20 min, followed by addition of 4-methoxyphenylboronic acid (1.2 equivalent), **L11** and Pd_2dba_3 complex solution (1.0 mol%), and water following the general procedure. The conversion of **30** was determined by their acid (1 step) crude ^{1}H NMR.

2. The importance of the carboxylate group of the substrate

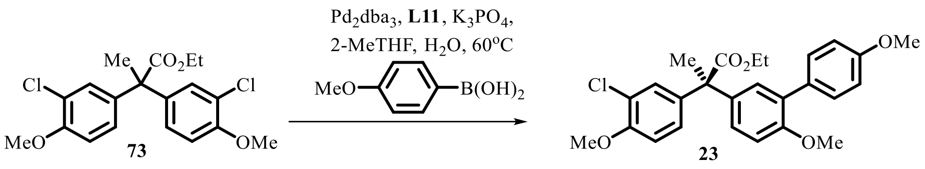

30% conv., 57:43 er

The procedure was the same as the procedure of the Suzuki cross-coupling desymmetrization reaction. The conversion was determined by the crude ^{1}H NMR.

Competition experiments

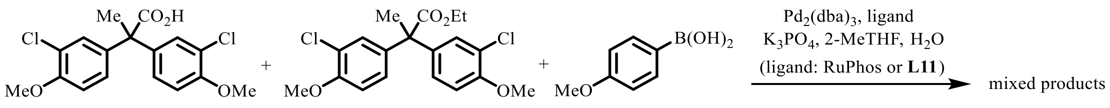

To the mixture of 2,2-bis(3-chloro-4-methoxyphenyl)-propanoic acid (35.4 mg, 0.1 mmol), ethyl 2,2-bis(3-chloro-4-methoxyphenyl)-propanoate (38.2 mg, 0.1 mmol), and K_3PO_4 (106 mg, 0.5 mmol) were added 2.5 mL 2-MeTHF and 0.1 mL H_2O. The resulting reaction mixture was stirred for 20 min, followed by the addition of 4-Methoxyphenylboronic acid (15.2 mg, 0.1 mmol), then 0.5 mL 2-MeTHF, **L11** (1.3 mg, 0.02 mmol) or RuPhos (0.93 mg, 0.002 mmol) and Pd_2dba_3 (0.92 mg, 0.001 mmol) complex solution was added. The resulting reaction mixture was stirred at 60^oC for 20 h. The conversion of each substrate was determined by analysis of the ^1H NMR spectra of the reaction mixtures.

Entry	Ligand	Conv. CO_2Et	Conv. CO_2H
1	**L11**	< 5%	79%
2	RuPhos	55%	14%

Kinetic resolution

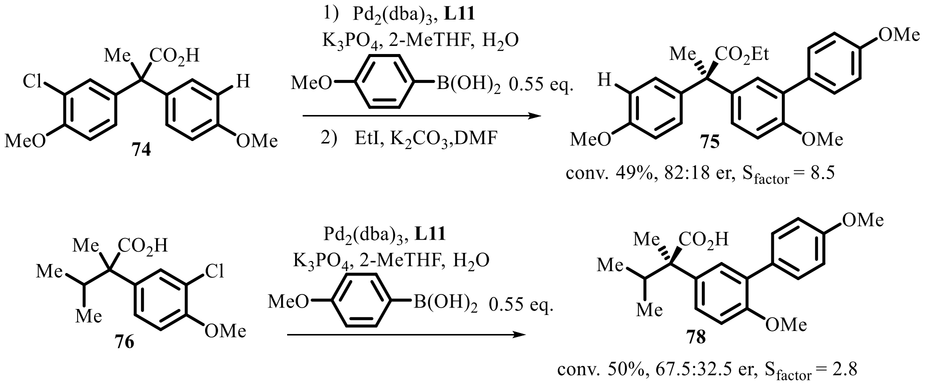

The procedure was the same as the procedure of the Suzuki cross-coupling desymmetrization reaction. The conversion was determined by the crude ^{1}H NMR.

Ethyl (*R*)-2-(4',6-dimethoxy-[1,1'-biphenyl]-3-yl)-2-(4-methoxyphenyl)-propanoate (75)

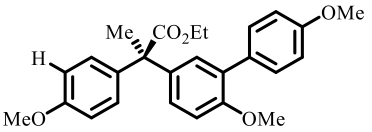

Conv. 49%, $[\alpha]_D^{20}$ = +2.50 (*c* 0.500 $CHCl_3$) for 82:18 er. ^{1}H NMR (500 MHz, Chloroform-*d*) δ 7.45 – 7.39 (m, 2H), 7.22 – 7.16 (m, 3H), 7.13 (dd, *J* = 8.5, 2.6 Hz, 1H), 6.95 – 6.91 (m, 2H), 6.89 (d, *J* = 8.6 Hz, 1H), 6.86 – 6.81 (m, 2H), 4.21 (q, *J* = 7.1 Hz, 2H), 3.83 (s, 3H), 3.81 – 3.80 (m, 6H), 1.90 (s, 3H), 1.23 (t, *J* = 7.1 Hz, 3H). ^{13}C NMR (126 MHz, Chloroform-*d*) δ 175.5, 158.7, 158.2, 155.1, 137.0, 136.9, 130.9, 130.6, 130.5, 129.6, 129.1, 127.6, 113.5, 113.3, 110.5, 61.2, 55.6, 55.3, 55.2, 55.2, 27.4, 14.1. HRMS (m/z, ESI): Calcd. for Chemical Formula: $C_{26}H_{28}NaO_5^+$ $[M+Na]^+$: 443.1829, Found: 443.1832. HPLC analysis of the reaction product: Daicel Chiralpak IA, hexane/*iso*-propanol = 97: 3, 1.0 mL/min, λ= 292 nm, retention time: 9.88 min (major) and 10.73 min (minor).

(*R*)-2-(4',6-dimethoxy-[1,1'-biphenyl]-3-yl)-2,3-dimethylbutanoic acid (77)

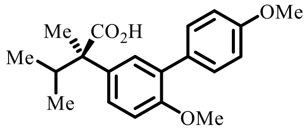

Conv. 50%, $[\alpha]_D^{20}$ = +0.70 (*c* 0.500 $CHCl_3$) for 67.5:32.5 er. ^{1}H NMR (500 MHz, Chloroform-*d*) δ 7.50 – 7.44 (m, 2H), 7.42 – 7.37 (m, 2H), 7.00 – 6.90 (m, 3H), 3.85 (s, 3H), 3.80 (s, 3H), 2.72 (p, *J* = 6.7 Hz, 1H), 1.49 (s, 3H), 1.02 (d, *J* = 6.7 Hz, 3H), 0.68 (d, *J* = 6.9 Hz, 3H). ^{13}C NMR (126 MHz, Chloroform-*d*) δ 182.2, 158.7, 155.3, 134.1, 131.0, 130.7, 129.8, 129.3, 126.5, 113.5, 110.8, 55.6, 55.3, 52.9, 34.5, 18.9, 17.1, 14.8. HRMS (m/z, ESI): Calcd. for Chemical Formula: $C_{20}H_{25}O_4^+$ $[M+H]^+$: 329.1747, Found: 329.1757. HPLC analysis of the reaction product: Daicel Chiralpak IA, hexane/*iso*-propanol = 97: 3, 1.0 mL/min, λ= 286 nm, retention time: 25.01 min (major) and 21.86 min (minor).

Computational study

To get a deeper insight into the reaction selectivity, DFT computations were performed using the Gaussian 16 set of programs.[15] Optimizations were carried out with the M11-L functional,[16] the LANL2DZ(ECP) basis set[17] for Pd and the 6-31G(d) basis set for the other elements. Thermal correction to the Gibbs free energy was obtained at the optimization level. SMD[18] single point energy calculations were achieved using the M11-L functional, the SDD (ECP)[19] basis set for Pd and the triple ζ6-311+ G(d,p) basis set for the other elements, using the parameters corresponding to a 19:1 v/v mixture of THF and H_2O. The values presented are solvent-corrected ΔG_{298} in kcal/mol.

The relative Gibbs free energies of diastereomeric oxidative addition transition states were computed. Regarding the various possible orientations between the metal center and the anionic phosphane ligand, we found having Pd close to the electron-rich aryl group of the biaryl core resulted in lower barriers, which agrees with the literature data.[20] This still leaves two possibilities for oxidative addition as shown in **Figure S1**. The preference for one or the other orientation is known to depend on the substitution pattern of the biaryl fragment.[20] In our case, Type 2 transition states were found more stable and therefore they will be the only type discussed below.

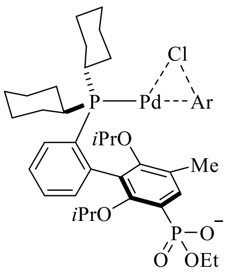

Type 1

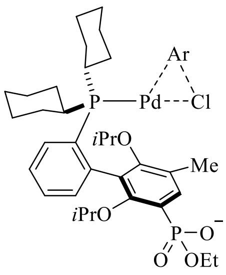

Type 2

Figure S1. Isomeric oxidative addition transition states (Pd-**L1** as the catalyst).

The study was specifically oriented toward the examples displayed in **Figure S2**, where ligand (*S*)-**L1** provided the *R* products as major stereoisomers, and the (*R*, *S*)-**L11** led to the *S* stereoisomer as the major one. Our intention was also to rationalize the increase of stereoselectivity conferred by (*R*, *S*)-**L11** compared to (*S*)-**L1**.

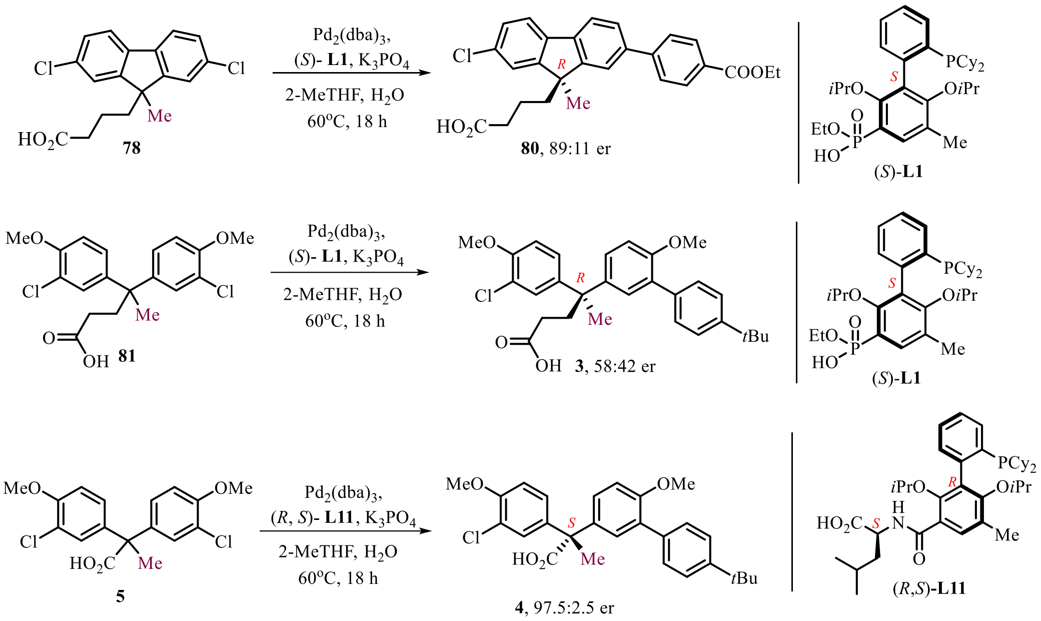

Figure S2. Experimental results to be studied computationally.

One or two potassium ions (@1K or @2K) were included in the computations (calculations without potassium could not be achieved due to convergence issues). The corresponding oxidative addition transition states are displayed in **Figure S3**.

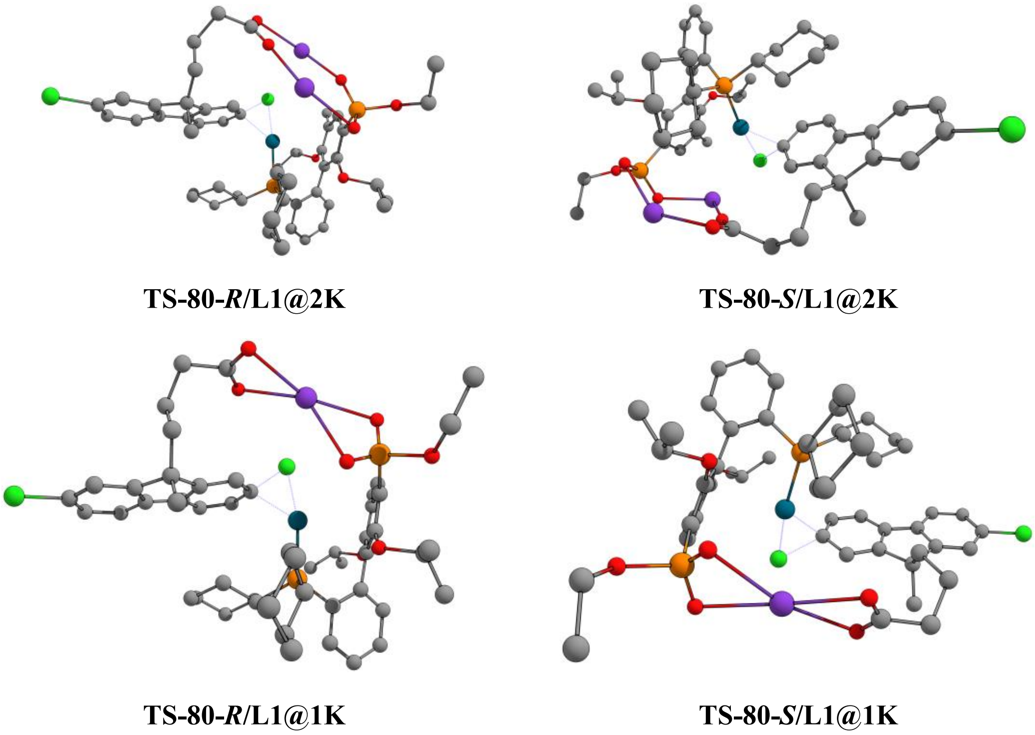

Figure S3. Computed oxidative addition transition states using compounds **80**, **3**, and **4**, and ligands **L1** and **L11** as shown in **Figure S2**.

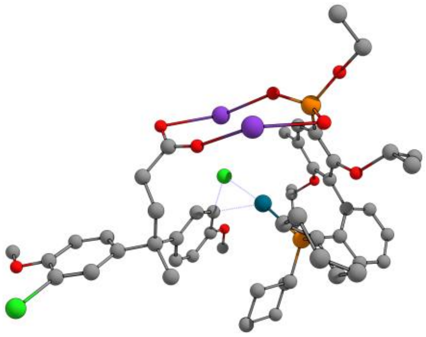

TS-3-*R*/L1@2K

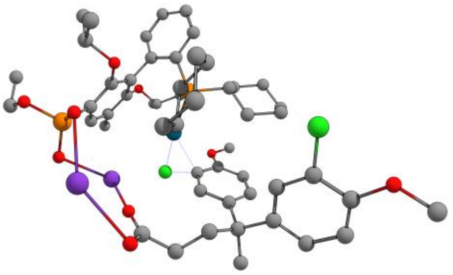

TS-3-*S*/L1@2K

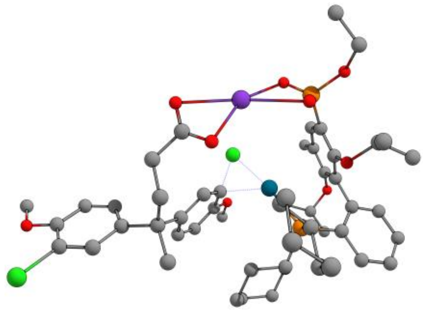

TS-3-*R*/L1@1K

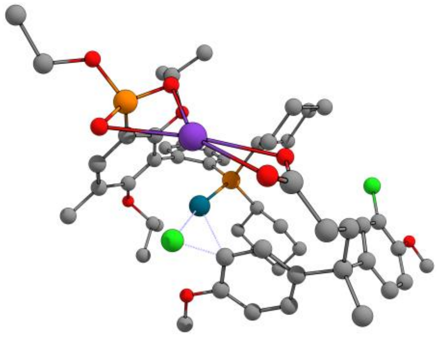

TS-3-*S*/L1@1K

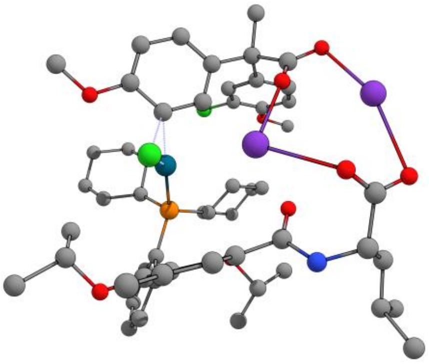

TS-4-*R*/L11@2K

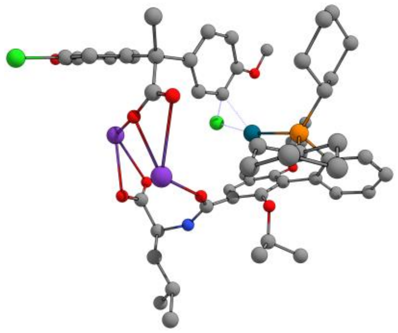

TS-4-*S*/L11@2K

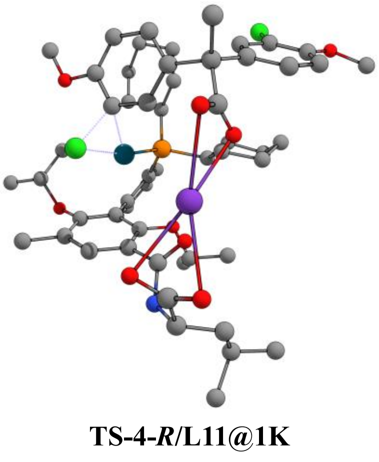

TS-4-*R*/L11@1K

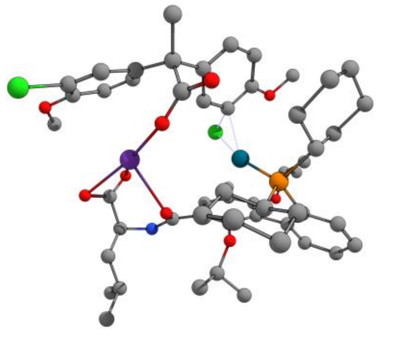

TS-4-*S*/L11@1K

Figure S3 (continued). Computed oxidative addition transition states using compounds **80**, **3**, and **4**, and ligands **L1** and **L11** as shown in **Figure S2**.

With 1 or 2 potassium ions, all reactions are markedly exergonic (between -17 to -34 kcal/mol; **Table S6**). The free energy barriers are all accessible at rt but span over a range between 2 to 13

kcal/mol. In any case, the oxidative additions are unlikely to be reversible.

Table S6. $\Delta G^{\ddagger}_{298}/\Delta G_{298}$ (kcal/mol) taking the pro-*R* Pd-complex as reference

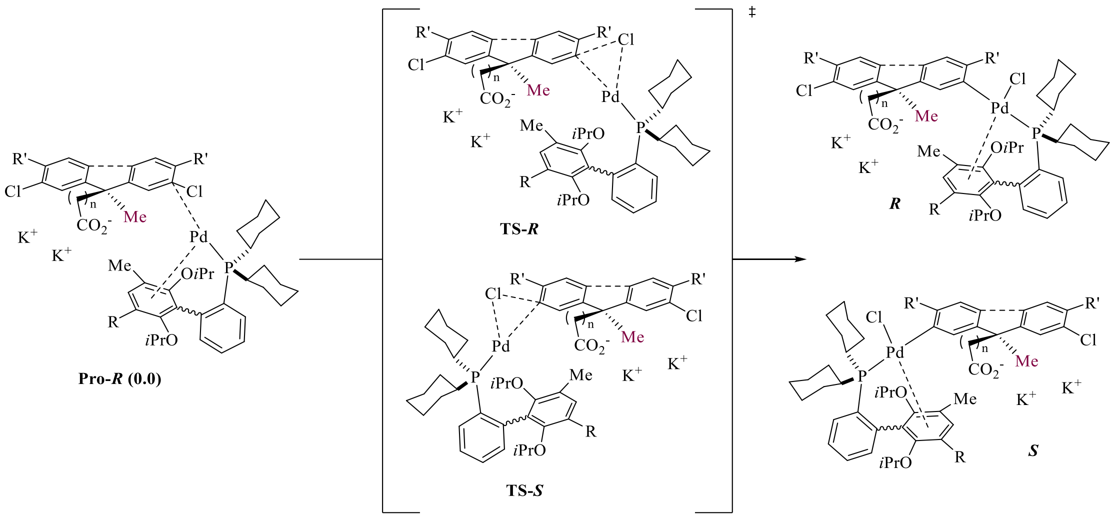

	@2K		@1K	
x	**L1**	**L11**	**L1**	**L11**
***R*-80**	7.0/-22.4	-	11.3/-17.5	-
***S*-80**	10.2/-17.6	-	12.5/-9.9	-
***R*-3**	4.4/-27.9	-	3.5/-23.5	-
***S*-3**	5.3/-23.2	-	5.4/-20.6	-
***R*-4**	-	2.2/-33.3	-	6.9/-28.2
***S*-4**	-	-0.7[a]/-33.9	-	2.6/-28.2

[a]The pro-*S* starting complex is more stable than the pro-*R* one by 9.9 kcal/mol, which means that the barrier in this case, is 9.2 kcal/mol.

The free energy difference $\Delta\Delta G^{\ddagger}_{298}$ between the corresponding diastereomeric oxidative addition transition states is summarized in **Table S7**. A positive value means that the *R* isomer is expected to be the major product. With compound **80** and ligand **L1**, the *R* isomer should dominate based on the computational results, which is the case experimentally. However, since the experimental *er* is 89:11 (**Figure S2**), the $\Delta\Delta G^{\ddagger}_{298}$ should be around 1.3 kcal/mol. In this case, the computational results of @1K matches the experimental *er* better than that of @2K.

For compound **3** and ligand **L1**, the *R* isomer is the experimentally major stereoisomer and the *er* of 58:42 suggests a $\Delta\Delta G^{\ddagger}_{298}$ of about 0.3 kcal/mol, versus 0.9 kcal/mol for the @2K value. On the other hand, the @1K value of 1.9 kcal/mol overestimates the actual *er*. Finally, based on a $\Delta\Delta G^{\ddagger}_{298}$of

-3.0 kcal/mol with @2K and -4.3 kcal/mol with @1K respectively, a virtually fully *S*-selective transformation is expected from compounds **4** and **L11**. There is indeed a strong preference for the *S* isomer in this case (*er* 97.5:2.5, estimated $\Delta\Delta G^{\ddagger}_{298}$ -2.3 kcal/mol).

Table S7. $\Delta\Delta G^{\ddagger}_{298}$ [TS-x-*S*/L – TS-x-*R*/L] (kcal/mol)

	@2K		@1K	
x	**L1**	**L11**	**L1**	**L11**
**80**	3.3	-	1.3	-
**3**	0.9	-	1.9	-
**4**	-	-3.0	-	-4.3

Thus, the free energy difference $\Delta\Delta G^{\ddagger}_{298}$ does not always coincide with the experimental *R/S* ratio. This might be due to the difficulty to take subtle effects into account, such as the presence of water and additional cations and anions in the reaction medium. Nevertheless, there is a good trend throughout the series, which encouraged us to use the computed structures to rationalize the stereoselectivity (**Figure S4**).

Focusing on the 8 membered cycle formed by the 2 potassium ions connecting the phosphate and the carboxylate moieties, the striking difference between **TS-80-*R*/L1@2K** and **TS-80-*S*/L1@2K** is its conformation (**Figure S4**). While the 8 atoms are virtually coplanar in the former, the cycle is twisted in the latter. We believe this distortion is the result of the steric hindrance brought about by one of the cyclohexyl substituents of the phosphine (highlighted in pink color), which is placed closer to the 8-membered ring in the less stable *S* transition state. Although it is difficult to quantify this steric hindrance, we hypothesized that it could influence the distance between Pd and the centroid of the electron-rich aromatic ring (Pd–Ar^{centroid} i.e., $\boldsymbol{d}_{\mathbf{Pd\text{-}Ar}}$) of the transition states. The electron-rich aromatic ring serves as a labile lignad, and its coordination to Pd is known to stabilizing the transition state of oxidative addition.[19] Within each pair of diastereomeric transition states, the one with shorter distance $\boldsymbol{d}_{\mathbf{Pd\text{-}Ar}}$ is favored computationally, which is also in agreement with the experimental results. For example, distance $\boldsymbol{d}_{\mathbf{Pd\text{-}Ar}}$ is shorter in **TS-80-*R*/L1@2K** compared to **TS-80-*S*/L1@2K** (3.17 vs 3.30 Å, respectively), and the R isomer is formed in excess. The results of acyclic compound **4** are most decipherable. In the @2K series, as for the *R* isomer, various structural constraints imposed by the geometry of the ligand's amide move the metal center

away from the Ar^{centroid} ($\boldsymbol{d}_{\mathbf{Pd\text{-}Ar}}$ 3.98 Å). By contrast, in the case of *S* isomer, the Pd–Ar^{centroid} distance is shortened to 2.88 Å. The short distance can be attributed to the participation of the amide carbonyl in the coordination of one potassium ion. Unlike the former structures, the carboxylate moiety of the substrates engages only one oxygen in the complexation, which is compensated for by the amide. A similar effect is observed in the @1K series.

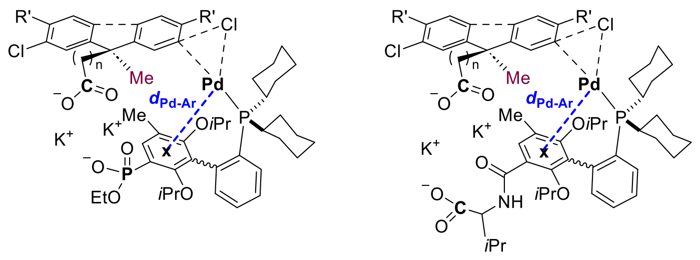

Illustration of Pd–Ar^{centroid} i.e., $\boldsymbol{d}_{\mathbf{Pd\text{-}Ar}}$

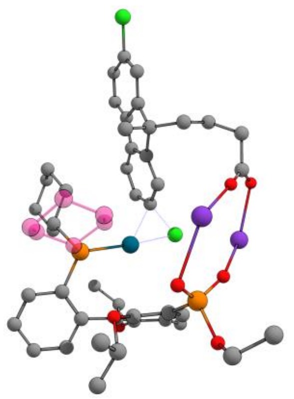

TS-80-*R*/L1@2K ($\boldsymbol{d}_{\mathbf{Pd\text{-}Ar}}$ 3.17 Å)

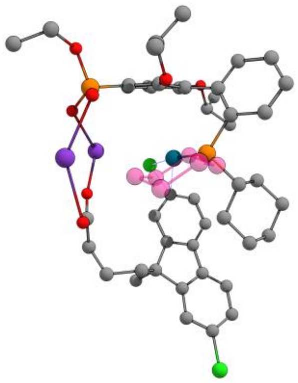

TS-80-*S*/L1@2K ($\boldsymbol{d}_{\mathbf{Pd\text{-}Ar}}$ 3.30 Å)

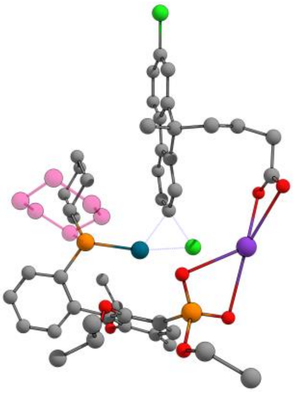

TS-80-*R*/L1@1K ($\boldsymbol{d}_{\mathbf{Pd\text{-}Ar}}$ 3.05 Å)

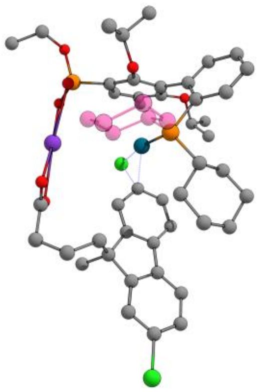

TS-80-*S*/L1@1K ($\boldsymbol{d}_{\mathbf{Pd\text{-}Ar}}$ 3.00 Å)

Figure S4. The computed oxidative addition transition states with $\boldsymbol{d}_{\mathbf{Pd\text{-}Ar}}$ distances.

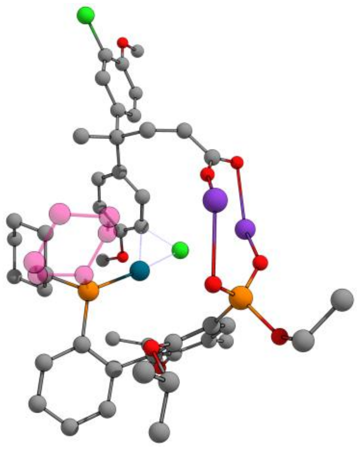

TS-3-*R*/L1@2K (***d*$_{\mathbf{Pd\text{-}Ar}}$** 3.22 Å)

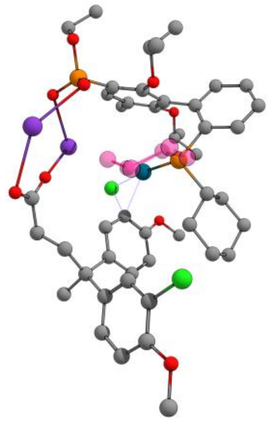

TS-3-*S*/L1@2K (***d*$_{\mathbf{Pd\text{-}Ar}}$** 3.48 Å)

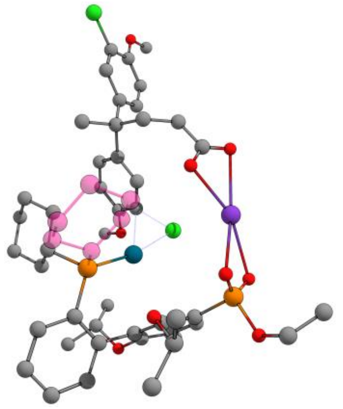

TS-3-*R*/L1@1K (***d*$_{\mathbf{Pd\text{-}Ar}}$** 3.21 Å)

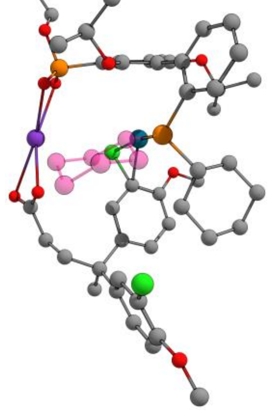

TS-3-*S*/L1@1K (***d*$_{\mathbf{Pd\text{-}Ar}}$** 3.23 Å)

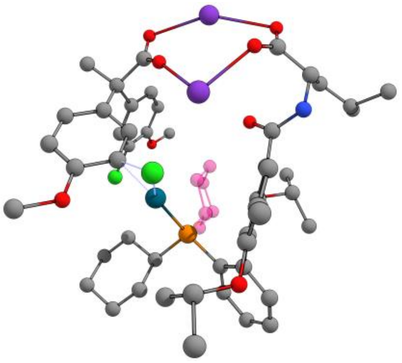

TS-4-*R*/L11@2K (***d*$_{\mathbf{Pd\text{-}Ar}}$** 3.98 Å)

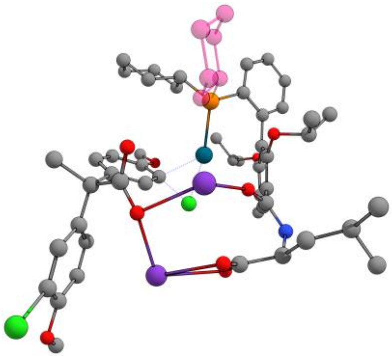

TS-4-*S*/L11@2K (***d*$_{\mathbf{Pd\text{-}Ar}}$** 2.88 Å)

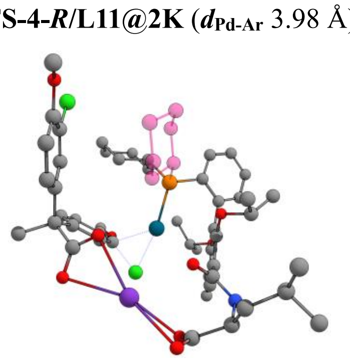

TS-4-*R*/L11@1K (***d*$_{\mathbf{Pd\text{-}Ar}}$** 3.01 Å)

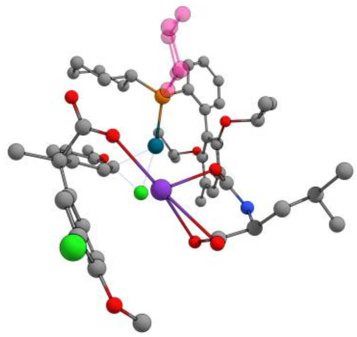

TS-4-*S*/L11@1K (***d*$_{\mathbf{Pd\text{-}Ar}}$** 2.93 Å)

Figure S4 (continued). The computed oxidative addition transition states with ***d*$_{\mathbf{Pd\text{-}Ar}}$** distances.

Table S8. Coordinates (x,y,z), thermal correction to the Gibbs free energy, SCRF energies (Hartree) and imaginary frequencies (cm^{-1}) of the computed species.

TS-80-*R*/L1@2K<br>Thermal correction to Gibbs Free Energy= 0.941008<br>E(RM11L) = -5440.15559193<br>Frequency -102.0294				TS-80-*S*/L1@2K<br>Thermal correction to Gibbs Free Energy= 0.945710<br>E(RM11L) = -5440.15508632<br>Frequency -177.7628			
Cl	-0.150918	0.666703	-2.950212	Cl	0.132179	0.310965	-3.489176
C	-1.455011	-0.198637	-1.90785	C	1.665554	0.491778	-2.248244
Pd	0.523593	-0.625933	-0.964878	Pd	-0.146769	0.7481	-1.080255
C	3.636603	-0.855957	-0.516783	C	-3.031503	1.820812	-0.195104
C	3.785614	-0.736162	-1.902913	C	-3.099407	2.14296	-1.552783
C	3.454959	0.30889	0.240049	C	-3.465751	0.556263	0.227267
C	3.738615	-2.173567	0.155166	C	-2.612723	2.814169	0.818744
C	3.669965	0.502996	-2.537212	C	-3.499714	1.191504	-2.496163
O	4.162993	-1.817809	-2.613651	O	-2.924282	3.425643	-1.925017
C	3.357484	1.600056	-1.753521	C	-3.793983	-0.082941	-2.038932
C	3.89116	0.648159	-4.001677	C	-3.648015	1.55036	-3.931926
C	3.244097	1.545669	-0.369378	C	-3.790048	-0.44109	-0.691992
H	3.22961	2.574606	-2.243773	H	-4.113891	-0.839162	-2.770968
O	3.44875	0.173797	1.576658	O	-3.515768	0.350678	1.551228
C	2.672433	-2.76049	0.867629	C	-1.454392	2.656108	1.604652
C	4.947736	-2.857742	0.070813	C	-3.426662	3.924157	1.018549
C	2.887235	-3.992516	1.483776	C	-1.210376	3.601517	2.600787
P	1.007575	-1.99653	0.885271	P	-0.210358	1.350266	1.236105
C	4.100184	-4.650387	1.401877	C	-2.045692	4.683173	2.80847
H	2.071007	-4.468618	2.039087	H	-0.321822	3.509034	3.233574
C	5.134989	-4.081679	0.68248	C	-3.152283	4.857092	1.999009
H	4.232503	-5.617036	1.898566	H	-1.817835	5.401389	3.60262
H	6.098714	-4.593758	0.597343	H	-3.811685	5.719936	2.1358

H	5.764401	-2.400072	-0.497387	H	-4.310794	4.040398	0.382809
C	-0.168679	-3.421691	0.976401	C	1.368519	2.118847	1.813384
C	0.91274	-1.203922	2.563484	C	-0.572388	0.039855	2.500365
C	4.541181	0.781148	2.254387	C	-4.804408	0.128258	2.110493
H	4.706644	1.78907	1.823661	H	-5.352581	-0.587569	1.467652
C	4.119515	0.912378	3.68632	C	-4.554649	-0.487203	3.453209
C	5.780914	-0.054064	2.07646	C	-5.560898	1.427108	2.185797
C	3.197487	-2.404566	-3.473609	C	-1.737544	3.777504	-2.624647
H	2.528857	-1.606449	-3.862976	H	-1.34356	2.878024	-3.143512
C	3.966136	-3.032254	-4.59917	C	-2.126506	4.829129	-3.623305
C	2.372962	-3.395513	-2.702161	C	-0.710292	4.271627	-1.642629
C	-1.591721	-2.880258	1.043604	C	2.530049	1.14834	1.650604
C	-0.041872	-4.333234	-0.233381	C	1.694379	3.412241	1.083956
H	0.01499	-4.025742	1.891057	H	1.283852	2.350357	2.896302
C	-2.630813	-3.979818	1.047829	C	3.824292	1.746555	2.162537
H	-1.75409	-2.219664	0.169646	H	2.626916	0.891682	0.578476
H	-1.731855	-2.24161	1.932644	H	2.334558	0.195814	2.173013
C	-1.054982	-5.455509	-0.187743	C	2.962166	4.028741	1.6349
H	-0.218381	-3.724512	-1.14547	H	1.830903	3.186608	0.006337
H	0.983004	-4.736	-0.324378	H	0.860196	4.133062	1.143498
C	-2.463518	-4.911242	-0.129771	C	4.129217	3.076453	1.513303
H	-3.64206	-3.530811	1.04888	H	4.656724	1.036602	2.002519
H	-2.54994	-4.560917	1.988832	H	3.746545	1.886323	3.260167
H	-0.926281	-6.11731	-1.062166	H	3.1792	4.980794	1.118949
H	-0.865611	-6.086925	0.703899	H	2.804218	4.289599	2.701343
H	-3.202714	-5.730199	-0.088683	H	5.046612	3.515856	1.943087
H	-2.6704	-4.351321	-1.064236	H	4.341287	2.912576	0.439456
C	0.039938	0.039559	2.488265	C	-0.124475	-1.34961	2.072927
C	0.539587	-2.097131	3.732492	C	-0.120441	0.363818	3.914375

H	1.945692	-0.855165	2.740696	H	-1.67764	0.012802	2.514121
C	-0.009669	0.786131	3.801446	C	-0.589148	-2.390219	3.070941
H	-0.98405	-0.241616	2.177437	H	0.977376	-1.413472	1.974681
H	0.443166	0.684214	1.683533	H	-0.516161	-1.566128	1.058114
C	0.54943	-1.30534	5.024367	C	-0.615964	-0.685256	4.886958
H	-0.468806	-2.535039	3.600229	H	0.985887	0.400751	3.973184
H	1.240746	-2.949525	3.802057	H	-0.484508	1.362578	4.219066
C	-0.392513	-0.124986	4.944918	C	-0.144561	-2.059758	4.477843
H	-0.730047	1.629256	3.740382	H	-0.194306	-3.37917	2.777436
H	0.980636	1.241908	3.994728	H	-1.698991	-2.43753	3.043754
H	0.287611	-1.95751	5.876016	H	-0.28384	-0.44253	5.911912
H	1.576946	-0.936985	5.220517	H	-1.724304	-0.669582	4.90767
H	-0.406823	0.429301	5.899439	H	-0.505534	-2.825028	5.187211
H	-1.426532	-0.495448	4.786734	H	0.962464	-2.095263	4.526247
H	3.891945	-0.079375	4.114528	H	-3.956984	0.197605	4.079316
H	4.926261	1.357992	4.287825	H	-5.503551	-0.680512	3.975799
H	3.228404	1.552023	3.754239	H	-4.013129	-1.436531	3.333465
H	6.634767	0.401623	2.601779	H	-6.561858	1.26597	2.615755
H	5.626091	-1.069833	2.476168	H	-5.024161	2.156398	2.814549
H	6.055297	-0.146329	1.013332	H	-5.697185	1.872921	1.187213
H	4.556067	-2.285961	-5.152045	H	-2.865816	4.451509	-4.344874
H	4.662371	-3.790019	-4.20614	H	-2.569687	5.696009	-3.107779
H	3.287041	-3.528949	-5.307757	H	-1.247628	5.180696	-4.183927
H	1.649472	-3.90538	-3.359165	H	0.196838	4.620973	-2.163882
H	3.018105	-4.155157	-2.228884	H	-1.114251	5.107297	-1.047028
H	1.803094	-2.884571	-1.91099	H	-0.411569	3.470595	-0.949857
H	2.979814	0.453016	-4.595144	H	-2.679326	1.653351	-4.453127
H	4.220195	1.669548	-4.247233	H	-4.225562	0.783001	-4.469482
H	4.663822	-0.048581	-4.362016	H	-4.1748	2.510752	-4.049994

C	-2.134337	0.572584	-0.961063	C	2.349053	1.69463	-2.405847
C	-3.380265	0.143711	-0.564392	C	3.668458	1.75871	-2.003082
C	-4.284967	0.742636	0.489863	C	4.290183	0.618734	-1.512878
C	-5.568474	-0.007376	0.225159	C	5.569718	0.450194	-0.862362
C	-2.050401	-1.299816	-2.527787	C	6.577853	1.35692	-0.570279
C	-3.323952	-1.679486	-2.151922	C	7.678746	0.936679	0.153642
C	-3.998081	-0.953195	-1.179135	C	7.761622	-0.383923	0.56778
C	-5.35703	-1.04321	-0.691139	C	6.771086	-1.309571	0.27056
C	-6.389972	-1.911679	-1.009298	C	5.673103	-0.877161	-0.438621
C	-7.628636	-1.736111	-0.419696	C	4.511752	-1.705209	-0.949211
C	-7.819896	-0.699013	0.480588	C	3.614849	-0.610976	-1.494212
C	-6.79546	0.172759	0.82217	C	2.298374	-0.698736	-1.890335
C	-3.797397	0.35286	1.882998	C	5.078427	-2.516203	-2.118579
H	-3.652393	-0.73643	1.977255	H	5.559682	-1.841315	-2.844014
H	-2.837866	0.849879	2.111178	H	5.846444	-3.226548	-1.77135
Cl	-9.373748	-0.489989	1.196627	H	6.873674	-2.346726	0.607081
H	-1.690789	1.499227	-0.579182	H	8.483249	1.631335	0.407287
H	-1.506991	-1.836129	-3.310228	H	6.511955	2.401729	-0.894127
H	-3.797333	-2.534714	-2.648562	H	4.198113	2.717885	-2.047536
H	-6.243138	-2.727815	-1.725048	H	1.822693	2.57551	-2.784842
H	-8.462621	-2.401385	-0.656752	H	1.723997	-1.630152	-1.833306
H	-6.978929	0.97763	1.541725	H	4.304667	-3.085792	-2.653685
H	-4.526198	0.665649	2.649894	P	-4.101599	-2.166631	-0.269797
P	2.70266	3.032353	0.487423	O	-3.451475	-2.974998	-1.342451
O	1.759815	3.730576	-0.436519	O	-3.729532	-2.501955	1.122554
O	2.190545	2.774485	1.853615	O	-5.698661	-2.201739	-0.439553
O	4.090264	3.84013	0.562127	C	-6.438133	-3.067592	0.376393
C	4.32631	4.664325	1.6694	H	-7.480293	-2.709728	0.320006
H	5.414223	4.848769	1.675092	H	-6.101915	-2.96974	1.427227

H	4.069077	4.123686	2.60133	C	-6.345117	-4.494588	-0.081487
C	3.572705	5.960121	1.581652	H	-5.316259	-4.875818	0.017135
H	2.485261	5.788618	1.622067	H	-6.631445	-4.58627	-1.140136
H	3.792023	6.477784	0.63548	H	-7.005604	-5.142619	0.516259
H	3.841381	6.629114	2.414517	C	3.856318	-2.580766	0.146357
C	-4.363475	2.259137	0.425442	H	2.944906	-2.067819	0.514994
H	-3.367886	2.646465	0.712174	H	4.53662	-2.596493	1.016661
H	-5.046976	2.61541	1.221563	C	3.517223	-4.020806	-0.1924
C	-4.74715	2.877762	-0.901523	H	3.265272	-4.533234	0.751866
H	-5.846884	2.92036	-0.996049	H	4.41631	-4.531979	-0.581512
H	-4.403642	2.2392	-1.737924	C	2.349429	-4.237228	-1.126409
C	-4.147034	4.265455	-1.068276	H	2.420874	-3.652624	-2.05852
H	-4.660101	4.802627	-1.880508	H	2.350439	-5.294122	-1.453494
H	-4.289968	4.841207	-0.134265	C	0.983599	-4.005031	-0.493603
C	-2.66464	4.189415	-1.41568	O	0.090914	-3.534302	-1.239831
O	-2.357208	4.287379	-2.603854	O	0.827823	-4.347504	0.679685
O	-1.849701	3.990102	-0.473835	K	-1.7948	-4.164138	0.605984
K	-0.280046	3.440616	1.384049	K	-1.444199	-2.279447	-2.688054
K	0.149348	3.837891	-2.357401	Cl	9.13442	-0.888736	1.480602
**TS-80-*R*/L1@1K**<br>Thermal correction to Gibbs Free Energy=<br>0.944057<br>E(RM11L) = -4840.26184366<br>Frequency -135.7712				**TS-80-*S*/L1@1K**<br>Thermal correction to Gibbs Free Energy=<br>0.946060<br>E(RM11L) = -4840.26184366<br>Frequency -174.0916			
Cl	0.1056	1.370053	-2.516902	Cl	0.306981	-0.146897	-3.278322
C	-1.156659	0.183885	-1.672772	C	-1.232247	-0.564277	-2.036569
Pd	0.826652	-0.180242	-0.798276	Pd	0.602019	-0.579609	-0.927729
C	3.793843	-0.580602	-0.411152	C	3.303903	-1.512658	-0.231301
C	4.224739	-0.444427	-1.73986	C	3.448119	-1.994339	-1.538362

C	3.358039	0.570515	0.260268	C	3.516787	-0.144994	0.003538
C	3.875121	-1.890659	0.275829	C	3.059581	-2.423803	0.910354
C	4.193337	0.783687	-2.393463	C	3.716021	-1.130943	-2.598849
O	4.766548	-1.517179	-2.36702	O	3.417268	-3.330822	-1.757094
C	3.666479	1.870062	-1.711014	C	3.792917	0.225759	-2.327563
C	4.716321	0.940453	-3.779228	C	3.889968	-1.643792	-3.984606
C	3.239256	1.80066	-0.394199	C	3.708766	0.754811	-1.046304
H	3.563974	2.836629	-2.219333	H	3.90752	0.932079	-3.158761
O	3.034561	0.450161	1.564032	O	3.49067	0.252361	1.288068
C	2.783233	-2.531052	0.897243	C	1.933568	-2.329907	1.752788
C	5.118387	-2.523885	0.29487	C	4.014804	-3.403292	1.170938
C	3.00211	-3.76576	1.509248	C	1.848013	-3.199415	2.839499
P	1.091825	-1.82542	0.843012	P	0.548522	-1.188916	1.365777
C	4.243286	-4.37216	1.524964	C	2.816684	-4.151963	3.095264
H	2.162188	-4.286595	1.982301	H	0.980159	-3.145706	3.506889
C	5.310397	-3.74397	0.908975	C	3.902502	-4.262153	2.245953
H	4.3738	-5.341617	2.017968	H	2.714602	-4.817632	3.959102
H	6.302774	-4.207371	0.905904	H	4.673159	-5.019663	2.424094
H	5.960295	-2.023133	-0.193898	H	4.877123	-3.474916	0.499532
C	0.000357	-3.308496	0.645656	C	-0.947432	-2.111718	1.941285
C	0.796614	-1.291261	2.606021	C	0.725629	0.173809	2.622206
C	3.98275	1.003885	2.472323	C	4.718603	0.759598	1.786904
H	4.474753	1.859455	1.97479	H	5.189007	1.381554	1.000925
C	3.204319	1.50653	3.653365	C	4.377974	1.61991	2.965591
C	5.006368	-0.037289	2.84274	C	5.62827	-0.390379	2.135225
C	3.96895	-2.121794	-3.366381	C	2.248213	-3.849436	-2.367524
H	3.506724	-1.328046	-3.991774	H	1.803705	-3.068833	-3.022679
C	4.918007	-2.935344	-4.197606	C	2.687697	-5.027803	-3.188917
C	2.878887	-2.945871	-2.742618	C	1.248887	-4.223759	-1.308157

C	-1.452225	-2.850799	0.635919	C	-2.185185	-1.266625	1.673355
C	0.298156	-4.055529	-0.643163	C	-1.124084	-3.469007	1.281863
H	0.138306	-4.016972	1.49145	H	-0.878459	-2.276555	3.038628
C	-2.418458	-3.990872	0.403347	C	-3.443692	-1.943774	2.170563
H	-1.571918	-2.095476	-0.160968	H	-2.257377	-1.091143	0.584853
H	-1.715534	-2.334194	1.574448	H	-2.097509	-0.264613	2.127657
C	-0.648957	-5.218706	-0.840389	C	-2.360004	-4.162661	1.815123
H	0.187029	-3.348297	-1.491661	H	-1.230739	-3.323314	0.188214
H	1.346981	-4.402607	-0.662623	H	-0.232487	-4.105464	1.426181
C	-2.086811	-4.753678	-0.857976	C	-3.602121	-3.329769	1.589104
H	-3.451026	-3.596229	0.360929	H	-4.327045	-1.32277	1.930161
H	-2.38683	-4.685682	1.268279	H	-3.406288	-2.013511	3.277696
H	-0.399263	-5.757603	-1.772329	H	-2.468606	-5.159044	1.349354
H	-0.508953	-5.949516	-0.017226	H	-2.231929	-4.346468	2.902104
H	-2.776238	-5.606311	-0.994069	H	-4.493247	-3.828877	2.010398
H	-2.238745	-4.087546	-1.730764	H	-3.783464	-3.242778	0.500056
C	-0.122814	-0.076089	2.610264	C	0.269226	1.502424	2.036361
C	0.343057	-2.370812	3.575815	C	0.135506	-0.079586	3.998613
H	1.795914	-0.958047	2.944624	H	1.820725	0.248679	2.752744
C	-0.432369	0.423596	4.00415	C	0.488662	2.655977	2.986842
H	-1.07256	-0.33825	2.105732	H	-0.801007	1.466459	1.747013
H	0.327256	0.730071	2.000639	H	0.840851	1.671614	1.106656
C	0.105748	-1.797878	4.957688	C	0.425635	1.087895	4.920418
H	-0.600381	-2.842493	3.24051	H	-0.962974	-0.213357	3.945624
H	1.091332	-3.183439	3.628126	H	0.541824	-1.013739	4.431283
C	-0.915205	-0.68474	4.909307	C	-0.11194	2.378368	4.345008
H	-1.173212	1.241448	3.949571	H	0.062822	3.572173	2.545951
H	0.47687	0.874098	4.447533	H	1.577083	2.826299	3.096073
H	-0.209075	-2.598838	5.651606	H	0.004322	0.891252	5.923759

H	1.05938	-1.397489	5.35998	H	1.523056	1.179087	5.057268
H	-1.128435	-0.303388	5.924177	H	0.077252	3.219894	5.035417
H	-1.874747	-1.087914	4.522193	H	-1.214401	2.301216	4.251325
H	2.813904	0.67089	4.260079	H	3.873561	1.027667	3.749644
H	3.846338	2.118262	4.306724	H	5.292511	2.047571	3.405877
H	2.355952	2.117385	3.308142	H	3.716866	2.435592	2.640916
H	5.686131	0.349155	3.620158	H	6.585271	-0.023159	2.540135
H	4.517144	-0.946791	3.233051	H	5.156732	-1.04373	2.889015
H	5.619956	-0.333755	1.97813	H	5.854129	-1.00666	1.249723
H	5.703792	-2.302479	-4.638076	H	3.409147	-4.731018	-3.964985
H	5.408314	-3.698124	-3.571164	H	3.173012	-5.778479	-2.544027
H	4.387405	-3.449842	-5.012603	H	1.827623	-5.506661	-3.681096
H	2.262783	-3.435591	-3.51443	H	0.356749	-4.697607	-1.751675
H	3.307588	-3.723713	-2.087547	H	1.696458	-4.923056	-0.581353
H	2.209293	-2.313302	-2.135681	H	0.913534	-3.328991	-0.762001
H	3.926956	0.842138	-4.544668	H	2.925952	-1.808063	-4.498765
H	5.159178	1.939413	-3.914014	H	4.454867	-0.923729	-4.595402
H	5.491051	0.191499	-4.007653	H	4.433295	-2.602376	-3.996158
C	-1.983417	0.744529	-0.691359	C	-1.784156	-1.837824	-2.221981
C	-3.238736	0.210185	-0.523979	C	-3.120512	-2.048907	-1.944095
C	-4.295782	0.618886	0.479058	C	-3.907175	-0.984257	-1.518382
C	-5.440071	-0.2804	0.07714	C	-5.273786	-0.913771	-1.054983
C	-1.632821	-0.811026	-2.538327	C	-5.556433	0.414534	-0.701888
C	-2.903029	-1.318665	-2.366642	C	-6.795515	0.761499	-0.210315
C	-3.711008	-0.814671	-1.354999	C	-7.74562	-0.239331	-0.060992
C	-5.068042	-1.125786	-0.975344	C	-4.359381	1.307765	-0.94318
C	-5.968443	-2.061576	-1.465132	C	-3.35394	0.297428	-1.441045
C	-7.233997	-2.143287	-0.911315	C	-7.483706	-1.557532	-0.393608
C	-7.583146	-1.291148	0.123316	C	-6.239229	-1.897458	-0.895506

C	-6.696928	-0.353254	0.633729	C	-2.033497	0.537793	-1.740318
C	-3.863135	0.301892	1.903119	C	-4.670695	2.312631	-2.043348
H	-3.57714	-0.758313	2.016233	H	-5.001002	1.782608	-2.951809
H	-3.001142	0.925817	2.194858	H	-5.487228	2.990163	-1.740159
Cl	-9.172041	-1.408388	0.80367	H	-7.040029	1.792689	0.064355
H	-1.606201	1.555199	-0.05629	H	-8.261748	-2.313355	-0.257214
H	-0.985183	-1.169508	-3.34336	H	-6.029748	-2.94064	-1.159977
H	-3.265955	-2.104973	-3.040669	H	-3.536665	-3.06028	-2.039308
H	-5.692571	-2.734563	-2.284934	H	-1.139244	-2.660623	-2.547271
H	-7.963431	-2.869771	-1.279256	H	-1.623003	1.554472	-1.700419
H	-7.005309	0.305383	1.452589	H	-3.783118	2.919763	-2.284114
H	-4.680134	0.510618	2.614925	P	3.648601	2.556808	-0.889667
P	2.322058	3.222316	0.242156	O	3.068536	3.062461	-2.151715
O	2.202306	4.20932	-0.856185	O	3.018592	2.965476	0.387606
O	1.075128	2.787388	0.919294	O	5.239818	2.879548	-0.823805
O	3.384037	3.75098	1.350525	C	5.645386	3.965447	-0.050522
C	2.948737	4.769509	2.198979	H	6.737988	3.853069	0.079637
H	3.46562	4.618065	3.166828	H	5.179142	3.914602	0.95454
H	1.863474	4.662558	2.40043	C	5.329827	5.282465	-0.70338
C	3.266546	6.127222	1.637804	H	4.241624	5.424965	-0.78844
H	2.760951	6.270973	0.671557	H	5.750597	5.324615	-1.71983
H	4.34932	6.229103	1.463611	H	5.740548	6.12117	-0.117015
H	2.95151	6.925043	2.330594	C	-3.82664	1.94299	0.360619
C	-4.623464	2.10609	0.37449	H	-2.722319	1.962061	0.311558
H	-3.70826	2.666418	0.652957	H	-4.055738	1.236697	1.182403
H	-5.360062	2.363584	1.16275	C	-4.276299	3.3346	0.769263
C	-5.113789	2.584837	-0.974971	H	-3.987146	3.445931	1.82963
H	-6.178198	2.31187	-1.106059	H	-5.381203	3.420734	0.754544
H	-4.550055	2.078775	-1.780039	C	-3.644935	4.487098	-0.004908

C	-4.888501	4.072253	-1.135651	H	-4.169995	4.67926	-0.953521
H	-5.341254	4.412568	-2.084549	H	-3.733287	5.401755	0.607773
H	-5.363511	4.640338	-0.314908	C	-2.162928	4.222506	-0.290299
C	-3.380664	4.361516	-1.172775	O	-1.856804	3.967482	-1.470849
O	-2.748451	3.752675	-2.052257	O	-1.397557	4.232888	0.684629
O	-2.914586	5.119927	-0.307442	Cl	-9.308867	0.173221	0.561672
K	-0.488287	4.210632	-0.842378	K	0.690651	3.575319	-0.895747
**TS-3-*R*/L1@2K**<br>Thermal correction to Gibbs Free Energy= 0.997051<br>E(RM11L) = -5631.06689735<br>Frequency -111.7766				**TS-3-*S*/L1@2K**<br>Thermal correction to Gibbs Free Energy= 1.001060<br>E(RM11L) = -5631.07236414<br>Frequency -116.9691			
Cl	-0.336887	-0.356244	-2.911032	Cl	-0.422169	0.783873	-3.512216
C	-1.429207	-1.044572	-1.551232	C	1.217674	0.934692	-2.593085
Pd	0.685656	-0.827702	-0.725633	Pd	-0.516872	1.025439	-1.077916
C	3.825596	-0.734437	-0.639705	C	-3.44277	1.186431	0.508534
C	3.71397	-0.951731	-2.015562	C	-3.881575	1.611051	-0.745662
C	3.773891	0.579841	-0.156481	C	-3.486722	-0.179469	0.824969
C	4.08362	-1.837348	0.313807	C	-3.026553	2.131024	1.568395
C	3.465637	0.109095	-2.891236	C	-4.286717	0.682841	-1.710464
O	3.97639	-2.175774	-2.513492	O	-4.045619	2.927655	-0.976353
C	3.298481	1.370637	-2.346371	C	-4.205398	-0.661358	-1.386023
C	3.409065	-0.110841	-4.361024	C	-4.825107	1.127146	-3.02408
C	3.4457	1.64765	-0.990047	C	-3.807471	-1.139999	-0.136612
H	3.094199	2.211364	-3.024569	H	-4.507894	-1.397287	-2.146096
O	4.004686	0.743913	1.155596	O	-3.143677	-0.495439	2.077442
C	3.177828	-2.19001	1.335842	C	-1.719419	2.151135	2.094076
C	5.291189	-2.519057	0.210698	C	-3.996074	2.96625	2.110636
C	3.565624	-3.179555	2.238495	C	-1.477104	2.967859	3.197845

P	1.466903	-1.517135	1.359344	P	-0.334434	1.289222	1.241171
C	4.782345	-3.829171	2.139101	C	-2.460566	3.769007	3.749119
H	2.886258	-3.475189	3.045312	H	-0.478135	2.997648	3.645016
C	5.644105	-3.509237	1.107007	C	-3.72296	3.787182	3.187552
H	5.050017	-4.600451	2.868662	H	-2.229616	4.393636	4.618168
H	6.602027	-4.028375	1.001535	H	-4.505236	4.434161	3.596946
H	5.973877	-2.245456	-0.600826	H	-5.000944	2.946801	1.674908
C	0.478476	-2.910414	2.074967	C	1.109813	2.377915	1.63809
C	1.497396	-0.224549	2.689391	C	-0.056491	-0.278656	2.193521
C	5.165727	1.481097	1.508683	C	-4.107587	-1.153599	2.889669
H	5.22335	2.383981	0.869872	H	-4.571309	-1.977236	2.317327
C	4.961635	1.884679	2.937541	C	-3.321052	-1.722096	4.030961
C	6.393328	0.634645	1.302619	C	-5.161848	-0.16856	3.317799
C	2.880332	-2.956535	-2.979267	C	-3.124363	3.575063	-1.848999
H	2.042627	-2.280187	-3.253304	H	-2.651597	2.812219	-2.504369
C	3.36848	-3.707186	-4.184283	C	-3.914497	4.557075	-2.665115
C	2.426508	-3.867117	-1.874088	C	-2.056423	4.240589	-1.027515
C	-0.971696	-2.484359	2.25326	C	2.401416	1.769459	1.117093
C	0.529958	-4.142223	1.184651	C	0.936527	3.766184	1.040416
H	0.871581	-3.187126	3.076886	H	1.217384	2.481176	2.738808
C	-1.832162	-3.607142	2.791514	C	3.596996	2.651712	1.403774
H	-1.366548	-2.143036	1.275691	H	2.306803	1.599572	0.026967
H	-1.047173	-1.617226	2.930995	H	2.572605	0.780033	1.567928
C	-0.304438	-5.270795	1.748789	C	2.117159	4.655691	1.362338
H	0.140915	-3.861145	0.182725	H	0.844626	3.659547	-0.062301
H	1.570336	-4.477125	1.024859	H	-0.003355	4.23751	1.379624
C	-1.742901	-4.848468	1.935779	C	3.410676	4.051153	0.867627
H	-2.880037	-3.263551	2.87145	H	4.507148	2.188887	0.979909
H	-1.510027	-3.851711	3.824206	H	3.760756	2.695829	2.499508

H	-0.238807	-6.15583	1.090505	H	1.963047	5.661219	0.93075
H	0.117743	-5.586094	2.724489	H	2.175201	4.801766	2.459955
H	-2.341614	-5.667652	2.371264	H	4.271323	4.68984	1.133715
H	-2.187757	-4.632586	0.943887	H	3.389425	4.009866	-0.240017
C	0.427039	0.83521	2.465354	C	0.560345	-1.348037	1.301675
C	1.508253	-0.725806	4.122246	C	0.676589	-0.131356	3.516987
H	2.464333	0.282895	2.516377	H	-1.078981	-0.627893	2.425755
C	0.566827	1.965054	3.463506	C	0.729	-2.65958	2.035533
H	-0.589893	0.402298	2.544128	H	1.542621	-1.012491	0.914343
H	0.506967	1.200212	1.420668	H	-0.073282	-1.487266	0.402642
C	1.661146	0.429872	5.089686	C	0.812234	-1.469928	4.214151
H	0.570104	-1.265011	4.361888	H	1.692912	0.283814	3.370046
H	2.329915	-1.45165	4.267271	H	0.140209	0.585542	4.167819
C	0.569721	1.455287	4.887544	C	1.499441	-2.480197	3.323632
H	-0.263245	2.690982	3.338487	H	1.236016	-3.393348	1.376492
H	1.514622	2.50656	3.261973	H	-0.280127	-3.058791	2.269459
H	1.662195	0.062835	6.131118	H	1.360936	-1.347117	5.164701
H	2.646577	0.911845	4.929526	H	-0.194989	-1.847543	4.483571
H	0.682407	2.291234	5.599872	H	1.616104	-3.44526	3.84713
H	-0.412551	0.991175	5.109345	H	2.526764	-2.128933	3.093566
H	4.840795	0.988412	3.570784	H	-2.771246	-0.918014	4.551006
H	5.82907	2.448289	3.312816	H	-3.983618	-2.211162	4.760301
H	4.062926	2.513109	3.022218	H	-2.602529	-2.463744	3.651273
H	7.301319	1.19751	1.57026	H	-5.92411	-0.663776	3.93979
H	6.349861	-0.274209	1.925304	H	-4.718329	0.65723	3.898837
H	6.494371	0.321519	0.250863	H	-5.67877	0.270162	2.44721
H	3.684616	-3.027229	-4.989071	H	-4.676059	4.060525	-3.284122
H	4.231234	-4.337837	-3.916098	H	-4.430329	5.270238	-2.002428
H	2.57865	-4.363376	-4.579981	H	-3.252535	5.131409	-3.330393

H	1.620095	-4.535862	-2.218407	H	-1.323071	4.750174	-1.675858
H	3.265857	-4.482043	-1.507973	H	-2.499648	4.979161	-0.338838
H	2.032008	-3.278883	-1.034025	H	-1.50756	3.497312	-0.432879
H	2.477507	-0.6083	-4.686162	H	-4.045699	1.522594	-3.700106
H	3.472641	0.845511	-4.902402	H	-5.316575	0.293315	-3.548398
H	4.241454	-0.746277	-4.703269	H	-5.56869	1.93062	-2.900094
C	-2.200628	-0.142818	-0.834331	C	1.834055	2.181783	-2.727085
C	-3.359604	-0.557223	-0.19344	C	3.207365	2.23393	-2.555404
C	-4.164869	0.376862	0.697507	C	3.932247	1.080768	-2.293811
C	-5.640558	0.337997	0.319641	C	3.322486	-0.164407	-2.185906
C	-1.81907	-2.376468	-1.715503	C	4.180366	-1.411877	-1.961432
C	-2.98044	-2.783685	-1.078222	C	5.209922	-1.118408	-0.876899
C	-3.734861	-1.889771	-0.33352	C	4.766933	-0.851309	0.416769
C	-6.064021	0.091162	-0.979302	C	5.648543	-0.612094	1.44425
C	-7.40085	0.13556	-1.335572	C	7.028641	-0.621318	1.2276
C	-8.374609	0.42794	-0.39331	C	7.476181	-0.885319	-0.055709
C	-7.957178	0.675985	0.913831	C	6.57845	-1.131229	-1.085217
C	-6.623645	0.636256	1.258924	C	1.942771	-0.224895	-2.363371
C	-3.951914	-0.097943	2.133207	C	4.862798	-1.750318	-3.278489
H	-4.379953	-1.098872	2.308417	H	5.503689	-0.92549	-3.62923
H	-2.871822	-0.152903	2.346036	H	5.482107	-2.659683	-3.192646
Cl	-9.140087	1.028029	2.109853	H	3.696365	-0.832106	0.638085
H	-1.835492	0.883169	-0.738859	H	8.547087	-0.9069	-0.270253
H	-3.314614	-3.82161	-1.161223	H	3.731073	3.192018	-2.618667
H	-7.680249	-0.065585	-2.372244	H	1.395896	-1.171026	-2.287345
H	-6.368689	0.848526	2.300644	H	4.117106	-1.926407	-4.066938
H	-4.388295	0.593852	2.872211	P	-3.600887	-2.924384	0.086753
P	3.069396	3.310588	-0.416903	O	-3.306056	-3.454435	-1.275044
O	1.971069	3.821552	-1.288665	O	-2.648879	-3.219438	1.173004

O	2.821612	3.378043	1.042781	O	-5.026681	-3.457721	0.590804
O	4.447945	4.055081	-0.775603	C	-6.158966	-3.30537	-0.21694
C	4.869955	5.111627	0.0404	H	-6.66629	-4.286214	-0.249177
H	5.941227	5.250495	-0.184732	H	-5.870529	-3.071135	-1.260549
H	4.782572	4.82058	1.105773	C	-7.070545	-2.257479	0.353332
C	4.107137	6.3765	-0.229844	H	-6.594705	-1.263056	0.343883
H	3.043867	6.261947	0.034646	H	-7.325766	-2.497887	1.39758
H	4.154616	6.643043	-1.29649	H	-8.008458	-2.191977	-0.220146
H	4.514753	7.214527	0.357654	C	3.34751	-2.613636	-1.477463
C	-3.668312	1.830458	0.628701	H	2.660936	-2.257166	-0.685477
H	-2.619377	1.862478	0.984011	H	4.023533	-3.317179	-0.962293
H	-4.246973	2.388782	1.387547	C	2.548469	-3.393102	-2.51718
C	-3.757395	2.522644	-0.719888	H	2.230106	-2.757702	-3.359477
H	-4.171124	1.857222	-1.495365	H	3.167439	-4.204783	-2.934676
H	-4.474272	3.360973	-0.687827	C	1.317905	-3.967481	-1.832907
C	-2.456594	3.077032	-1.292903	O	0.242573	-3.344009	-2.023312
O	-2.453102	3.386265	-2.483418	O	1.458658	-4.937055	-1.089107
O	-1.461067	3.152088	-0.527071	K	-0.976761	-4.759105	-0.099949
K	0.334391	4.040699	0.906328	K	-1.699208	-2.0007	-2.647345
K	0.092479	2.870108	-2.697122	Cl	5.061707	-0.297688	3.030589
H	-5.328582	-0.155984	-1.75173	H	6.988241	-1.342699	-2.076566
H	-4.661245	-2.243829	0.132535	H	5.017222	1.161862	-2.161794
O	-9.684913	0.488373	-0.62719	O	1.032982	3.219201	-2.986563
C	-10.094941	0.258367	-1.930627	C	1.628171	4.46901	-3.044976
H	-9.665676	0.99575	-2.633796	H	2.097509	4.746125	-2.081448
H	-9.830181	-0.759434	-2.272455	H	2.387184	4.529321	-3.84694
H	-11.186807	0.361295	-1.925258	H	0.820237	5.17815	-3.265219
O	-1.022322	-3.15151	-2.458966	O	7.802138	-0.374354	2.283462
C	-1.369402	-4.489185	-2.547155	C	9.171099	-0.396088	2.070055

H	-1.360108	-4.982213	-1.554727	H	9.517253	-1.38613	1.72004
H	-2.364003	-4.630361	-3.009521	H	9.486455	0.372955	1.34069
H	-0.611441	-4.958458	-3.18723	H	9.630848	-0.178373	3.041554
**TS-3-*R*/L1@1K** Thermal correction to Gibbs Free Energy= 0.998994 E(RM11L) = -5031.17701475 Frequency -130.4011				**TS-3-*S*/L1@1K** Thermal correction to Gibbs Free Energy= 1.000572 E(RM11L) = -5031.18160707 Frequency -137.2203			
Cl	-0.181007	0.902353	-2.480009	Cl	-0.878158	-1.688015	-2.601269
C	-1.262491	-0.335283	-1.474551	C	0.841794	-0.86905	-2.282914
Pd	0.885562	-0.403395	-0.75603	Pd	-0.74844	0.094207	-0.987882
P	1.454849	-1.535381	1.193051	P	-0.192676	1.525081	0.763034
C	3.1817	-2.124447	1.38887	C	-1.404872	2.793059	1.306807
C	4.197231	-1.6687	0.53046	C	-2.750748	2.72545	0.905612
C	4.006217	-0.62295	-0.50084	C	-3.31279	1.614474	0.103204
C	4.185098	-0.927243	-1.853239	C	-3.785884	1.848079	-1.191117
C	3.775568	0.705597	-0.126668	C	-3.463165	0.343824	0.667257
C	4.13133	0.058535	-2.830239	C	-4.378711	0.836287	-1.935595
O	4.475744	-2.199932	-2.221069	O	-3.71457	3.098972	-1.710404
C	3.868793	1.355129	-2.415321	C	-4.459304	-0.420702	-1.353681
C	4.311485	-0.272016	-4.270742	C	-4.864699	1.073753	-3.322952
C	3.686077	1.717068	-1.086893	C	-4.0217	-0.708237	-0.066488
H	3.753144	2.14434	-3.168317	H	-4.842528	-1.257683	-1.951688
O	3.594992	0.925678	1.188312	O	-2.983505	0.193467	1.913219
C	5.473558	-2.217134	0.642656	C	-3.618361	3.752922	1.269009
C	3.505428	-3.092266	2.339736	C	-1.000816	3.882513	2.07824
C	4.782338	-3.60963	2.453747	C	-1.879302	4.883597	2.449555
H	2.728409	-3.472536	3.01191	H	0.045138	3.964174	2.394223
C	5.773251	-3.172672	1.592938	C	-3.198071	4.820912	2.036972

H	5.000728	-4.364646	3.216818	H	-1.526184	5.721348	3.060837
H	6.788459	-3.577848	1.662166	H	-3.905784	5.609608	2.314128
H	6.249098	-1.863212	-0.045641	H	-4.65923	3.69298	0.931861
C	0.487763	-3.092803	1.453865	C	1.318858	2.542493	0.431378
C	1.188641	-0.505704	2.721065	C	0.141734	0.595859	2.341674
C	4.544419	1.766147	1.823859	C	-3.924857	-0.150629	2.918045
H	4.820462	2.5795	1.127209	H	-4.647447	-0.877446	2.502504
C	3.864218	2.350006	3.025223	C	-3.149202	-0.808086	4.017917
C	5.768427	0.956891	2.167921	C	-4.645579	1.096436	3.362354
C	3.408353	-3.11809	-2.082536	C	-2.410474	3.524558	-2.059096
H	2.817057	-2.840457	-1.188718	H	-1.692356	3.093295	-1.335081
C	2.499878	-3.060008	-3.280831	C	-2.043734	3.029193	-3.431269
C	4.028402	-4.469029	-1.876182	C	-2.400369	5.020989	-1.948101
C	-0.99986	-2.790299	1.388249	C	2.455286	1.63382	-0.008653
C	0.836546	-4.123477	0.390633	C	1.056535	3.572594	-0.656667
H	0.710209	-3.53471	2.449726	H	1.640321	3.081879	1.34885
C	-1.845142	-4.037515	1.514742	C	3.710176	2.403519	-0.353423
H	-1.215001	-2.297444	0.422999	H	2.121333	1.063863	-0.894386
H	-1.288575	-2.060622	2.162749	H	2.677113	0.87937	0.76338
C	0.002619	-5.377874	0.529187	C	2.30527	4.361859	-0.984137
H	0.648116	-3.669163	-0.608036	H	0.714597	3.035326	-1.570009
H	1.913518	-4.373513	0.422607	H	0.235308	4.255766	-0.369507
C	-1.473961	-5.062091	0.467907	C	3.434654	3.450903	-1.40774
H	-2.914543	-3.769199	1.433802	H	4.491481	1.700997	-0.699038
H	-1.709995	-4.480243	2.523343	H	4.113276	2.889943	0.559631
H	0.2819	-6.107043	-0.253463	H	2.087045	5.109015	-1.769427
H	0.233771	-5.86597	1.498167	H	2.615633	4.939724	-0.089522
H	-2.078918	-5.979924	0.580654	H	4.345954	4.033864	-1.634459
H	-1.714421	-4.650996	-0.534352	H	3.149169	2.940115	-2.351091

C	0.212675	0.625492	2.422936	C	0.434235	-0.868123	2.039973
C	0.827941	-1.252062	3.99479	C	1.172767	1.202851	3.276858
H	2.183018	-0.044276	2.88123	H	-0.837483	0.629341	2.859068
C	-0.004755	1.534709	3.608892	C	0.649962	-1.683986	3.292671
H	-0.765947	0.209865	2.10342	H	1.328325	-0.955773	1.387655
H	0.581229	1.212892	1.560544	H	-0.400856	-1.295767	1.453281
C	0.64629	-0.294525	5.155145	C	1.340459	0.361465	4.526382
H	-0.115669	-1.817819	3.866994	H	2.157413	1.281343	2.778399
H	1.603636	-1.999856	4.243804	H	0.886065	2.233653	3.557887
C	-0.398273	0.75157	4.838955	C	1.699285	-1.067286	4.186424
H	-0.758761	2.293703	3.340609	H	0.914155	-2.714871	3.003612
H	0.933283	2.085944	3.82079	H	-0.305402	-1.746434	3.850576
H	0.386585	-0.856731	6.071399	H	2.10317	0.816837	5.185694
H	1.611647	0.210534	5.36531	H	0.390391	0.36921	5.100269
H	-0.553967	1.421136	5.704059	H	1.831418	-1.662325	5.108231
H	-1.372636	0.249113	4.659793	H	2.682059	-1.081663	3.671774
H	3.600611	1.56157	3.752645	H	-2.407059	-0.111739	4.447523
H	4.532817	3.061667	3.535377	H	-3.824669	-1.12061	4.829466
H	2.954774	2.877554	2.703891	H	-2.638225	-1.693687	3.61563
H	6.512268	1.575497	2.696221	H	-5.366062	0.868386	4.164808
H	5.500502	0.10376	2.815333	H	-3.92782	1.845628	3.738958
H	6.250111	0.553013	1.2626	H	-5.204088	1.556466	2.530437
H	2.011043	-2.074876	-3.365152	H	-2.004308	1.927474	-3.458201
H	3.05943	-3.266865	-4.209188	H	-2.767094	3.383929	-4.185561
H	1.701028	-3.814268	-3.1809	H	-1.042646	3.400412	-3.708207
H	3.25046	-5.242349	-1.774173	H	-1.412107	5.423308	-2.221871
H	4.666991	-4.738441	-2.73408	H	-3.149291	5.46604	-2.624536
H	4.650777	-4.478517	-0.967679	H	-2.633015	5.33889	-0.919817
H	5.003939	-1.114617	-4.414677	H	-5.115657	2.130817	-3.492324

H	3.358382	-0.55604	-4.750945	H	-4.105878	0.794822	-4.075516
H	4.699751	0.596176	-4.82504	H	-5.758235	0.466331	-3.536276
C	-2.140177	0.215211	-0.547136	C	1.256713	0.01029	-3.291191
C	-3.334656	-0.423969	-0.233234	C	2.615601	0.188755	-3.486479
C	-4.300832	0.115402	0.815647	C	3.537286	-0.558281	-2.769238
C	-5.714005	0.164207	0.246395	C	3.13275	-1.501332	-1.831447
C	-1.598439	-1.503274	-2.171457	C	4.187437	-2.372618	-1.142907
C	-2.774291	-2.152557	-1.834763	C	5.138046	-1.45906	-0.371773
C	-3.62823	-1.620057	-0.880219	C	4.746615	-0.893667	0.841992
C	-5.954037	0.423645	-1.097097	C	5.569277	-0.026749	1.526069
C	-7.23536	0.55411	-1.603053	C	6.829704	0.319106	1.039911
C	-8.339567	0.424515	-0.775456	C	7.232583	-0.2462	-0.156453
C	-8.107956	0.162204	0.571725	C	6.397936	-1.11688	-0.840896
C	-6.828516	0.039765	1.070776	C	1.769435	-1.638417	-1.587942
C	-4.225154	-0.818811	2.020109	C	4.953371	-3.137571	-2.217249
H	-4.574545	-1.838956	1.786948	H	5.385539	-2.485728	-2.992037
H	-3.179469	-0.887761	2.362183	H	5.770721	-3.729508	-1.768853
Cl	-9.459136	-0.009795	1.62893	H	3.76611	-1.121251	1.272419
H	-1.851008	1.145381	-0.042499	H	8.214728	-0.013	-0.575097
H	-3.043554	-3.092568	-2.325299	H	2.975306	0.919824	-4.216162
H	-7.365092	0.762577	-2.667675	H	1.399408	-2.334864	-0.82559
H	-6.722416	-0.155375	2.141105	H	4.283292	-3.83426	-2.739816
H	-4.808942	-0.442247	2.876628	P	-4.051336	-2.443658	0.449043
P	3.176396	3.421621	-0.778608	O	-4.004271	-3.253512	-0.788145
O	2.496238	3.894727	-2.003815	O	-3.067242	-2.67651	1.521153
O	2.468084	3.553722	0.514887	O	-5.516292	-2.591438	1.134811
O	4.643718	4.113987	-0.644923	C	-6.623638	-2.600594	0.293888
C	4.759691	5.20892	0.207325	H	-6.447538	-3.283036	-0.560097
H	5.843962	5.350431	0.375372	H	-6.793993	-1.586611	-0.131671

H	4.293571	4.981493	1.188015	C	-7.81741	-3.030295	1.092877
C	4.156738	6.456099	-0.377986	H	-7.99122	-2.347881	1.939627
H	3.068227	6.345007	-0.498808	H	-7.664291	-4.039873	1.504805
H	4.579573	6.662705	-1.373352	H	-8.726055	-3.043832	0.4702
H	4.343111	7.327347	0.271921	C	3.569163	-3.39507	-0.179437
C	-3.924333	1.515916	1.321105	H	2.945383	-2.888655	0.578359
H	-2.932126	1.461063	1.809102	H	4.406574	-3.85074	0.381616
H	-4.638395	1.751125	2.132882	C	2.703507	-4.475633	-0.802201
C	-3.884129	2.644007	0.31081	H	2.467678	-4.245042	-1.857387
H	-4.038219	2.274122	-0.718593	H	3.21808	-5.450234	-0.846804
H	-4.709986	3.359609	0.459731	C	1.329207	-4.679986	-0.143595
C	-2.567308	3.436637	0.268787	O	0.975395	-3.834461	0.700754
O	-2.515329	4.362766	-0.547421	O	0.657341	-5.622987	-0.573554
O	-1.645025	3.045137	1.010638	K	-1.493866	-4.1031	-0.080953
K	0.112697	3.892048	-0.69398	Cl	5.046812	0.678156	3.007757
H	-5.109088	0.521146	-1.785524	H	6.770096	-1.53827	-1.778474
H	-4.570494	-2.137755	-0.666418	H	4.604394	-0.392544	-2.957358
O	-9.615921	0.525873	-1.163699	O	0.294028	0.636949	-3.983866
C	-9.829674	0.820791	-2.497256	C	0.716215	1.51455	-4.959469
H	-9.376621	1.787802	-2.786033	H	1.273407	2.373169	-4.53048
H	-9.436016	0.031512	-3.165672	H	1.356867	1.020333	-5.715462
H	-10.918291	0.883601	-2.622743	H	-0.191734	1.889089	-5.450373
O	-0.733167	-1.915215	-3.109943	O	7.550118	1.17141	1.777345
C	-1.045976	-3.090029	-3.759877	C	8.79064	1.517316	1.278275
H	-1.060271	-3.955893	-3.065572	H	9.460045	0.640834	1.183925
H	-2.024267	-3.034663	-4.275625	H	8.717444	2.014429	0.292215
H	-0.257692	-3.245895	-4.508199	H	9.223667	2.220154	2.001601
**TS-4-*R*/L11@2K**				**TS-4-*S*/L11@2K**			
Thermal correction to Gibbs Free Energy=				Thermal correction to Gibbs Free Energy=			

1.039355<br>E(RM11L) = -5459.95444923<br>Frequency -115.3015				1.038942<br>E(RM11L) = -5459.95874437<br>Frequency -148.4331			
C	1.092	0.897	-2.69	C	0.535	-2.015	-0.966
C	2.337	-2.51	1.758	C	-3.387	0.883	-1.33
C	1.634	-3.558	1.136	C	-3.334	0.634	-2.714
C	0.541	-3.394	0.147	C	-2.432	1.749	-0.786
C	0.579	-4.07	-1.082	C	-4.495	0.354	-0.497
C	-0.633	-2.723	0.494	C	-2.352	1.203	-3.526
C	-0.521	-4.098	-1.941	C	-1.382	1.991	-2.925
C	-1.658	-3.407	-1.559	C	-2.327	0.961	-4.993
C	-0.479	-4.85	-3.223	C	-1.396	2.266	-1.569
C	-1.731	-2.7	-0.368	C	-4.314	-0.436	0.657
C	1.916	-4.872	1.511	C	-5.791	0.673	-0.9
C	3.253	-2.84	2.759	C	-5.45	-0.864	1.344
C	3.498	-4.145	3.138	C	-6.728	-0.534	0.934
C	2.832	-5.173	2.496	C	-6.898	0.241	-0.198
C	3.918	-0.125	1.404	C	-2.906	-2.693	1.823
C	1.291	0.019	2.715	C	-2.338	0.015	2.733
C	-1.627	-2.726	2.605	C	-2.858	3.482	0.713
C	-2.154	-1.641	3.493	C	-2.259	3.882	2.029
C	-0.945	-3.835	3.36	C	-4.35	3.668	0.655
C	2.547	-4.281	-2.361	C	-3.937	-1.427	-3.677
C	3.289	-5.46	-2.919	C	-4.895	-1.805	-4.766
C	3.451	-3.262	-1.73	C	-3.99	-2.349	-2.494
C	3.957	1.335	0.972	C	-1.602	-3.236	2.394
C	4.905	-0.913	0.558	C	-3.412	-3.625	0.736
C	5.344	1.916	1.123	C	-1.772	-4.647	2.913
C	6.303	-0.347	0.697	C	-3.583	-5.038	1.25

C	6.354	1.119	0.329	C	-2.3	-5.574	1.844
C	0.335	1.097	2.221	C	-0.841	0.247	2.903
C	2.161	0.517	3.858	C	-3.001	-0.475	4.009
C	-0.446	1.742	3.346	C	-0.516	1.082	4.125
C	1.312	1.097	4.97	C	-2.684	0.449	5.166
C	0.458	2.231	4.455	C	-1.191	0.556	5.371
C	2.054	1.339	-3.613	C	1.577	-1.592	-0.158
C	2.019	2.673	-3.967	C	2.277	-2.485	0.648
C	1.039	3.525	-3.463	C	3.475	-1.979	1.431
C	0.084	3.103	-2.555	C	4.68	-1.875	0.51
C	-0.954	4.032	-1.962	C	0.235	-3.38	-1.084
C	-0.637	4.302	-0.489	C	0.923	-4.267	-0.279
C	0.687	4.298	-0.055	C	1.927	-3.825	0.581
C	1.017	4.707	1.22	C	4.677	-2.279	-0.816
C	0.045	5.13	2.124	C	5.786	-2.107	-1.639
C	-1.274	5.124	1.696	C	6.948	-1.532	-1.151
C	-1.604	4.726	0.413	C	6.958	-1.124	0.188
C	0.135	1.756	-2.175	C	5.856	-1.297	0.994
C	-0.947	5.396	-2.646	C	3.791	-2.869	2.621
C	-2.407	3.467	-2.13	C	3.115	-0.553	1.957
C	3.84	0.882	-5.006	C	8.068	-1.768	-3.163
C	-0.514	5.789	4.269	C	-0.99	-5.043	-2.138
C	-2.921	-1.842	-0.069	C	-0.259	2.952	-0.908
N	-4.105	-2.444	-0.219	N	0.221	4.042	-1.51
C	-5.357	-1.752	-0.057	C	1.465	4.611	-1.057
C	-5.453	-0.481	-0.933	C	1.295	5.416	0.214
C	-5.679	-1.465	1.395	C	2.545	3.497	-0.972
C	-5.712	-2.664	2.323	C	0.206	6.471	0.196
C	-6.039	-2.21	3.727	C	0.095	7.123	1.554

C	-6.696	-3.723	1.874	C	0.426	7.513	-0.878
H	7.372	1.521	0.471	H	-2.448	-6.592	2.243
Cl	0.576	-1.034	-3.079	Cl	0.258	-0.971	-2.559
Pd	1.539	-0.034	-0.899	Pd	-1.205	-0.701	-0.631
P	2.182	-0.749	1.264	O	-4.304	-0.109	-3.279
O	1.659	-4.816	-1.384	H	-0.543	2.352	-3.535
H	-2.529	-3.408	-2.232	O	-2.547	2.11	0.505
O	-0.707	-2.129	1.695	P	-2.643	-0.977	1.188
H	3.815	-2.043	3.256	H	-5.336	-1.497	2.231
H	4.222	-4.358	3.931	H	-7.594	-0.89	1.501
H	3.021	-6.216	2.768	H	-7.902	0.51	-0.543
H	1.373	-5.681	1.011	H	-5.921	1.28	-1.802
H	-2.467	-3.151	2.015	H	-2.38	4.077	-0.095
H	1.951	-3.791	-3.159	H	-2.897	-1.406	-4.067
H	4.264	-0.169	2.459	H	-3.656	-2.686	2.644
H	3.646	1.395	-0.094	H	-0.825	-3.214	1.605
H	3.223	1.945	1.524	H	-1.218	-2.594	3.204
H	4.587	-0.854	-0.505	H	-2.682	-3.619	-0.1
H	4.897	-1.985	0.821	H	-4.363	-3.255	0.312
H	5.345	2.975	0.811	H	-0.81	-5.017	3.309
H	5.628	1.911	2.195	H	-2.475	-4.639	3.77
H	7.011	-0.929	0.081	H	-3.944	-5.695	0.438
H	6.639	-0.473	1.746	H	-4.375	-5.051	2.026
H	4.156	-2.852	-2.47	H	-3.74	-3.382	-2.79
H	6.128	1.227	-0.751	H	-1.536	-5.664	1.046
H	0.682	-0.815	3.115	H	-2.797	0.994	2.498
H	0.906	1.88	1.681	H	-0.287	-0.708	2.97
H	-0.357	0.66	1.475	H	-0.47	0.724	1.975
H	2.855	1.307	3.511	H	-2.656	-1.492	4.278

H	2.791	-0.3	4.254	H	-4.095	-0.545	3.872
H	-1.064	2.563	2.94	H	0.58	1.097	4.278
H	-1.158	1.008	3.769	H	-0.844	2.131	3.964
H	1.958	1.431	5.802	H	-3.184	0.097	6.085
H	0.659	0.302	5.386	H	-3.098	1.457	4.957
H	-0.136	2.673	5.276	H	-0.958	1.197	6.239
H	1.116	3.039	4.077	H	-0.783	-0.446	5.608
H	-1.362	-1.249	4.152	H	-2.731	3.338	2.865
H	-2.955	-2.034	4.138	H	-2.403	4.959	2.206
H	-2.555	-0.81	2.892	H	-1.179	3.664	2.036
H	-1.621	-4.263	4.117	H	-4.627	4.715	0.858
H	-0.046	-3.454	3.874	H	-4.845	3.024	1.401
H	-0.628	-4.649	2.691	H	-4.75	3.398	-0.335
H	2.601	-6.186	-3.378	H	-4.832	-1.112	-5.619
H	3.842	-5.975	-2.119	H	-5.929	-1.782	-4.387
H	4.012	-5.14	-3.684	H	-4.69	-2.822	-5.132
H	4.027	-3.713	-0.904	H	-4.996	-2.344	-2.04
H	2.872	-2.415	-1.327	H	-3.255	-2.045	-1.73
H	-0.088	-4.236	-4.054	H	-1.717	0.081	-5.263
H	-1.485	-5.183	-3.519	H	-1.892	1.821	-5.525
H	0.166	-5.739	-3.147	H	-3.339	0.793	-5.39
H	0.006	5.926	-2.484	H	4.124	-3.873	2.316
H	-1.756	6.005	-2.222	H	2.907	-2.957	3.268
H	1.496	3.978	-0.721	Cl	8.38	-0.387	0.815
H	-2.07	5.447	2.372	H	1.828	-0.524	-0.156
H	2.759	3.076	-4.664	H	0.682	-5.334	-0.308
H	-0.594	1.383	-1.445	H	5.734	-2.453	-2.674
H	-1.125	5.309	-3.731	H	5.907	-0.929	2.023
O	-2.555	2.284	-2.486	H	4.599	-2.435	3.231

O	-3.325	4.259	-1.896	O	2.373	-0.468	2.93
K	-2.374	-0.193	-2.61	O	3.586	0.401	1.301
Cl	2.666	4.709	1.727	K	4.401	1.16	-0.928
H	-2.647	4.773	0.091	H	3.782	-2.741	-1.244
H	1.045	4.561	-3.812	H	2.46	-4.569	1.178
O	2.917	0.433	-4.075	O	8.065	-1.323	-1.85
H	4.509	1.654	-4.584	H	7.306	-1.251	-3.776
H	3.348	1.288	-5.909	H	7.898	-2.858	-3.228
H	4.437	0.005	-5.286	H	9.063	-1.534	-3.558
O	0.466	5.503	3.334	O	-0.729	-3.697	-1.959
H	-1.197	4.932	4.423	H	-1.406	-5.513	-1.226
H	-1.11	6.677	3.987	H	-0.087	-5.602	-2.447
H	0.016	5.999	5.207	H	-1.74	-5.104	-2.938
O	-2.805	-0.665	0.216	O	0.211	2.55	0.142
H	-4.105	-3.438	-0.391	H	-0.123	4.266	-2.431
H	-6.125	-2.435	-0.469	H	1.8	5.284	-1.868
O	-6.236	0.379	-0.528	H	1.092	4.723	1.051
O	-4.782	-0.449	-1.981	H	2.269	5.883	0.449
H	-6.655	-0.95	1.414	O	3.252	3.465	0.048
H	-4.947	-0.726	1.772	K	1.949	2.202	2.028
H	-4.702	-3.126	2.342	O	2.606	2.756	-1.957
H	-7.059	-1.791	3.775	H	-0.758	5.96	-0.015
H	-5.35	-1.42	4.07	H	1.027	7.656	1.816
H	-5.99	-3.039	4.453	H	-0.088	6.378	2.348
H	-6.772	-4.54	2.609	H	-0.724	7.859	1.594
H	-6.42	-4.186	0.911	H	0.397	7.088	-1.894
H	-7.707	-3.296	1.752	H	1.408	8.004	-0.756
K	-5.133	2.315	-1.819	H	-0.341	8.303	-0.837
**TS-4-*R*/L11@1K**				**TS-4-*S*/L11@1K**			

Thermal correction to Gibbs Free Energy= 1.040786<br>E(RM11L) = -4860.05634589<br>Frequency -147.4584				Thermal correction to Gibbs Free Energy=1.039500<br>E(RM11L) = -4860.06198340<br>Frequency -130.8448			
C	0.819	-1.05	-2.609	C	-0.477	-2.148	-1.729
C	-2.737	-1.907	0.836	C	-2.652	2.166	-0.624
C	-3.415	-2.58	-0.195	C	-2.895	2.201	-2.007
C	-2.778	-0.508	0.841	C	-1.365	2.491	-0.179
C	-2.129	-2.634	1.978	C	-3.745	1.9	0.344
C	-4.069	-1.888	-1.215	C	-1.884	2.501	-2.92
C	-4.037	-0.502	-1.193	C	-0.607	2.729	-2.433
C	-4.794	-2.606	-2.298	C	-2.157	2.549	-4.382
C	-3.407	0.199	-0.18	C	-0.322	2.727	-1.078
C	-0.781	-2.503	2.375	C	-3.723	0.851	1.288
C	-2.972	-3.458	2.721	C	-4.855	2.74	0.296
C	-0.364	-3.186	3.517	C	-4.82	0.707	2.136
C	-1.219	-3.985	4.252	C	-5.913	1.552	2.076
C	-2.532	-4.129	3.844	C	-5.93	2.576	1.147
C	1.951	-2.623	1.438	C	-3.133	-1.973	1.753
C	0.836	-0.056	2.304	C	-1.433	0.097	2.902
C	-3.048	0.882	2.711	C	-0.84	3.886	1.611
C	-2.301	2.107	3.154	C	0.062	3.736	2.8
C	-3.472	-0.006	3.851	C	-2.121	4.617	1.918
C	-2.705	-4.634	-1.098	C	-4.416	0.751	-3.07
C	-3.424	-5.925	-1.367	C	-5.536	0.994	-4.04
C	-1.324	-4.834	-0.548	C	-4.757	-0.267	-2.02
C	3.068	-1.953	0.65	C	-2.041	-3.034	1.831
C	1.727	-4.019	0.883	C	-4.173	-2.402	0.732
C	4.341	-2.77	0.672	C	-2.604	-4.394	2.172
C	2.992	-4.847	0.938	C	-4.758	-3.753	1.088

C	4.113	-4.178	0.176	C	-3.685	-4.813	1.203
C	1.16	1.084	1.344	C	0.047	-0.204	2.721
C	1.868	-0.2	3.411	C	-1.98	-0.49	4.193
C	1.487	2.377	2.057	C	0.875	0.138	3.938
C	2.069	1.121	4.125	C	-1.134	-0.068	5.377
C	2.514	2.187	3.15	C	0.304	-0.492	5.186
C	1.645	-2.119	-2.969	C	0.714	-2.385	-1.075
C	2.995	-1.863	-3.133	C	1.071	-3.652	-0.619
C	3.504	-0.576	-2.977	C	2.313	-3.807	0.226
C	2.687	0.496	-2.623	C	3.534	-3.153	-0.386
C	3.196	1.927	-2.488	C	-1.35	-3.201	-2.025
C	3.988	2.175	-1.219	C	-1.003	-4.464	-1.584
C	4.631	1.181	-0.493	C	0.184	-4.686	-0.89
C	5.35	1.484	0.647	C	3.61	-2.624	-1.666
C	5.467	2.788	1.111	C	4.696	-1.866	-2.087
C	4.852	3.79	0.373	C	5.783	-1.653	-1.254
C	4.135	3.482	-0.766	C	5.761	-2.281	-0.004
C	1.333	0.227	-2.469	C	4.662	-2.991	0.422
C	4.065	2.275	-3.689	C	2.594	-5.261	0.544
C	1.922	2.847	-2.499	C	1.94	-3.046	1.587
C	1.85	-4.375	-3.457	C	6.644	0.038	-2.564
C	6.069	4.235	2.808	C	-3.35	-3.91	-2.951
C	-3.323	1.689	-0.252	C	1.08	2.842	-0.582
N	-4.515	2.305	-0.322	N	1.821	3.798	-1.151
C	-4.573	3.667	-0.795	C	3.243	3.841	-0.921
C	-3.725	3.811	-2.094	C	3.575	4.423	0.44
C	-4.171	4.658	0.277	C	3.88	2.431	-1.128
C	-4.899	4.532	1.601	C	2.904	5.738	0.786
C	-4.305	5.485	2.613	C	3.264	6.147	2.196

C	-6.388	4.76	1.469	C	3.246	6.84	-0.19
H	5.043	-4.773	0.237	H	-4.127	-5.783	1.495
Cl	-0.99	-1.179	-3.159	Cl	-0.471	-0.701	-3.008
Pd	-0.459	-1.326	-0.792	Pd	-1.288	-0.333	-0.748
O	-3.5	-3.929	-0.155	O	-4.153	2.01	-2.46
H	-4.479	0.067	-2.023	H	0.216	2.852	-3.149
O	-2.186	0.144	1.855	O	-1.158	2.583	1.144
P	0.42	-1.587	1.332	P	-2.341	-0.357	1.337
H	0.681	-3.104	3.839	H	-4.828	-0.112	2.864
H	-0.853	-4.505	5.144	H	-6.758	1.405	2.758
H	-3.223	-4.768	4.405	H	-6.788	3.254	1.082
H	-4.013	-3.562	2.396	H	-4.866	3.545	-0.446
H	-3.941	1.192	2.13	H	-0.293	4.423	0.808
H	-2.631	-4.033	-2.029	H	-3.506	0.413	-3.608
H	2.287	-2.718	2.494	H	-3.636	-1.915	2.744
H	2.728	-1.815	-0.395	H	-1.523	-3.084	0.854
H	3.278	-0.941	1.031	H	-1.248	-2.781	2.551
H	1.402	-3.924	-0.174	H	-3.687	-2.46	-0.263
H	0.905	-4.533	1.415	H	-4.978	-1.648	0.639
H	5.119	-2.268	0.07	H	-1.779	-5.127	2.188
H	4.737	-2.802	1.708	H	-3.023	-4.373	3.2
H	2.801	-5.862	0.543	H	-5.52	-4.045	0.341
H	3.299	-4.982	1.995	H	-5.296	-3.671	2.055
H	-0.685	-5.376	-1.267	H	-5.003	-1.238	-2.482
H	3.844	-4.134	-0.899	H	-3.225	-4.97	0.207
H	-0.12	0.194	2.801	H	-1.559	1.195	2.972
H	2.012	0.813	0.689	H	0.207	-1.274	2.498
H	0.309	1.241	0.655	H	0.417	0.344	1.835
H	2.851	-0.522	3.013	H	-1.978	-1.596	4.157

H	1.556	-0.979	4.133	H	-3.033	-0.184	4.348
H	1.821	3.115	1.306	H	1.907	-0.208	3.762
H	0.563	2.788	2.511	H	0.918	1.238	4.08
H	2.801	1.001	4.945	H	-1.556	-0.487	6.31
H	1.115	1.429	4.601	H	-1.179	1.036	5.487
H	2.706	3.141	3.676	H	0.908	-0.231	6.074
H	3.483	1.878	2.71	H	0.353	-1.594	5.085
H	-1.431	1.837	3.777	H	-0.459	3.234	3.632
H	-2.955	2.757	3.758	H	0.39	4.726	3.155
H	-1.943	2.674	2.281	H	0.948	3.141	2.532
H	-4.089	0.549	4.576	H	-1.918	5.62	2.325
H	-2.586	-0.402	4.375	H	-2.712	4.051	2.658
H	-4.058	-0.868	3.493	H	-2.742	4.734	1.016
H	-4.429	-5.752	-1.783	H	-5.256	1.735	-4.804
H	-3.538	-6.496	-0.431	H	-6.423	1.373	-3.507
H	-2.858	-6.546	-2.078	H	-5.819	0.061	-4.55
H	-1.356	-5.398	0.4	H	-5.614	0.068	-1.411
H	-0.846	-3.859	-0.364	H	-3.894	-0.436	-1.356
H	-4.14	-2.846	-3.155	H	-2.054	1.559	-4.862
H	-5.614	-1.988	-2.693	H	-1.448	3.221	-4.89
H	-5.224	-3.554	-1.937	H	-3.176	2.909	-4.589
H	4.984	1.666	-3.735	H	2.707	-5.873	-0.369
H	4.376	3.331	-3.638	H	1.783	-5.666	1.168
H	4.554	0.132	-0.794	Cl	7.119	-2.094	1.043
H	4.911	4.831	0.701	H	1.373	-1.53	-0.9
H	3.681	-2.676	-3.391	H	-1.67	-5.311	-1.771
H	0.643	1.022	-2.175	H	4.669	-1.402	-3.075
H	3.49	2.143	-4.616	H	4.66	-3.371	1.448
O	1.501	3.173	-3.611	H	3.526	-5.359	1.122

O	1.409	3.081	-1.395	O	1.197	-3.663	2.335
Cl	6.087	0.22	1.567	O	2.407	-1.903	1.694
H	3.627	4.292	-1.299	K	3.273	0.067	0.29
H	4.574	-0.419	-3.15	H	2.775	-2.749	-2.362
O	1.047	-3.315	-3.096	H	0.405	-5.704	-0.561
H	2.621	-4.596	-2.693	O	6.838	-0.897	-1.546
H	2.356	-4.204	-4.428	H	5.713	0.613	-2.409
H	1.186	-5.245	-3.55	H	6.633	-0.445	-3.561
O	6.146	2.973	2.251	H	7.506	0.714	-2.513
H	5.019	4.543	2.983	O	-2.467	-2.881	-2.703
H	6.562	5.002	2.181	H	-3.749	-4.354	-2.016
H	6.593	4.176	3.771	H	-2.892	-4.718	-3.556
O	-2.266	2.282	-0.203	H	-4.182	-3.471	-3.518
H	-5.286	1.701	-0.57	O	1.509	2.137	0.312
H	-5.63	3.843	-1.074	H	1.439	4.255	-1.965
O	-3.21	4.917	-2.276	H	3.655	4.498	-1.711
O	-3.625	2.784	-2.775	H	3.32	3.671	1.207
H	-4.314	5.67	-0.141	H	4.673	4.531	0.494
H	-3.084	4.562	0.445	O	4.843	2.173	-0.399
H	-4.748	3.498	1.978	O	3.339	1.714	-1.975
H	-4.431	6.534	2.289	H	1.804	5.582	0.751
H	-3.224	5.312	2.742	H	4.35	6.328	2.29
H	-4.782	5.388	3.604	H	3.003	5.359	2.923
H	-6.895	4.717	2.449	H	2.749	7.073	2.503
H	-6.871	4.01	0.822	H	2.895	6.62	-1.212
H	-6.598	5.753	1.033	H	4.34	6.992	-0.246
K	-0.992	3.391	-2.507	H	2.796	7.803	0.107

Derivatization of desymmetrization products

A) Ethyl (*S*)-4-(4'-(tert-butyl)-6-methoxy-[1,1'-biphenyl]-3-yl)-4-(4-methoxy-3-((4-methoxy-phenyl)amino)phenyl)pentanoate (83)

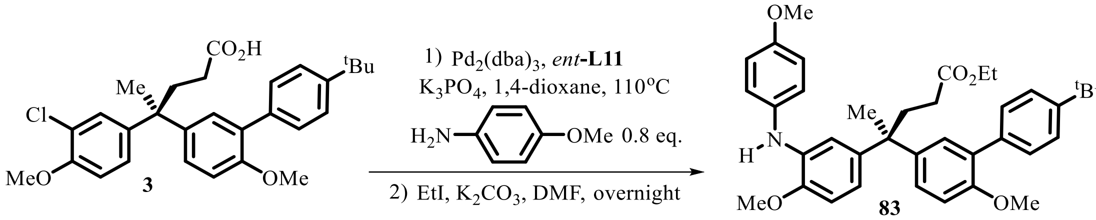

Under N_2 atmosphere, a mixture of $Pd_2(dba)_3$ (0.32 mg, 0.000354 mmol) and *ent*-**L11** (0.45 mg, 0.000708 mmol) was added 0.1 mL 1,4-dioxane, and the mixture was stirred at room temperature for 20 min. The resulting metal-ligand complex solution was added to a reaction flask containing 0.8 mL 1,4-dioxane solution of (*S*)-4-(4'-(tert-butyl)-6-methoxy-[1,1'-biphenyl]-3-yl)-4-(3-chloro-4-methoxyphenyl)-pentanoic acid **3** (17 mg, 0.0354 mmol), *p*-anisidine (3.49 mg, 0.0283 mmol), and K_3PO_4 (45 mg, 0.212 mmol). Then the resulting reaction mixture was stirred at 110°C for 5 h. The reaction was then quenched with water, neutralized to pH 4 – 6 with saturated $KH_2PO_{4\,(aq.)}$, and extracted with ethyl acetate (2 mL X 3) three times. The combined organic phase was washed with brine, dried over Na_2SO_4, and concentrated in vacuo. After that, the obtained crude product was dissolved in DMF (1.0 mL), and treated with EtI (78 mg, 0.5 mmol) and K_2CO_3 (138 mg, 1.0 mmol). The reaction mixture was stirred overnight at room temperature. Then the mixture was diluted with ethyl acetate (20 mL) and washed with water. The organic layer was concentrated in vacuo and purified by flash column chromatography with hexane and acetone (50: 1 – 10: 1) to give the desired product, ethyl (*S*)-4-(4'-(tert-butyl)-6-methoxy-[1,1'-biphenyl]-3-yl)-4-(4-methoxy-3-((4-methoxyphen -yl)-amino)-phenyl)-pentanoate **83**, reddish solid, 11 mg, 65%, $[\alpha]_D^{20}$ = -10.50 (*c*

1.000 $CHCl_3$) for 93:7 er ^{1}H NMR (400 MHz, Chloroform-*d*) δ 7.36 – 7.30 (m, 4H), 7.09 (d, J = 2.5 Hz, 1H), 7.04 (dd, J = 8.6, 2.5 Hz, 1H), 6.87 (d, J = 8.7 Hz, 3H), 6.78 (d, J = 8.6 Hz, 1H), 6.68 (d, J = 8.6 Hz, 3H), 6.61 (d, J = 8.5 Hz, 1H), 3.99 (q, J = 7.1 Hz, 2H), 3.78 (s, 3H), 3.72 (s, 3H), 3.68 (s, 3H), 2.34 – 2.26 (m, 2H), 2.09 – 2.05 (m, 2H), 1.49 (s, 3H), 1.27 (s, 9H), 1.12 (t, J = 7.1 Hz, 3H). ^{13}C NMR (101 MHz, Chloroform-*d*) δ 174.1, 154.5, 149.6, 145.9, 141.3, 135.8, 133.6, 129.9, 129.8, 129.2, 128.2, 127.0, 124.9, 121.1, 117.3, 114.6, 113.4, 113.3, 110.6, 109.6, 60.3, 55.6, 44.8, 36.5, 34.5, 31.4, 30.5, 27.6, 21.1, 14.2. HRMS (m/z, ESI): Calcd. for Chemical Formula: $C_{38}H_{46}ClNO_5^+$ $[M+Na]^+$: 596.3371, Found: 596.3376. HPLC analysis of the reaction product: Daicel Chiralpak IA, hexane/iso-propanol = 97: 3, 1.0 mL/min, λ= 280 nm, retention time (5 h): 11.93 min (major) and 10.21 min (minor).

B) Diethyl 2,2'-(ethane-1,2-diylbis(4-methoxy-3,1-phenylene))-(2*R*,2'*R*)-bis(2-(4',6-dimethoxy-[1,1'-biphenyl]-3-yl)propanoate) (86)

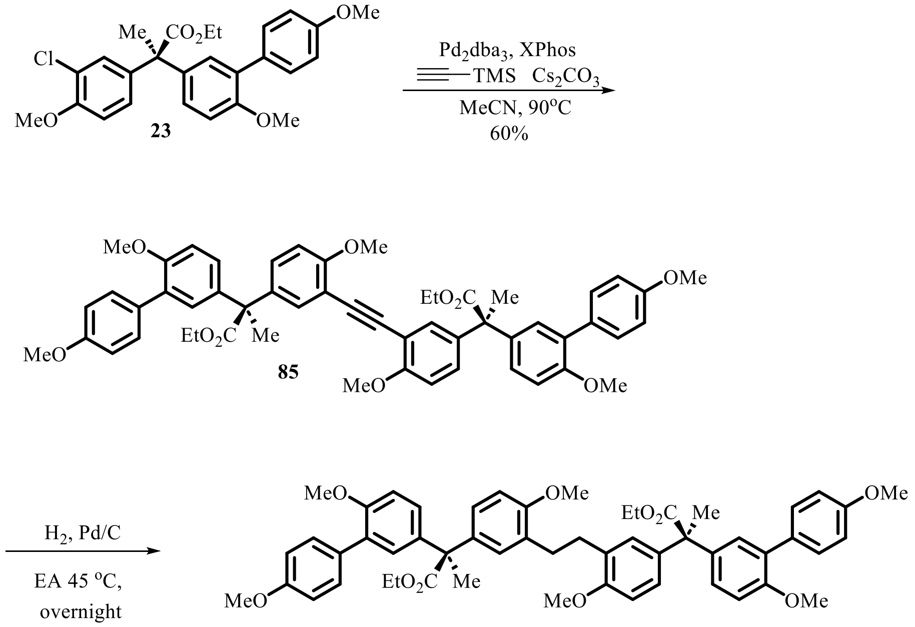

86, 93%, 99.5:0.5 er

Under N_2 atmosphere, $Pd_2(dba)_3$ (6.7 mg, 0.00735 mmol), Cs_2CO_3 (205 mg, 0.63 mmol), Xphos

(7.0 mg, 0.0147 mmol), and ethyl (*S*)-2-(3-chloro-4-methoxyphenyl)-2-(4',6-dimethoxy-[1,1'-biphenyl]-3-yl)-propanoate **23** (94 mg, 0.21 mmol) were added to 2.0 mL MeCN, the mixture was then stirred at room temperature for 25 min. After that, trimethylsilylacetylene **84** (12 mg, 0.126 mmol) was added to the above solution. Then the resulting reaction mixture was stirred at 90°C for 20 h. The reaction was then quenched with water and extracted with ethyl acetate (7 mL X 3) three times. The combined organic phase was washed with brine, dried over Na_2SO_4, concentrated in vacuo, and purified by flash column chromatography with hexane and acetone (20:1 – 3:1) to give the desired product **85**, diethyl 2,2'-(ethyne-1,2-diylbis(4-methoxy-3,1-phenylene))-(2*S*,2'*S*)-bis(2-(4',6-dimethoxy-[1,1'-biphenyl]-3-yl)propanoate), white solid, 55 mg, 61%, $[\alpha]_D^{20}$ = -4.80 (*c* 0.500 $CHCl_3$). ^{1}H NMR (400 MHz, Chloroform-*d*) δ 7.47 (d, *J* = 2.5 Hz, 2H), 7.45 – 7.40 (m, 4H), 7.20 – 7.14 (m, 4H), 7.10 (dd, *J* = 8.6, 2.6 Hz, 2H), 6.95 – 6.90 (m, 4H), 6.88 (d, *J* = 8.7 Hz, 2H), 6.81 (d, *J* = 8.8 Hz, 2H), 4.21 (q, *J* = 7.1 Hz, 4H), 3.89 (s, 6H), 3.83 (s, 6H), 3.80 (s, 6H), 1.91 (s, 6H), 1.22 (t, *J* = 7.1 Hz, 6H). ^{13}C NMR (101 MHz, Chloroform-*d*) δ 175.2, 158.7, 158.7, 155.2, 136.9, 136.5, 133.0, 130.9, 130.6, 130.4, 129.7, 127.6, 113.5, 112.2, 110.6, 110.2, 89.8, 61.3, 56.0, 55.6, 55.3, 55.1, 27.3, 14.1. HRMS (m/z, ESI): Calcd. for Chemical Formula: $C_{54}H_{54}NaO_{10}^+$ $[M+Na]^+$: 885.3609, Found: 885.3610.

Under H_2 atmosphere, Pd/C (33 mg, 10 wt% in Carbon) and diethyl 2,2'-(ethyne-1,2-diylbis(4-methoxy-3,1-phenylene))-(2*S*,2'*S*)-bis(2-(4',6-dimethoxy-[1,1'-biphenyl]-3-yl)propanoate) **85** (55 mg, 0.064 mmol) were added 10.0 mL EA. Then the mixture was stirred overnight at 45°C. After that, the reaction solution was filtered with celite. The filtrate was washed with brine, dried over Na_2SO_4, concentrated in vacuo, and purified by flash column chromatography with hexane and acetone (5:1 – 2:1) to give the desired product **86**, white solid, 51 mg, 93%, $[\alpha]_D^{20}$ = +5.90 (*c* 1.000

$CHCl_3$) for 99.5:0.5 er. ^{1}H NMR (400 MHz, Chloroform-*d*) δ 7.46 – 7.41 (m, 4H), 7.20 (d, J = 2.5 Hz, 2H), 7.12 – 7.02 (m, 6H), 6.95 – 6.90 (m, 4H), 6.87 (d, J = 8.7 Hz, 2H), 6.74 (d, J = 8.5 Hz, 2H), 4.20 (qd, J = 7.1, 1.3 Hz, 4H), 3.83 (s, 6H), 3.80 (s, 6H), 3.76 (s, 6H), 2.83 (s, 4H), 1.87 (s, 6H), 1.23 (t, J = 7.1 Hz, 6H). ^{13}C NMR (101 MHz, Chloroform-*d*) δ 175.6, 158.6, 156.3, 155.1, 137.2, 136.2, 131.0, 130.7, 130.6, 130.2, 129.52, 129.49, 127.8, 126.4, 113.5, 110.5, 109.6, 61.1, 55.6, 55.30, 55.27, 55.2, 30.8, 27.5, 14.1. HRMS (m/z, ESI): Calcd. for Chemical Formula: $C_{54}H_{59}O_{10}^+$ $[M+H]^+$: 867.4103, Found: 867.4100. A sample of the ester was reduced to provide the parent primary alcohol for the HPLC analysis of the reaction product: Daicel Chiralpak IC, hexane/iso-propanol = 90: 10, 0.6 mL/min, λ= 258 nm, retention time: 48.21 min (major) and 56.13 min (minor).

C) Diethyl 2,2'-(6,6''-dimethoxy-[1,1':3',1''-terphenyl]-3,3''-diyl)-(2*S*,2'*S*)-bis(2-(3-chloro-4-meth -oxyphenyl)-propanoate) (88)

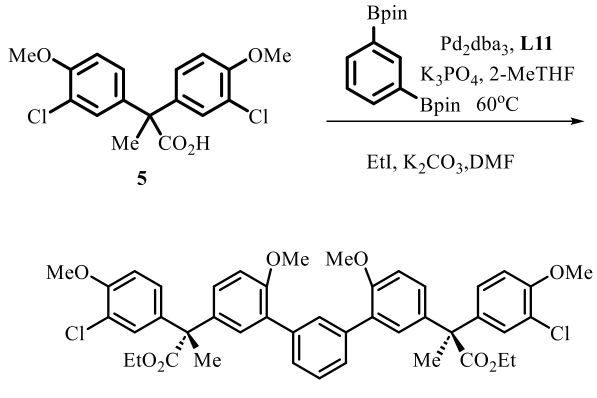

88

Under N_2 atmosphere, a mixture of $Pd_2(dba)_3$ (4.58 mg, 0.005 mmol) and **L11** (6.37 mg, 0.010 mmol) was added to 1.0 mL 2-MeTHF, and the mixture was stirred at room temperature for 20 min. The resulting metal-ligand complex solution was added to a reaction flask containing 9.0 mL 2-MeTHF solution of 2,2-bis(3-chloro-4-methoxyphenyl)-propanoic acid **5** (177 mg, 0.5 mmol) and 1,3-

Bis(4,4,5,5-tetramethyl-1,3,2-dioxaborolan-2-yl)-benzene **87** (54.5 mg, 0.165 mmol), and K_3PO_4 (530 mg, 2.50 mmol), followed by addition of 1.0 mL H_2O. Then the resulting reaction mixture was stirred at 60°C for 36 h. The reaction was then quenched with water, neutralized to pH 3 – 5 with 1.0 M HCl $_{(aq.)}$, and extracted with ethyl acetate (4.0 mL X 3) three times. The combined organic phase was washed with brine, dried over Na_2SO_4, and concentrated in vacuo. After that, the obtained crude product was dissolved in DMF (2.5 mL), and treated with EtI (390 mg, 2.5 mmol) and K_2CO_3 (414 mg, 3.0 mmol). The reaction mixture was stirred overnight at room temperature. Then the mixture was diluted with ethyl acetate (20 mL) and washed with water. The organic layer was concentrated in vacuo and purified by flash column chromatography with hexane and acetone (25:1 – 5:1) to give the desired product **88**, white solid, 92 mg, 72%, $[\alpha]_D^{20}$ = +9.55 (*c* 1.000 $CHCl_3$) for 99.5:0.5 er. ^{1}H NMR (400 MHz, Chloroform-*d*) δ 7.62 – 7.61 (m, 1H), 7.45 – 7.40 (m, 3H), 7.30 (d, *J* = 2.4 Hz, 2H), 7.22 (d, *J* = 2.6 Hz, 2H), 7.13 (td, *J* = 8.4, 2.5 Hz, 4H), 6.91 (d, *J* = 8.7 Hz, 2H), 6.85 (d, *J* = 8.7 Hz, 2H), 4.21 (q, *J* = 7.1 Hz, 4H), 3.89 (s, 6H), 3.80 (s, 6H), 1.90 (s, 6H), 1.22 (t, *J* = 7.1 Hz, 6H). ^{13}C NMR (101 MHz, Chloroform-*d*) δ 174.9, 155.4, 153.7, 138.0, 137.9, 136.4, 130.8, 130.6, 130.2, 129.9, 128.3, 127.9, 127.7, 127.5, 121.9, 111.5, 110.7, 61.4, 56.1, 55.6, 55.1, 27.2, 14.1. HRMS (m/z, ESI): Calcd. for Chemical Formula: $C_{44}H_{45}Cl_2O_8^+$ $[M+H]^+$: 771.2486, Found: 771.2484. HPLC analysis of the reaction product: Daicel Chiralpak IA, hexane/iso-propanol = 95: 5, 1.0 mL/min, λ= 288 nm, retention time: 15.98 min (major) and 21.98 min (minor).

D) Ethyl (*S*)-2-(3-chloro-4-methoxyphenyl)-2-(6-methoxy-3'-(4,4,5,5-tetramethyl-1,3,2-dioxabo-rolan-2-yl)-[1,1'-biphenyl]-3-yl)-propanoate (91)

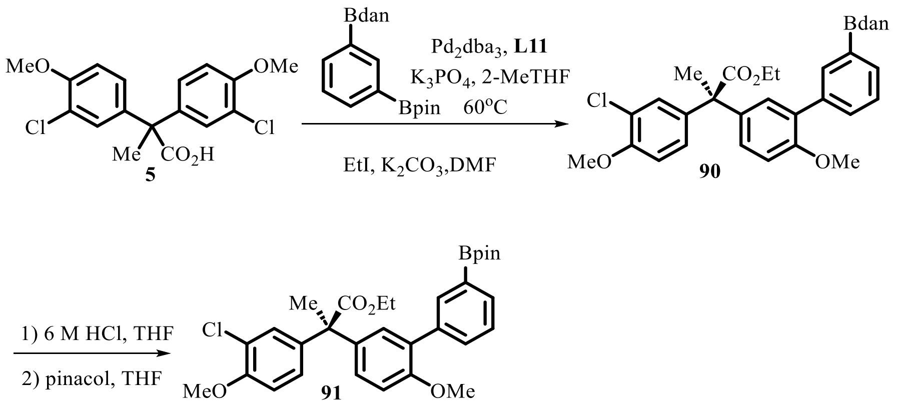

Under N_2 atmosphere, a mixture of $Pd_2(dba)_3$ (9.16 mg, 0.01 mmol) and **L11** (12.74 mg, 0.02 mmol) was added to 1.0 mL 2-MeTHF, the mixture was then stirred at room temperature for 20 min. The resulting metal-ligand complex solution was added to a reaction flask containing 9.0 mL 2-MeTHF solution of 2,2-bis(3-chloro-4-methoxyphenyl)propanoic acid **5** (354 mg, 1.0 mmol) and 2-(3-(4,4,5,5-tetramethyl-1,3,2-dioxaborolan-2-yl)phenyl)-2,3-dihydro-1H-naphtho[1,8-de][1,3,2]-diazaborinine **89** (444 mg, 1.2 mmol), and K_3PO_4 (1.06 g, 5.0 mmol), followed by addition of 1.0 mL H_2O. Then the resulting reaction mixture was stirred at 60°C for 18 h. The reaction was then quenched with water, neutralized to pH 4 – 6 with saturated KH_2PO_4 $_{(aq.)}$, and extracted with ethyl acetate (8.0 mL X 3) three times. The combined organic phase was washed with brine, dried over Na_2SO_4, and concentrated in vacuo. After that, the obtained crude products were dissolved in DMF (2.5 mL), and treated with EtI (468 mg, 3.0 mmol) and K_2CO_3 (690 mg, 5.0 mmol). The reaction mixture was stirred overnight at room temperature. Then the mixture was diluted with ethyl acetate (20 mL) and washed with water. The organic layer was concentrated in vacuo and purified by flash column chromatography with hexane and acetone (50:1 – 10:1) to give the desired product, ethyl (*S*)-2-(3-chloro-4-methoxyphenyl)-2-(6-methoxy-3'-(1H-naphtho[1,8-de][1,3,2]diazaborinin-2(3H)

-yl)-[1,1'-biphenyl]-3-yl)-propanoate **90**, white solid, 437 mg, 74% $[\alpha]_D^{20}$ = +1.03 (*c* 0.300 $CHCl_3$) for 95:5 er. ^{1}H NMR (500 MHz, Chloroform-*d*) δ 7.71 (t, *J* = 1.6 Hz, 1H), 7.61 – 7.56 (m, 2H), 7.47 (t, *J* = 7.5 Hz, 1H), 7.32 (d, *J* = 2.4 Hz, 1H), 7.21 – 7.12 (m, 5H), 7.06 (dd, *J* = 8.4, 1.0 Hz, 2H), 6.94 (dd, *J* = 8.0, 0.9 Hz, 1H), 6.87 (d, *J* = 8.6 Hz, 1H), 6.42 (dd, *J* = 7.3, 1.0 Hz, 2H), 6.05 (s, 2H), 4.23 (q, *J* = 7.1 Hz, 2H), 3.89 (s, 3H), 3.83 (s, 3H), 1.92 (s, 3H), 1.25 (t, *J* = 7.1 Hz, 3H). ^{13}C NMR (126 MHz, Chloroform-*d*) δ 174.9, 155.3, 153.7, 141.1, 138.4, 137.9, 136.5, 136.4, 132.6, 131.6, 130.5, 130.12, 130.09, 130.06, 128.2, 128.0, 127.6, 127.4, 121.9, 119.9, 117.8, 111.4, 110.7, 106.0, 61.5, 56.2, 55.7, 55.1, 27.2, 14.1. HRMS (m/z, ESI): Calcd. for Chemical Formula: $C_{35}H_{33}[11B]ClN_2O_4^+$ $[M+H]^+$: 591.2216, Found: 591.2226. HPLC analysis of the reaction product: Daicel Chiralpak IC, hexane/iso-propanol = 93: 7, 1.0 mL/min, λ= 272 nm, retention time: 25.95 min (major) and 23.03 min (minor).

Under air atmosphere, ethyl (*S*)-2-(3-chloro-4-methoxyphenyl)-2-(6-methoxy-3'-(1H-naphtho-[1,8-de]-[1,3,2]-diazaborinin-2(3H)-yl)-[1,1'-biphenyl]-3-yl)-propanoate **90** (437 mg, 0.74 mmol) was dissolved into 5.0 mL THF, and 6.0 M HCl $_{(aq.)}$ (6.1 mL) was added into the solution. After that, the reaction mixture was stirred overnight at room temperature. The reaction was then quenched with water and extracted with ethyl acetate (8.0 mL X 3) three times. The combined organic phase was washed with brine, dried over Na_2SO_4, and concentrated in vacuo. The crude product was dissolved into 5.0 mL THF, and pinacol (356 mg, 3.03 mmol) was added to the solution. After that, the reaction mixture was stirred overnight at room temperature. Then the mixture was diluted with ethyl acetate (20 mL) and washed with water. The organic layer was concentrated in vacuo and purified by flash column chromatography with hexane and acetone (100:1 – 20:1) to give the desired product **91**, yellowish oil, 270 mg, 66% over two steps, $[\alpha]_D^{20}$ = -4.55 (*c* 2.000 $CHCl_3$) for 94.5:5.5 er. ^{1}H NMR

(500 MHz, Chloroform-*d*) δ 7.87 (t, *J* = 1.7 Hz, 1H), 7.76 (dt, *J* = 7.4, 1.3 Hz, 1H), 7.60 – 7.57 (m, 1H), 7.42 – 7.37 (m, 1H), 7.29 (d, *J* = 2.4 Hz, 1H), 7.19 (d, *J* = 2.6 Hz, 1H), 7.13 – 7.09 (m, 2H), 6.89 (d, *J* = 8.7 Hz, 1H), 6.85 (d, *J* = 8.7 Hz, 1H), 4.25 – 4.19 (m, 2H), 3.89 (s, 3H), 3.79 (s, 3H), 1.89 (s, 3H), 1.34 (s, 12H), 1.25 (t, *J* = 7.1 Hz, 3H). ^{13}C NMR (126 MHz, Chloroform-*d*) δ 174.9, 155.4, 153.6, 138.1, 137.9, 136.1, 135.7, 133.5, 132.6, 130.5, 130.3, 130.0, 128.0, 127.5, 127.3, 121.9, 111.4, 110.6, 83.7, 61.4, 56.1, 55.6, 55.1, 27.3, 24.9, 14.1. HRMS (m/z, ESI): Calcd. for Chemical Formula: $C_{31}H_{36}$[11B]$ClNaO_6^+$ $[M+Na]^+$: 573.2186, Found: 573.2193. HPLC analysis of the reaction product: Daicel Chiralpak IA, hexane/iso-propanol = 99: 1, 1.0 mL/min, λ= 287 nm, retention time: 9.68 min (major) and 10.73 min (minor).

Triethyl (4*r*,8*r*,12*r*)-1^6,3^6,5^4,7^6,9^4,11^6-hexamethoxy-4,8,12-trimethyl-1,2,3,5,6,7,9,10,11(1,3)-nonabenzenacyclododecaphane-4,8,12-tricarboxylate (93) and oligomerization (92)

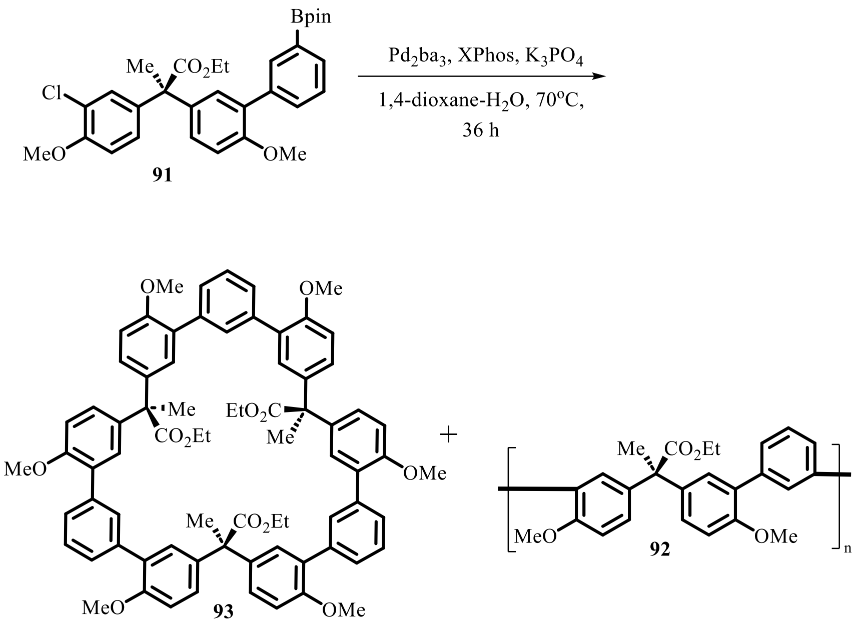

91, **93**, **92**

Under N_2 atmosphere, a mixture of $Pd_2(dba)_3$ (1.14 mg, 0.00125 mmol) and XPhos (2.38 mg, 0.005 mmol) was added to 0.2 mL 1,4-dioxane, the mixture was then stirred at room temperature for 20 min. The resulting metal-ligand complex solution was added to a reaction flask containing ethyl (*S*)-2-(3-chloro-4-methoxyphenyl)-2-(6-methoxy-3'-(4,4,5,5-tetramethyl-1,3,2-dioxabo-rolan-2-yl)-[1,1'-biphenyl]-3-yl)-propanoate **91** (21.2 mg, 0.05 mmol) and K_3PO_4 (53 mg, 0.25 mmol), followed by addition of 1,4-dioxane (0.3 mL) and H_2O (0.10 mL). Then the resulting reaction mixture was stirred at 70°C for 36 h. The reaction was then quenched with water, neutralized to pH 4 – 6 with saturated KH_2PO_4 $_{(aq.)}$, and extracted with ethyl acetate (1.5 mL X 3) three times. The combined organic phase was washed with brine, dried over Na_2SO_4, and concentrated in vacuo. The organic layer was concentrated in vacuo and purified by PLC with hexane and acetone (3:1) to give the desired product **93**, Triethyl (4*r*,8*r*,12*r*)-1^6,3^6,5^4,7^6,9^4,11^6-hexamethoxy-4,8,12-trimethyl-1,2,3,5,6,7,9,10,11(1,3)-nonabenzenacyclododecaphane-4,8,12-tricarboxylate, white solid, 2.3 mg,

4%. $[\alpha]_D^{20}$ = - 1.96 (*c* 0.230 $CHCl_3$). ^{1}H NMR (400 MHz, Chloroform-*d*) δ 7.47 (t, *J* = 1.8 Hz, 3H), 7.38 (dd, *J* = 7.3, 1.8 Hz, 6H), 7.30 – 7.27 (m, 3H), 7.22 – 7.16 (m, 12H), 6.87 (d, *J* = 8.5 Hz, 6H), 4.16 (q, *J* = 7.2 Hz, 6H), 3.74 (s, 18H), 1.90 (s, 9H), 1.16 (t, *J* = 7.1 Hz, 9H). ^{13}C NMR (126 MHz, Chloroform-*d*) δ 175.4, 155.2, 138.2, 136.7, 130.8, 130.6, 130.1, 128.2, 127.9, 127.3, 110.5, 61.2, 55.5, 29.7, 27.3, 14.1. HRMS (m/z, ESI): Calcd. for Chemical Formula: $C_{75}H_{73}O_{12}^+$ $[M+H]^+$: 1165.5097, Found: 1165.5094.

After the oligomerization, the precipitation purification gave a yellowish solid **92**, 12 mg, 62%. ^{1}H NMR (500 MHz, Chloroform-*d*) δ 7.68 – 7.61 (m, 1H), 7.43 – 7.35 (m, 3H), 7.31 – 7.30 (m, 2H), 7.20 – 7.15 (m, 2H), 6.89 – 6.87 (m, 2H), 4.21 – 4.17 (m, 2H), 3.79 – 3.74 (m, 6H), 1.93– 1.90 (m, 3H), 1.22 – 1.18 (m, 3H).

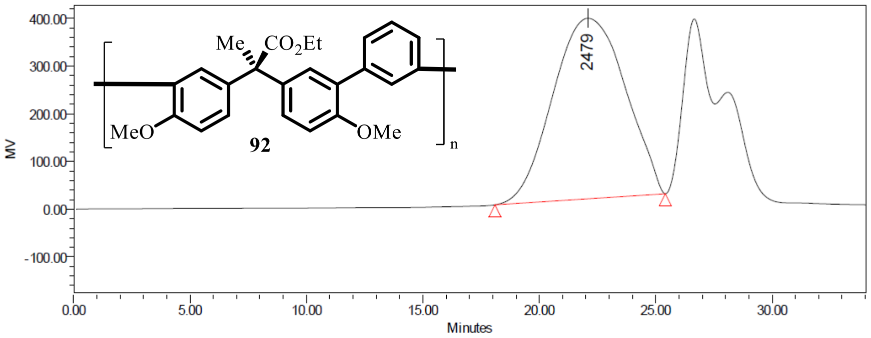

GPC Results

	Retention Time (min)	Mn	Mw	MP	Mz	Mz+1	Mv	Polydispersity
1	22.096	1966	3093	2479	4646	6339		1.572820

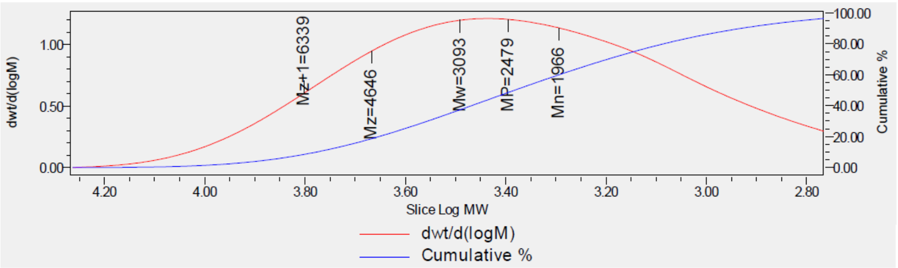

Figure S5. Gel permeation chromatography (GPC) for measuring the molecular weight of **92**

E) Dodecyl (*S*)-2-(3-chloro-4-methoxyphenyl)-2-(6-methoxy-4'-(4,4,5,5-tetramethyl-1,3,2-dioxa- borolan-2-yl)-[1,1'-biphenyl]-3-yl)-propanoate(96)

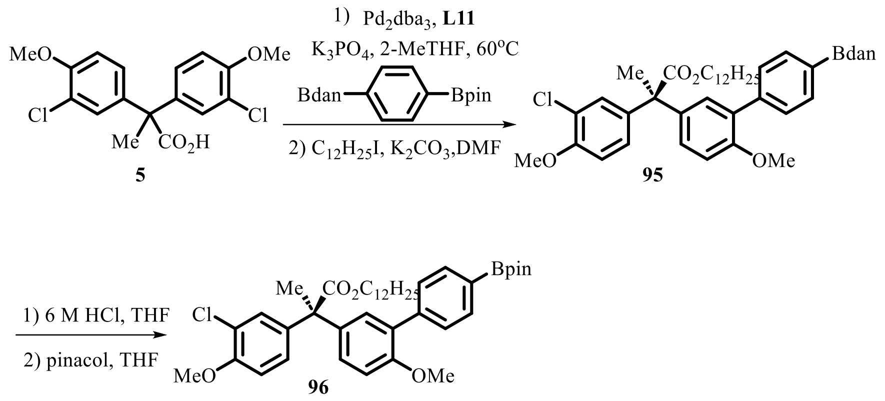

Under N_2 atmosphere, a mixture of $Pd_2(dba)_3$ (9.16 mg, 0.010 mmol) and **L11** (12.74 mg, 0.020 mmol) was added to 1.0 mL 2-MeTHF, the mixture was then stirred at room temperature for 20 min. The resulting metal-ligand complex solution was added to a reaction flask containing 9.0 mL 2-MeTHF solution of 2,2-bis(3-chloro-4-methoxyphenyl)propanoic acid **5** (354 mg, 1.0 mmol) and 2-(4-(4,4,5,5-tetramethyl-1,3,2-dioxaborolan-2-yl)phenyl)-2,3-dihydro-1H-naphtho[1,8-de][1,3,2] -di-azaborinine **94** (444 mg, 1.20 mmol), and K_3PO_4 (1.06 g, 5.0 mmol), followed by addition of 1.0 mL H_2O. Then the resulting reaction mixture was stirred at 60°C for 18 h. The reaction was then quenched with water, neutralized to pH 4 – 6 with saturated $KH_2PO_{4\,(aq.)}$, and extracted with ethyl acetate (15 mL X 3) three times. The combined organic phase was washed with brine, dried over Na_2SO_4, and concentrated in vacuo. After that, the obtained crude product was dissolved in DMF (2.5 mL) and treated with 1-iodododecane (888 mg, 3.0 mmol) and K_2CO_3 (690 mg, 5.0 mmol). The reaction mixture was stirred for 24 h at room temperature. Then the mixture was diluted with ethyl acetate (20 mL) and washed with water. The organic layer was concentrated in vacuo and purified by flash column chromatography with hexane and acetone (100:1 – 20:1) to give the desired product **95**, dodecyl (*S*)-2-(3-chloro-4-methoxyphenyl)-2-(6-methoxy-4'-(1H-naphtho[1,8-de]

[1,3,2] diaza- borinin-2(3H)-yl)-[1,1'-biphenyl]-3-yl)-propanoate, brownish red oil, 420 mg, 57%, $[\alpha]_D^{20}$ = +1.50 (*c* 0.200 $CHCl_3$) for 95:5 er. ^{1}H NMR (400 MHz, Chloroform-*d*) δ 7.67 (d, *J* = 8.2 Hz, 2H), 7.58 – 7.54 (m, 2H), 7.31 (d, *J* = 2.4 Hz, 1H), 7.20 – 7.13 (m, 4H), 7.13 – 7.11 (m, 1H), 7.06 (dd, *J* = 8.3, 1.0 Hz, 2H), 6.93 (d, *J* = 8.5 Hz, 1H), 6.86 (d, *J* = 8.7 Hz, 1H), 6.42 (dd, *J* = 7.2, 1.1 Hz, 2H), 6.05 (s, 2H), 4.14 (t, *J* = 6.6 Hz, 2H), 3.90 (s, 3H), 3.83 (s, 3H), 1.90 (s, 3H), 1.26 – 1.21 (m, 20H), 0.89 – 0.85 (m, 3H). ^{13}C NMR (101 MHz, Chloroform-*d*) δ 174.9, 155.3, 153.7, 141.1, 140.5, 137.8, 136.6, 136.4, 131.1, 130.5, 130.0, 129.8, 129.4, 128.3, 127.6, 127.4, 121.9, 117.8, 111.4, 110.8, 106.0, 65.6, 56.1, 55.6, 55.2, 31.9, 29.69, 29.67, 29.61, 29.5, 29.4, 29.2, 28.5, 27.2, 25.9, 22.7, 14.1. HRMS (m/z, ESI): Calcd. for Chemical Formula: $C_{45}H_{53}[11B]ClN_2O_4^+$ $[M+H]^+$: 731.3781, Found: 731.3784. HPLC analysis of the reaction product: Daicel Chiralpak IA, hexane/iso-propanol = 90: 10, 1.0 mL/min, λ= 262 nm, retention time: 22.65 min (major) and 15.46 min (minor).

Under air atmosphere, dodecyl (*S*)-2-(3-chloro-4-methoxyphenyl)-2-(6-methoxy-4'-(1H-naphtho[1,8-de] [1,3,2] diazaborinin-2(3H)-yl)-[1,1'-biphenyl]-3-yl)-propanoate **95** (420 mg, 0.57 mmol) was dissolved into 5.0 mL THF, and 6.0 M HCl $_{(aq.)}$ (5.8 mL) was added into the solution. After that, the reaction mixture was stirred overnight at room temperature. The reaction was then quenched with water and extracted with ethyl acetate (12 mL X 3) three times. The combined organic phase was washed with brine, dried over Na_2SO_4, and concentrated in vacuo. The crude product was dissolved into 5.0 mL THF, and pinacol (339 mg, 2.87 mmol) was added to the solution. After that, the reaction mixture was stirred overnight at room temperature. Then the mixture was diluted with ethyl acetate (30 mL) and washed with water. The organic layer was concentrated in vacuo and purified by flash column chromatography with hexane and acetone (100:1 – 20:1) to give

the desired product **96**, brownish red oil, 273 mg, 85% over two steps, $[\alpha]_D^{20}$ = -3.05 (*c* 1.000 $CHCl_3$) for 95:5 er. ^{1}H NMR (400 MHz, Chloroform-*d*) δ 7.83 (d, *J* = 7.5 Hz, 2H), 7.50 – 7.46 (m, 2H), 7.30 (d, *J* = 2.4 Hz, 1H), 7.17 – 7.09 (m, 3H), 6.90 (d, *J* = 8.5 Hz, 1H), 6.84 (d, *J* = 8.7 Hz, 1H), 4.13 (t, *J* = 6.6 Hz, 2H), 3.89 (s, 3H), 3.79 (s, 3H), 1.89 (s, 3H), 1.35 (s, 12H), 1.31 – 1.11 (m, 20H), 0.88 (t, *J* = 6.9 Hz, 3H). ^{13}C NMR (101 MHz, Chloroform-*d*) δ 174.9, 155.3, 153.7, 141.4, 137.9, 136.5, 134.4, 130.4, 130.1, 130.0, 128.9, 128.2, 127.4, 121.9, 111.4, 110.8, 83.7, 65.6, 56.1, 55.6, 55.2, 31.9, 29.7, 29.64, 29.57, 29.5, 29.4, 29.2, 28.5, 27.2, 25.9, 24.9, 22.7, 14.1. HRMS (m/z, ESI): Calcd. for Chemical Formula: $C_{41}H_{56}$[11B]$ClNaO_6^+$ $[M+Na]^+$: 713.3751, Found: 713.3760. HPLC analysis of the reaction product: Daicel Chiralpak IA, hexane/iso-propanol = 99: 1, 0.8 mL/min, λ= 286 nm, retention time: 8.86 min (major) and 9.69 min (minor).

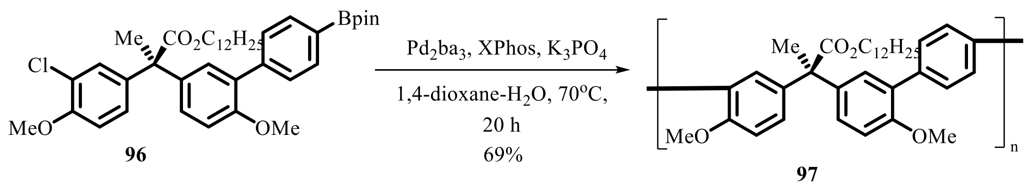

96 **97**

Under N_2 atmosphere, a mixture of $Pd_2(dba)_3$ (1.14 mg, 0.00125 mmol) and XPhos (2.38 mg, 0.005 mmol) was added to 0.05 mL 1,4-dioxane, the mixture was then stirred at room temperature for 20 min. The resulting metal-ligand complex solution was added to a reaction flask containing dodecyl (*S*)-2-(3-chloro-4-methoxyphenyl)-2-(6-methoxy-4'-(4,4,5,5-tetramethyl-1,3,2-dioxa- borolan-2-yl)-[1,1'-biphenyl]-3-yl)-propanoate **96** (28.2 mg, 0.05 mmol) and K_3PO_4 (53 mg, 0.25 mmol), followed by addition of 1,4-dioxane (0.05 mL) and H_2O (0.10 mL). Then the resulting reaction mixture was stirred at 70°C for 20 h. The reaction was then quenched with water, neutralized to pH 4 – 6 with saturated $KH_2PO_{4\,(aq.)}$, and extracted with ethyl acetate (1.5 mL X 3) three times. The combined organic phase was washed with brine, dried over Na_2SO_4, and

concentrated in vacuo. The organic layer was concentrated in vacuo and purified by PLC with hexane and acetone (3:1) to give the desired product **97**, 18 mg, 69%, yellowish solid. ^{1}H NMR (500 MHz, Chloroform-*d*) δ 7.57 – 7.47 (m, 4H), 7.34 – 7.28 (m, 2H), 7.19 – 7.17 (m, 2H), 6.91 – 6.89 (m, 2H), 4.16 – 4.13 (m, 2H), 3.82 – 3.81 (m, 6H), 1.94 – 1.93 (m, 3H), 1.27 – 1.19 (m, 20H), 0.87 – 0.84 (m, 3H).

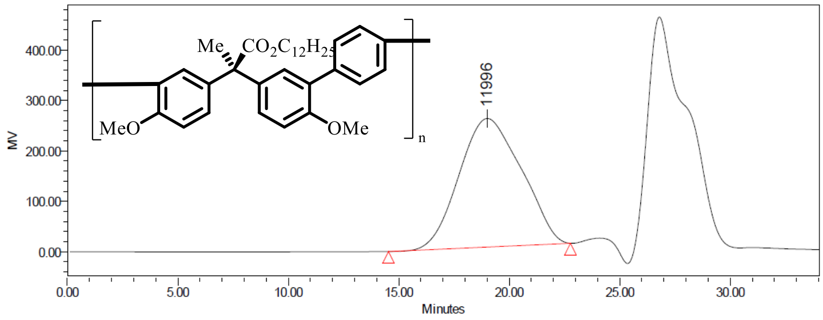

GPC Results

	Retention Time (min)	Mn	Mw	MP	Mz	Mz+1	Mv	Polydispersity
1	19.012	8978	12907	11996	17747	23021		1.437563

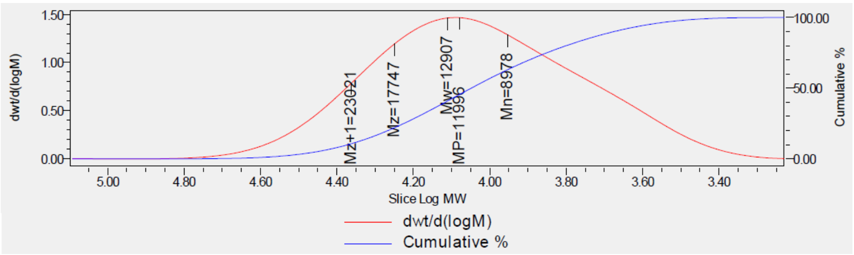

Figure S6. Gel permeation chromatography (GPC) for measuring the molecular weight of **97**

References

1. Lou, Y. Z., Wei, J. Q., Li, M. F., Zhu, Y.,*J. Am. Chem. Soc.* **2022**, *144*, 123−129.
2. Li, M. F., Chia, X. L., Tian, C., Zhu, Y., *Chem* **2022**, *8*, 2843–2855.
3. On, K. W. I, Hong, W. Y., Zhu, Y. *Chem. Catal.* **2023**, *3*, 100523.
4. Kim, B.; Chinn, A. J.; Fandrick, D. R.; Senanayake, C. H.; Singer, R. A.; Miller, S. J. *J. Am. Chem. Soc.* **2016**, *138*, 7939−7945.
5. Shi, B. F., Zhang, Y. H., Lam, J. K., Wang, D. H., Yu, J. Q., *J. Am. Chem. Soc.* **2010**, *132*, 460–461.
6. X. Shao, X. Jiang, X. Zhao, C. Zhao, Y. Chen and Z. Li, *J. Org. Chem.* **2004**, *69*, 899.
7. Fleckenstein, C. A.; Plenio, H. *Chem. Eur. J.* **2007**, *13*, 2701.
8. Cheng, X. F., Fei, F., Li, Y., Hou, Y. M., Zhou, X., Wang, X. S. *Org. Lett.* **2020**, *22*, 6394–6398.
9. Bergman, J., Janosik, T., Koch, E., Pelcman, B., *J. Chem. Soc.*, *Perkin Trans. 1*, **2000**, 2615–2621.
10. Jaquith, James B. Synthesis of angiotensin-converting enzyme (ACE) inhibitors using dynamic kinetic resolution. Synthesis of the highly methylated tryptophan residue of hemiasterlin using glycidic ester ring opening reactions. Synthesis of benz(o)indenes, **1999**, *61*, 203.
11. Gullickson, G. C., Lewis, D. E., *Synthesis*, **2003**, 681–684.
12. Kolasa, T., Gunn, D. E., Bhatia, P., Basha, A., Craig, R. A., Stewart, A. O., Bouska, J. B., Harris, R. R., Hulkower, K. I., E. Malo, P. E., Bell, R. L., Carter, G. W., Brooks, C. B. *J. Med. Chem.* **2000**, *43*, 3322–3334.
13. Jeongju, M., Sunwoo, L. *J. Organomet. Chem*. **2009**, *694*, 473–477.

14. Granados, A., Jia, Z. Y., Olmo, M. D., Vallribera, A. *Eur. J. Org. Chem.* **2019**, 2812–2818.

15. Gaussian 16, Revision C.01, Frisch, M. J.; Trucks, G. W.; Schlegel, H. B.; Scuseria, G. E.; Robb, M. A.; Cheeseman, J. R.; Scalmani, G.; Barone, V.; Petersson, G. A.; Nakatsuji, H.; Li, X.; Caricato, M.; Marenich, A. V.; Bloino, J.; Janesko, B. G.; Gomperts, R.; Mennucci, B.; Hratchian, H. P.; Ortiz, J. V.; Izmaylov, A. F.; Sonnenberg, J. L.; Williams-Young, D.; Ding, F.; Lipparini, F.; Egidi, F.; Goings, J.; Peng, B.; Petrone, A.; Henderson, T.; Ranasinghe, D.; Zakrzewski, V. G.; Gao, J.; Rega, N.; Zheng, G.; Liang, W.; Hada, M.; Ehara, M.; Toyota, K.; Fukuda, R.; Hasegawa, J.; Ishida, M.; Nakajima, T.; Honda, Y.; Kitao, O.; Nakai, H.; Vreven, T.; Throssell, K.; Montgomery, J. A., Jr.; Peralta, J. E.; Ogliaro, F.; Bearpark, M. J.; Heyd, J. J.; Brothers, E. N.; Kudin, K. N.; Staroverov, V. N.; Keith, T. A.; Kobayashi, R.; Normand, J.; Raghavachari, K.; Rendell, A. P.; Burant, J. C.; Iyengar, S. S.; Tomasi, J.; Cossi, M.; Millam, J. M.; Klene, M.; Adamo, C.; Cammi, R.; Ochterski, J. W.; Martin, R. L.; Morokuma, K.; Farkas, O.; Foresman, J. B.; Fox, D. J. Gaussian, Inc., Wallingford CT, 2016.

16. Peverati, R.; Truhlar, D. G. *J. Phys. Chem. Lett*. **2012**, *3*, 117–124.

17. Hay, P. J.; Wadt, W. R. *J. Chem. Phys*. **1985**, *82*, 299–310.

18. Marenich, A. V.; Cramer, C. J.; Truhlar, D. G. *J. Phys. Chem. B*, **2009**, *113*, 6378–6396.

19. Hay, P. J.; Wadt, W. R. *J. Chem. Phys*. **1985**, *82*, 299–310.

20. Barder, T. E.; Biscoe, M. R.; Buchwald, S. L. *Organometallics*, **2007**, *26*, 2183–2192.

NMR spectra

L6-(*R*)-2'-(dicyclohexylphosphaneyl)-2,6-diisopropoxy-5-methyl-[1,1'-biphenyl]-3-carboxylic acid

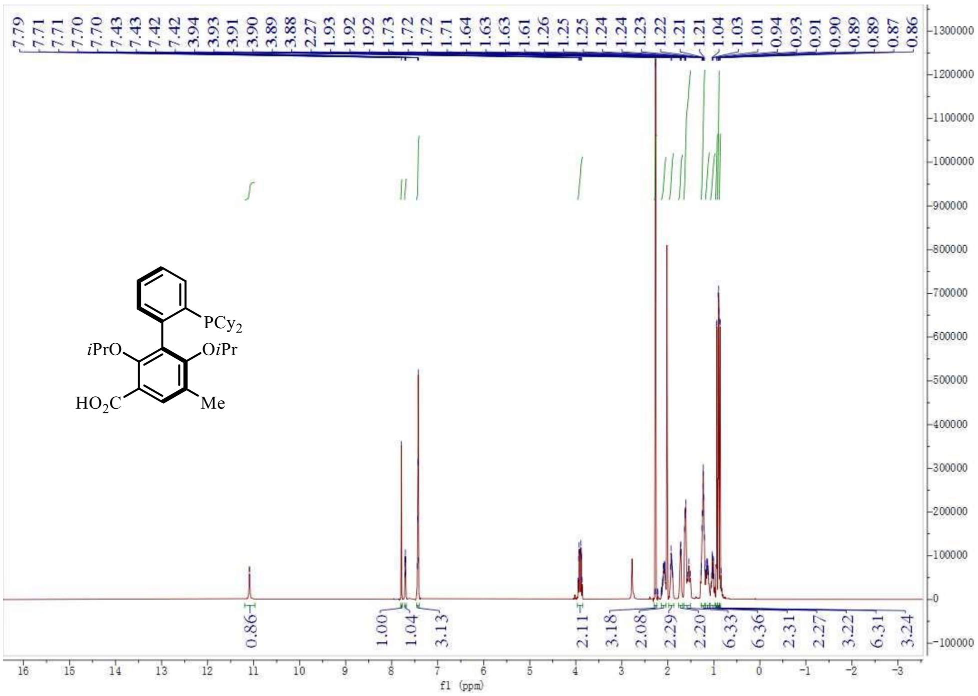

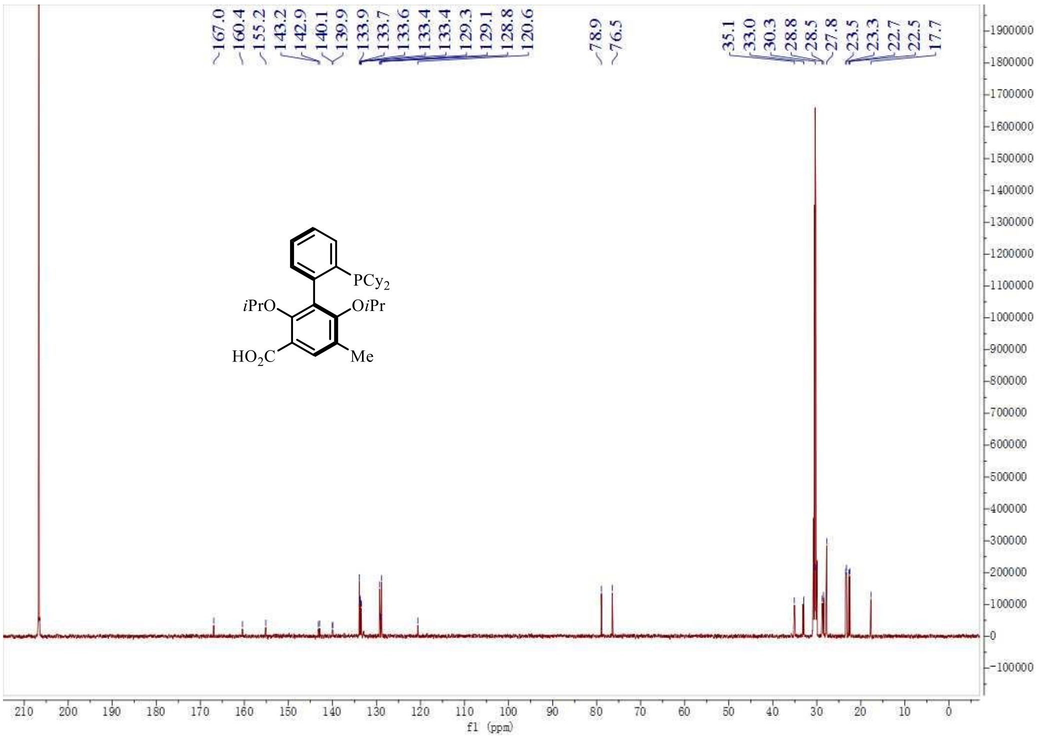

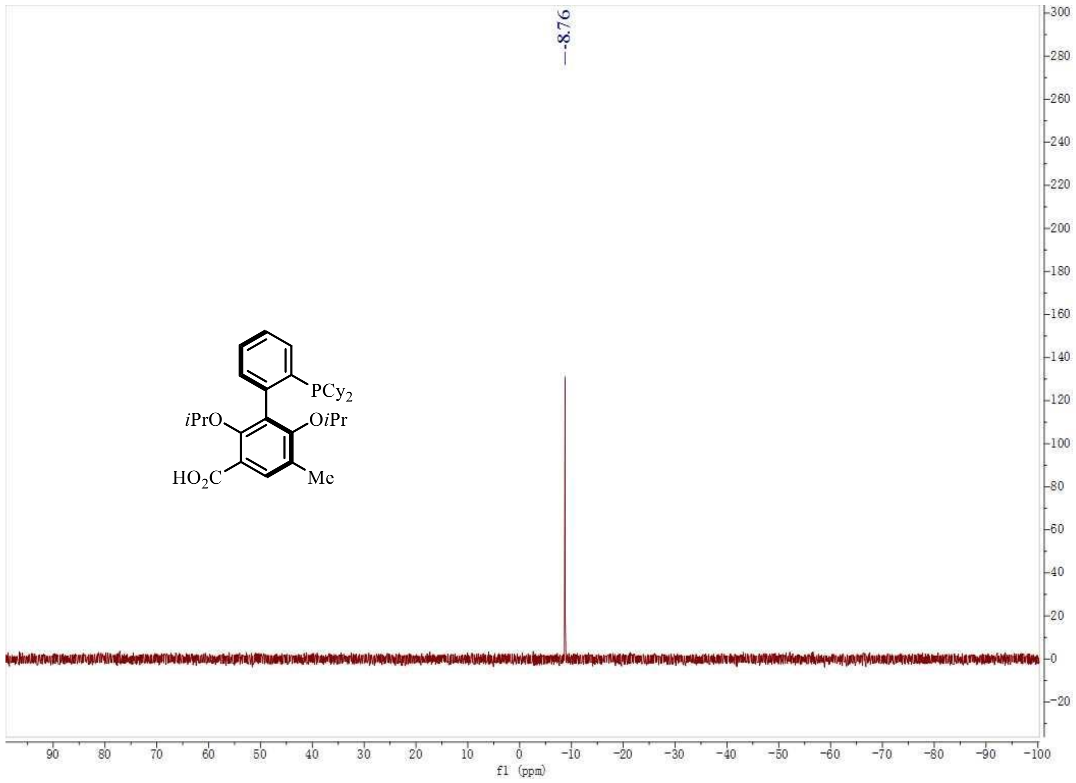

-8.76
PCy2
iPrO
OiPr
HO2C
Me
f1 (ppm)

L7-(*R*)(2'-(dicyclohexylphosphaneyl)-2,6-diisopropoxy-5-methyl-[1,1'-biphenyl]-3-carbonyl)glycine

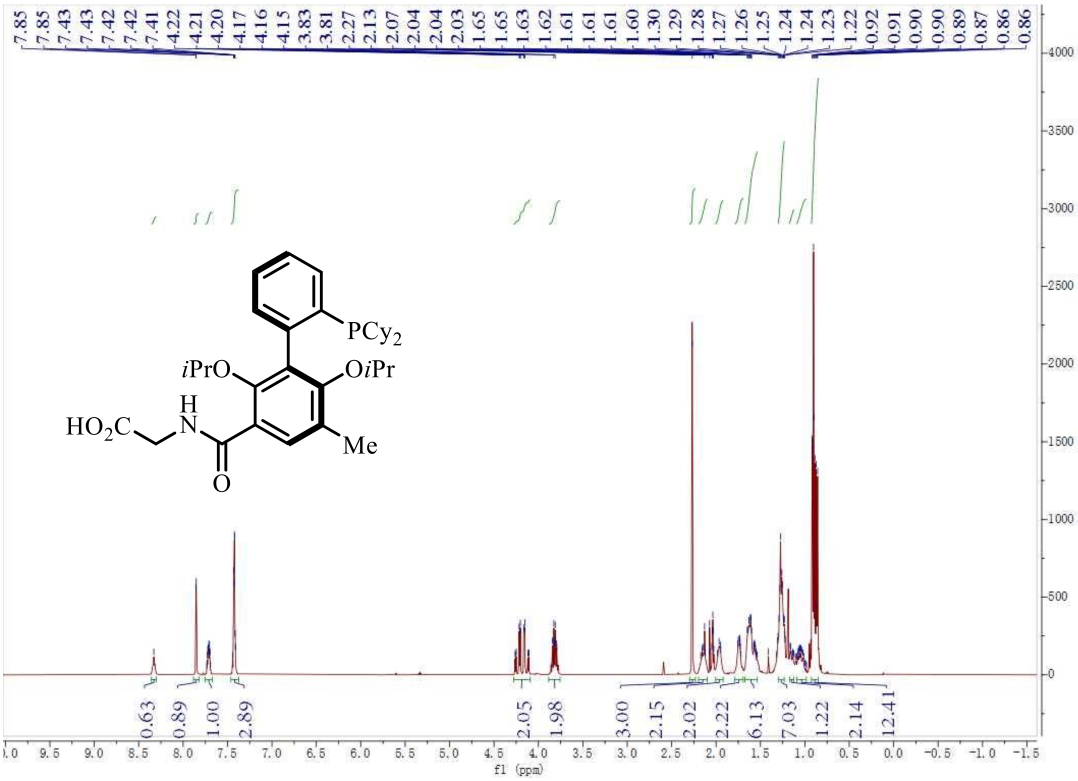

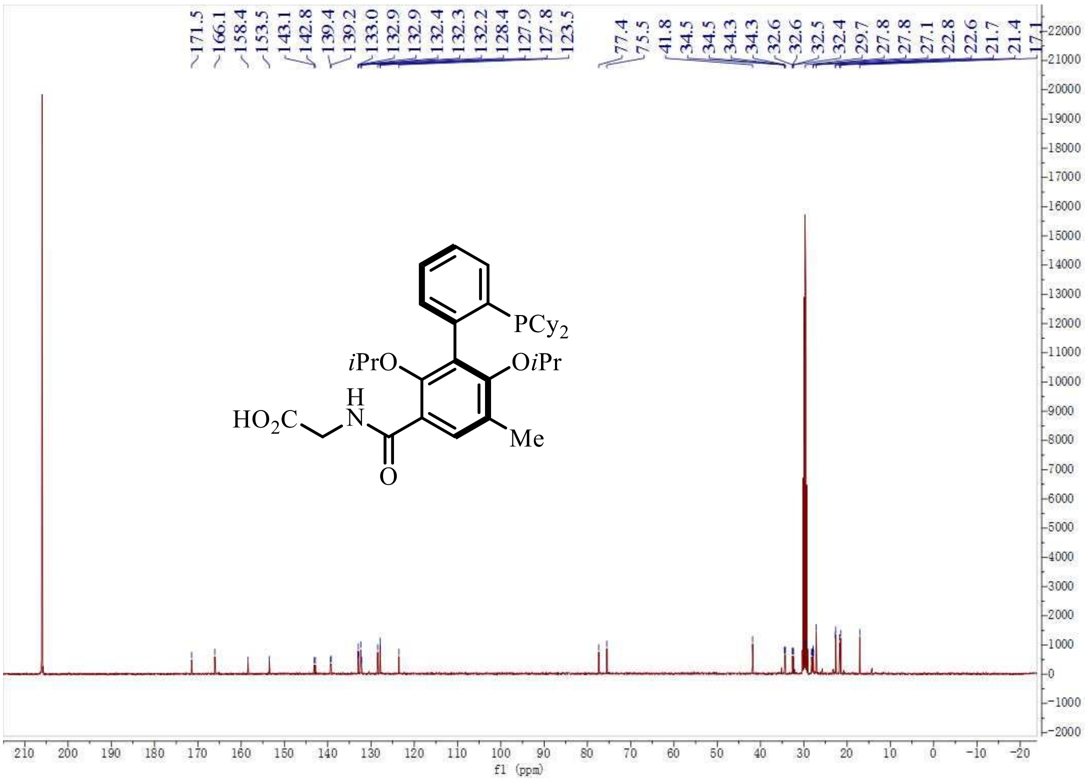

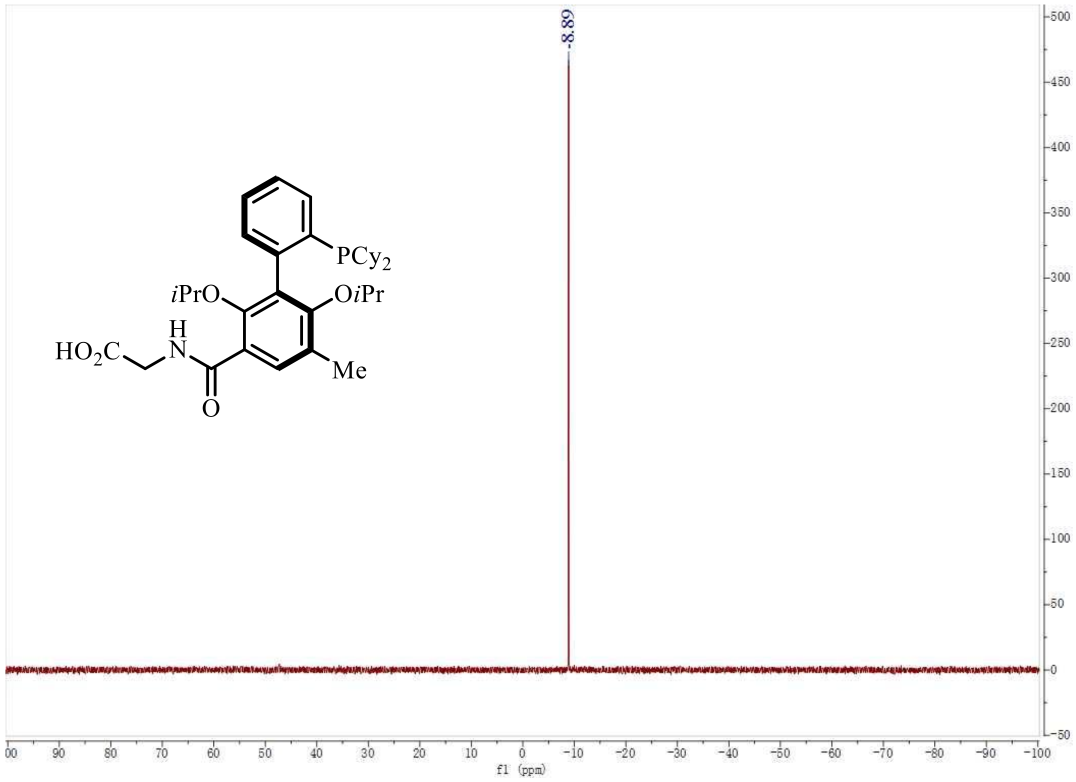
-8.89
PCy2
iPrO
OiPr
H
N
HO2C
Me
O
500
450
400
350
300
250
200
150
100
50
0
−50
00
90
80
70
60
50
40
30
20
10
0
−10
−20
−30
−40
−50
−60
−70
−80
−90
−100
f1 (ppm)

L8-(*R*)-(2'-(dicyclohexylphosphaneyl)-2,6-diisopropoxy-5-methyl-[1,1'-biphenyl]-3-carbonyl)-L-alanine

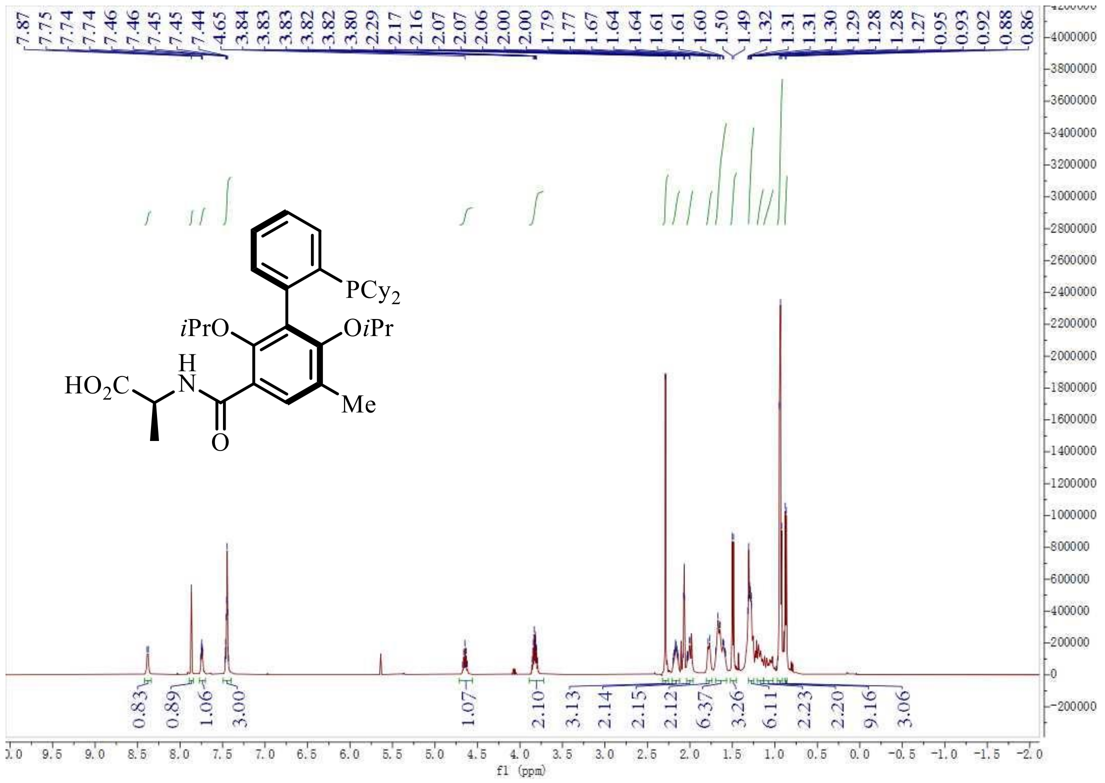

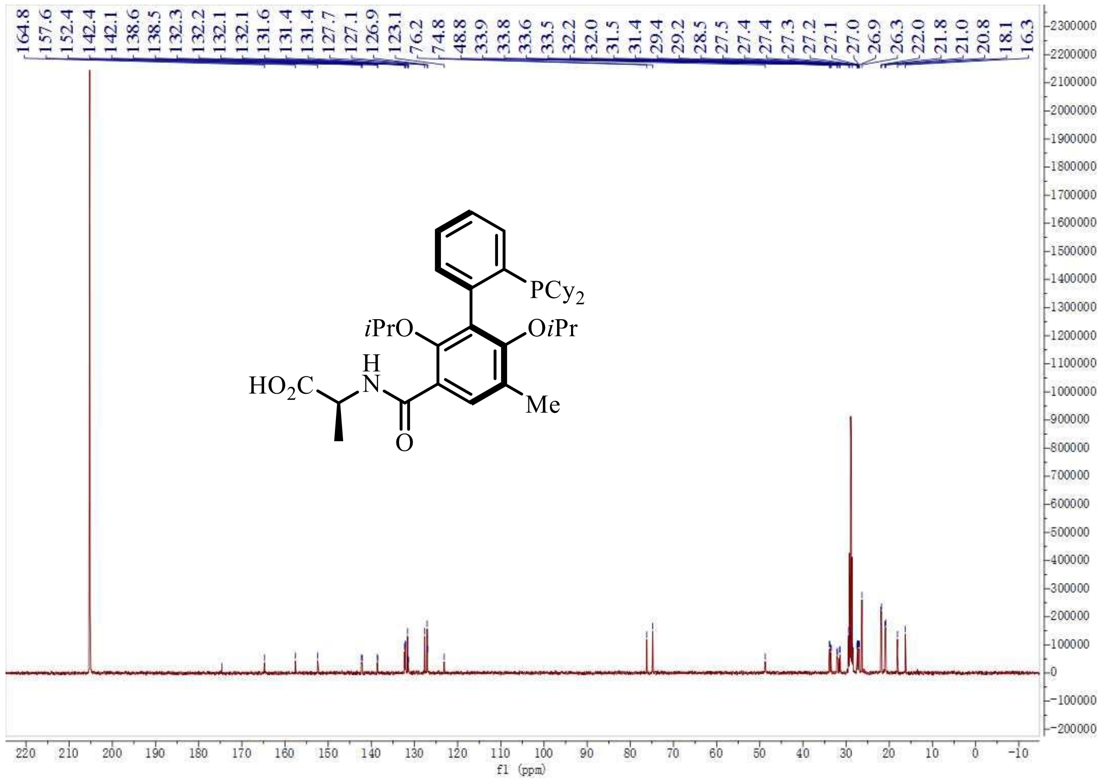

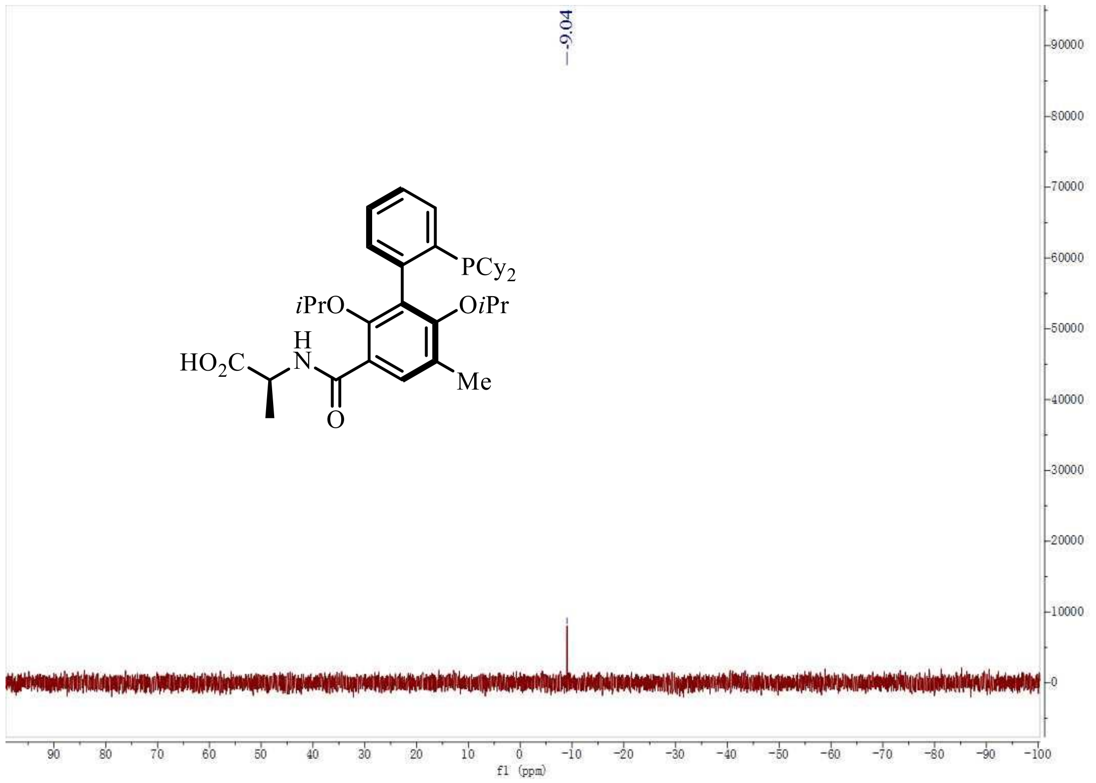
-9.04
PCy2
iPrO
OiPr
H
N
HO2C
Me
O
90000
80000
70000
60000
50000
40000
30000
20000
10000
0
90 80 70 60 50 40 30 20 10 0 -10 -20 -30 -40 -50 -60 -70 -80 -90 -100
f1 (ppm)

L9-(*R*)-2'-(dicyclohexylphosphaneyl)-2,6-diisopropoxy-5-methyl-[1,1'-biphenyl]-3-carbonyl)-

L-valine

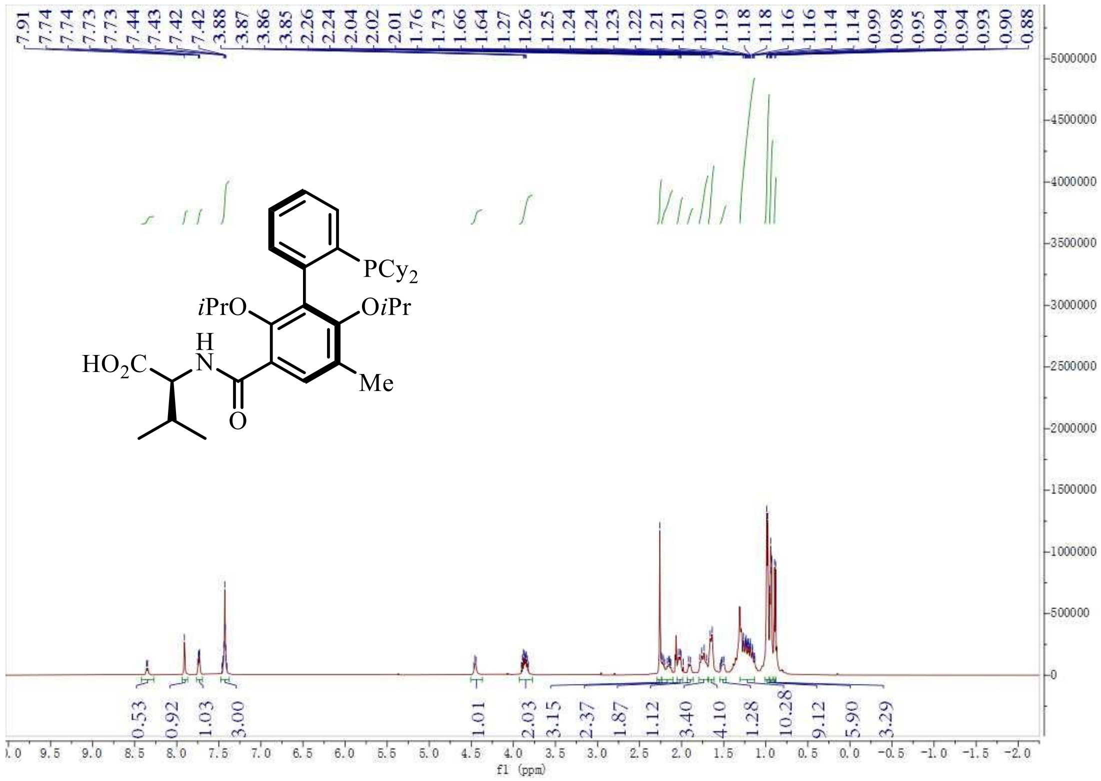

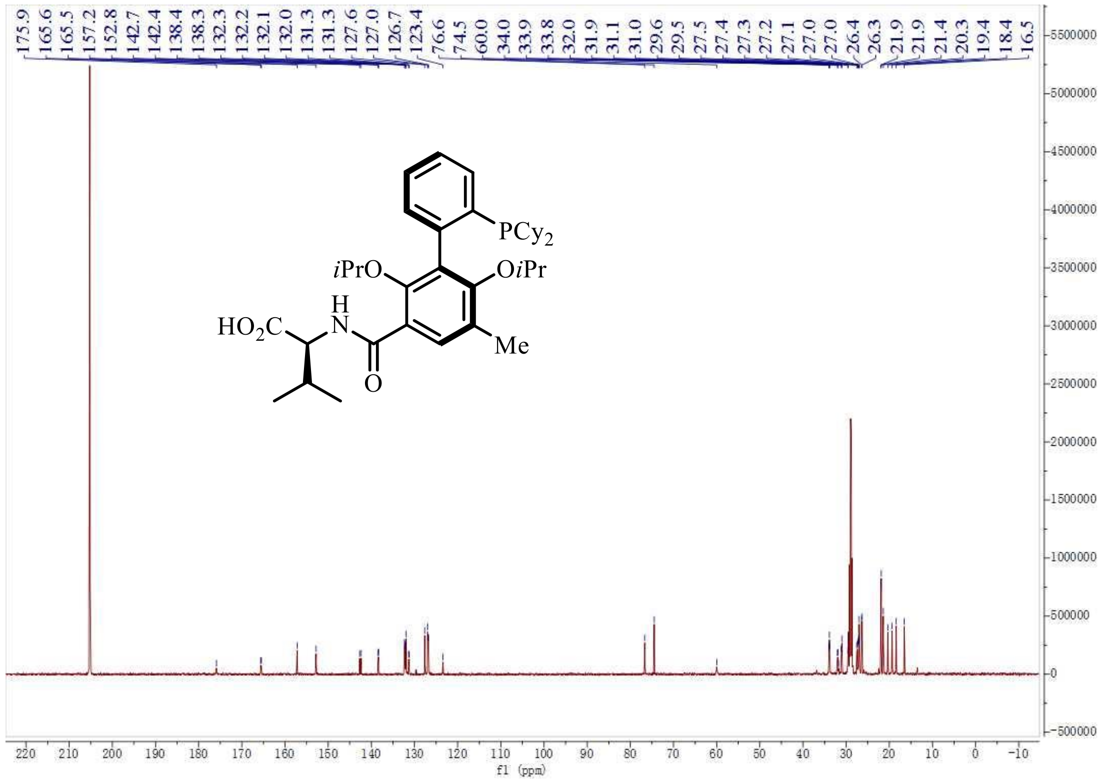

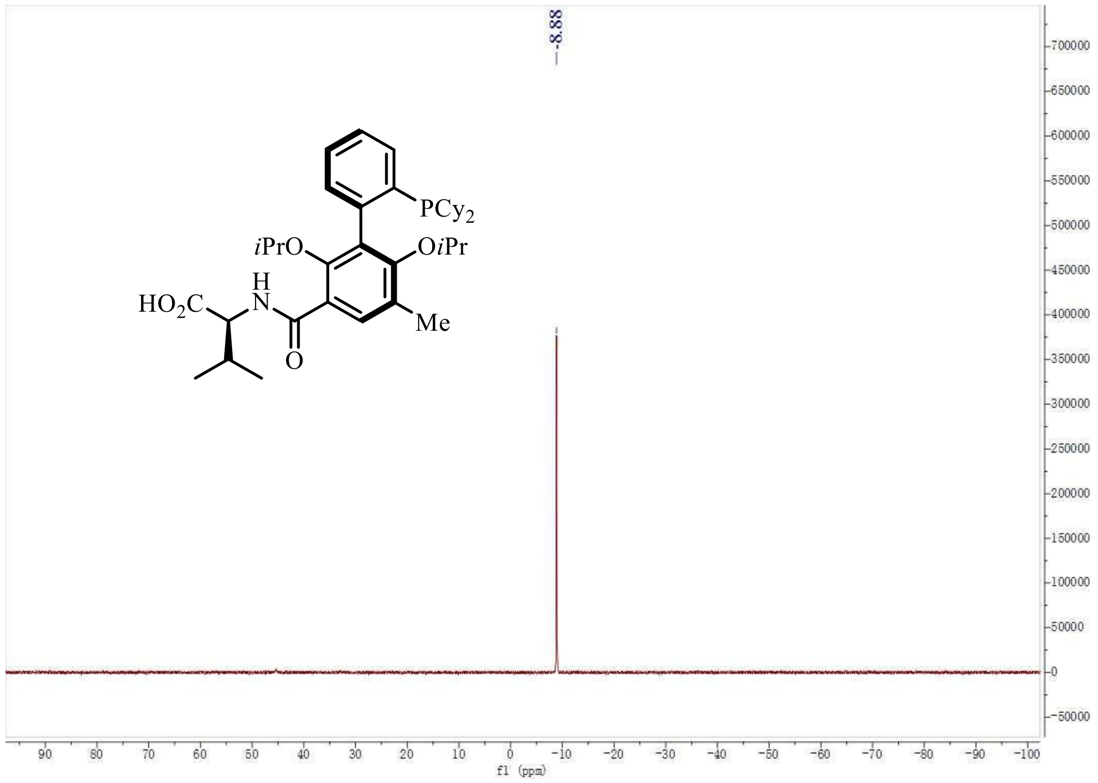

-8.88
PCy2
iPrO
OiPr
H
N
HO2C
O
Me
f1 (ppm)

L10-(*R*)-2'-(dicyclohexylphosphaneyl)-2,6-diisopropoxy-5-methyl-[1,1'-biphenyl]-3-carbonyl-L-isoleucine

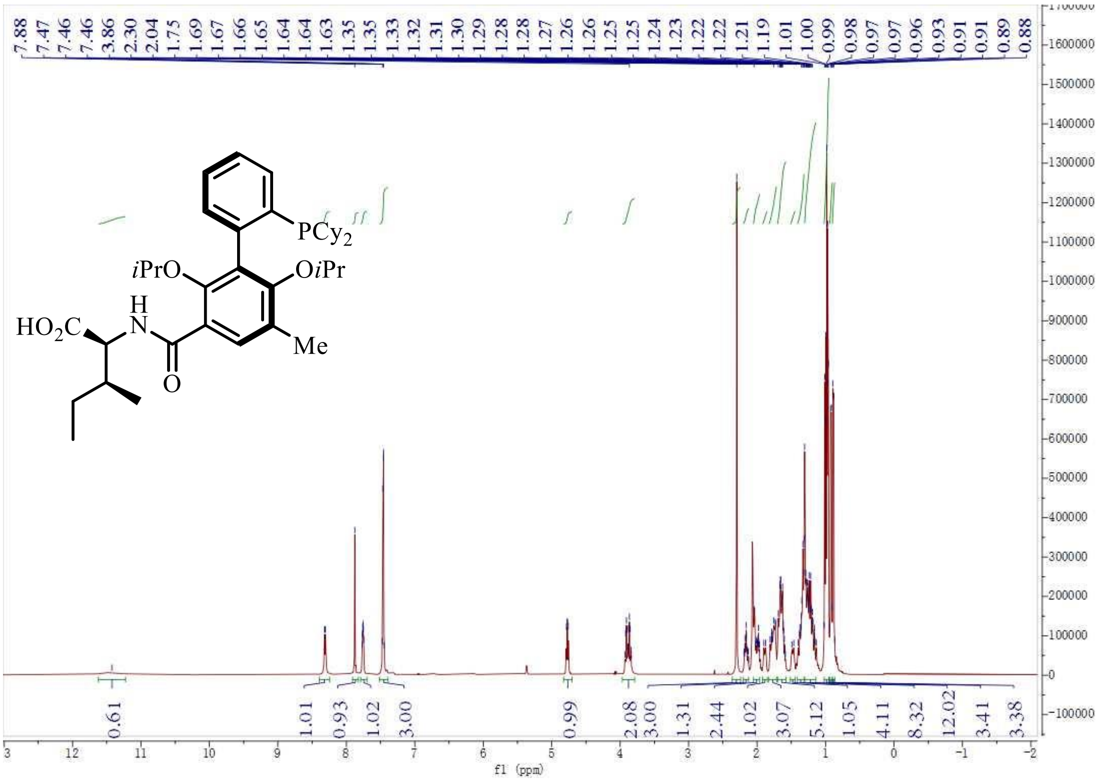

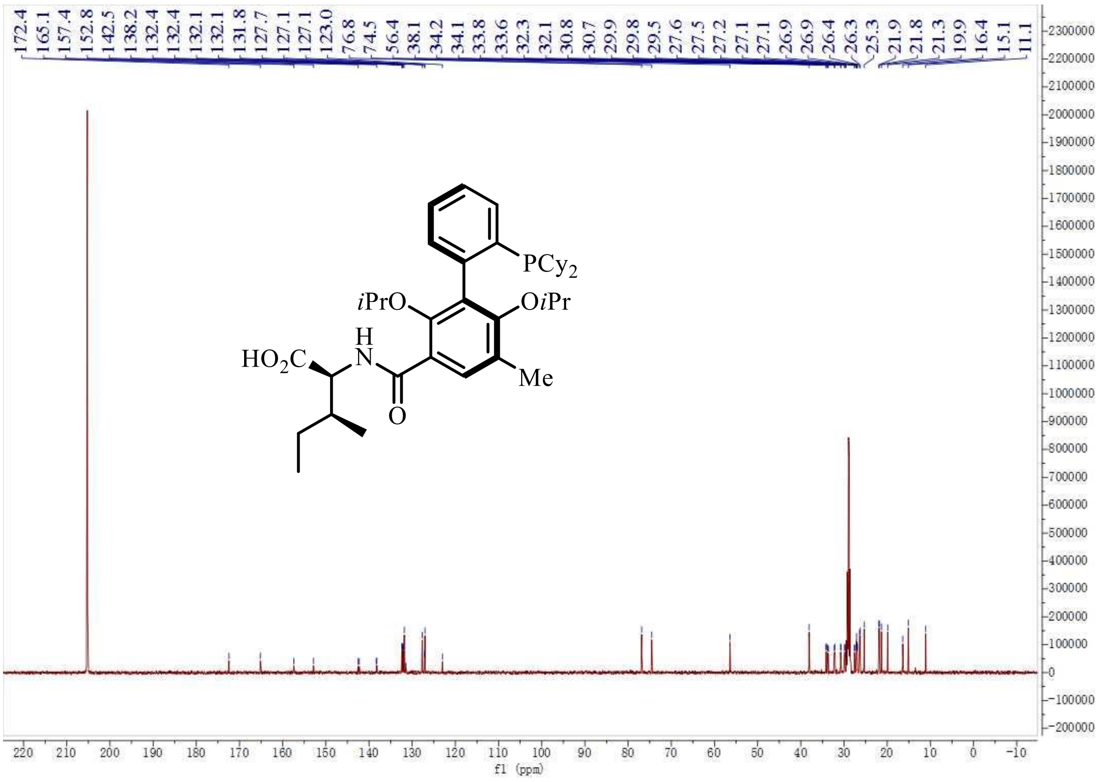

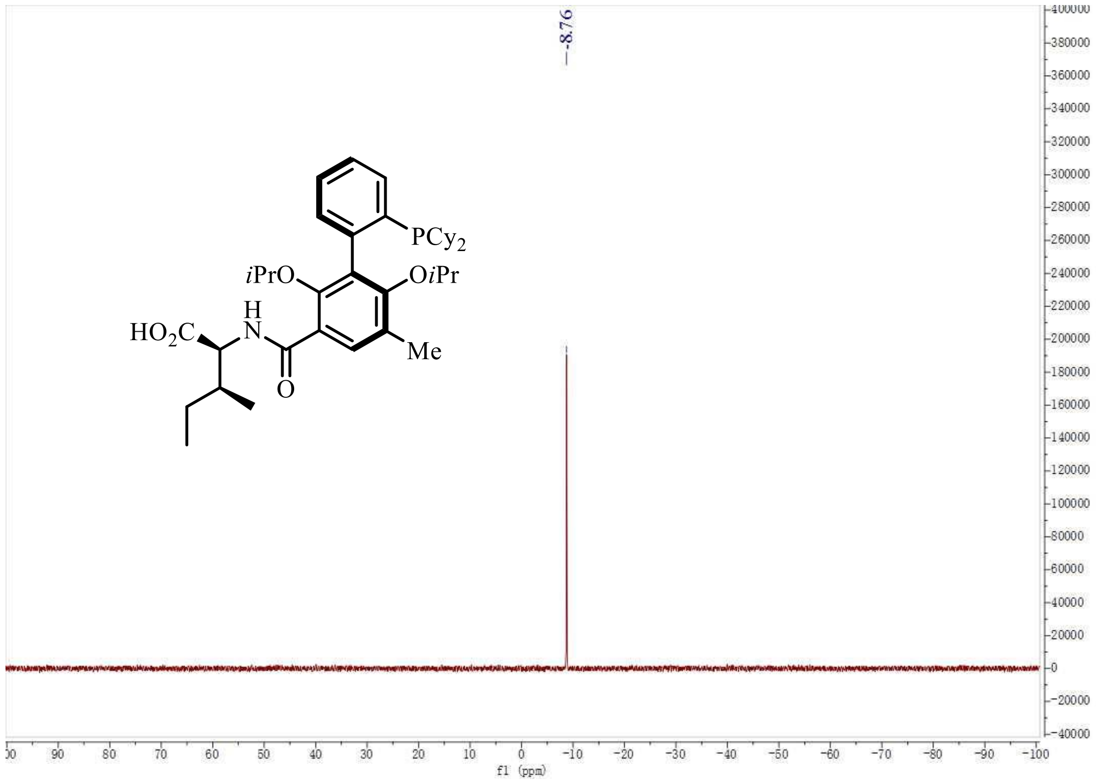

-8.76
HO2C
H
N
O
iPrO
OiPr
PCy2
Me
f1 (ppm)

L11-(*R*)2'-(dicyclohexylphosphaneyl)-2,6-diisopropoxy-5-methyl-[1,1'-biphenyl]-3-carbonyl-L-leucine

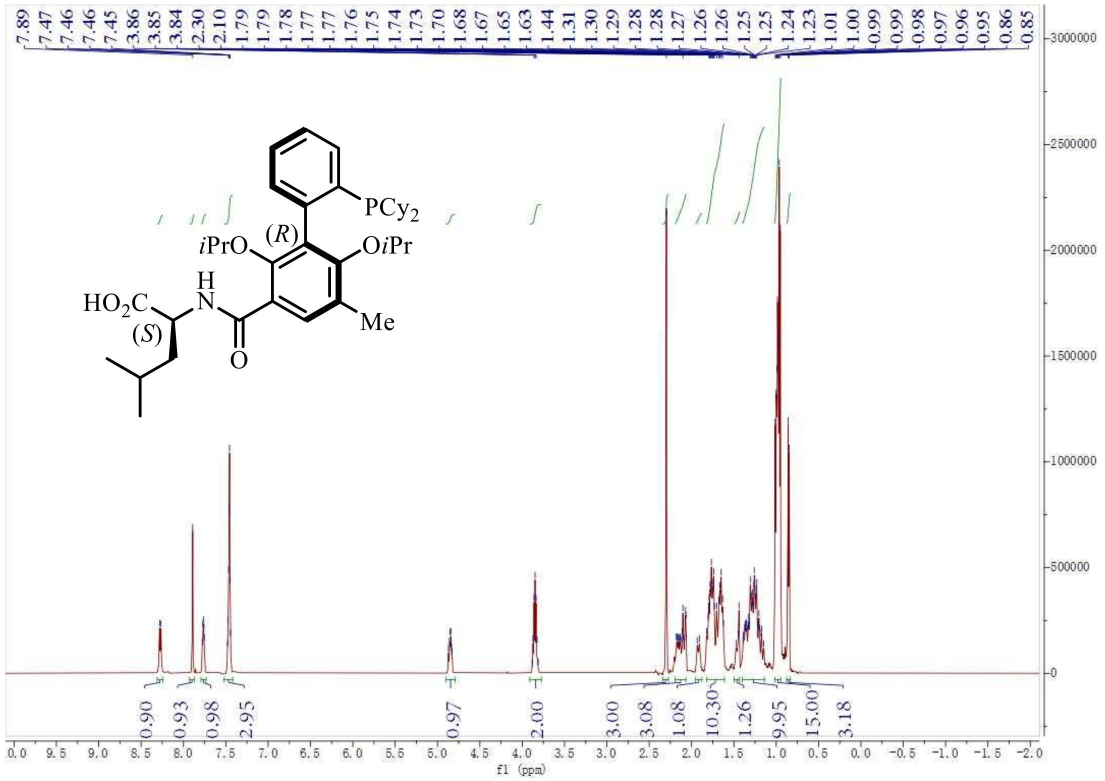

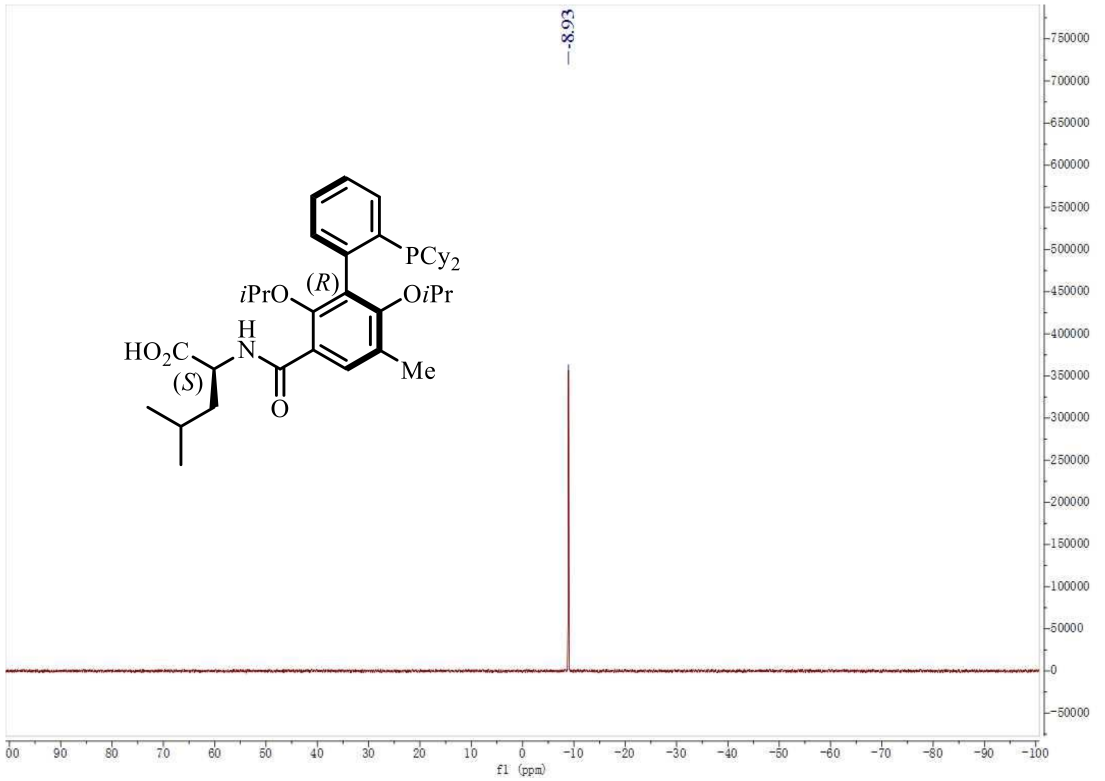

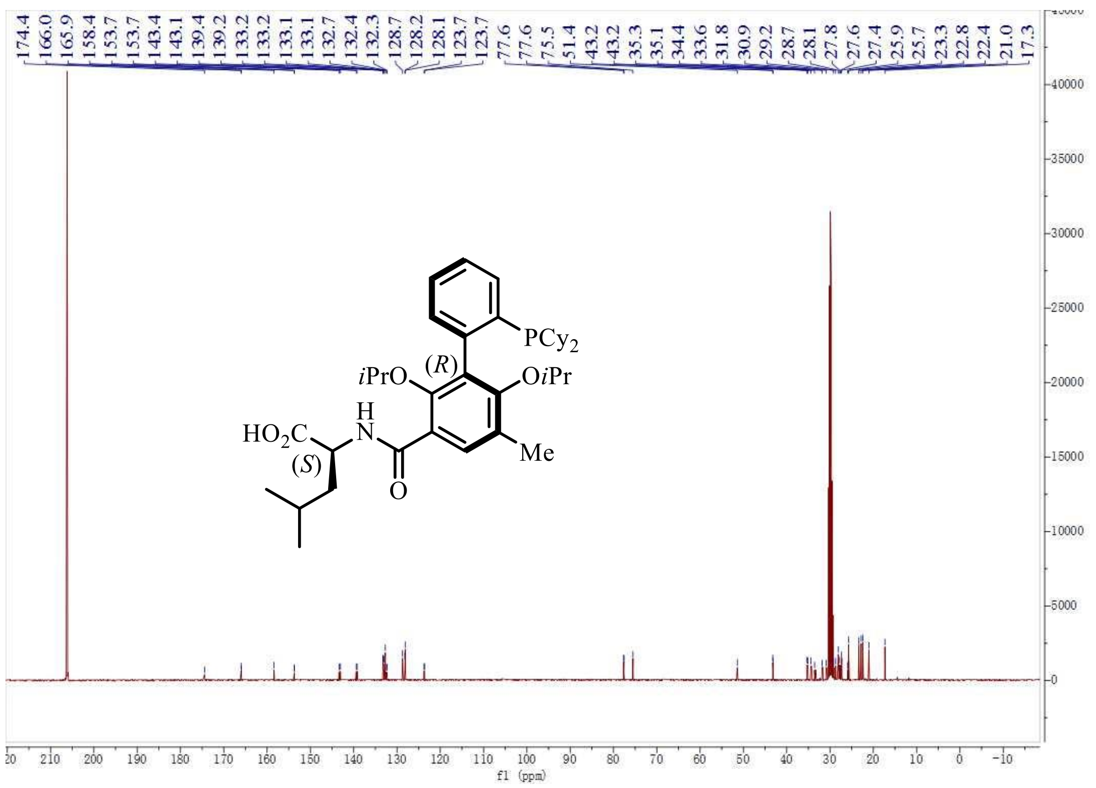

174.4
166.0
165.9
158.4
153.7
153.7
143.4
143.1
139.4
139.2
133.2
133.2
133.1
133.1
132.7
132.4
132.3
128.7
128.2
128.1
123.7
123.7
77.6
77.6
75.5
51.4
43.2
43.2
35.3
35.1
34.4
33.6
31.8
30.9
29.2
28.7
28.1
27.8
27.6
27.4
25.9
25.7
23.3
22.8
22.4
21.0
17.3
PCy2
iPrO
(R)
OiPr
H
N
HO2C
(S)
O
Me
f1 (ppm)

L12-(*R*)-2'-(dicyclohexylphosphaneyl)-2,6-diisopropoxy-5-methyl-[1,1'-biphenyl]-3-carbonyl-L-phenylalanine

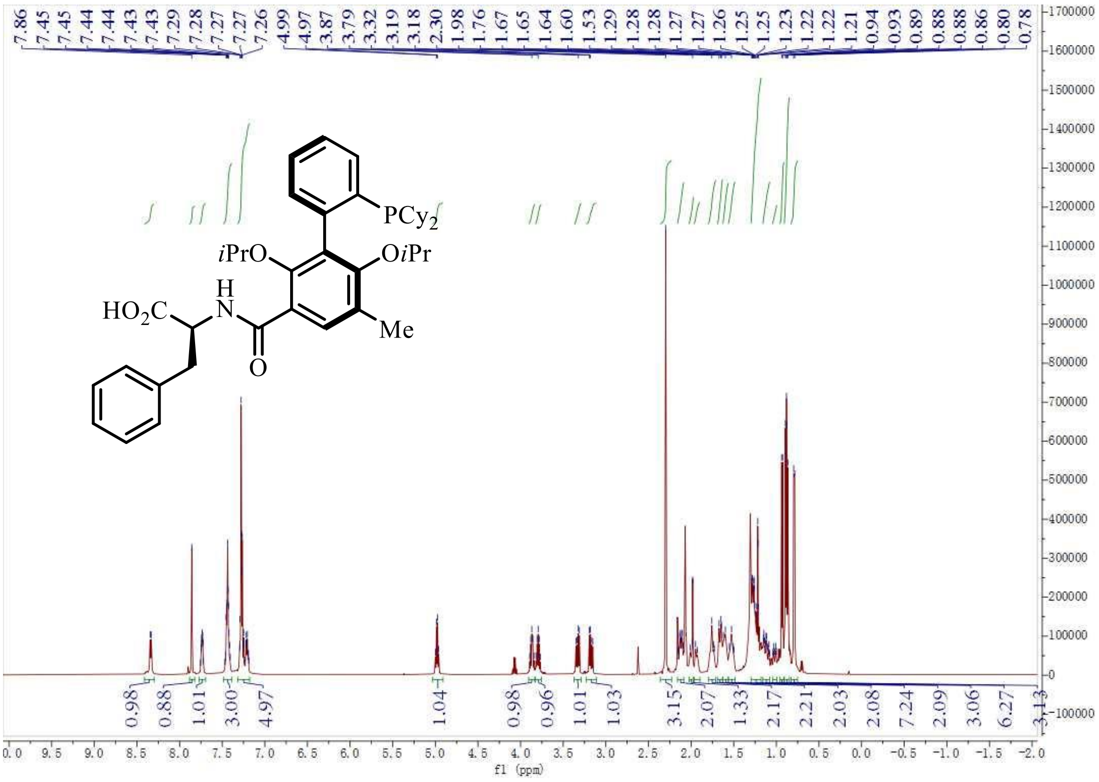

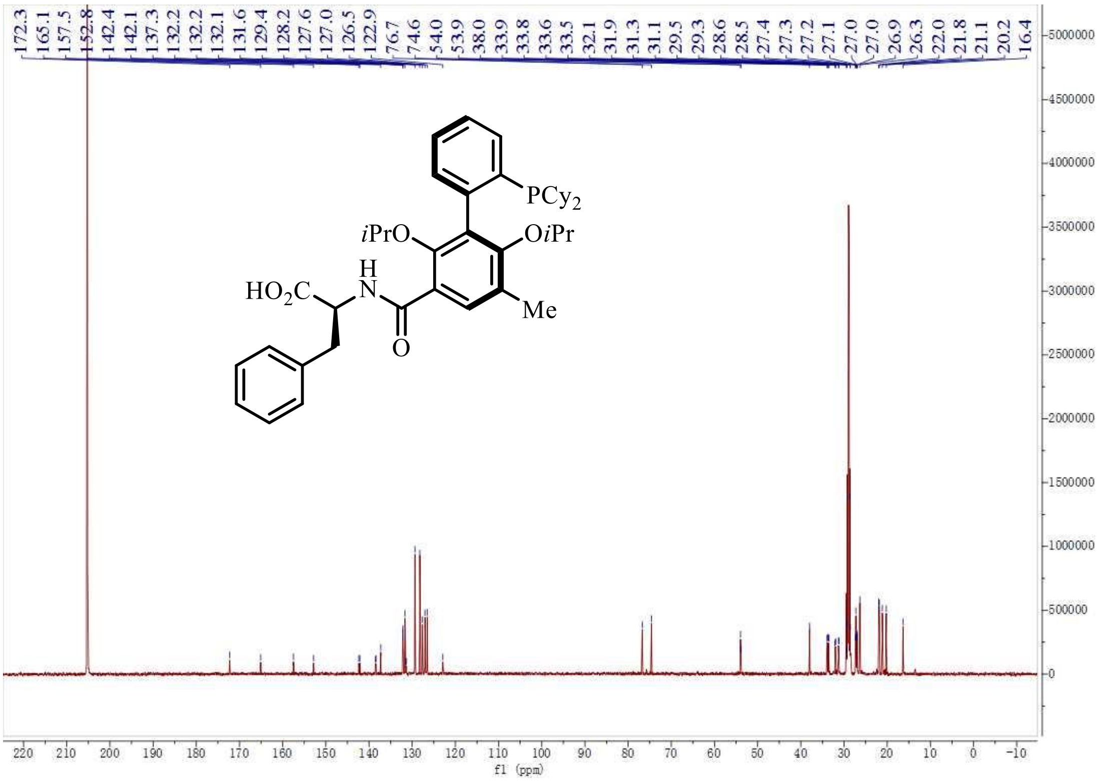

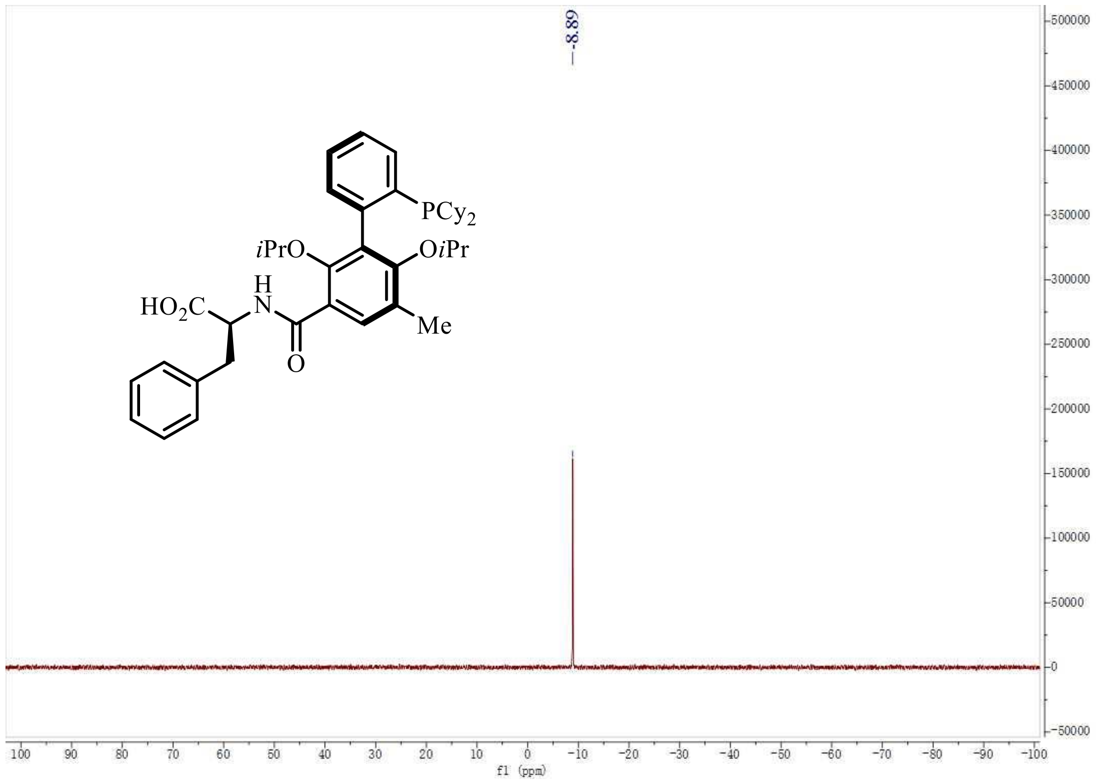

-8.89
PCy2
iPrO
OiPr
HO2C
H
N
O
Me
f1 (ppm)

L13-(*R*)-2'-(dicyclohexylphosphaneyl)-2,6-diisopropoxy-5-methyl-[1,1'-biphenyl]-3-carbonyl)-L-tryptophan

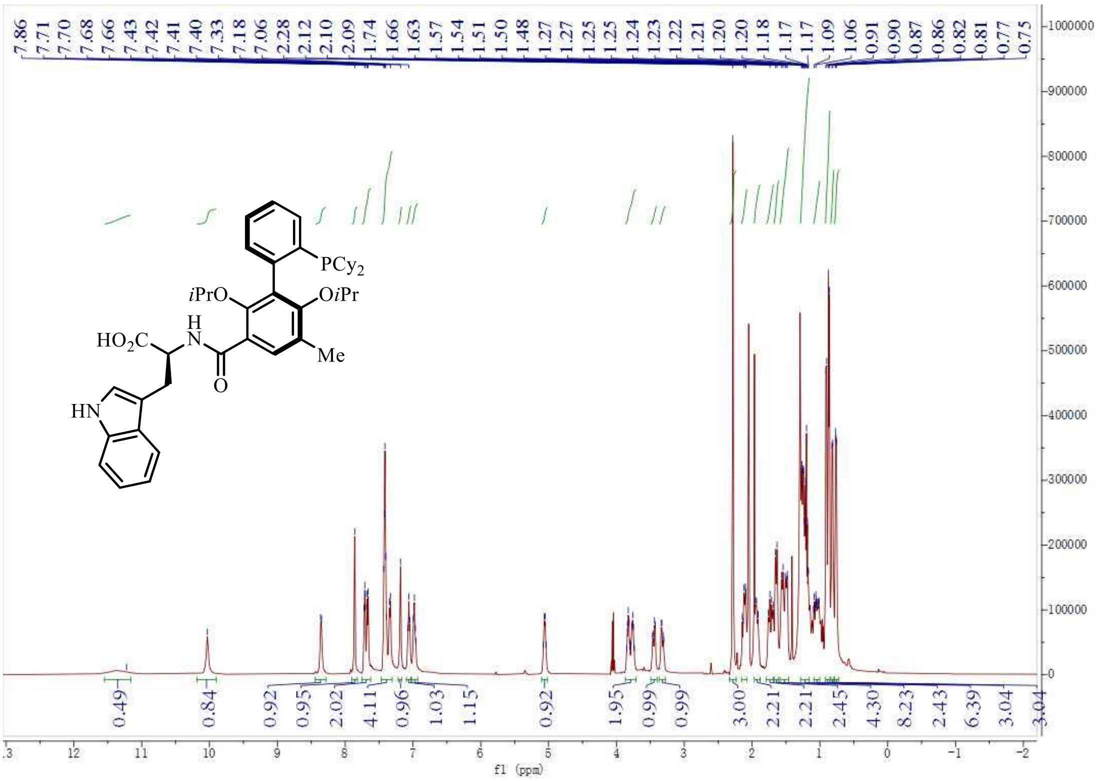

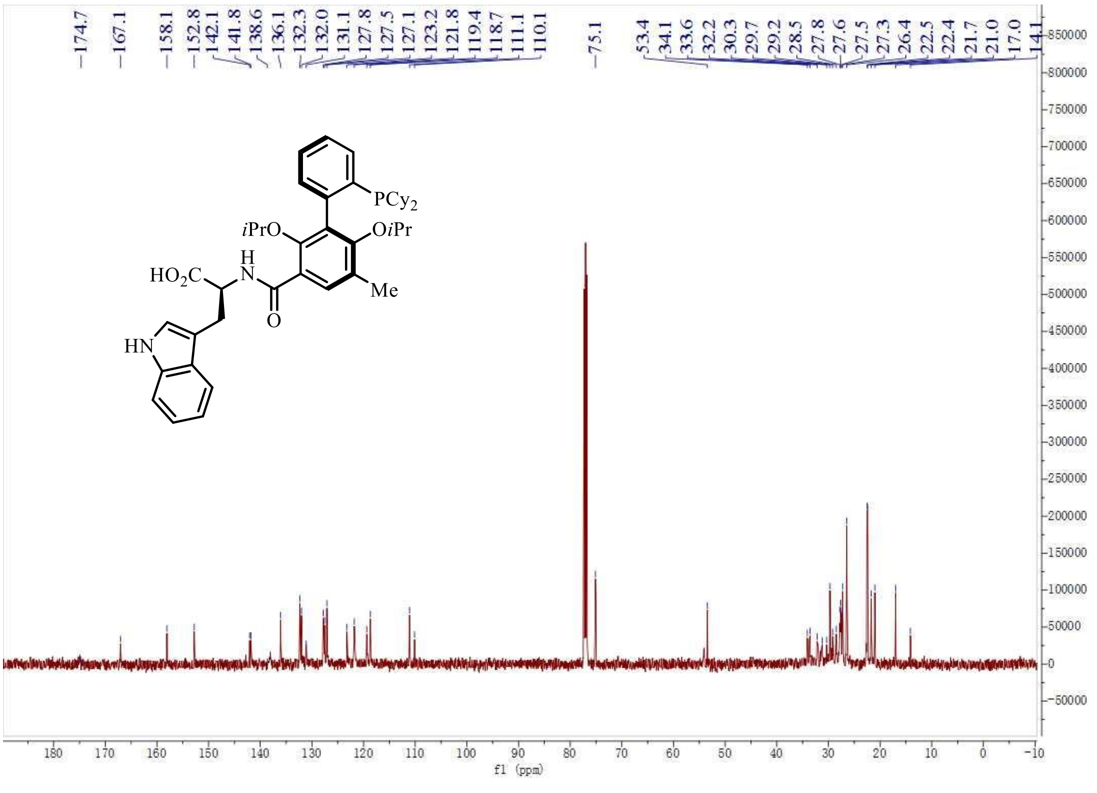

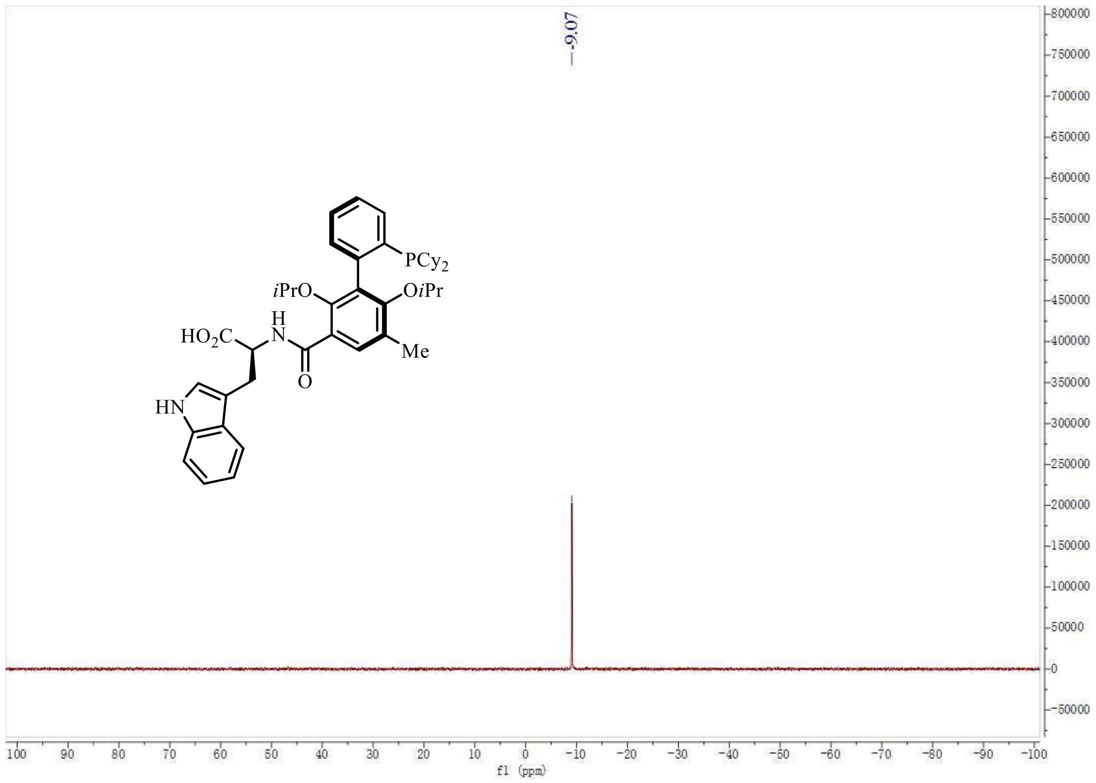
-9.07
PCy2
iPrO
OiPr
H
N
HO2C
Me
O
HN
800000
750000
700000
650000
600000
550000
500000
450000
400000
350000
300000
250000
200000
150000
100000
50000
0
-50000
100 90 80 70 60 50 40 30 20 10 0 -10 -20 -30 -40 -50 -60 -70 -80 -90 -100
f1 (ppm)

L14-(*S*)-2'-(dicyclohexylphosphaneyl)-2,6-diisopropoxy-5-methyl-[1,1'-biphenyl]-3-carbonyl-L-leucine

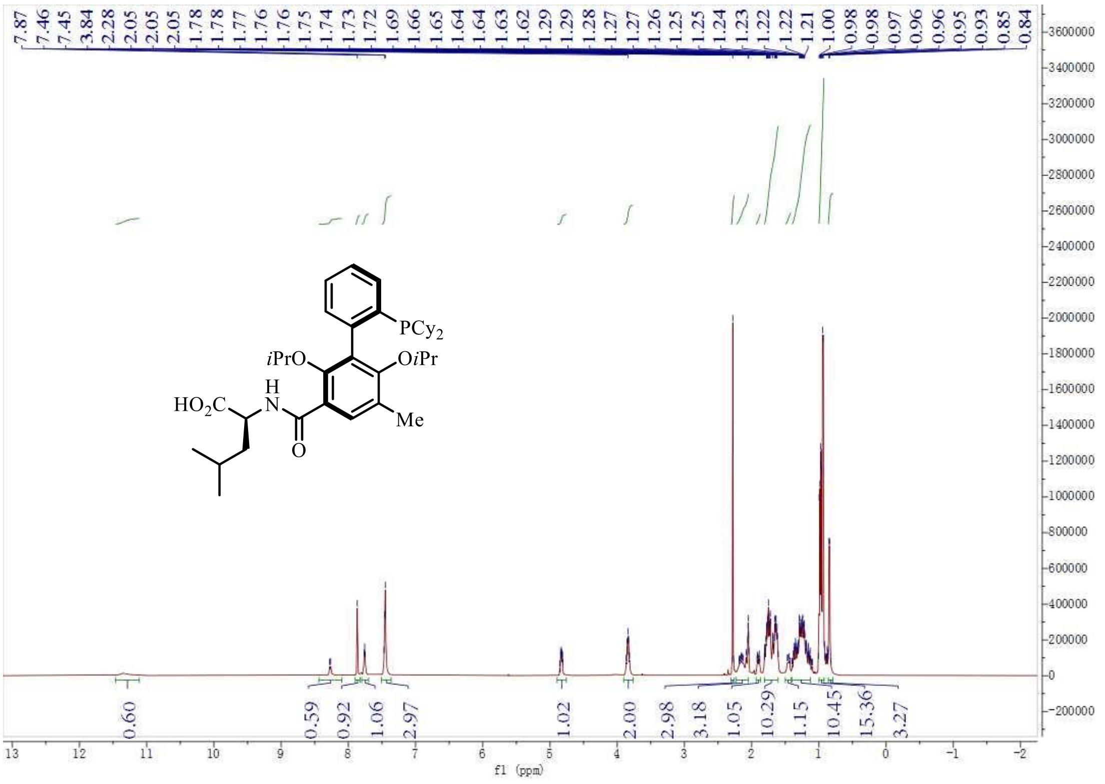

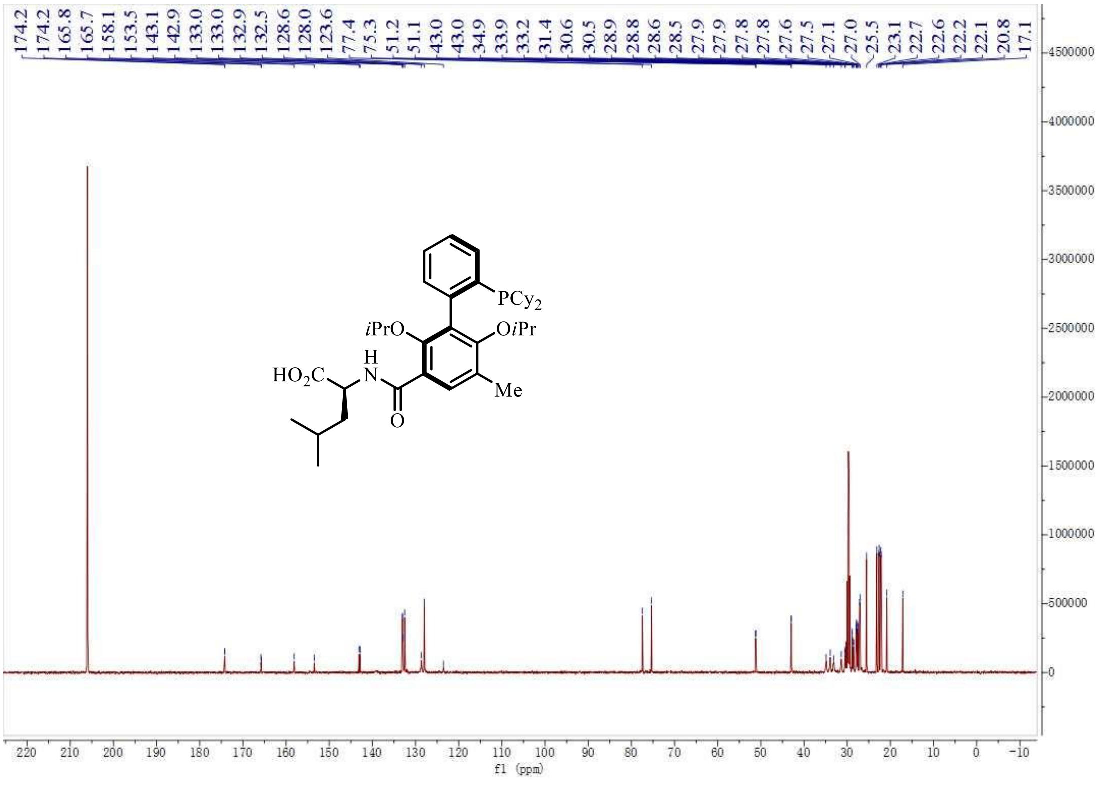

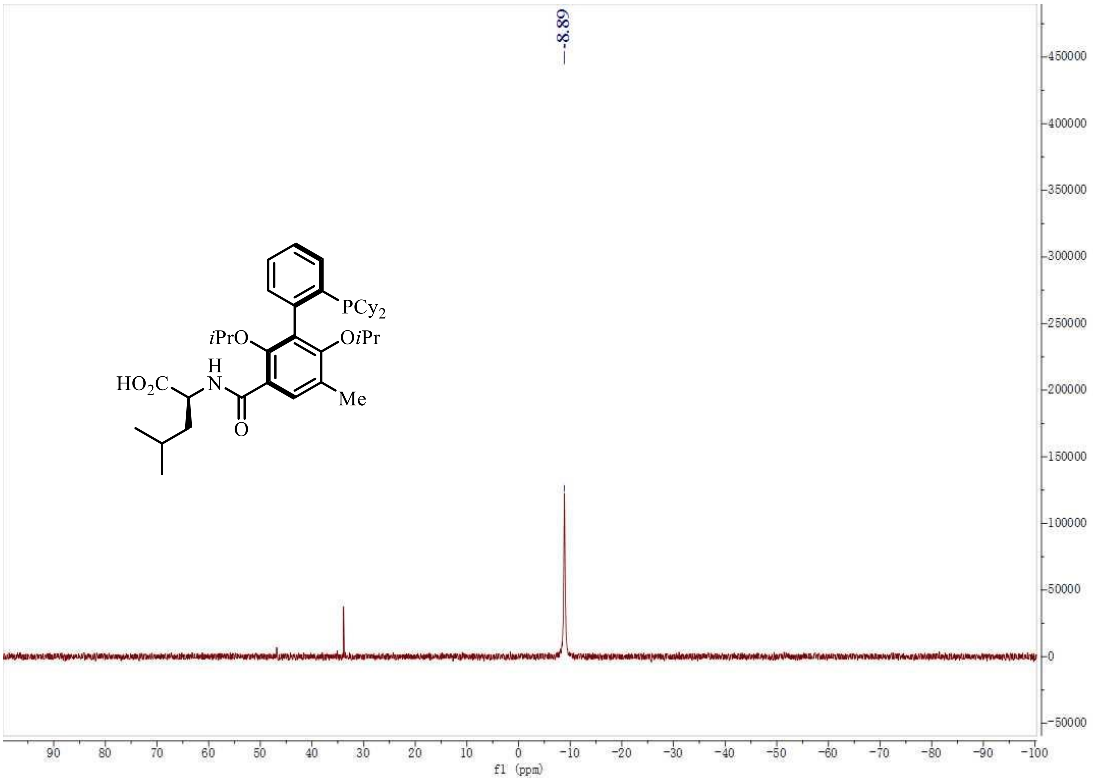

-8.89
PCy2
iPrO
OiPr
H
N
HO2C
Me
O
450000
400000
350000
300000
250000
200000
150000
100000
50000
0
-50000
90
80
70
60
50
40
30
20
10
0
-10
-20
-30
-40
-50
-60
-70
-80
-90
-100
f1 (ppm)

2,2-bis(3-chloro-4-methoxyphenyl)propanoic acid

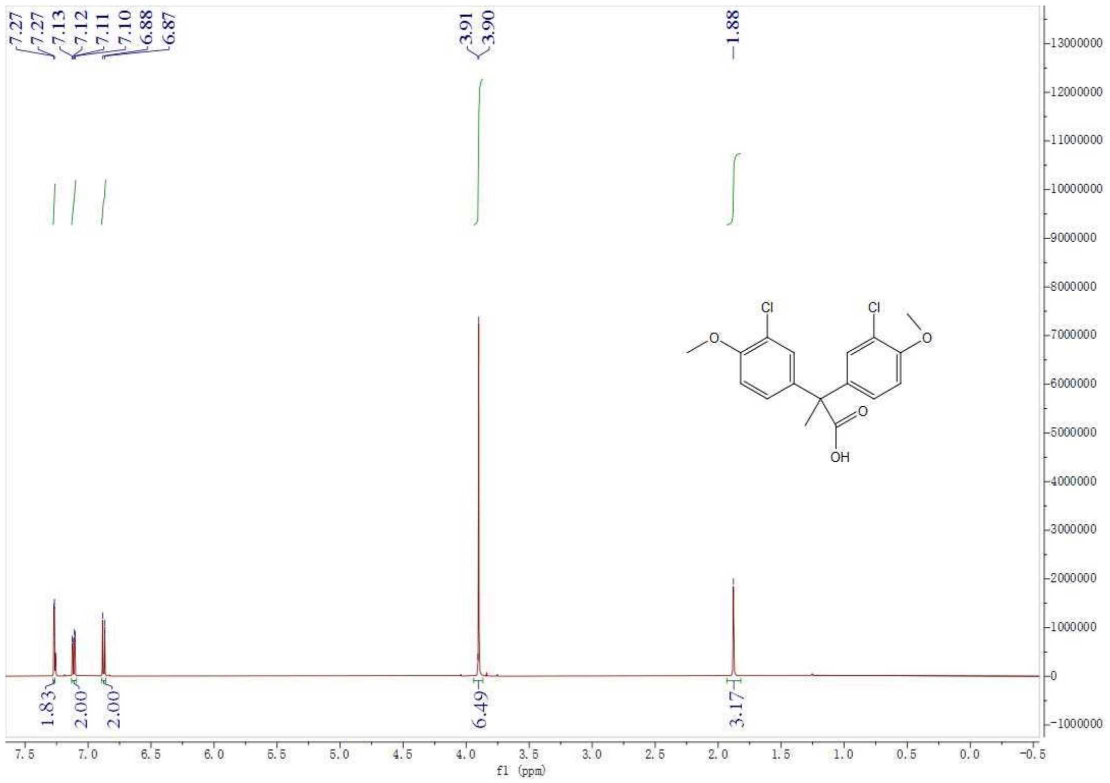

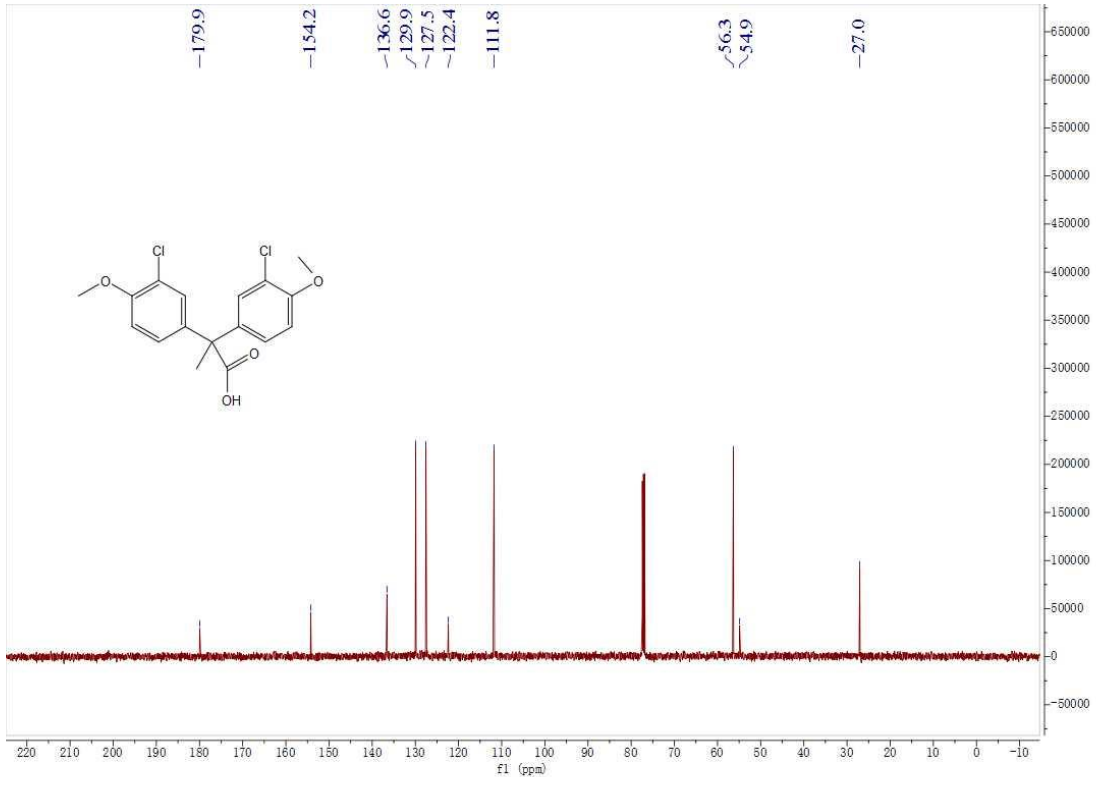

2,2-bis(3-chloro-4-methoxy-5-methylphenyl)propanoic acid

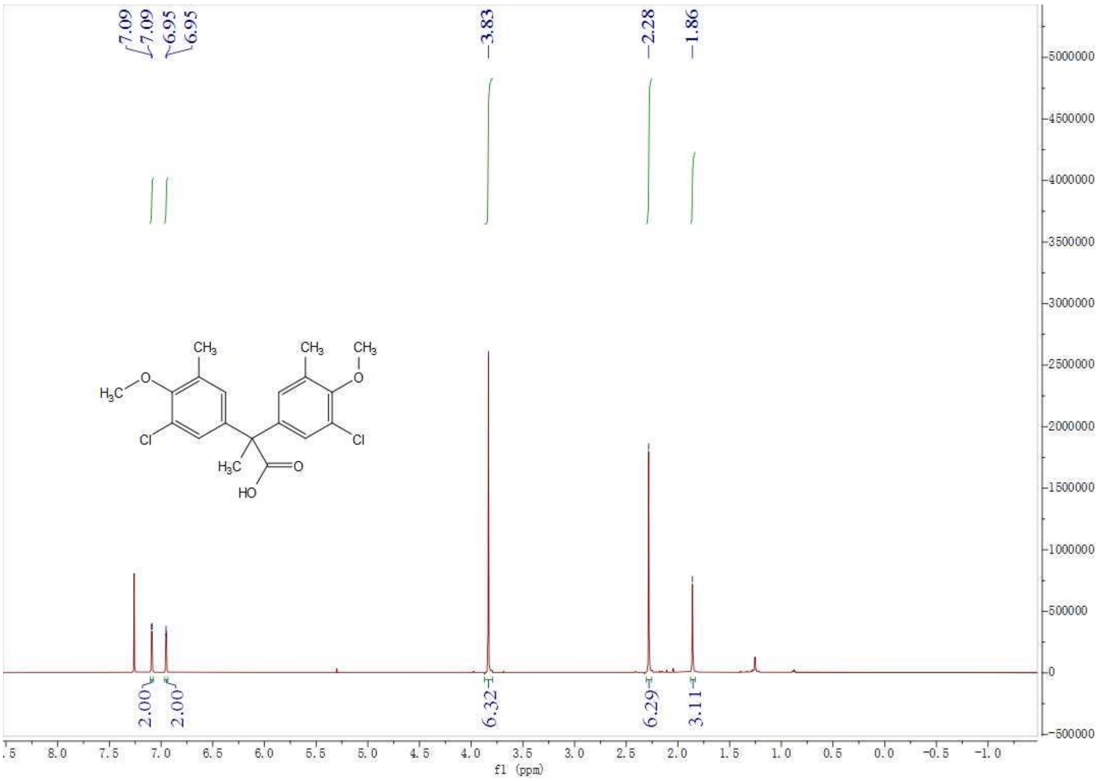

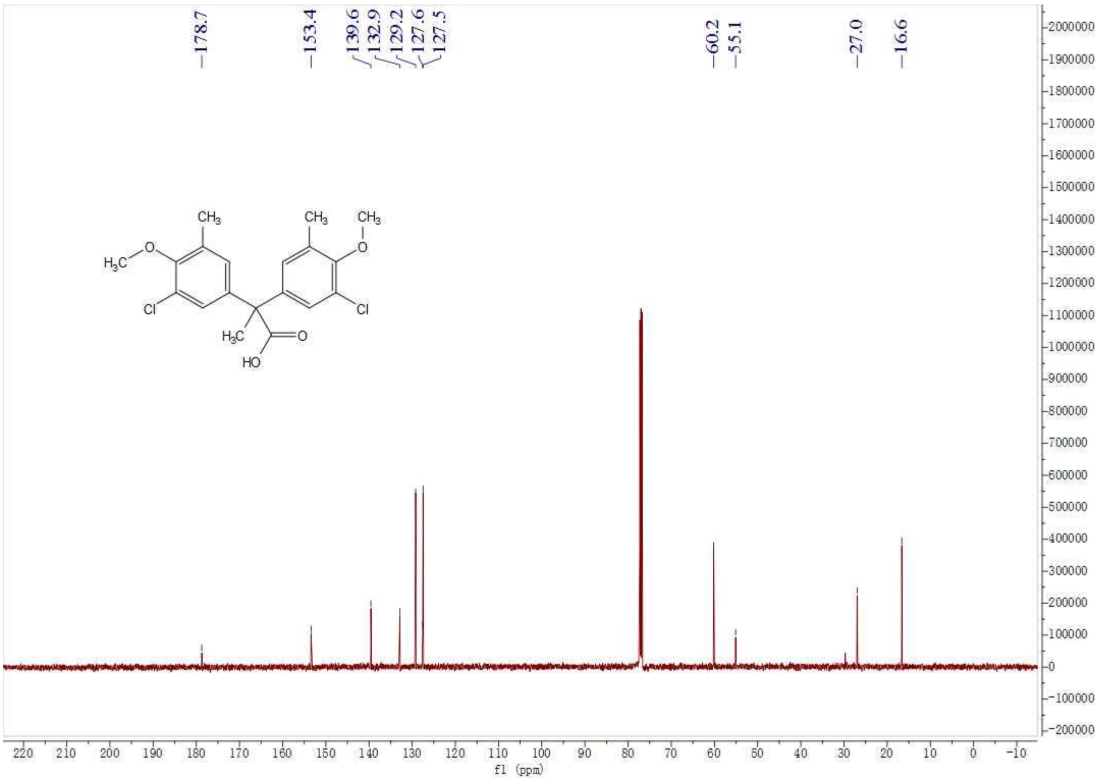

2,2-bis(3-chlorophenyl)propanoic acid

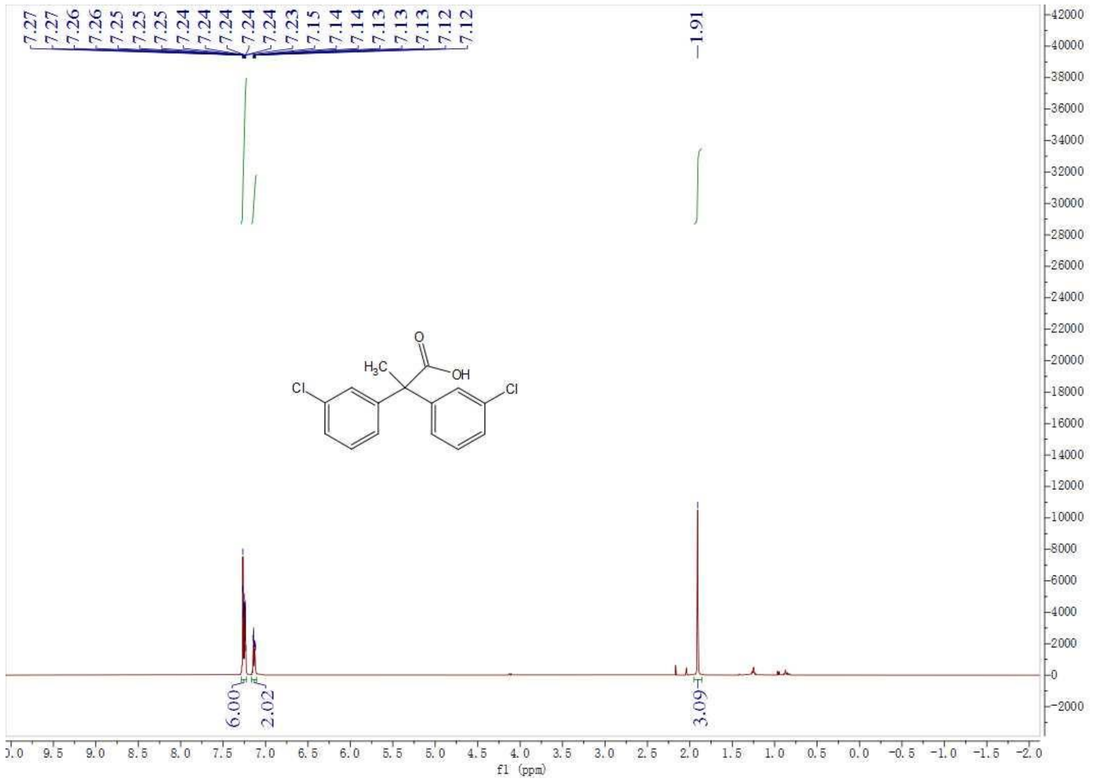

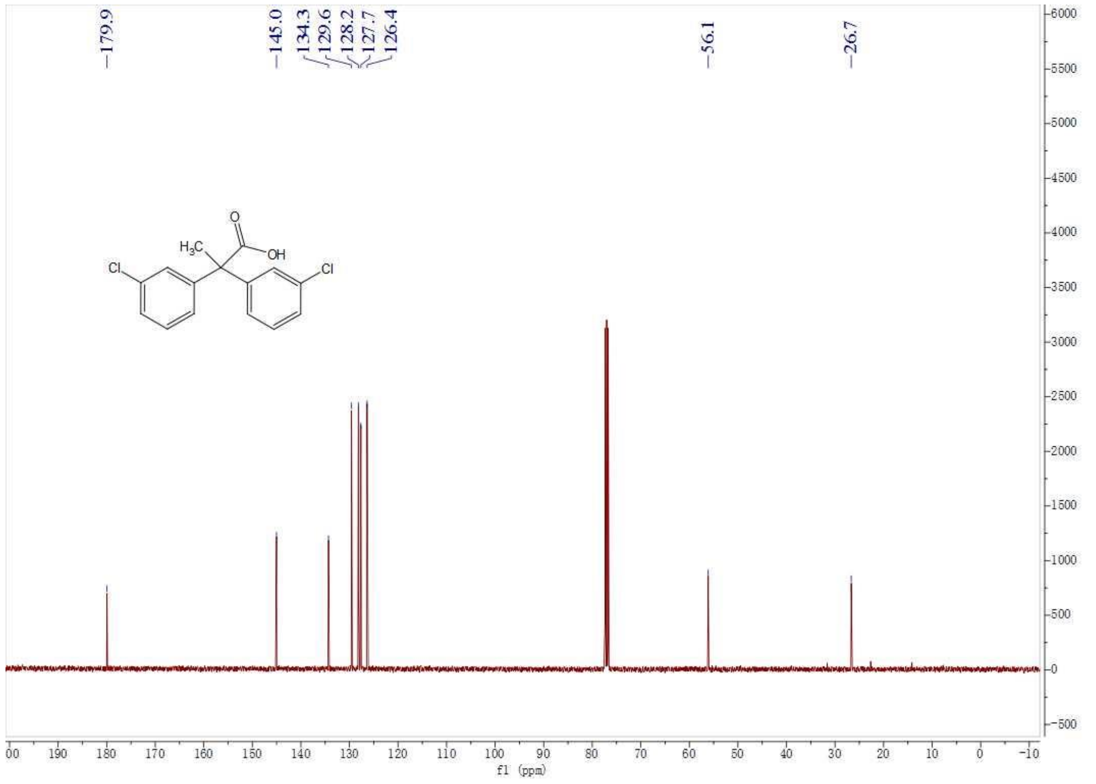

2,2-bis(5-chlorobenzo[b]thiophen-3-yl)propanoic acid

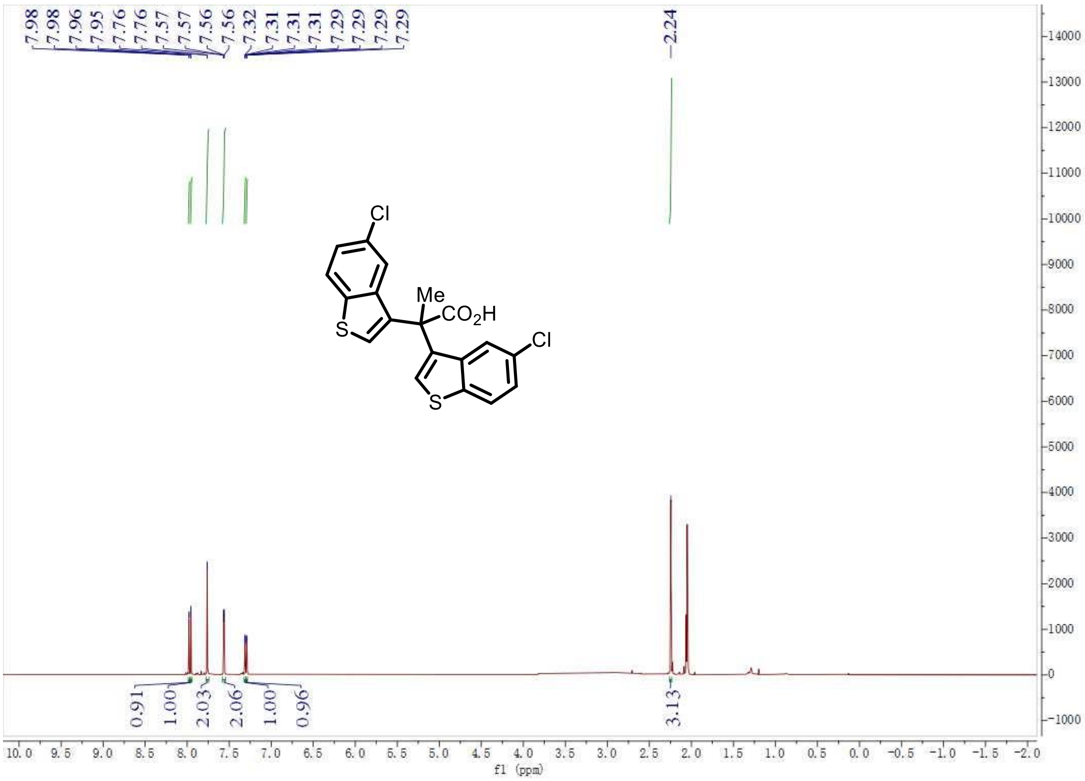

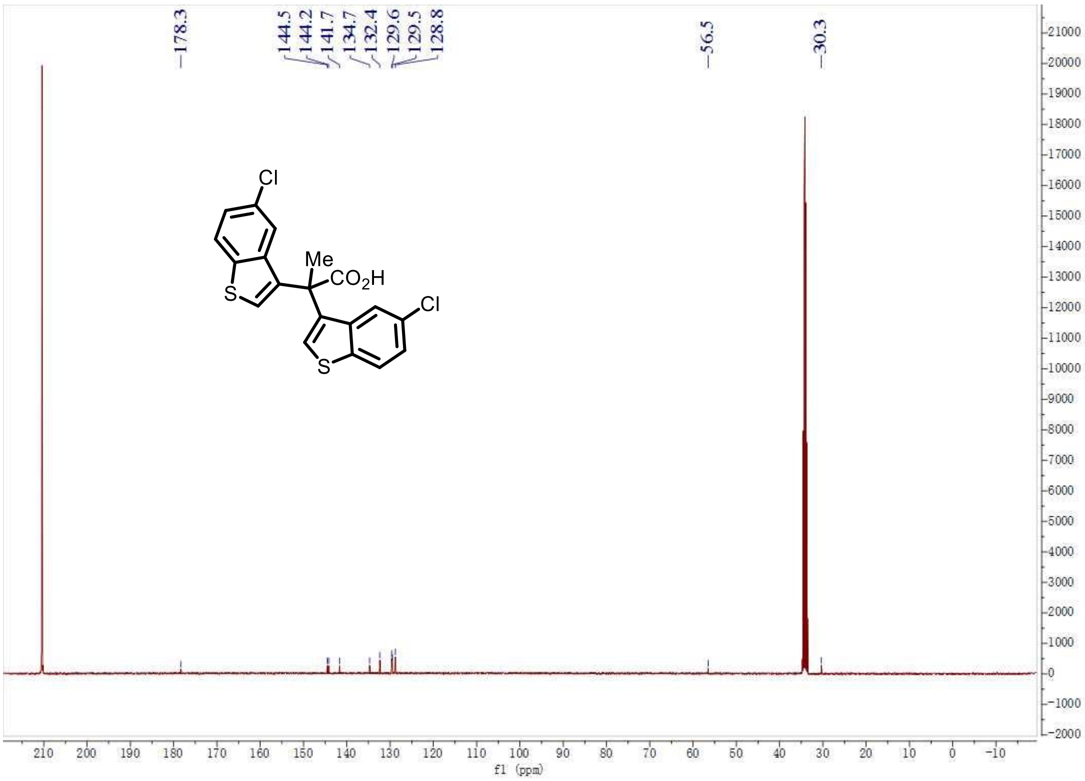

2,2-bis(3-chloro-4-isopropoxyphenyl)propanoic acid

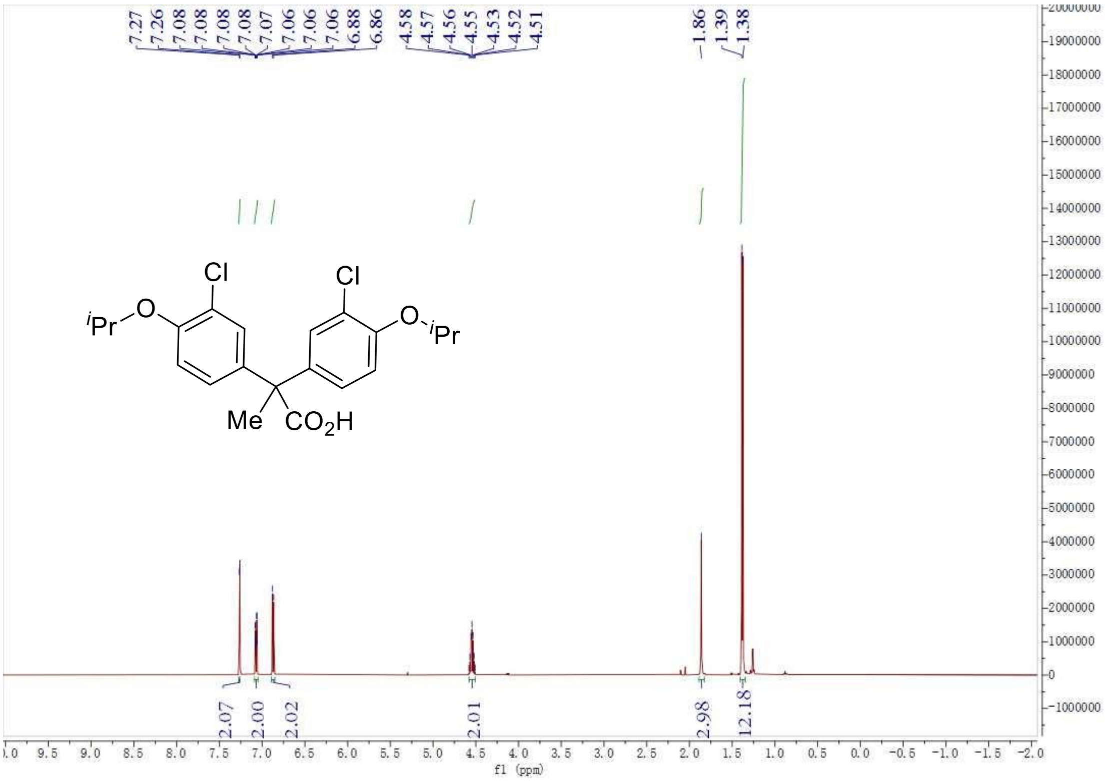

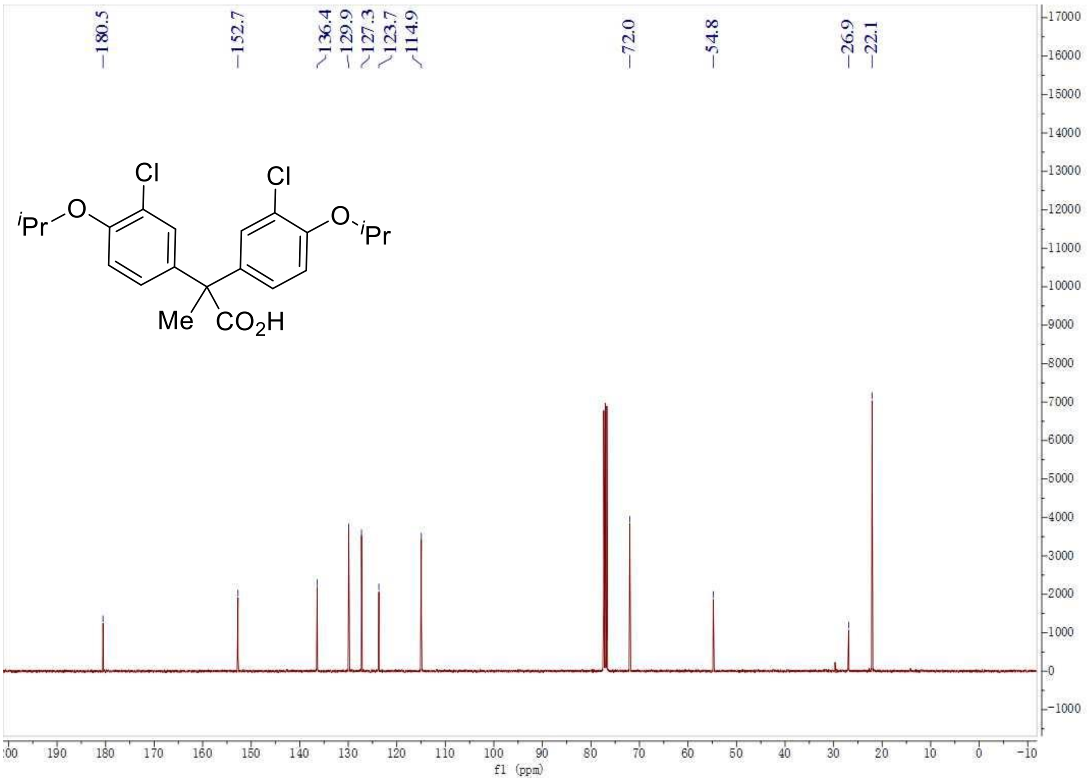

2,2-bis(4-butoxy-3-chlorophenyl)propanoic acid

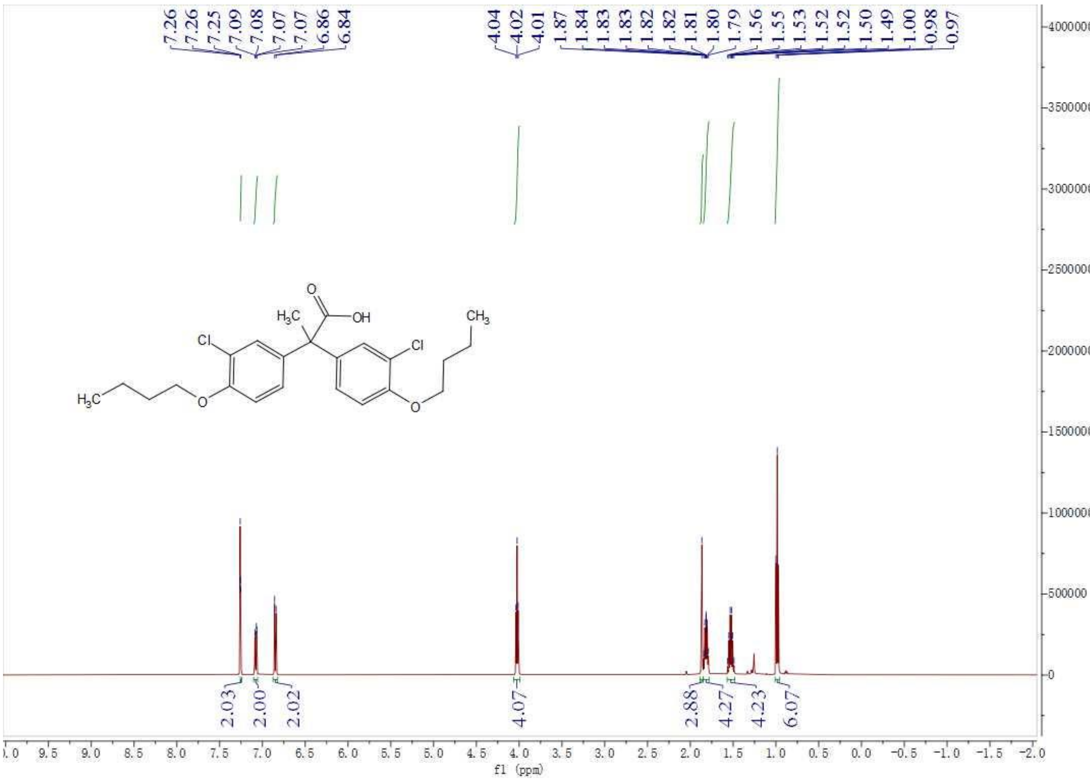

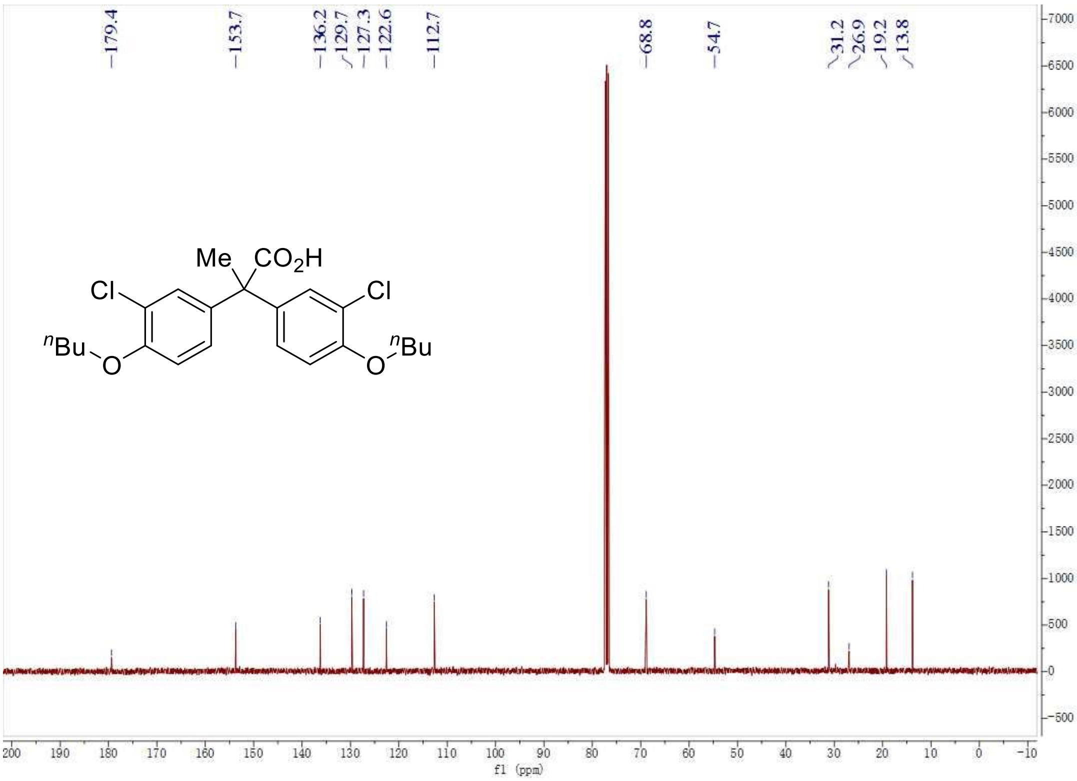

2,2-bis(3-bromo-4-methoxyphenyl)propanoic acid

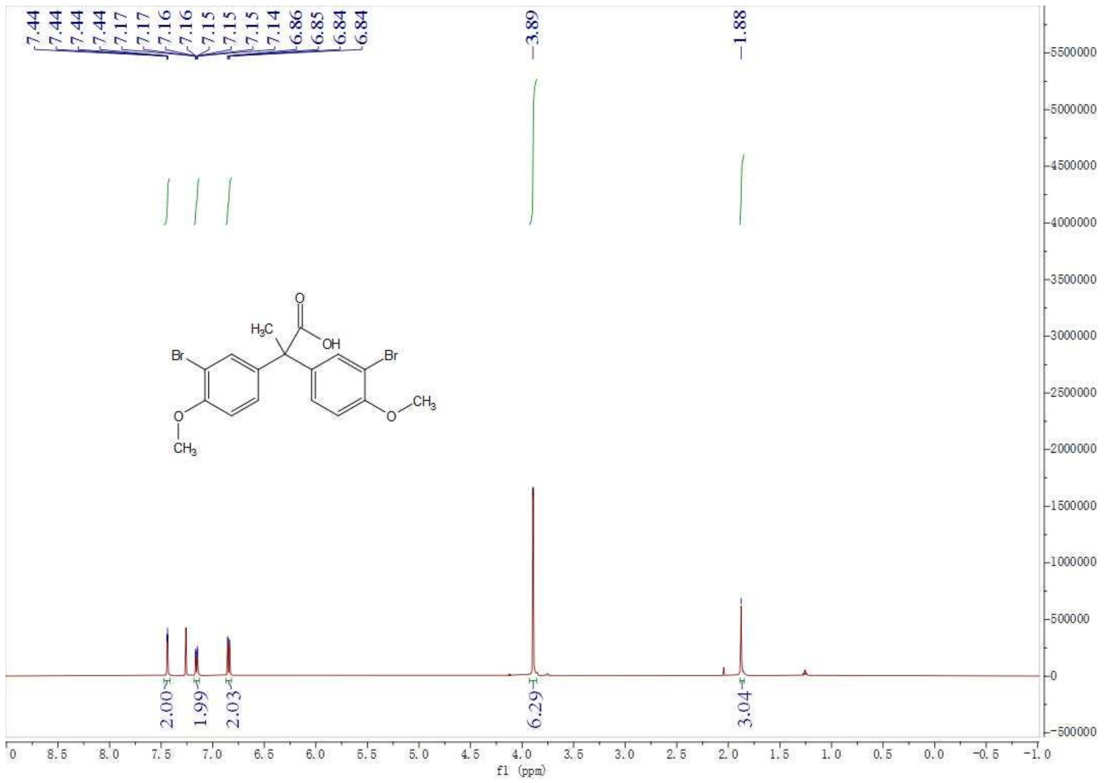

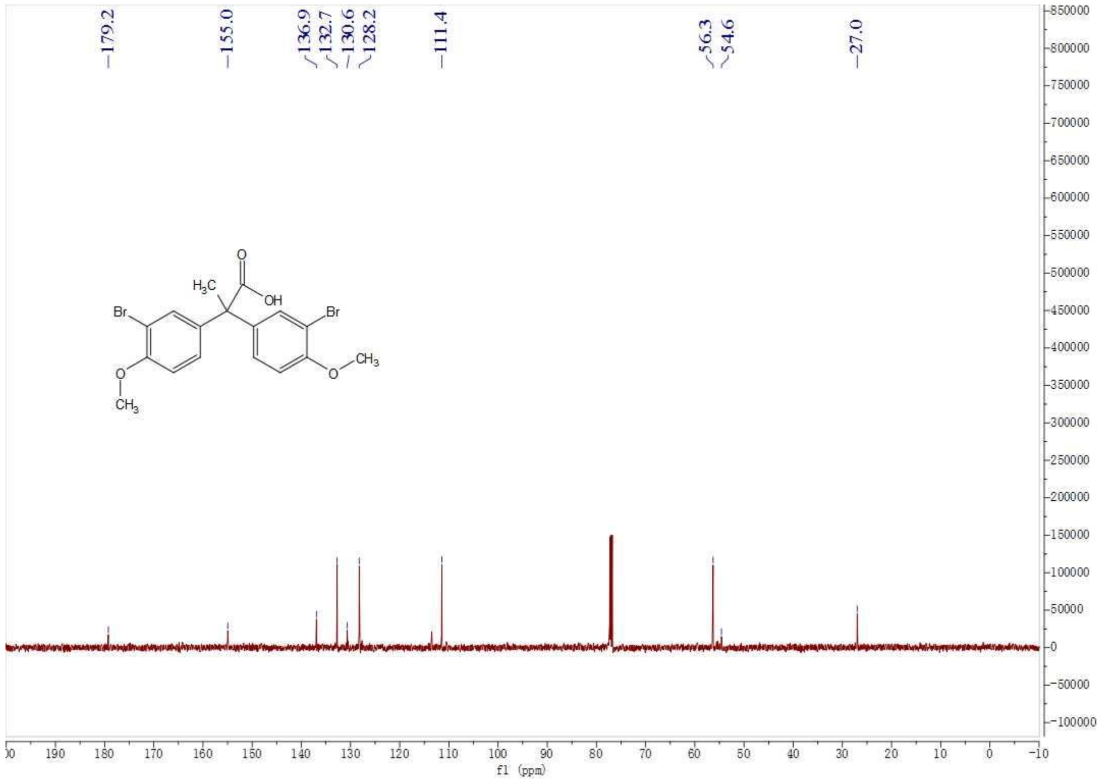

2,2-bis(3-chloro-4-methoxyphenyl)acetic acid

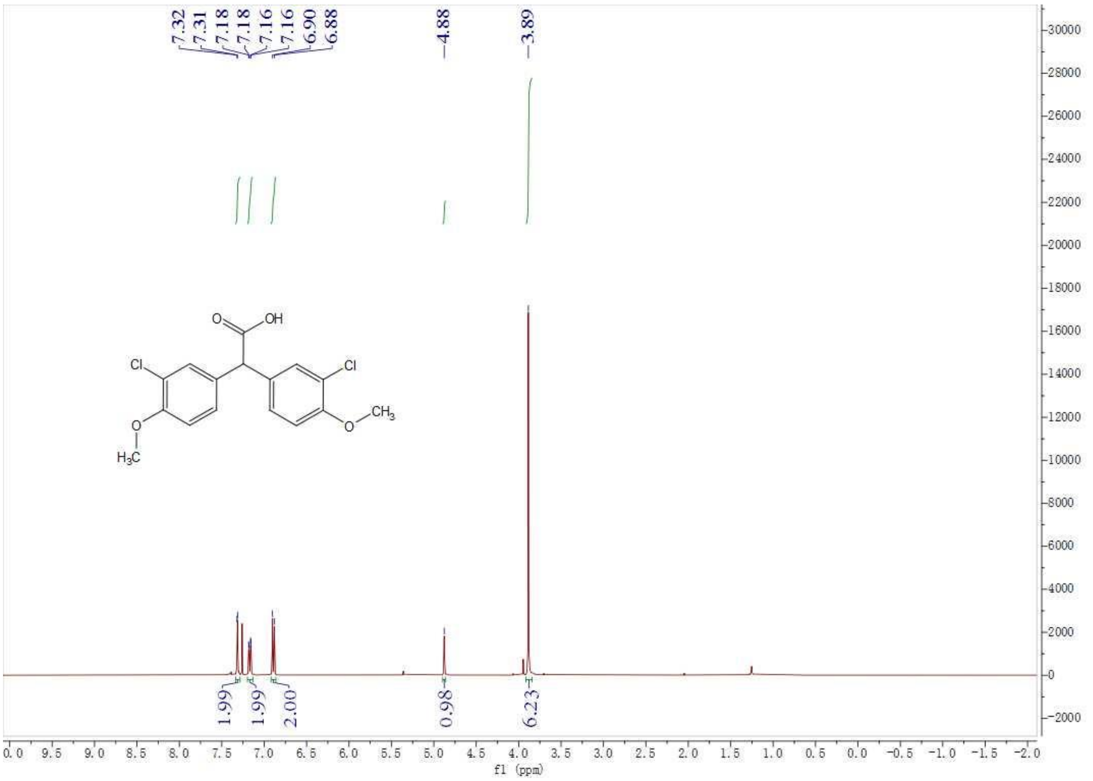

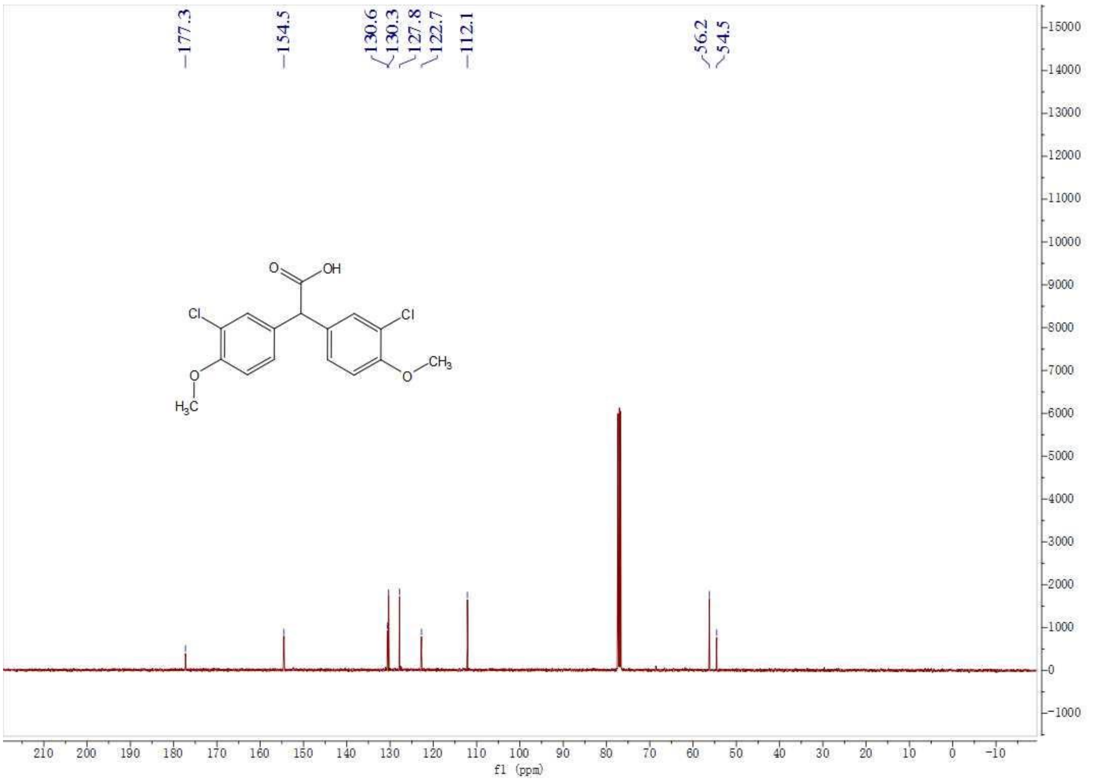

2,2-bis(3-chloro-4-methoxyphenyl)-3-methylbutanoic acid

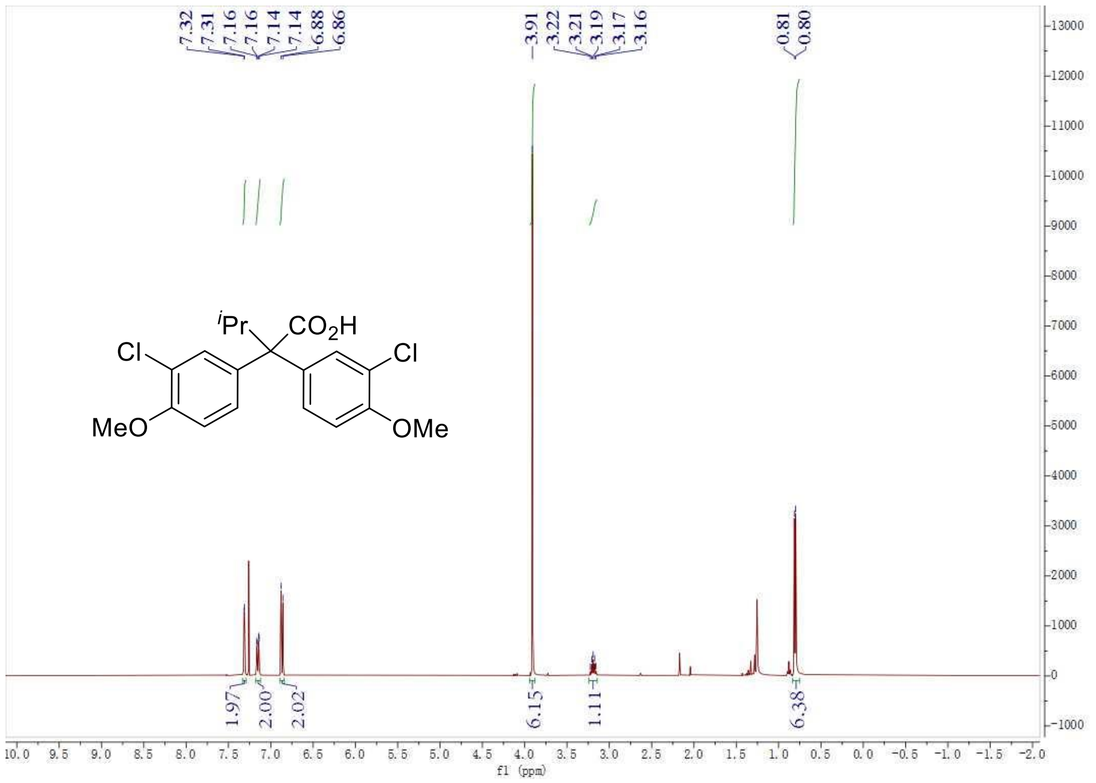

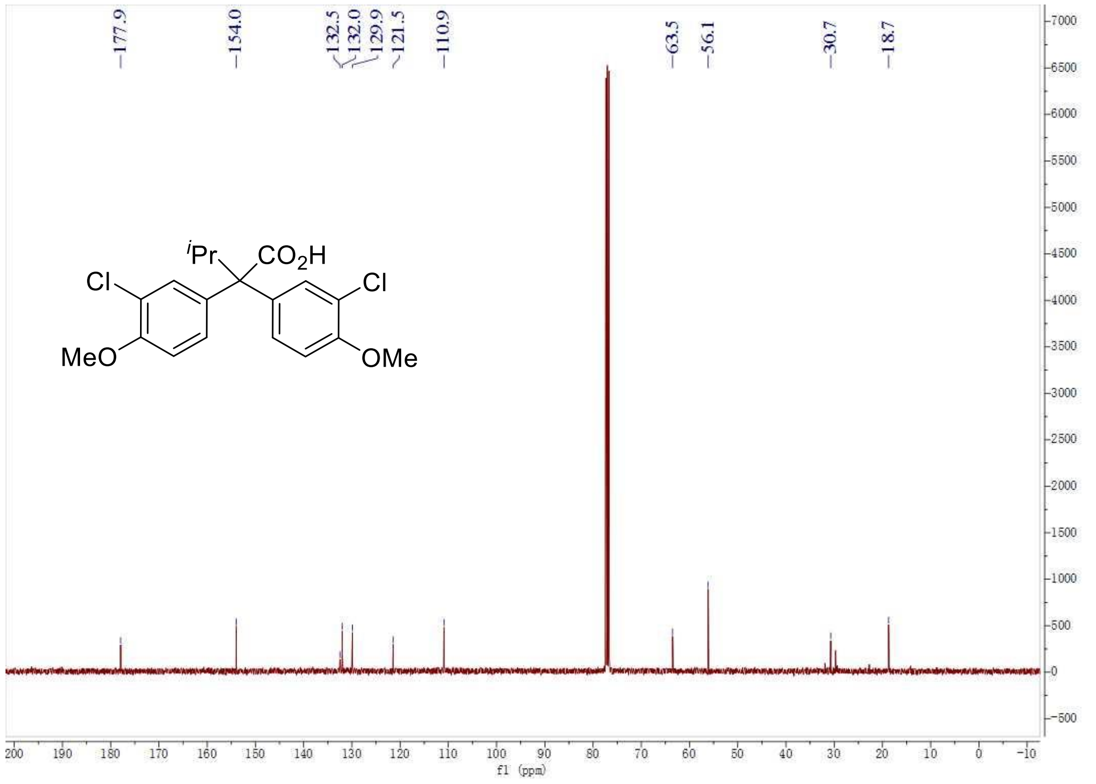

2,2-bis(3-chloro-4-methoxyphenyl)hexanoic acid

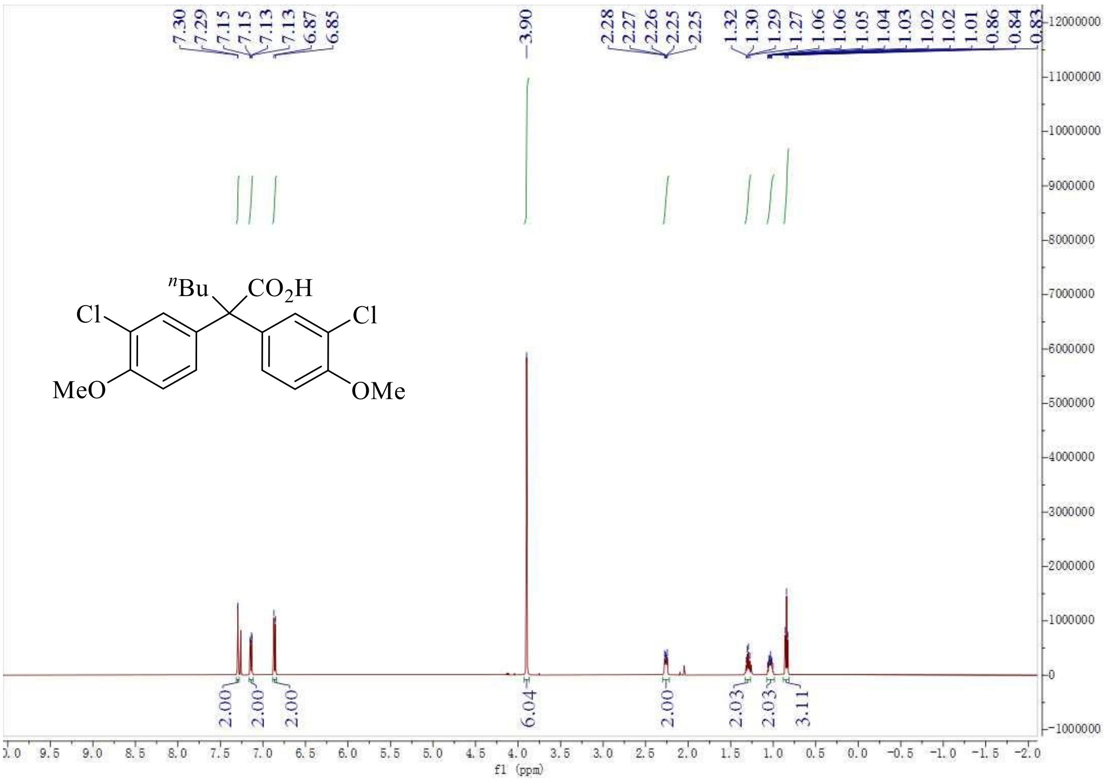

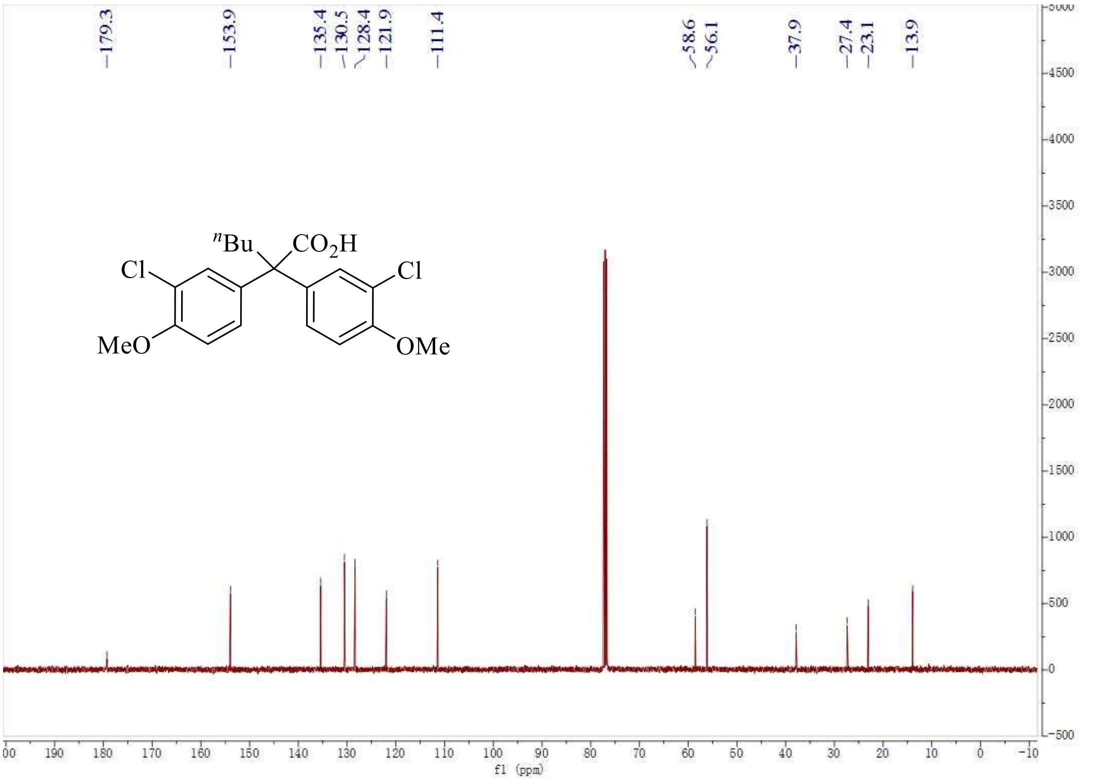

2,2-bis(3-chloro-4-methoxyphenyl)-4-methylpentanoic acid

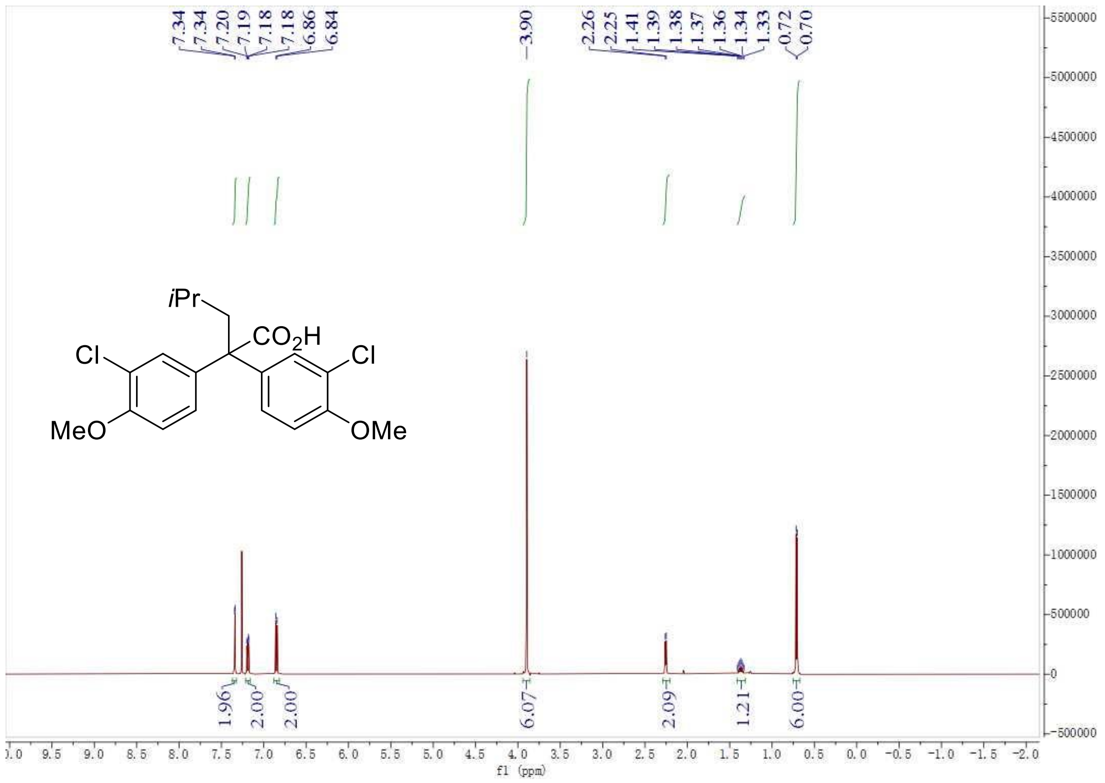

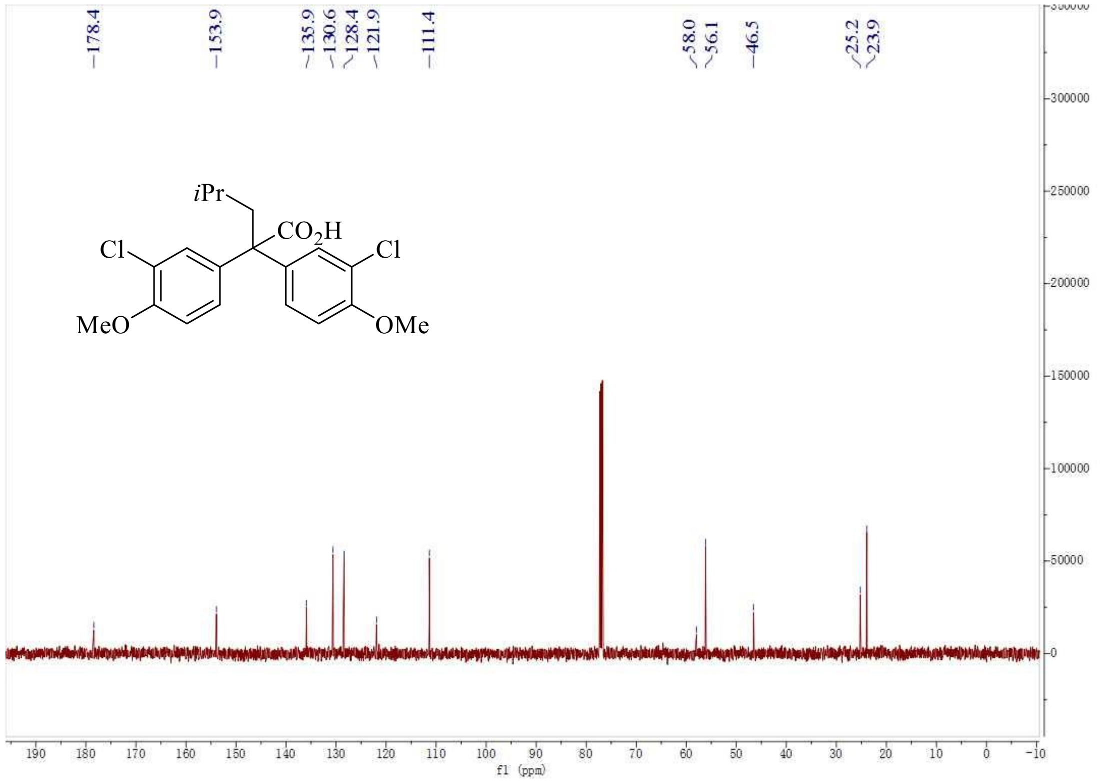

2,2-bis(3-chloro-4-methoxyphenyl)-3-phenylpropanoic acid

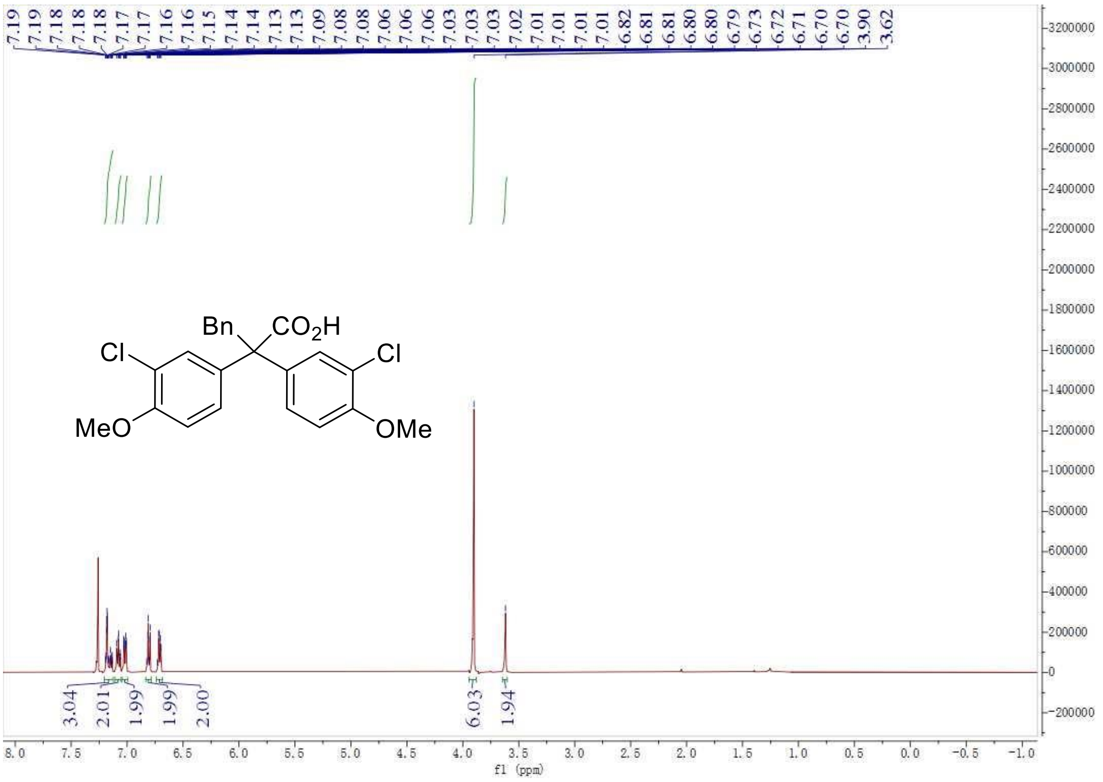

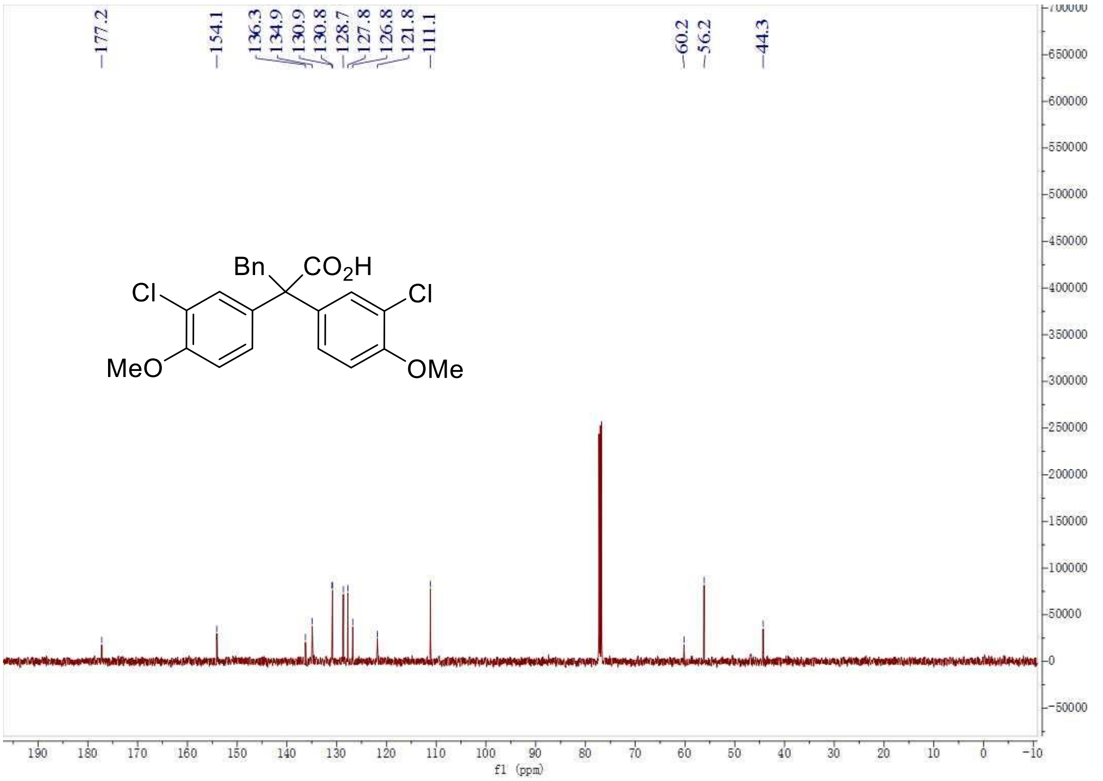

2,2-bis(3-chloro-4-methylphenyl)propanoic acid

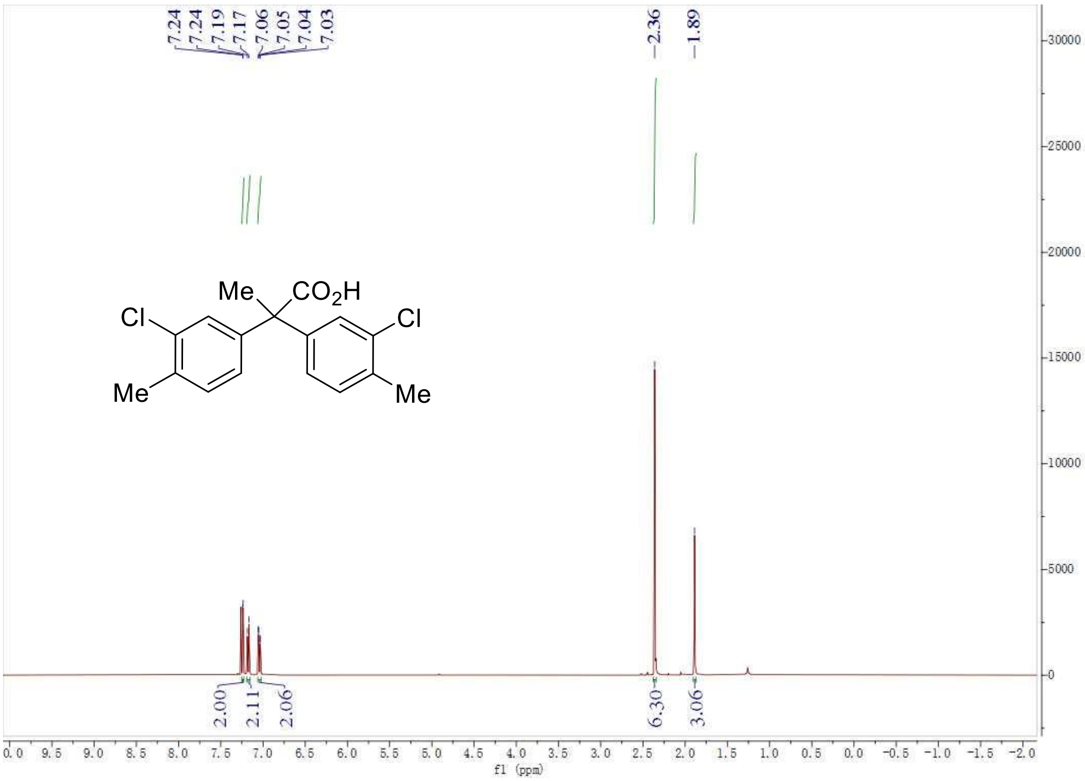

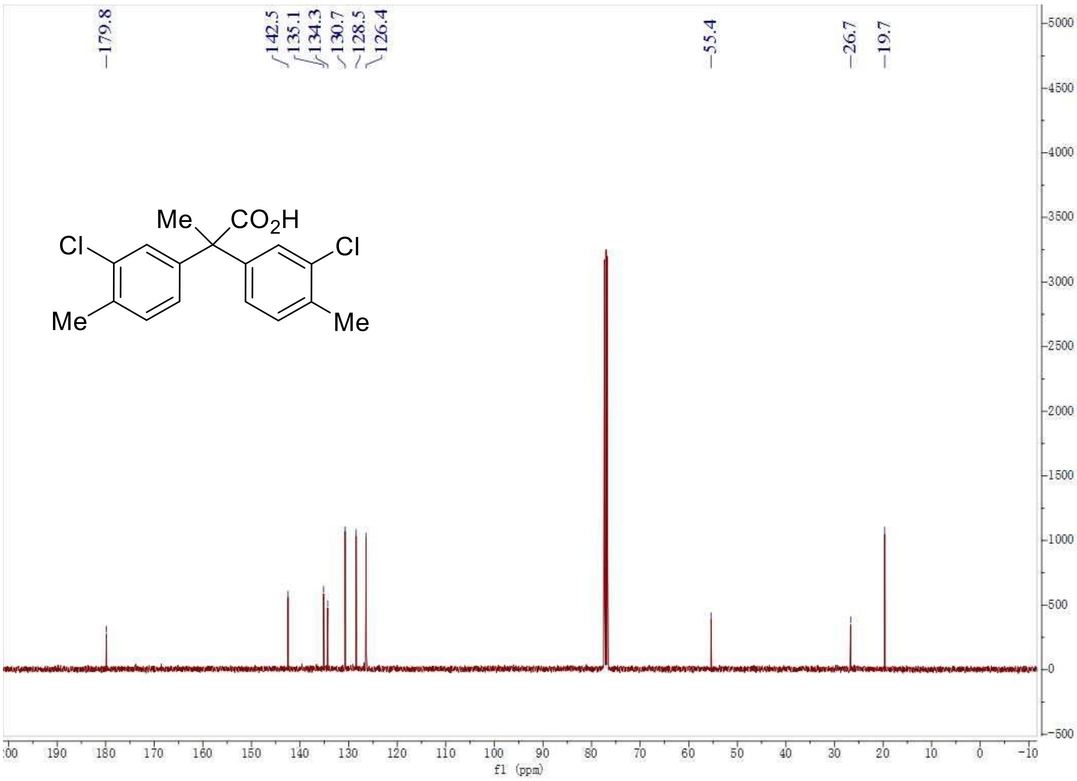

2,2-bis(3-chloro-5-methylphenyl)propanoic acid

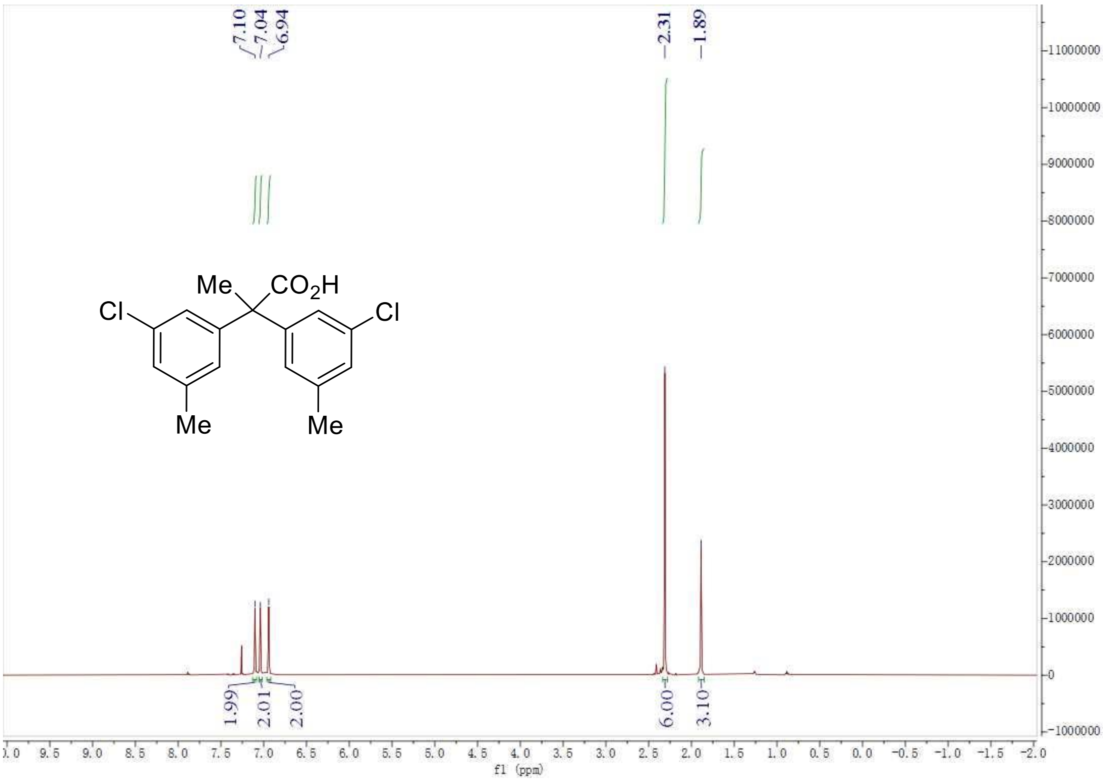

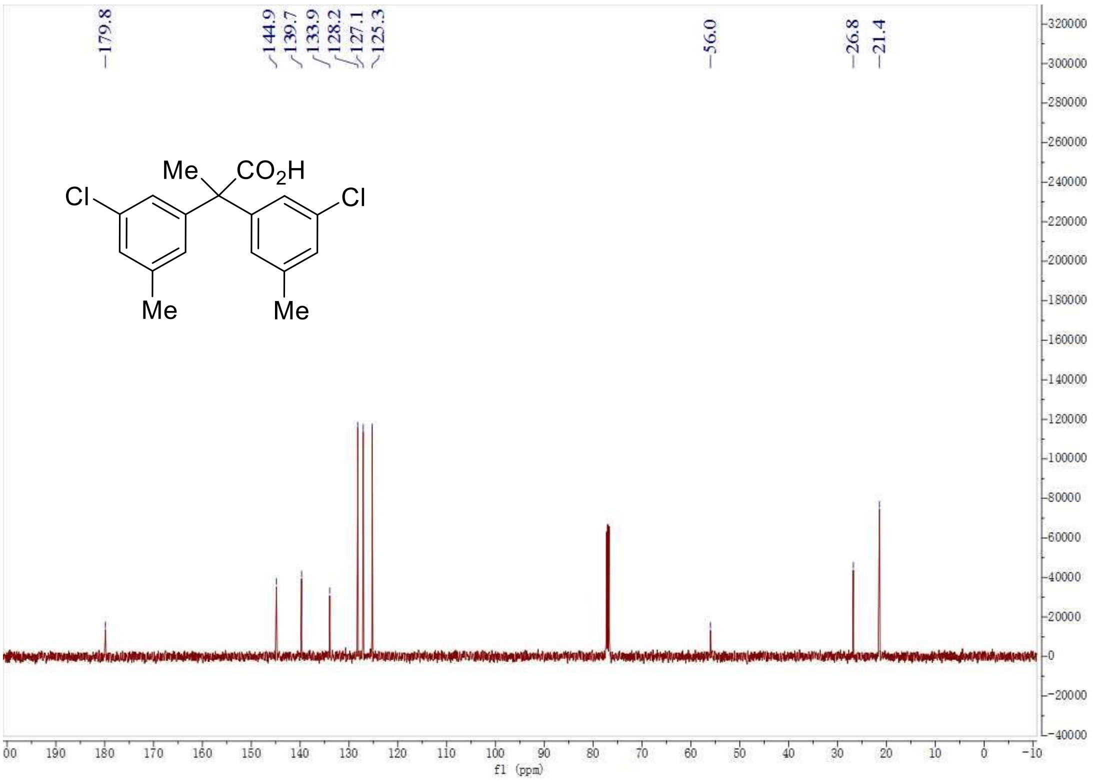

2,2-bis(2-chlorophenyl)acetic acid

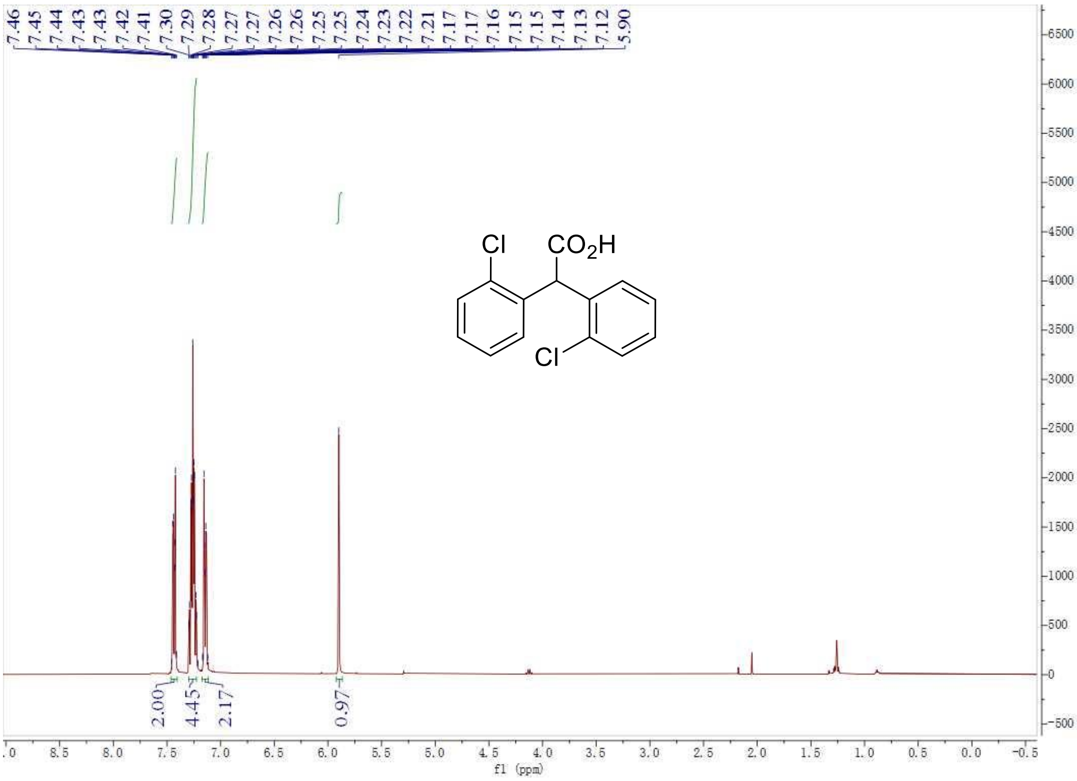

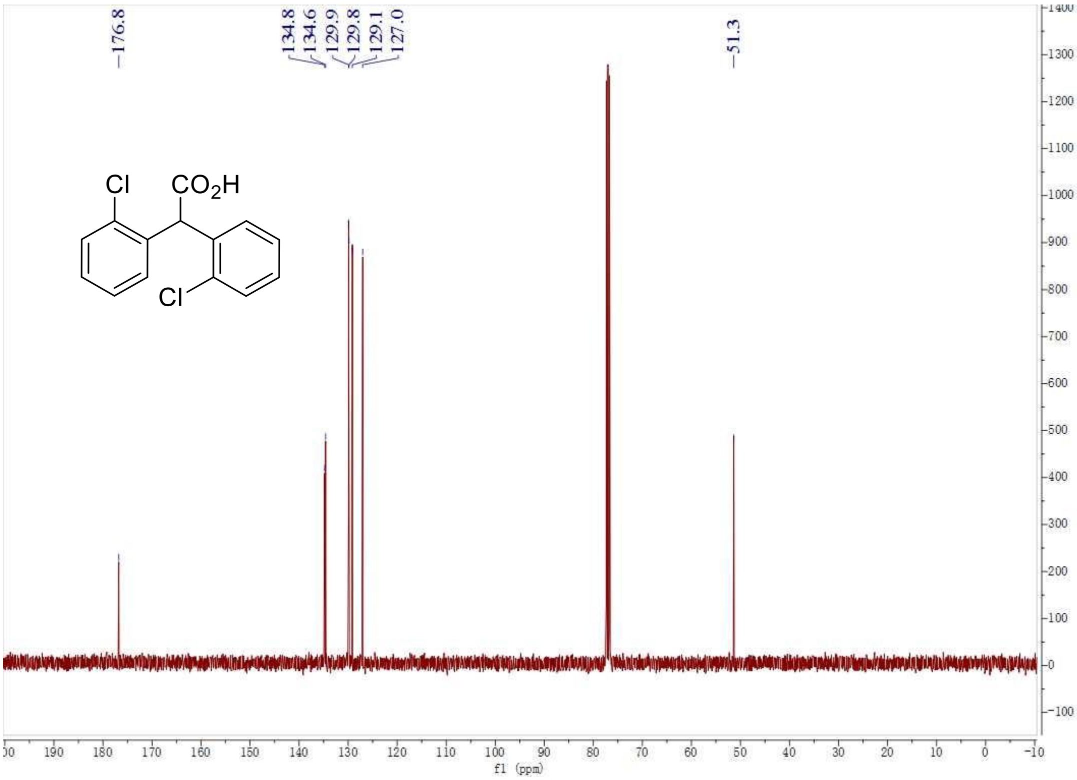

2,2-bis(2-chlorophenyl)propanoic acid

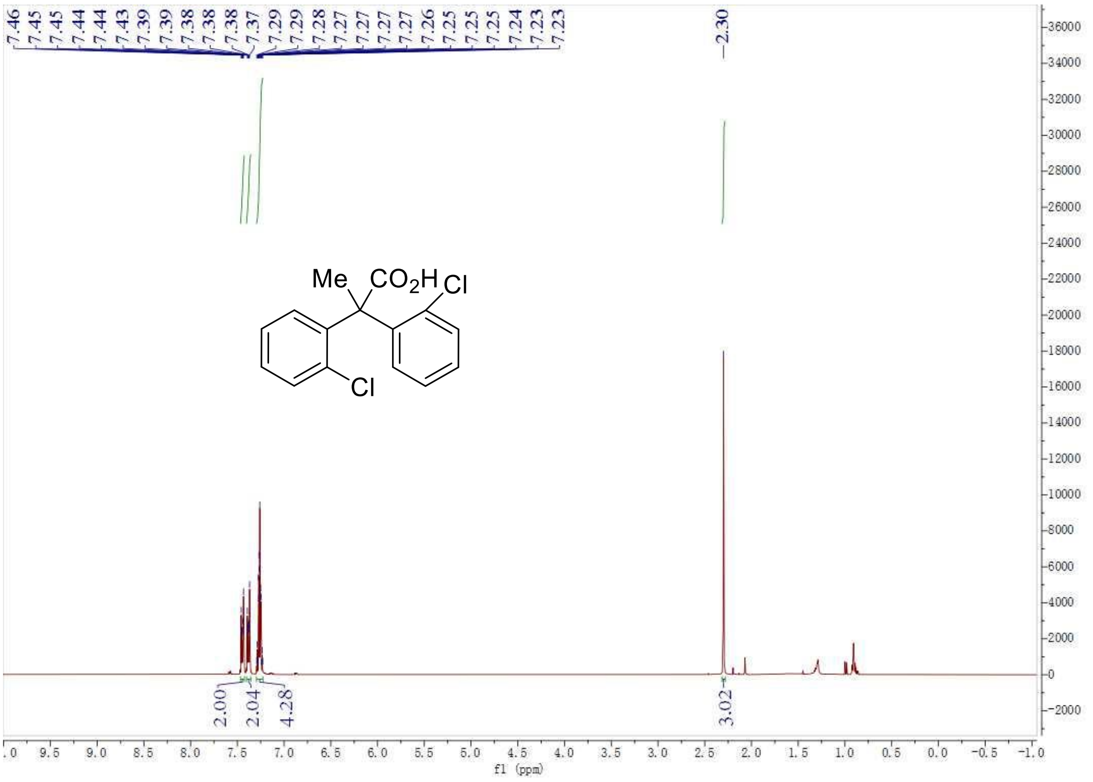

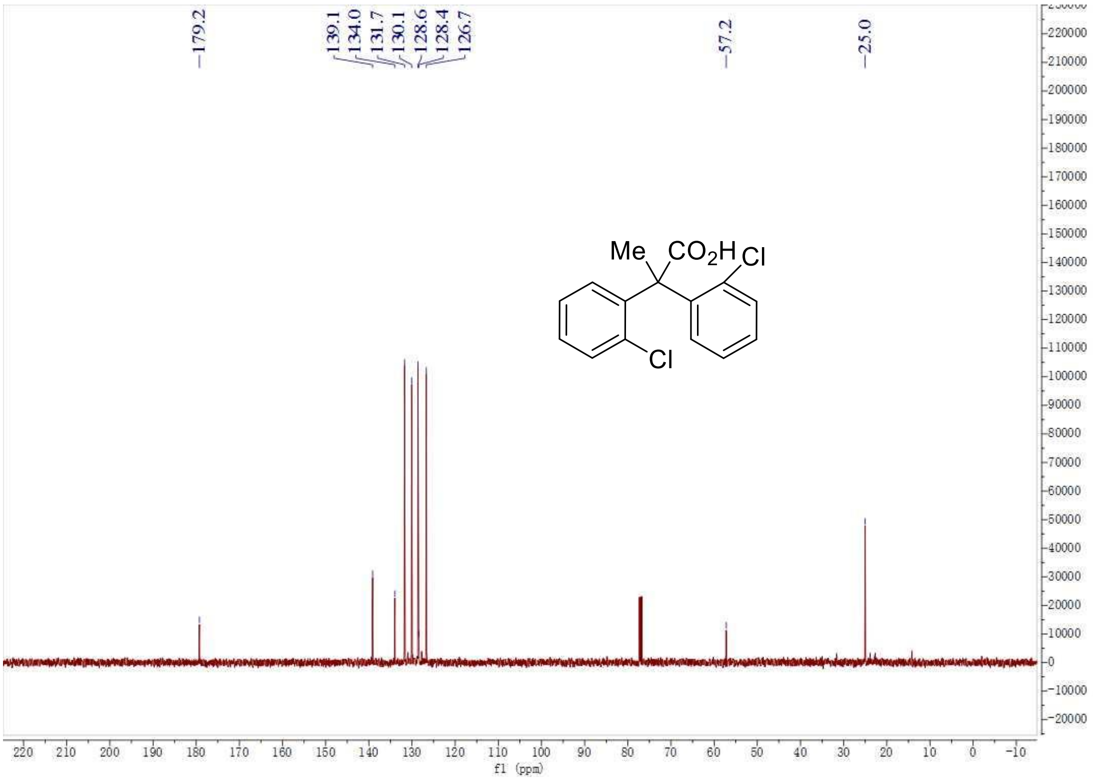

2,2-bis(4-chlorophenyl)propanoic acid

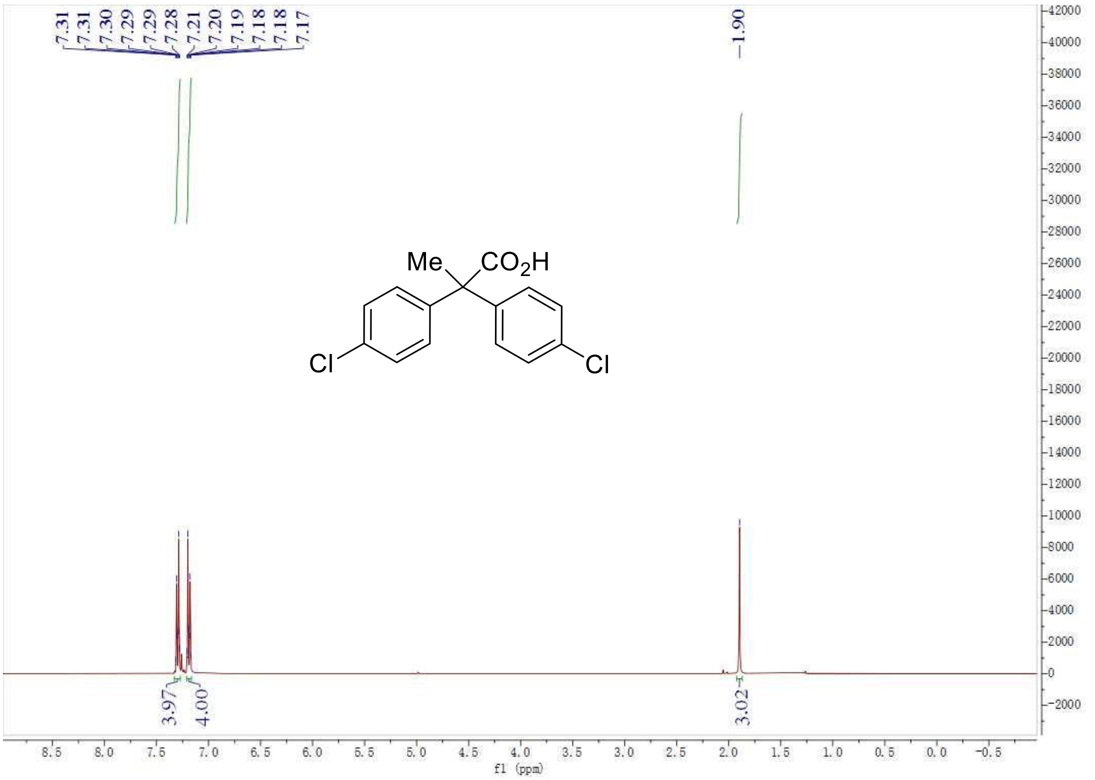

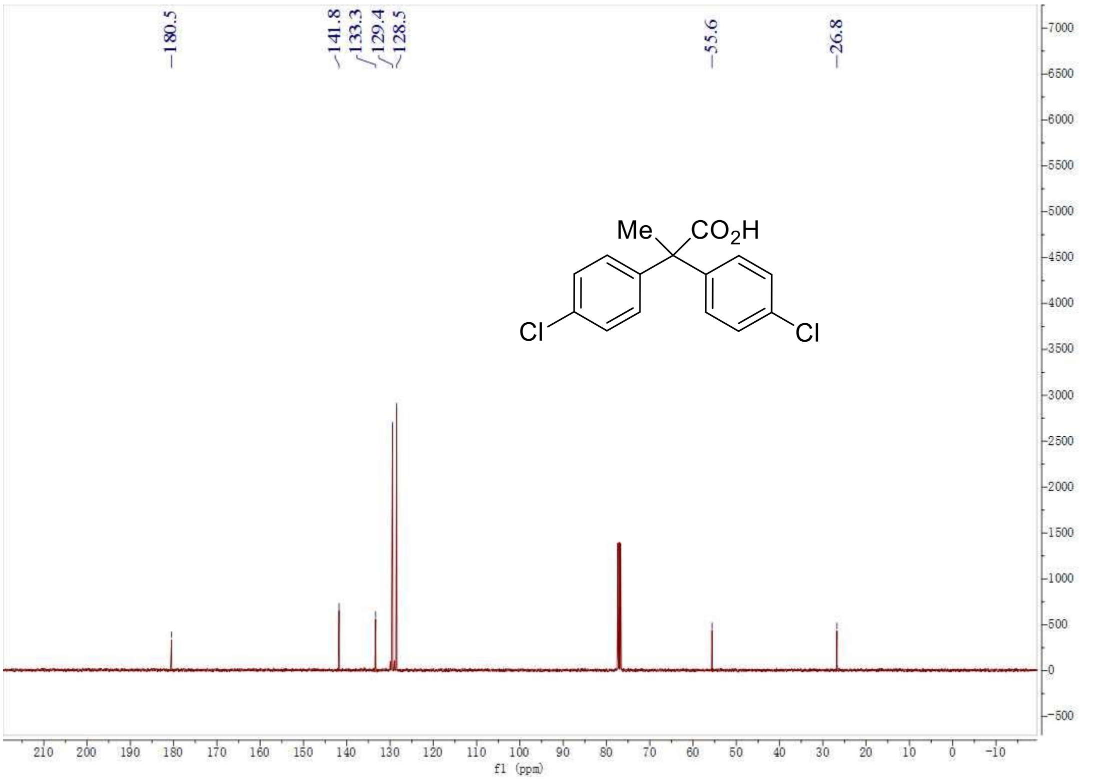

Methyl 2,2-bis(5-chloro-1-methyl-1H-indol-3-yl)propanoate

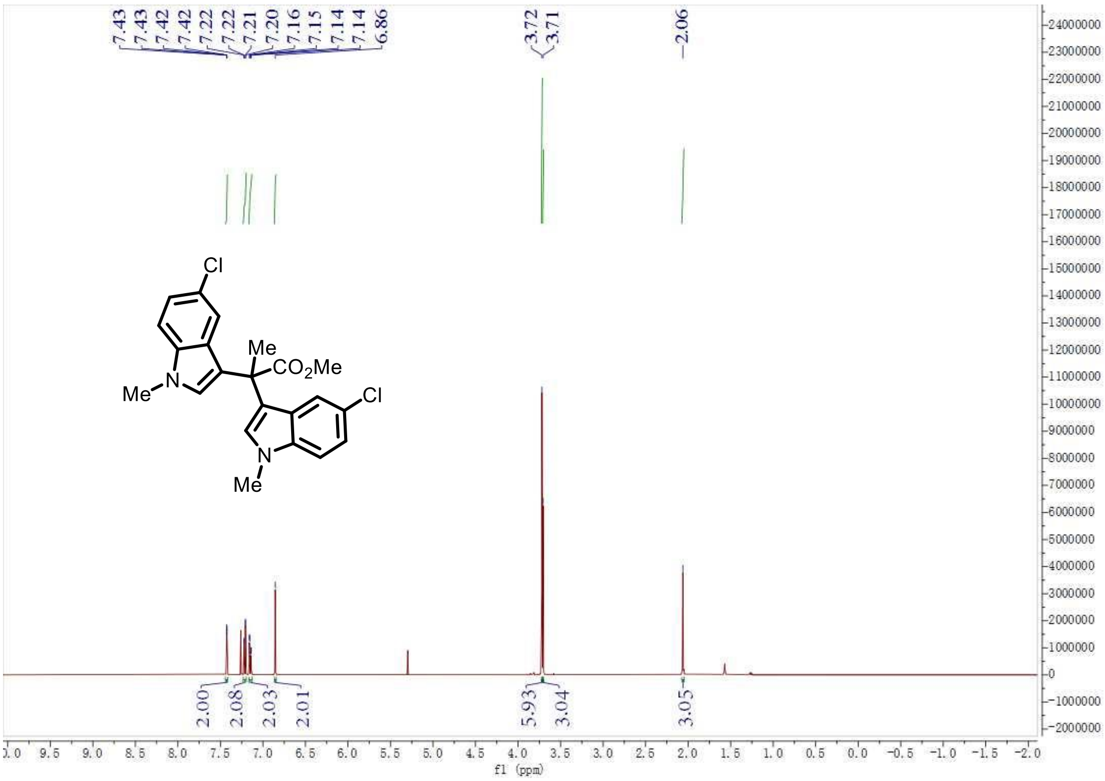

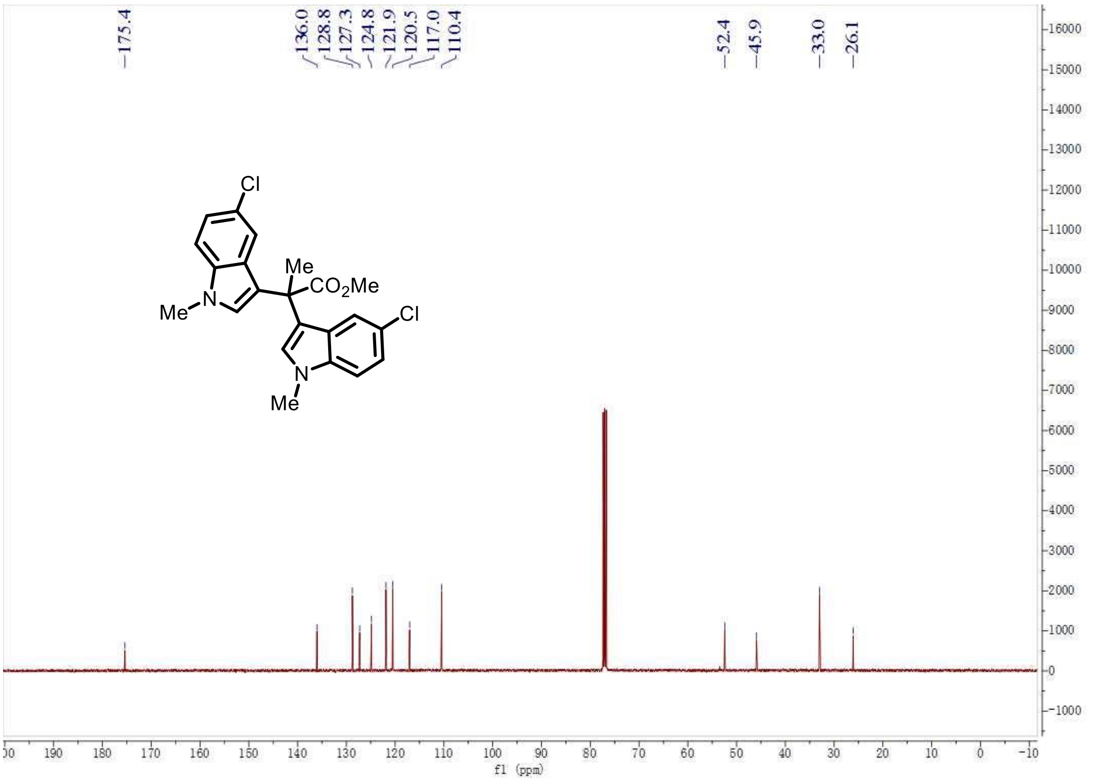

Ethyl 2,2-bis(5-chloro-1-ethyl-1H-indol-3-yl)-4-oxopentanoate

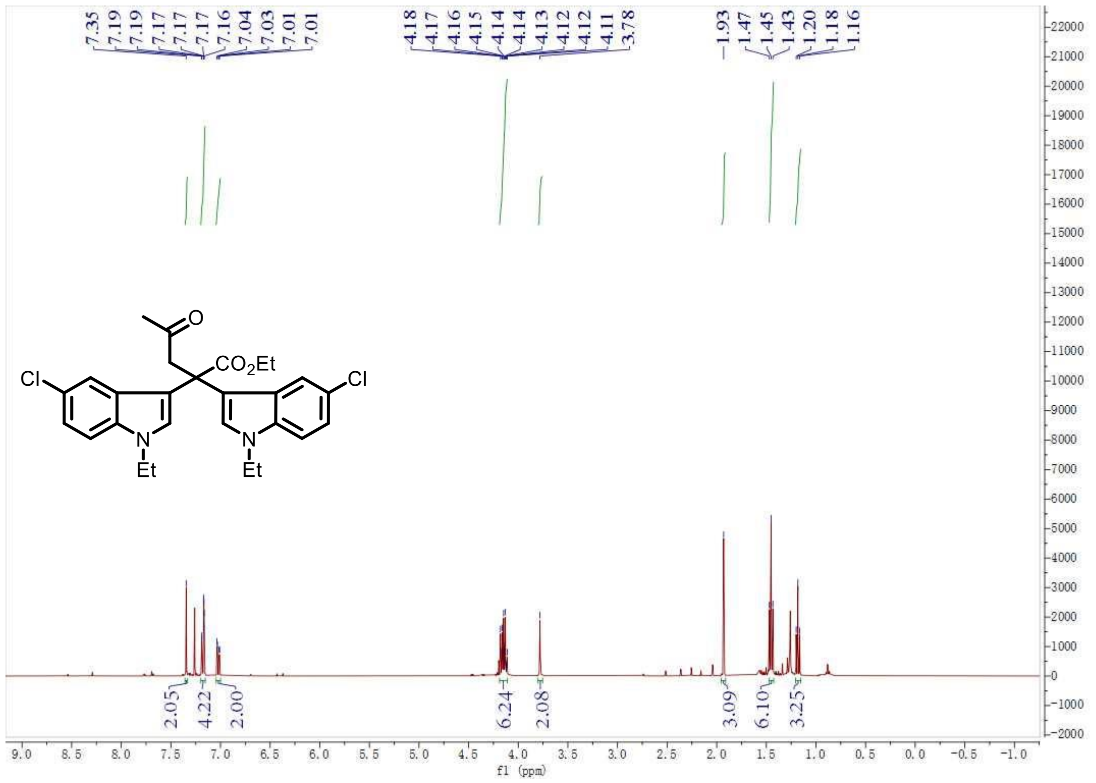

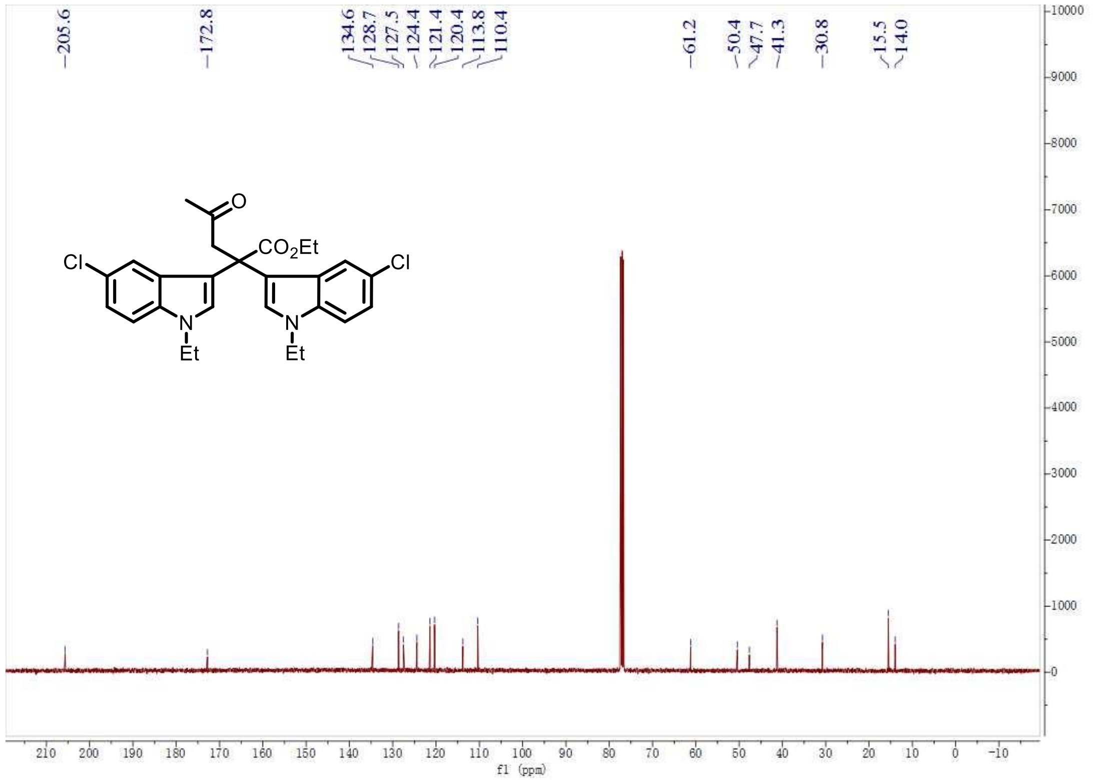

Methyl 2,2-bis(5-chloro-1-methyl-1H-indol-3-yl)-3-methylbutanoate

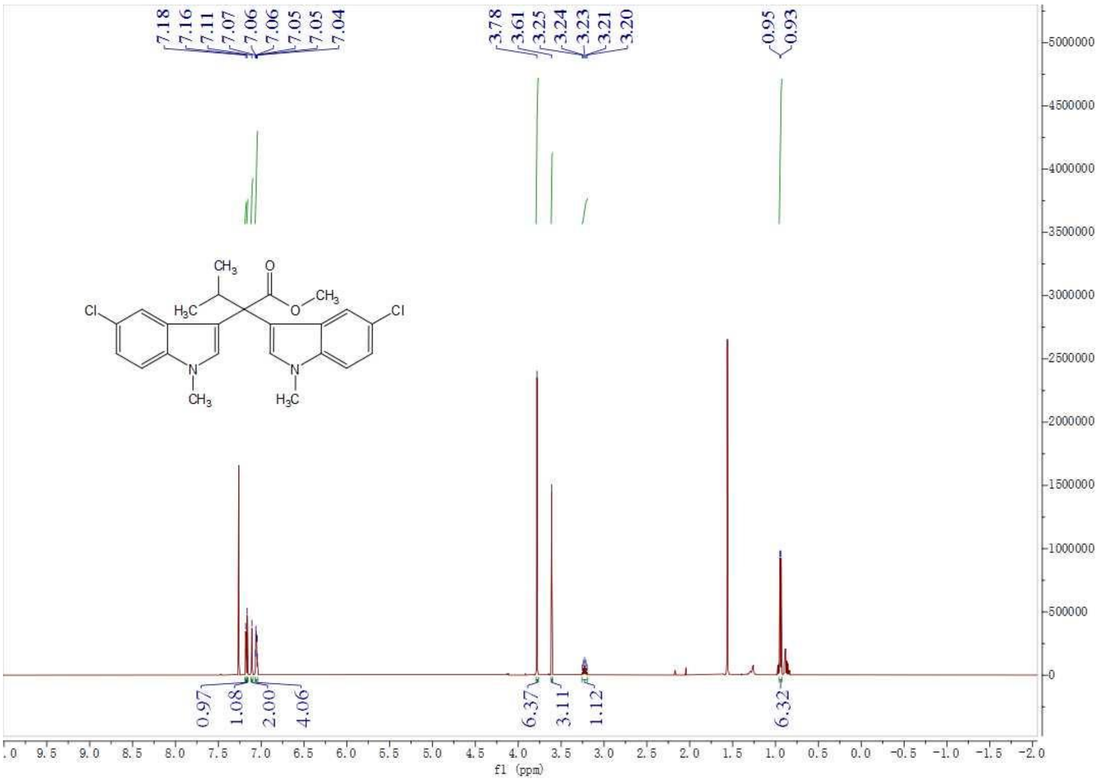

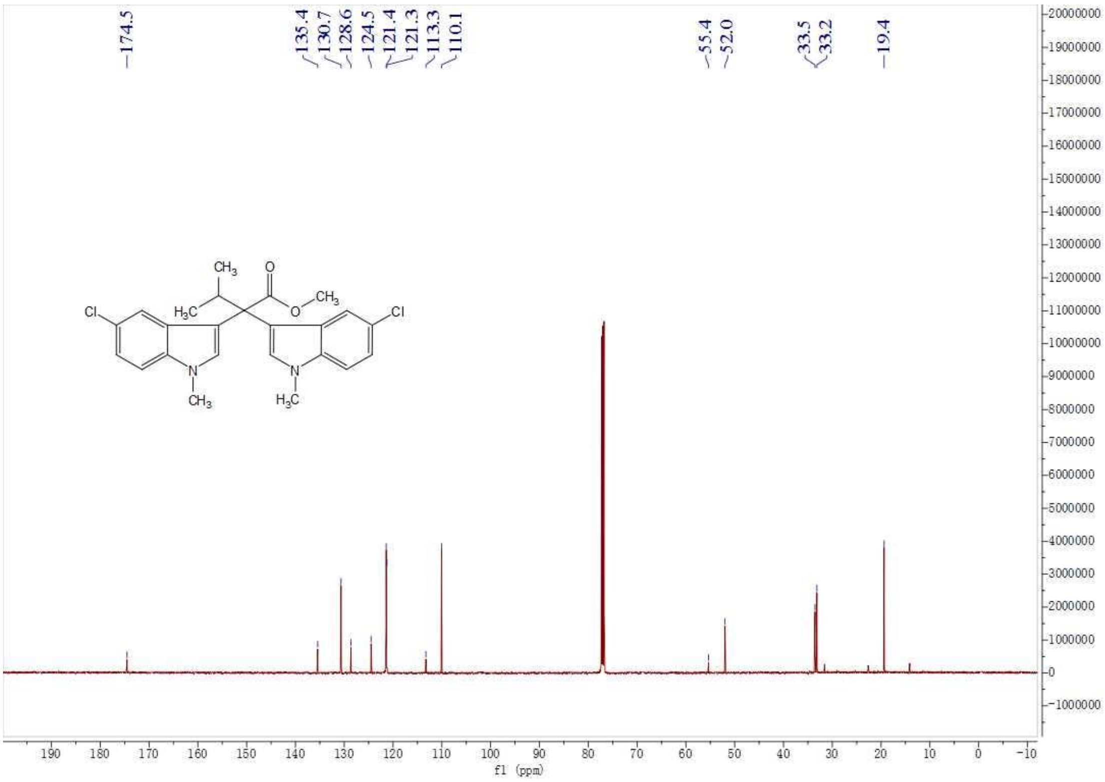

2,2-bis(3-chlorophenyl)-2-methoxyacetic acid

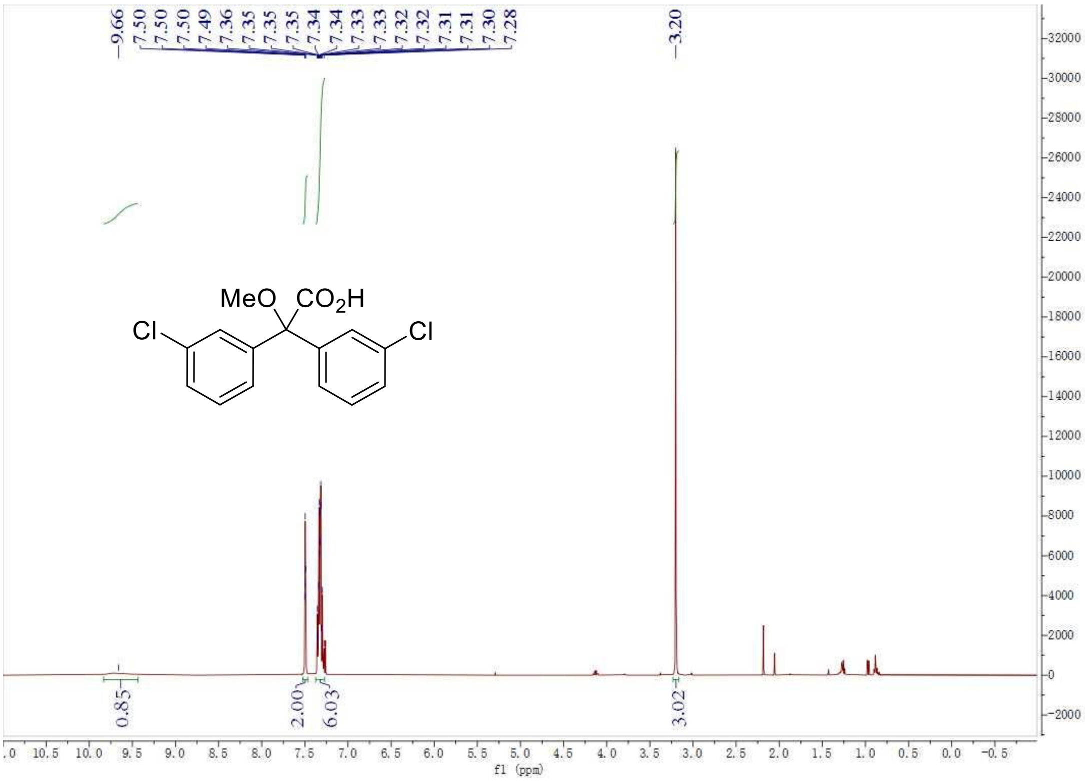

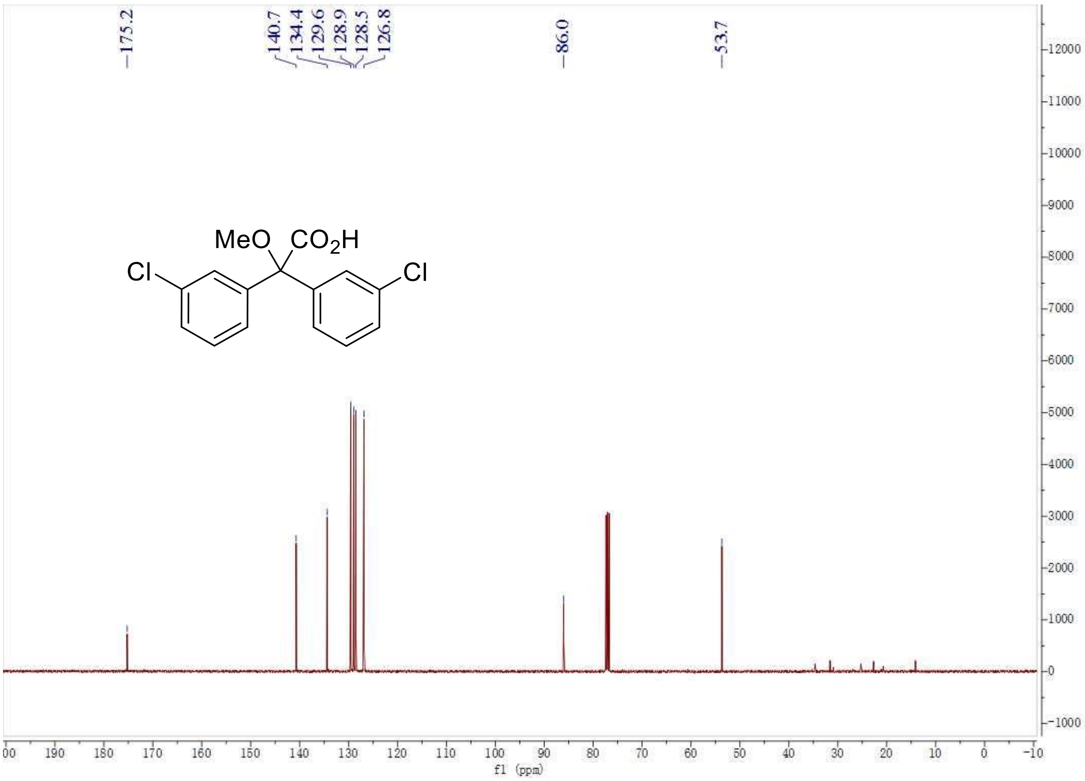

2,2-bis(3-chloro-4-methylphenyl)-2-methoxyacetic acid

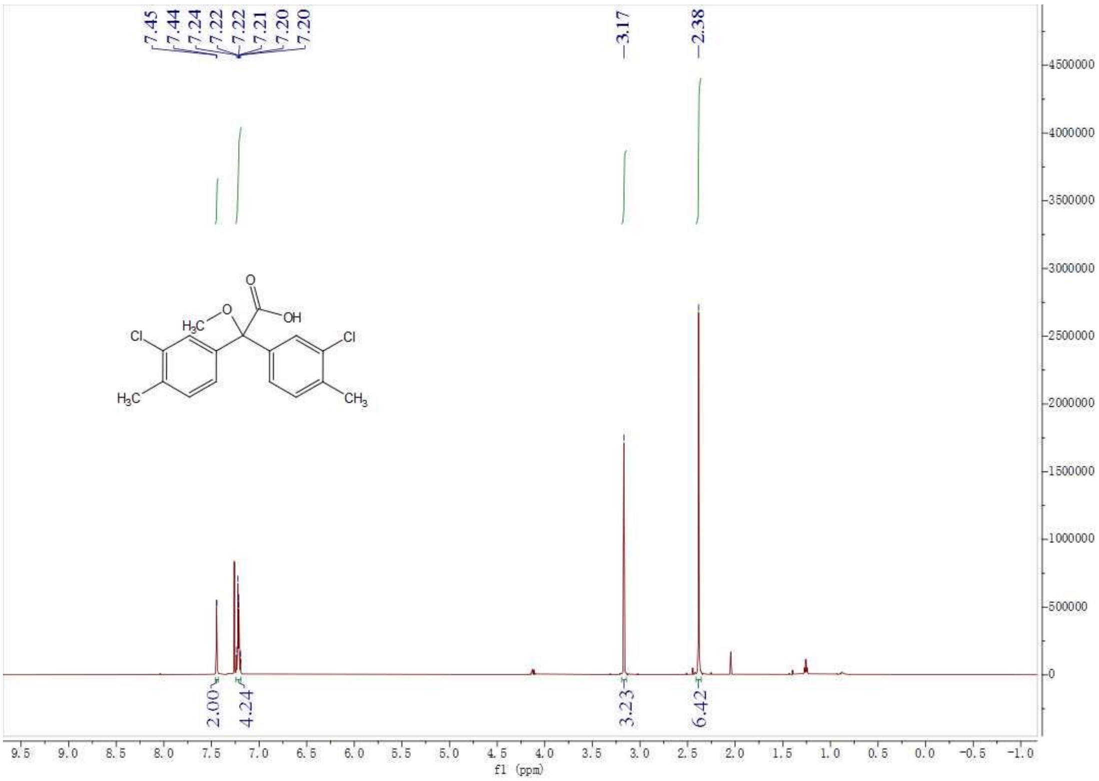

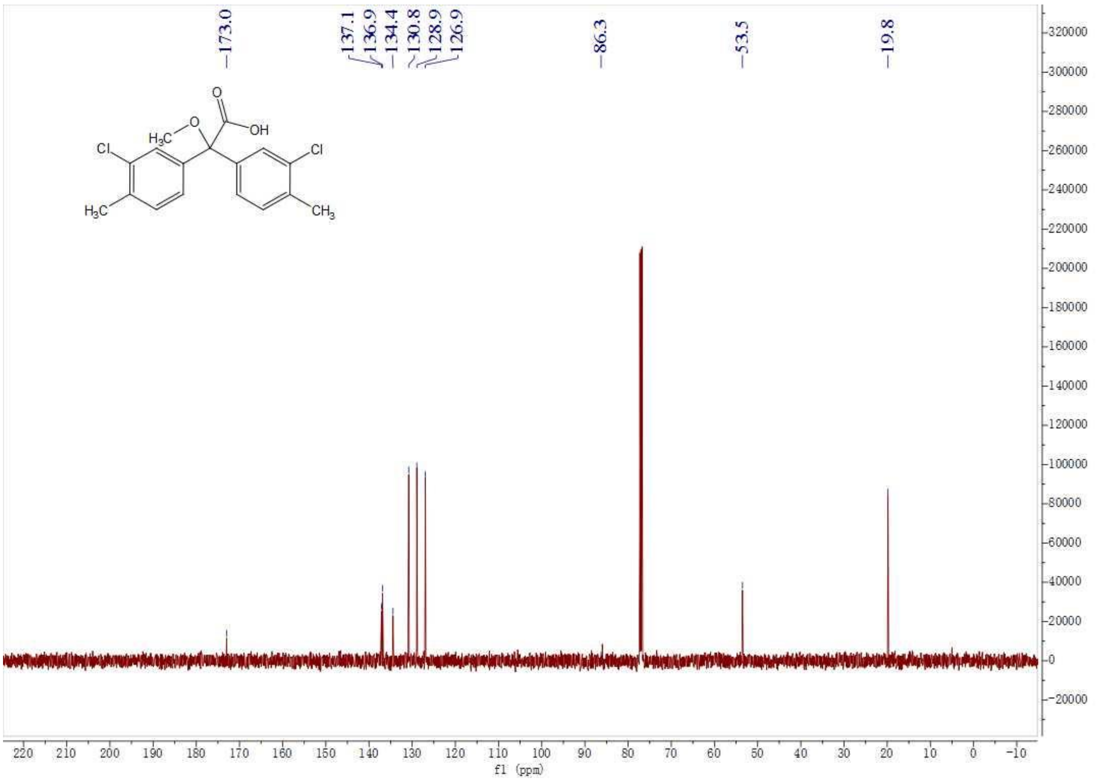

2,2-bis(3-chloro-4-methoxyphenyl)-2-methoxyacetic acid

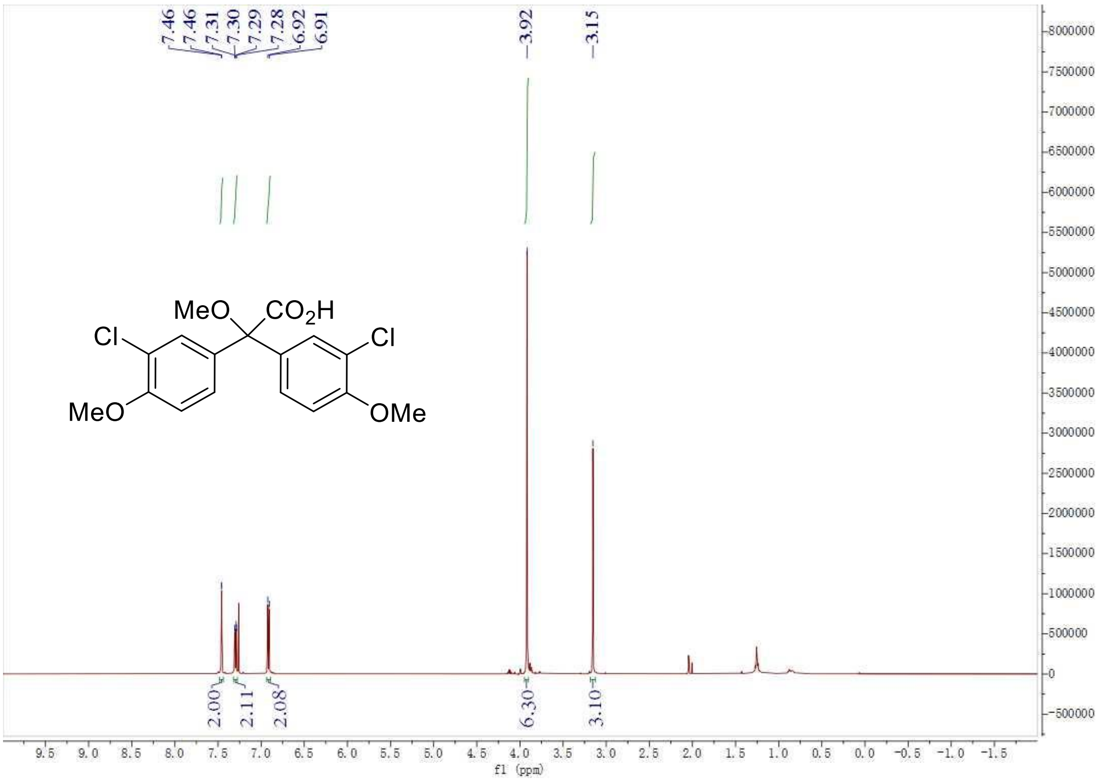

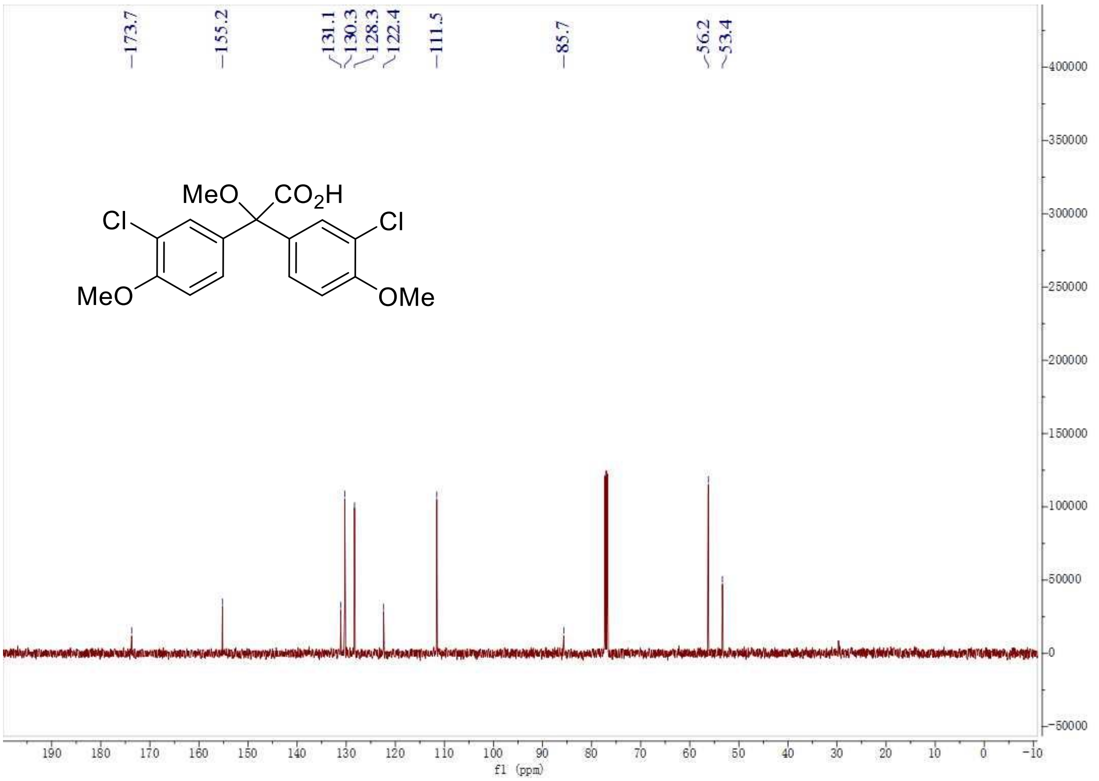

2,2-bis(3-chloro-4-methylphenyl)-2-fluoroacetic acid

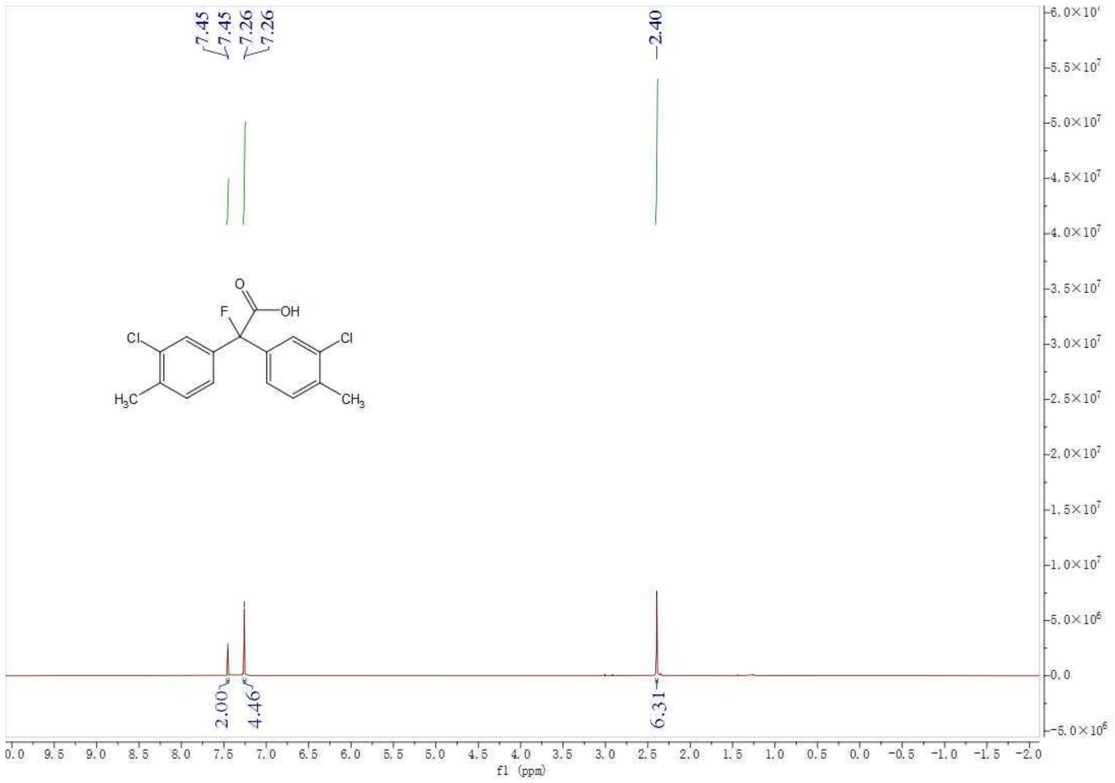

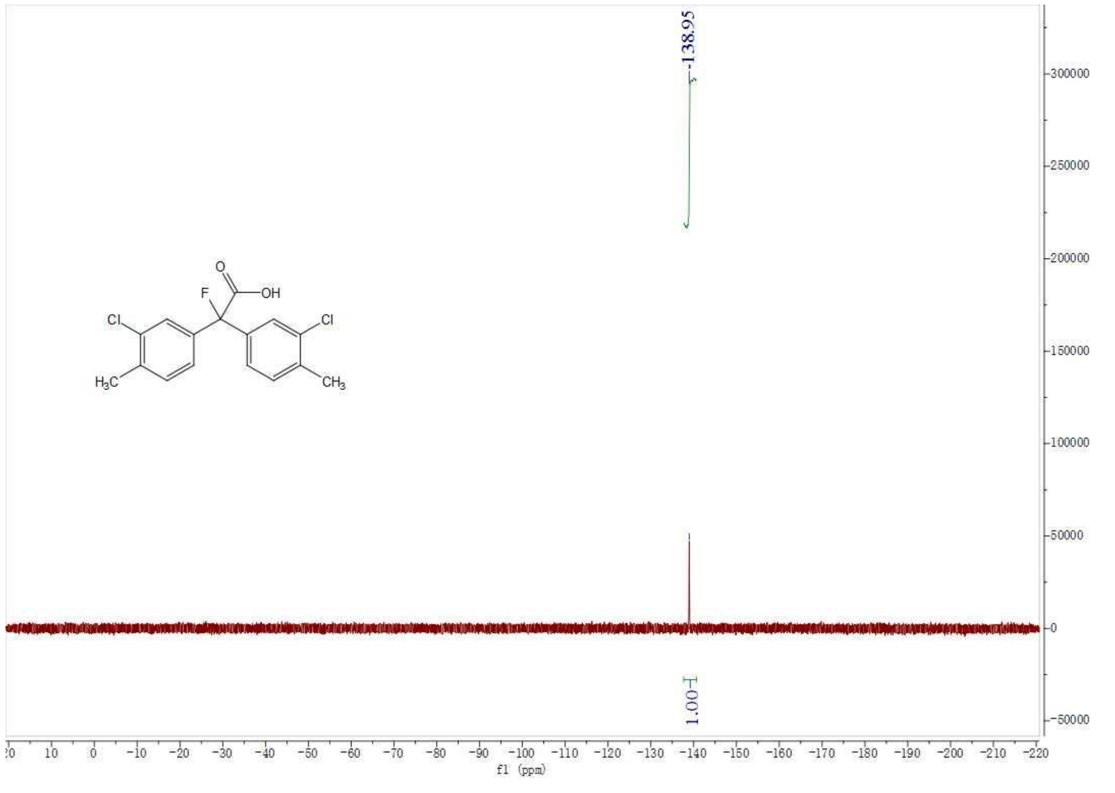

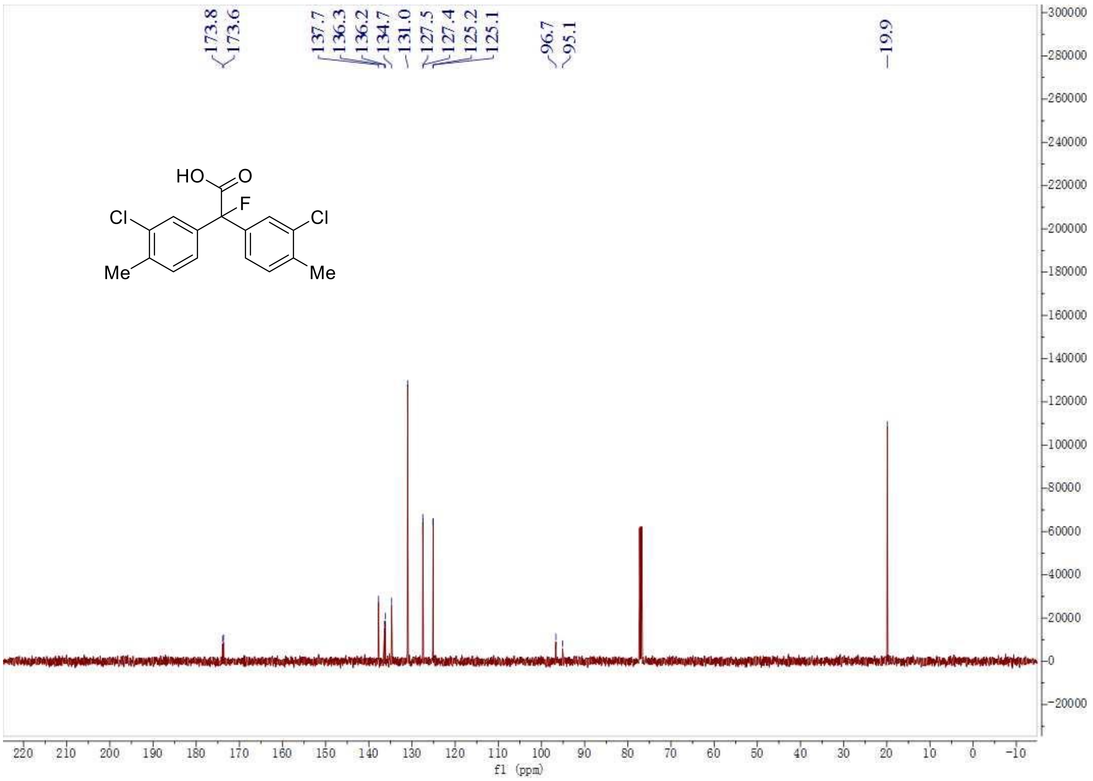
173.8
173.6
137.7
136.3
136.2
134.7
131.0
127.5
127.4
125.2
125.1
96.7
95.1
19.9
HO
O
F
Cl
Cl
Me
Me
f1 (ppm)

2-acetamido-2,2-bis(3-chloro-4-methoxy- phenyl)acetic acid

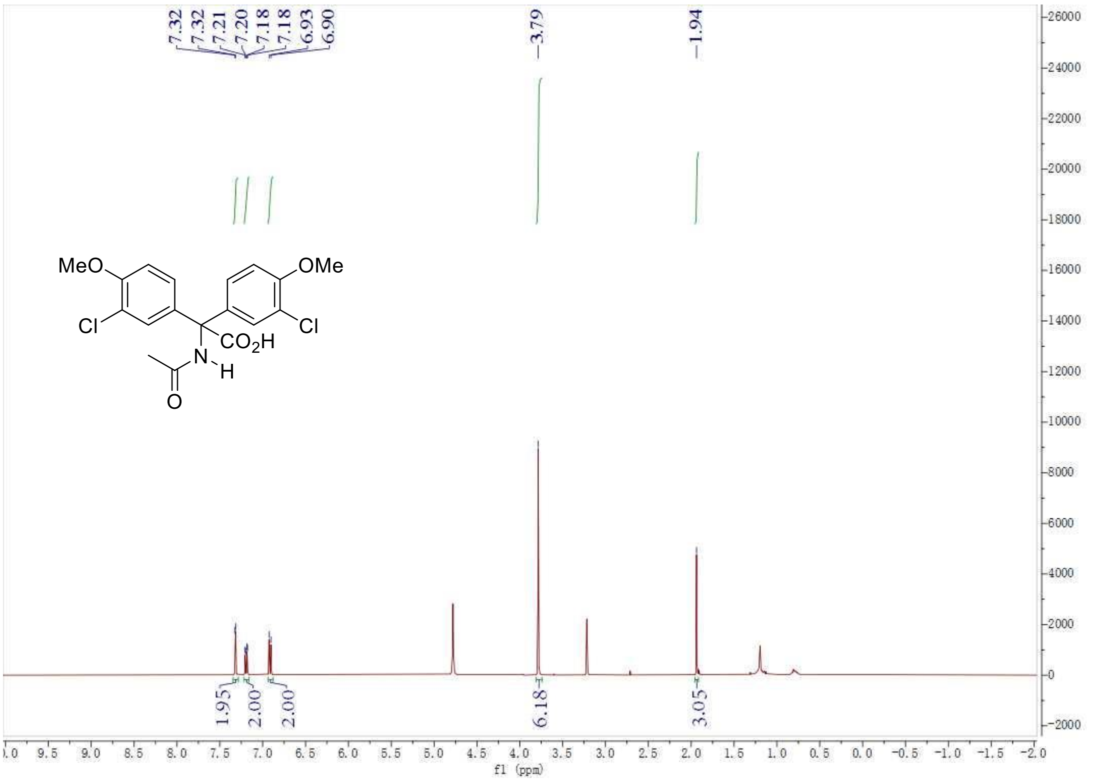

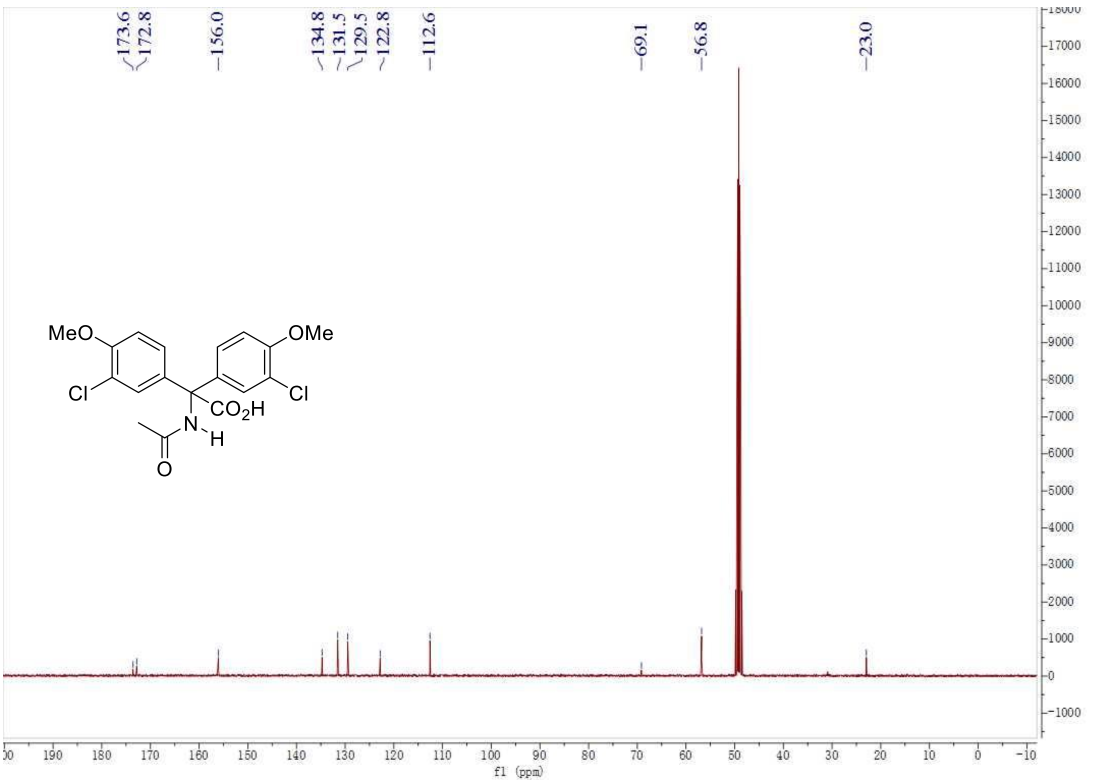

3,3-bis(3-chloro-4-methoxyphenyl)butanoic acid

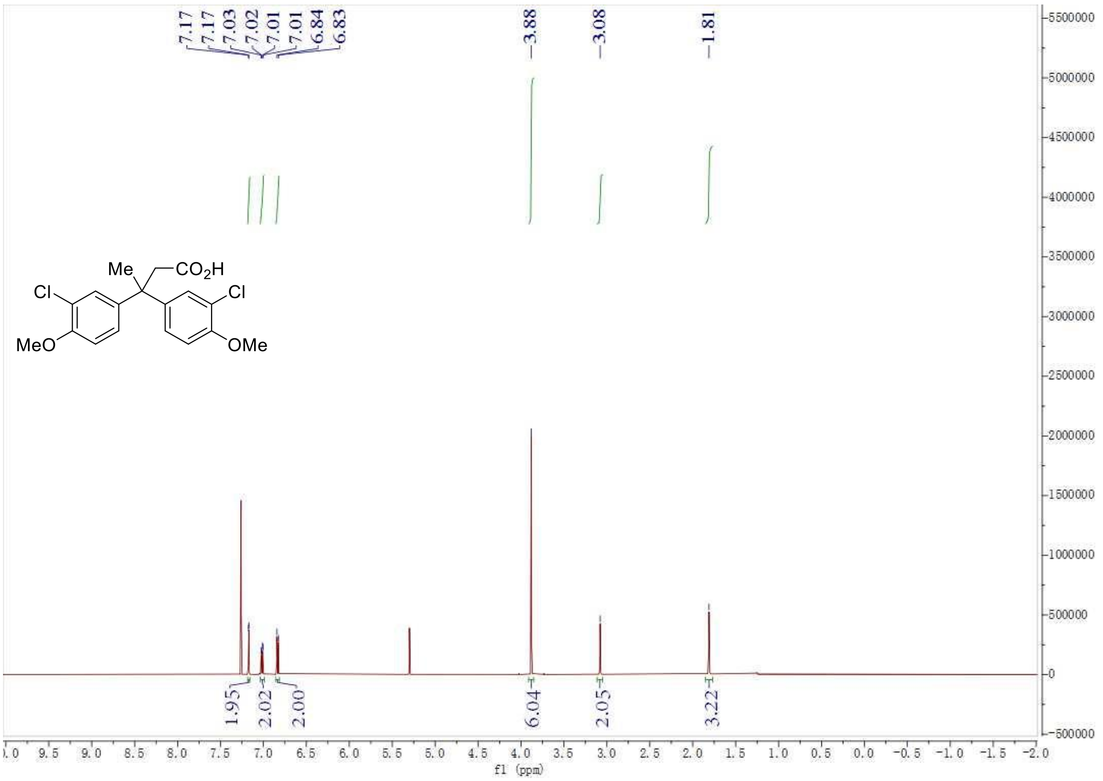

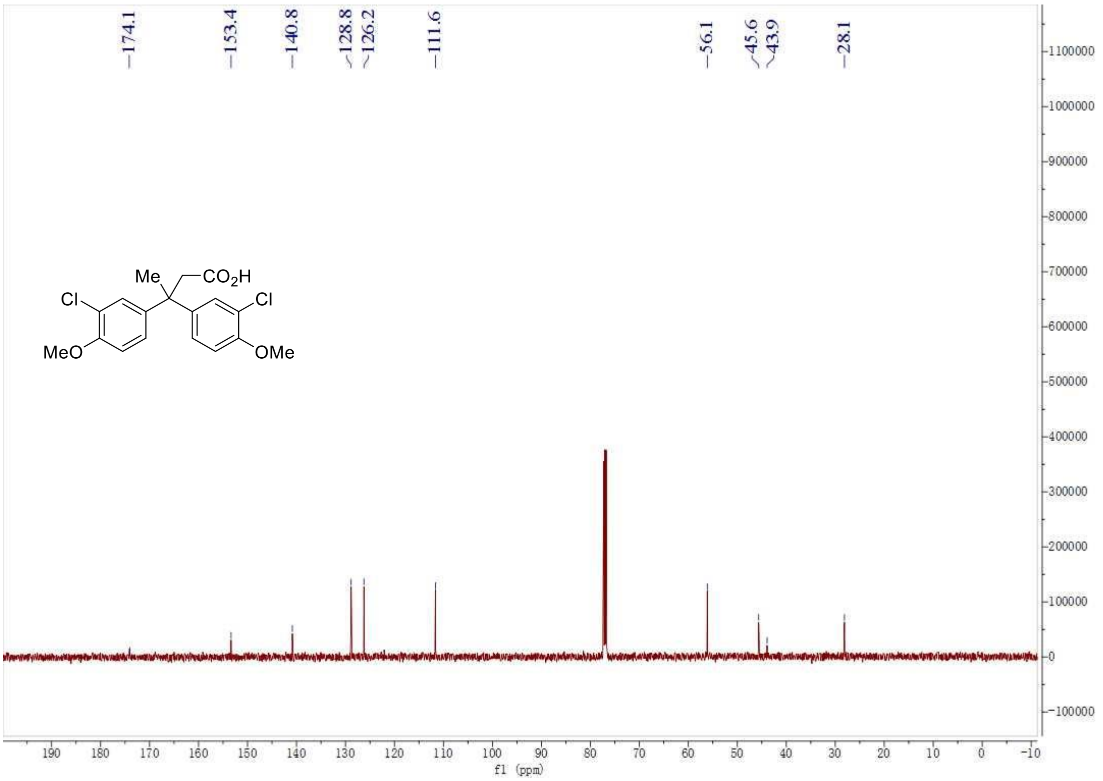

4,4-bis(3-chloro-4-methoxyphenyl)pentanoic acid

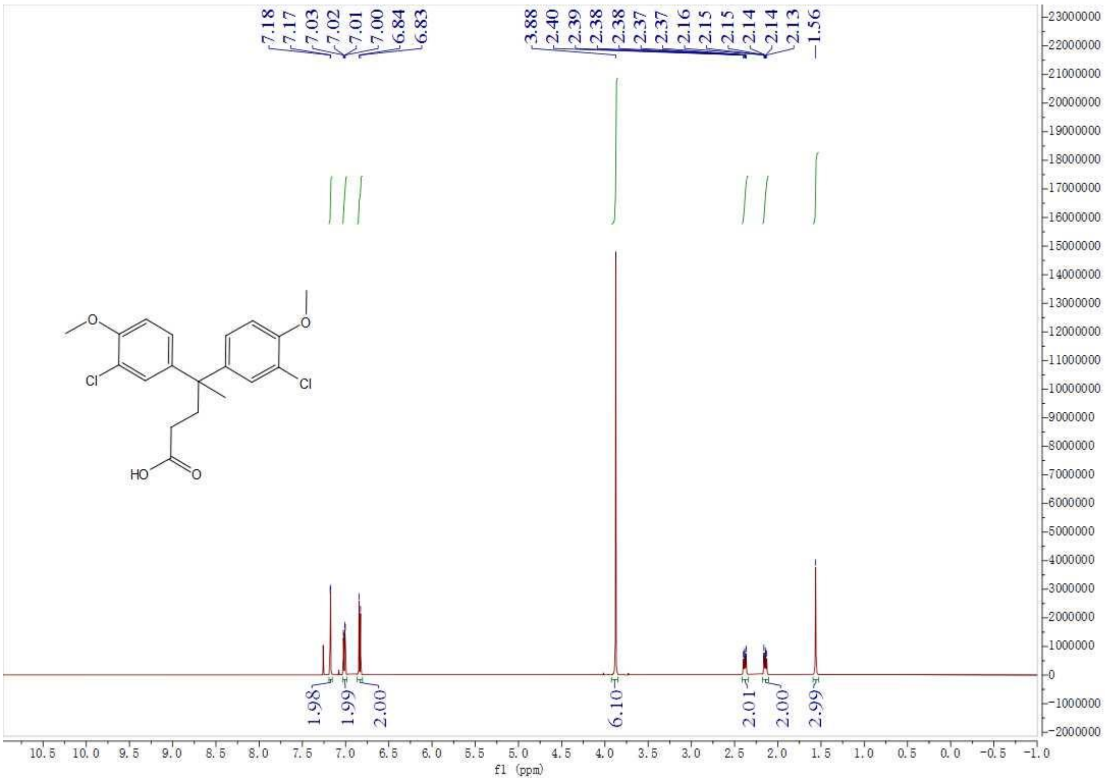

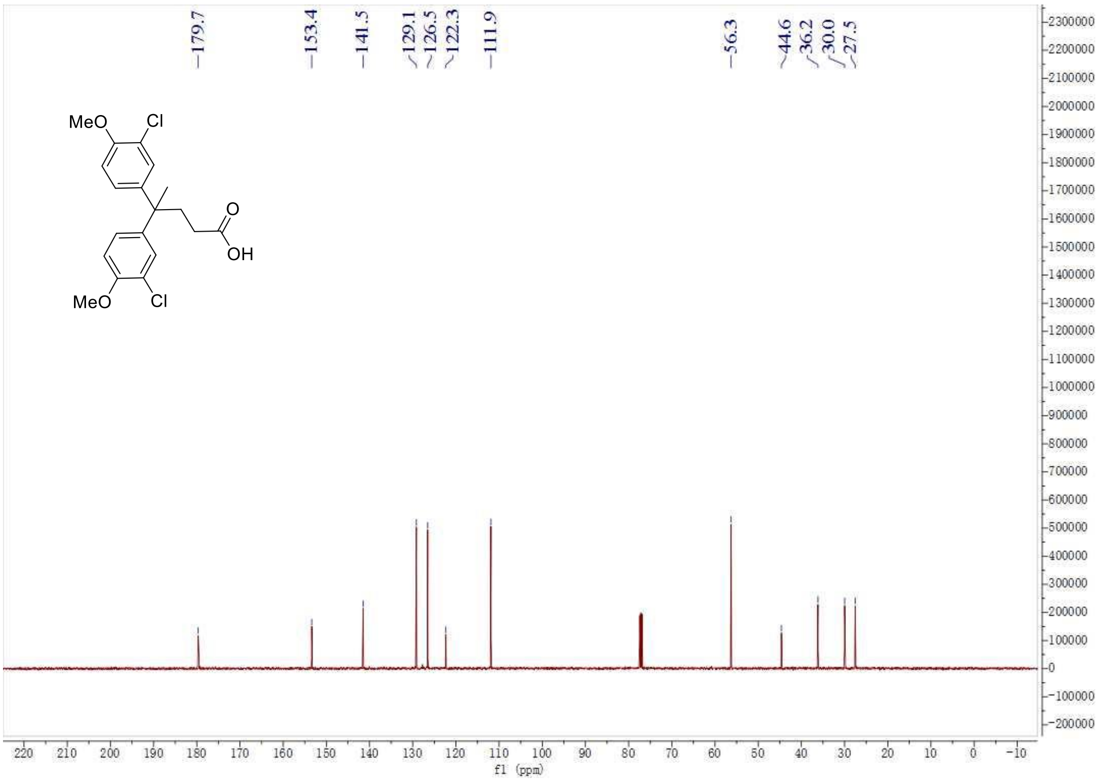

2-(3-chloro-4-methoxyphenyl)-2-(4-methoxyphenyl)propanoic acid

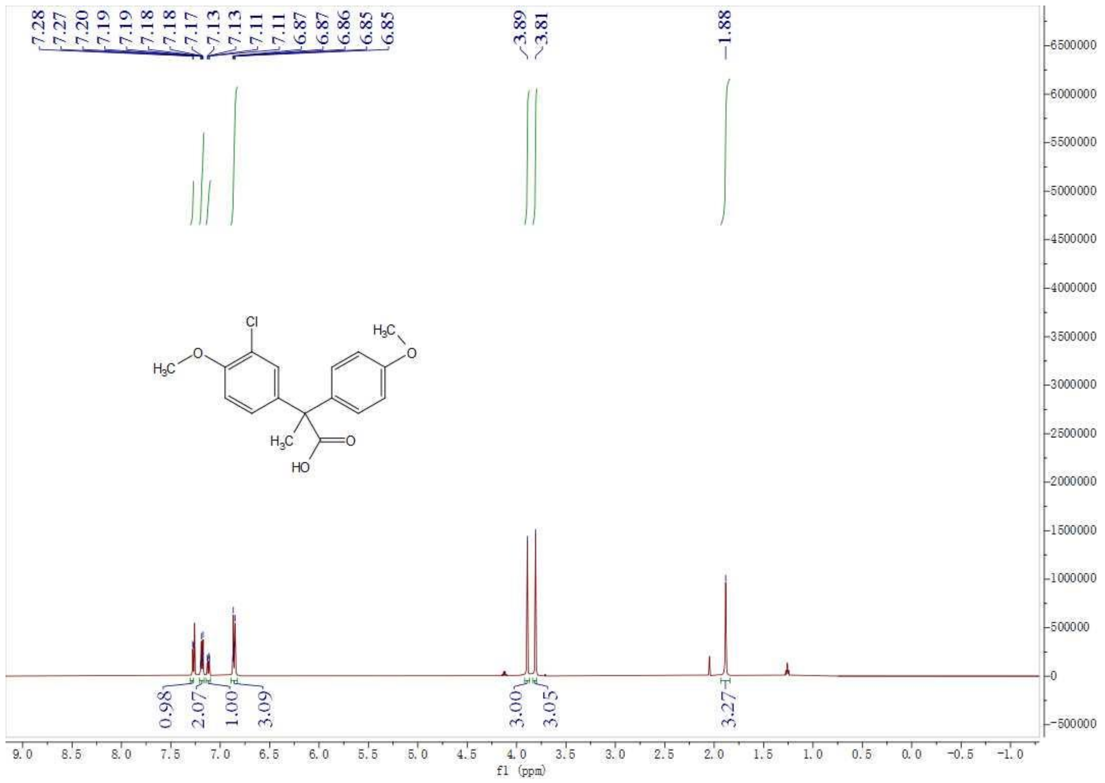

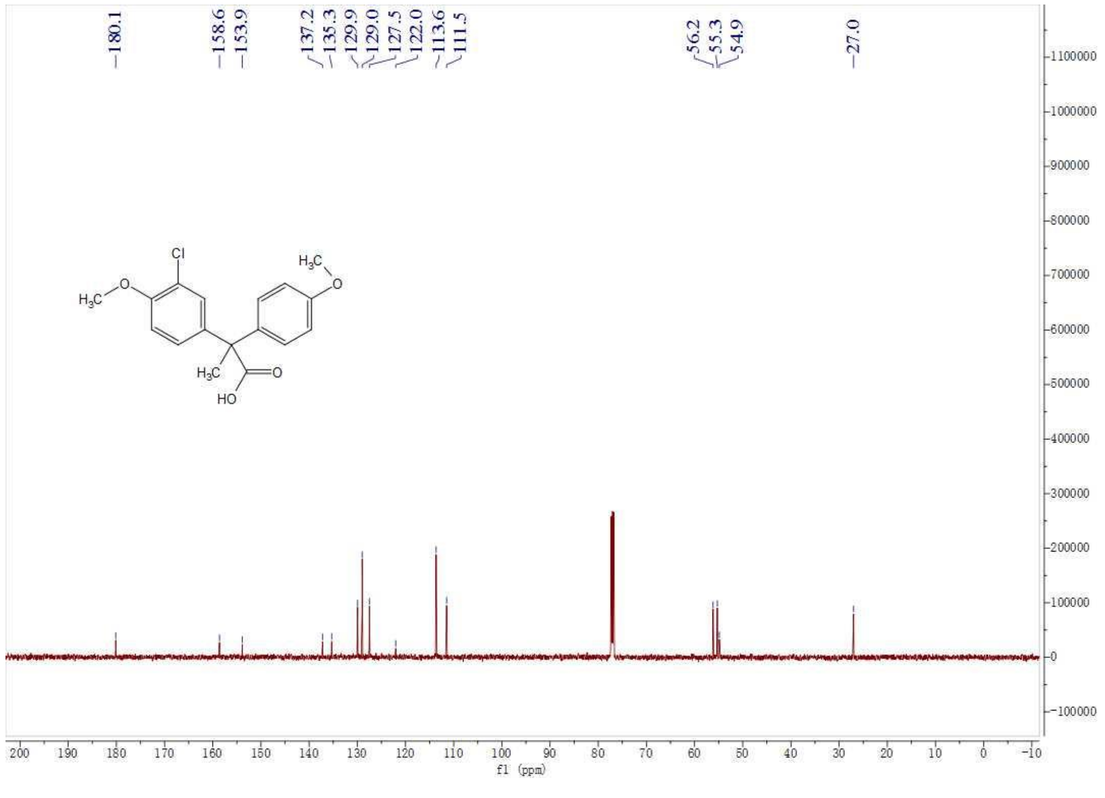

2-(3-chloro-4-methoxyphenyl)-2,3-dimethyl- butanoic acid

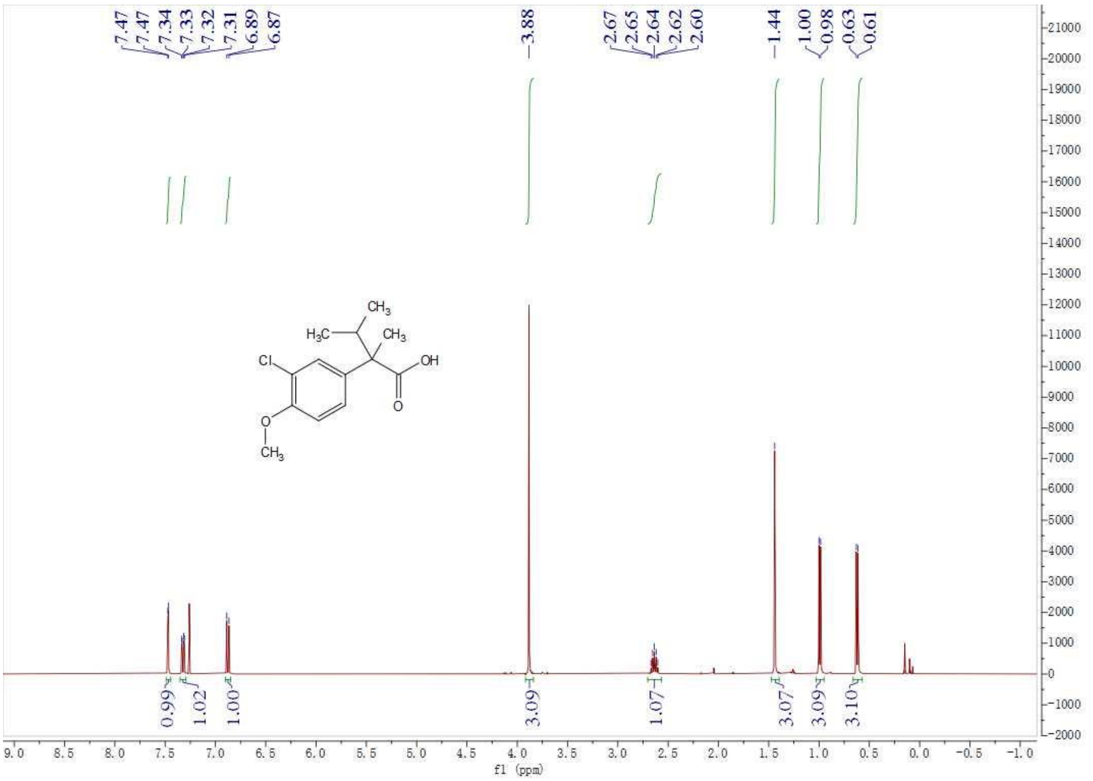

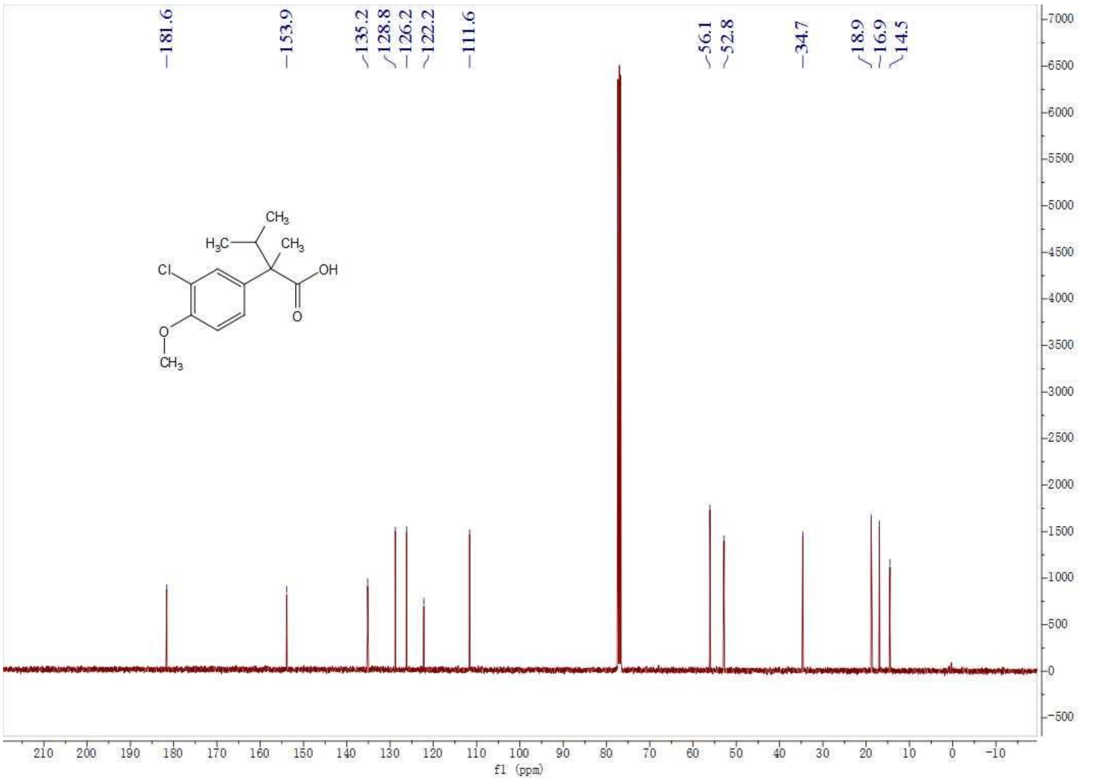

1

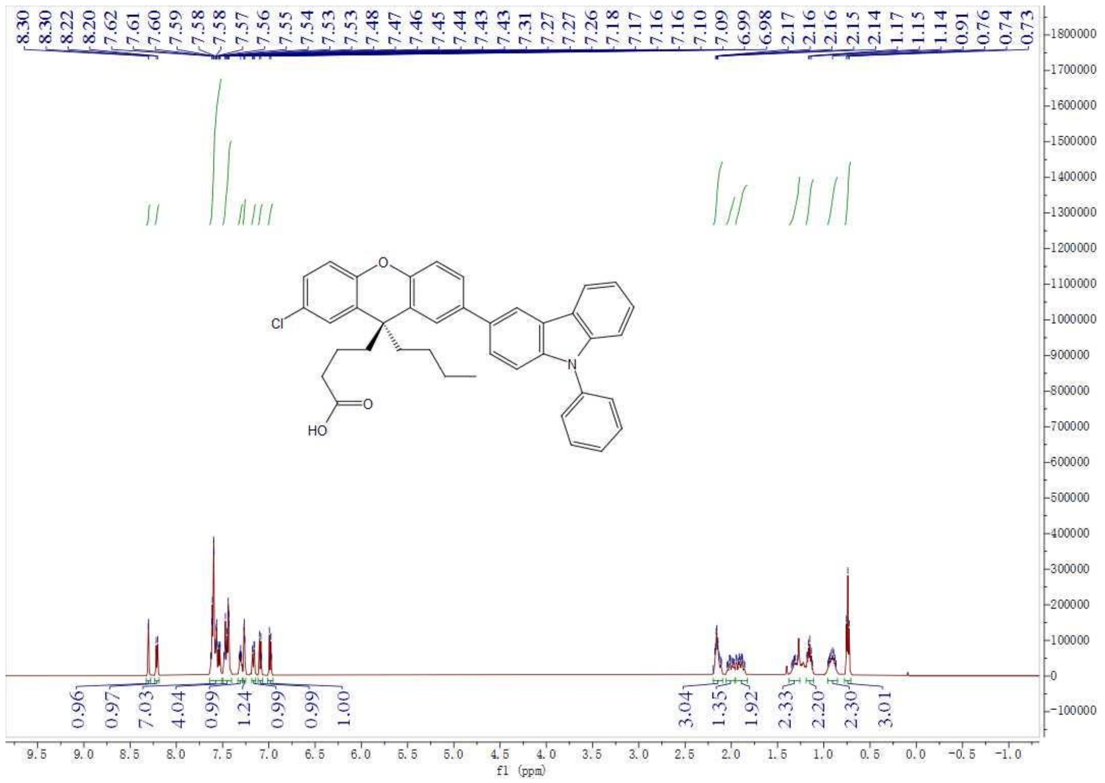

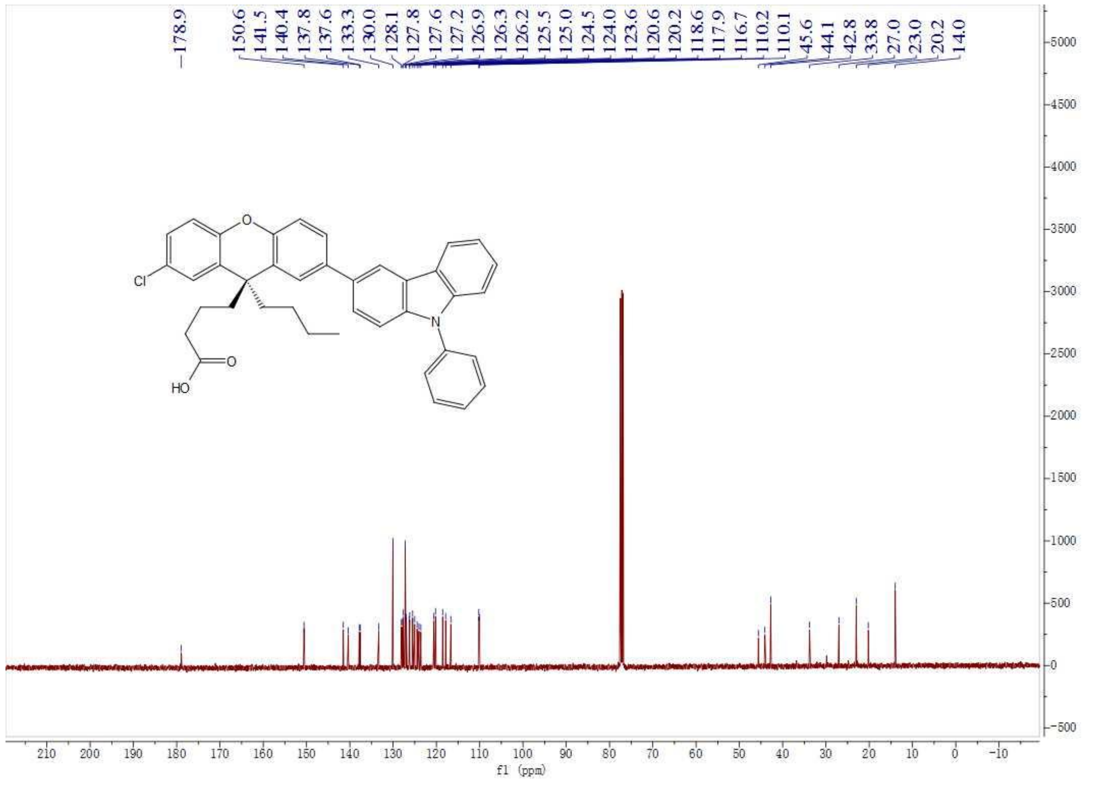

2

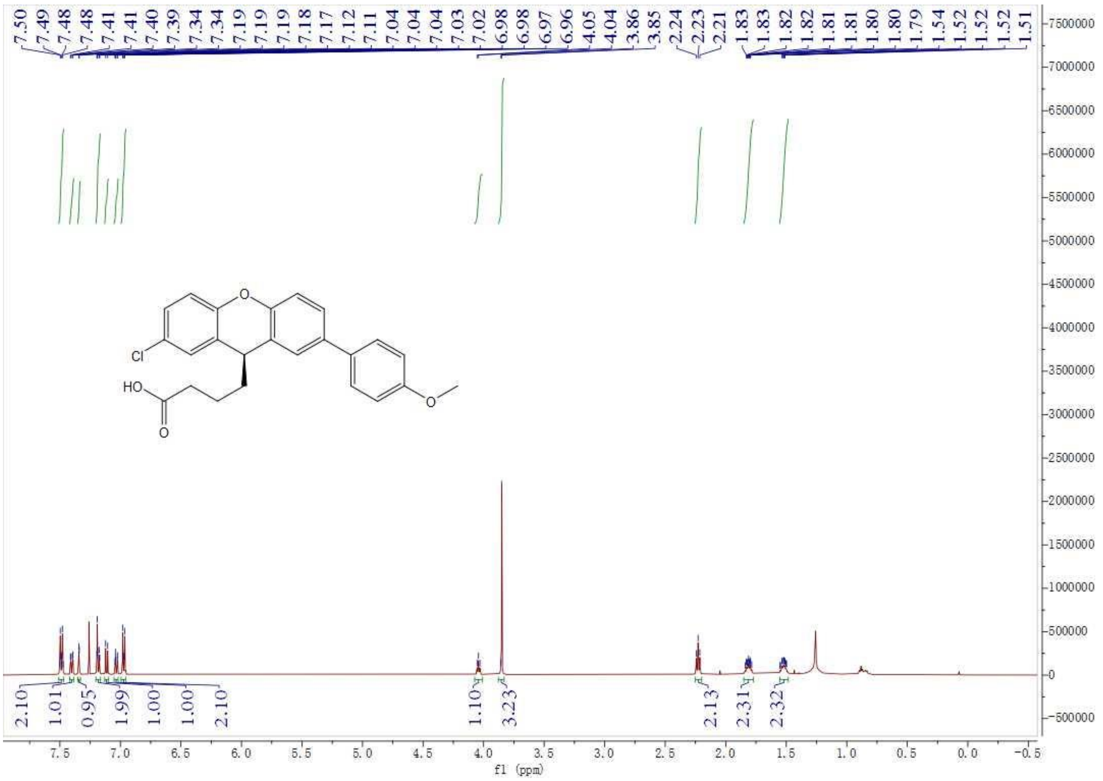

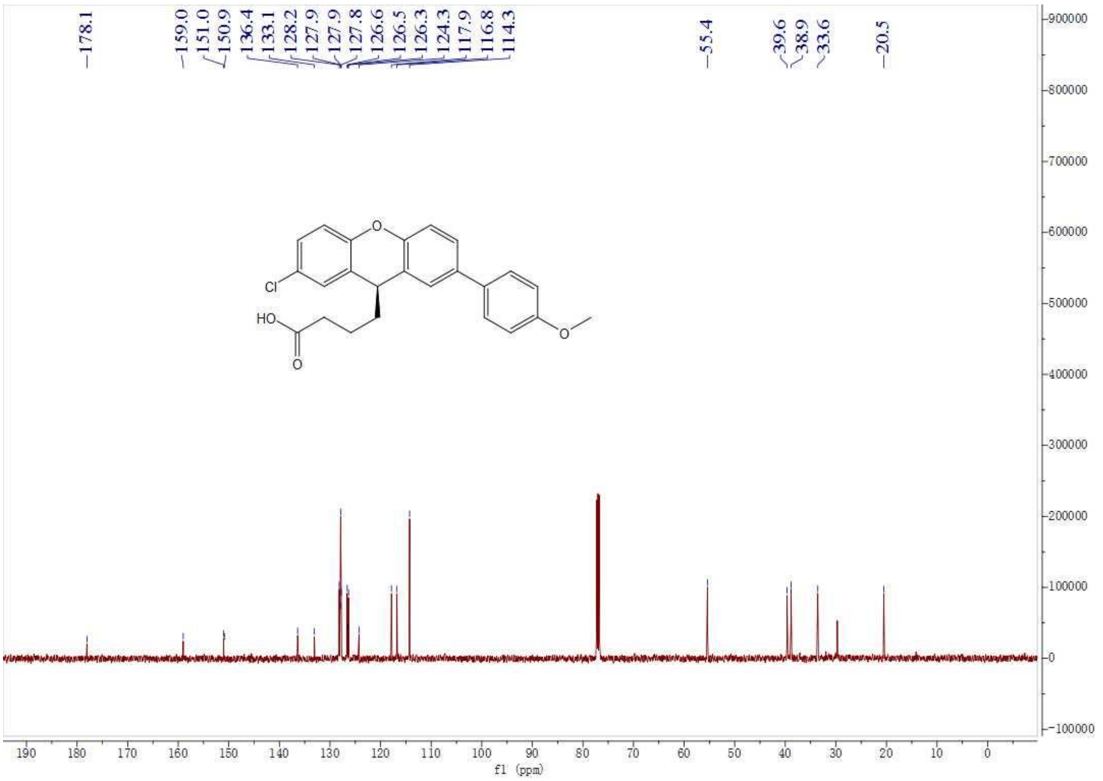

4

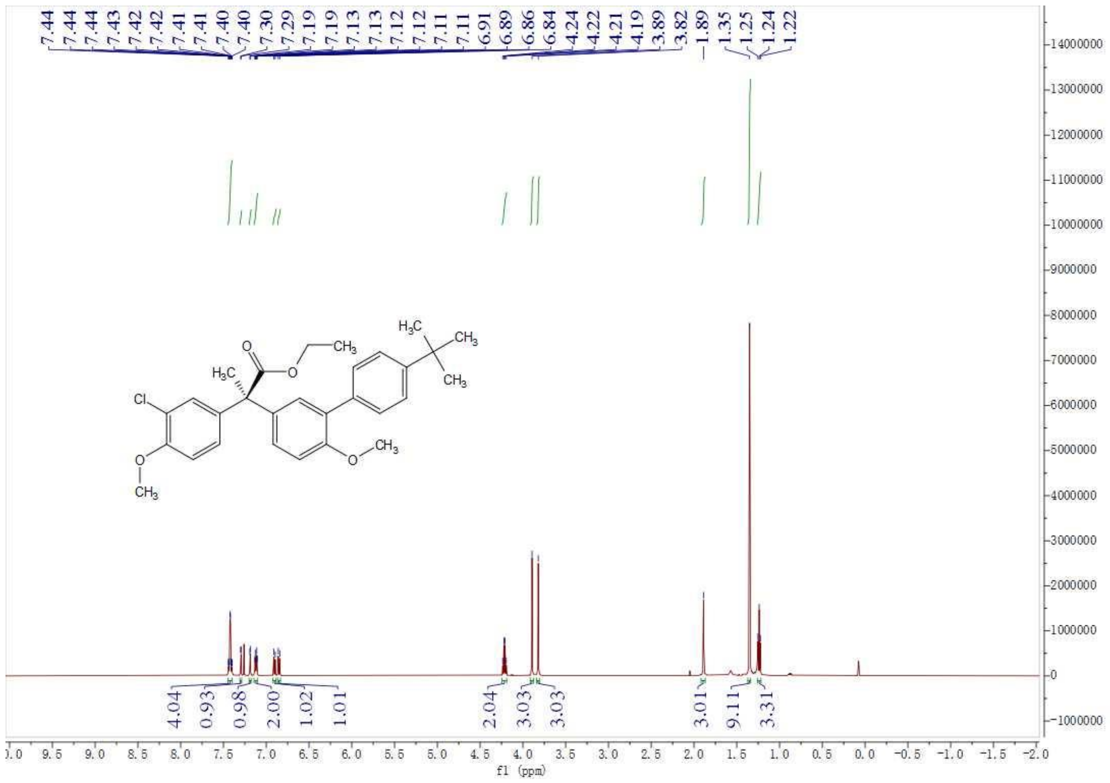
7.44
7.44
7.44
7.43
7.42
7.42
7.41
7.41
7.40
7.40
7.30
7.29
7.19
7.19
7.13
7.13
7.12
7.12
7.11
7.11
6.91
6.89
6.86
6.84
4.24
4.22
4.21
4.19
3.89
3.82
1.89
1.35
1.25
1.24
1.22
4.04
0.93
0.98
2.00
1.02
1.01
2.04
3.03
3.03
3.01
9.11
3.31
fl (ppm)

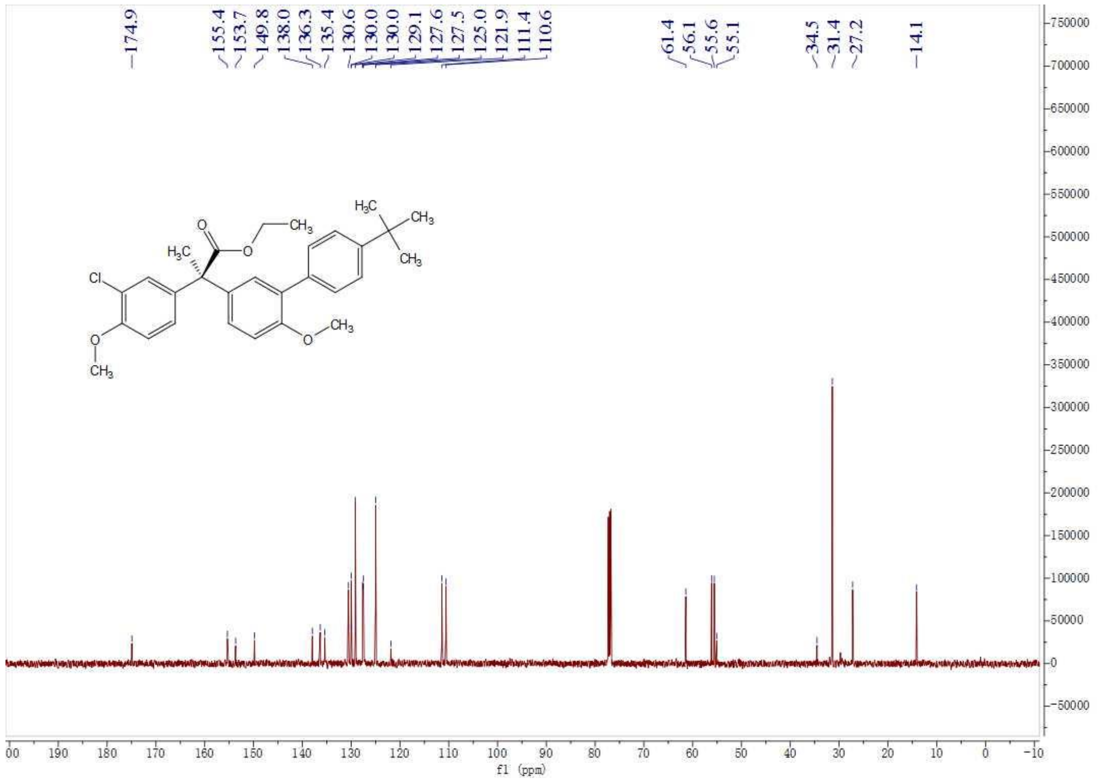
174.9
155.4
153.7
149.8
138.0
136.3
135.4
130.6
130.0
130.0
129.1
127.6
127.5
125.0
121.9
111.4
110.6
61.4
56.1
55.6
55.1
34.5
31.4
27.2
14.1
fl (ppm)

7

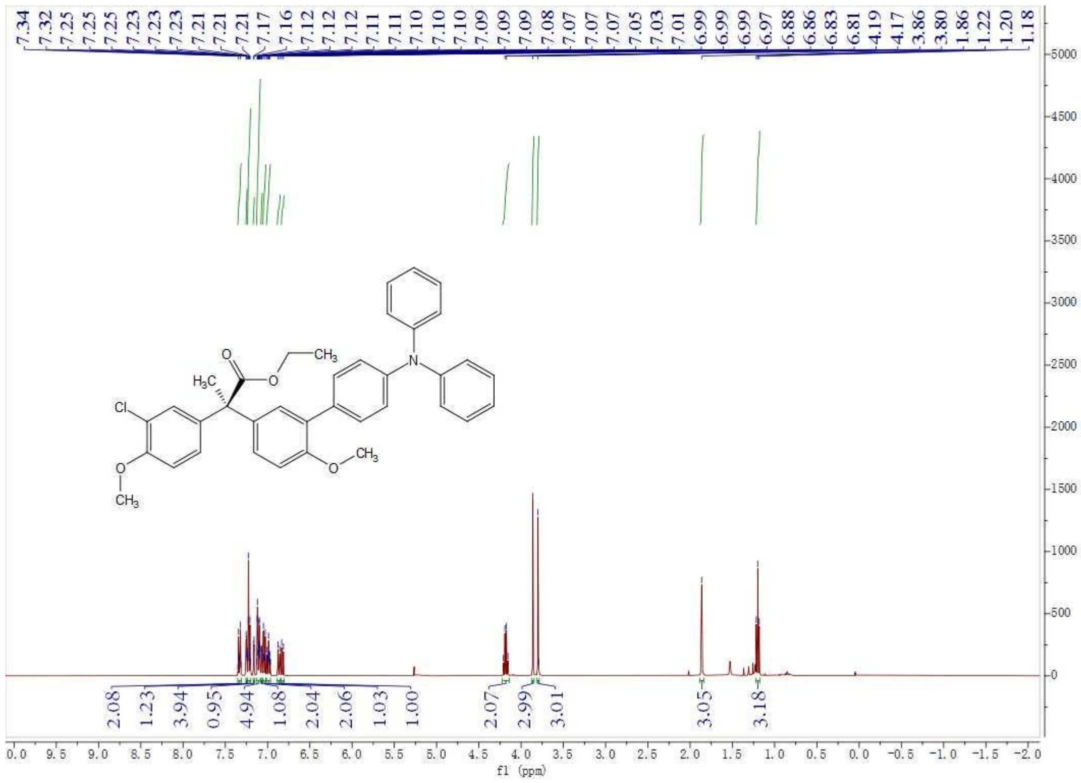

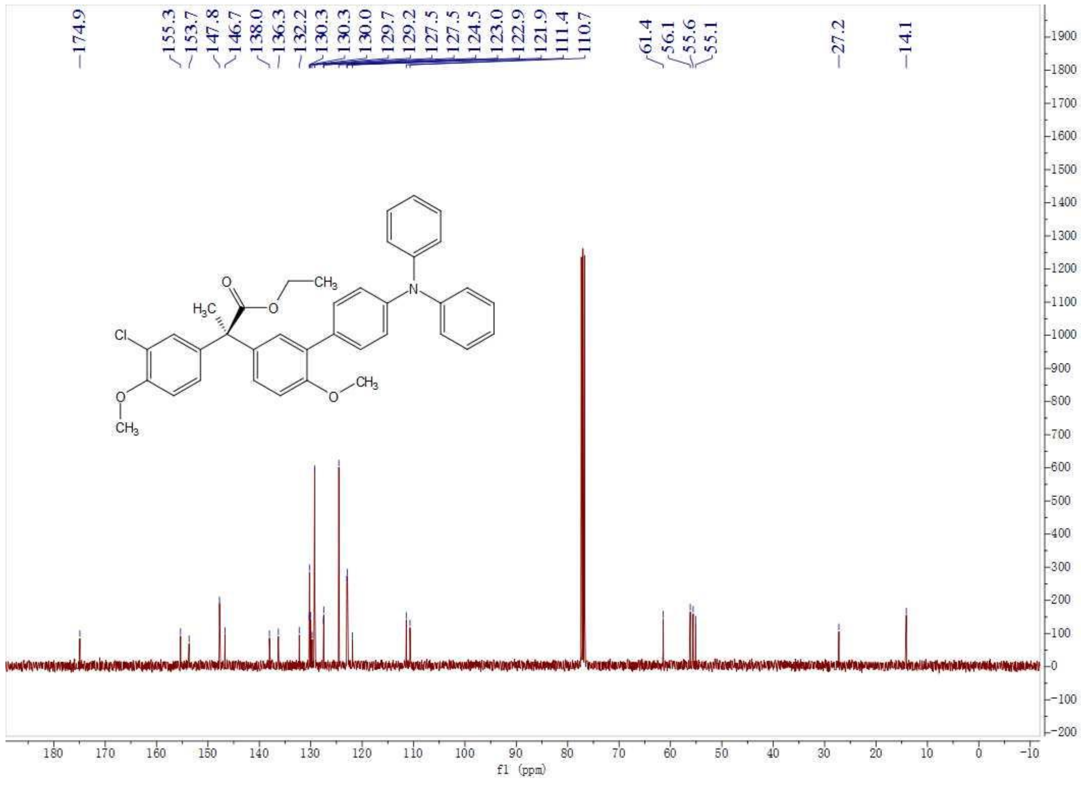

8

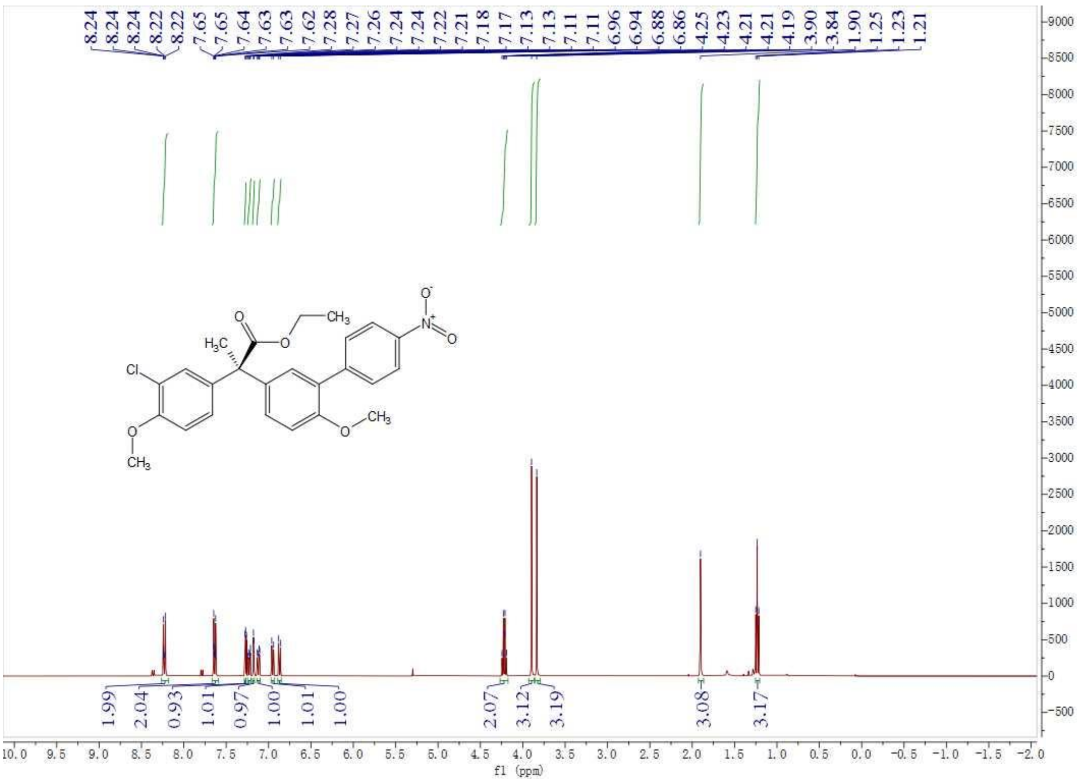

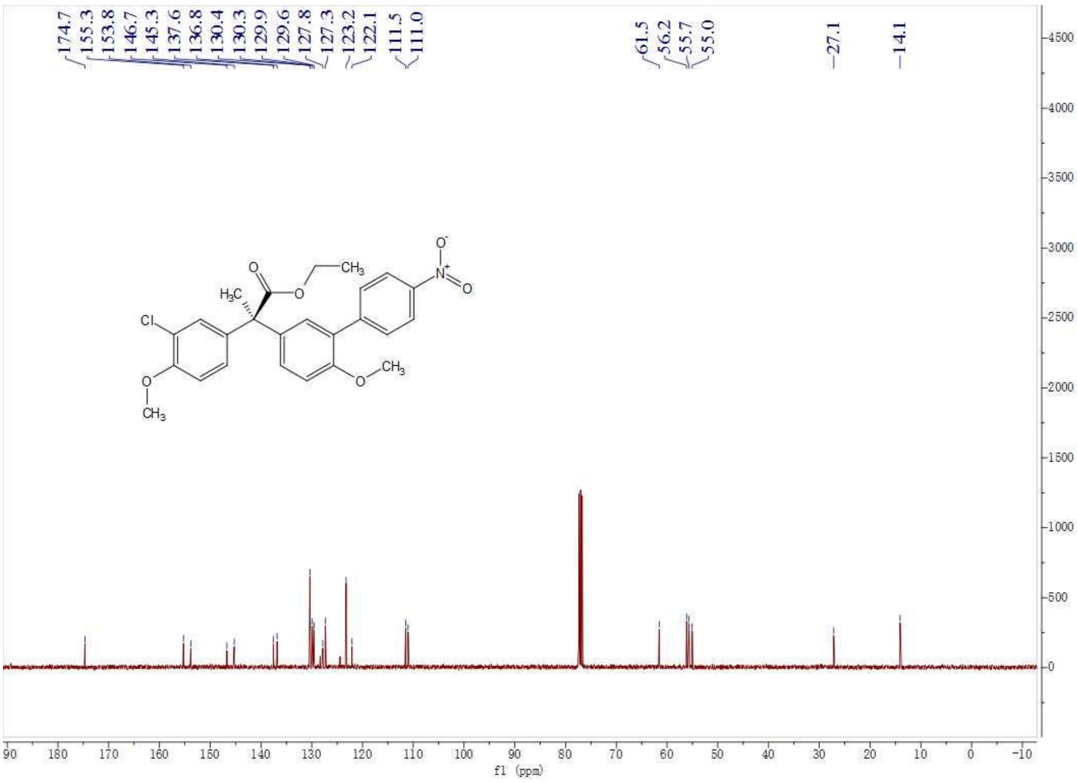

9

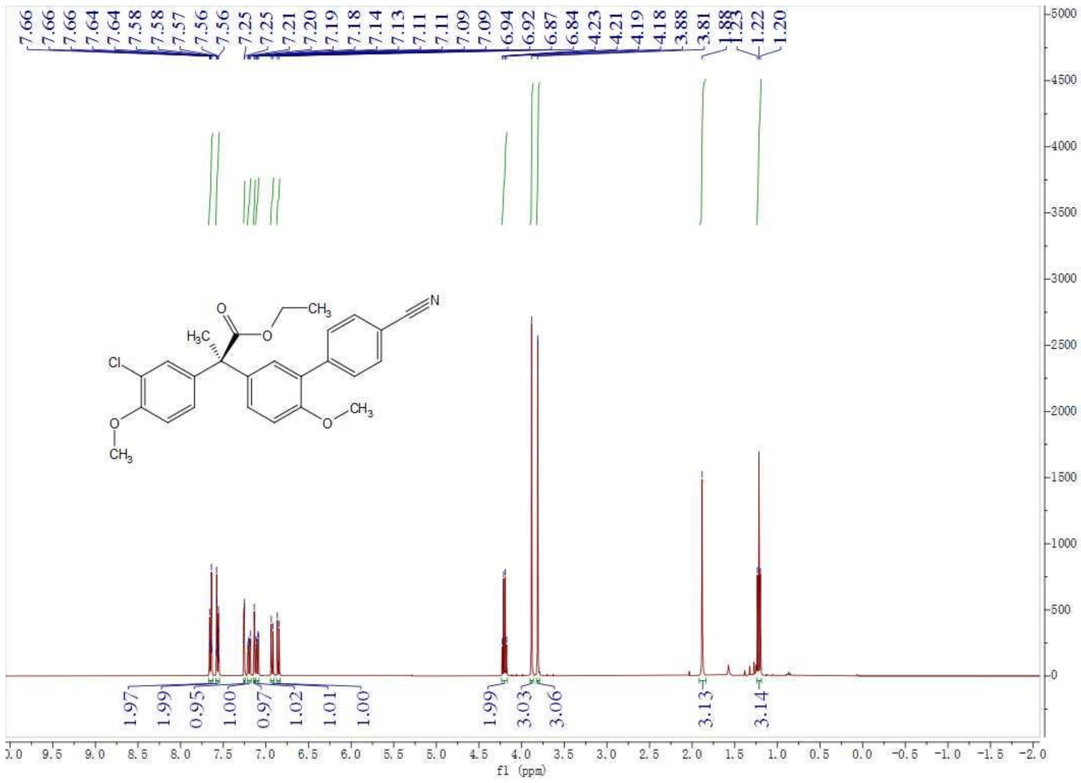

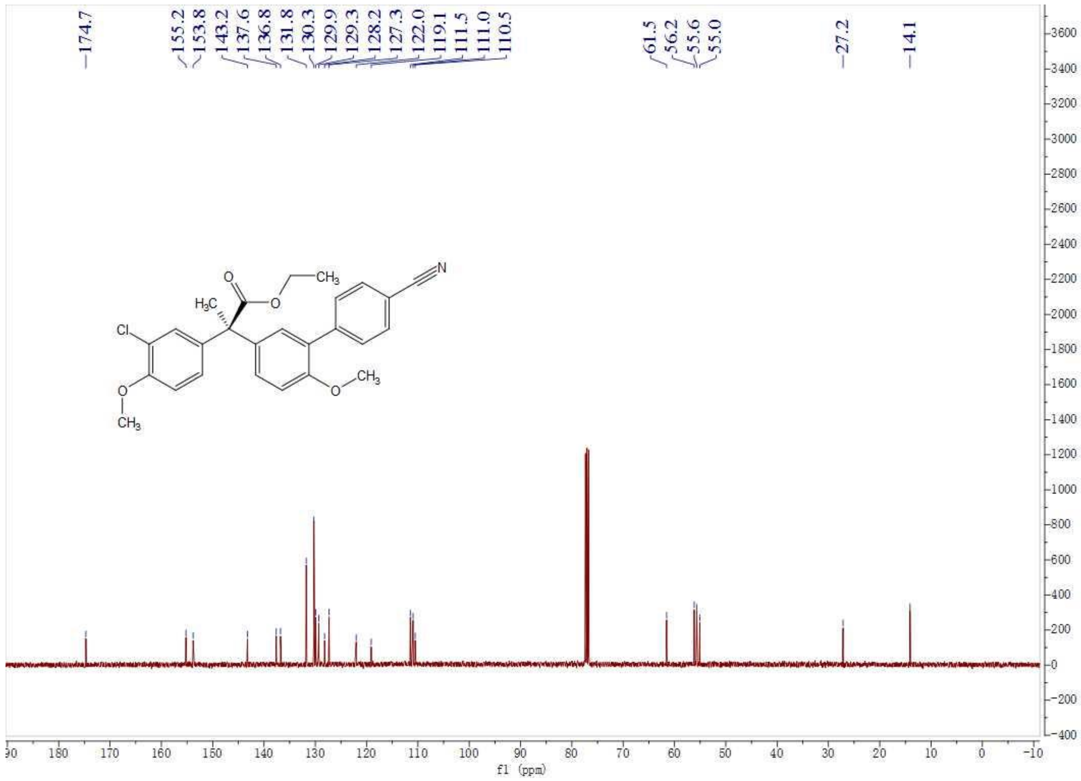

10

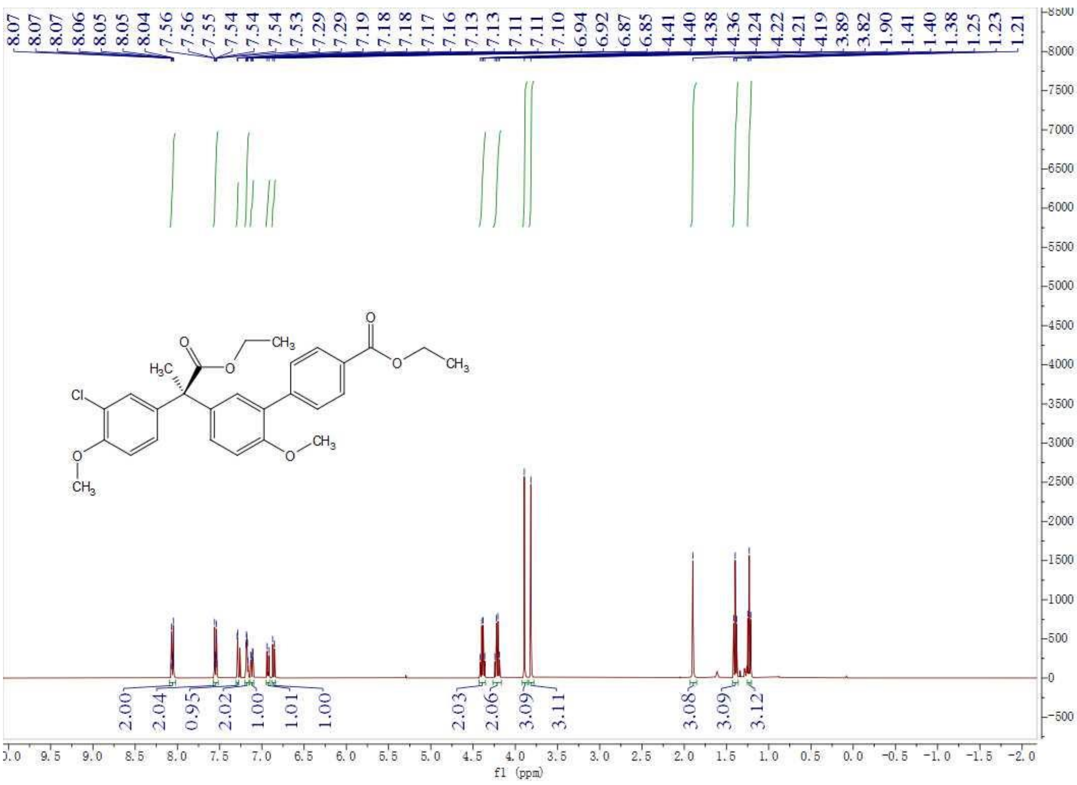

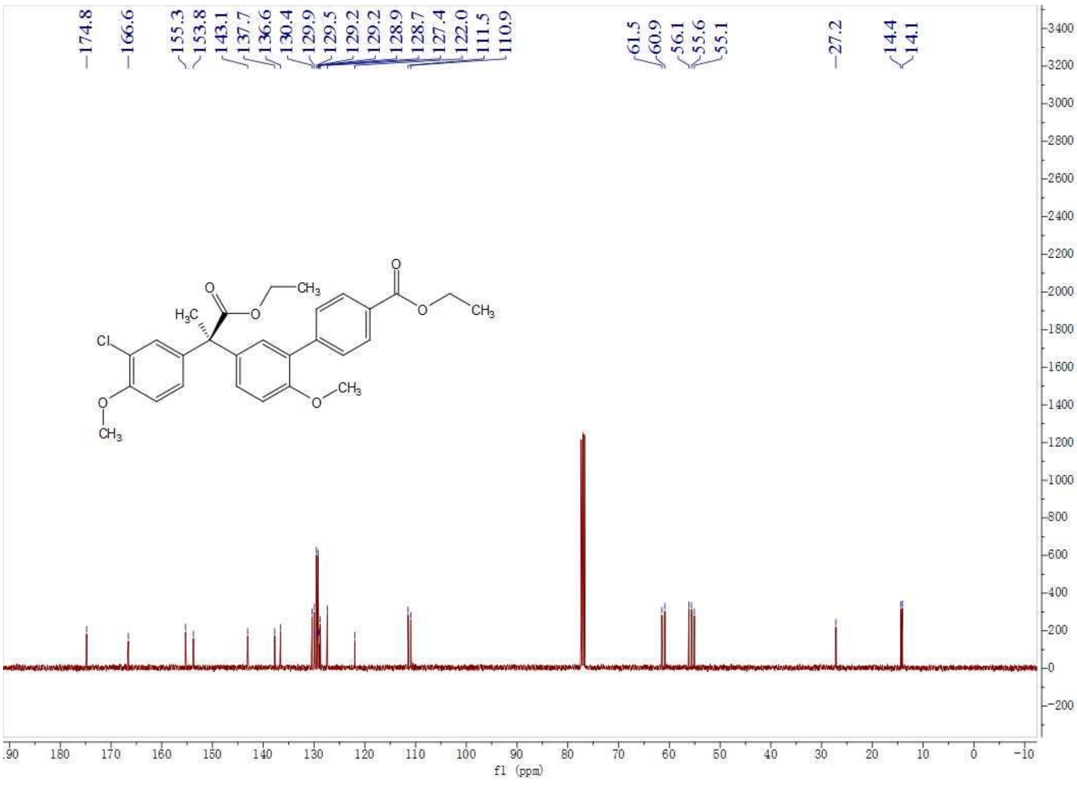

11

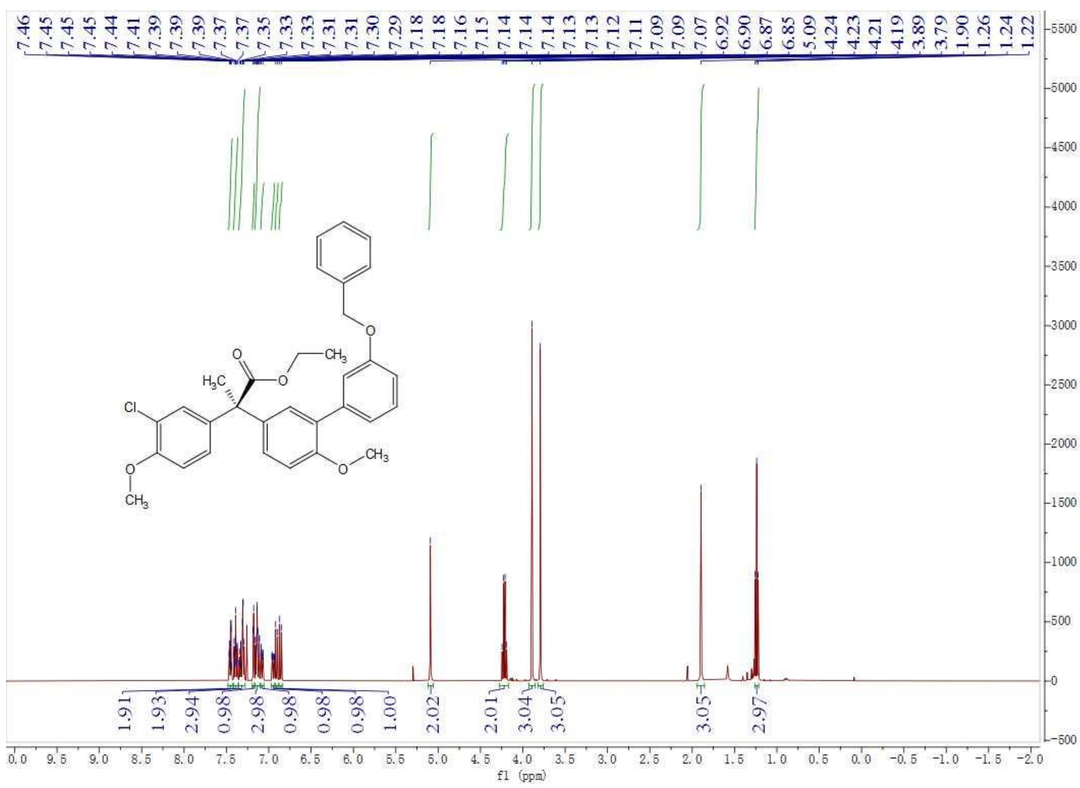

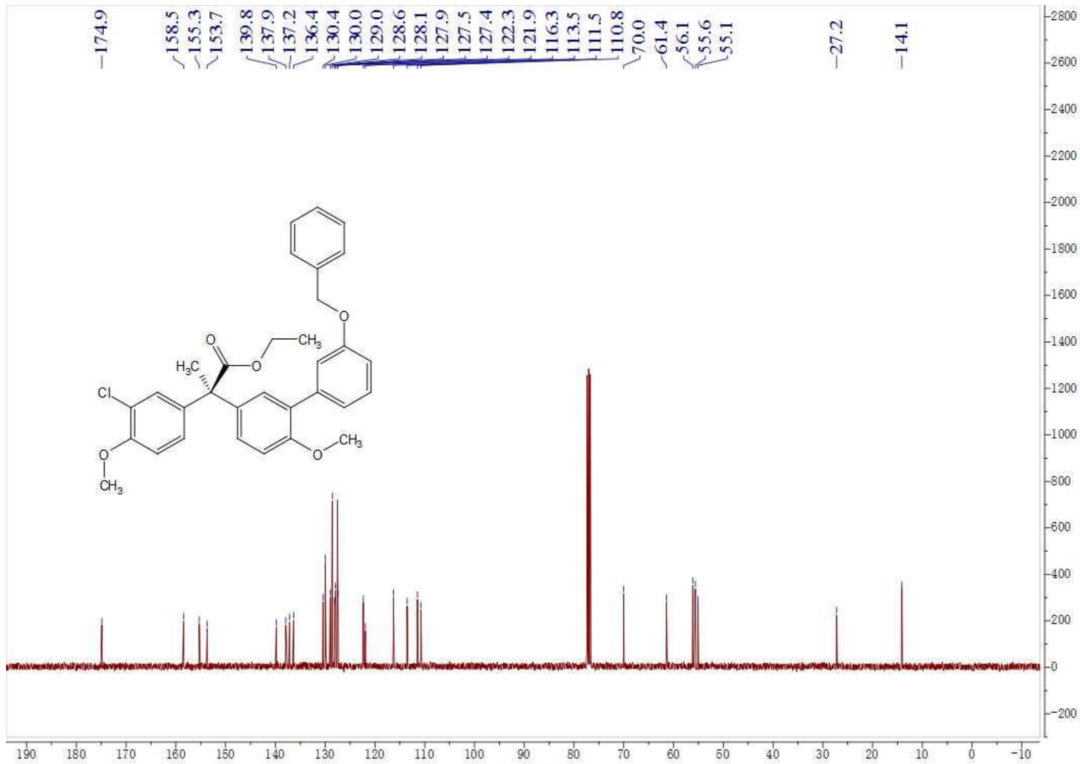

12

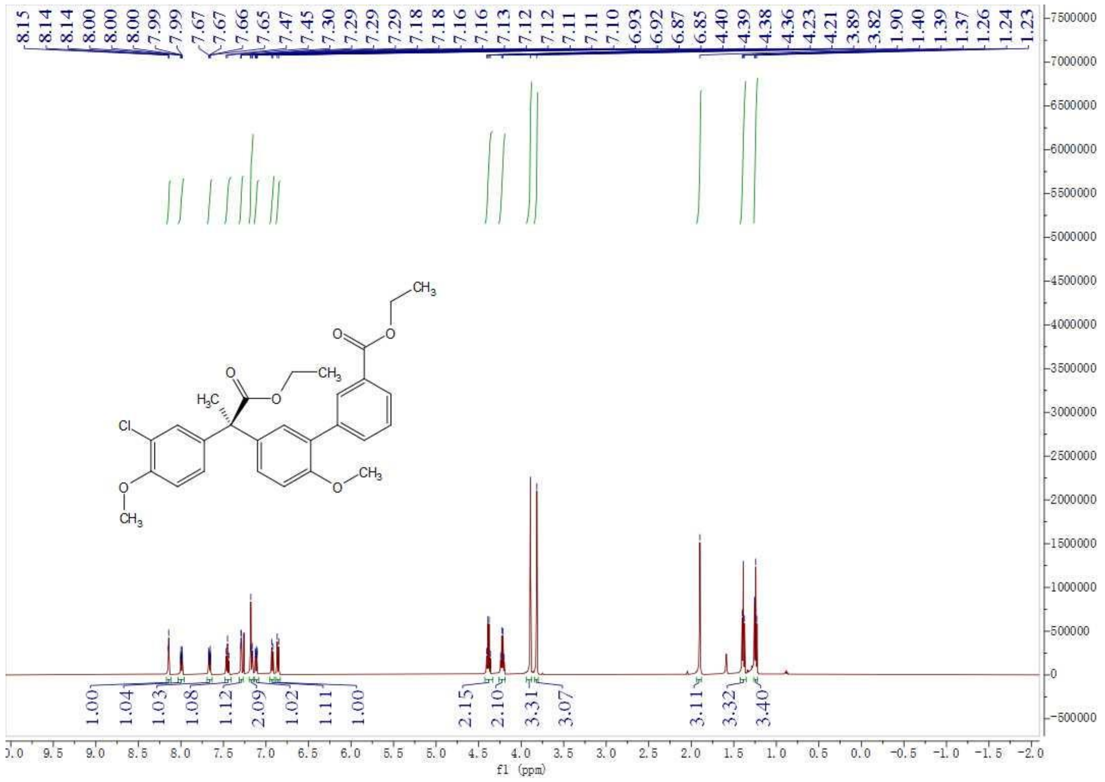

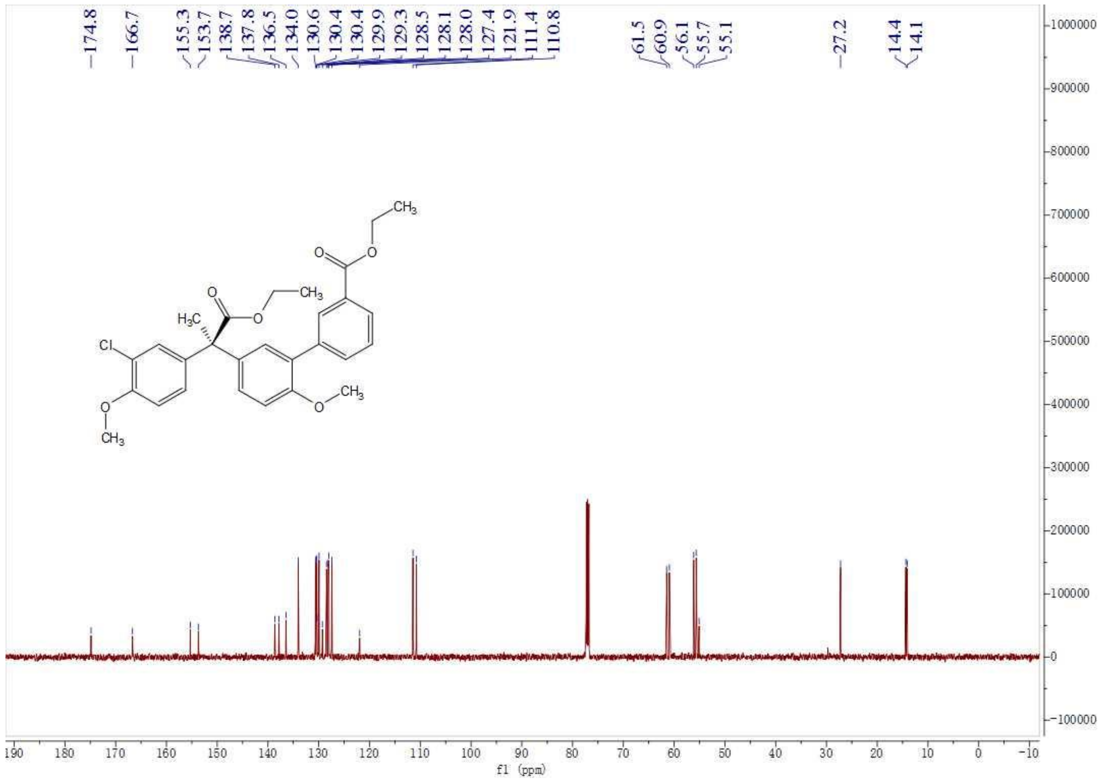

13

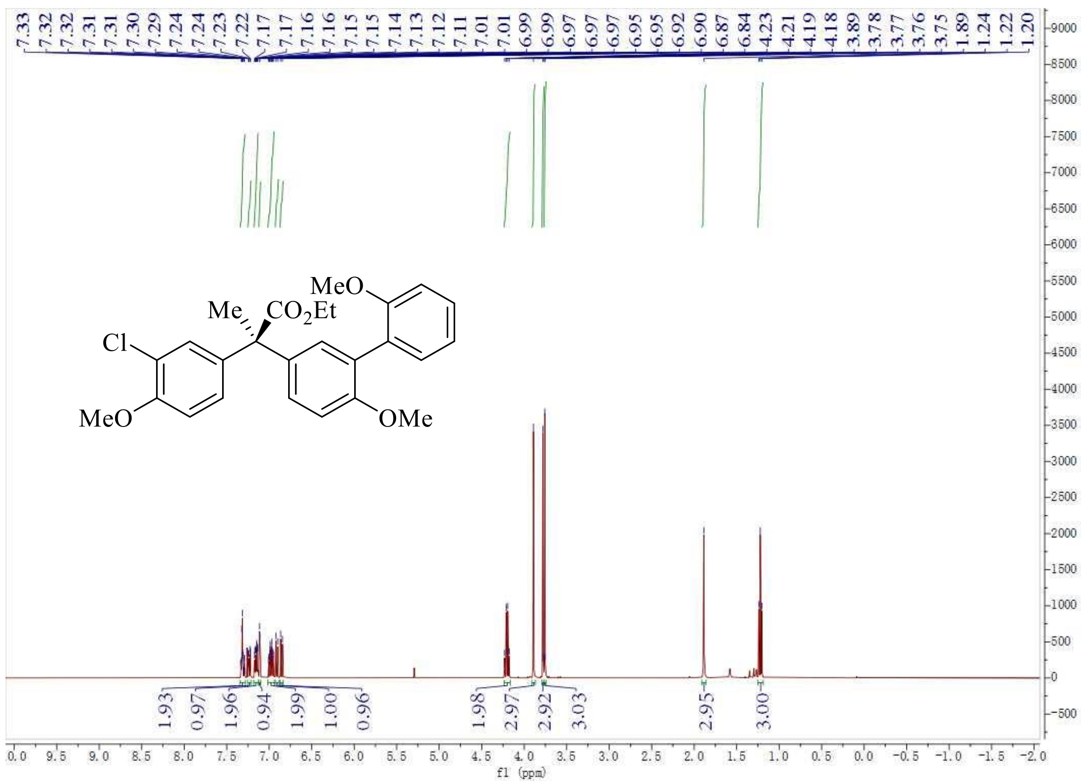

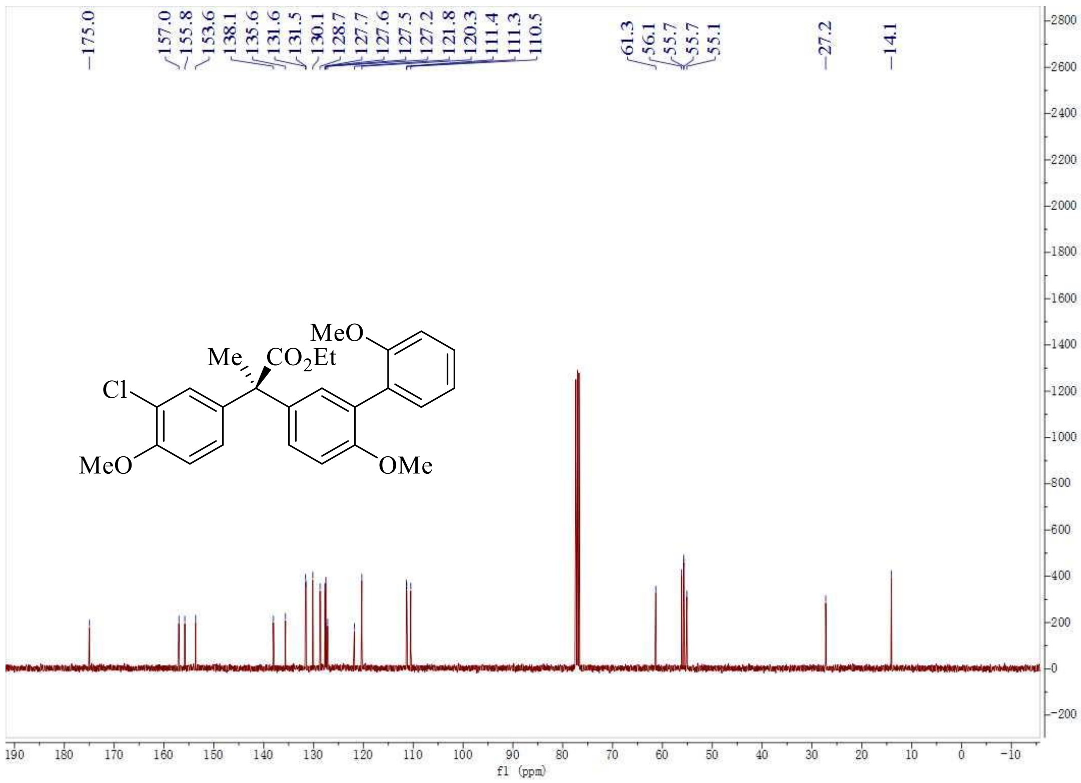

14

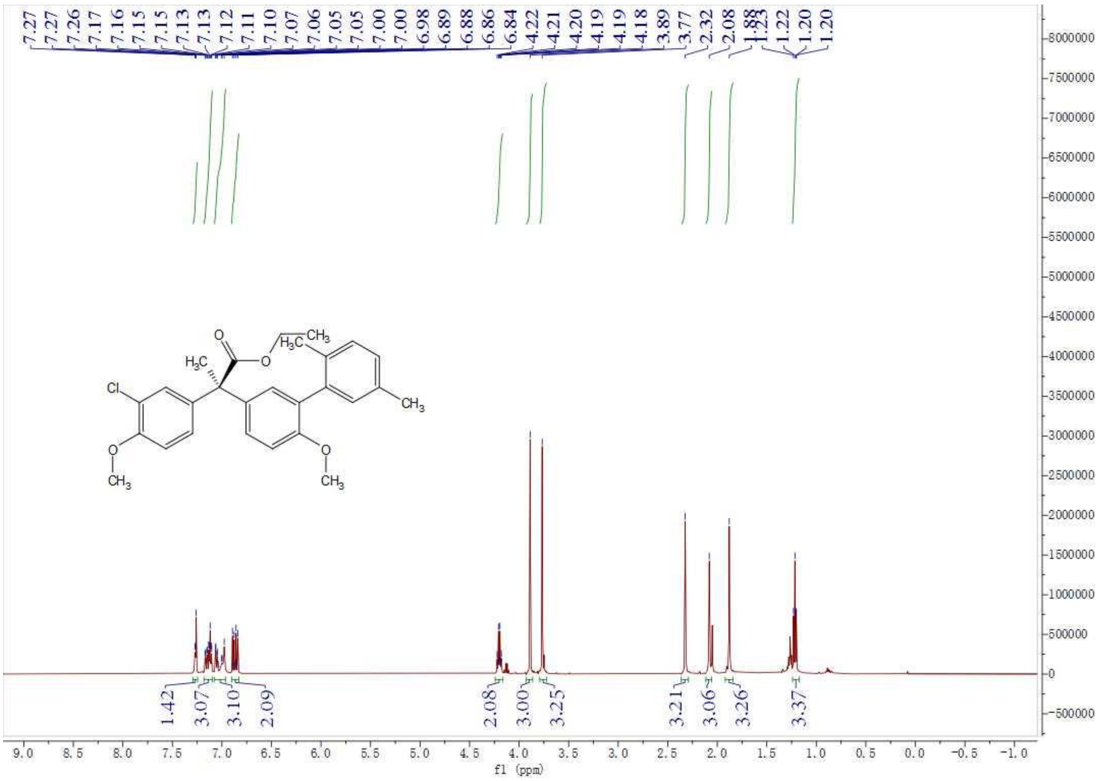
7.27 7.27 7.26 7.17 7.16 7.15 7.15 7.13 7.13 7.12 7.11 7.10 7.07 7.06 7.05 7.05 7.00 7.00 6.98 6.89 6.88 6.86 6.84 4.22 4.21 4.20 4.19 4.19 4.18 3.89 3.77 2.32 2.08 1.88 1.23 1.22 1.20 1.20
1.42 3.07 3.10 2.09 2.08 3.00 3.25 3.21 3.06 3.26 3.37
f1 (ppm)

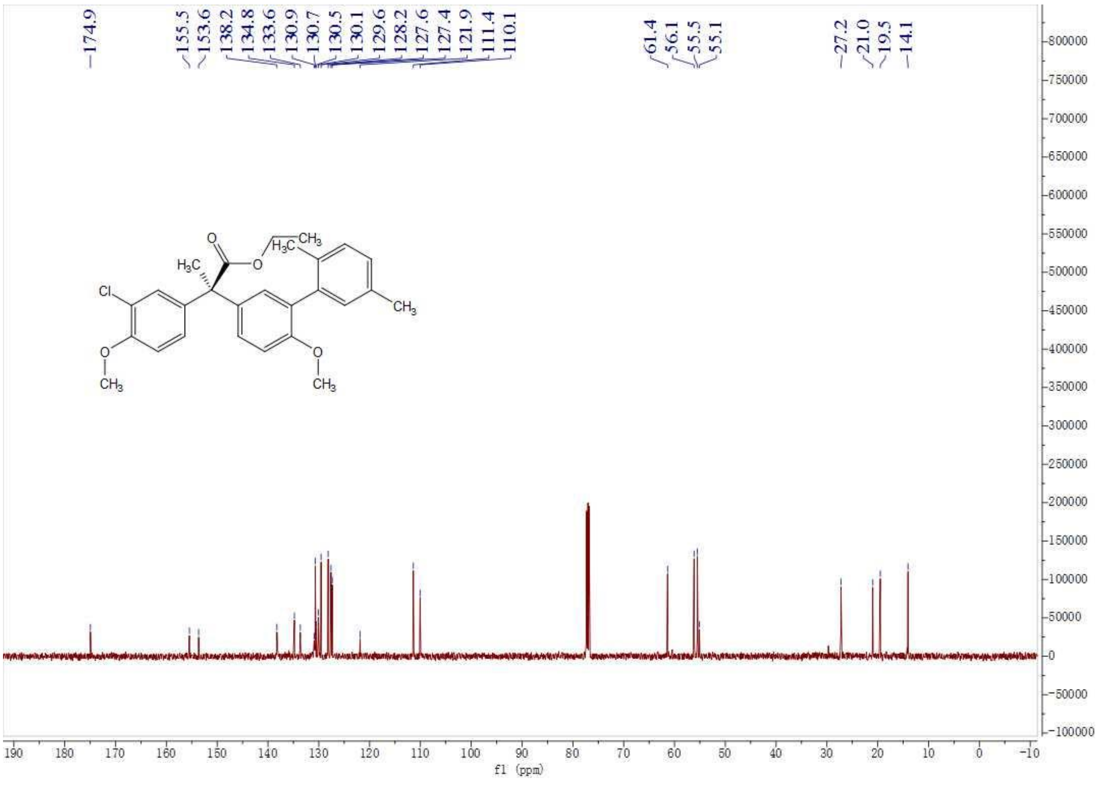
174.9 155.5 153.6 138.2 134.8 133.6 130.9 130.7 130.5 130.1 129.6 128.2 127.6 127.4 121.9 111.4 110.1 61.4 56.1 55.5 55.1 27.2 21.0 19.5 14.1
f1 (ppm)

15

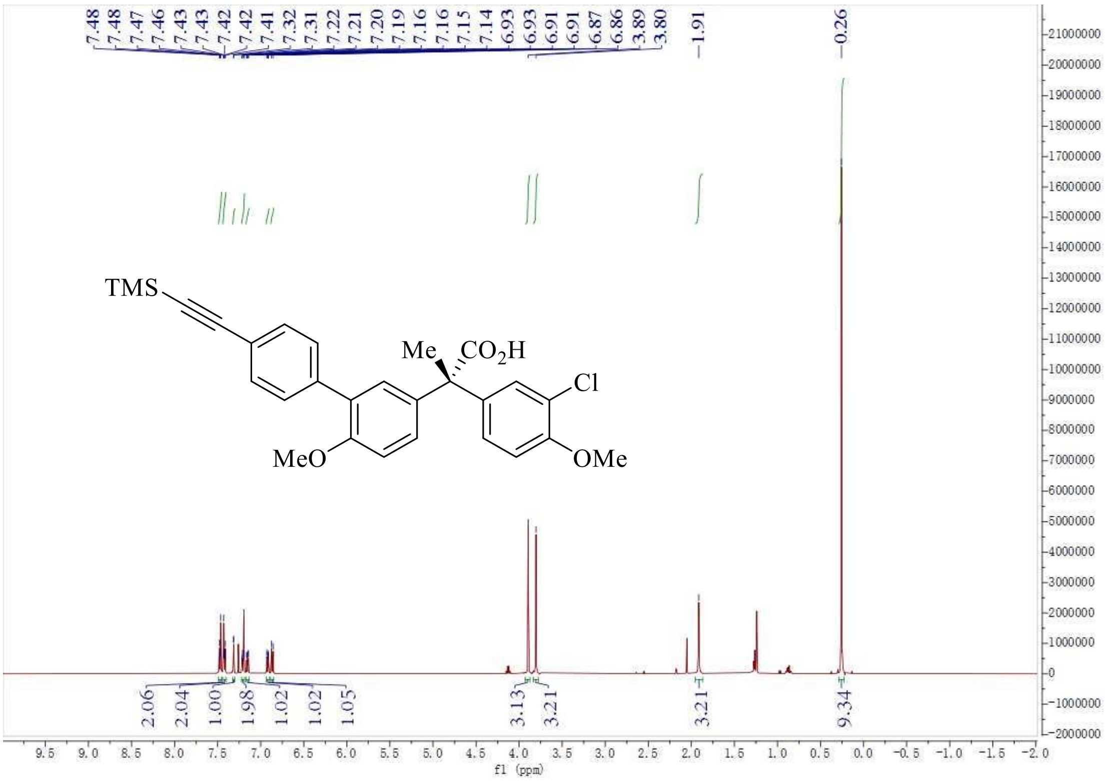

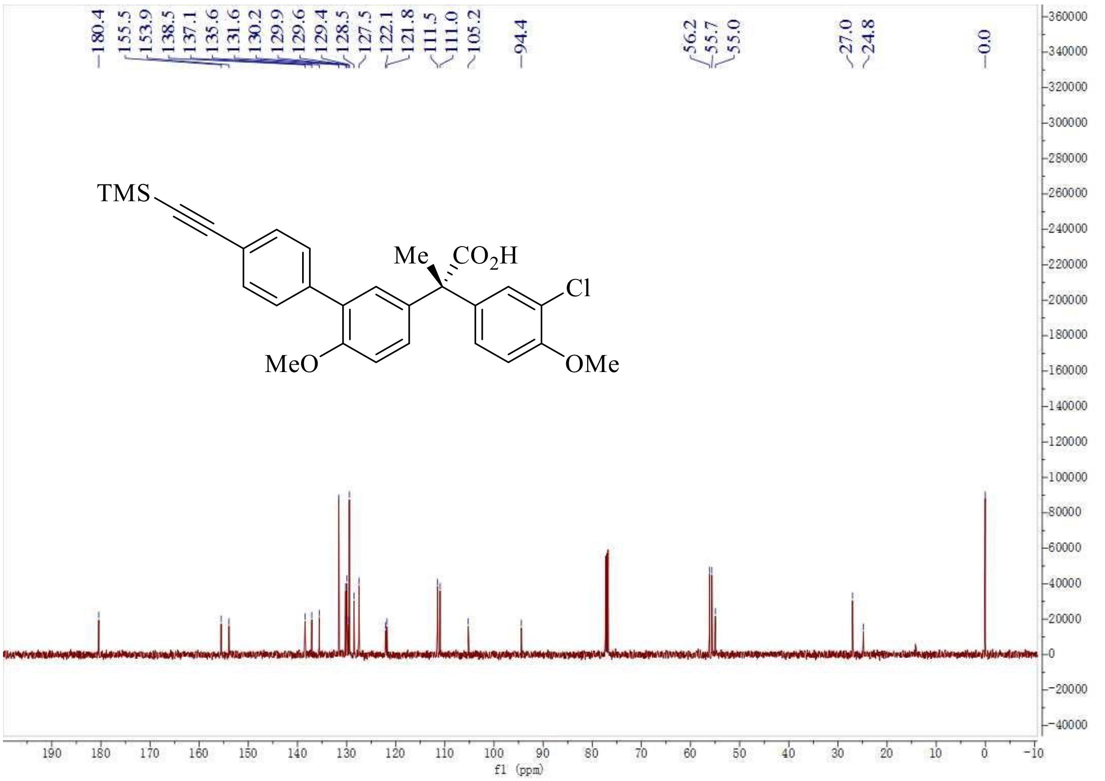

S-15

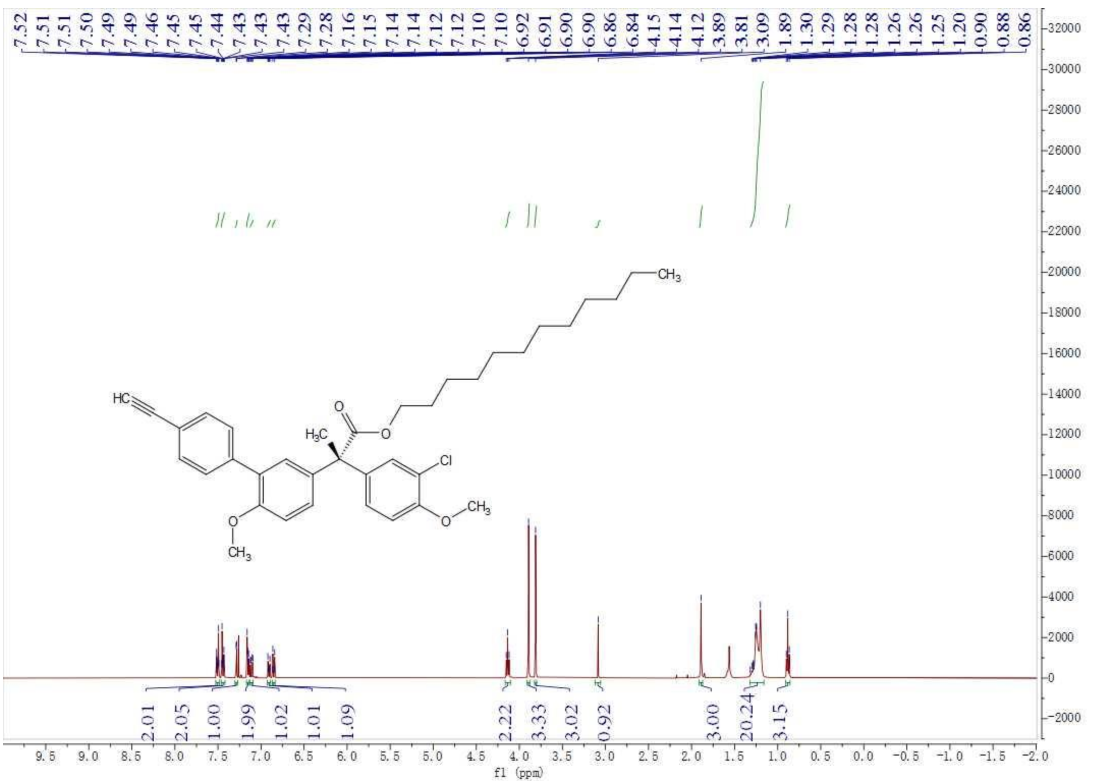

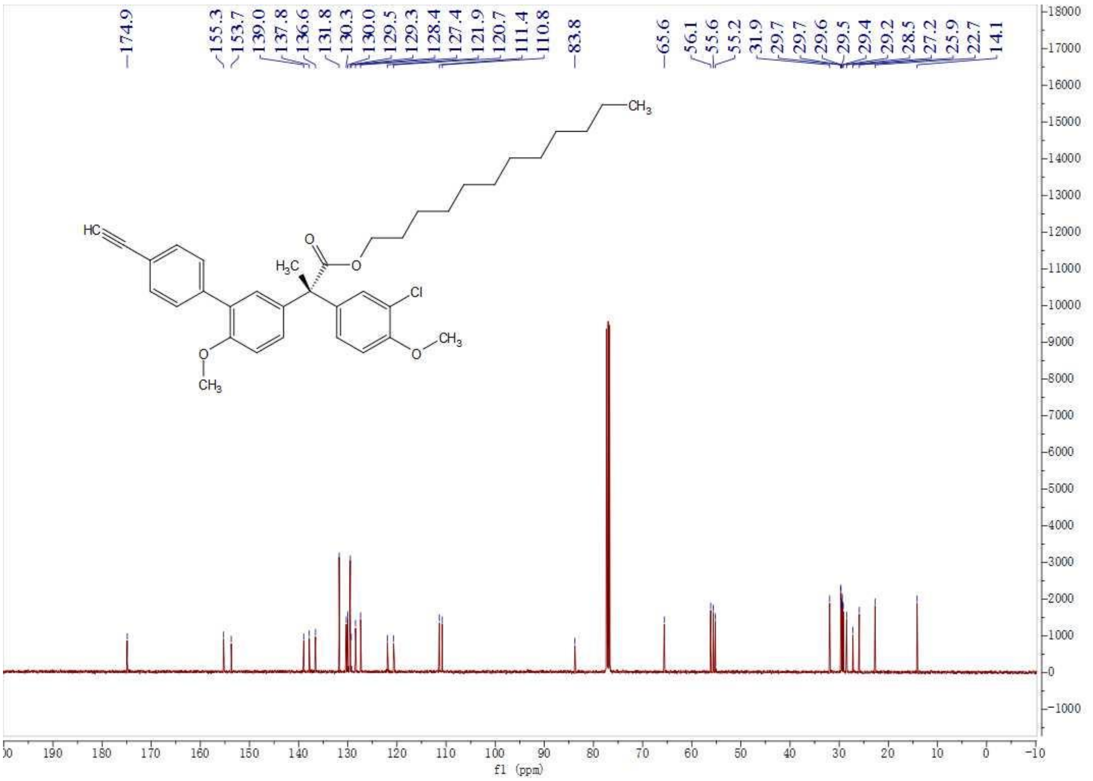

16

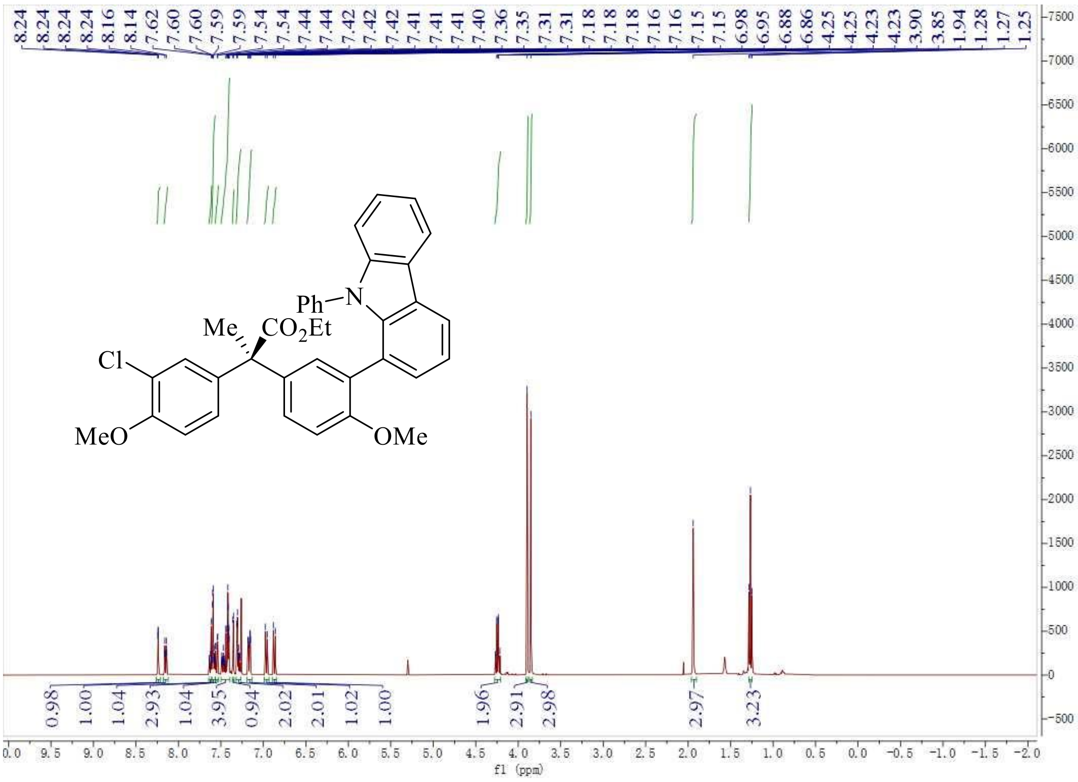

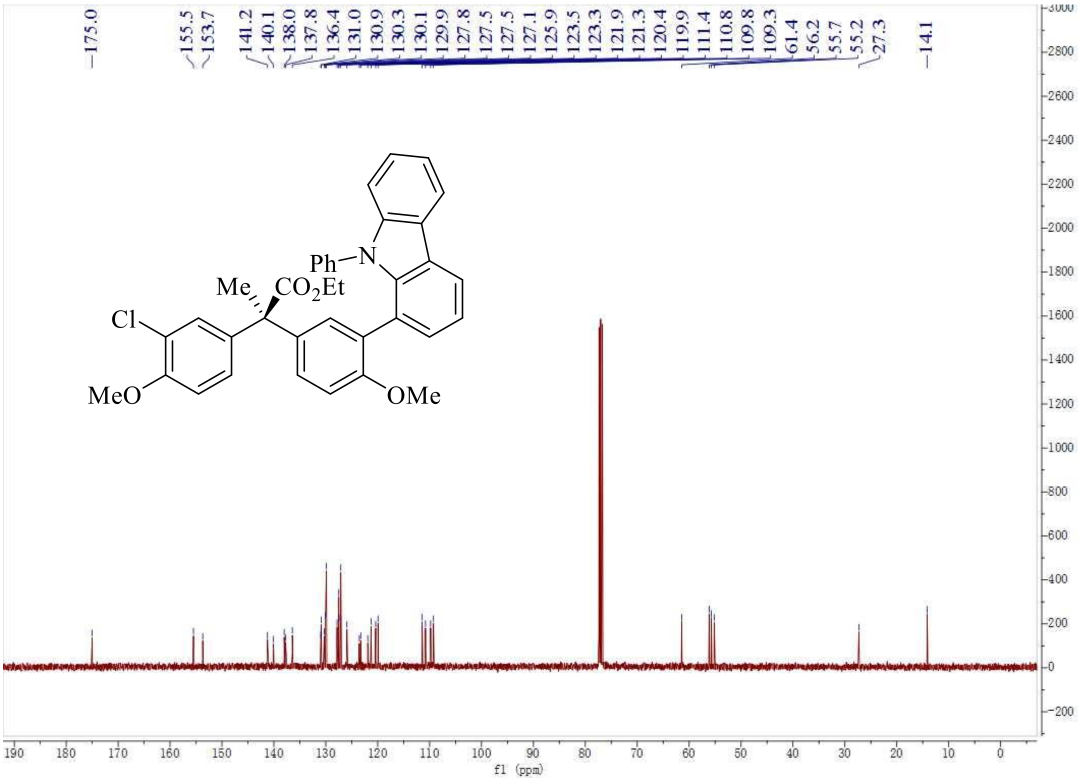

17

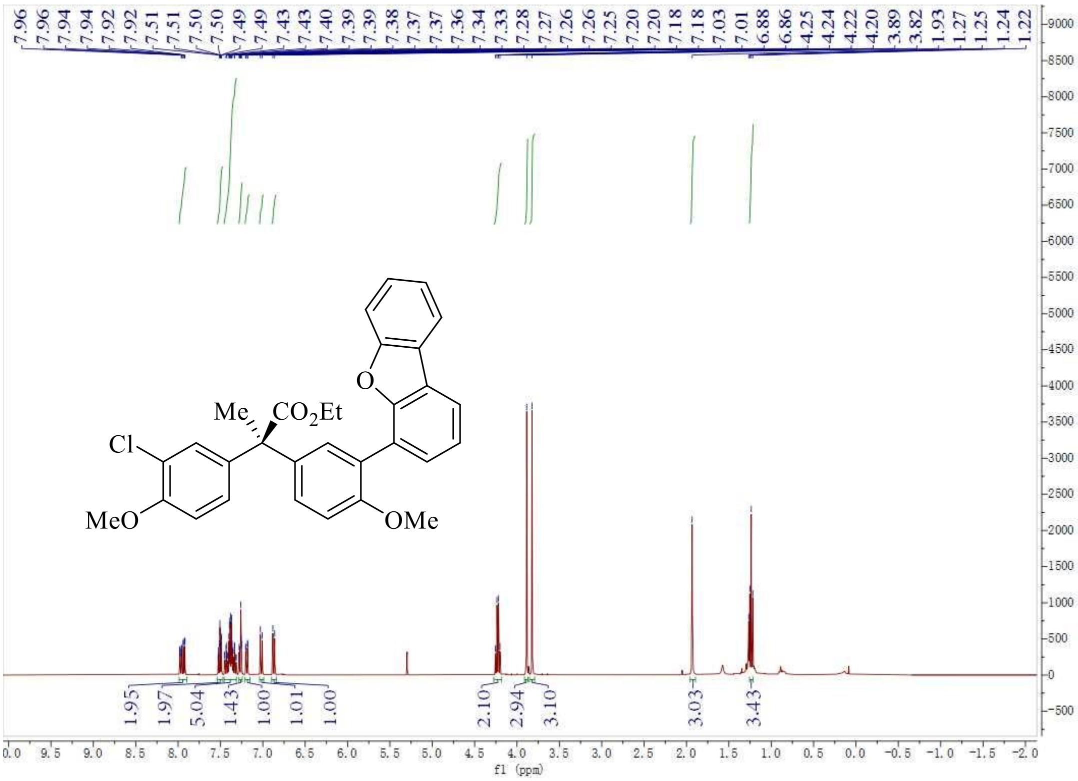
7.96
7.96
7.94
7.94
7.92
7.92
7.51
7.51
7.50
7.50
7.49
7.49
7.43
7.43
7.40
7.39
7.39
7.38
7.37
7.37
7.36
7.34
7.33
7.28
7.27
7.26
7.26
7.25
7.20
7.20
7.18
7.18
7.03
7.01
6.88
6.86
4.25
4.24
4.22
4.20
3.89
3.82
1.93
1.27
1.25
1.24
1.22
Me
CO_2Et
Cl
MeO
O
OMe
1.95
1.97
5.04
1.43
1.00
1.01
1.00
2.10
2.94
3.10
3.03
3.43
f1 (ppm)

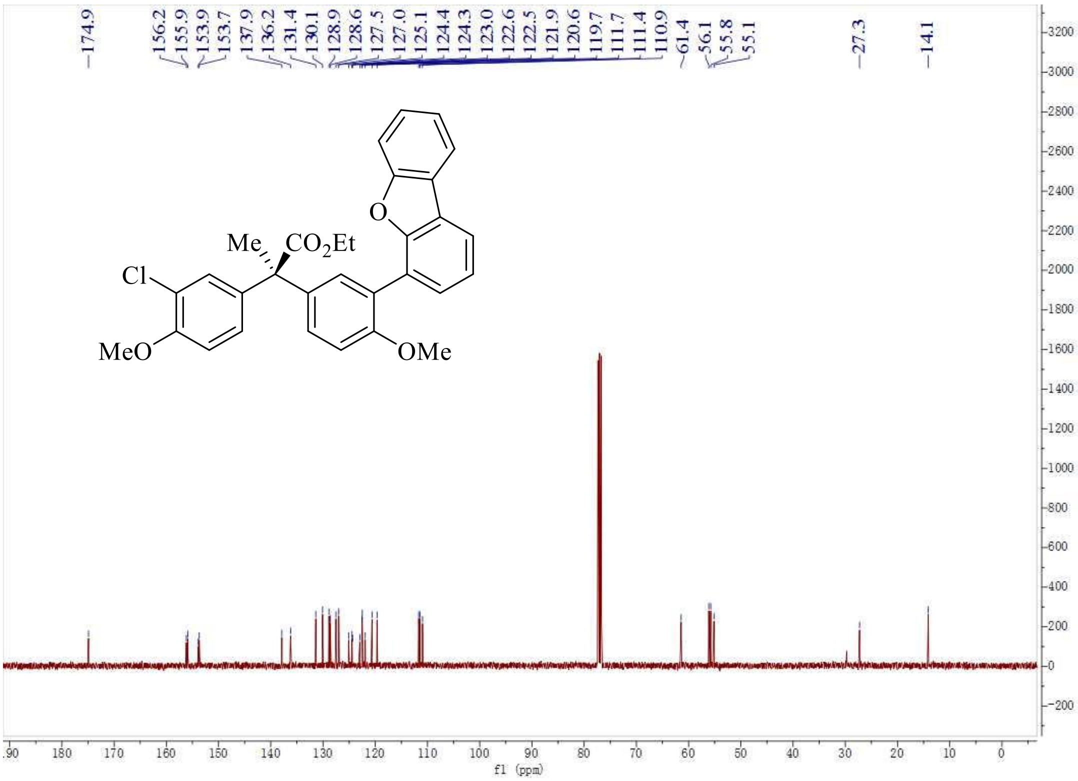
174.9
156.2
155.9
153.9
153.7
137.9
136.2
131.4
130.1
128.9
128.6
127.5
127.0
125.1
124.4
124.3
123.0
122.6
122.5
121.9
120.6
119.7
111.7
111.4
110.9
61.4
56.1
55.8
55.1
27.3
14.1
Me
CO_2Et
Cl
MeO
O
OMe
f1 (ppm)

18

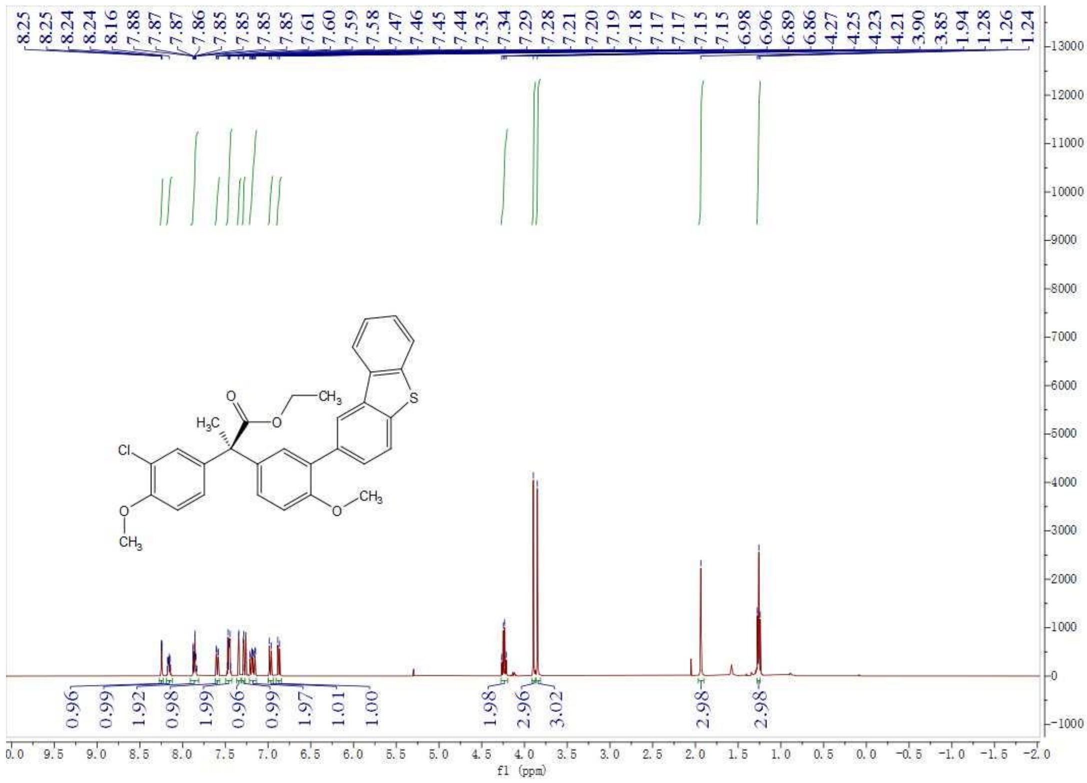

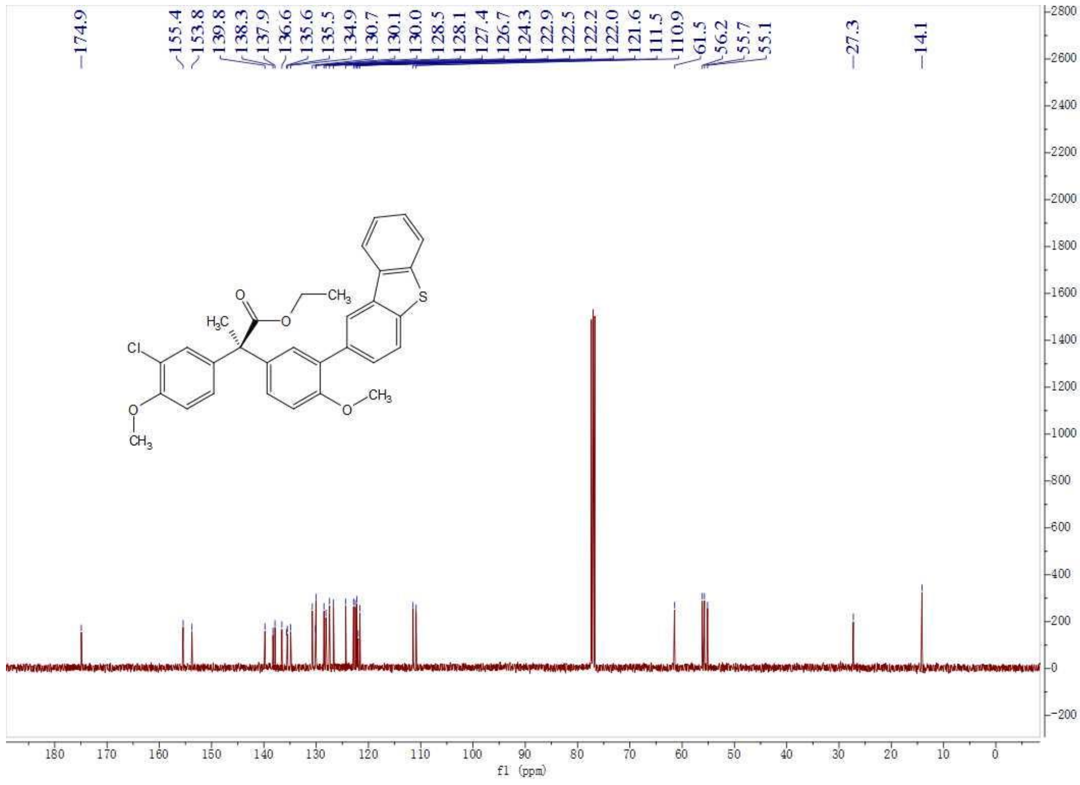

19

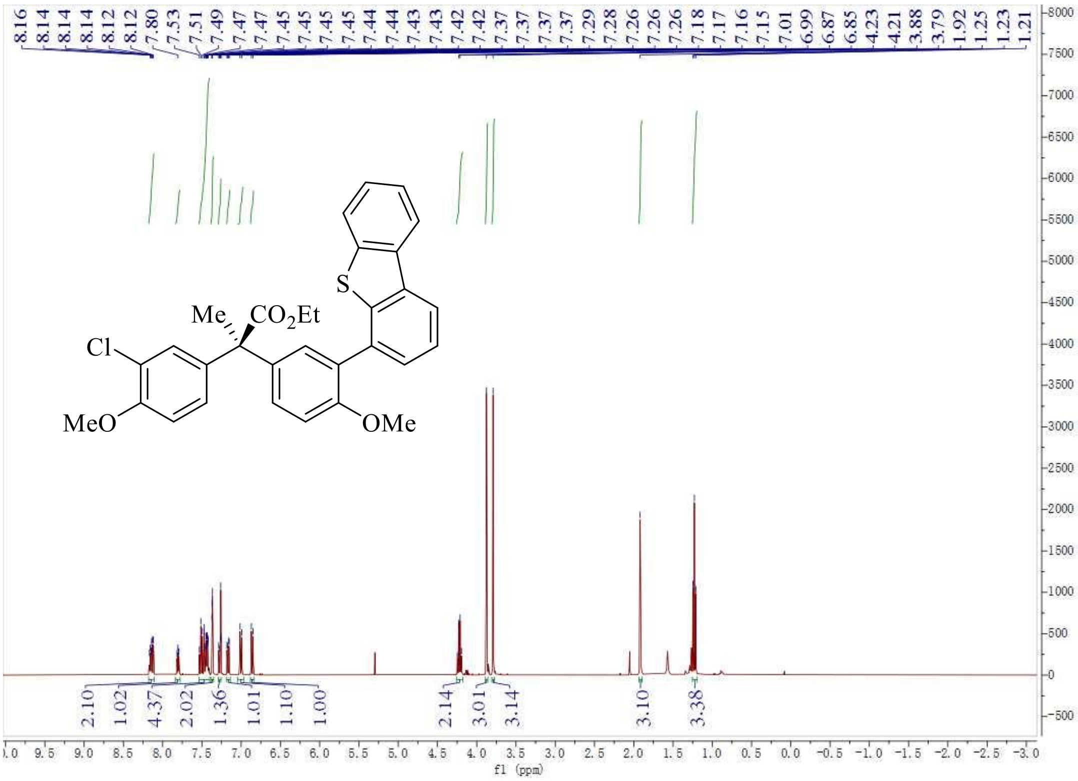
8.16
8.14
8.14
8.14
8.12
8.12
7.80
7.53
7.51
7.49
7.47
7.47
7.45
7.45
7.45
7.44
7.44
7.43
7.43
7.42
7.42
7.37
7.37
7.37
7.37
7.29
7.28
7.26
7.26
7.18
7.17
7.16
7.15
7.01
6.99
6.87
6.85
4.23
4.21
3.88
3.79
1.92
1.25
1.23
1.21
Me
CO2Et
Cl
MeO
S
OMe
2.10
1.02
4.37
2.02
1.36
1.01
1.10
1.00
2.14
3.01
3.14
3.10
3.38
f1 (ppm)

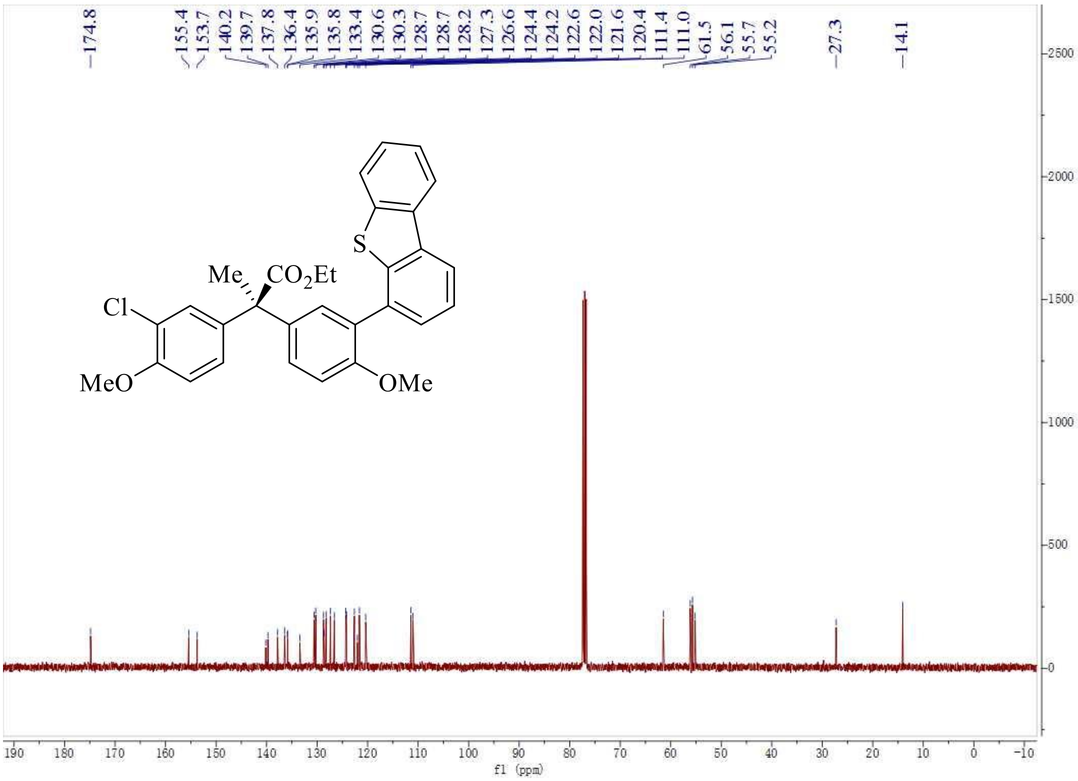
174.8
155.4
153.7
140.2
139.7
137.8
136.4
135.9
135.8
133.4
130.6
130.3
128.7
128.7
128.2
127.3
126.6
124.4
124.2
122.6
122.0
121.6
120.4
111.4
111.0
61.5
56.1
55.7
55.2
27.3
14.1
Me
CO2Et
Cl
MeO
S
OMe
f1 (ppm)

20

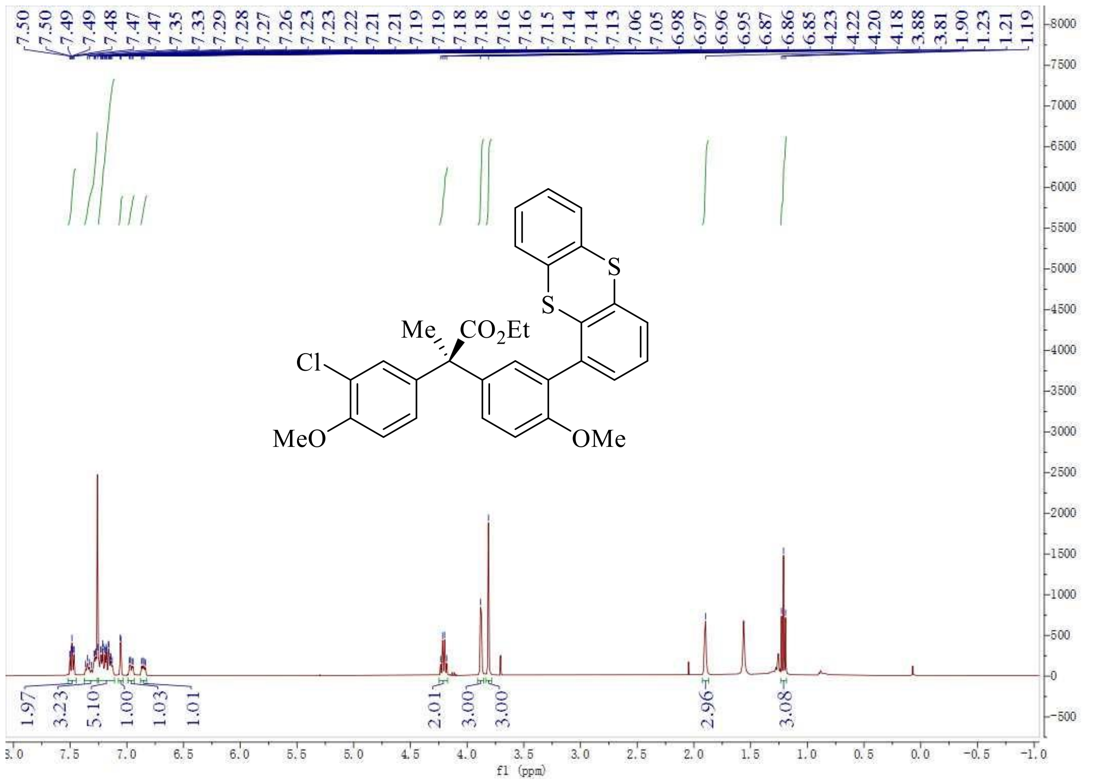

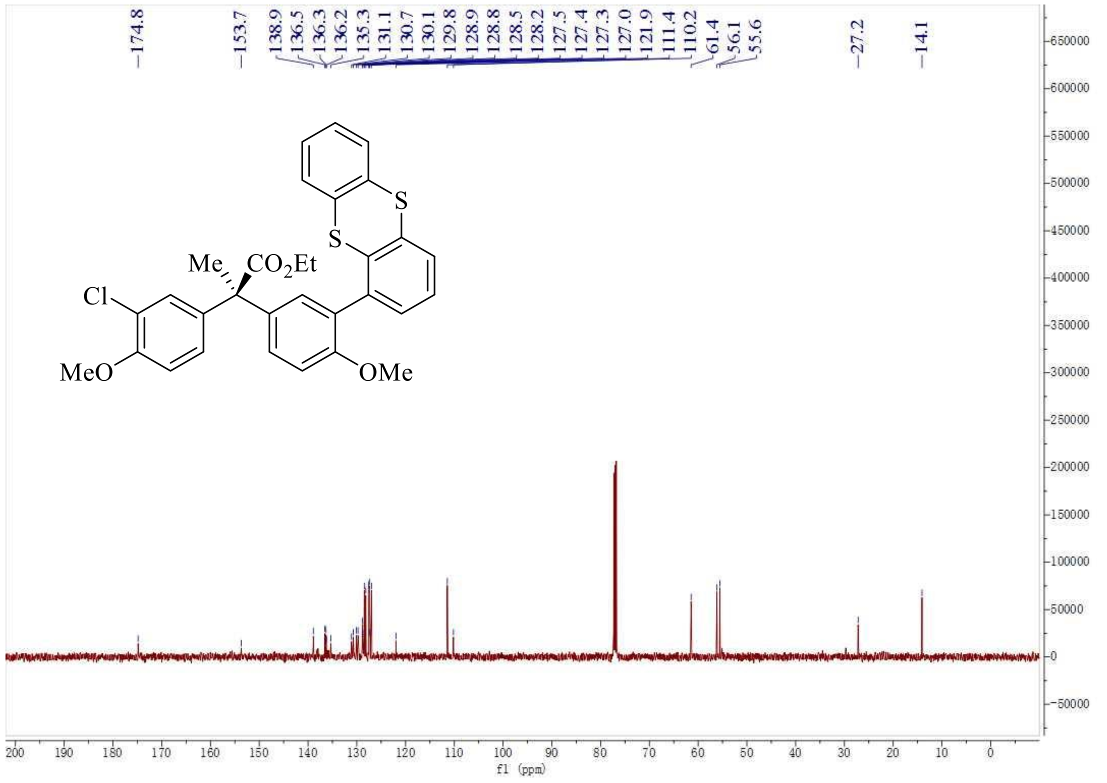

21

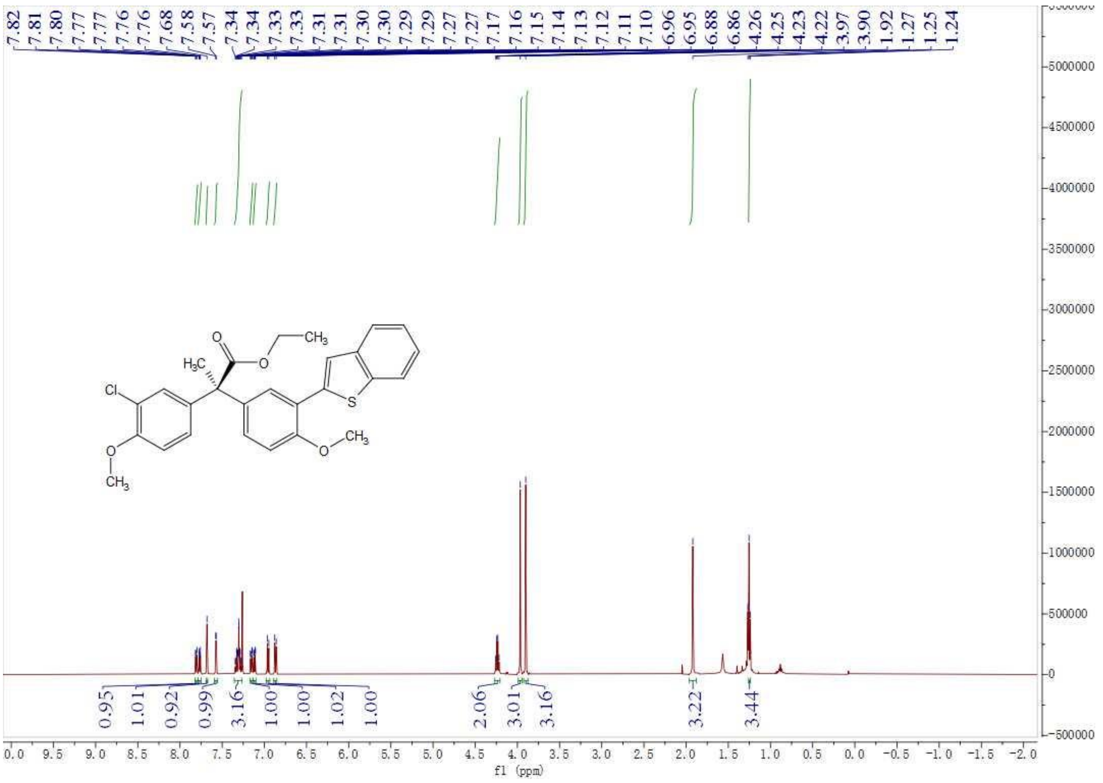

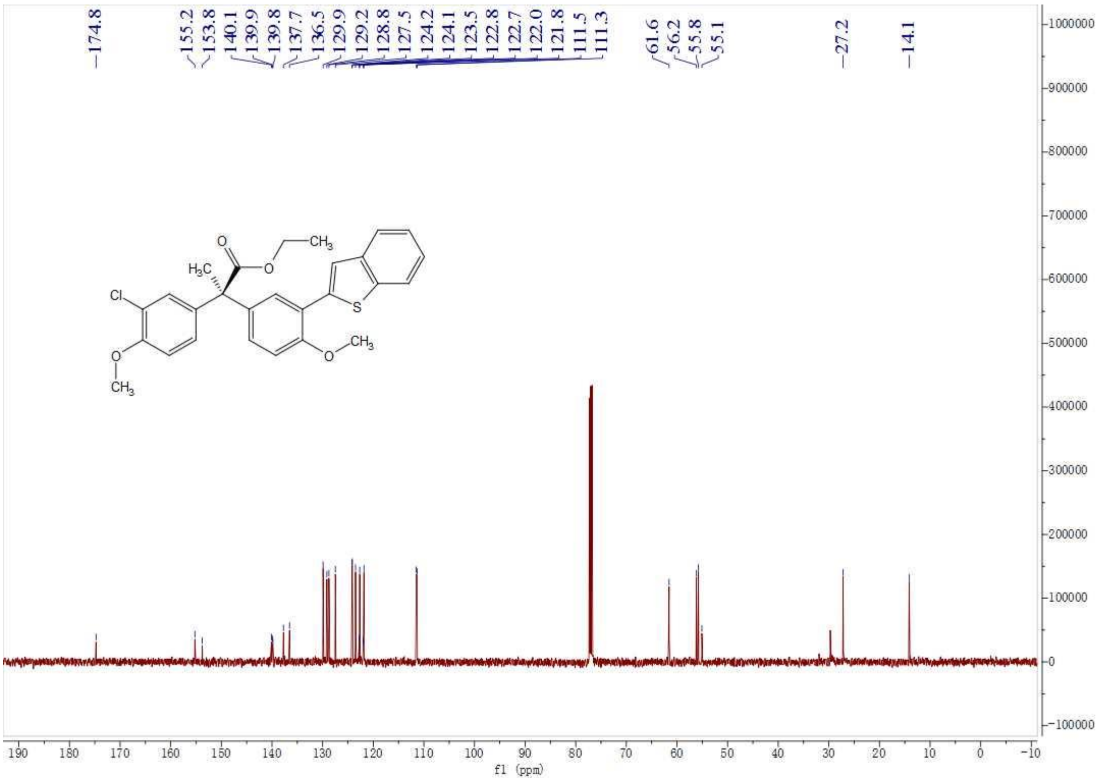

Bis-21

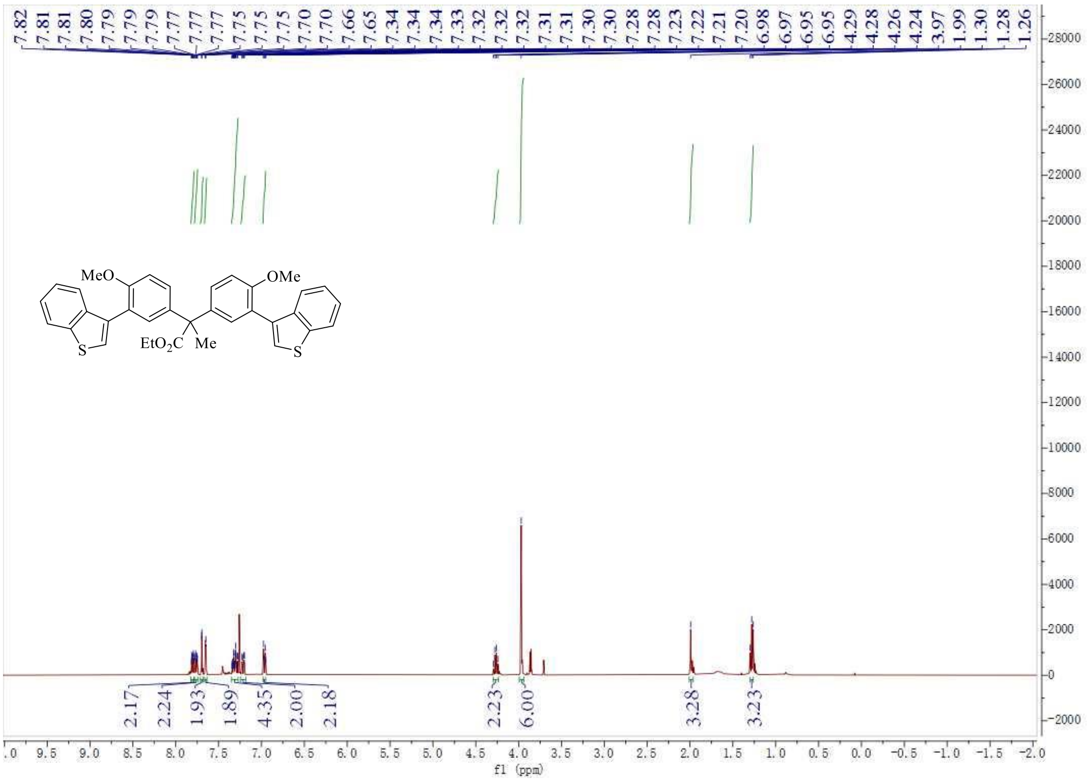

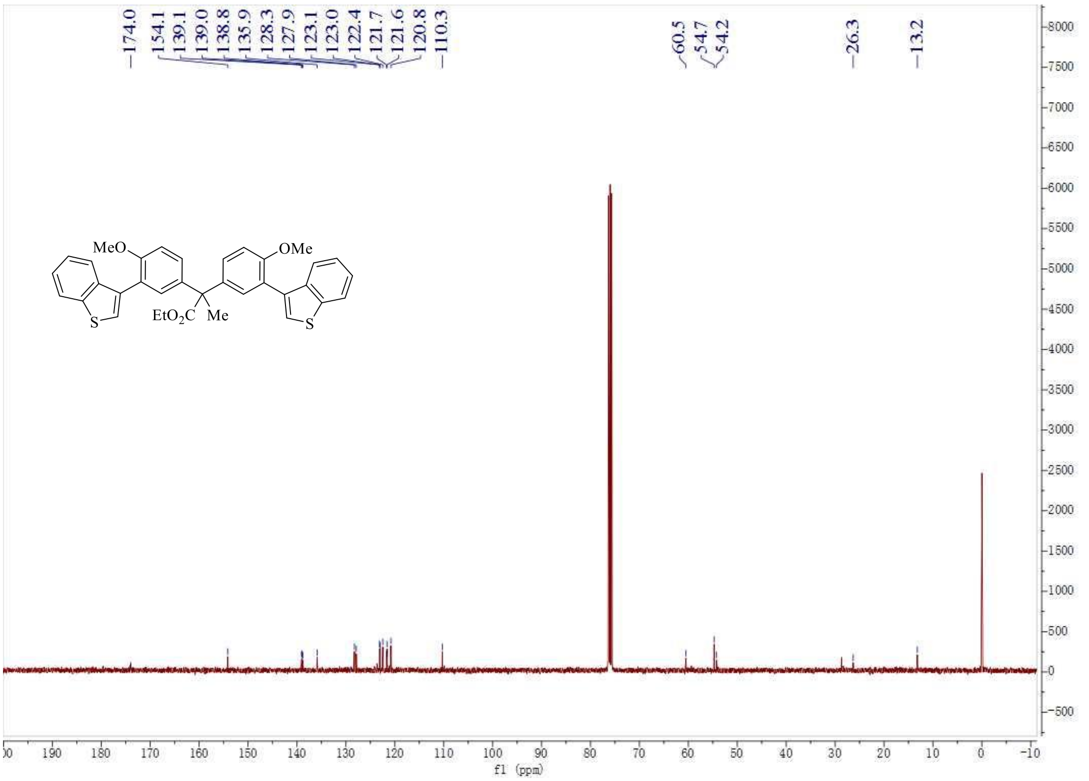

22

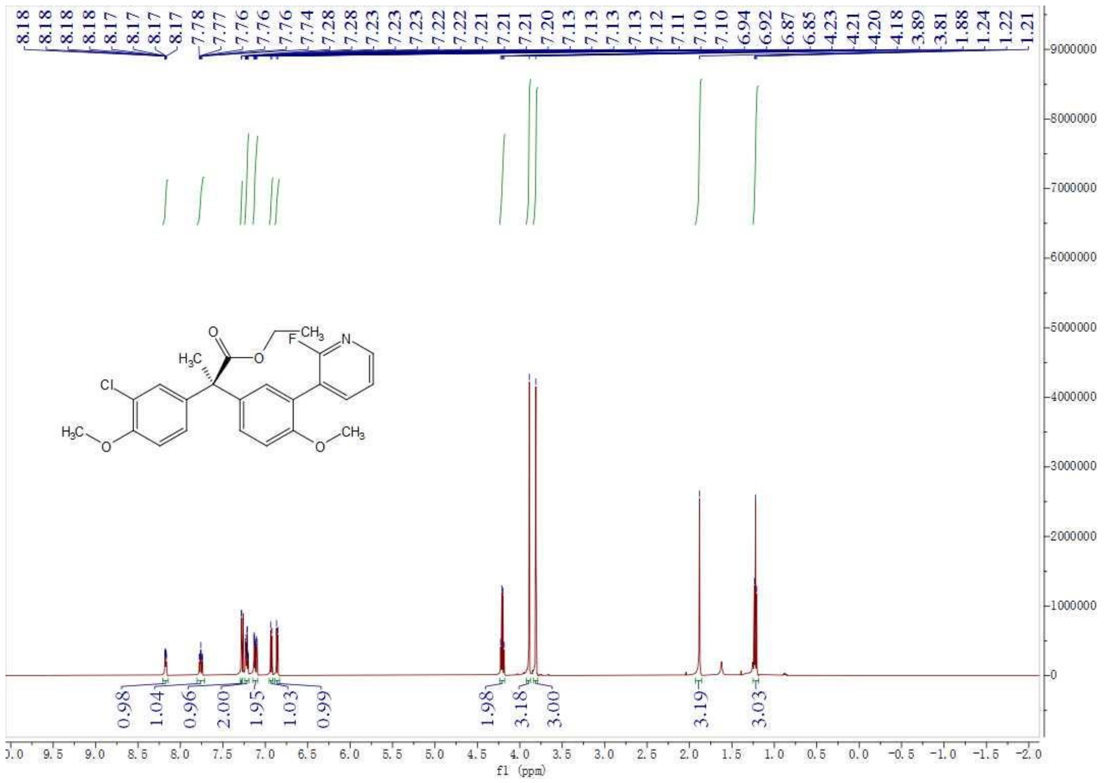

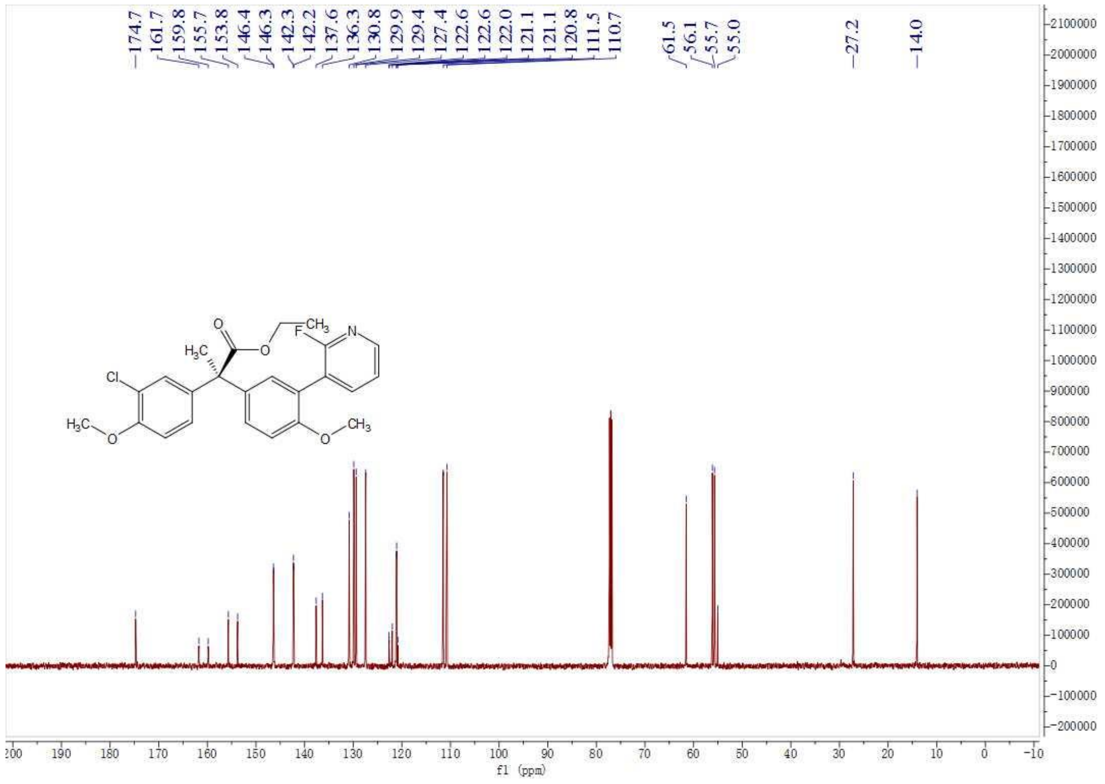

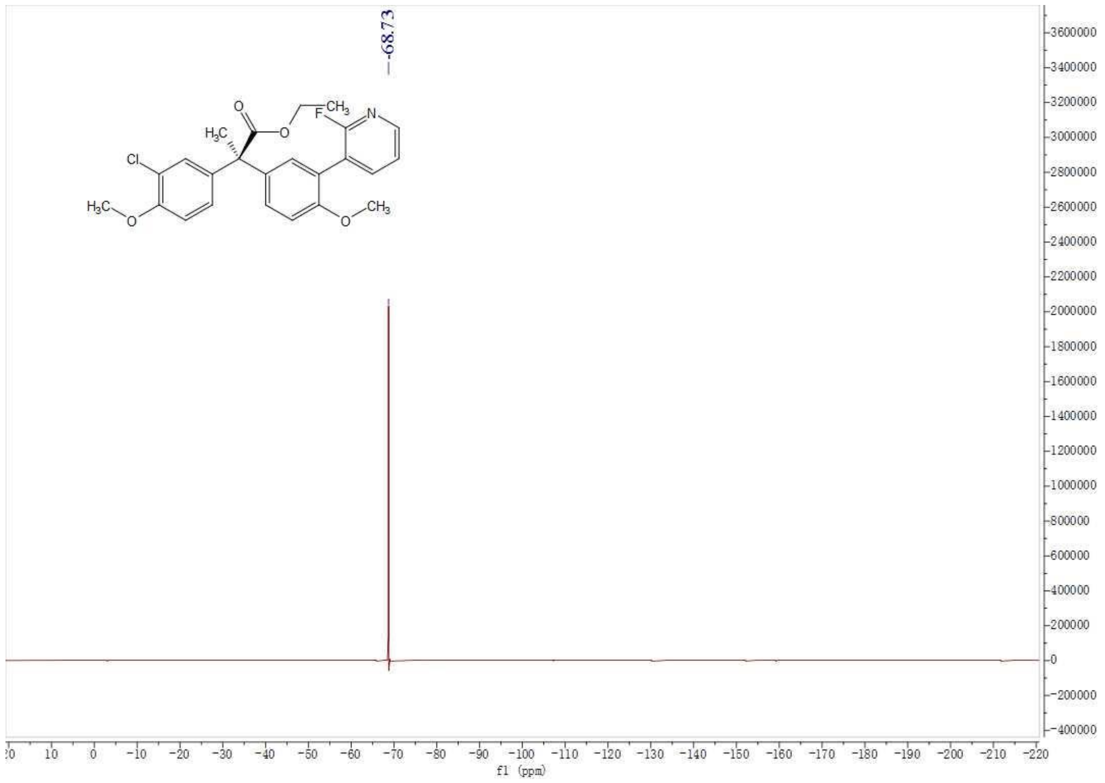

-68.73
f1 (ppm)

23

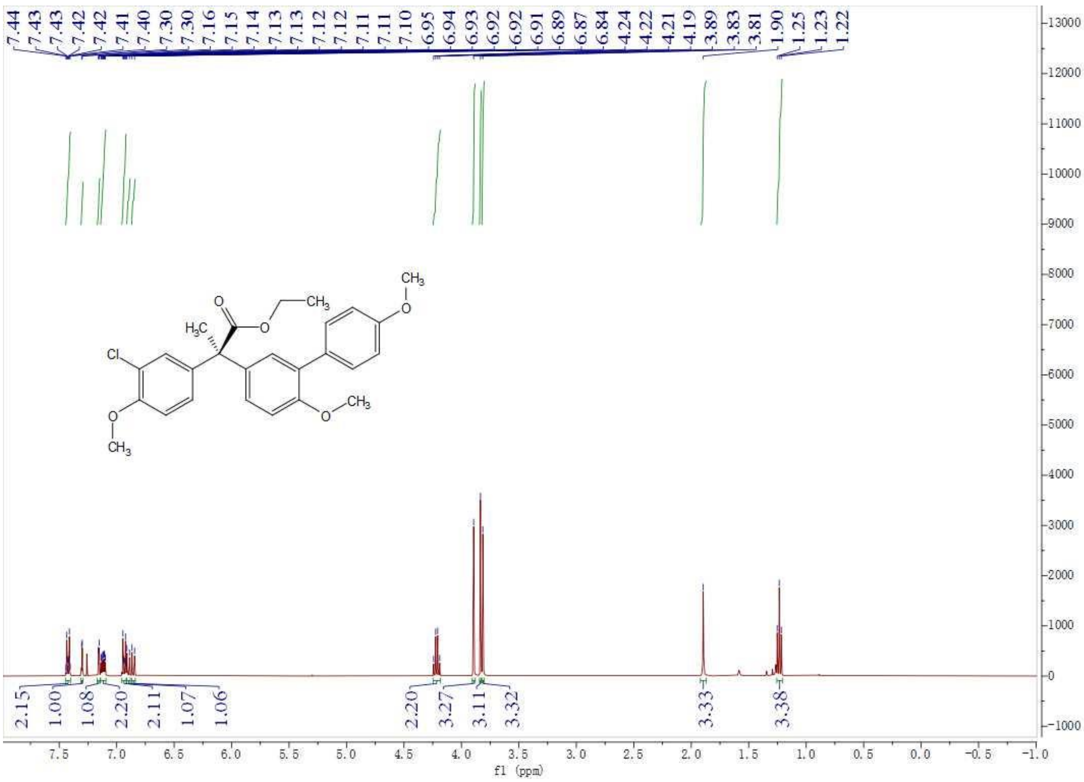
7.44 7.43 7.43 7.42 7.42 7.41 7.40 7.30 7.16 7.15 7.14 7.13 7.13 7.12 7.12 7.11 7.11 7.10 6.95 6.94 6.93 6.92 6.92 6.91 6.89 6.87 6.84 4.24 4.22 4.21 4.19 3.89 3.83 3.81 1.90 1.25 1.23 1.22
2.15 1.00 1.08 2.20 2.11 1.07 1.06 2.20 3.27 3.11 3.32 3.33 3.38
fl (ppm)

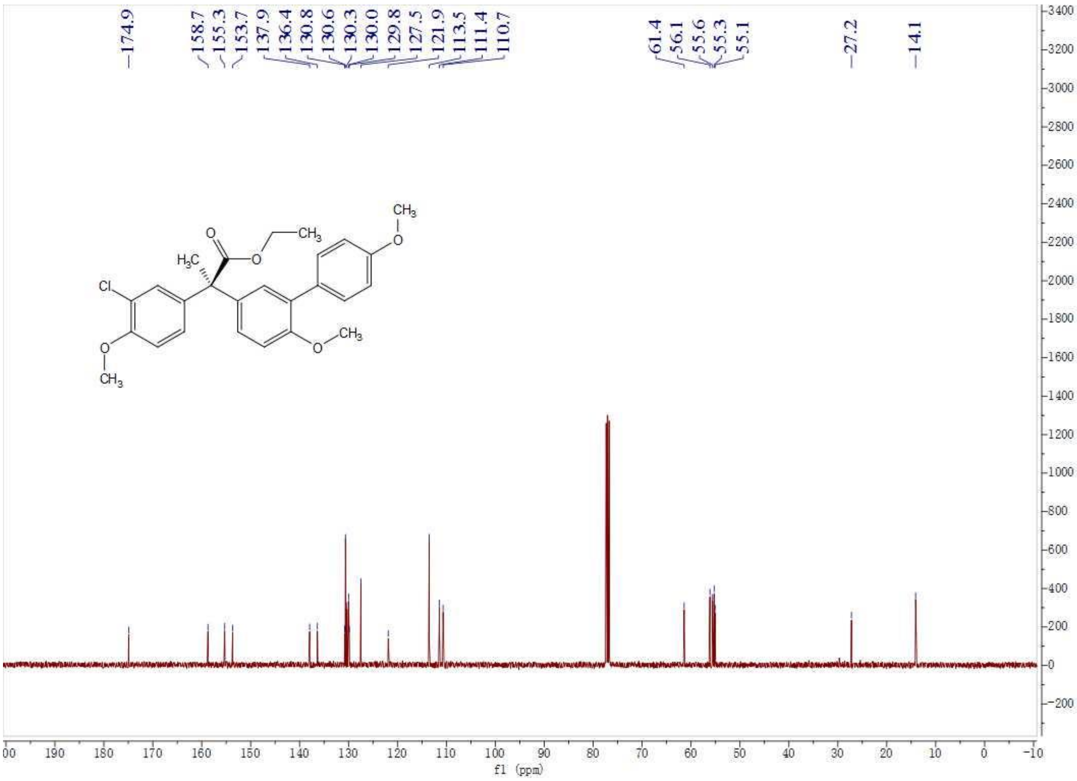
174.9 158.7 155.3 153.7 137.9 136.4 130.8 130.6 130.3 130.0 129.8 127.5 121.9 113.5 111.4 110.7 61.4 56.1 55.6 55.3 55.1 27.2 14.1
fl (ppm)

24

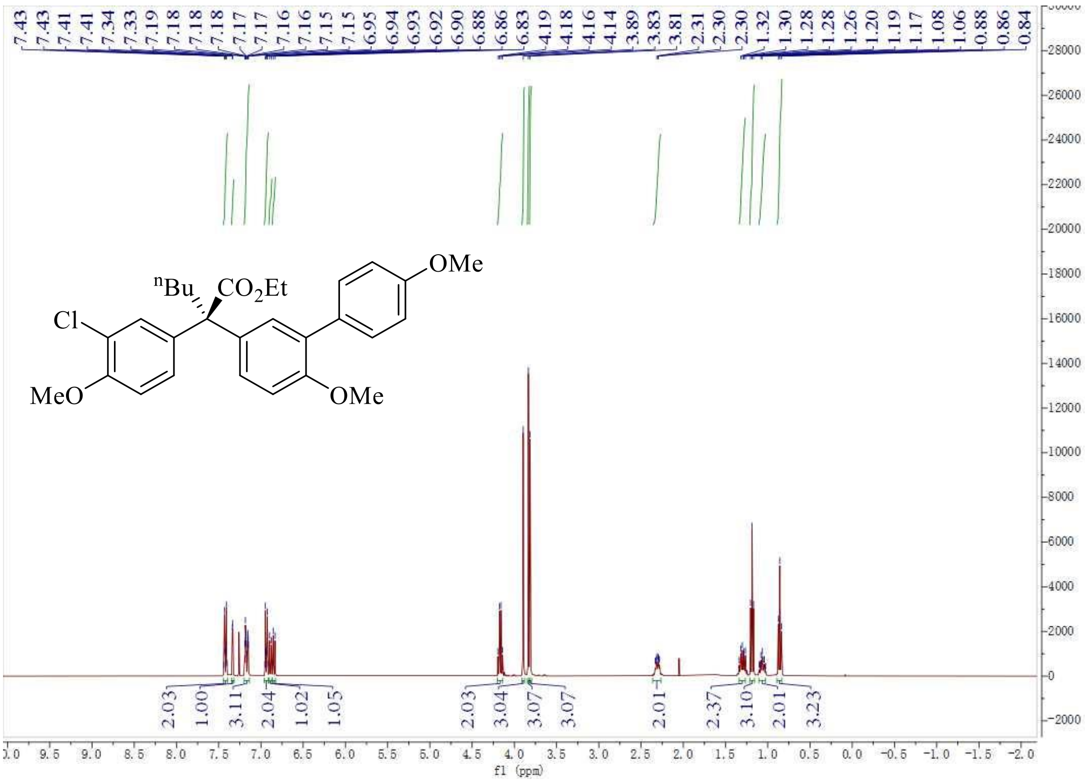

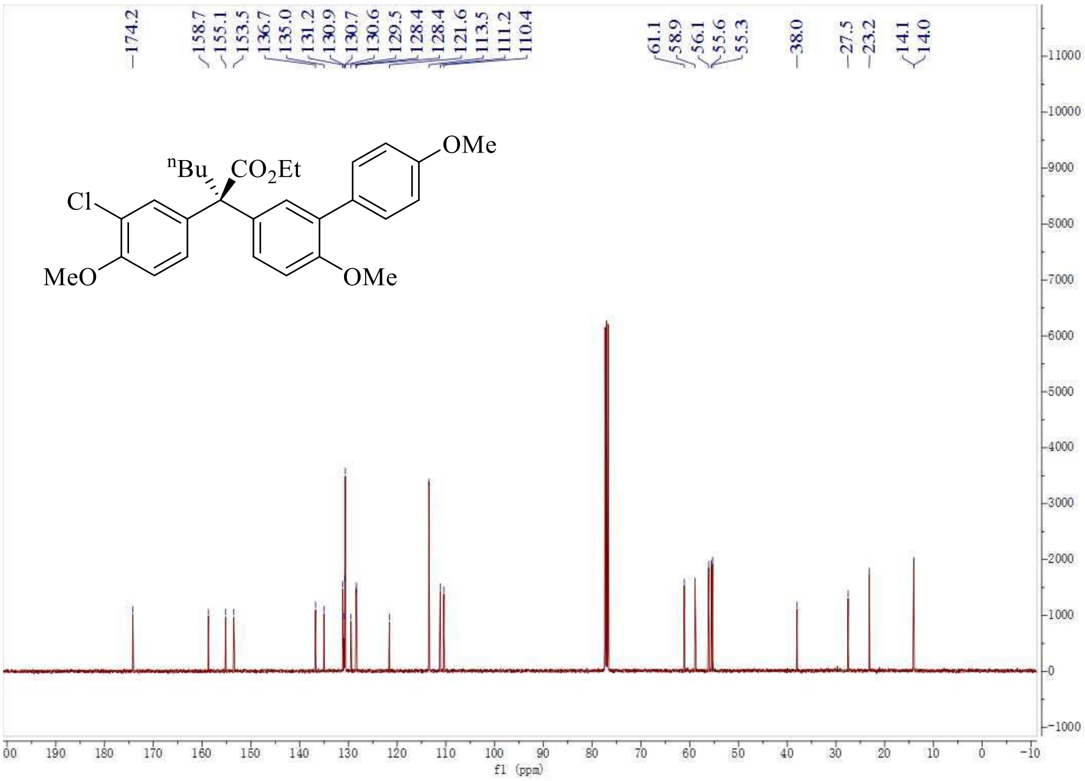

25

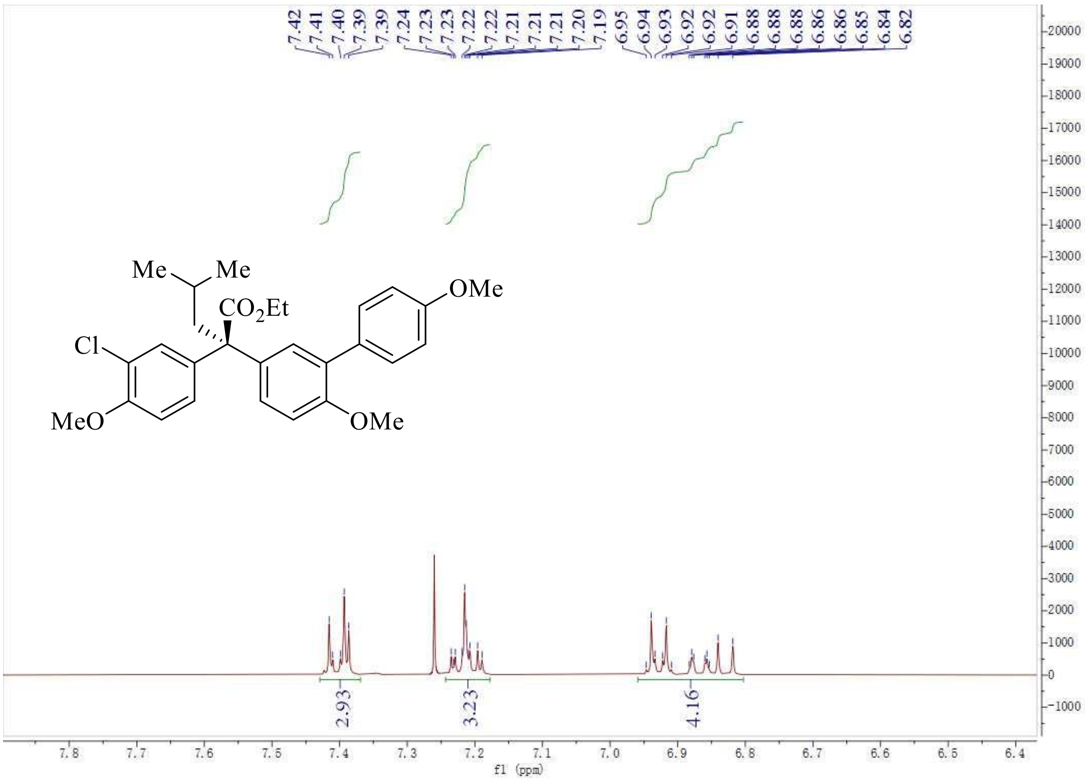

7.42
7.41
7.40
7.39
7.39
7.24
7.23
7.23
7.22
7.22
7.21
7.21
7.21
7.20
7.19
6.95
6.94
6.93
6.92
6.92
6.91
6.88
6.88
6.88
6.86
6.86
6.85
6.84
6.82
Me
Me
CO2Et
OMe
Cl
MeO
OMe
2.93
3.23
4.16
f1 (ppm)

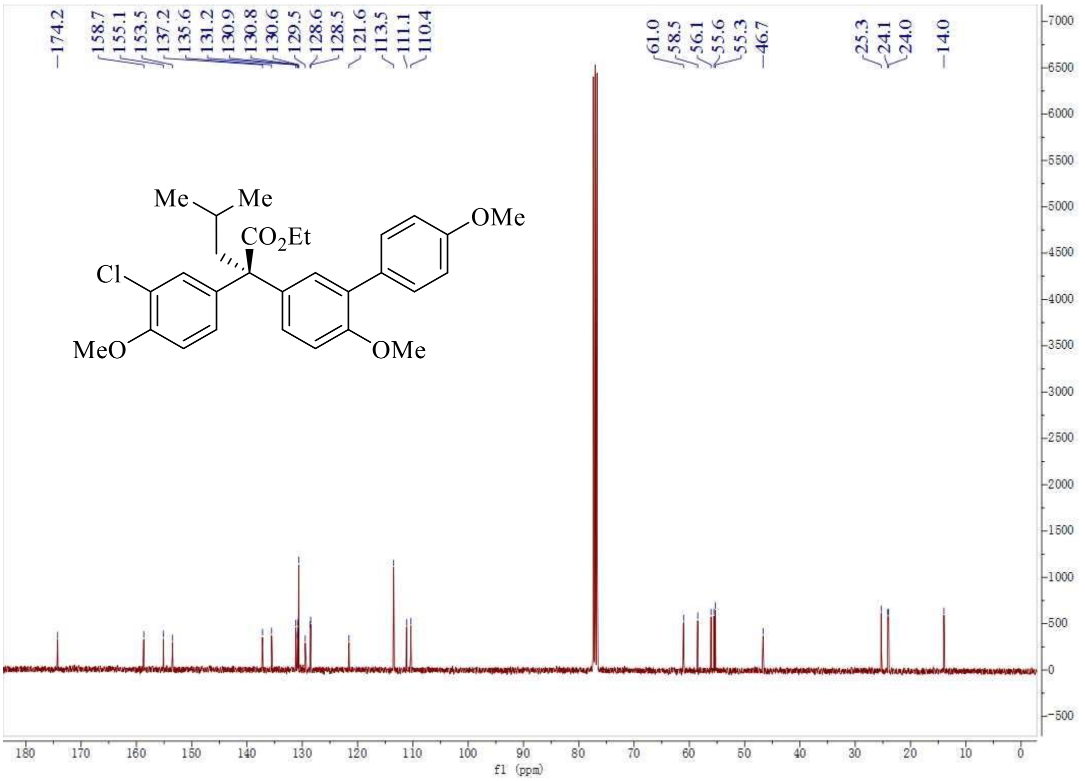

174.2
158.7
155.1
153.5
137.2
135.6
131.2
130.9
130.8
130.6
129.5
128.6
128.5
121.6
113.5
111.1
110.4
61.0
58.5
56.1
55.6
55.3
46.7
25.3
24.1
24.0
14.0
Me
Me
CO2Et
OMe
Cl
MeO
OMe
f1 (ppm)

26

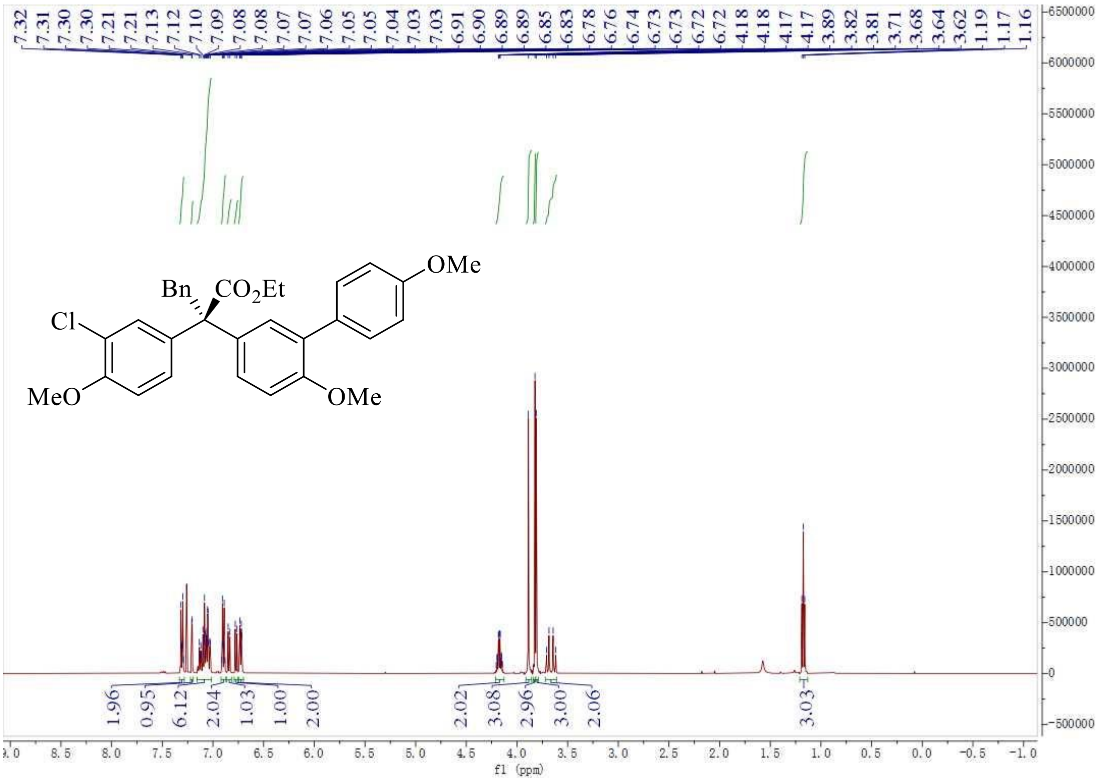

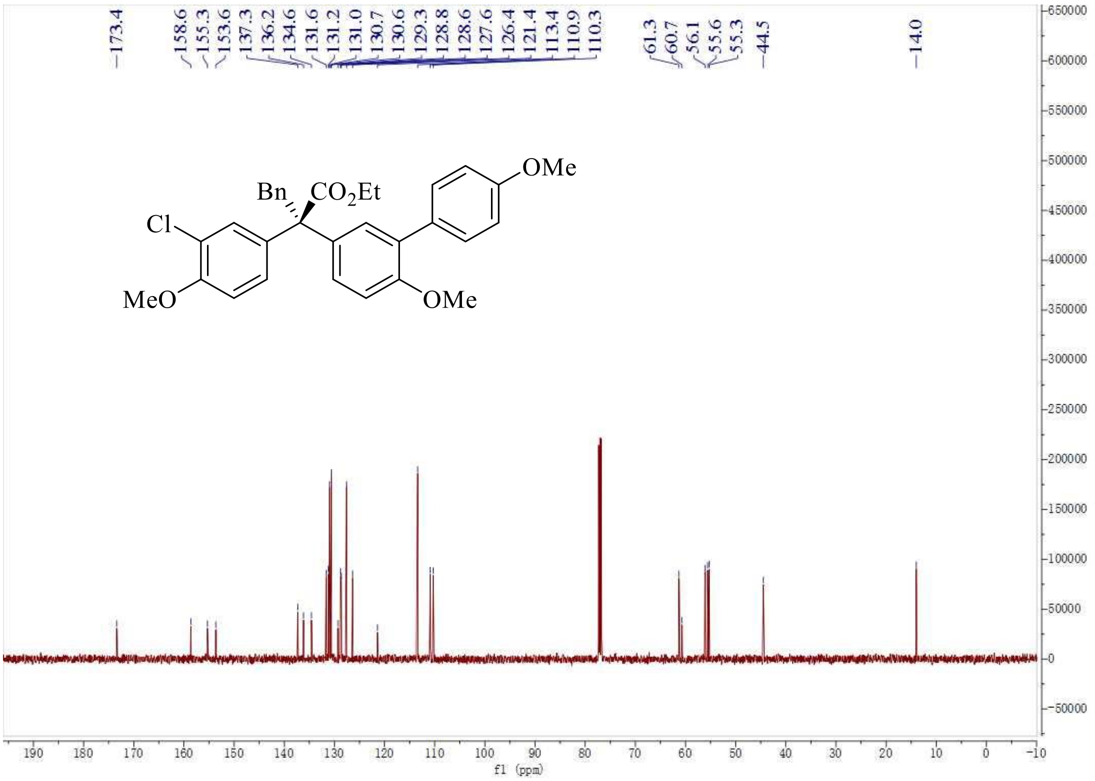

27

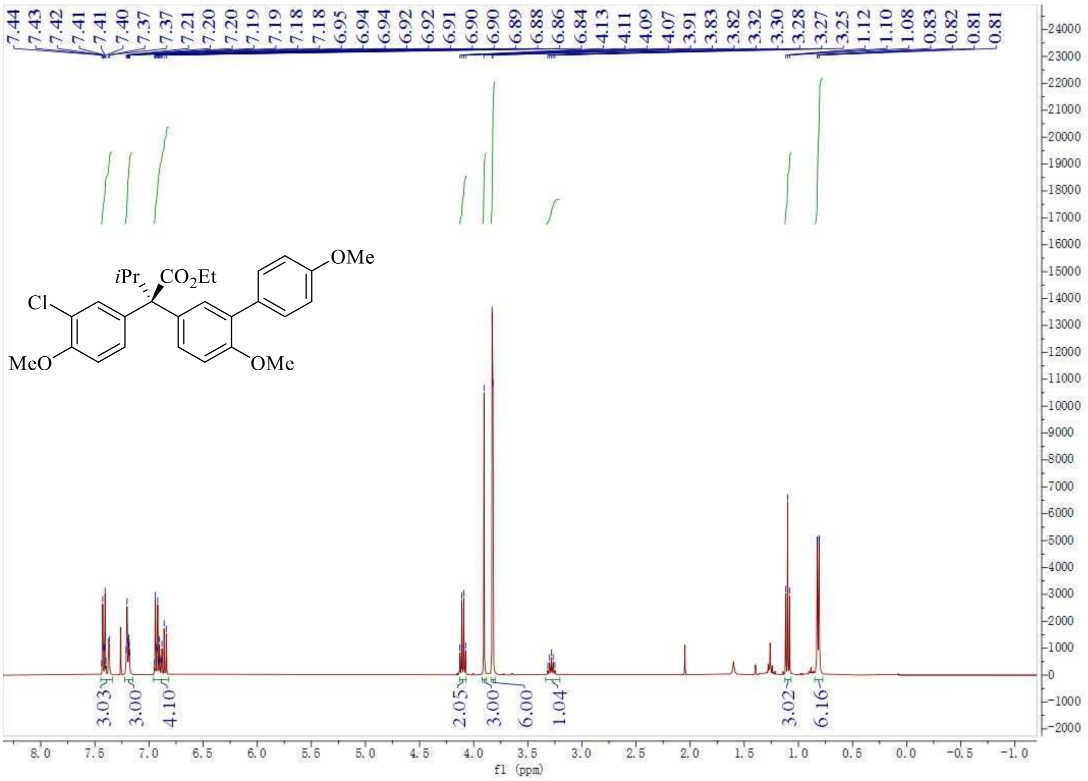

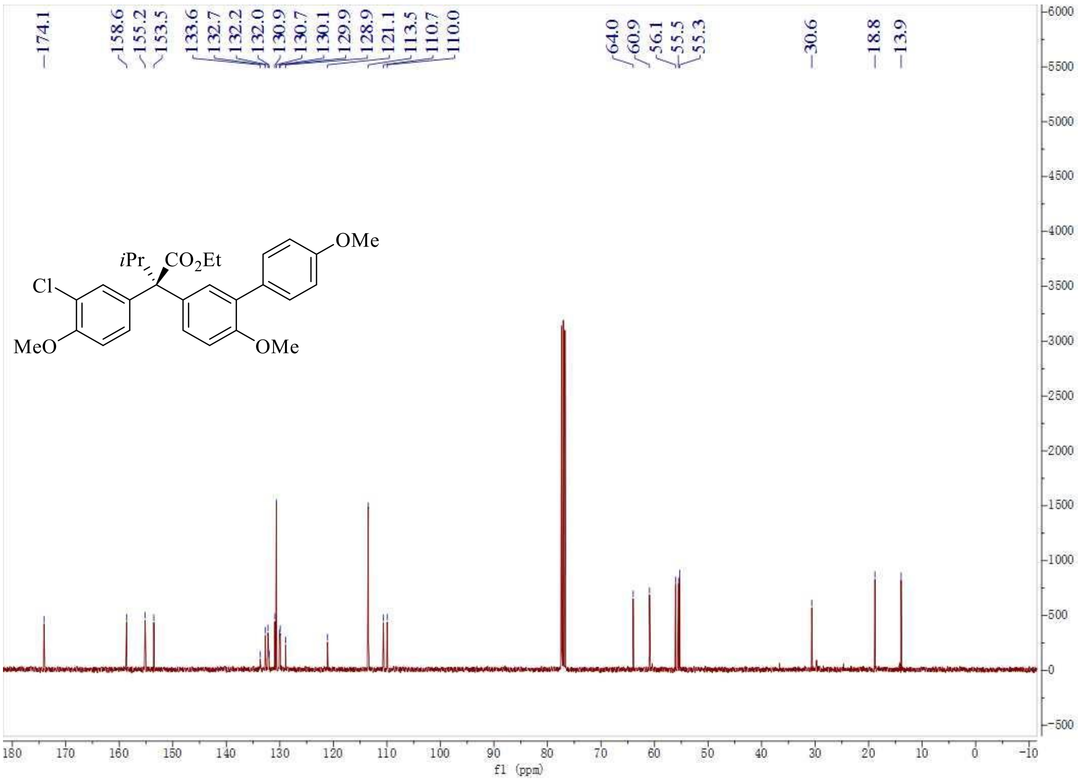

28

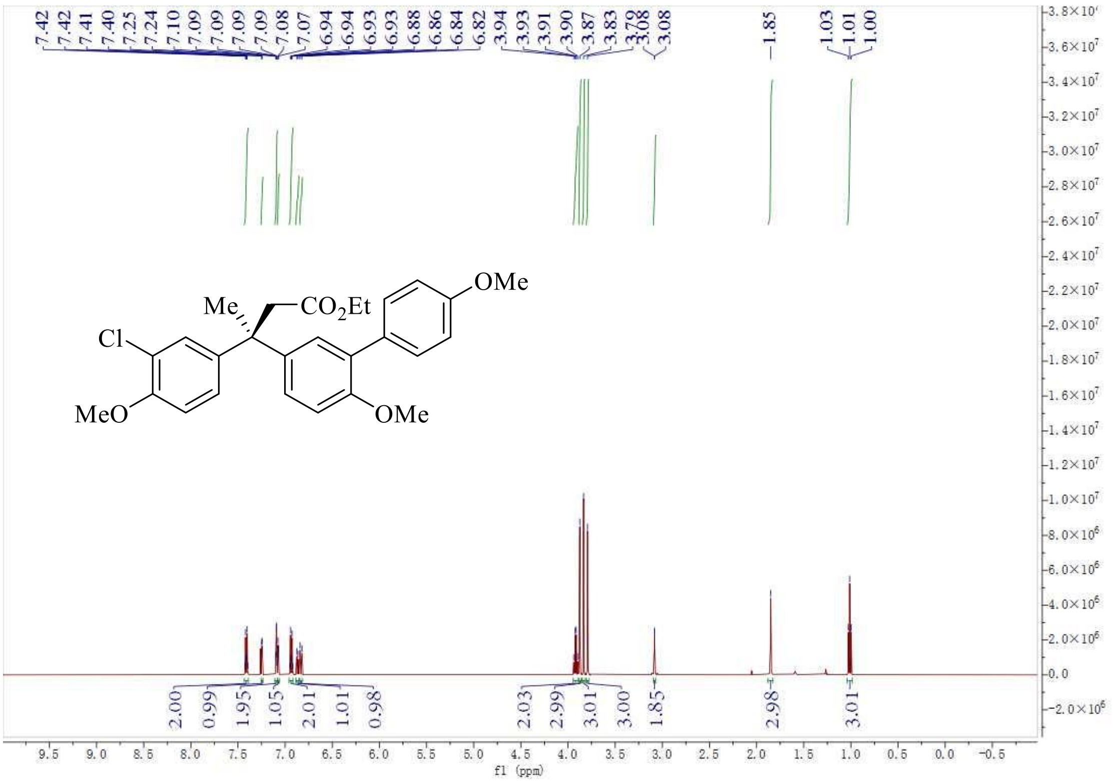

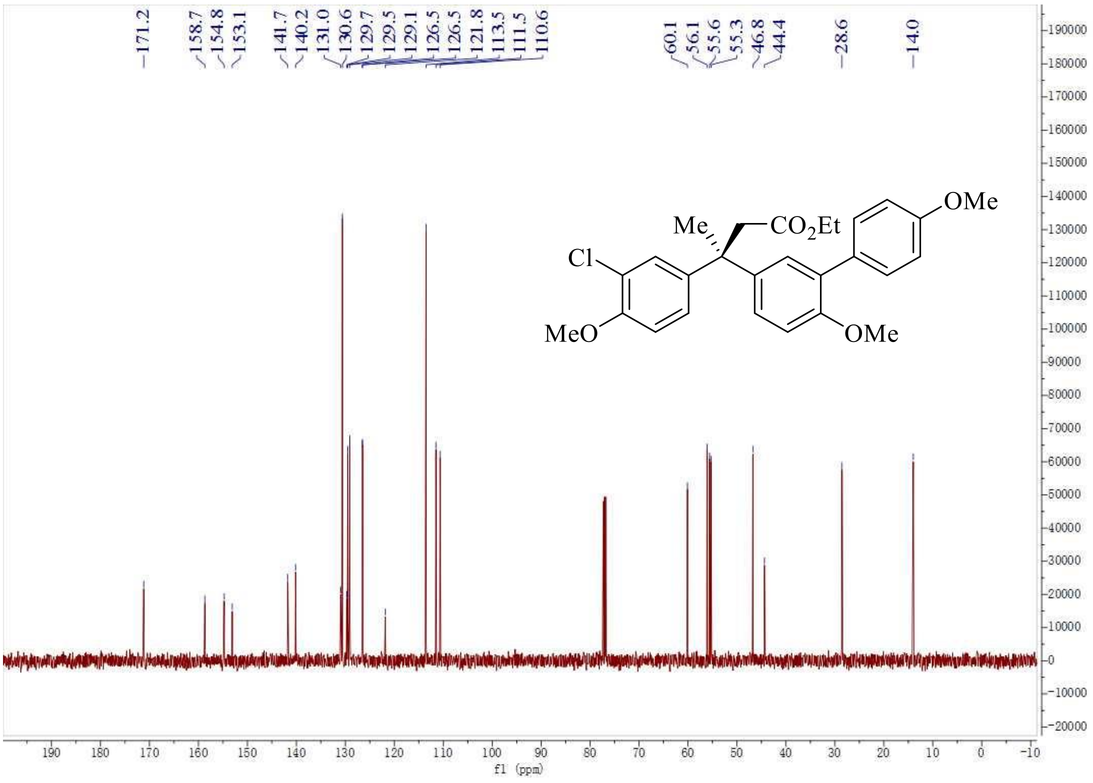

29

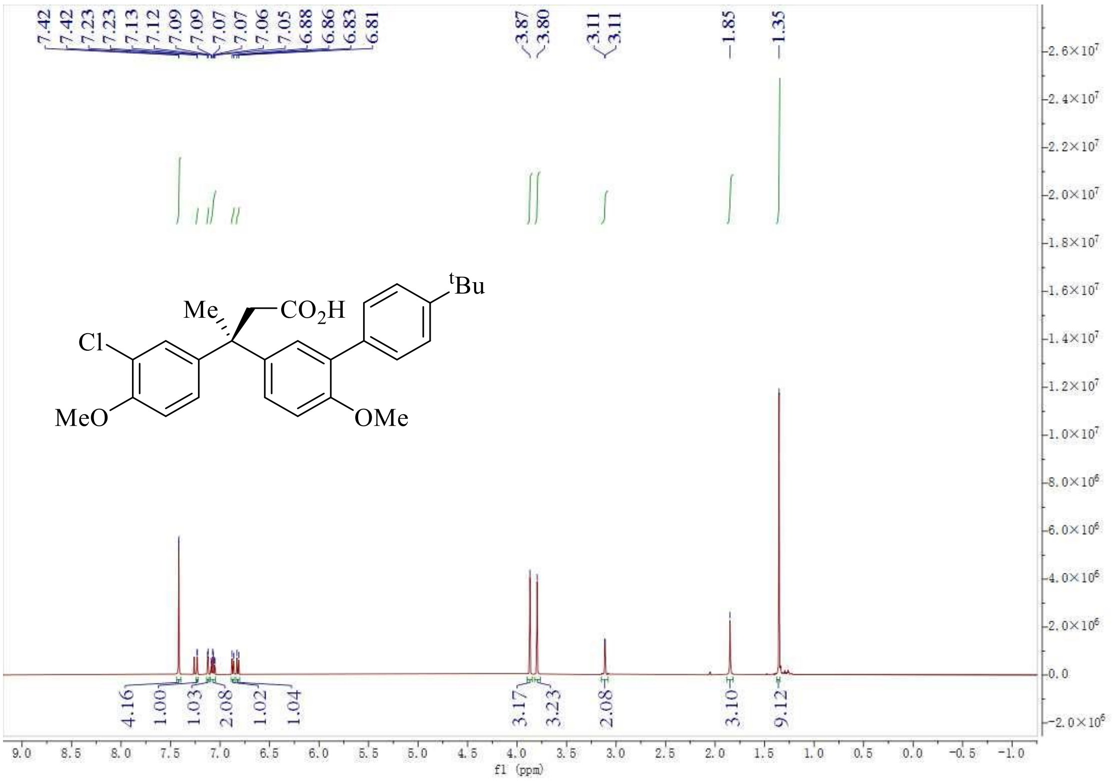

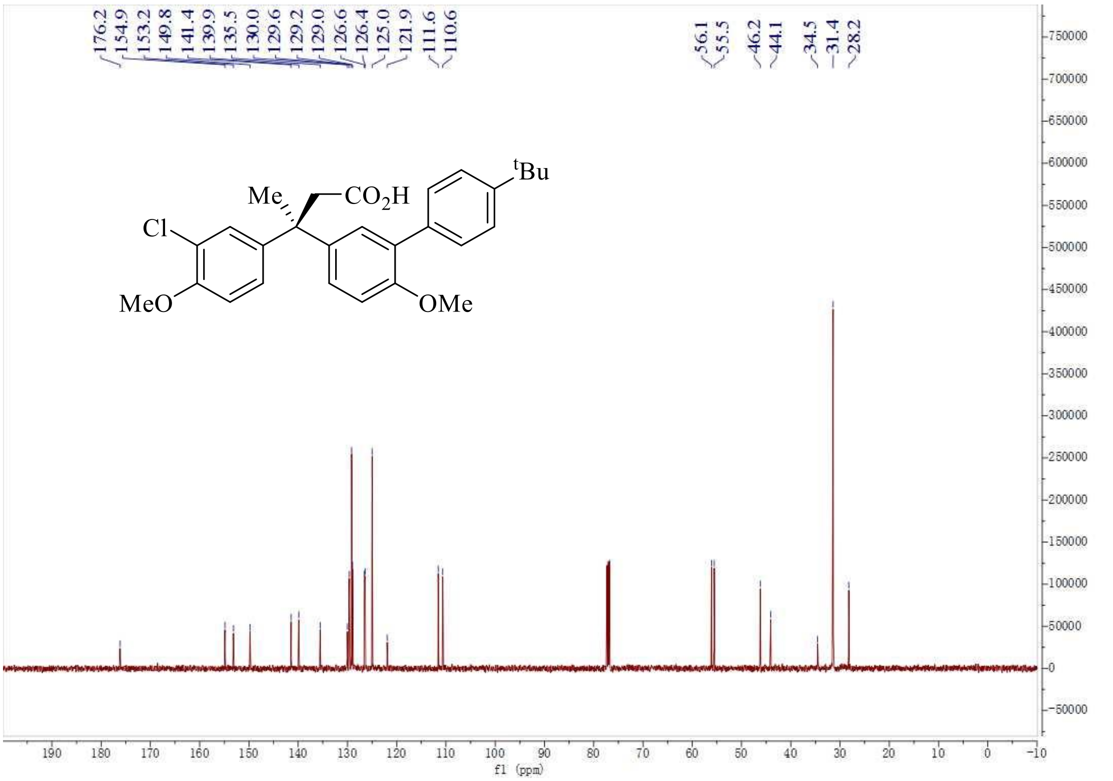

3

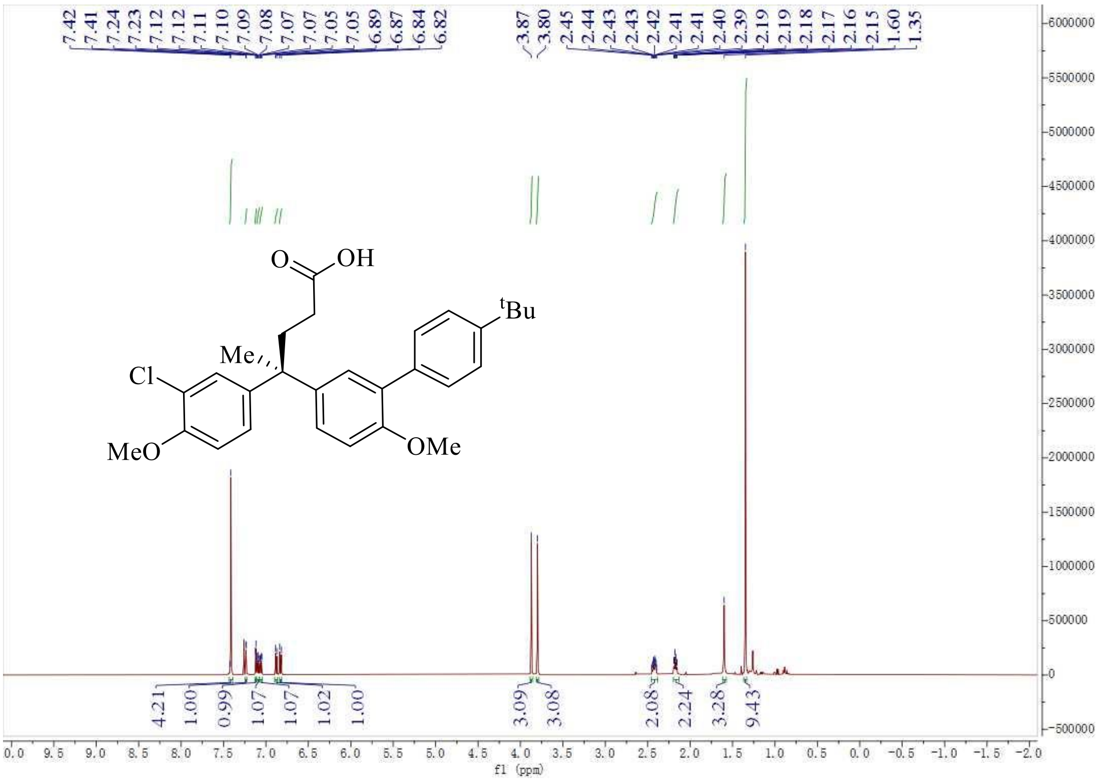
7.42
7.41
7.24
7.23
7.12
7.12
7.11
7.10
7.09
7.08
7.07
7.07
7.05
7.05
6.89
6.87
6.84
6.82
3.87
3.80
2.45
2.44
2.43
2.43
2.42
2.41
2.41
2.40
2.39
2.19
2.19
2.18
2.17
2.16
2.15
1.60
1.35
O
OH
tBu
Me
Cl
MeO
OMe
4.21
1.00
0.99
1.07
1.07
1.02
1.00
3.09
3.08
2.08
2.24
3.28
9.43
f1 (ppm)

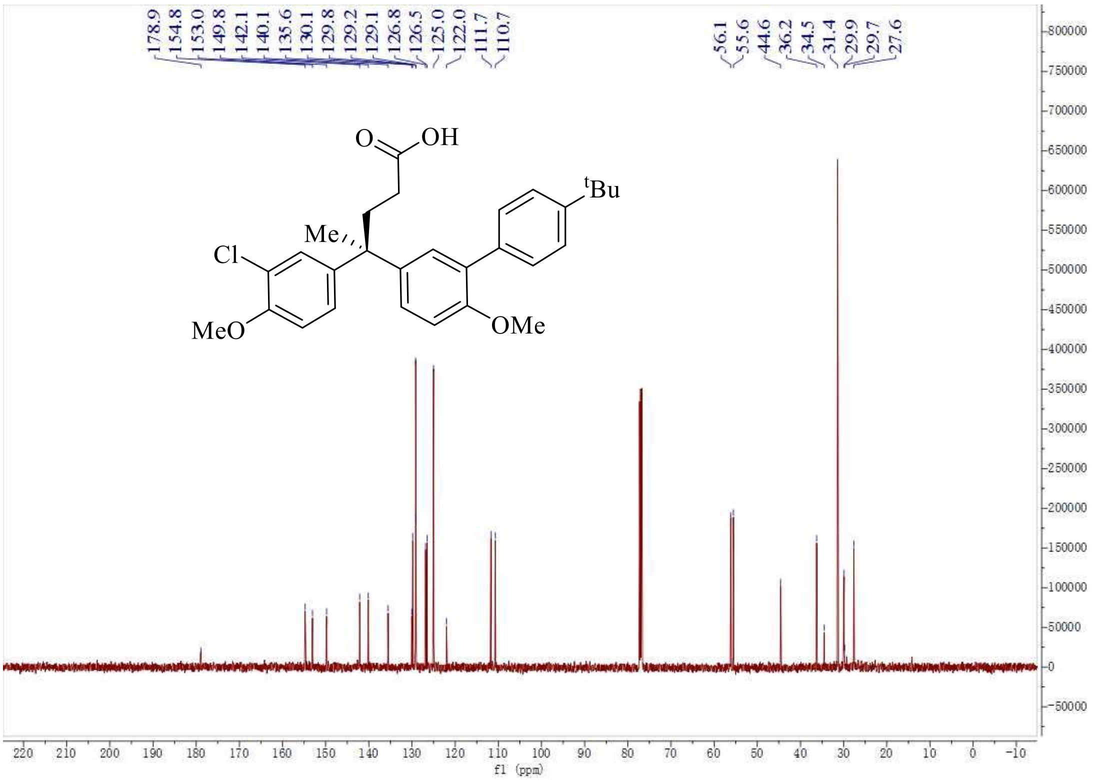
178.9
154.8
153.0
149.8
142.1
140.1
135.6
130.1
129.8
129.2
129.1
126.8
126.5
125.0
122.0
111.7
110.7
56.1
55.6
44.6
36.2
34.5
31.4
29.9
29.7
27.6
O
OH
tBu
Me
Cl
MeO
OMe
f1 (ppm)

30

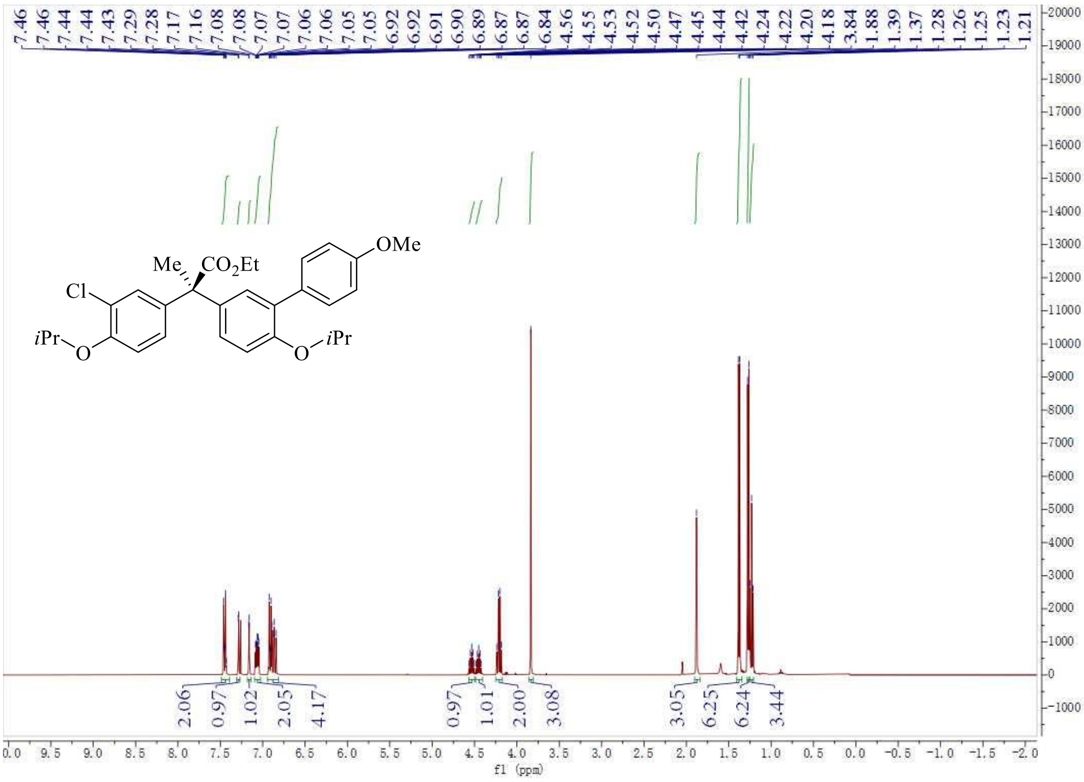

7.46
7.46
7.44
7.44
7.43
7.29
7.28
7.17
7.16
7.08
7.08
7.07
7.07
7.06
7.06
7.05
7.05
6.92
6.92
6.91
6.90
6.89
6.87
6.87
6.84
4.56
4.55
4.53
4.52
4.50
4.47
4.45
4.44
4.42
4.24
4.22
4.20
4.18
3.84
1.88
1.39
1.37
1.28
1.26
1.25
1.23
1.21
Me
CO_2Et
OMe
Cl
iPr
O
2.06
0.97
1.02
2.05
4.17
0.97
1.01
2.00
3.08
3.05
6.25
6.24
3.44
f1 (ppm)

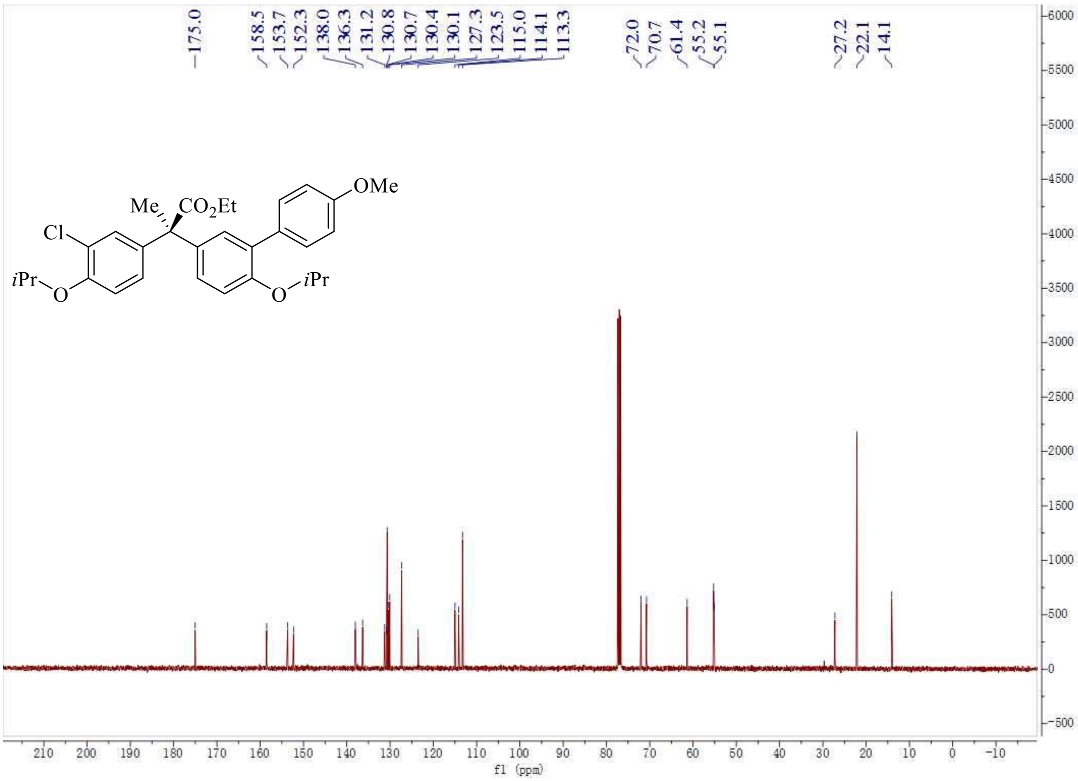

175.0
158.5
153.7
152.3
138.0
136.3
131.2
130.8
130.7
130.4
130.1
127.3
123.5
115.0
114.1
113.3
72.0
70.7
61.4
55.2
55.1
27.2
22.1
14.1
Me
CO_2Et
OMe
Cl
iPr
O
f1 (ppm)

31

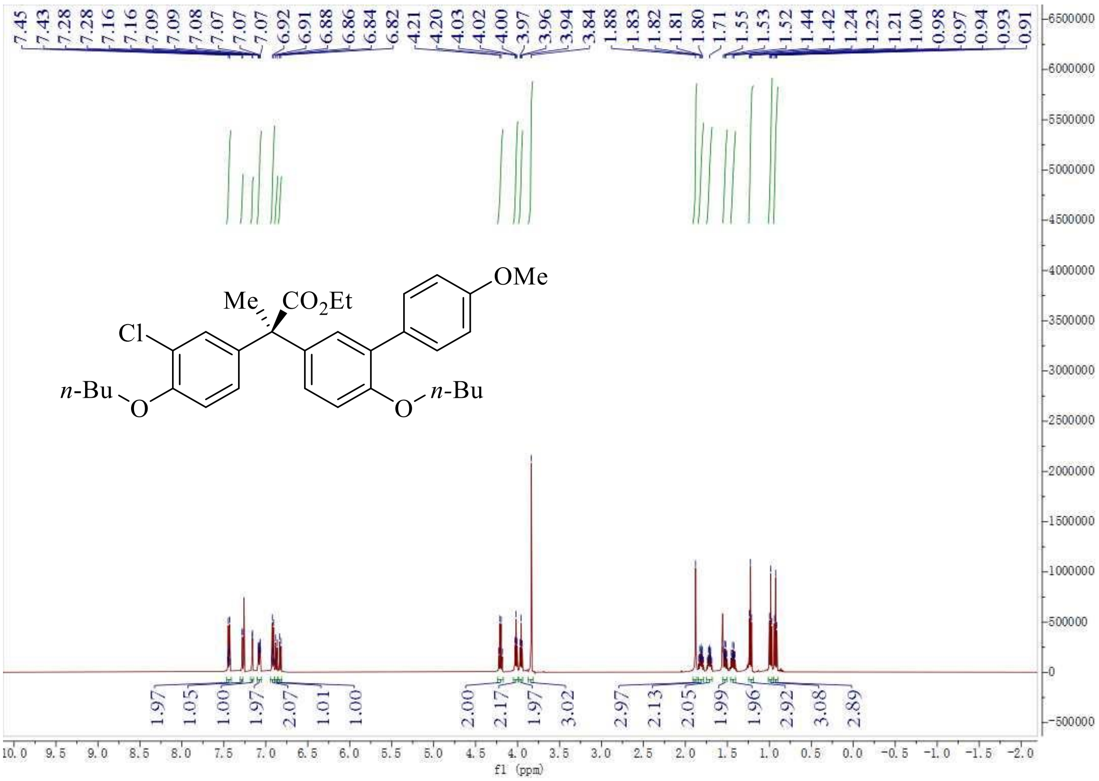

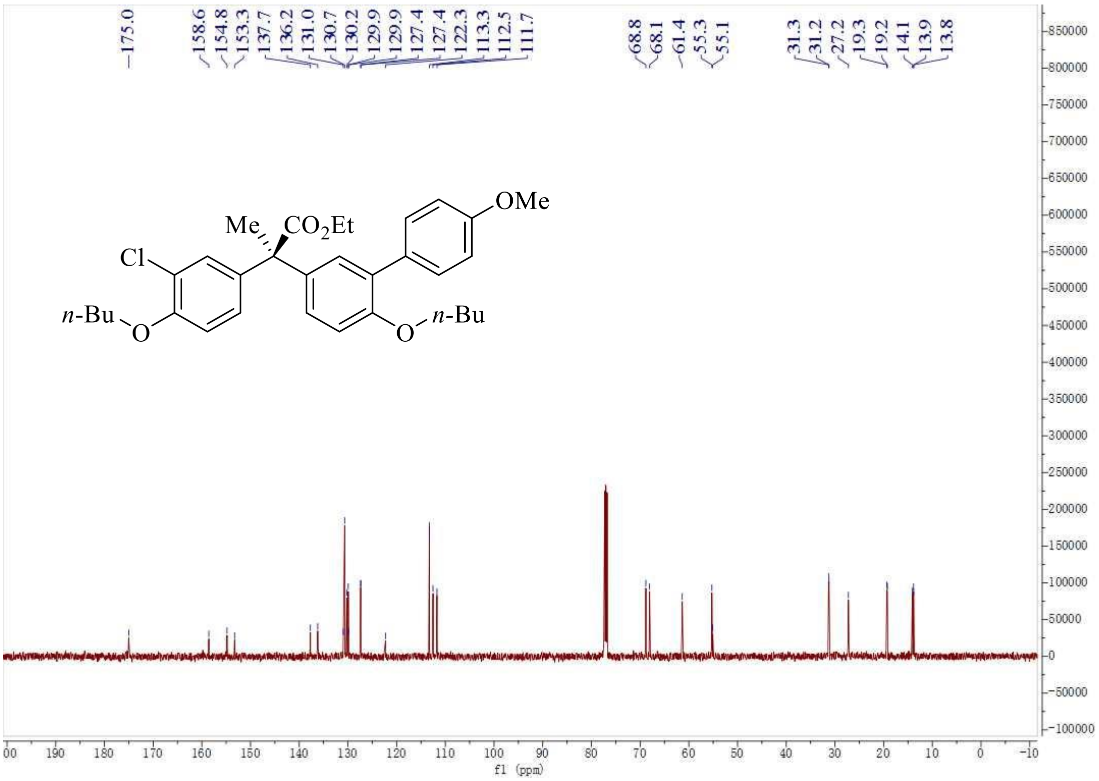

32

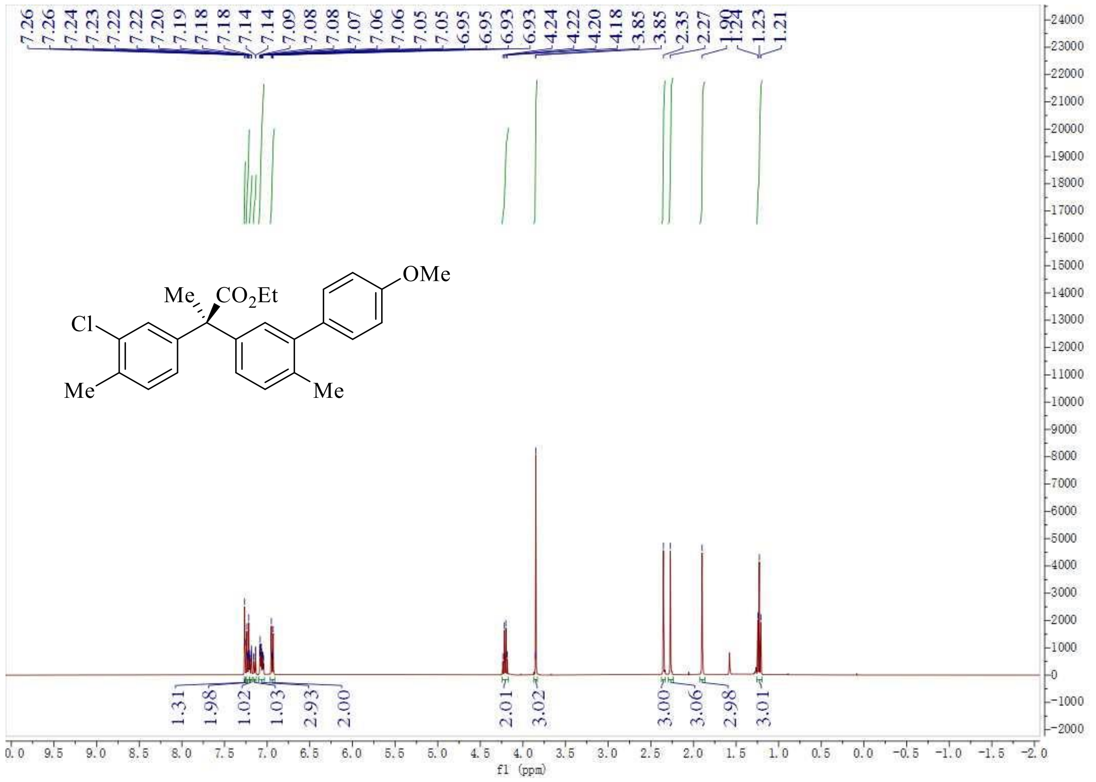

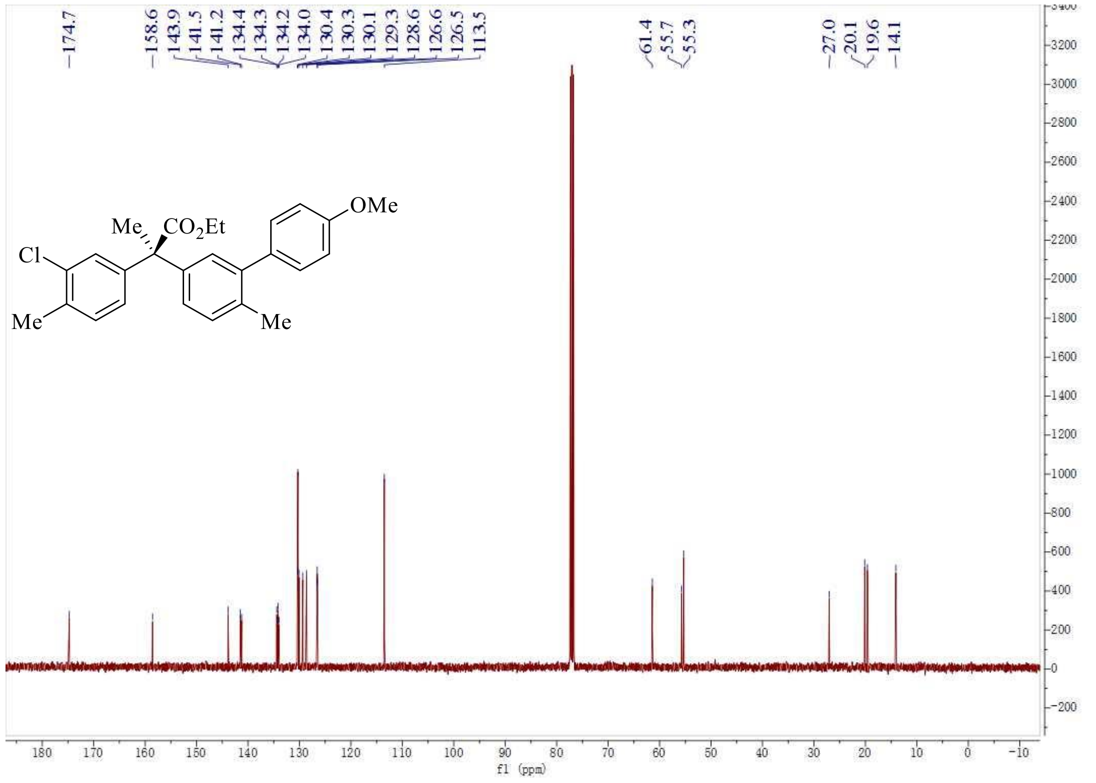

33

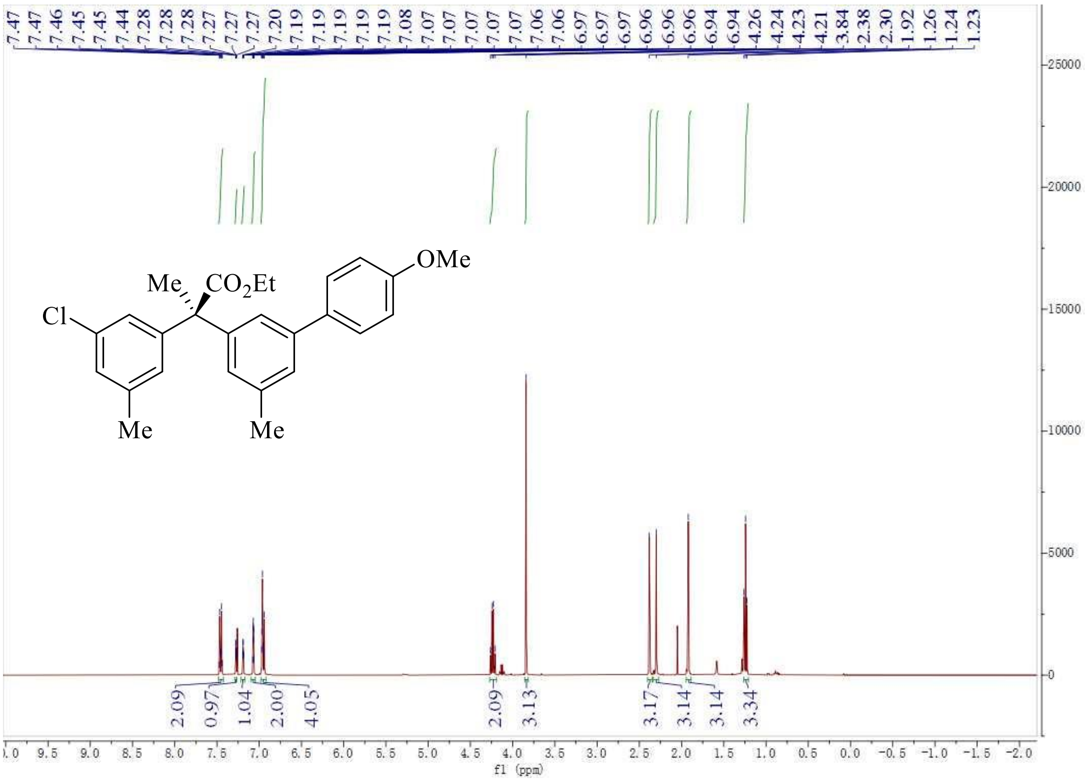

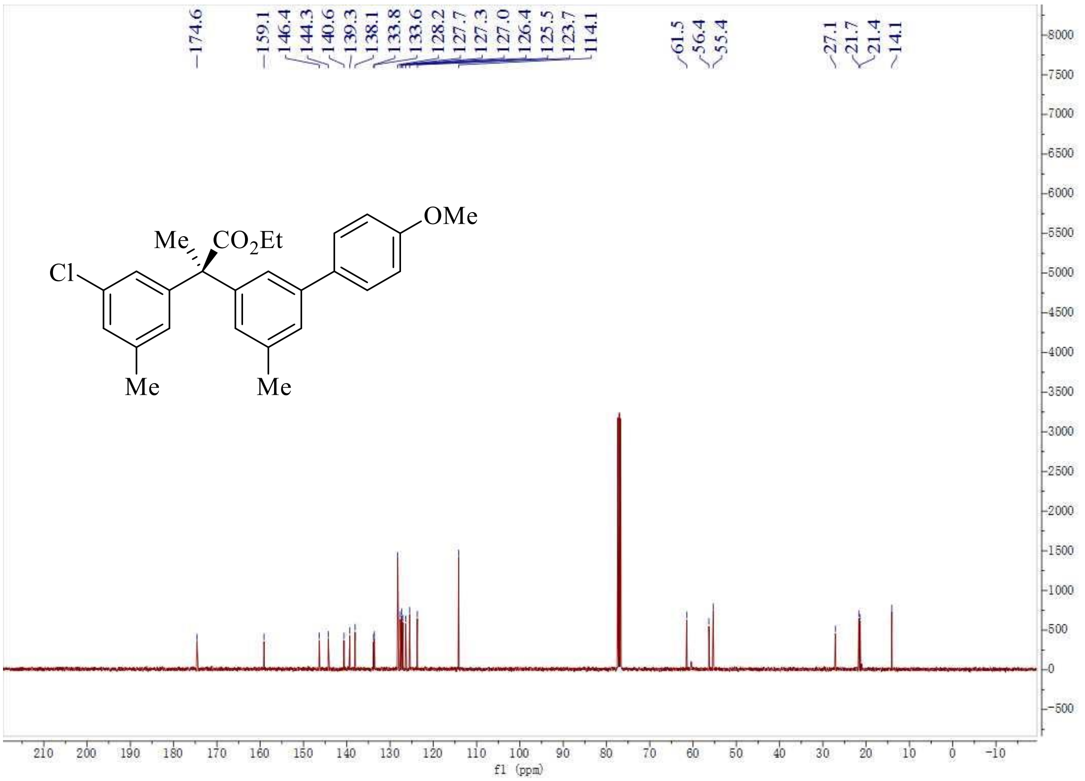

34

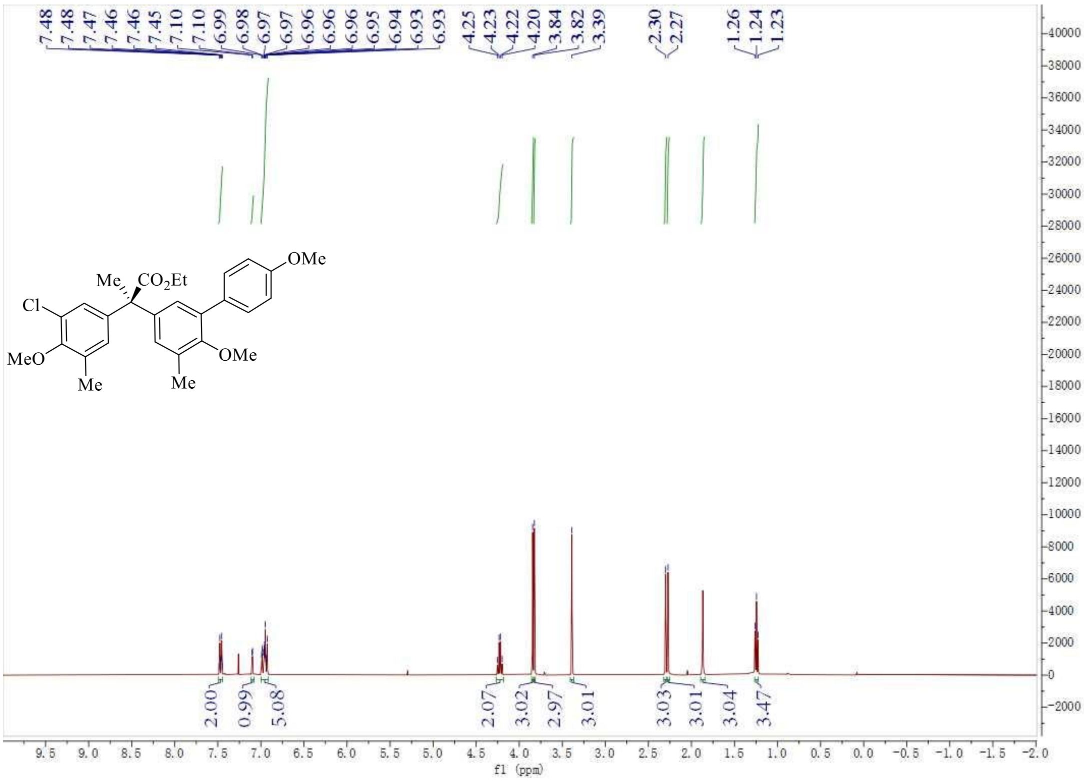

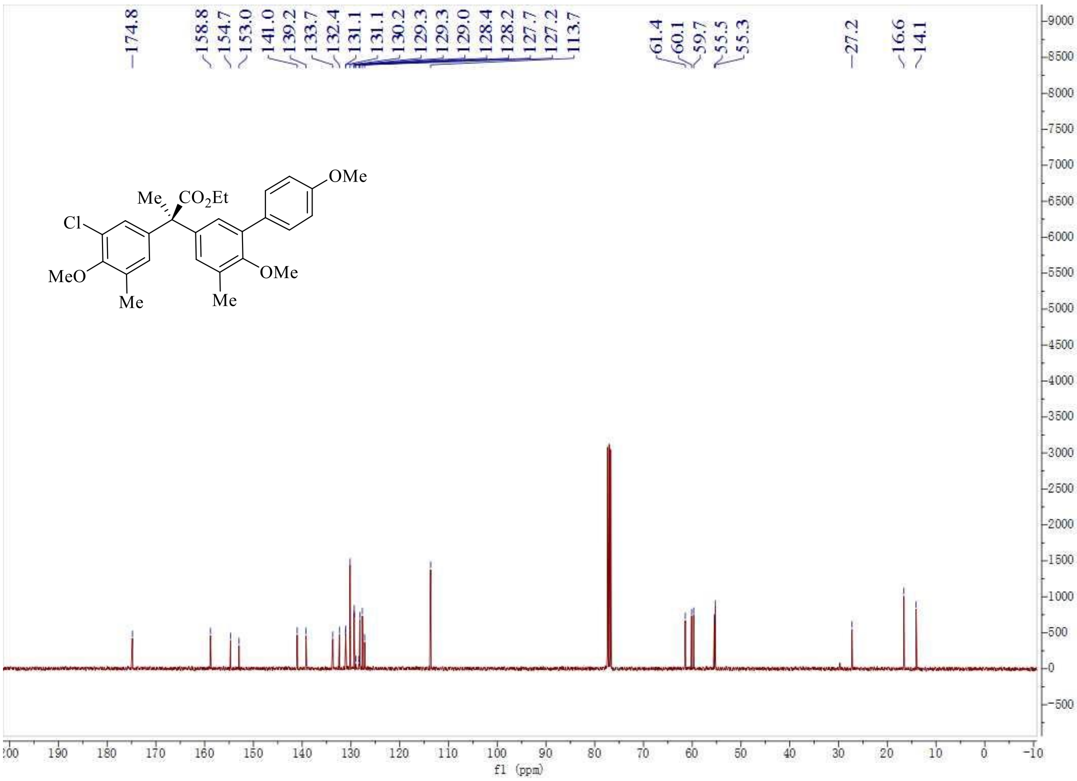

35

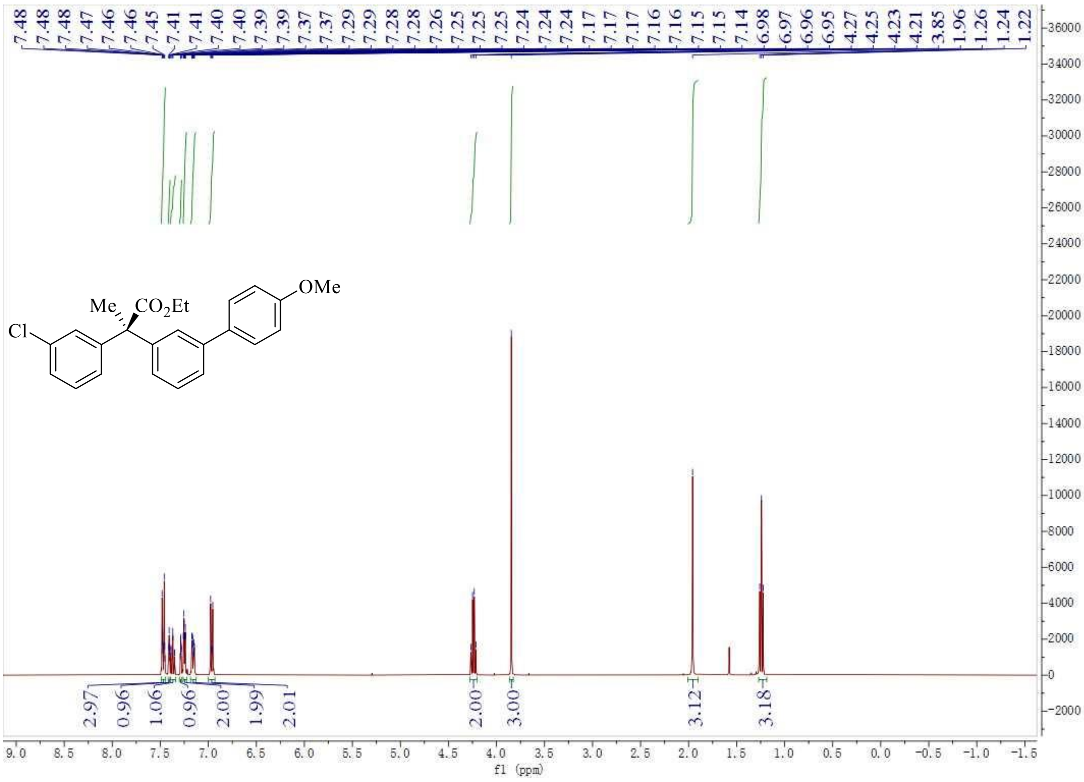

7.48 7.48 7.48 7.47 7.46 7.46 7.45 7.41 7.41 7.40 7.40 7.39 7.39 7.37 7.37 7.29 7.29 7.28 7.28 7.26 7.25 7.25 7.25 7.24 7.24 7.24 7.17 7.17 7.17 7.16 7.16 7.15 7.15 7.14 6.98 6.97 6.96 6.95 4.27 4.25 4.23 4.21 3.85 1.96 1.26 1.24 1.22
OMe
Me
CO_2Et
Cl
2.97 0.96 1.06 0.96 2.00 1.99 2.01 2.00 3.00 3.12 3.18
f1 (ppm)

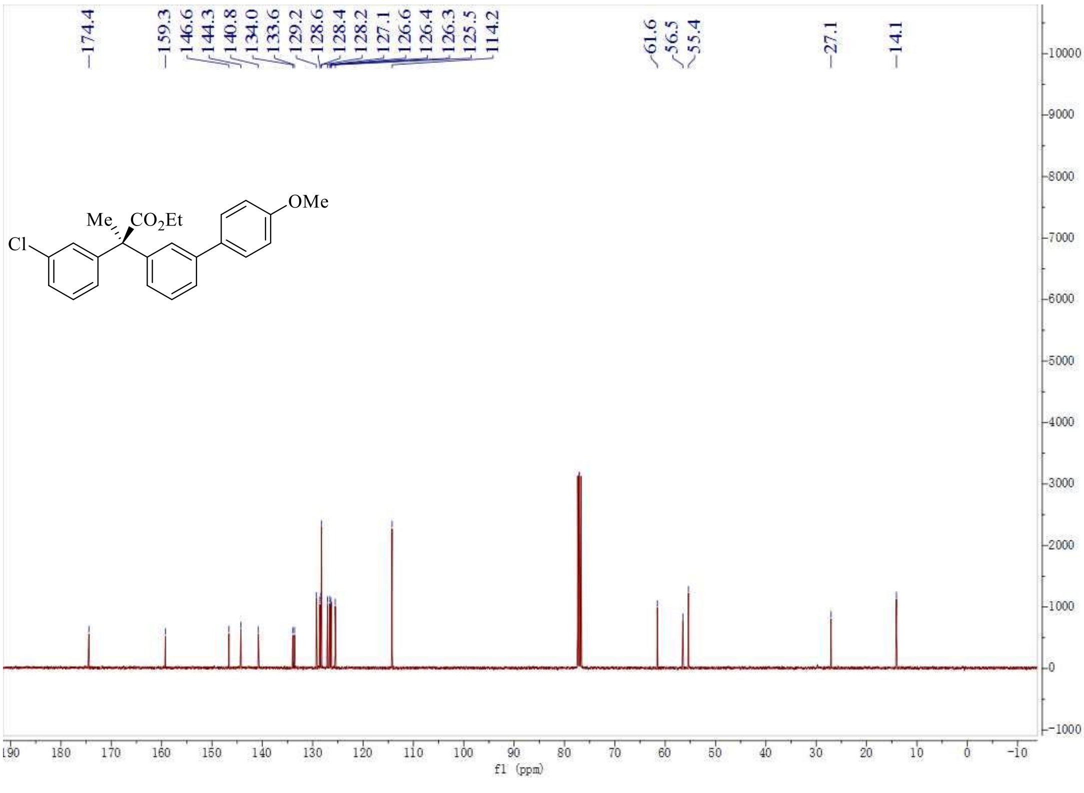

174.4 159.3 146.6 144.3 140.8 134.0 133.6 129.2 128.6 128.4 128.2 127.1 126.6 126.4 126.3 125.5 114.2 61.6 56.5 55.4 27.1 14.1
OMe
Me
CO_2Et
Cl
f1 (ppm)

36

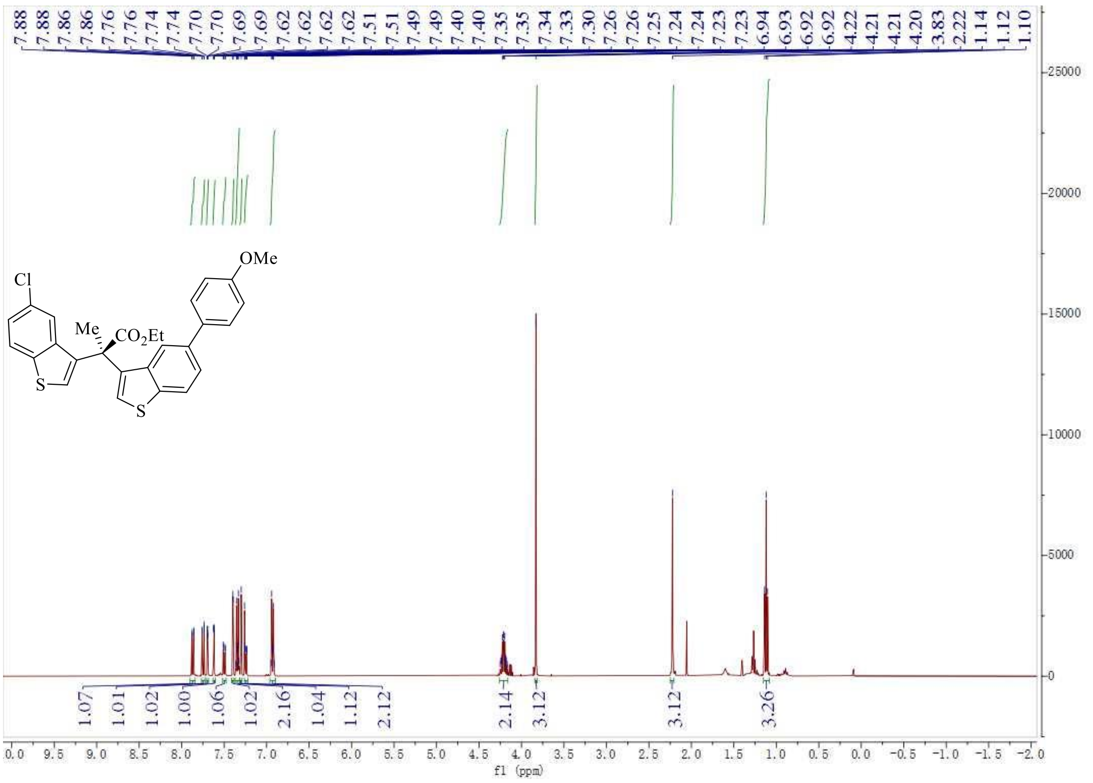

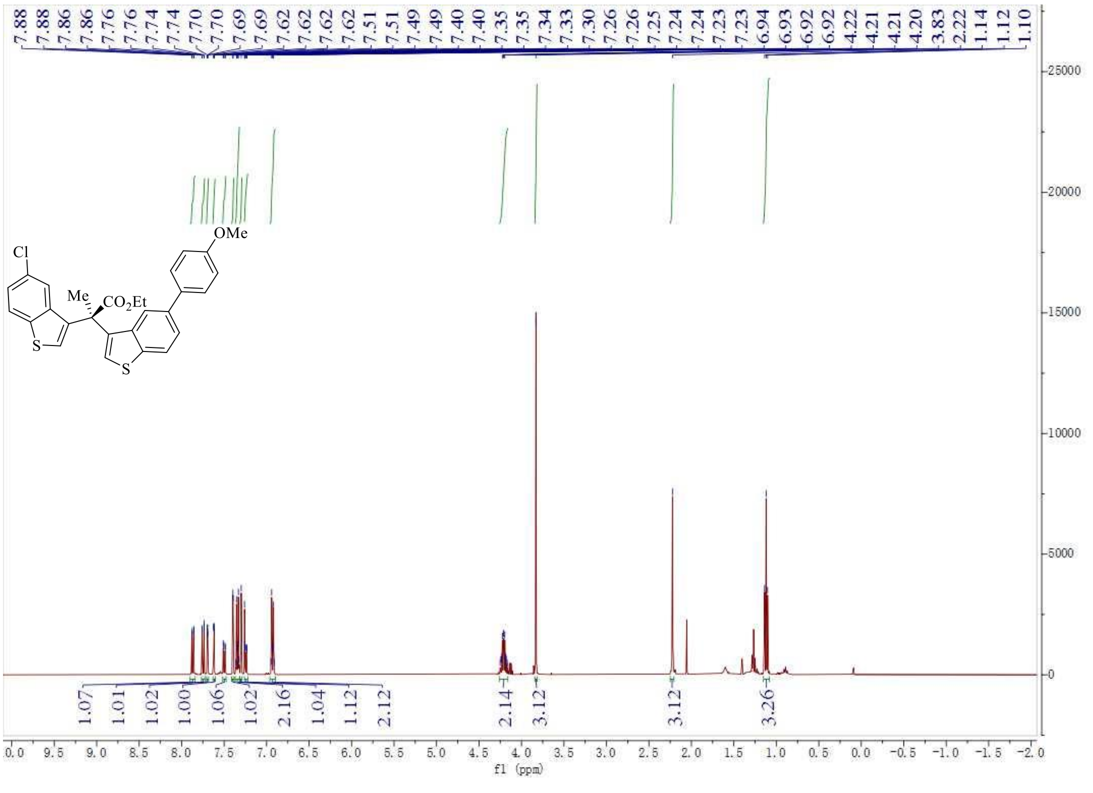

37

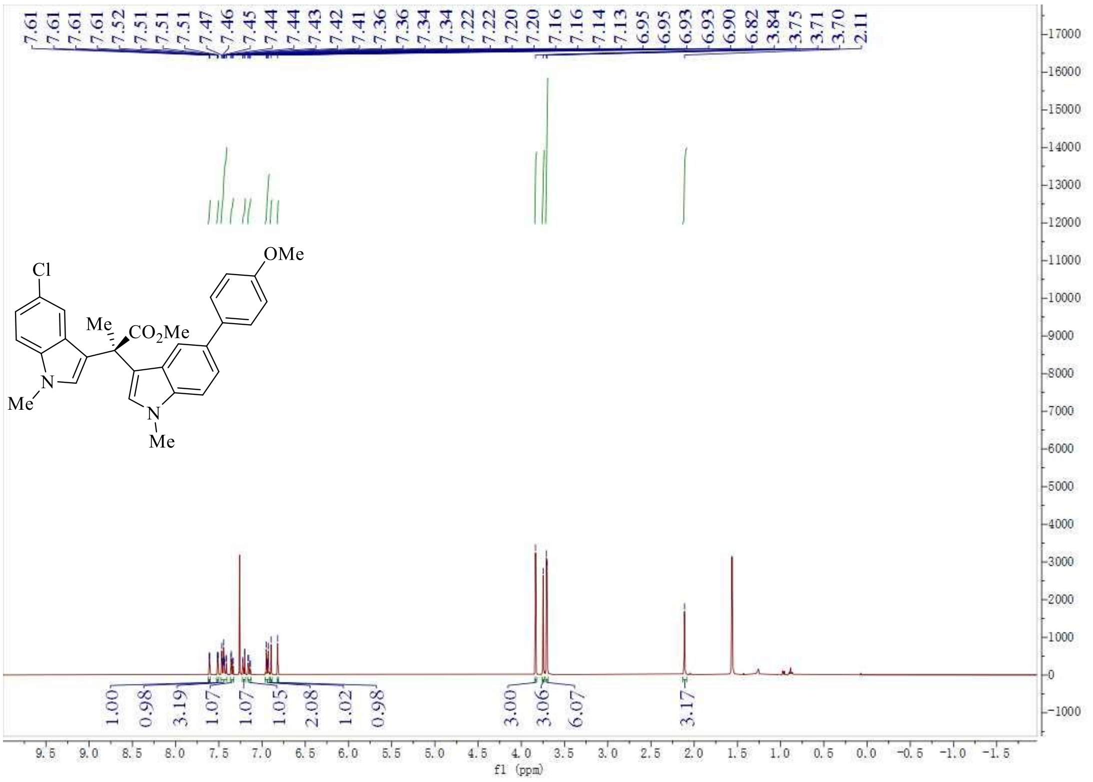

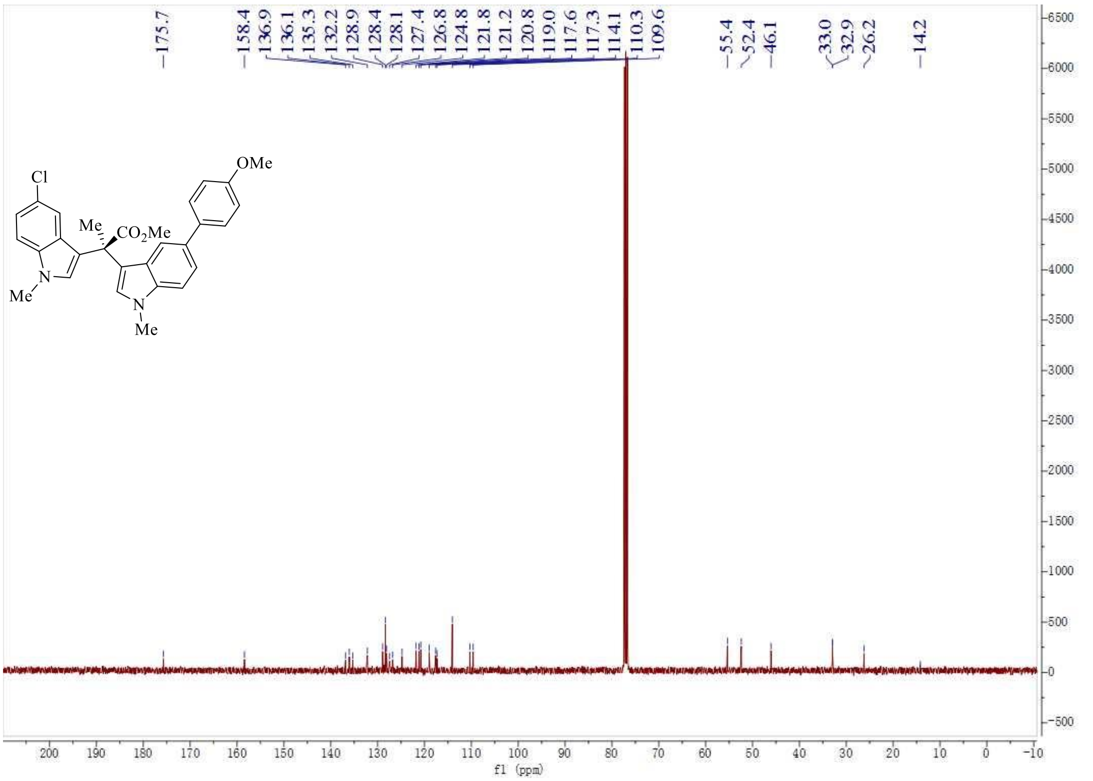

38

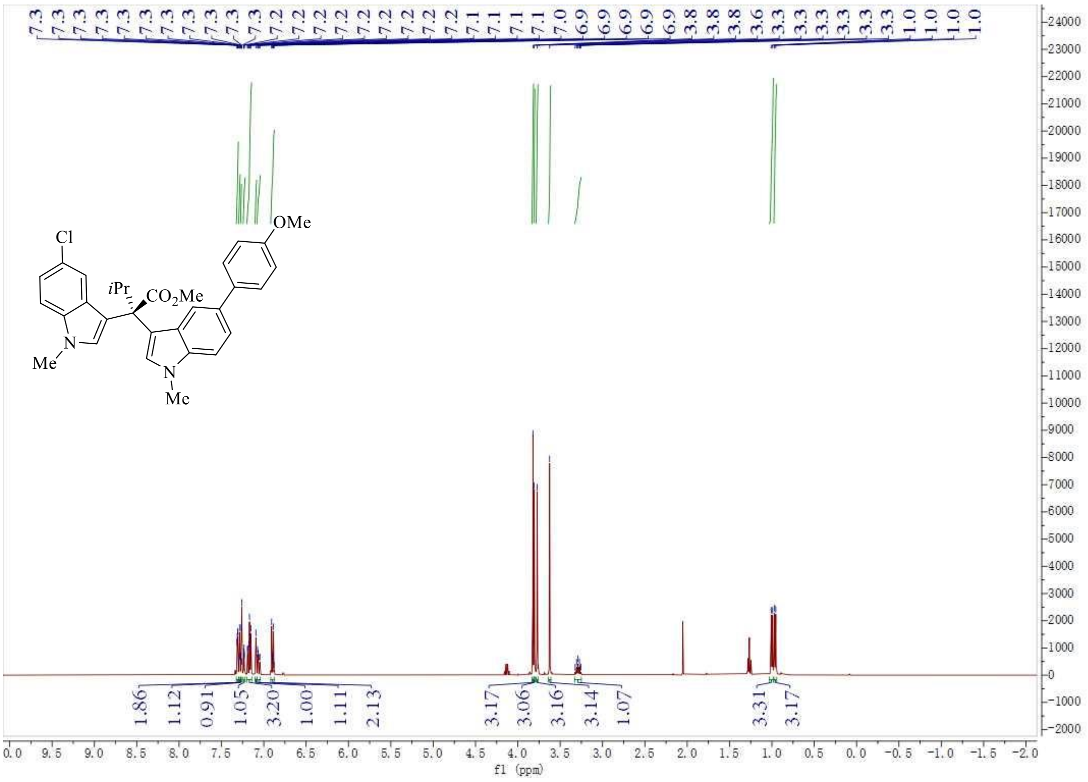
Cl
*i*Pr
CO_2Me
OMe
N
Me
N
Me
1.86 1.12 0.91 1.05 3.20 1.00 1.11 2.13 3.17 3.06 3.16 3.14 1.07 3.31 3.17
f1 (ppm)

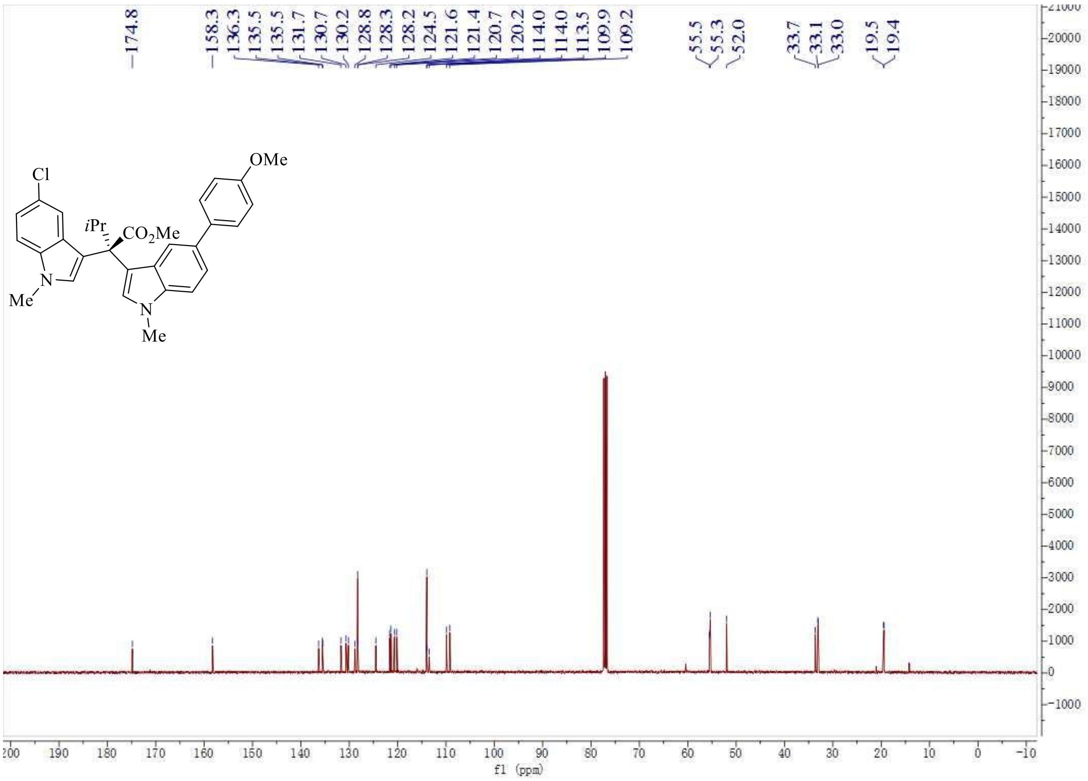
174.8 158.3 136.3 135.5 135.5 131.7 130.7 130.2 128.8 128.3 128.2 124.5 121.6 121.4 120.7 120.2 114.0 114.0 113.5 109.9 109.2
55.5 55.3 52.0 33.7 33.1 33.0 19.5 19.4
Cl
*i*Pr
CO_2Me
OMe
N
Me
N
Me
f1 (ppm)

39

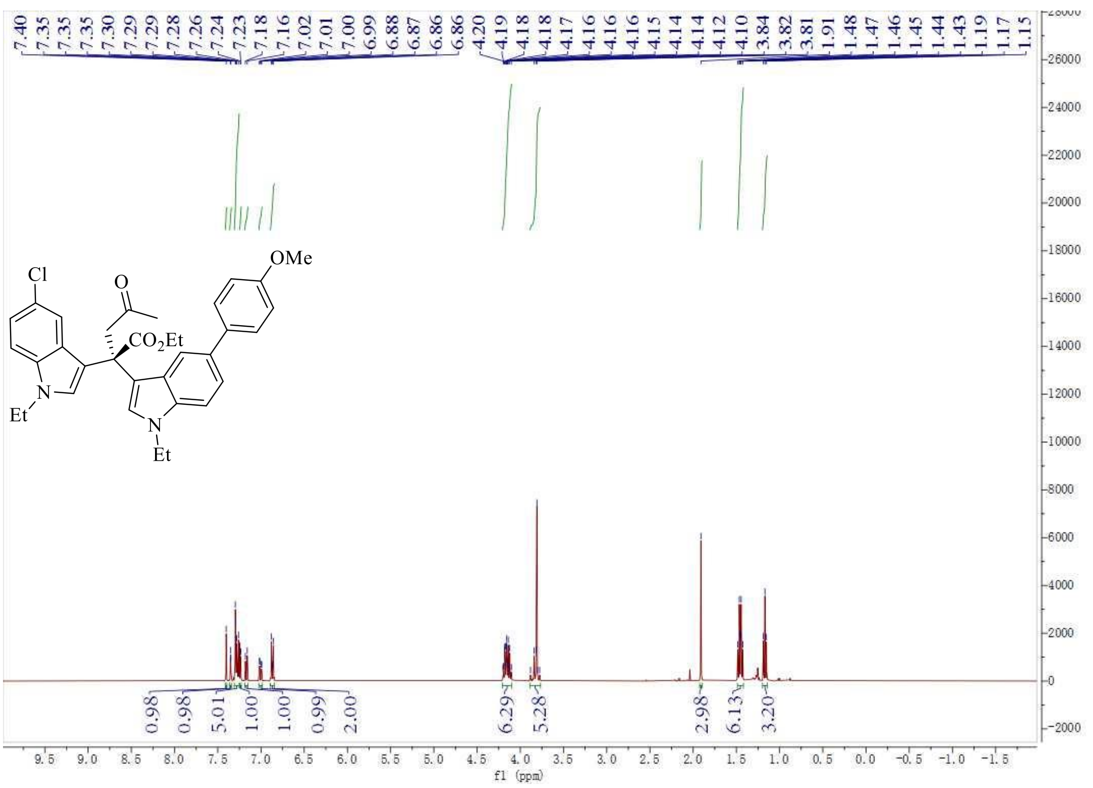

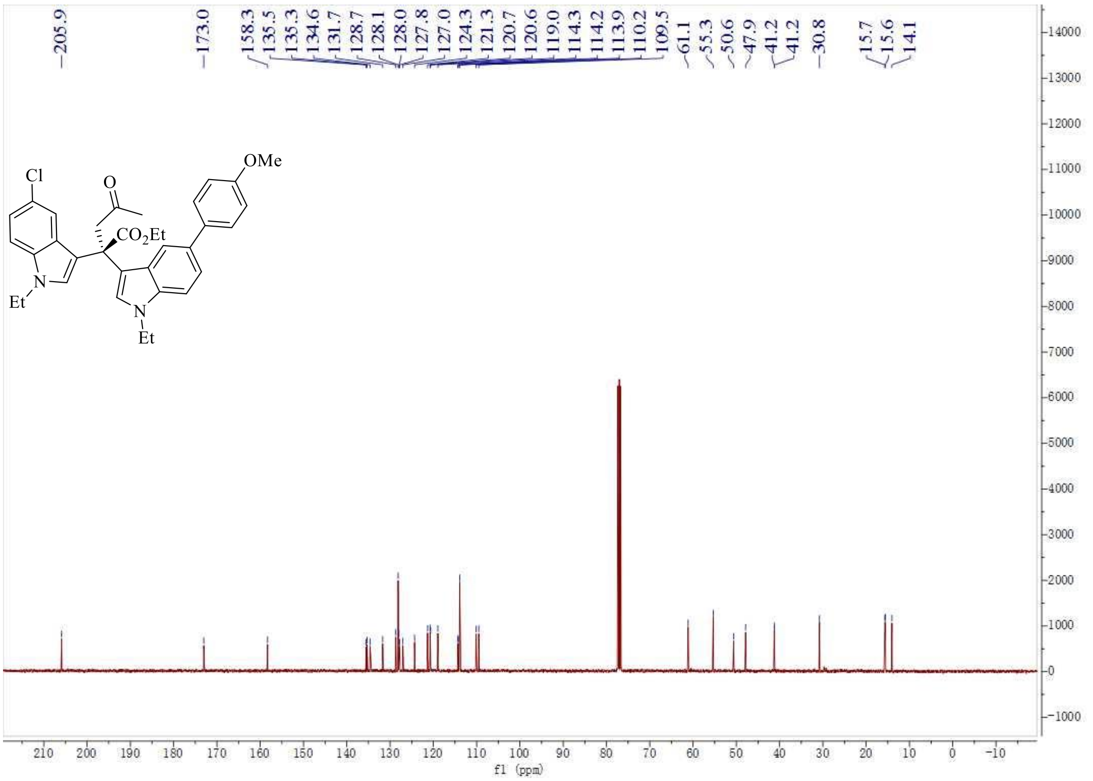

40

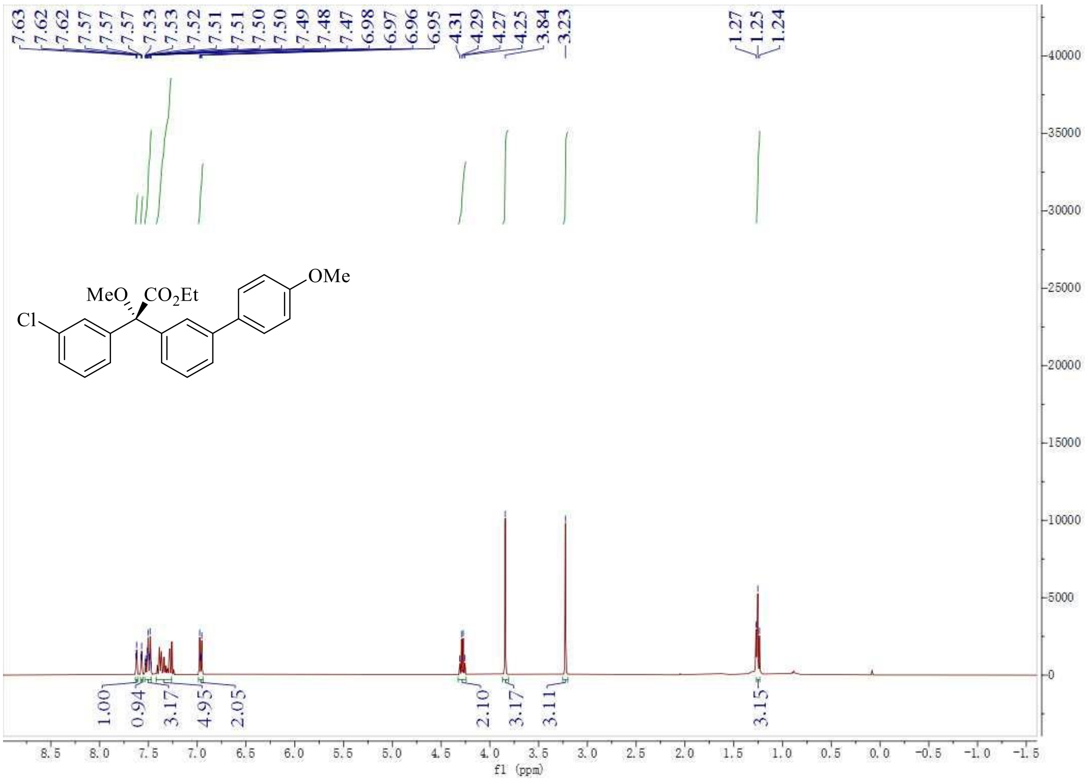

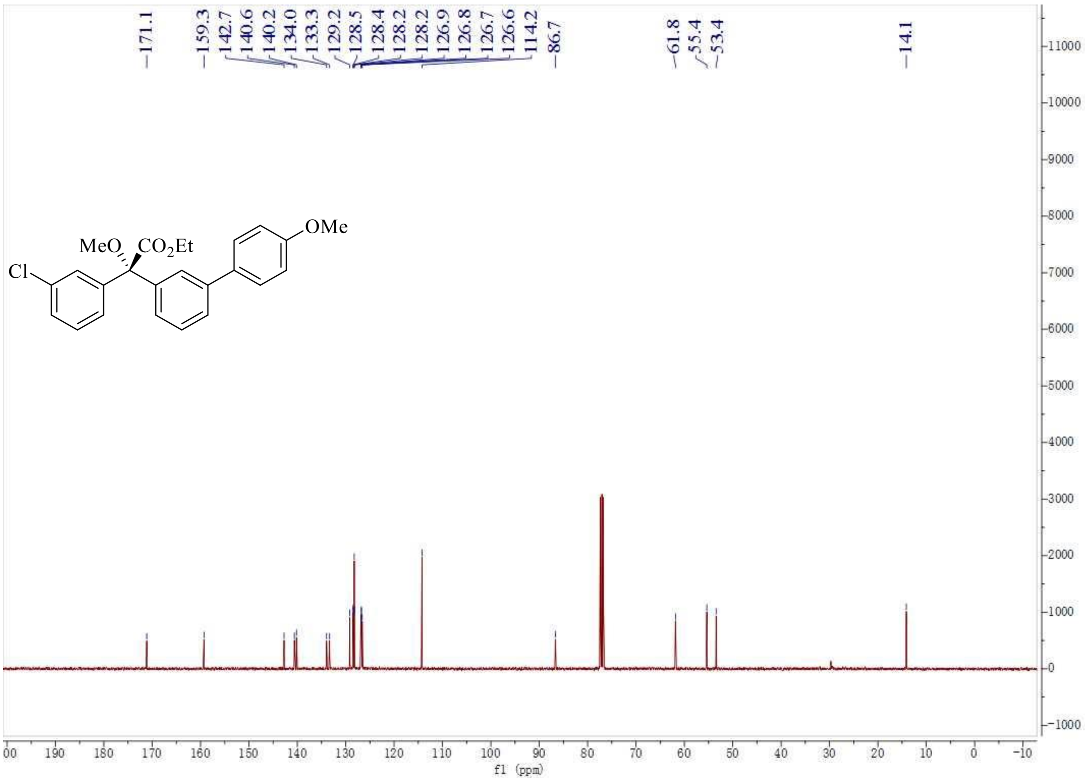

41

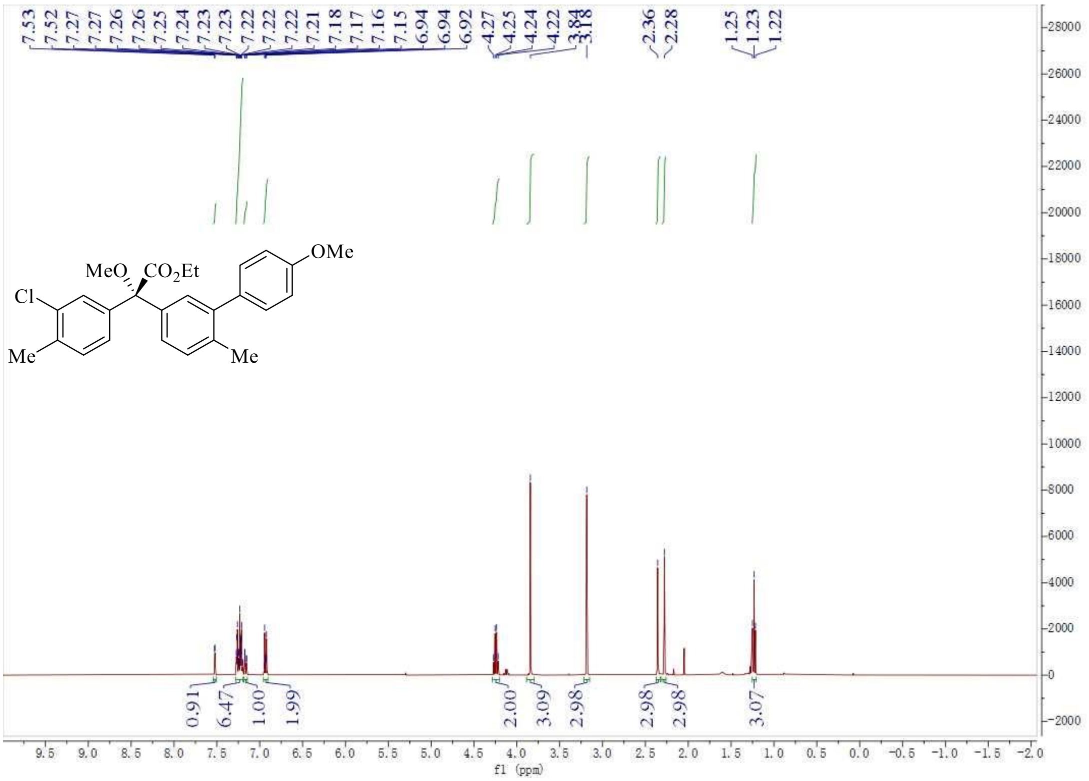

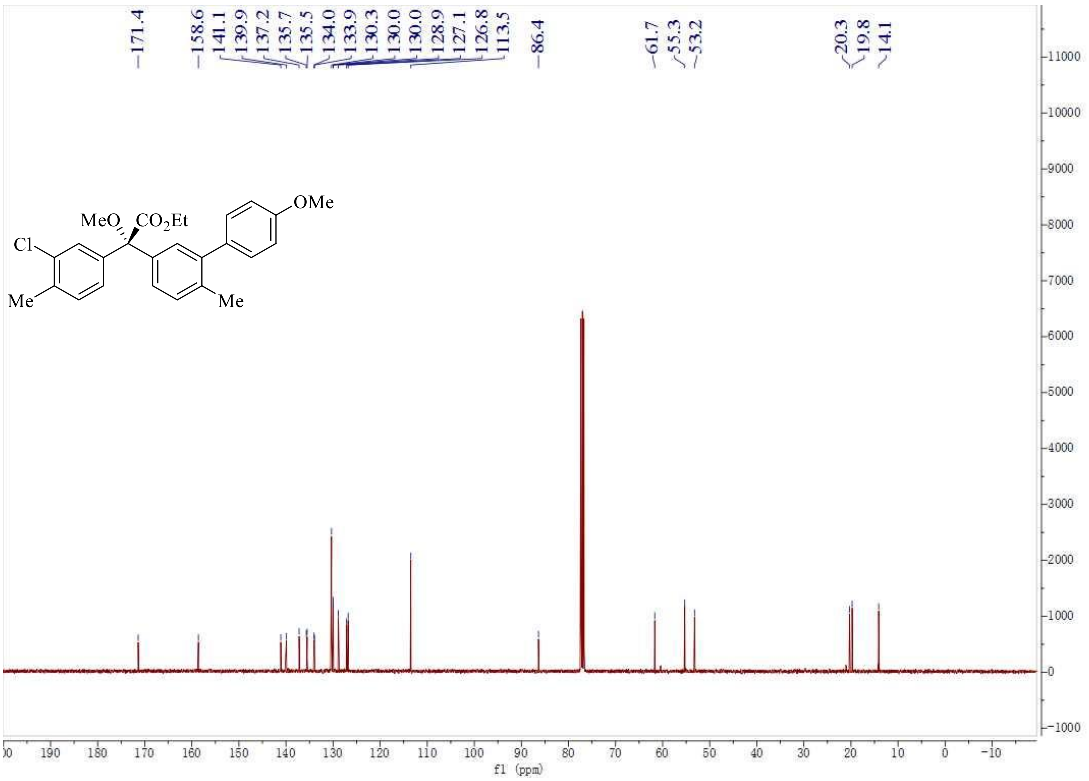

42

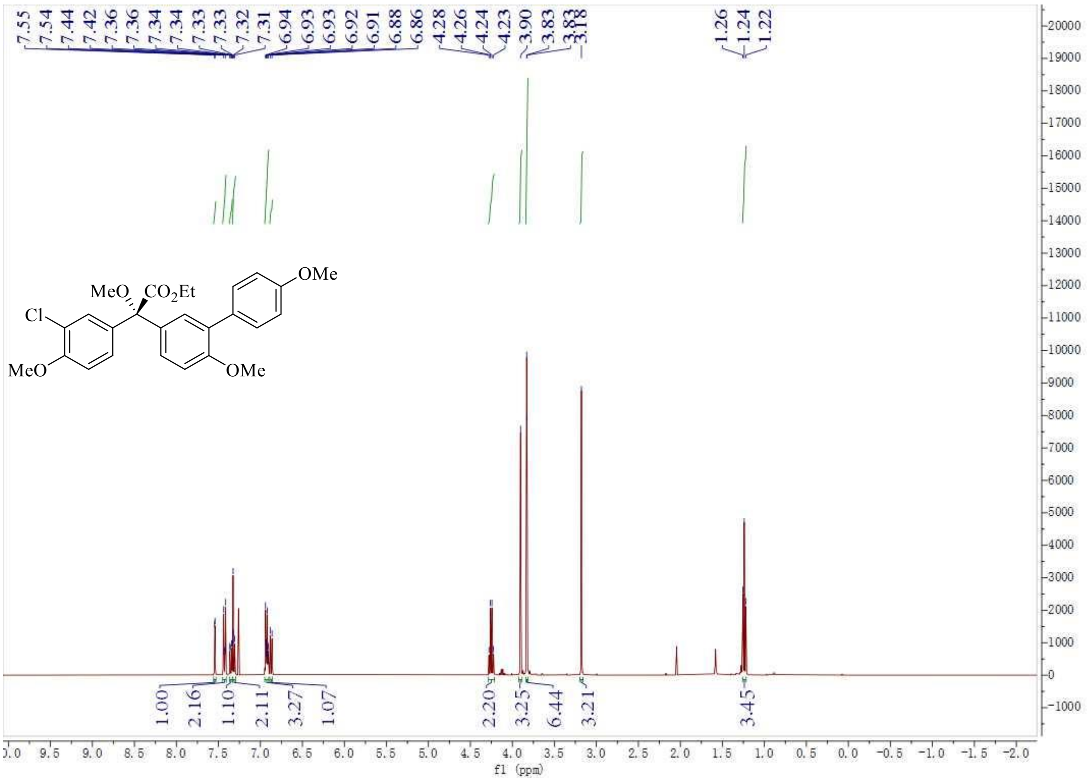

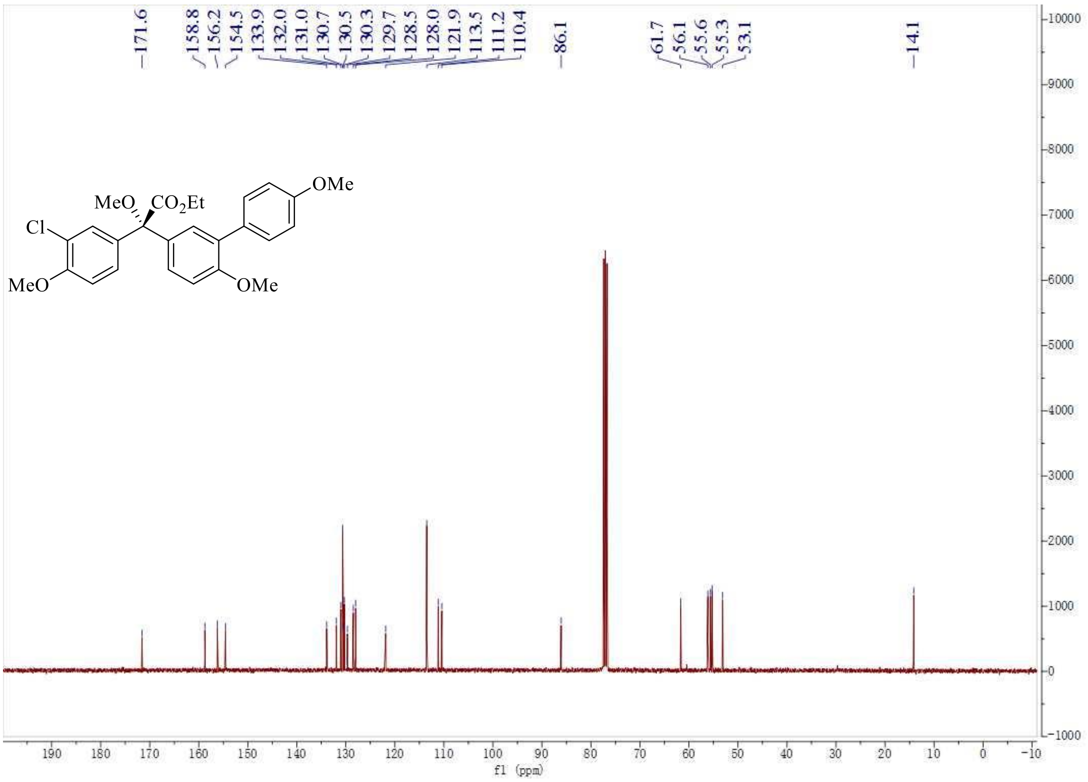

43

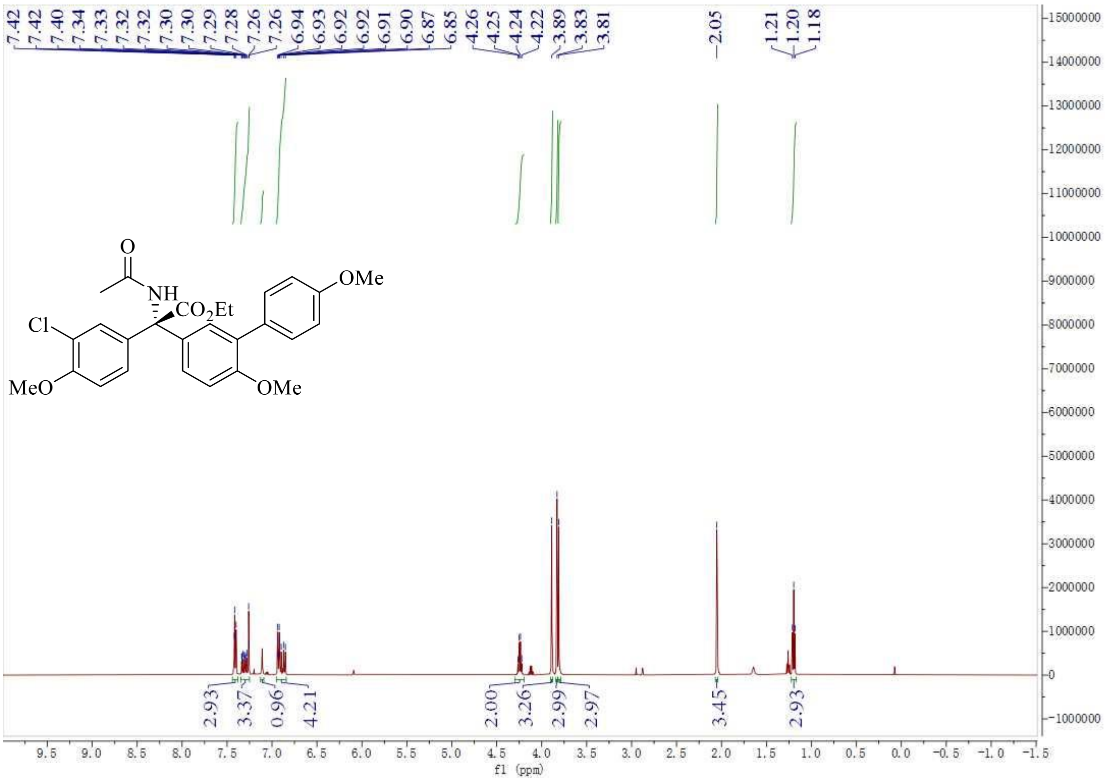

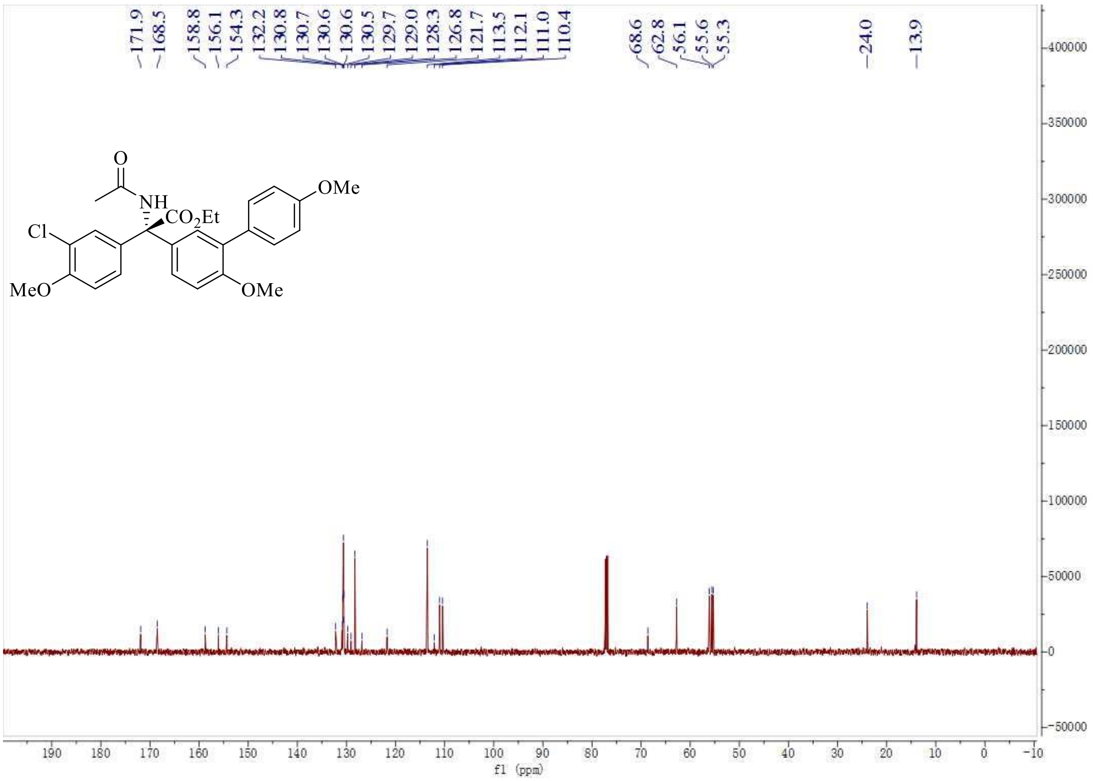

44

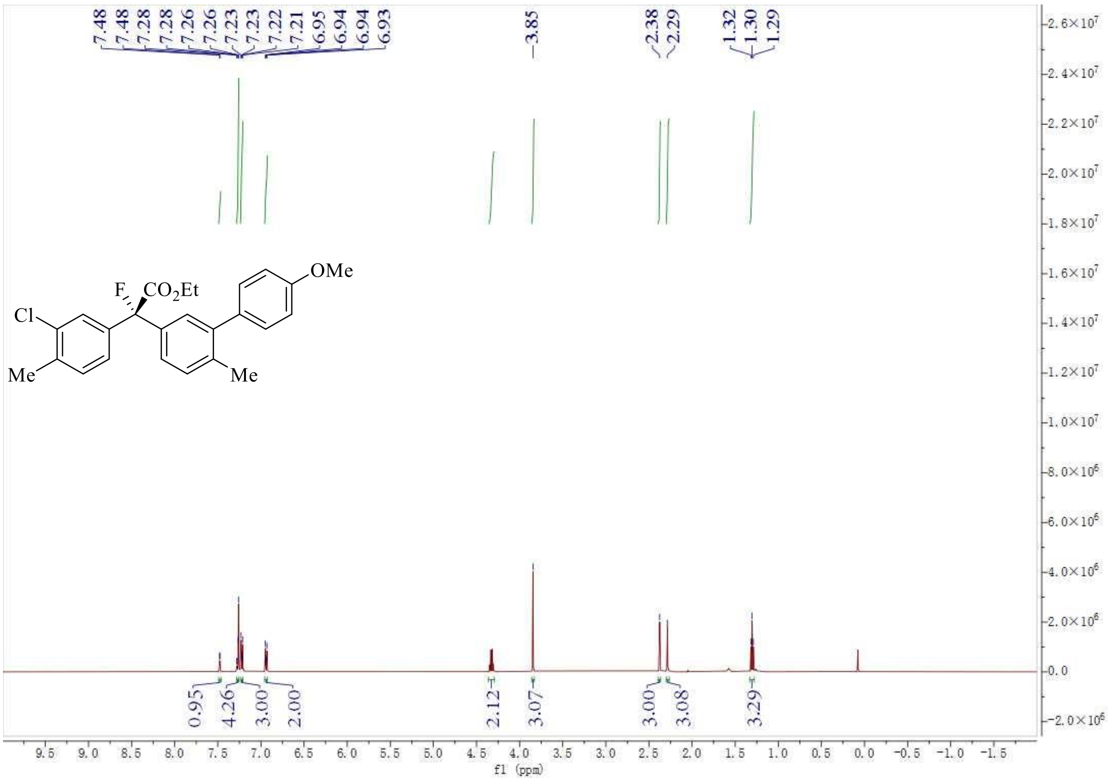

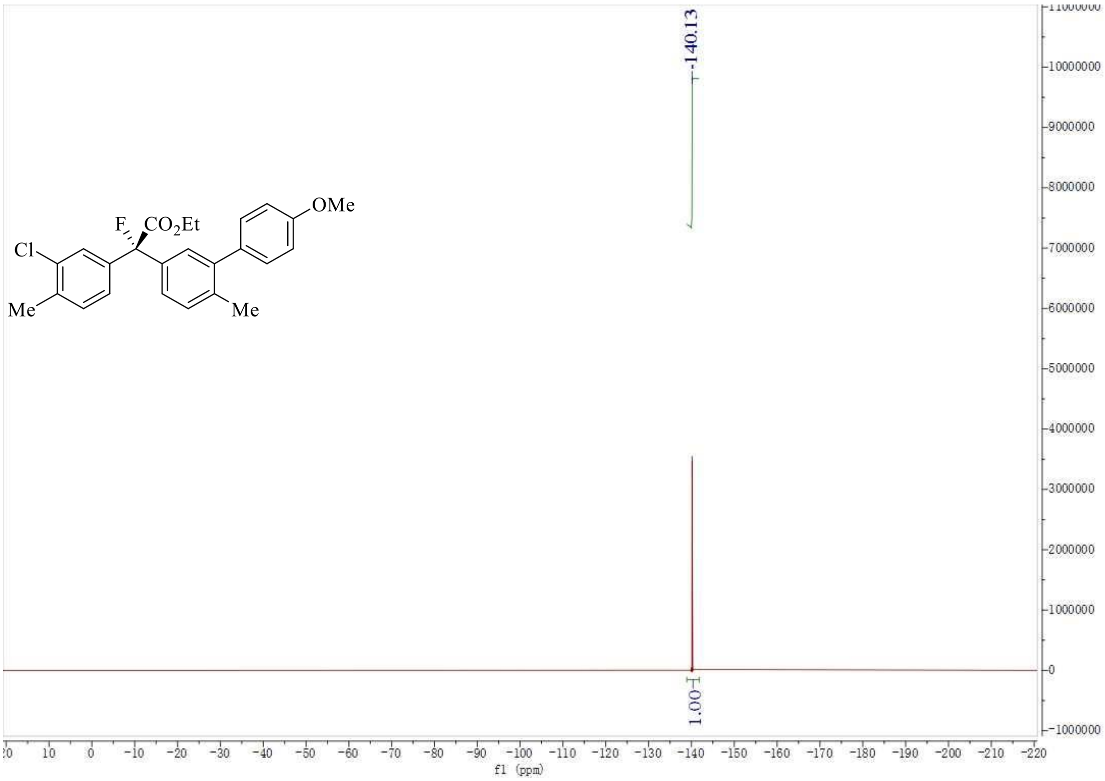

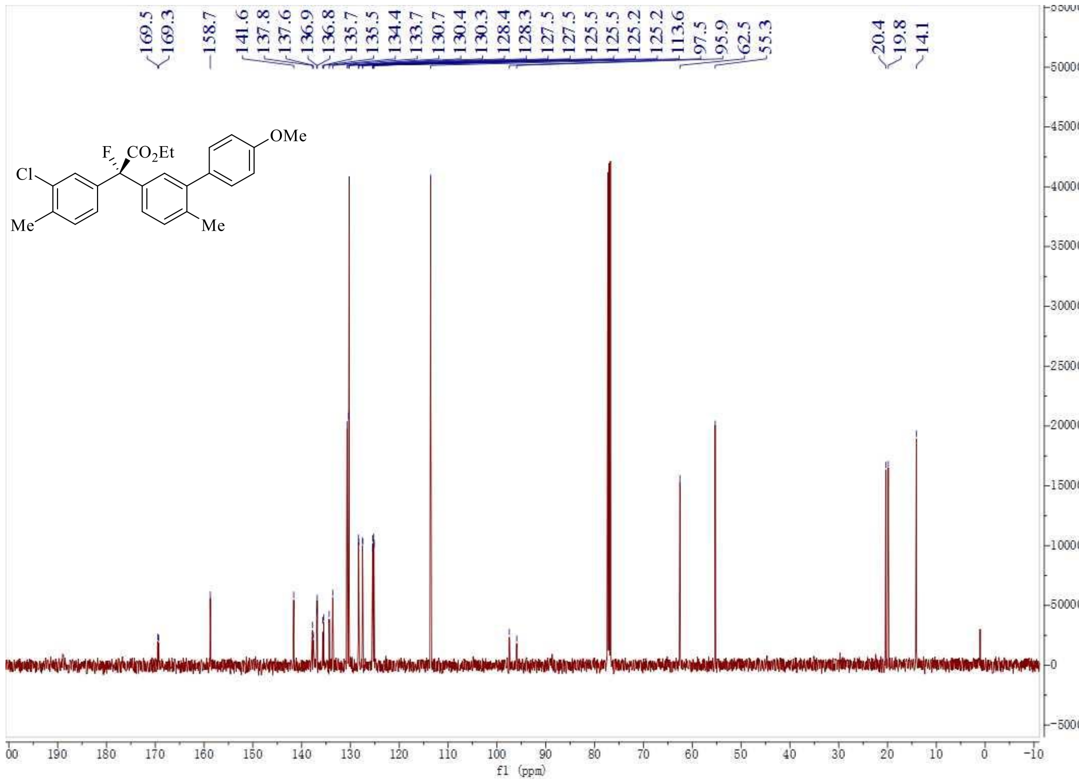
169.5
169.3
158.7
141.6
137.8
137.6
136.9
136.8
135.7
135.5
134.4
133.7
130.7
130.4
130.3
128.4
128.3
127.5
127.5
125.5
125.5
125.2
125.2
113.6
97.5
95.9
62.5
55.3
20.4
19.8
14.1
F
CO2Et
OMe
Cl
Me
Me
f1 (ppm)

45

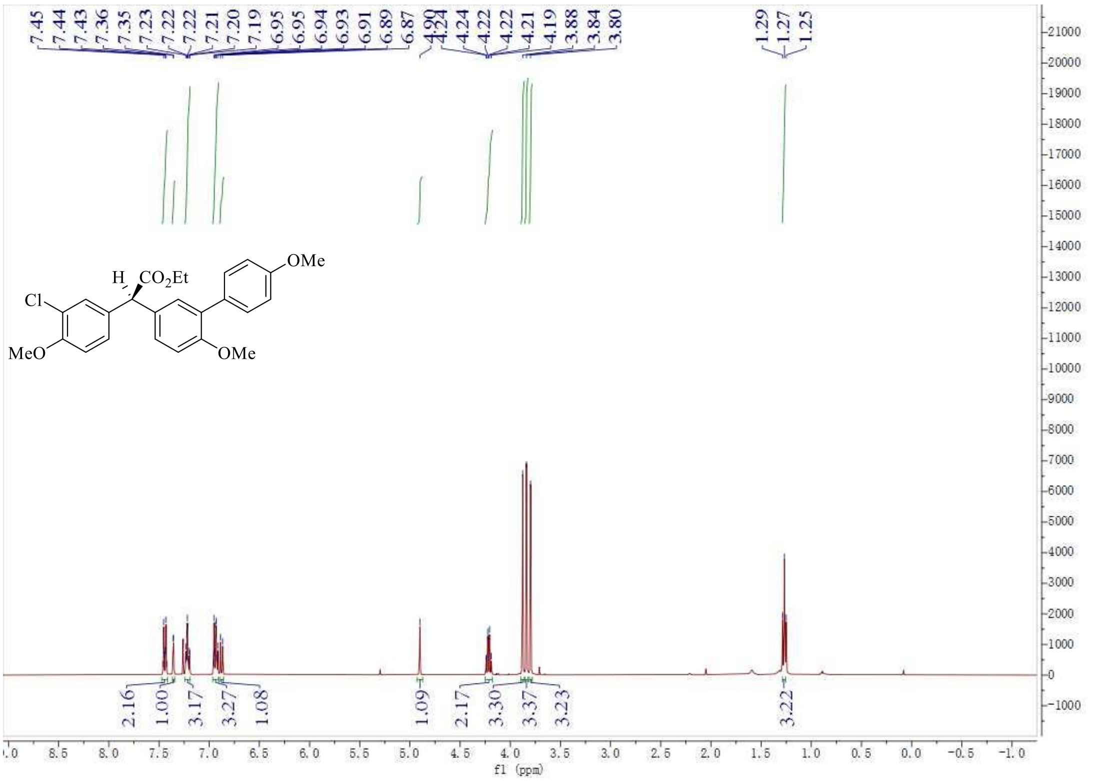

7.45 7.44 7.43 7.36 7.35 7.23 7.22 7.22 7.21 7.20 7.19 6.95 6.95 6.94 6.93 6.91 6.89 6.87 4.90 4.24 4.24 4.22 4.22 4.21 4.19 3.88 3.84 3.80
1.29 1.27 1.25
H
CO2Et
Cl
MeO
OMe
OMe
2.16 1.00 3.17 3.27 1.08 1.09 2.17 3.30 3.37 3.23 3.22
f1 (ppm)

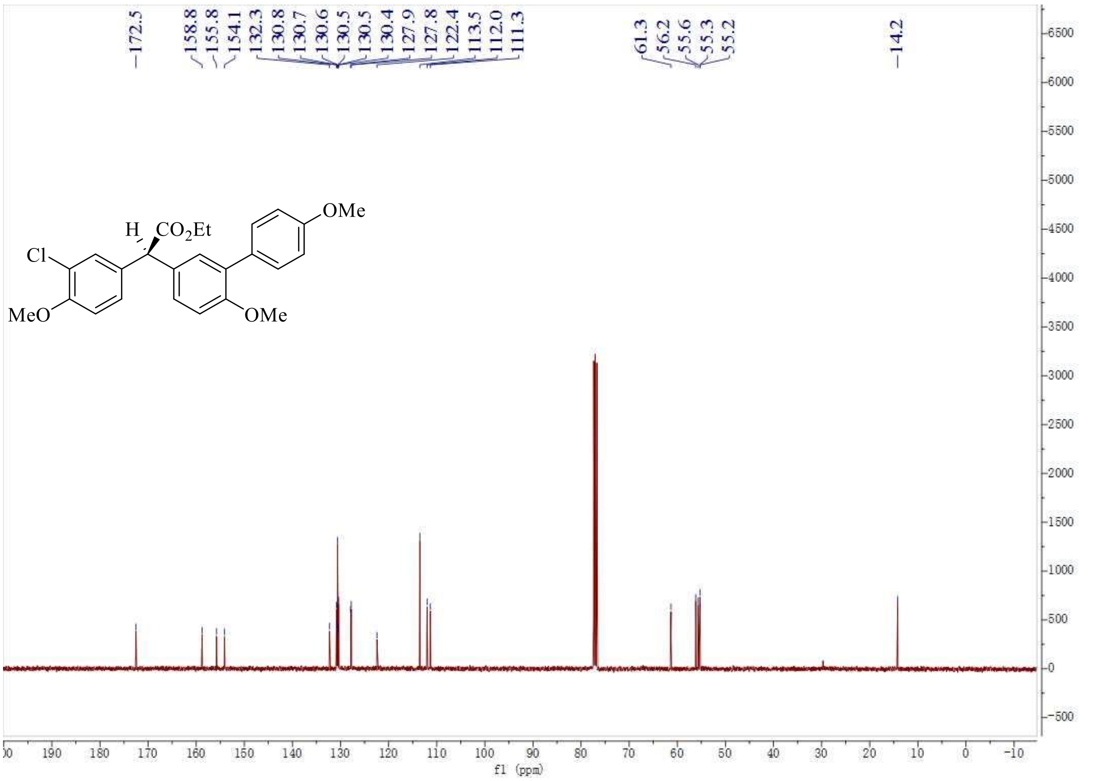

172.5
158.8 155.8 154.1 132.3 130.8 130.7 130.6 130.5 130.5 130.4 127.9 127.8 122.4 113.5 112.0 111.3
61.3 56.2 55.6 55.3 55.2
14.2
H
CO2Et
Cl
MeO
OMe
OMe
f1 (ppm)

46

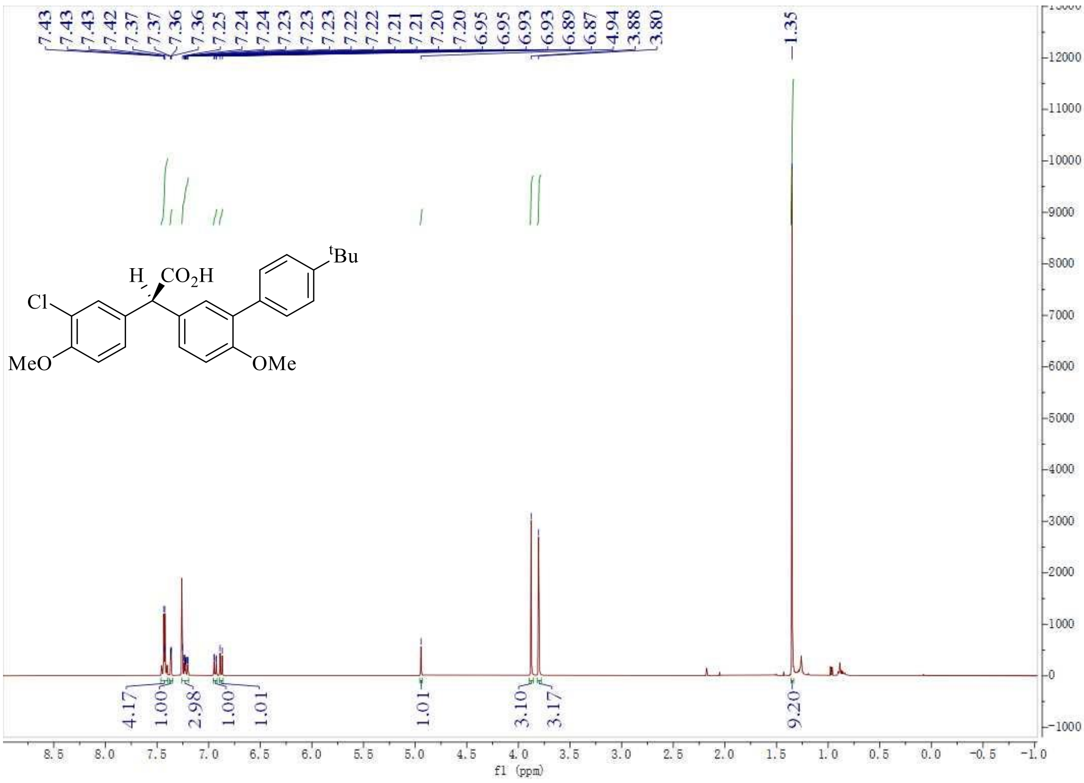

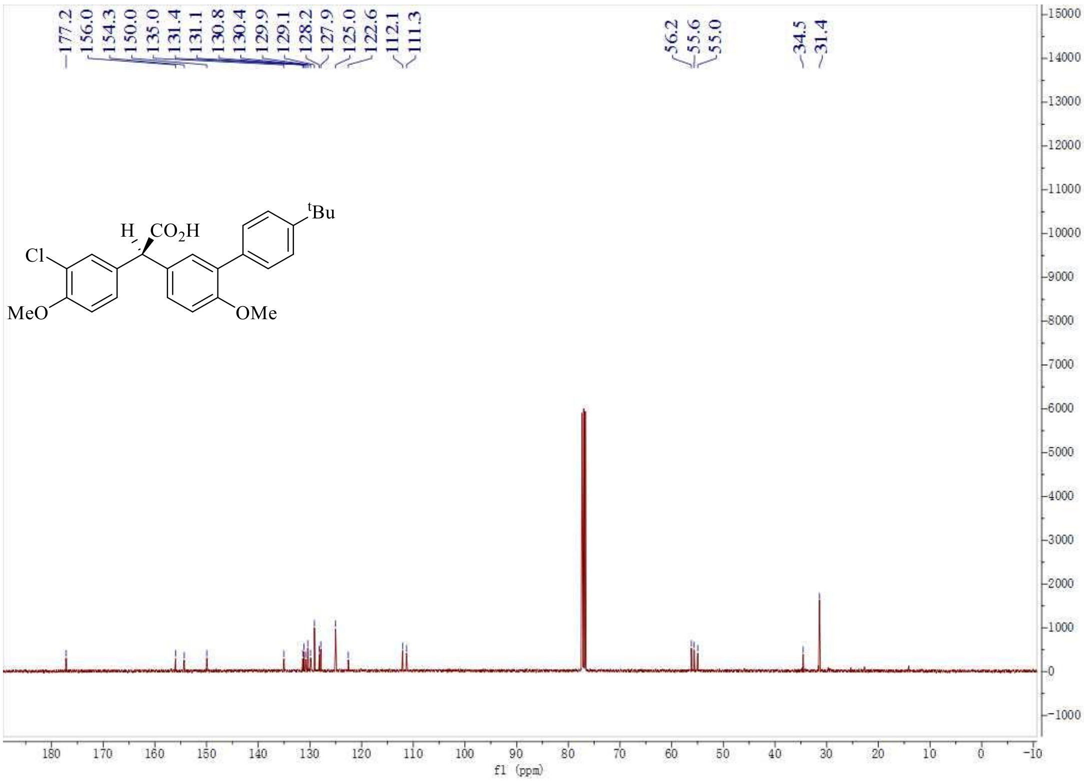

47

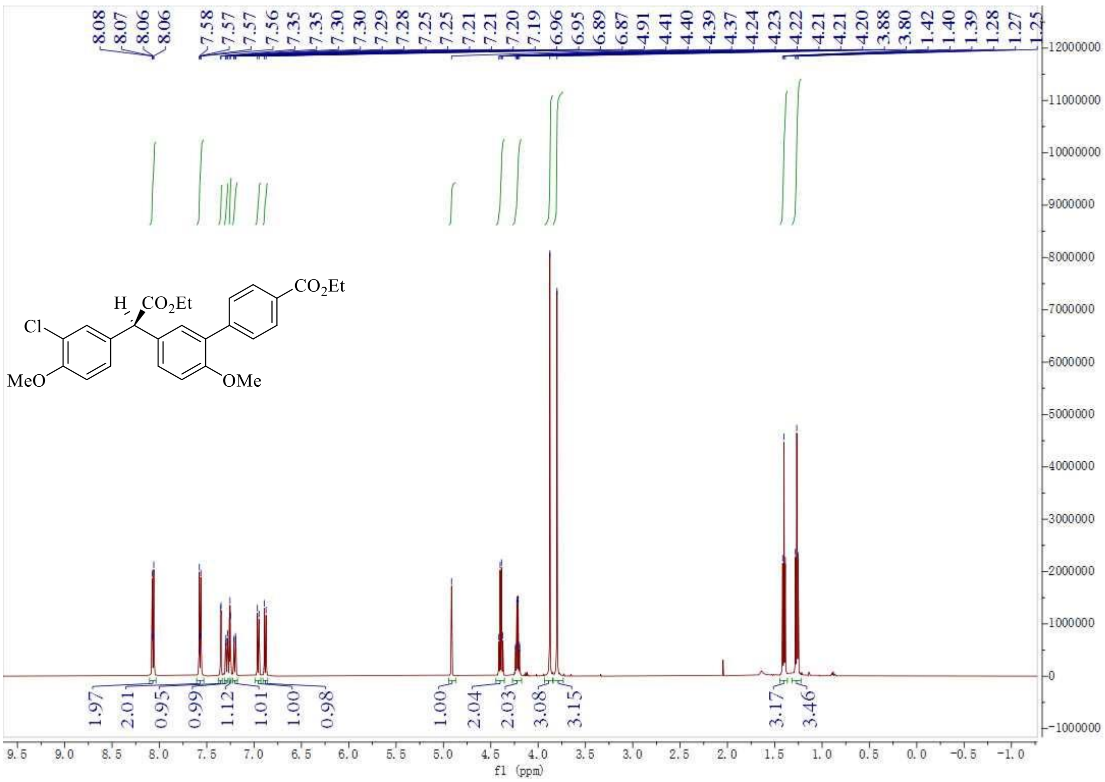

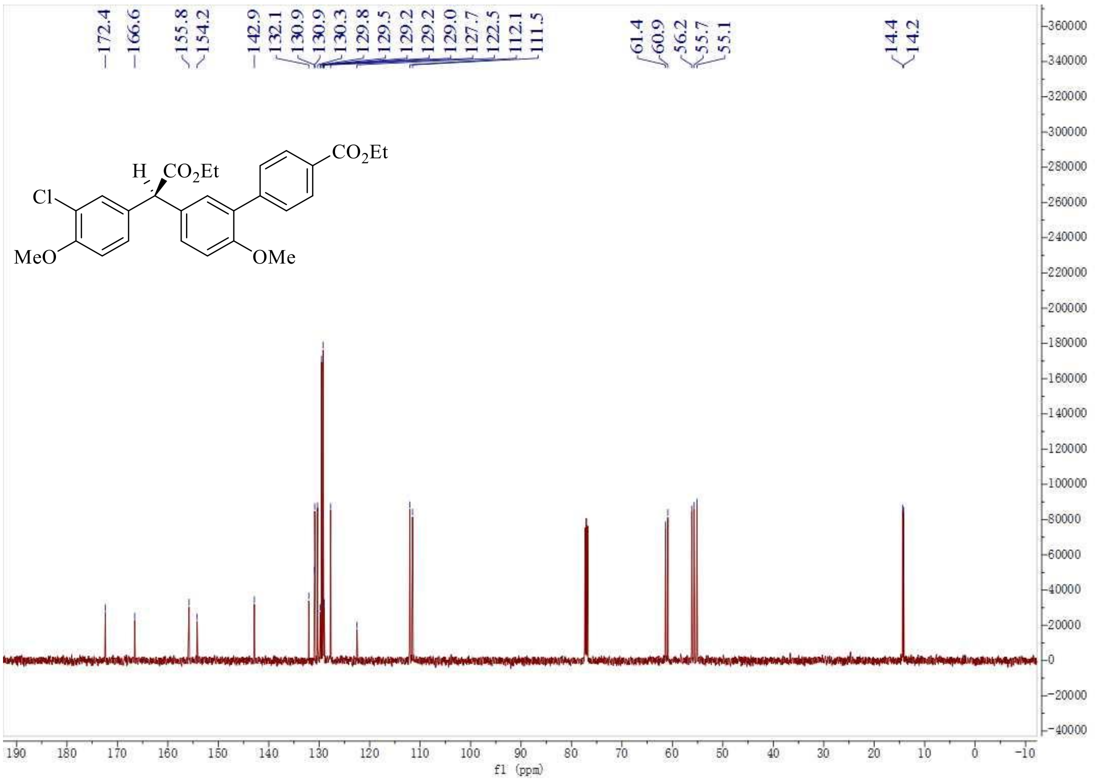

48

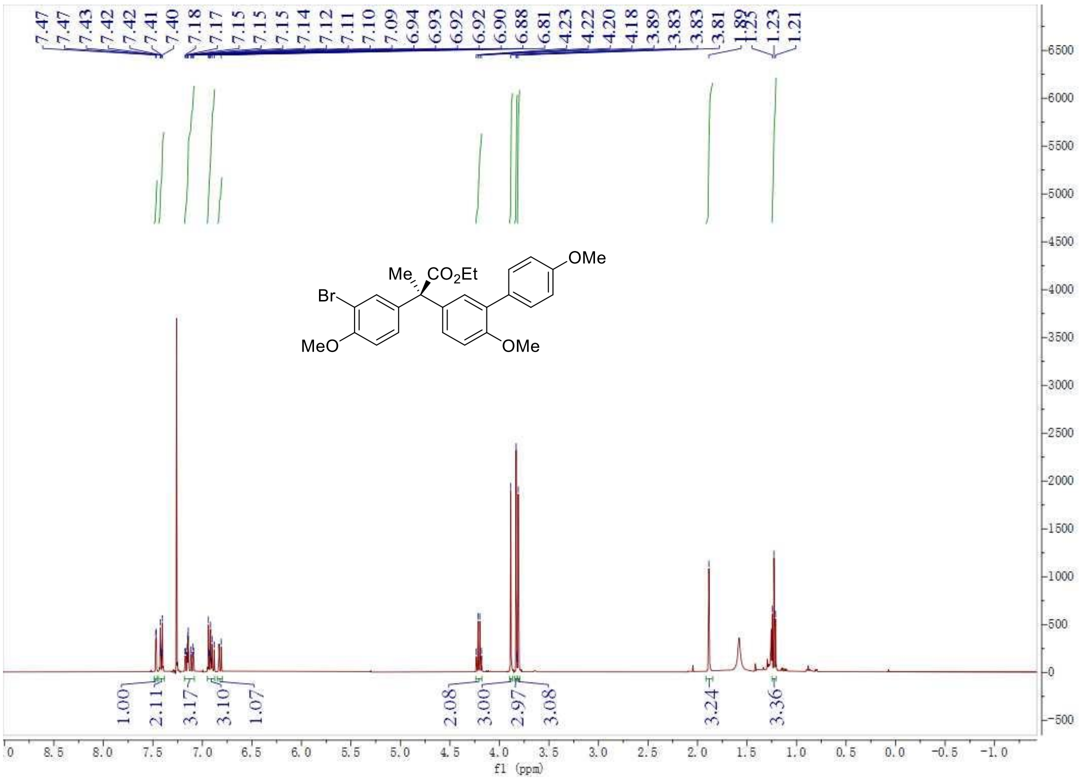

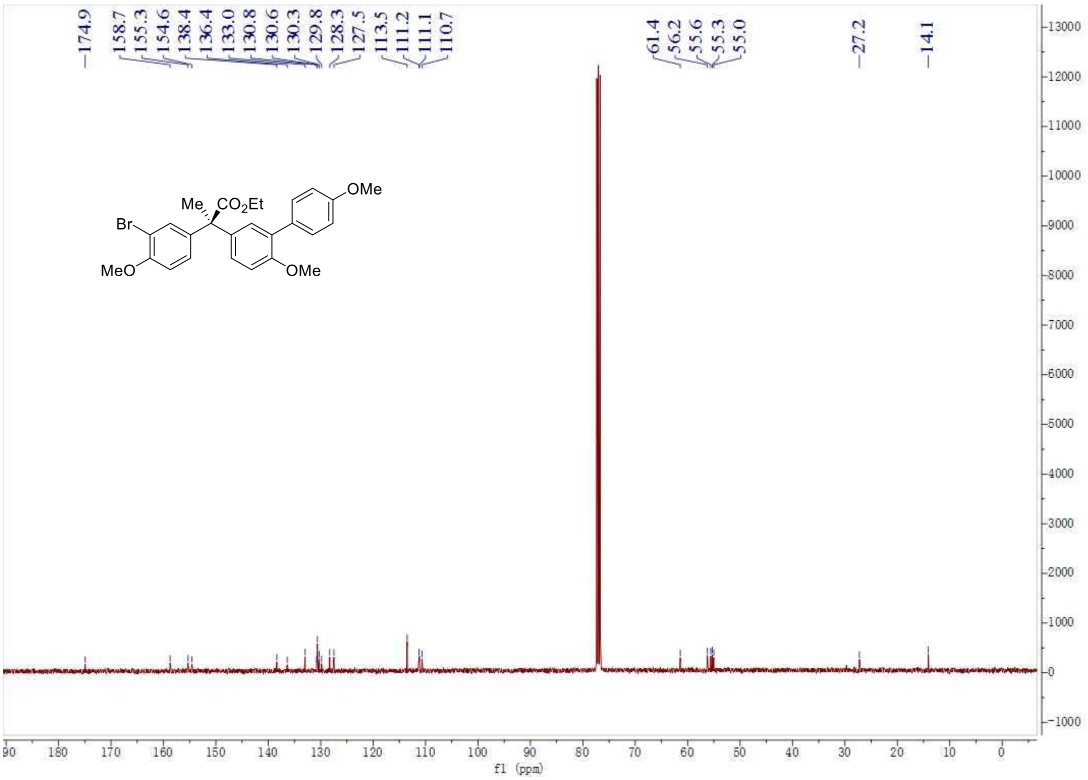

49

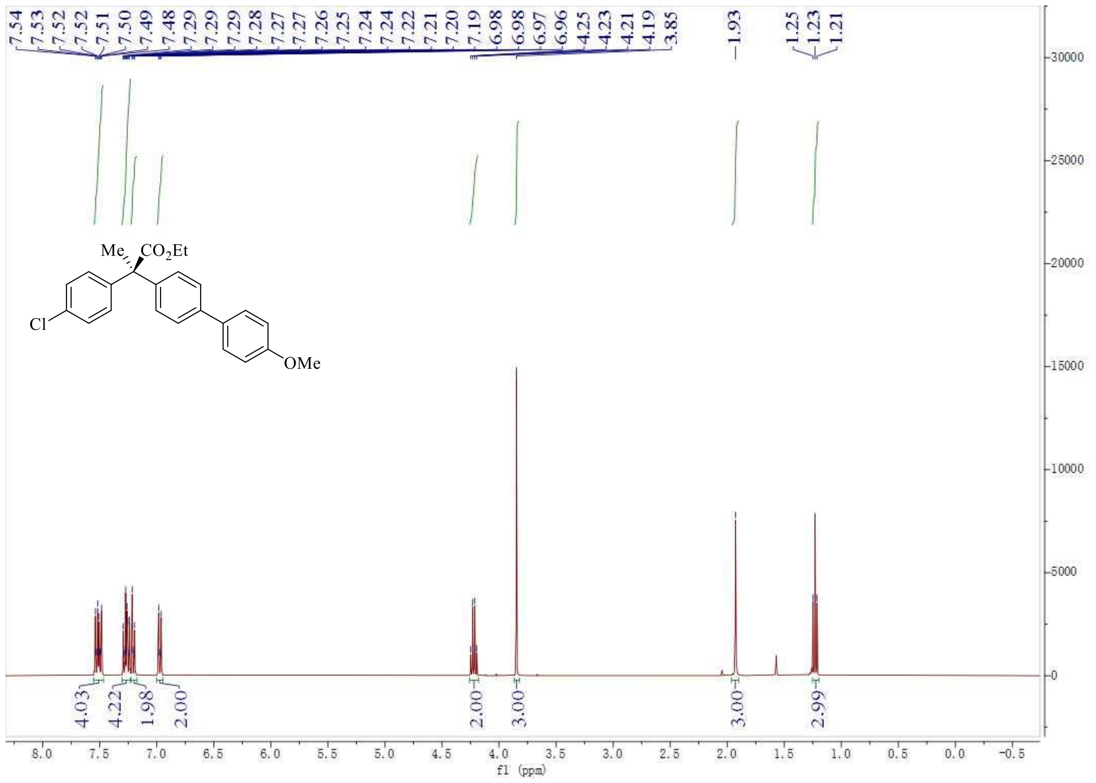

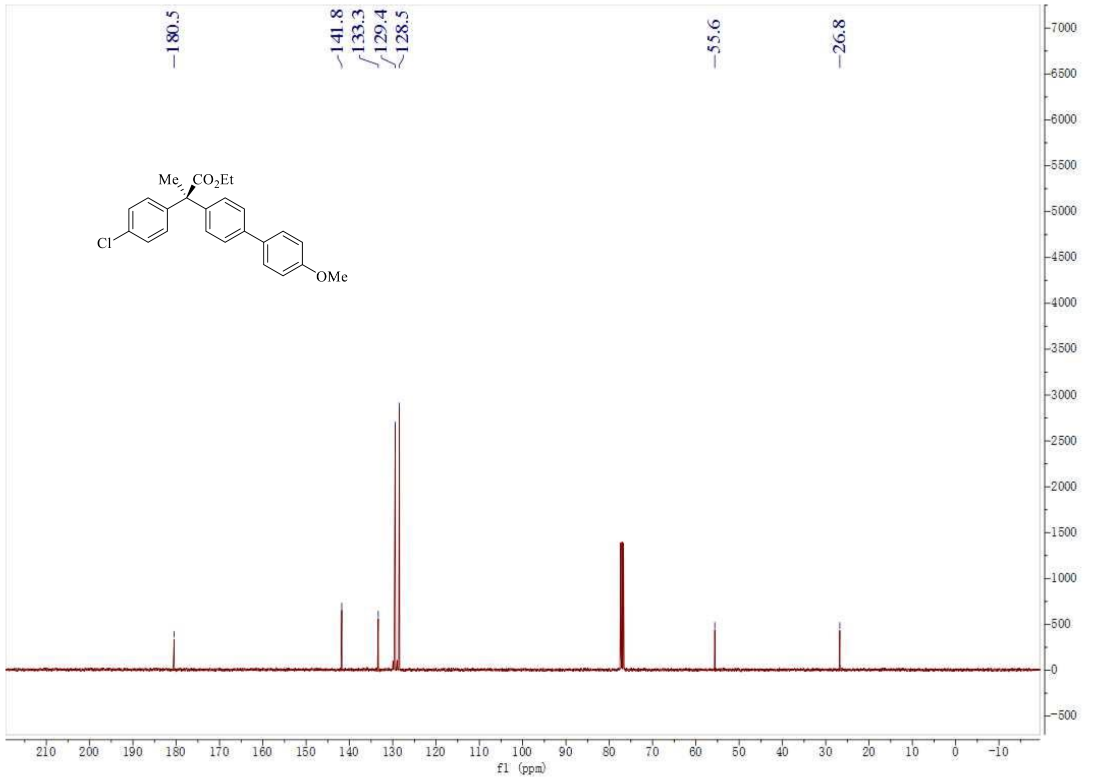

50

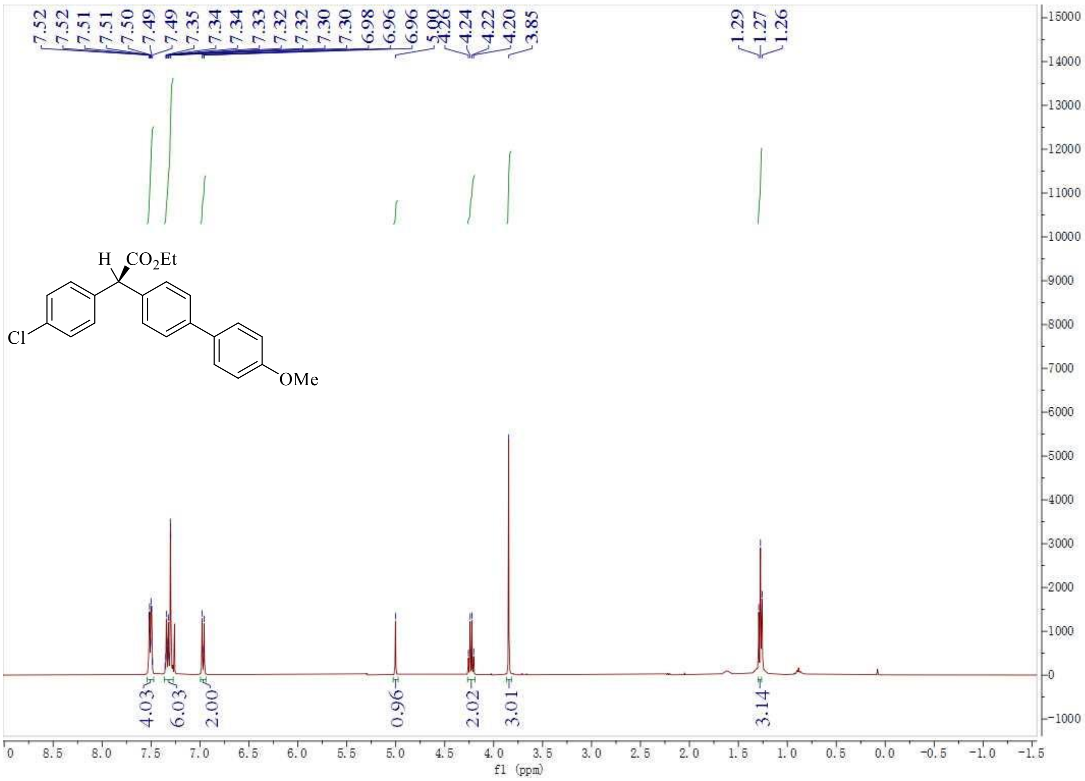

7.52
7.52
7.51
7.51
7.50
7.49
7.49
7.35
7.34
7.34
7.33
7.32
7.32
7.30
7.30
6.98
6.96
5.00
4.26
4.24
4.22
4.20
3.85
1.29
1.27
1.26
H
CO2Et
Cl
OMe
4.03
6.03
2.00
0.96
2.02
3.01
3.14
f1 (ppm)

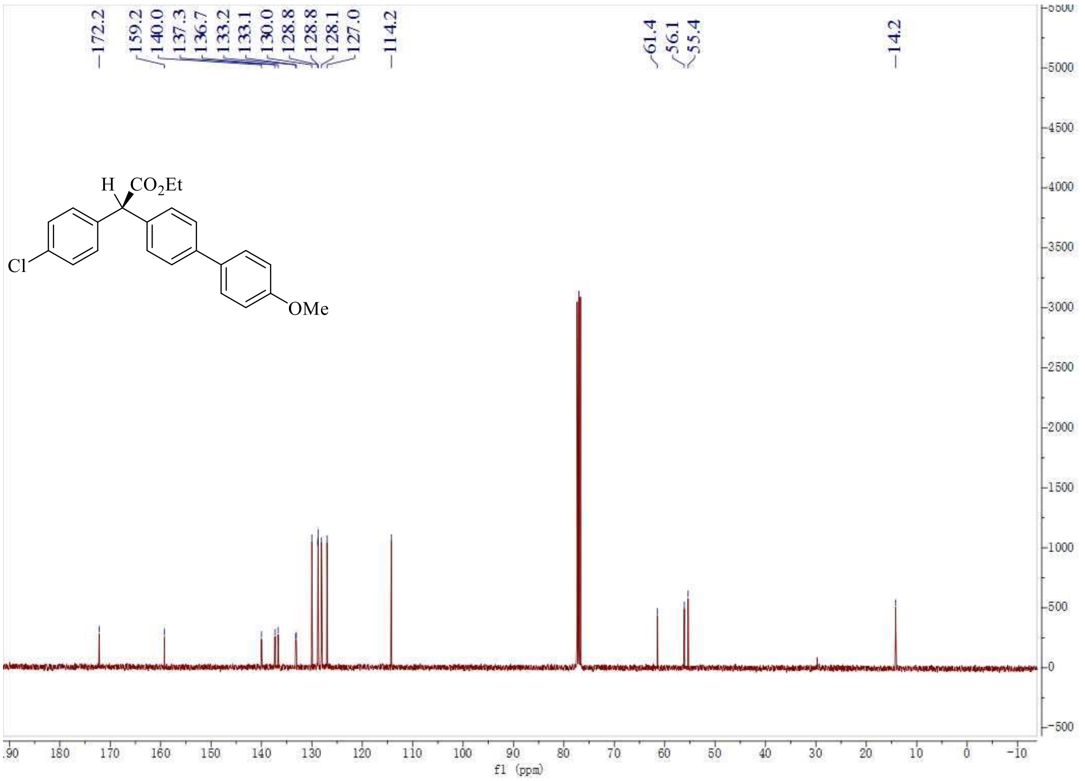

172.2
159.2
140.0
137.3
136.7
133.2
133.1
130.0
128.8
128.8
128.1
127.0
114.2
61.4
56.1
55.4
14.2
H
CO2Et
Cl
OMe
f1 (ppm)

51 (racemic)

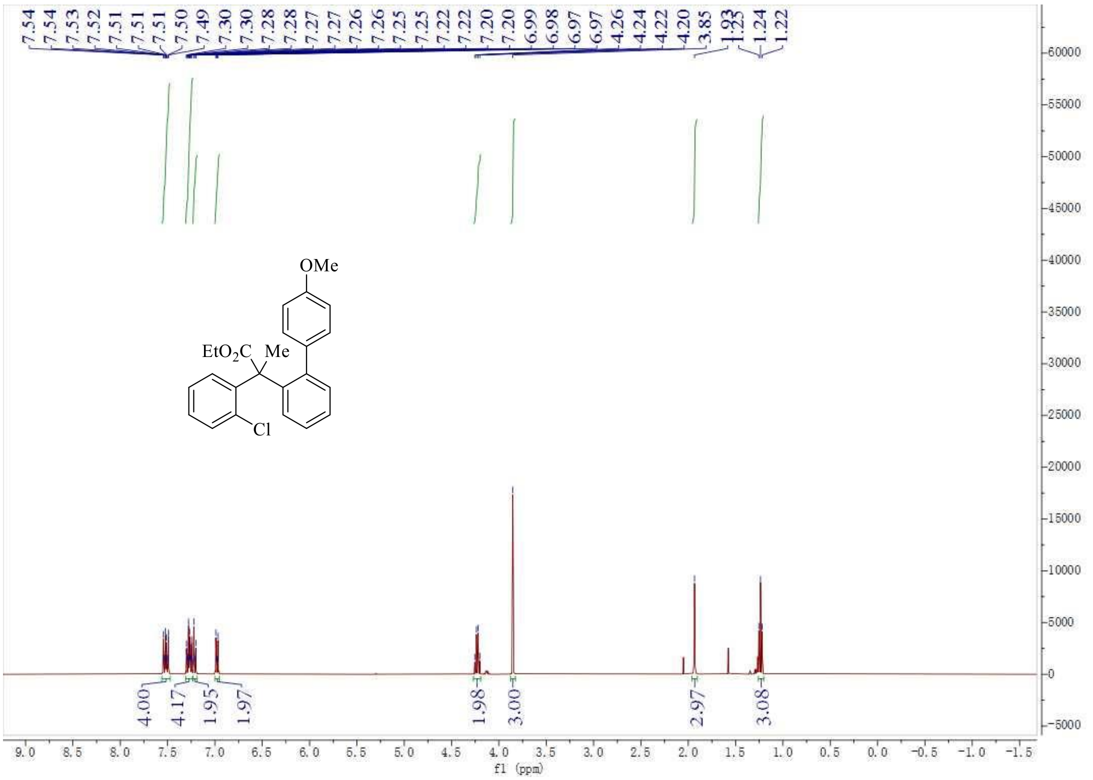

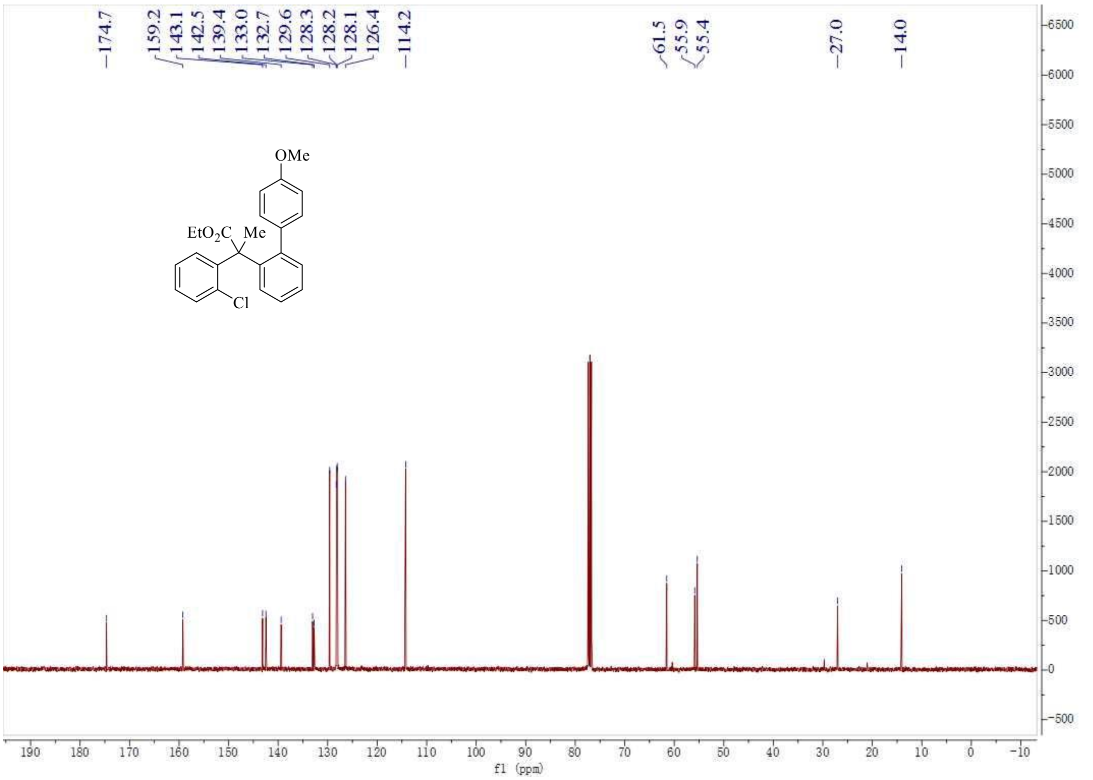

52

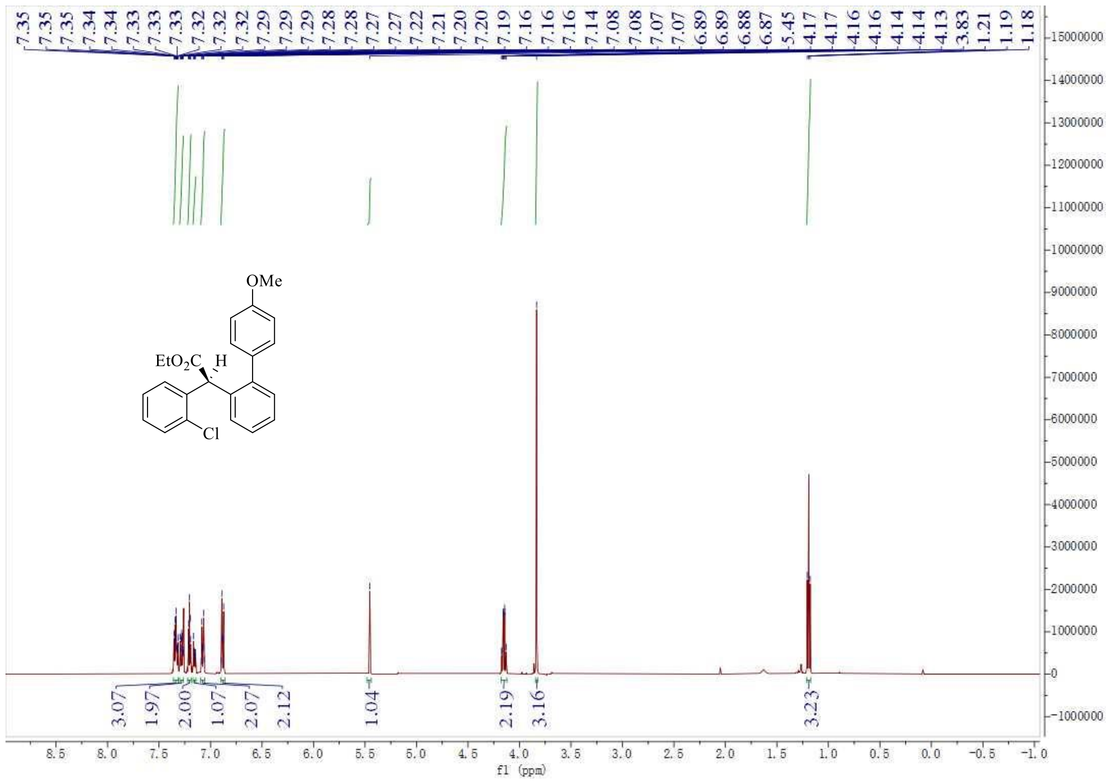

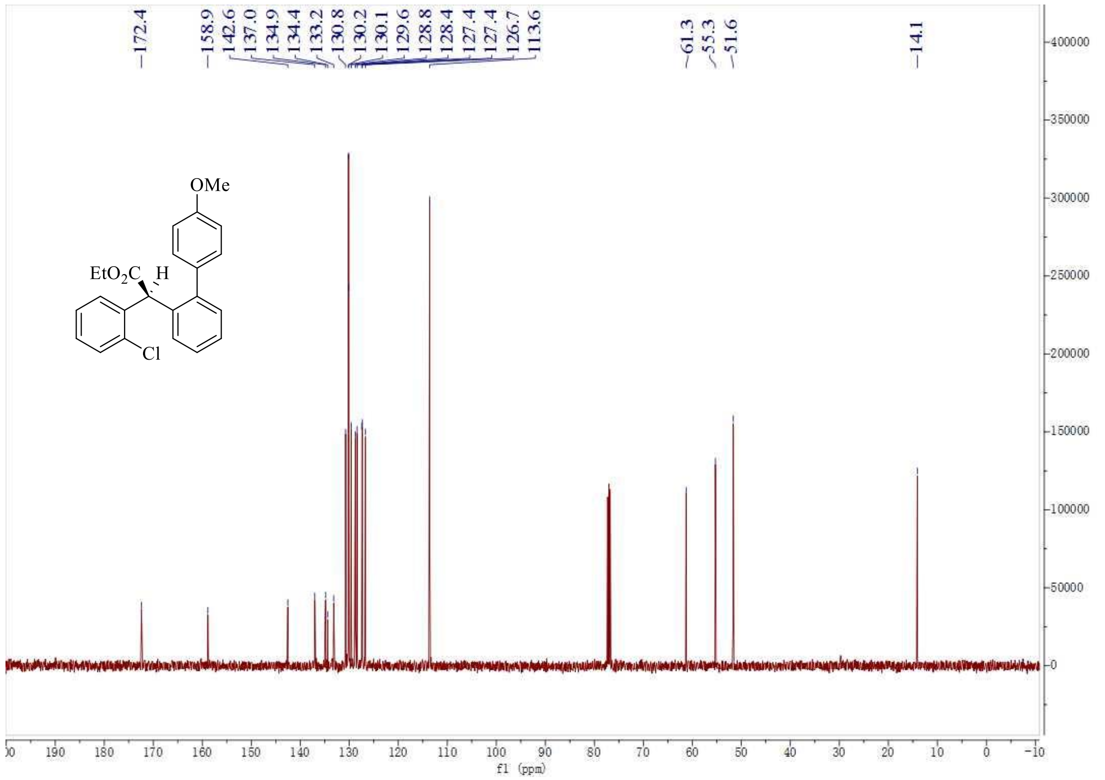

53

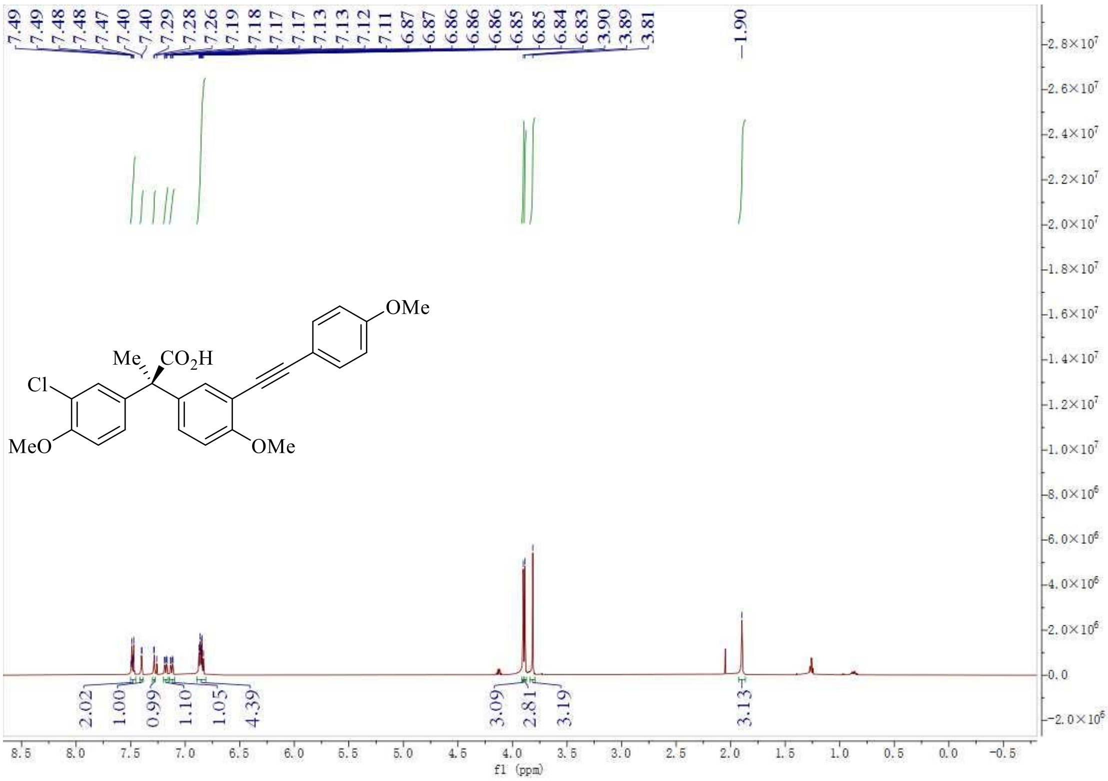

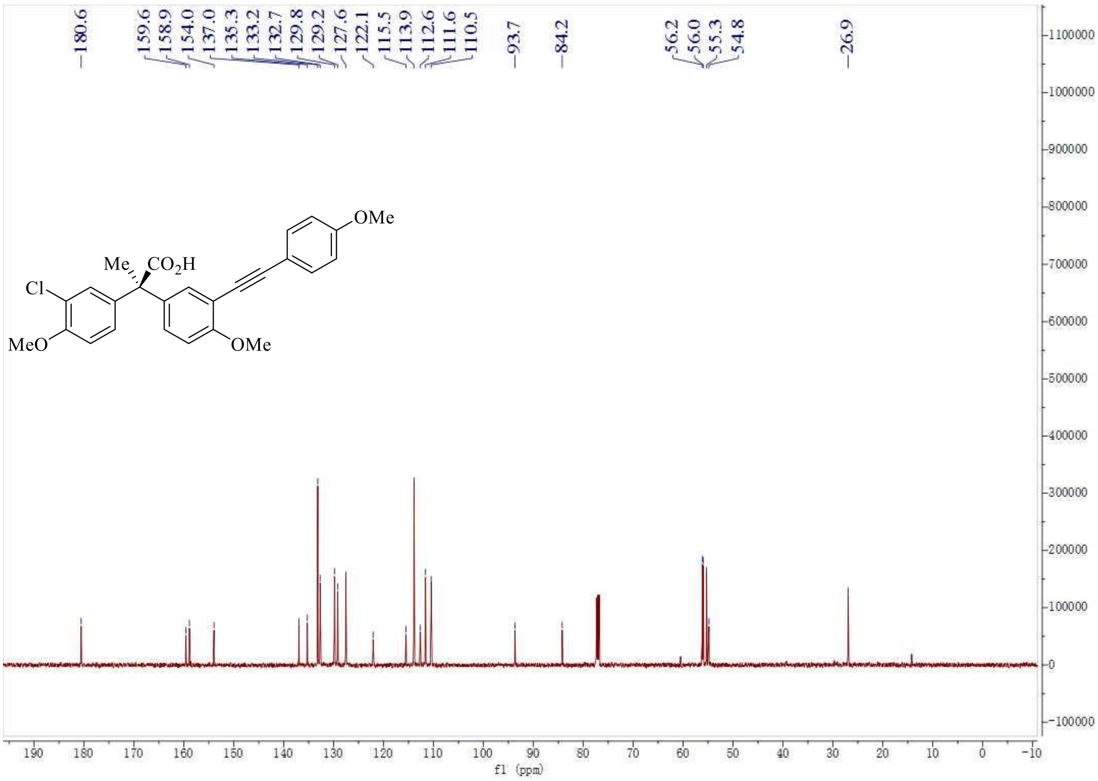

54

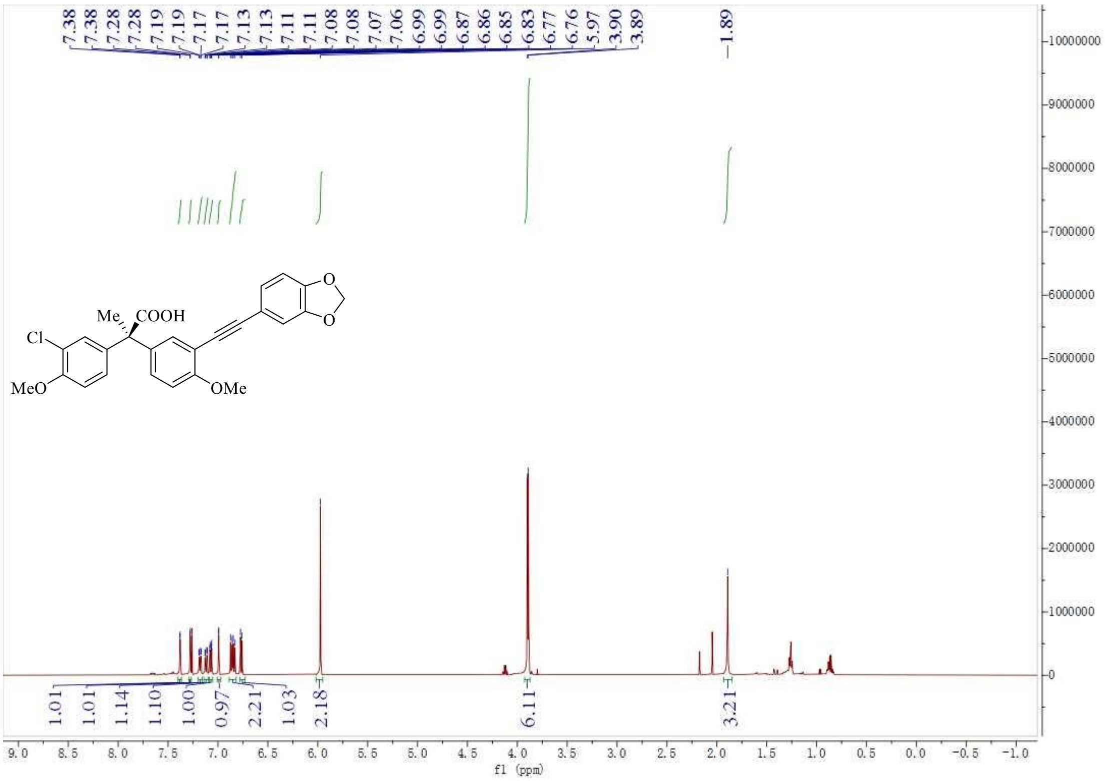
7.38
7.38
7.28
7.28
7.19
7.19
7.17
7.17
7.13
7.13
7.11
7.11
7.08
7.08
7.07
7.06
6.99
6.99
6.87
6.86
6.85
6.83
6.77
6.76
5.97
3.90
3.89
1.89
Me
COOH
Cl
MeO
OMe
O
O
1.01
1.01
1.14
1.10
1.00
0.97
2.21
1.03
2.18
6.11
3.21
f1 (ppm)

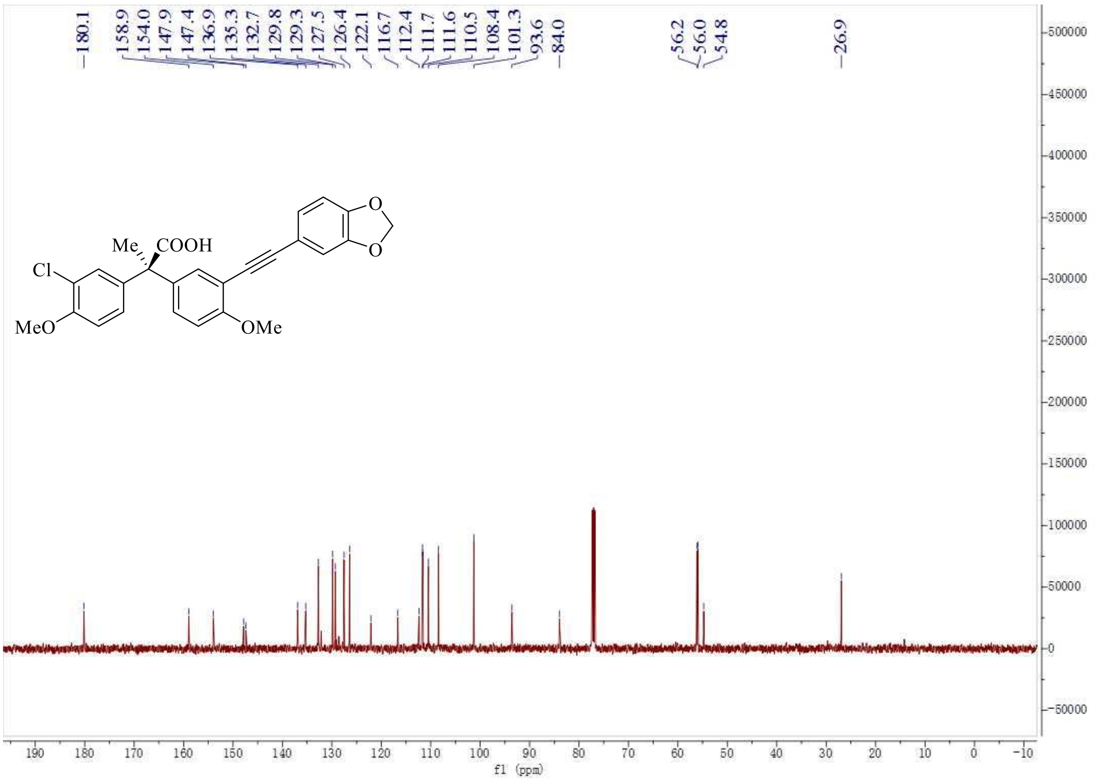
180.1
158.9
154.0
147.9
147.4
136.9
135.3
132.7
129.8
129.3
127.5
126.4
122.1
116.7
112.4
111.7
111.6
110.5
108.4
101.3
93.6
84.0
56.2
56.0
54.8
26.9
Me
COOH
Cl
MeO
OMe
O
O
f1 (ppm)

55

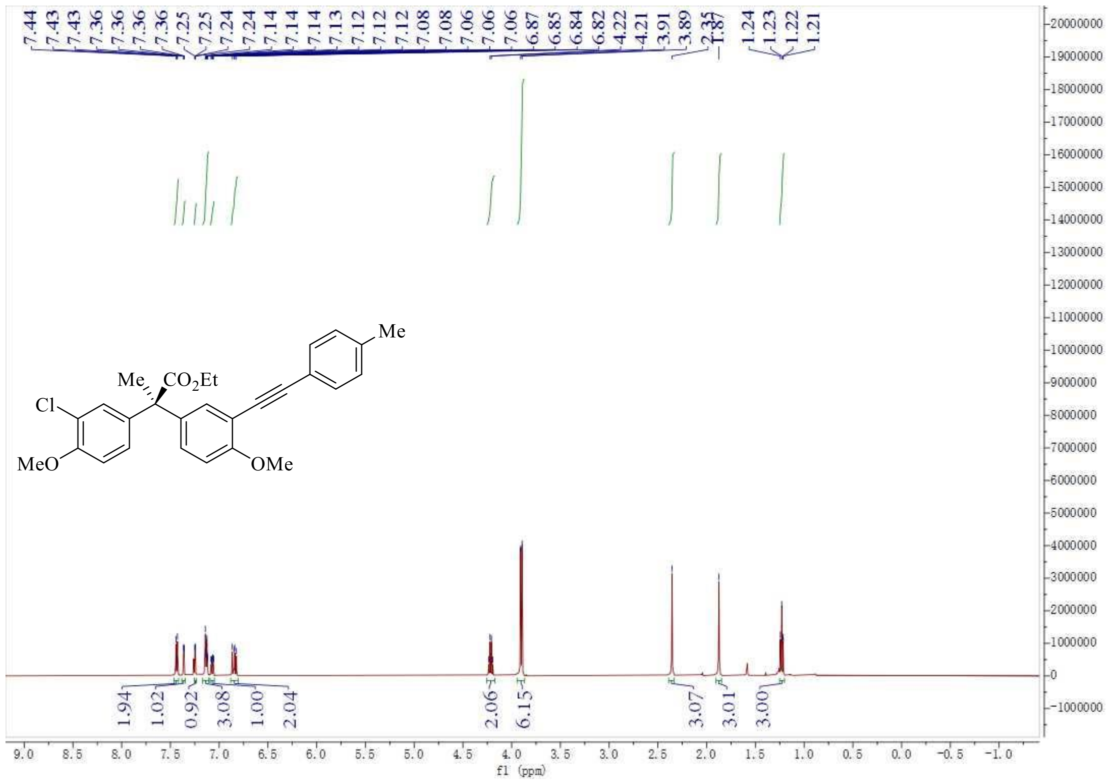

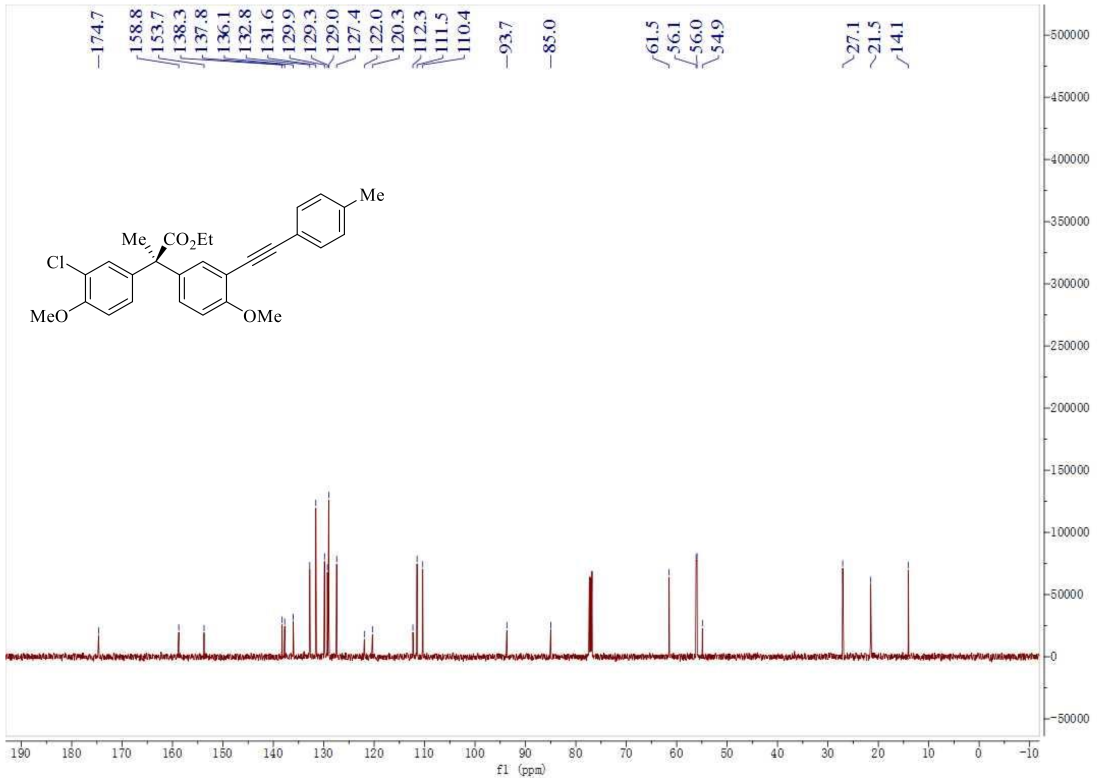

56

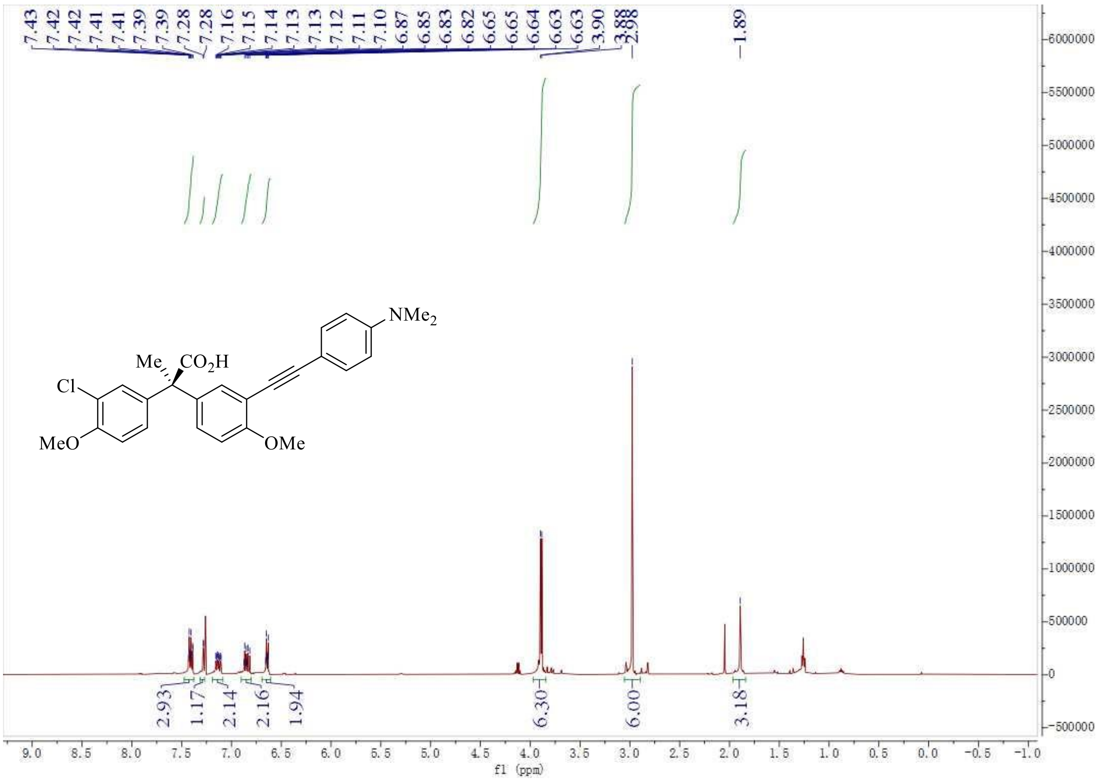

7.43
7.42
7.42
7.41
7.41
7.39
7.39
7.28
7.28
7.16
7.15
7.14
7.13
7.13
7.12
7.11
7.10
6.87
6.85
6.83
6.82
6.65
6.65
6.64
6.63
6.63
3.90
3.88
2.98
1.89
NMe2
Me
CO2H
Cl
MeO
OMe
2.93
1.17
2.14
2.16
1.94
6.30
6.00
3.18
f1 (ppm)

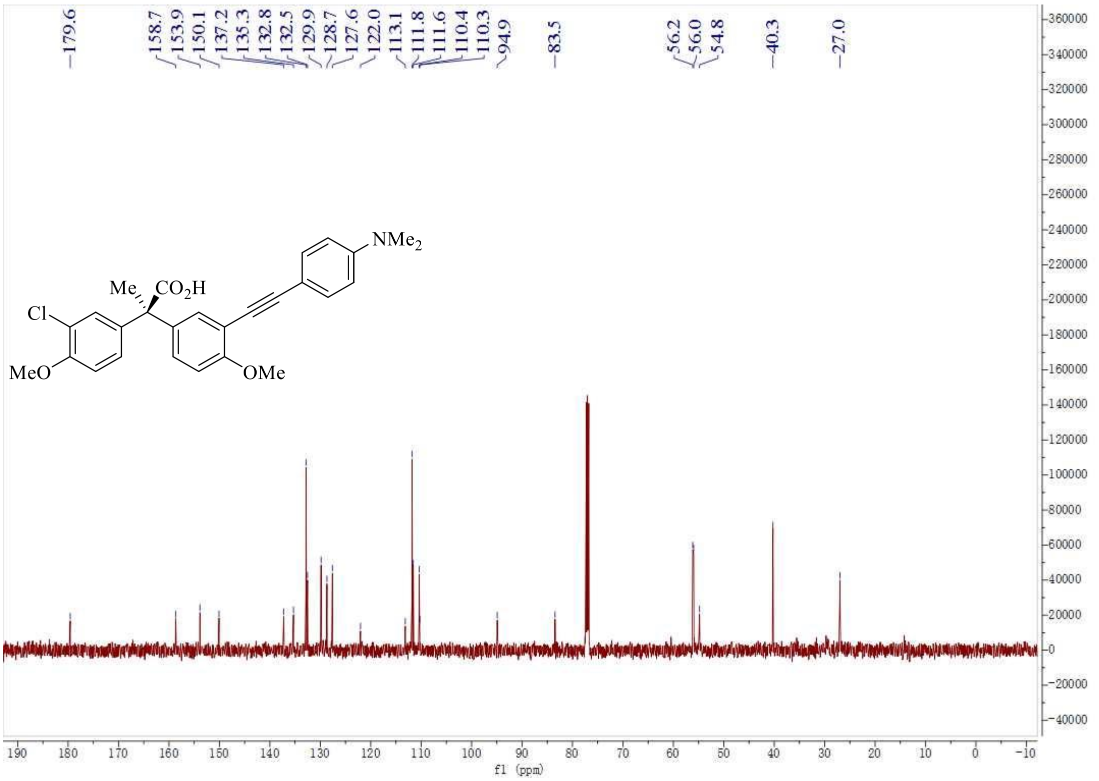

179.6
158.7
153.9
150.1
137.2
135.3
132.8
132.5
129.9
128.7
127.6
122.0
113.1
111.8
111.6
110.4
110.3
94.9
83.5
56.2
56.0
54.8
40.3
27.0
NMe2
Me
CO2H
Cl
MeO
OMe
f1 (ppm)

57

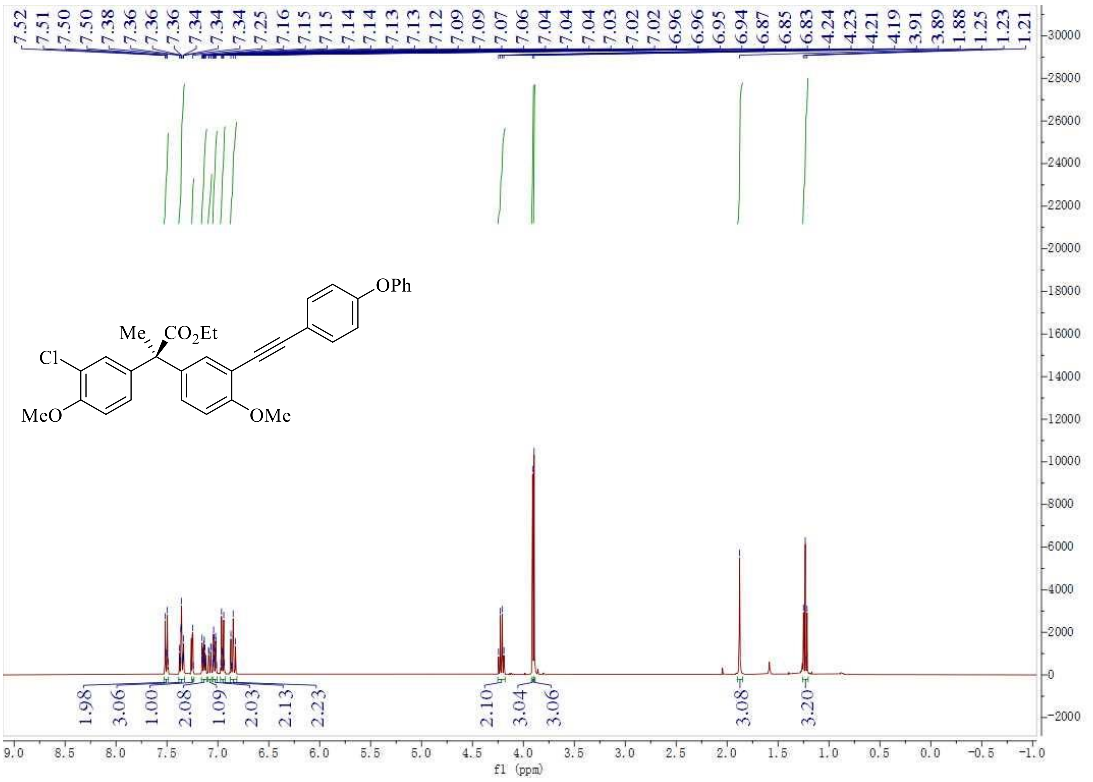
7.52
7.51
7.50
7.50
7.38
7.36
7.36
7.36
7.34
7.34
7.34
7.34
7.25
7.16
7.15
7.15
7.14
7.14
7.13
7.13
7.12
7.09
7.09
7.07
7.06
7.04
7.04
7.04
7.03
7.02
7.02
6.96
6.96
6.95
6.94
6.87
6.85
6.83
4.24
4.23
4.21
4.19
3.91
3.89
1.88
1.25
1.23
1.21
OPh
Me
CO2Et
Cl
MeO
OMe
1.98
3.06
1.00
2.08
1.09
2.03
2.13
2.23
2.10
3.04
3.06
3.08
3.20
f1 (ppm)

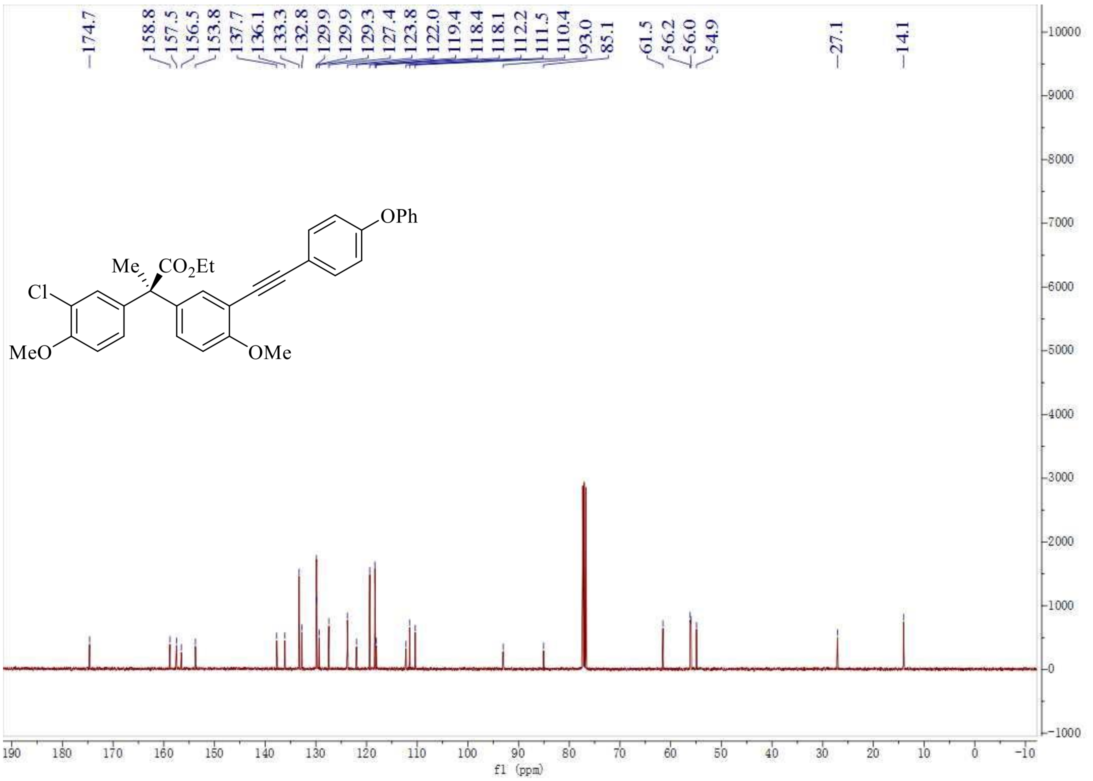
174.7
158.8
157.5
156.5
153.8
137.7
136.1
133.3
132.8
129.9
129.9
129.3
127.4
123.8
122.0
119.4
118.4
118.1
112.2
111.5
110.4
93.0
85.1
61.5
56.2
56.0
54.9
27.1
14.1
OPh
Me
CO2Et
Cl
MeO
OMe
f1 (ppm)

58

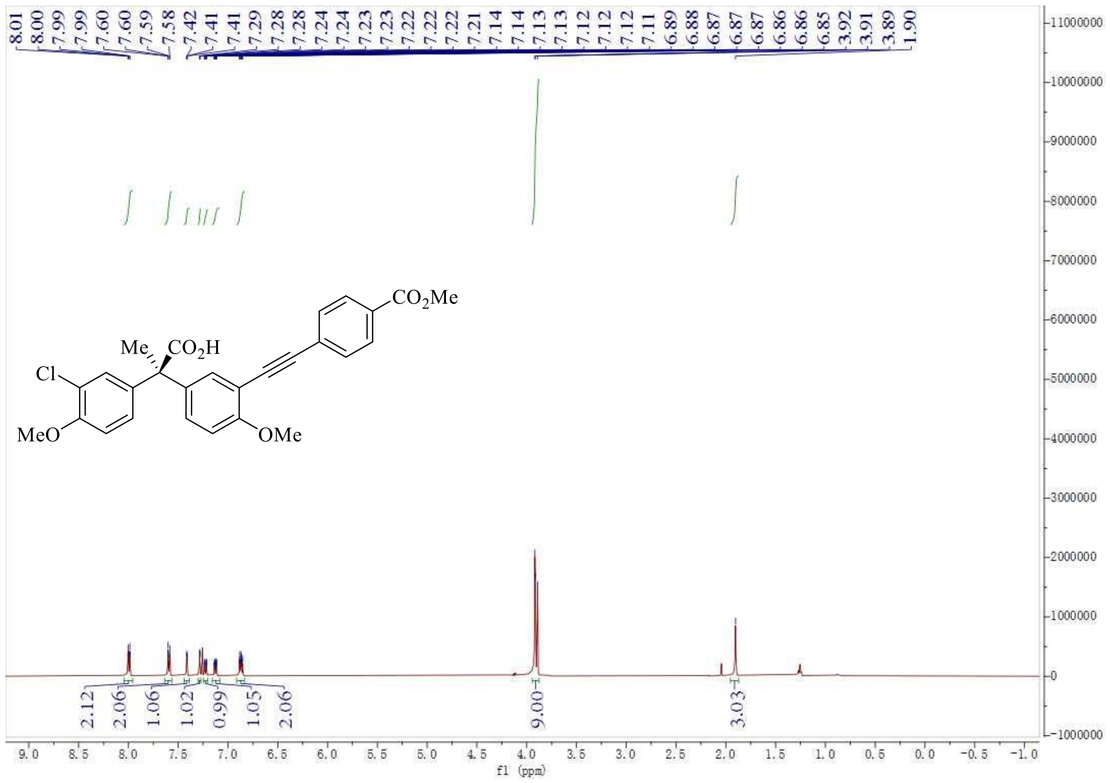

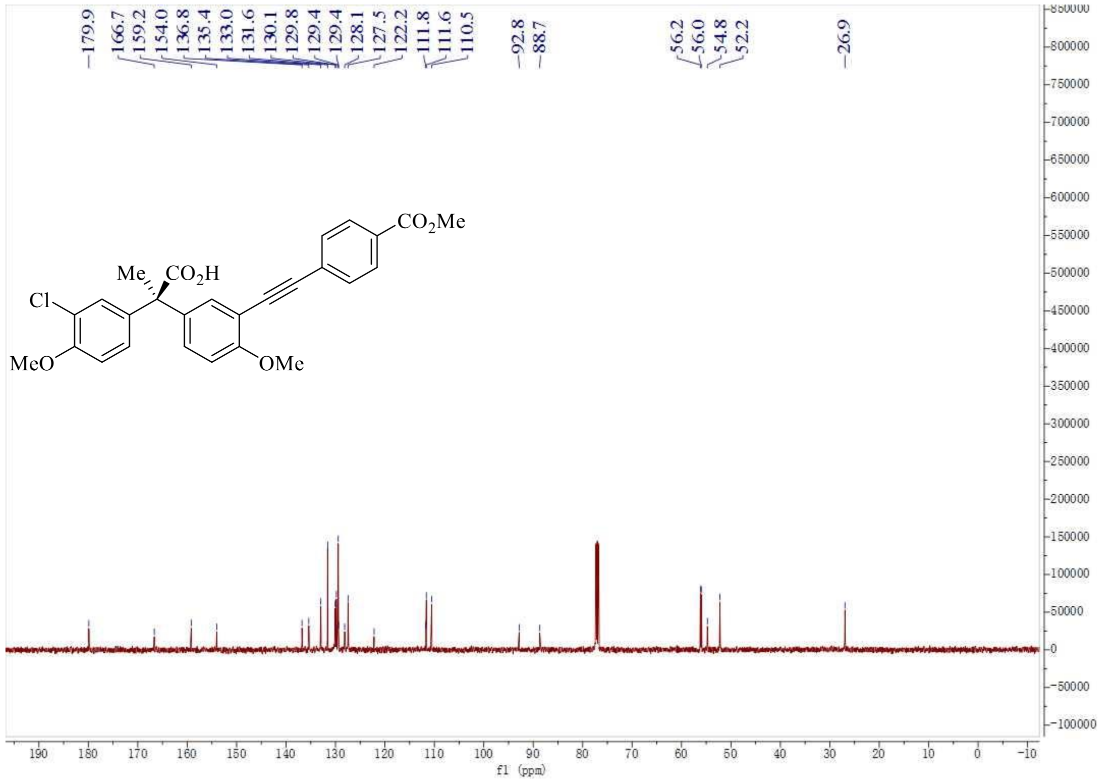

59

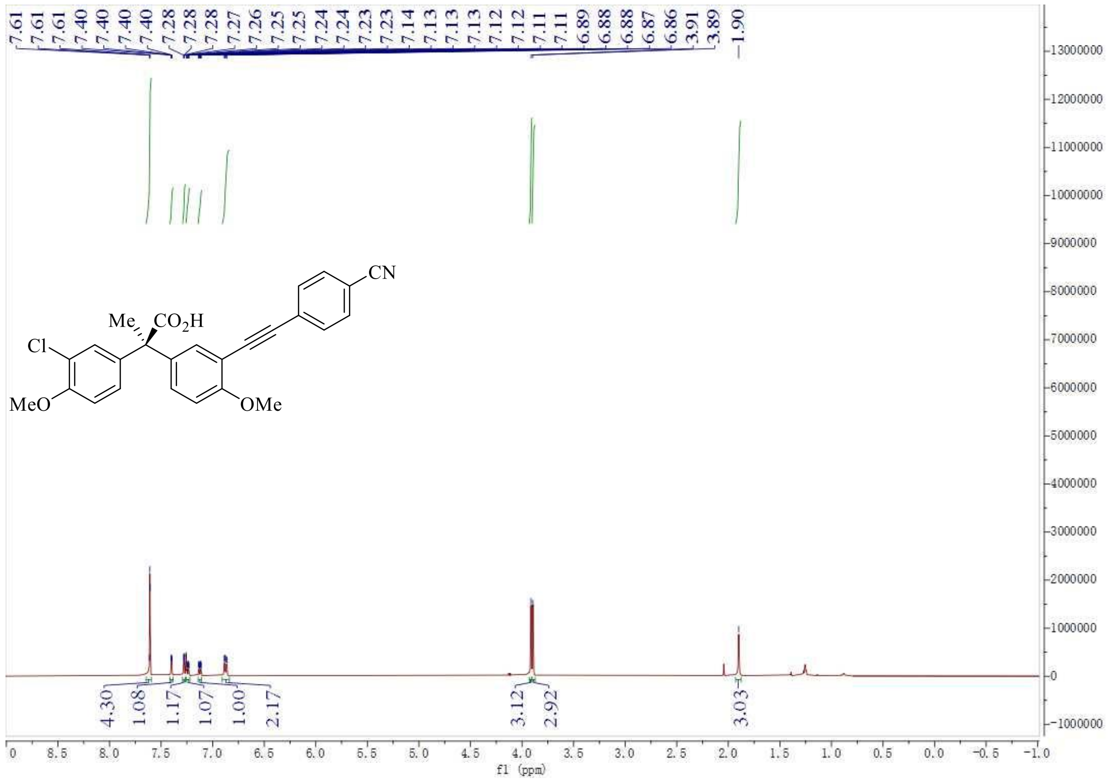

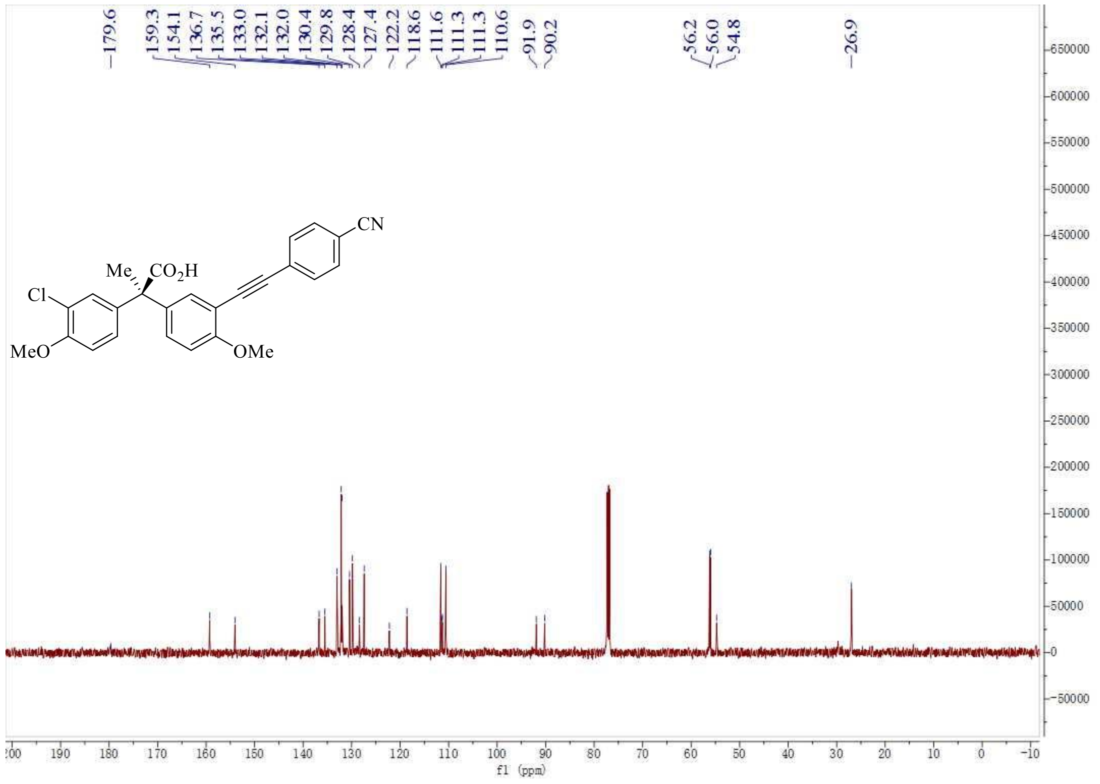

60

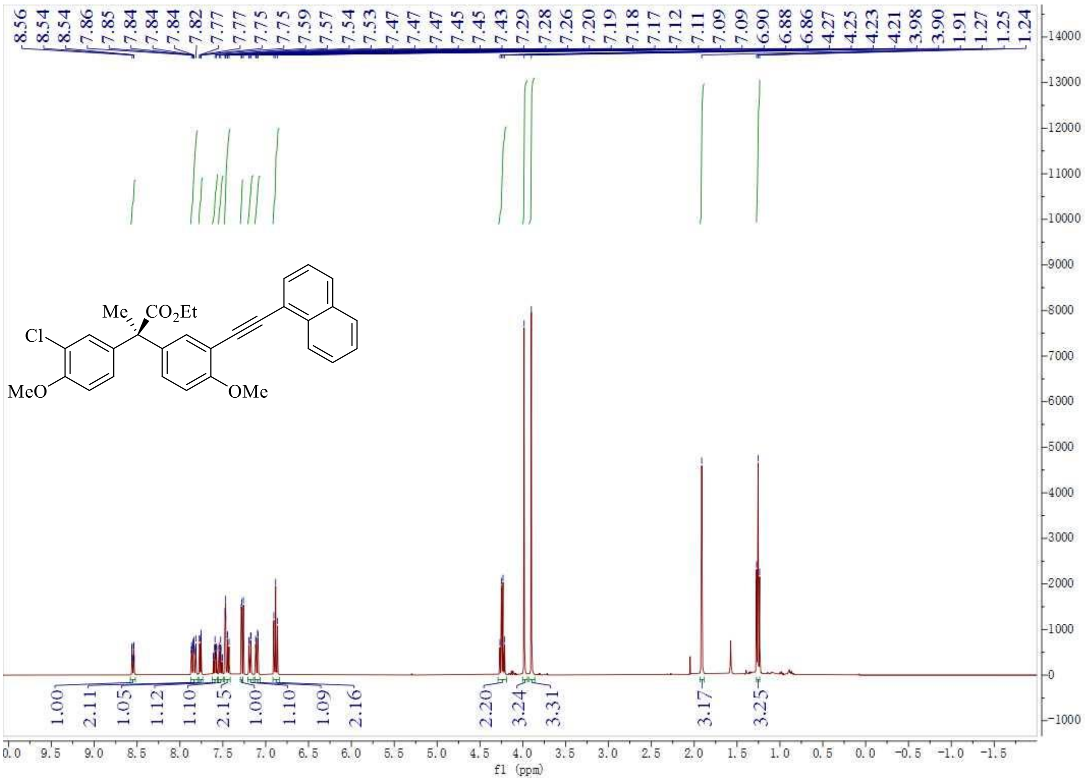

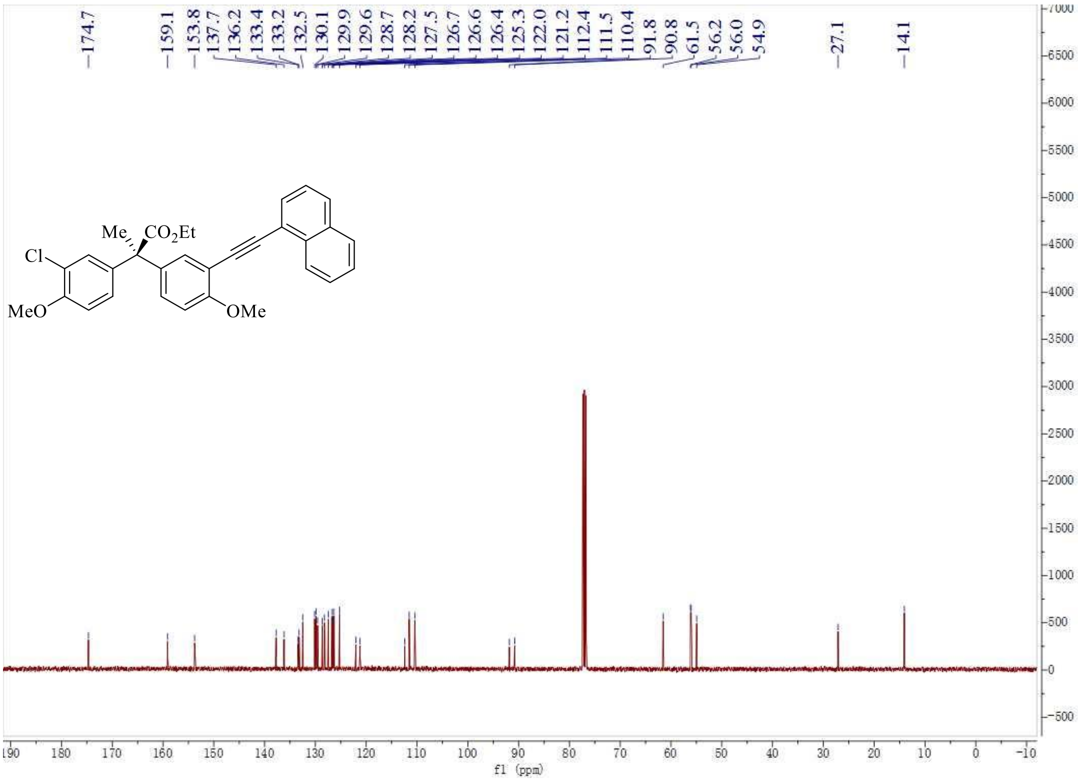

61

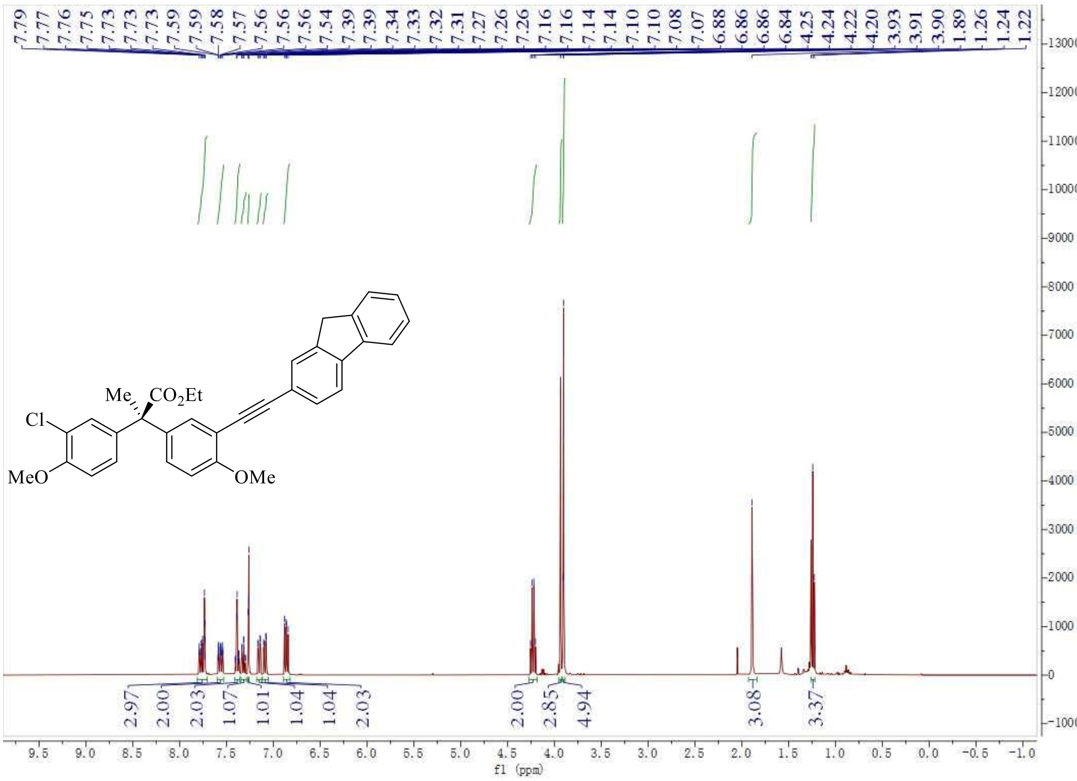
7.79
7.77
7.76
7.75
7.73
7.73
7.73
7.59
7.59
7.58
7.57
7.56
7.56
7.54
7.39
7.39
7.34
7.33
7.32
7.31
7.27
7.26
7.26
7.16
7.16
7.14
7.14
7.10
7.10
7.08
7.07
6.88
6.86
6.86
6.84
4.25
4.24
4.22
4.20
3.93
3.91
3.90
1.89
1.26
1.24
1.22
Me
CO2Et
Cl
MeO
OMe
2.97
2.00
2.03
1.07
1.01
1.04
1.04
2.03
2.00
2.85
4.94
3.08
3.37
f1 (ppm)

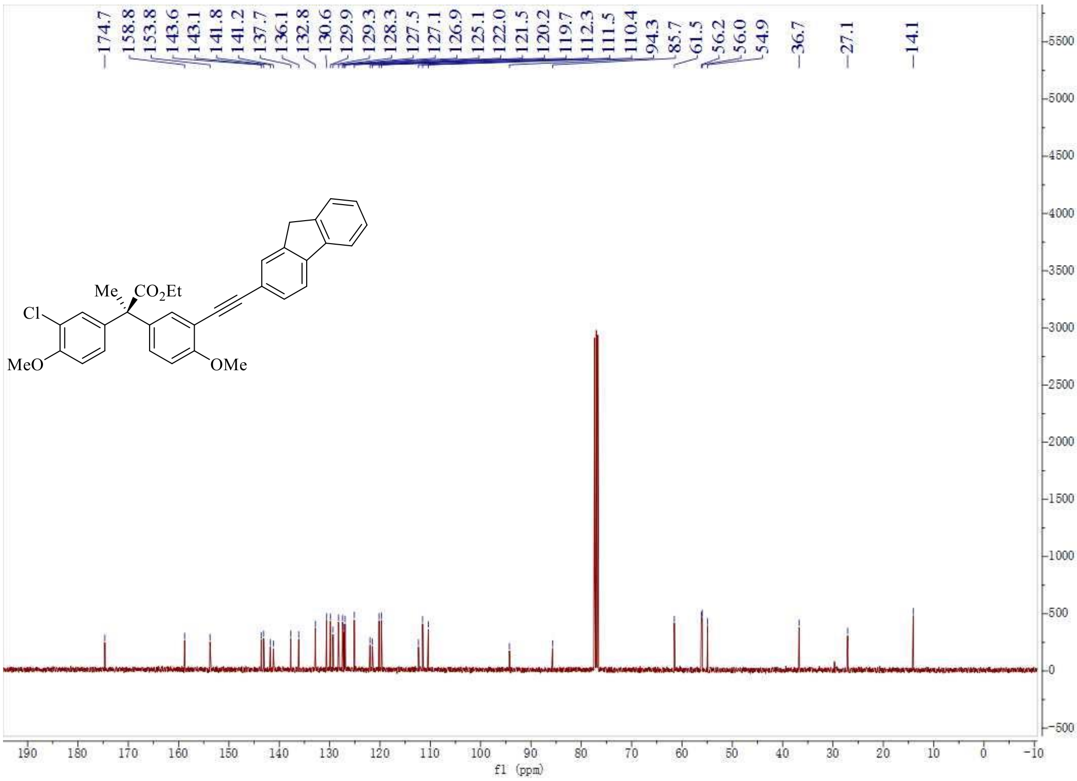
174.7
158.8
153.8
143.6
143.1
141.8
141.2
137.7
136.1
132.8
130.6
129.9
129.3
128.3
127.5
127.1
126.9
125.1
122.0
121.5
120.2
119.7
112.3
111.5
110.4
94.3
85.7
61.5
56.2
56.0
54.9
36.7
27.1
14.1
Me
CO2Et
Cl
MeO
OMe
f1 (ppm)

62

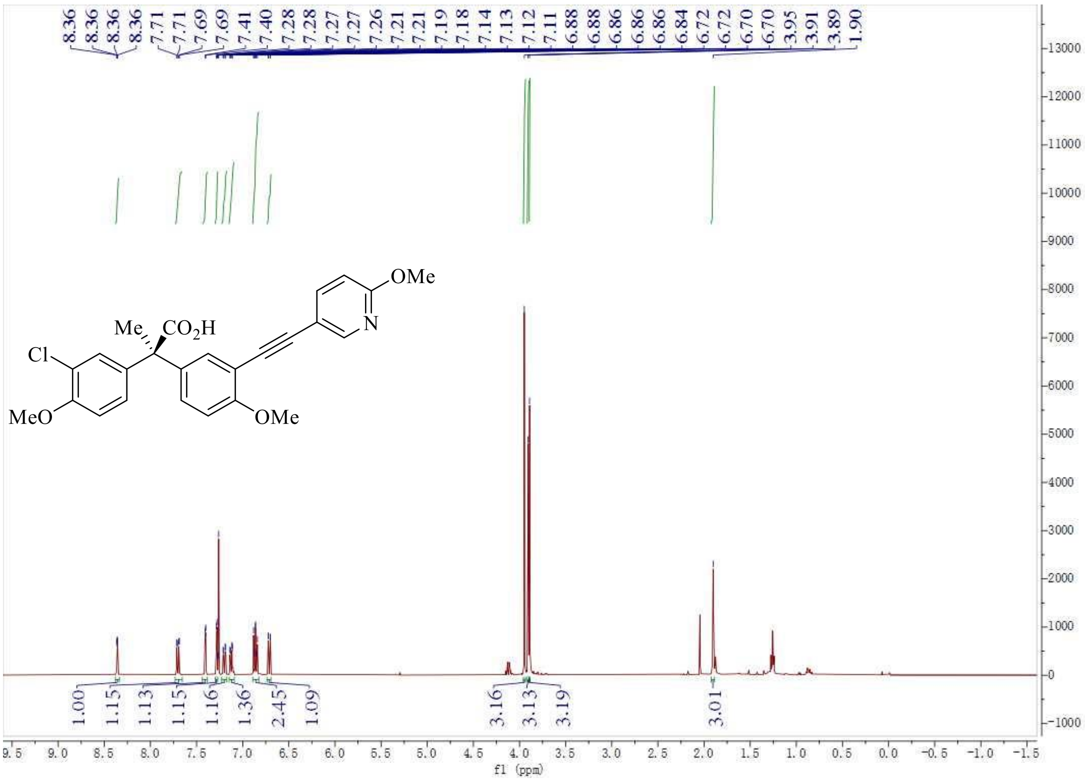

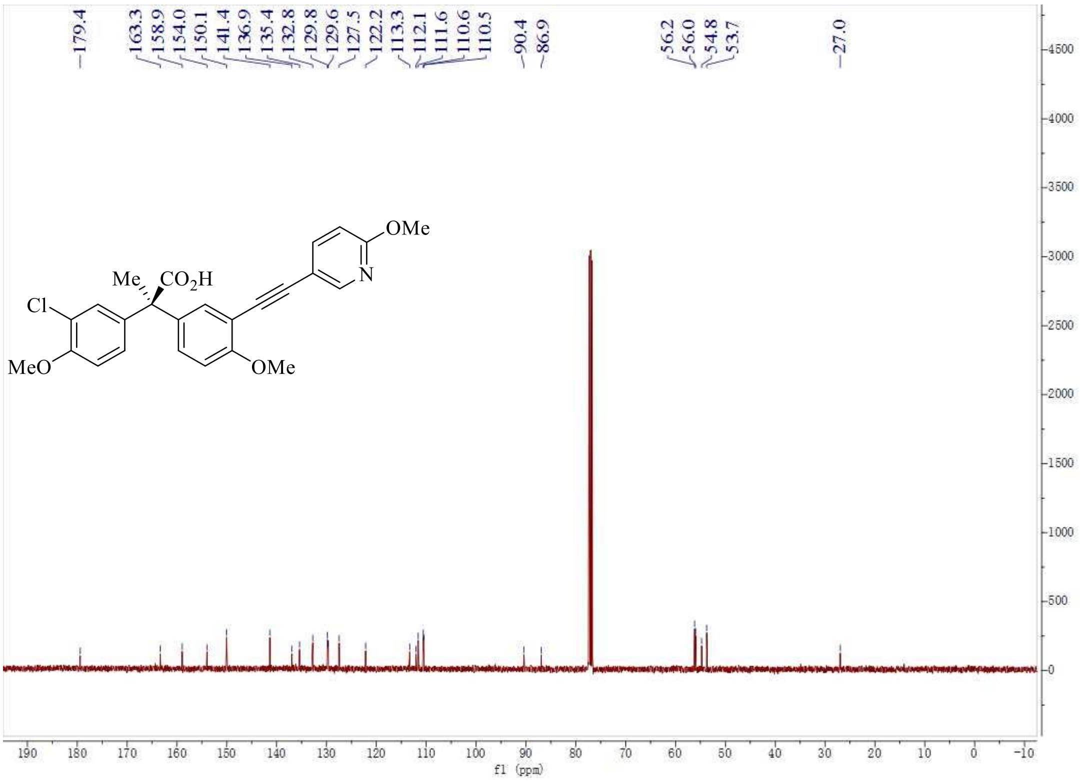

63

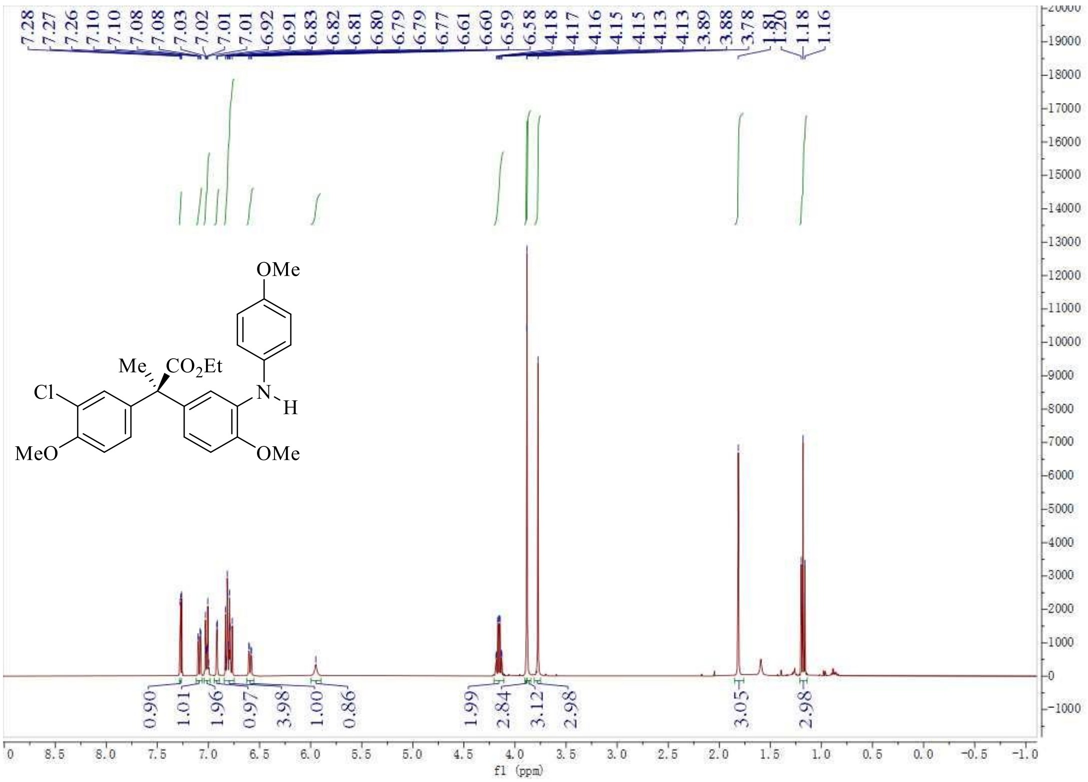
7.28
7.27
7.26
7.10
7.10
7.08
7.08
7.03
7.02
7.01
7.01
6.92
6.91
6.83
6.82
6.81
6.80
6.79
6.79
6.77
6.61
6.60
6.59
6.58
4.18
4.17
4.16
4.15
4.15
4.13
4.13
3.89
3.88
3.78
1.81
1.20
1.18
1.16
OMe
Me
CO2Et
Cl
N
H
MeO
OMe
0.90
1.01
1.96
0.97
3.98
1.00
0.86
1.99
2.84
3.12
2.98
3.05
2.98
f1 (ppm)

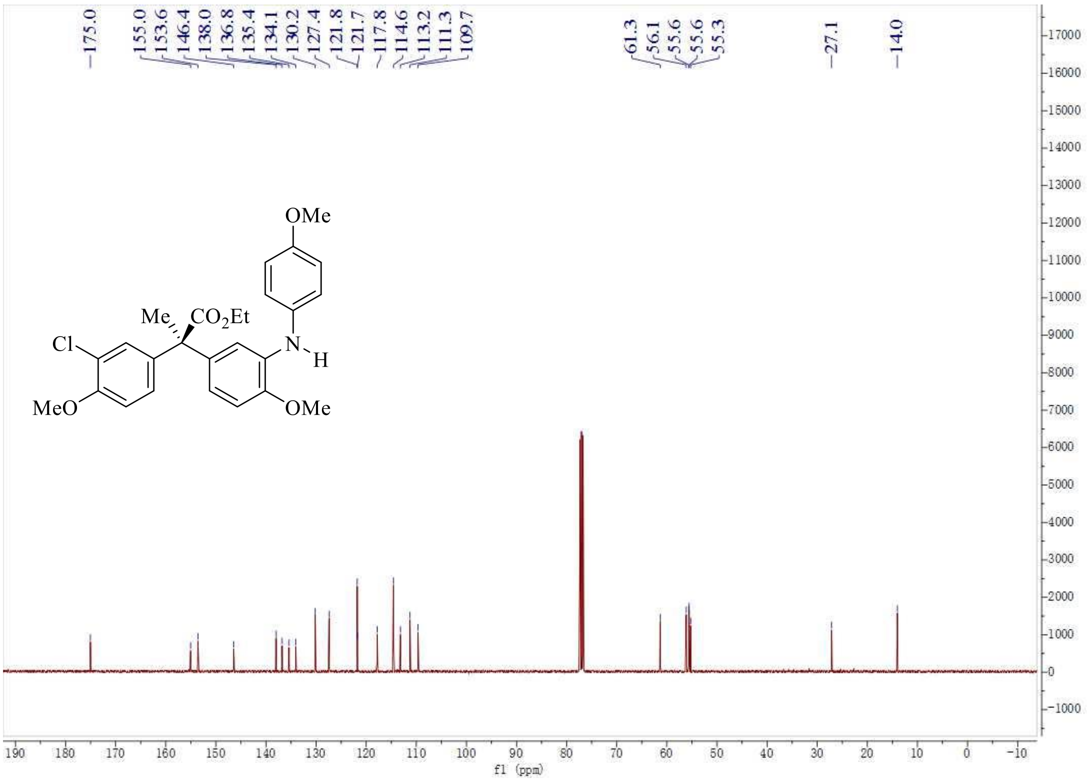
175.0
155.0
153.6
146.4
138.0
136.8
135.4
134.1
130.2
127.4
121.8
121.7
117.8
114.6
113.2
111.3
109.7
61.3
56.1
55.6
55.6
55.3
27.1
14.0
OMe
Me
CO2Et
Cl
N
H
MeO
OMe
f1 (ppm)

64

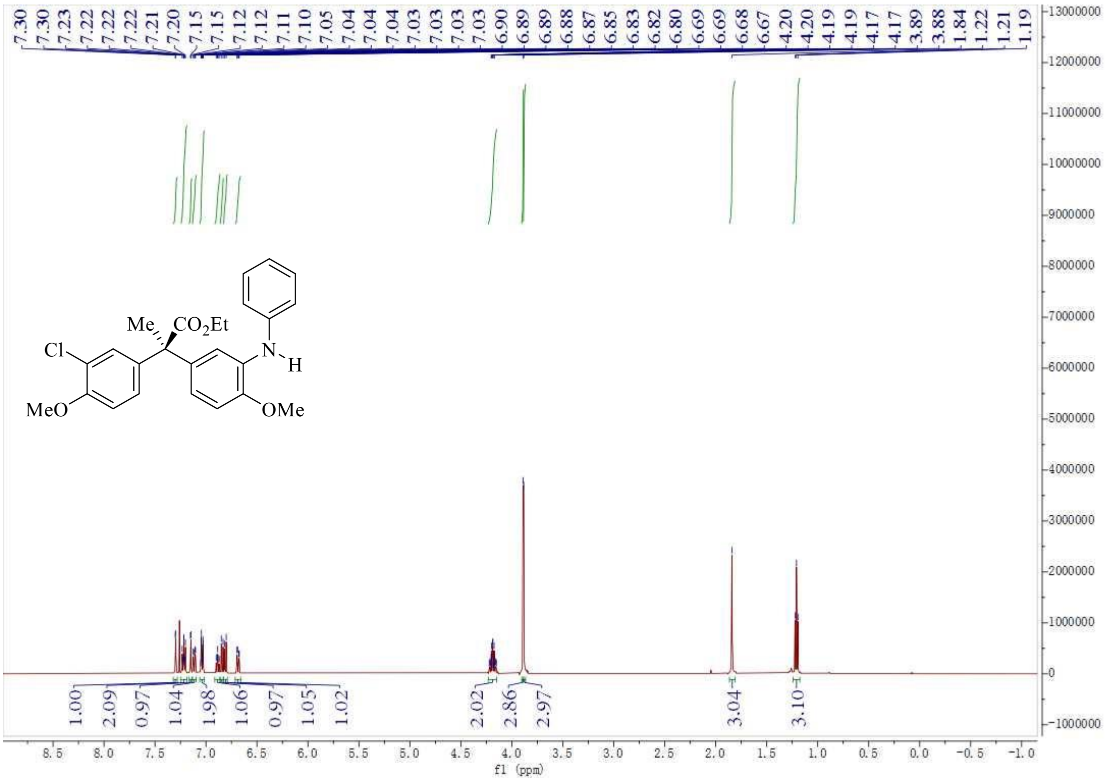

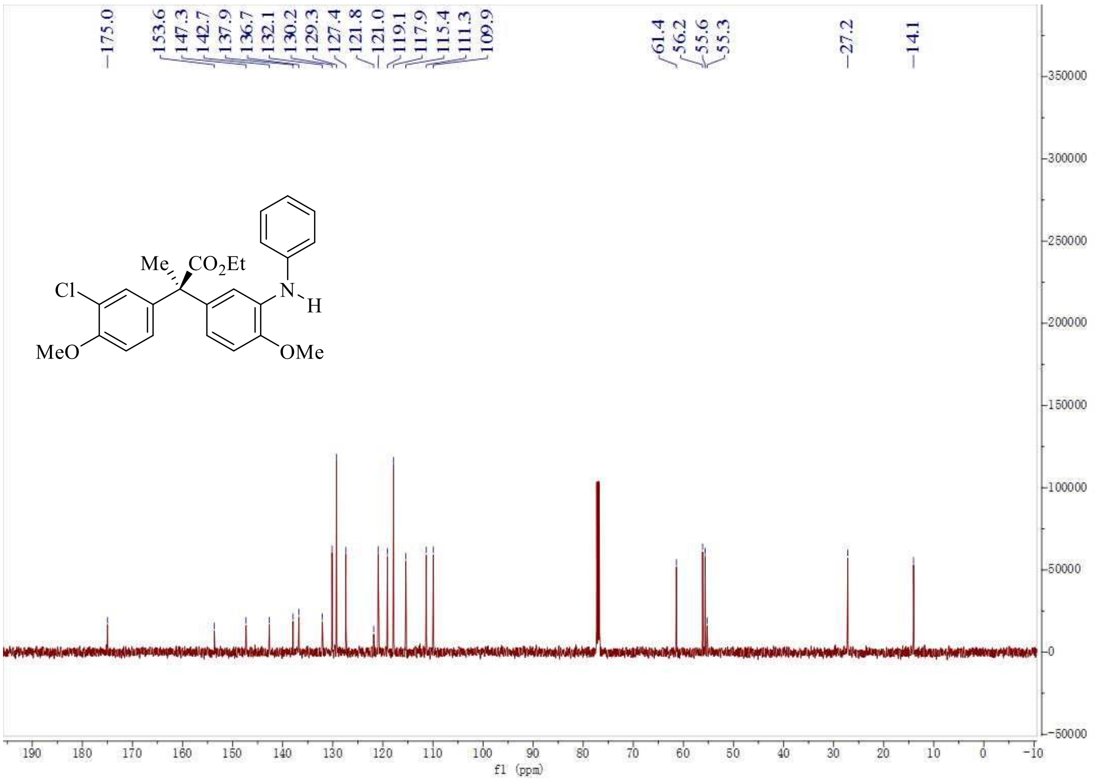

65

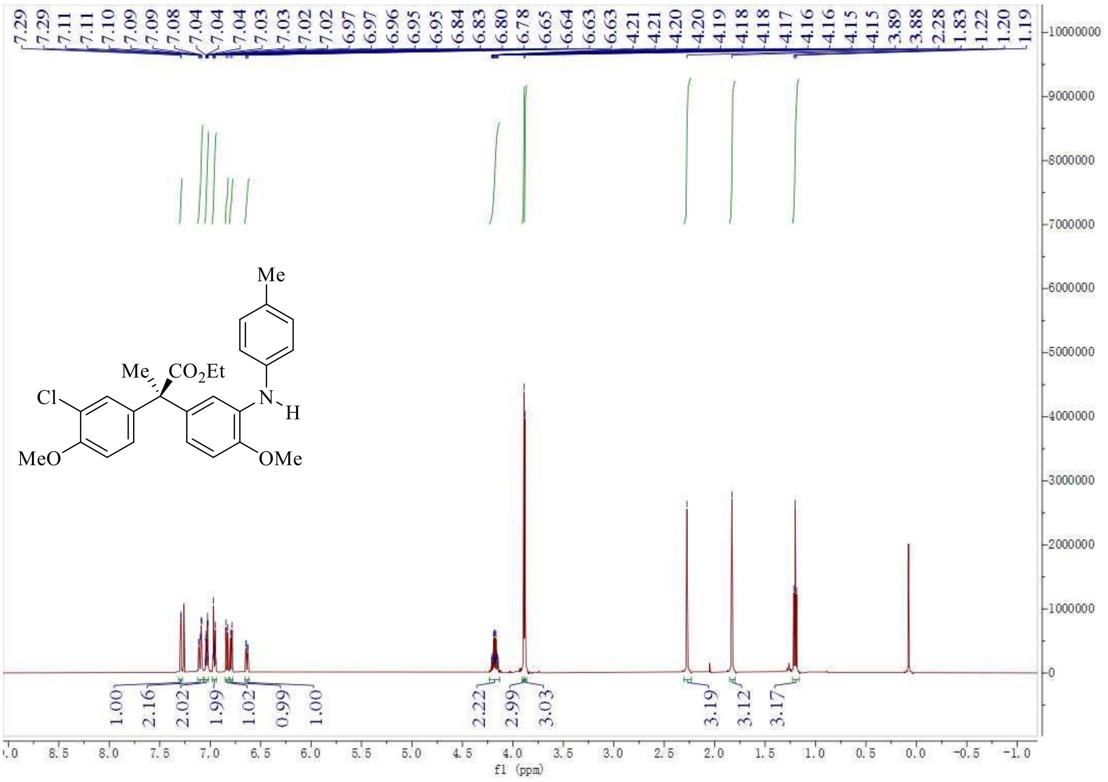

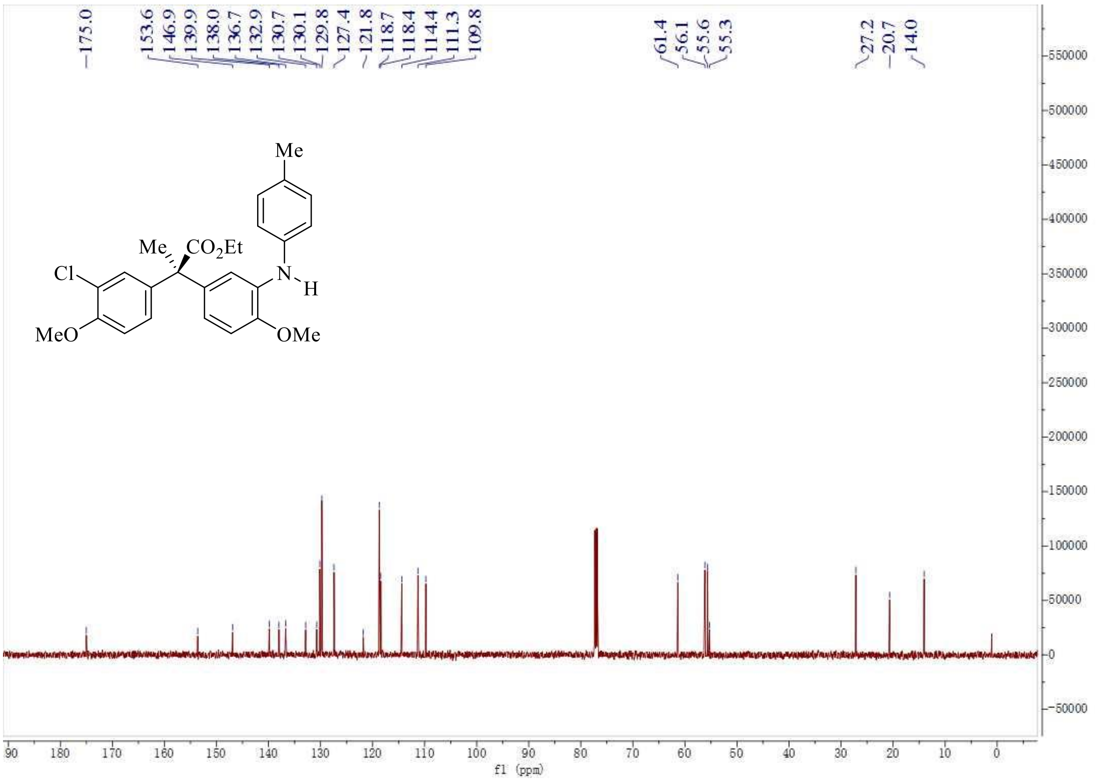

66

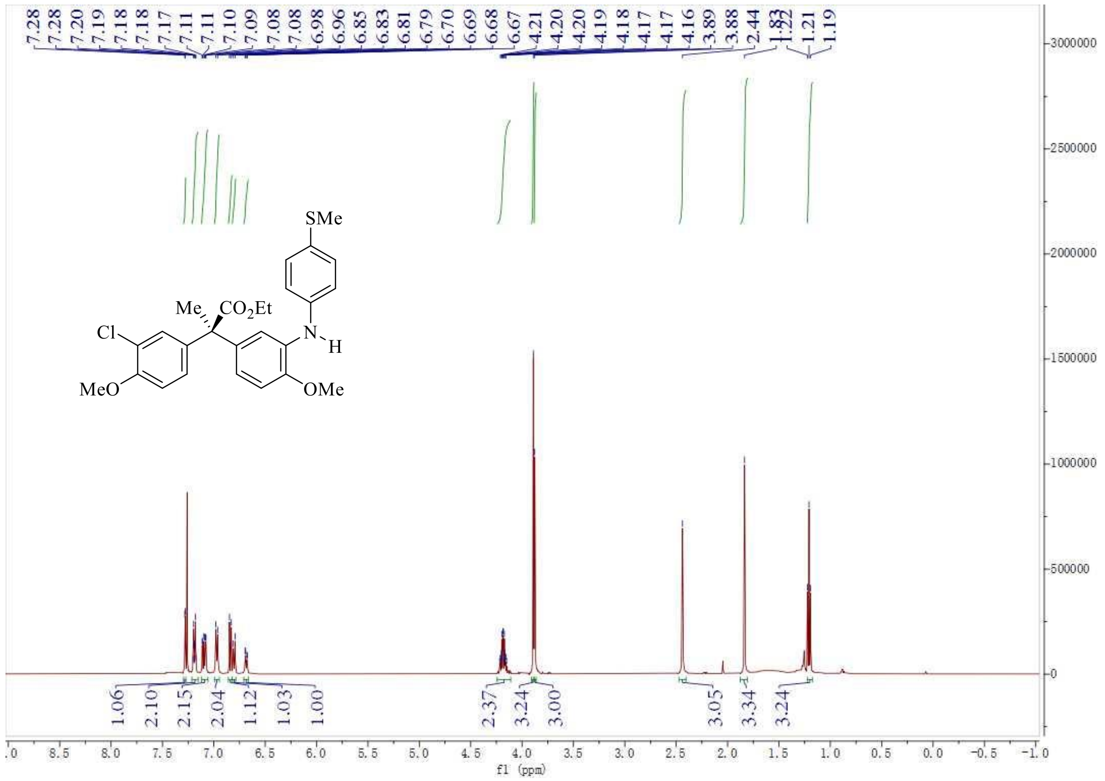

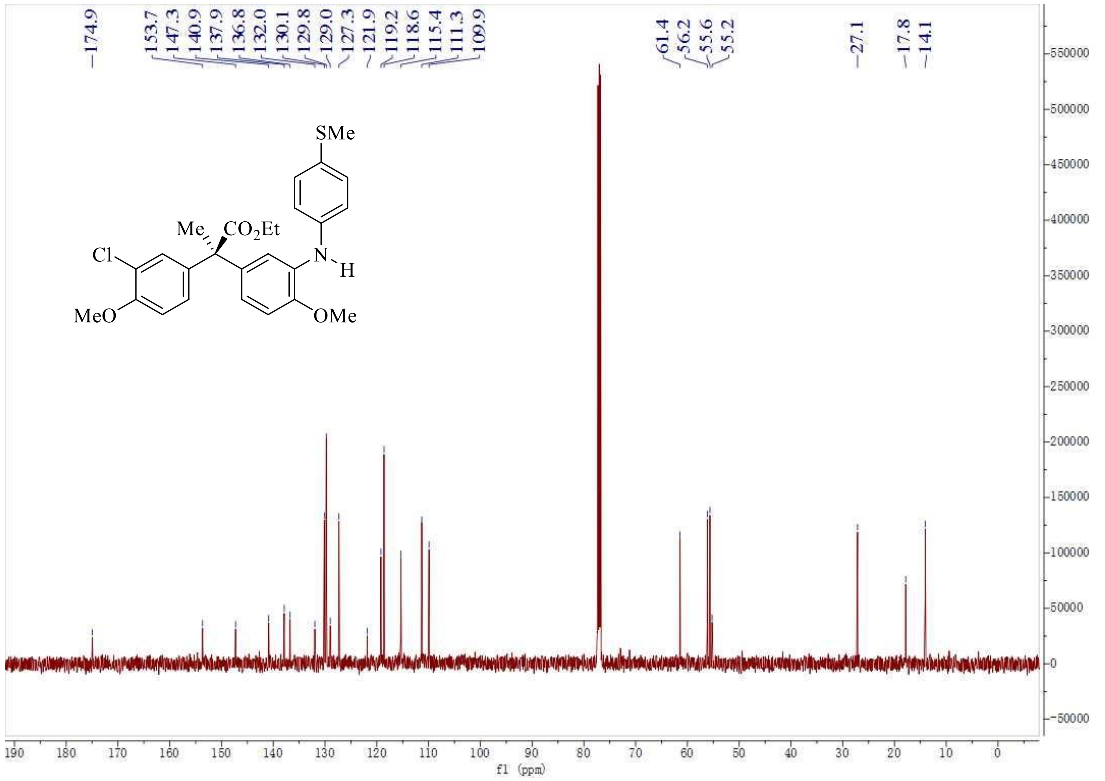

67

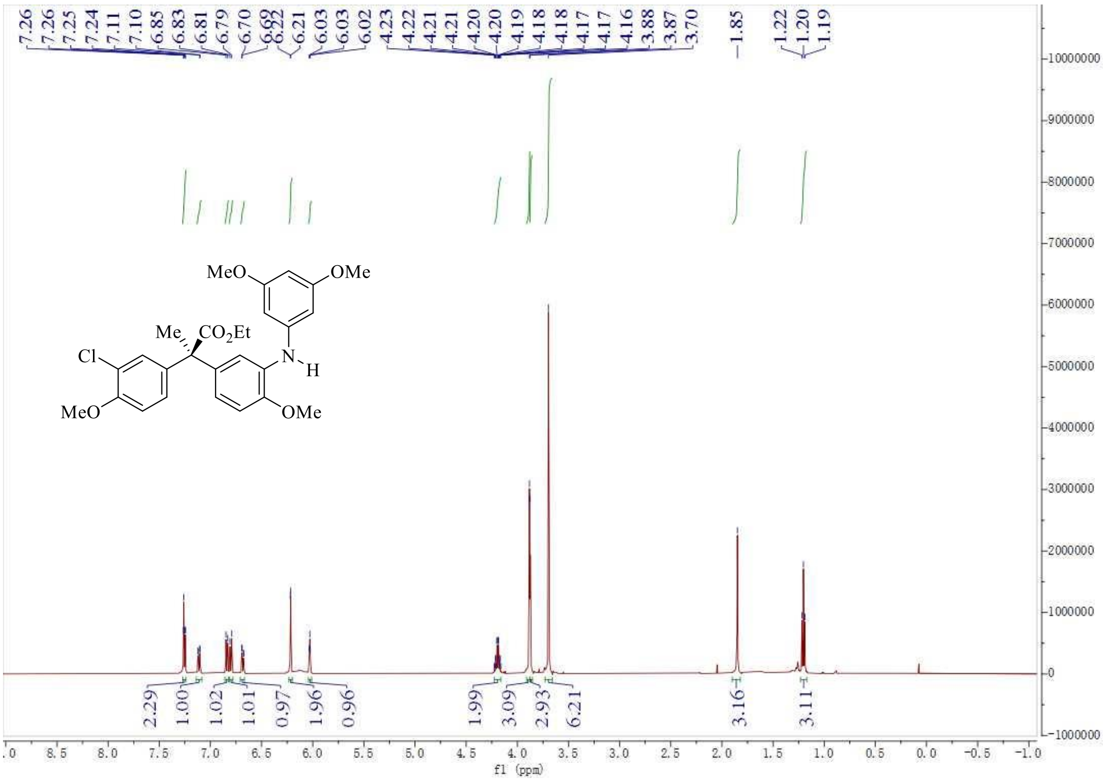

7.26
7.26
7.25
7.24
7.11
7.10
6.85
6.83
6.81
6.79
6.70
6.69
6.22
6.21
6.03
6.03
6.02
4.23
4.22
4.21
4.21
4.20
4.20
4.19
4.18
4.18
4.17
4.17
4.16
3.88
3.87
3.70
1.85
1.22
1.20
1.19
MeO
OMe
Me
CO2Et
N
H
Cl
MeO
OMe
2.29
1.00
1.02
1.01
0.97
1.96
0.96
1.99
3.09
2.93
6.21
3.16
3.11
f1 (ppm)

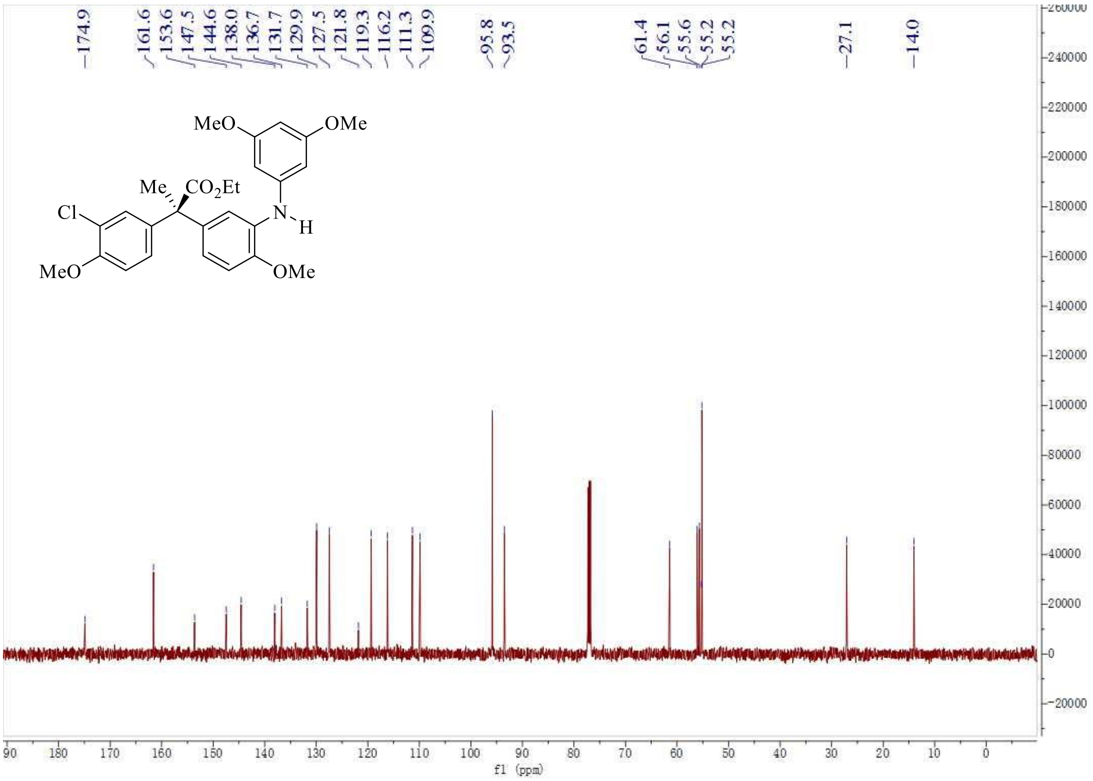

174.9
161.6
153.6
147.5
144.6
138.0
136.7
131.7
129.9
127.5
121.8
119.3
116.2
111.3
109.9
95.8
93.5
61.4
56.1
55.6
55.2
55.2
27.1
14.0
MeO
OMe
Me
CO2Et
N
H
Cl
MeO
OMe
f1 (ppm)

68

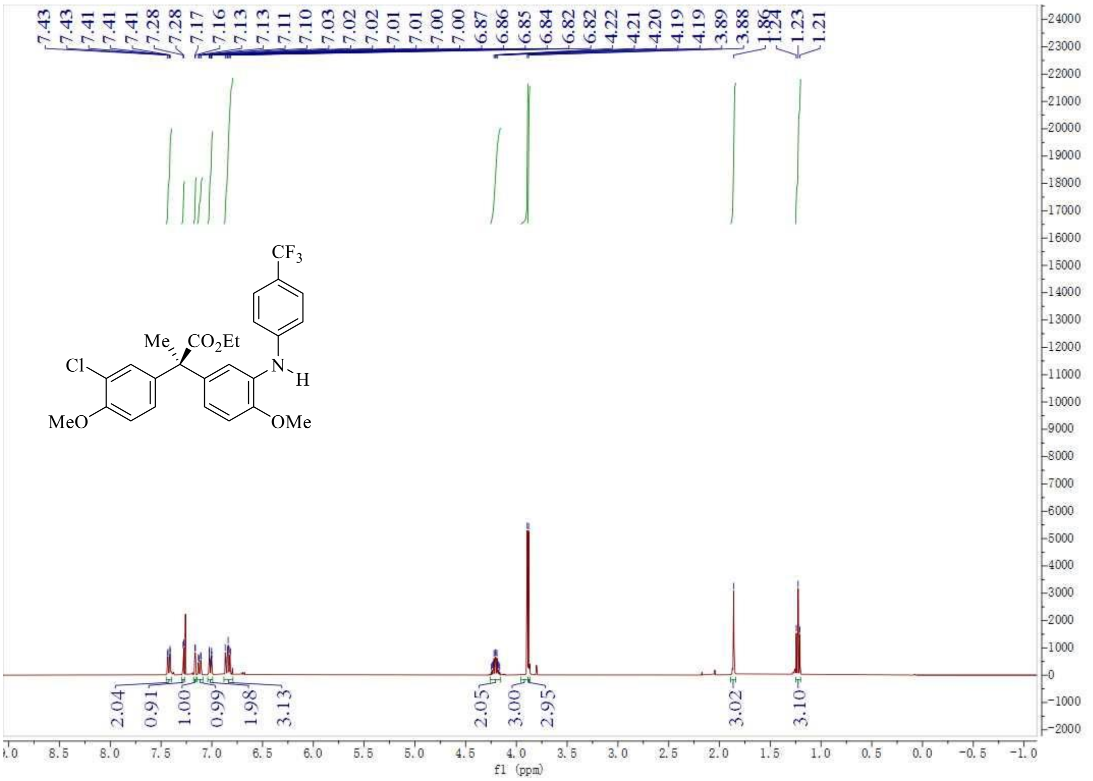

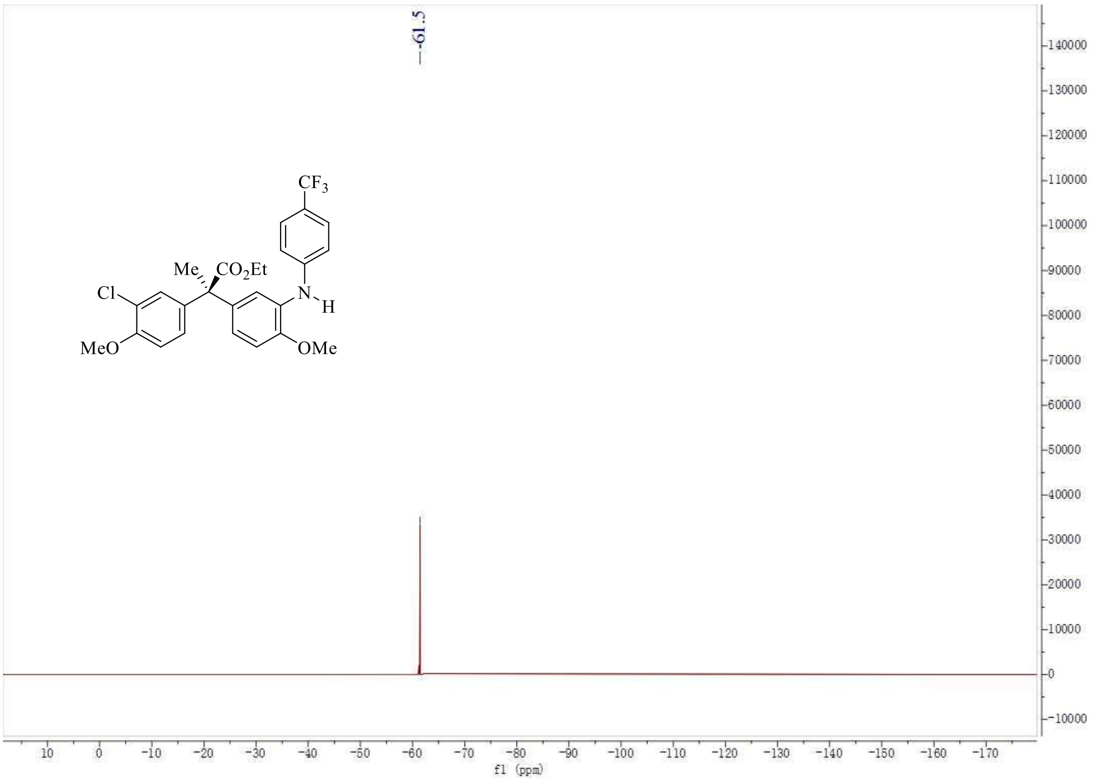

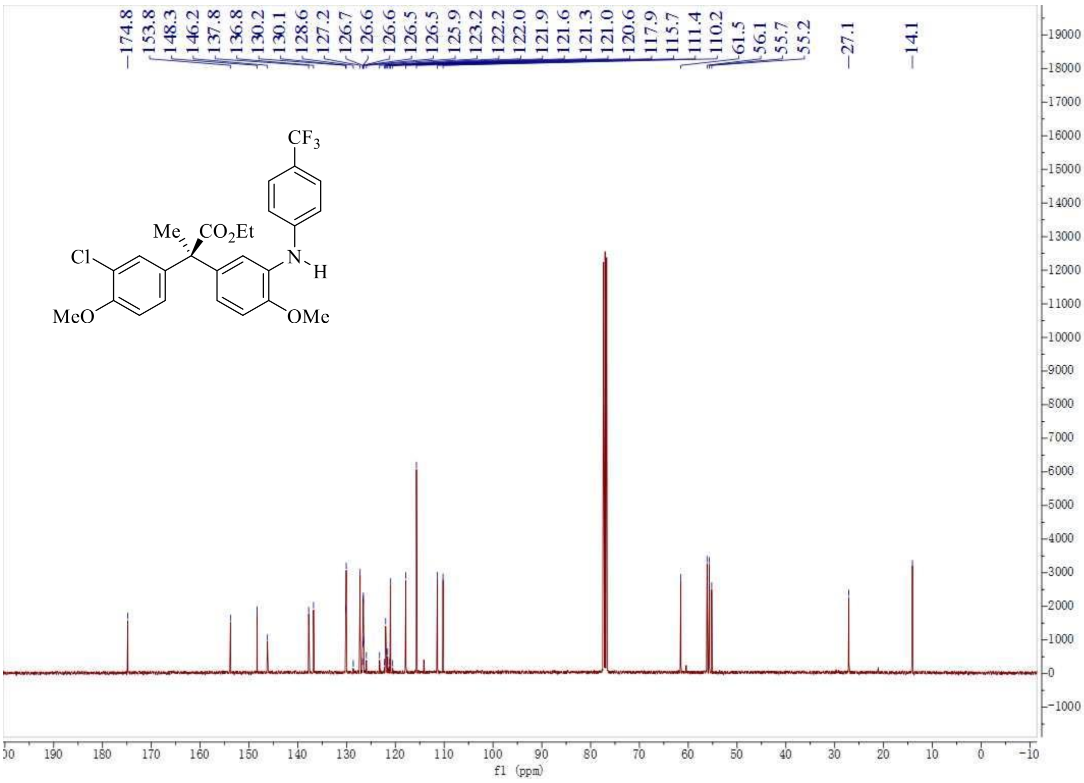

174.8
153.8
148.3
146.2
137.8
136.8
130.2
130.1
128.6
127.2
126.7
126.6
126.6
126.5
126.5
125.9
123.2
122.2
122.0
121.9
121.6
121.3
121.0
120.6
117.9
115.7
111.4
110.2
61.5
56.1
55.7
55.2
27.1
14.1
CF3
Me
CO2Et
Cl
N
H
MeO
OMe
f1 (ppm)

69

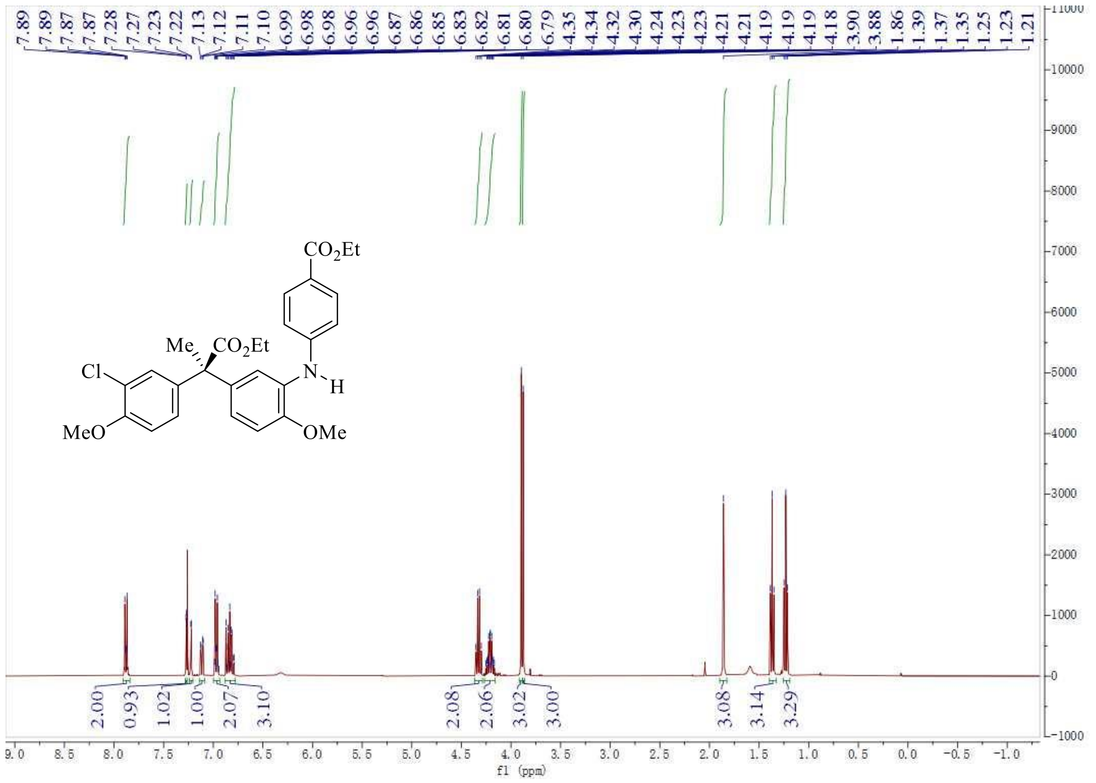

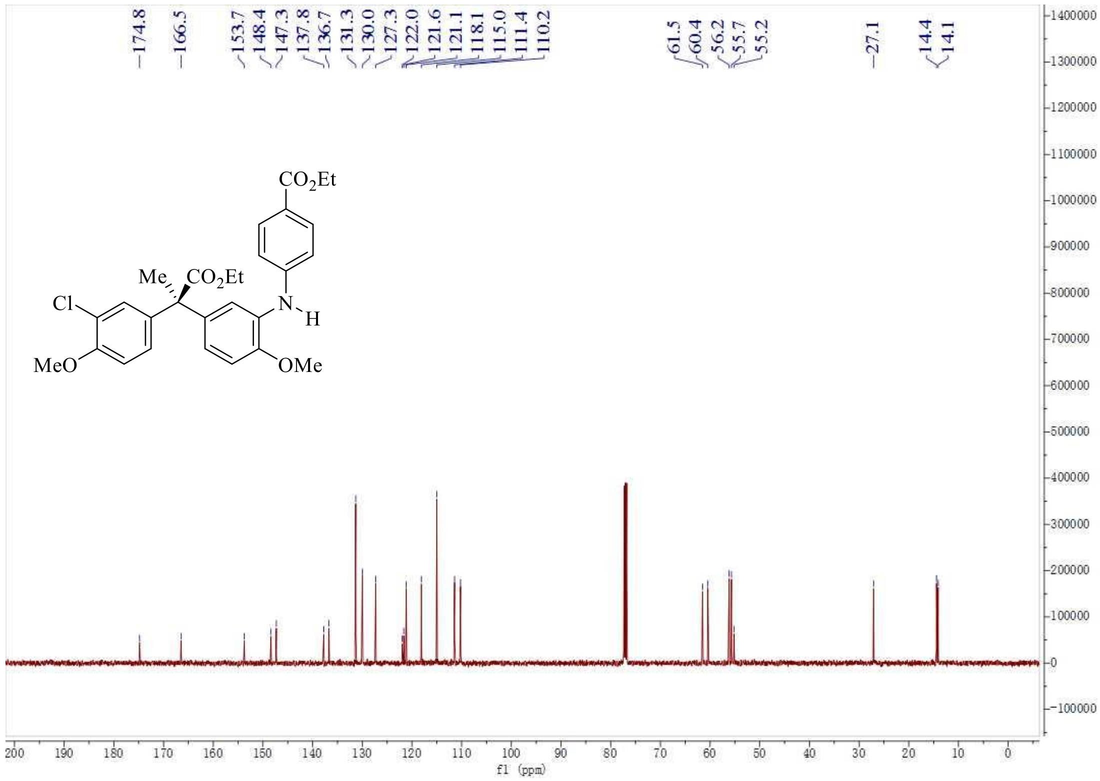

70

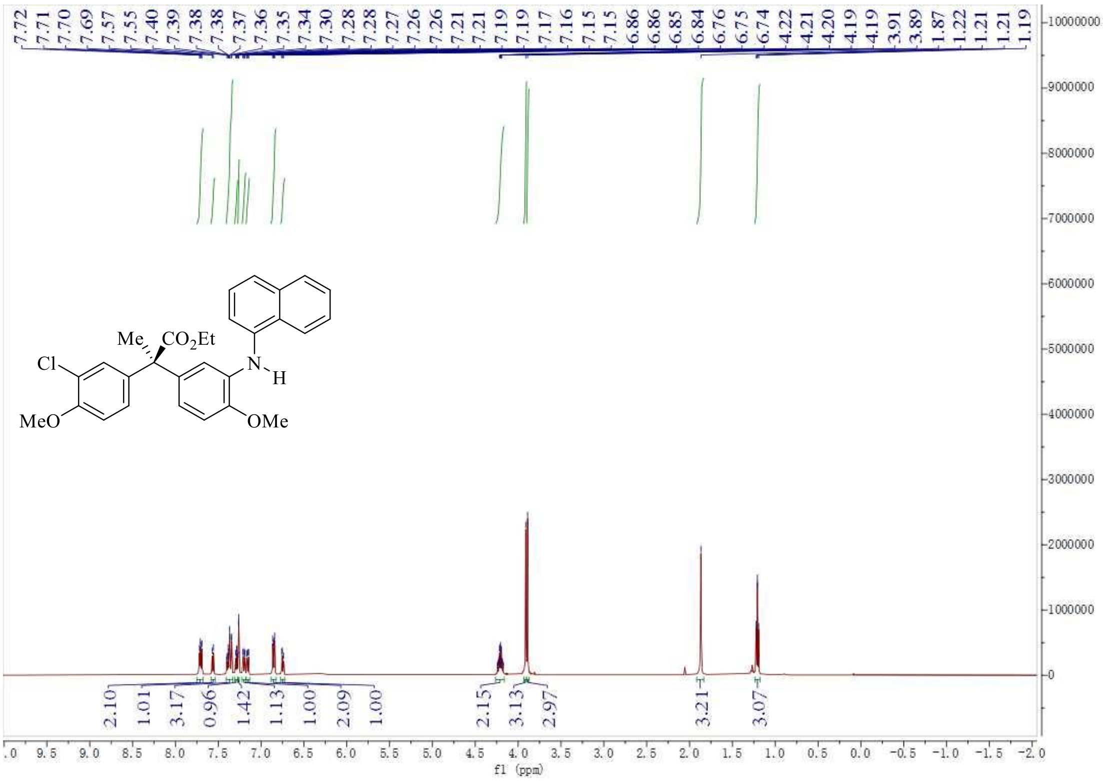

7.72 7.71 7.70 7.69 7.57 7.55 7.40 7.39 7.38 7.38 7.37 7.36 7.35 7.34 7.30 7.28 7.28 7.27 7.26 7.26 7.21 7.21 7.19 7.19 7.17 7.16 7.15 7.15 6.86 6.86 6.85 6.84 6.76 6.75 6.74 4.22 4.21 4.20 4.19 4.19 3.91 3.89 1.87 1.22 1.21 1.21 1.19
Me
CO2Et
Cl
MeO
N
H
OMe
2.10 1.01 3.17 0.96 1.42 1.13 1.00 2.09 1.00 2.15 3.13 2.97 3.21 3.07
f1 (ppm)

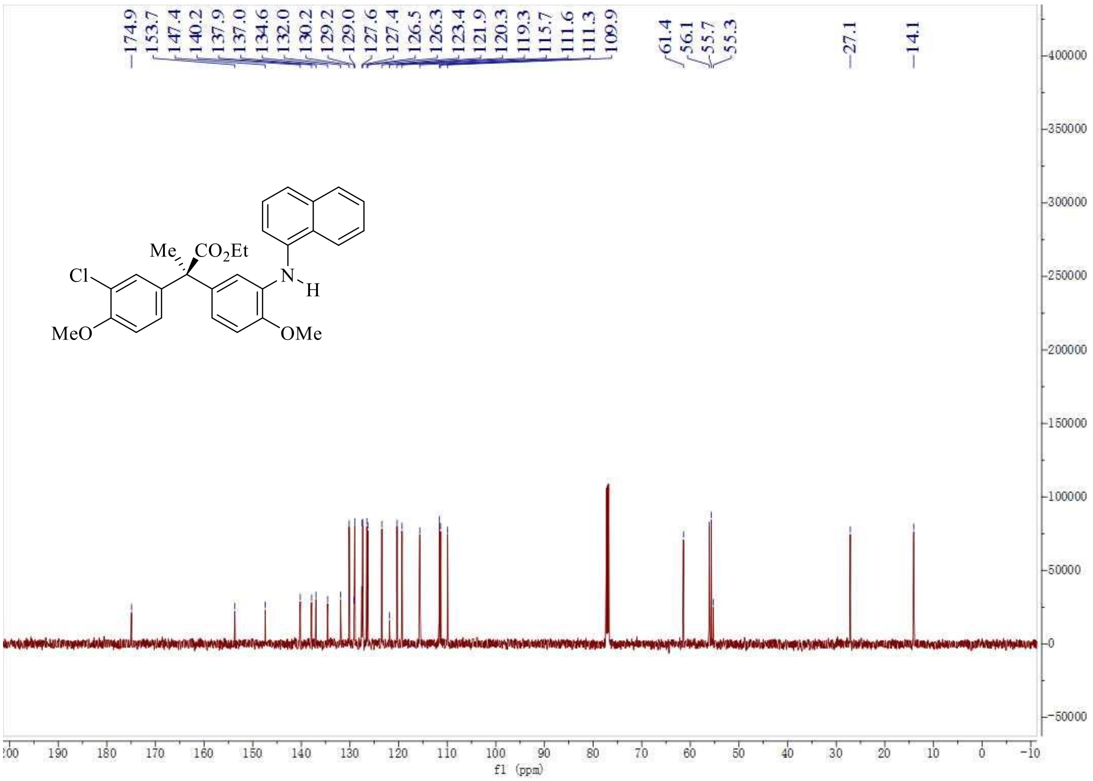

174.9 153.7 147.4 140.2 137.9 137.0 134.6 132.0 130.2 129.2 129.0 127.6 127.4 126.5 126.3 123.4 121.9 120.3 119.3 115.7 111.6 111.3 109.9 61.4 56.1 55.7 55.3 27.1 14.1
Me
CO2Et
Cl
MeO
N
H
OMe
f1 (ppm)

71

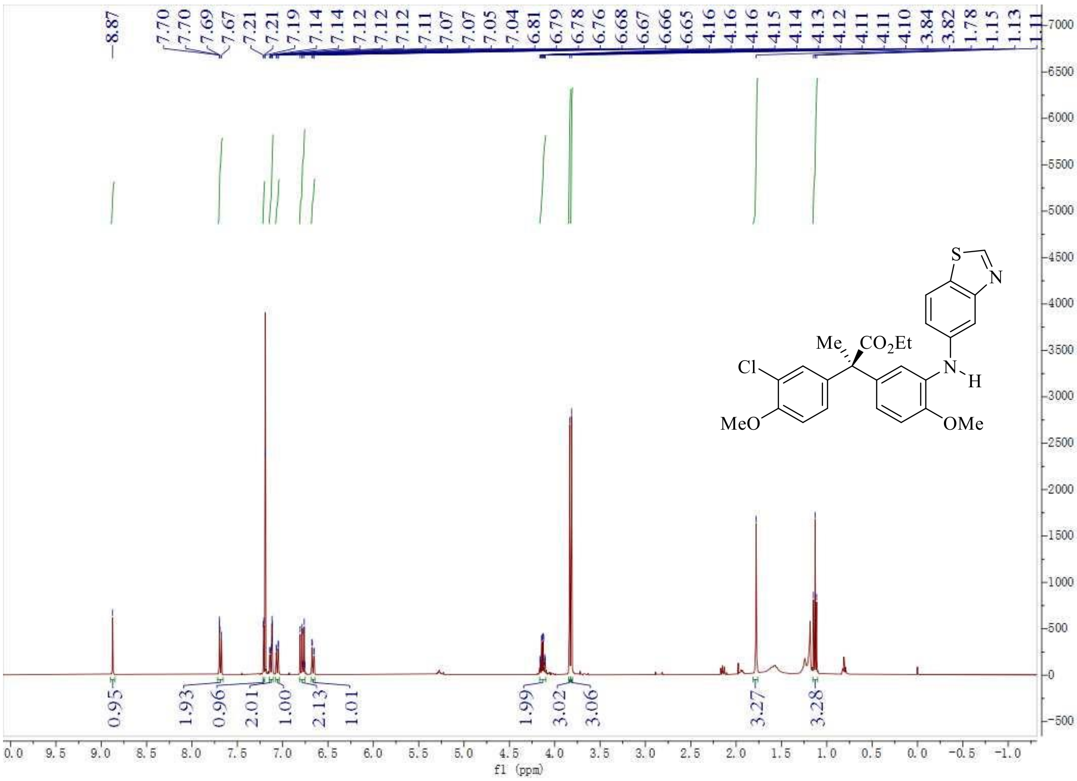
8.87
7.70
7.70
7.69
7.67
7.21
7.21
7.19
7.14
7.14
7.12
7.12
7.12
7.11
7.07
7.07
7.05
7.04
6.81
6.79
6.78
6.76
6.68
6.67
6.66
6.65
4.16
4.16
4.16
4.15
4.14
4.13
4.12
4.11
4.11
4.10
3.84
3.82
1.78
1.15
1.13
1.11
0.95
1.93
0.96
2.01
1.00
2.13
1.01
1.99
3.02
3.06
3.27
3.28
Me
CO2Et
Cl
MeO
OMe
N
H
S
N
f1 (ppm)

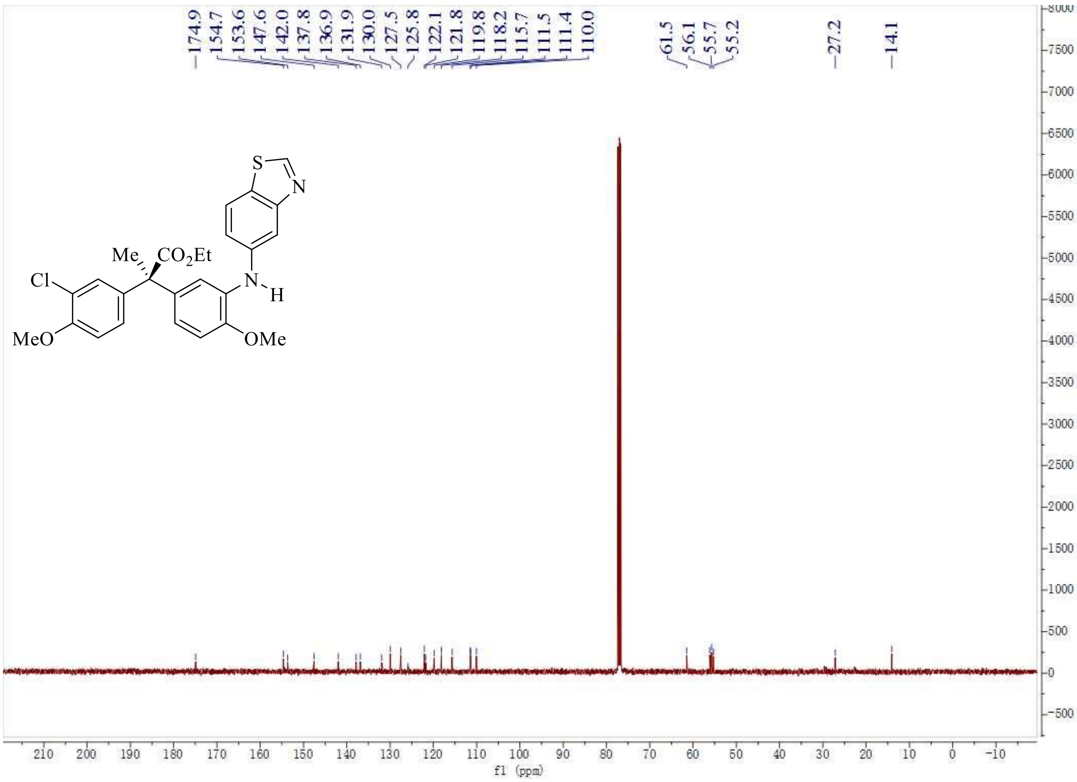
174.9
154.7
153.6
147.6
142.0
137.8
136.9
131.9
130.0
127.5
125.8
122.1
121.8
119.8
118.2
115.7
111.5
111.4
110.0
61.5
56.1
55.7
55.2
27.2
14.1
Me
CO2Et
Cl
MeO
OMe
N
H
S
N
f1 (ppm)

75

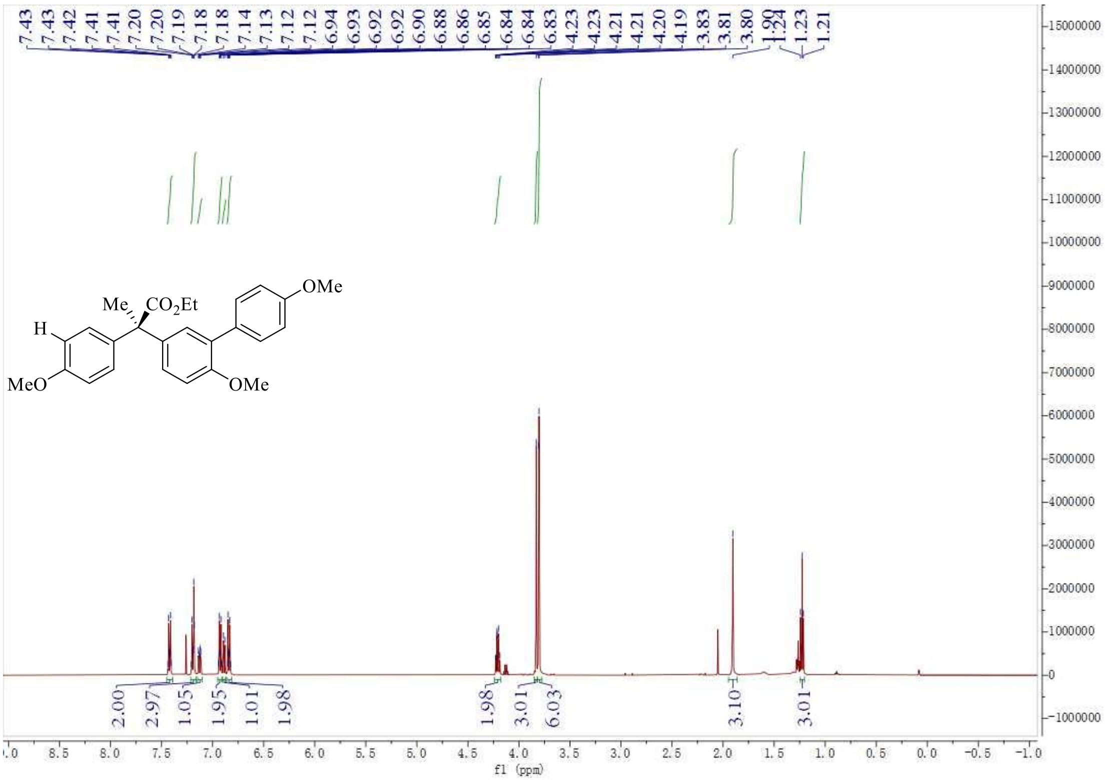

7.43
7.43
7.42
7.41
7.41
7.20
7.20
7.19
7.18
7.18
7.14
7.13
7.12
7.12
6.94
6.93
6.92
6.92
6.90
6.88
6.86
6.85
6.84
6.84
6.83
4.23
4.23
4.21
4.21
4.20
4.19
3.83
3.81
3.80
1.90
1.24
1.23
1.21
Me
CO2Et
OMe
H
MeO
OMe
2.00
2.97
1.05
1.95
1.01
1.98
1.98
3.01
6.03
3.10
3.01
f1 (ppm)

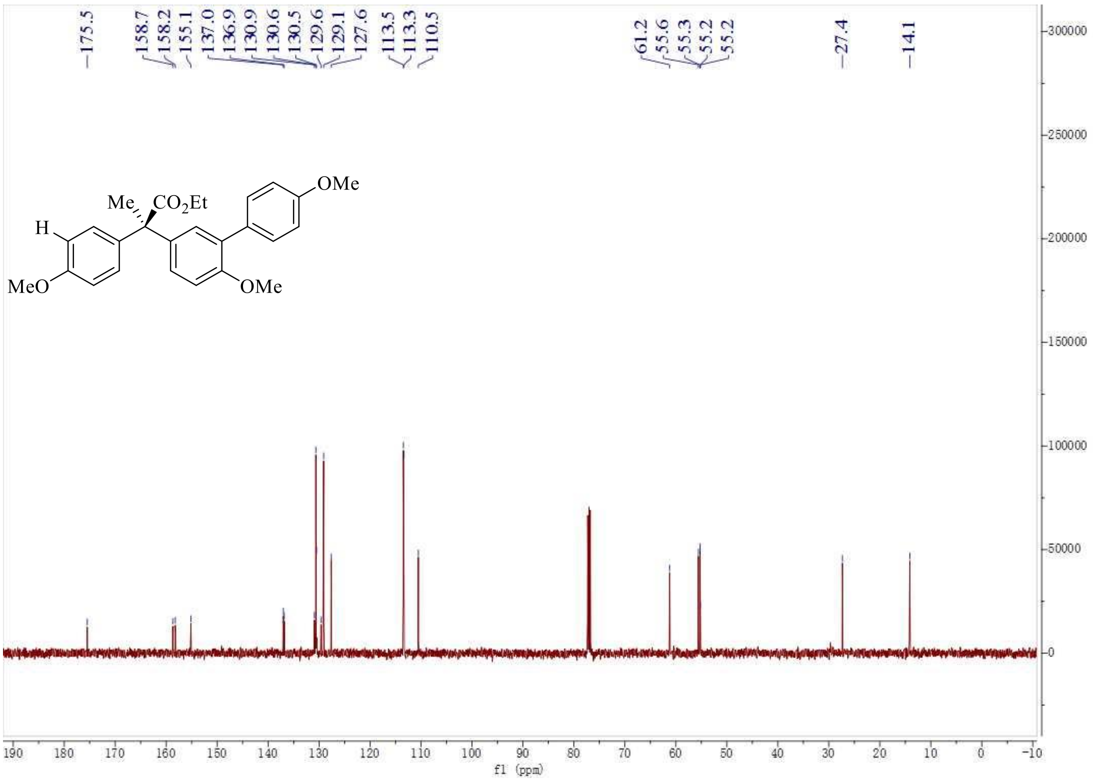

175.5
158.7
158.2
155.1
137.0
136.9
130.9
130.6
130.5
129.6
129.1
127.6
113.5
113.3
110.5
61.2
55.6
55.3
55.2
55.2
27.4
14.1
Me
CO2Et
OMe
H
MeO
OMe
f1 (ppm)

77

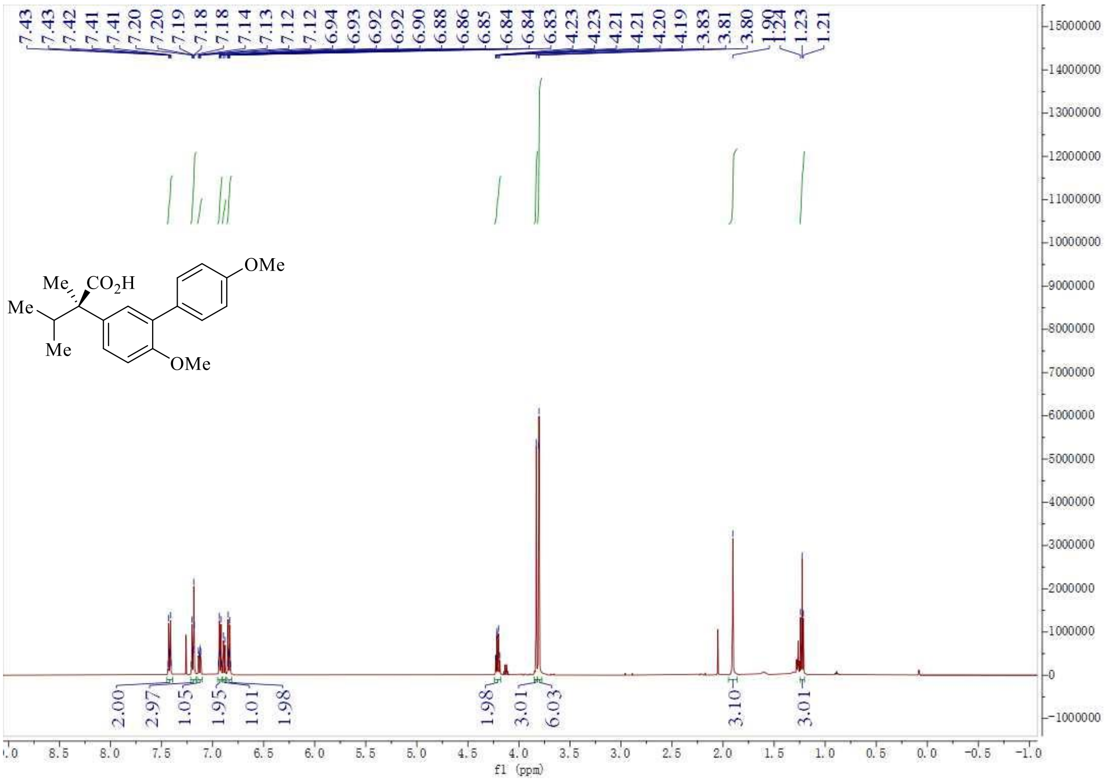

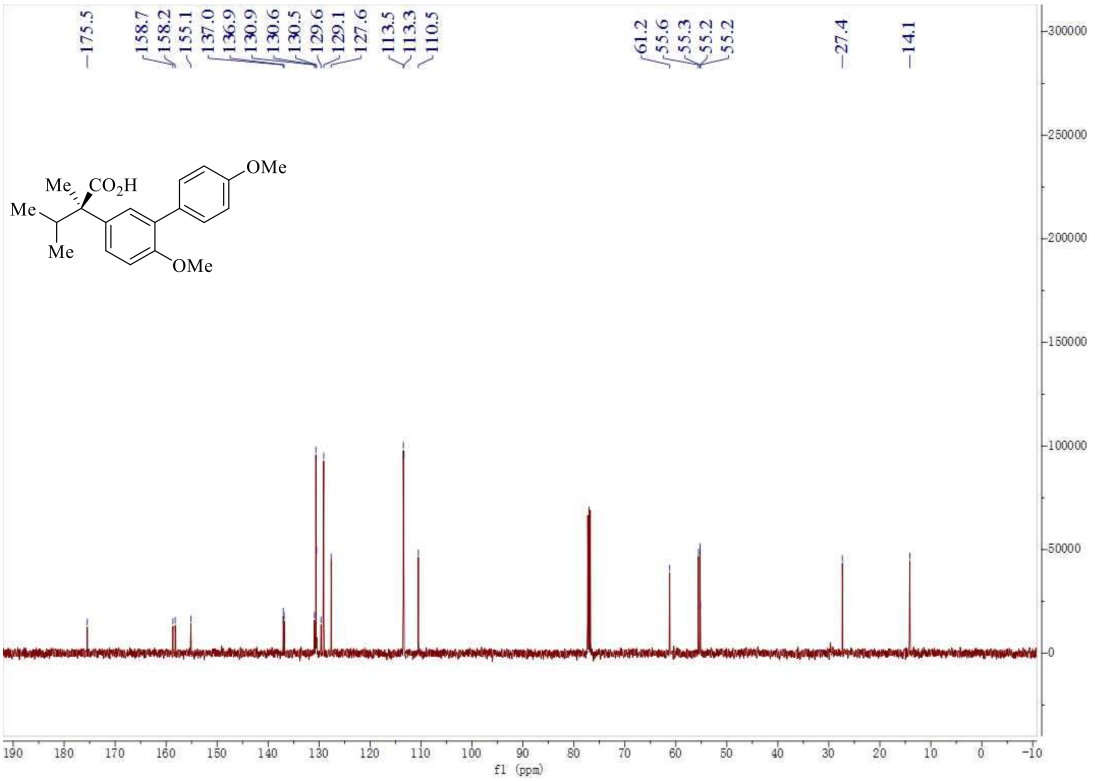

83

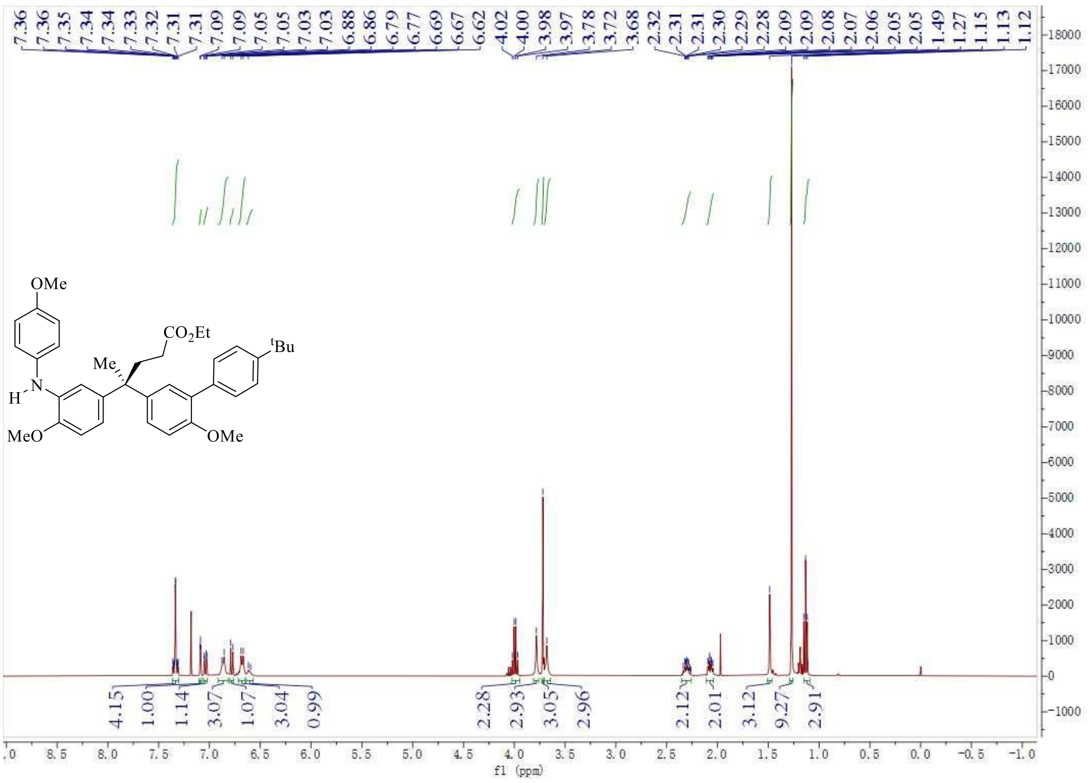

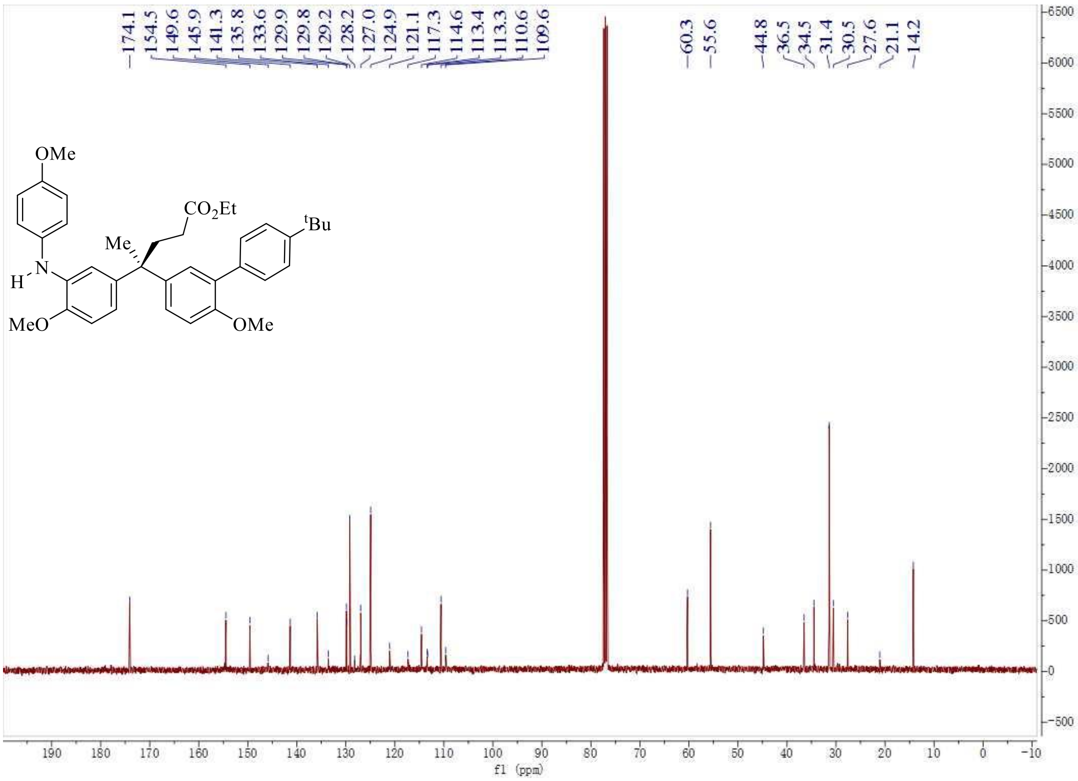

85

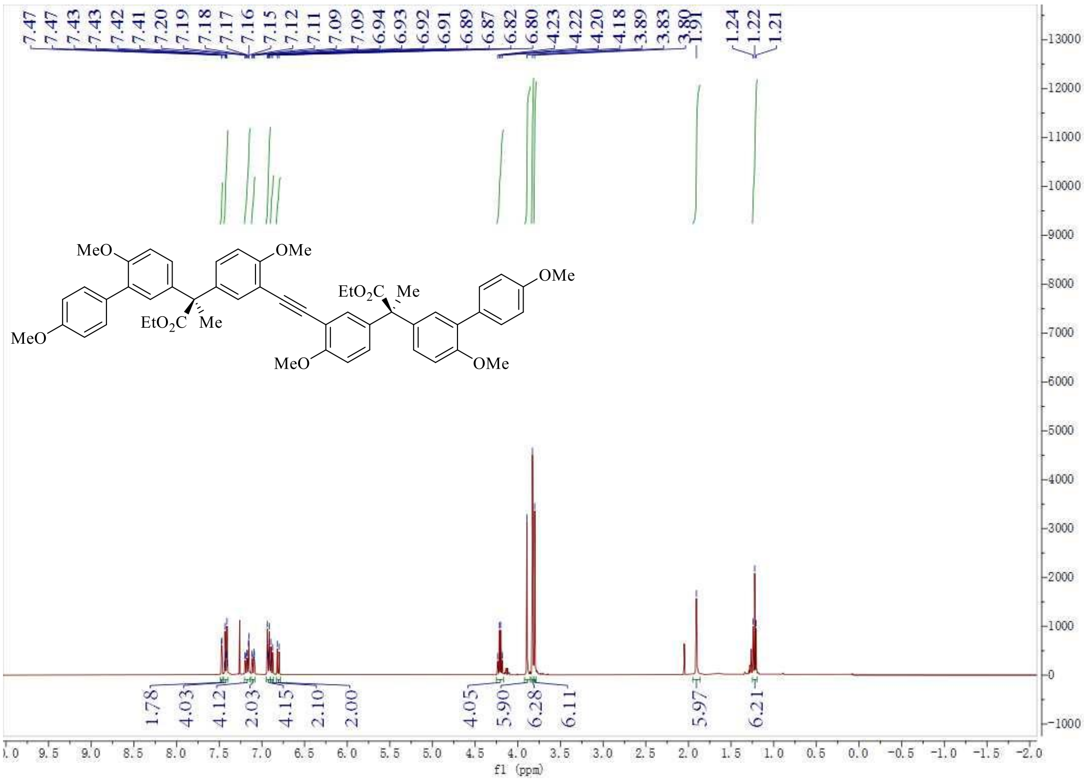
7.47
7.47
7.43
7.43
7.42
7.41
7.20
7.19
7.18
7.17
7.16
7.15
7.12
7.11
7.09
7.09
6.94
6.93
6.92
6.91
6.89
6.87
6.82
6.80
4.23
4.22
4.20
4.18
3.89
3.83
3.80
1.91
1.24
1.22
1.21
1.78
4.03
4.12
2.03
4.15
2.10
2.00
4.05
5.90
6.28
6.11
5.97
6.21
MeO
OMe
EtO2C
Me
OMe
MeO
EtO2C
Me
MeO
OMe
f1 (ppm)

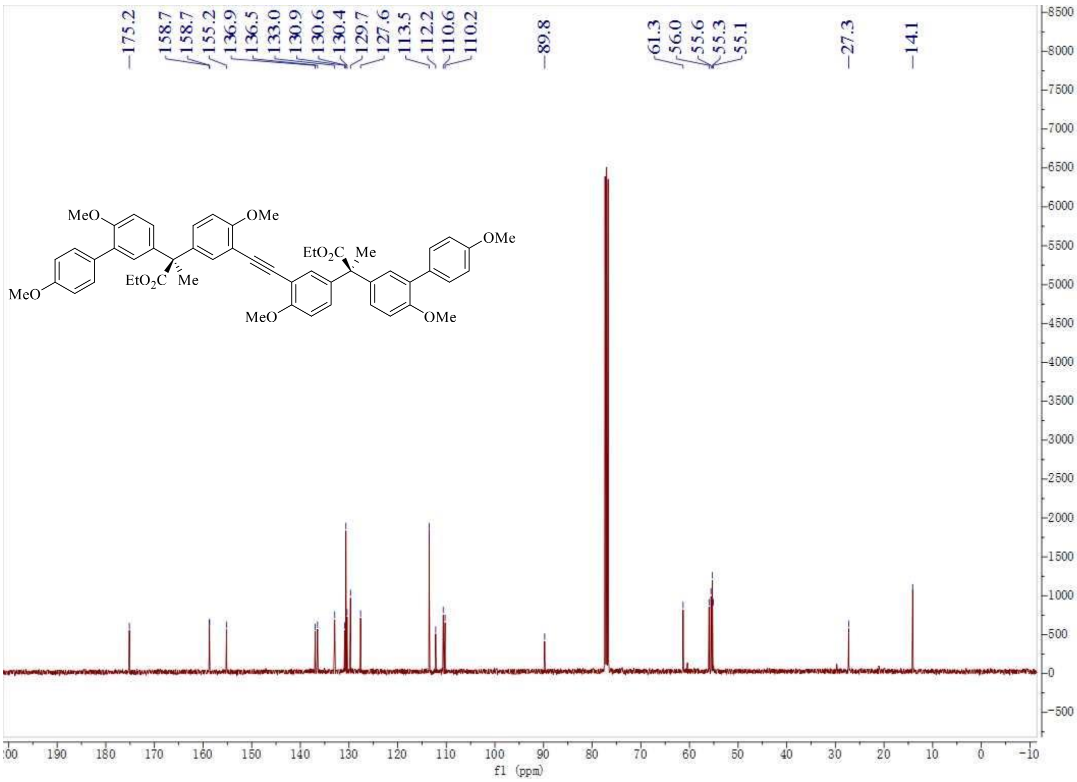
175.2
158.7
158.7
155.2
136.9
136.5
133.0
130.9
130.6
130.4
129.7
127.6
113.5
112.2
110.6
110.2
89.8
61.3
56.0
55.6
55.3
55.1
27.3
14.1
MeO
OMe
EtO2C
Me
OMe
MeO
EtO2C
Me
MeO
OMe
f1 (ppm)

86

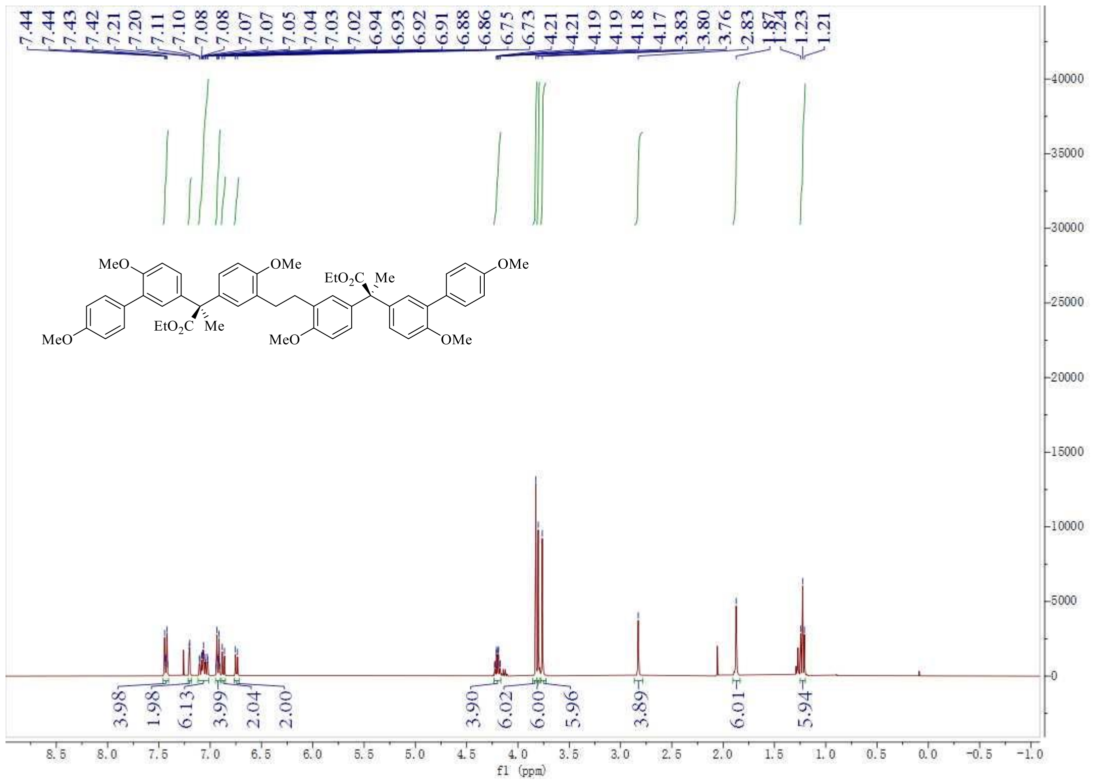

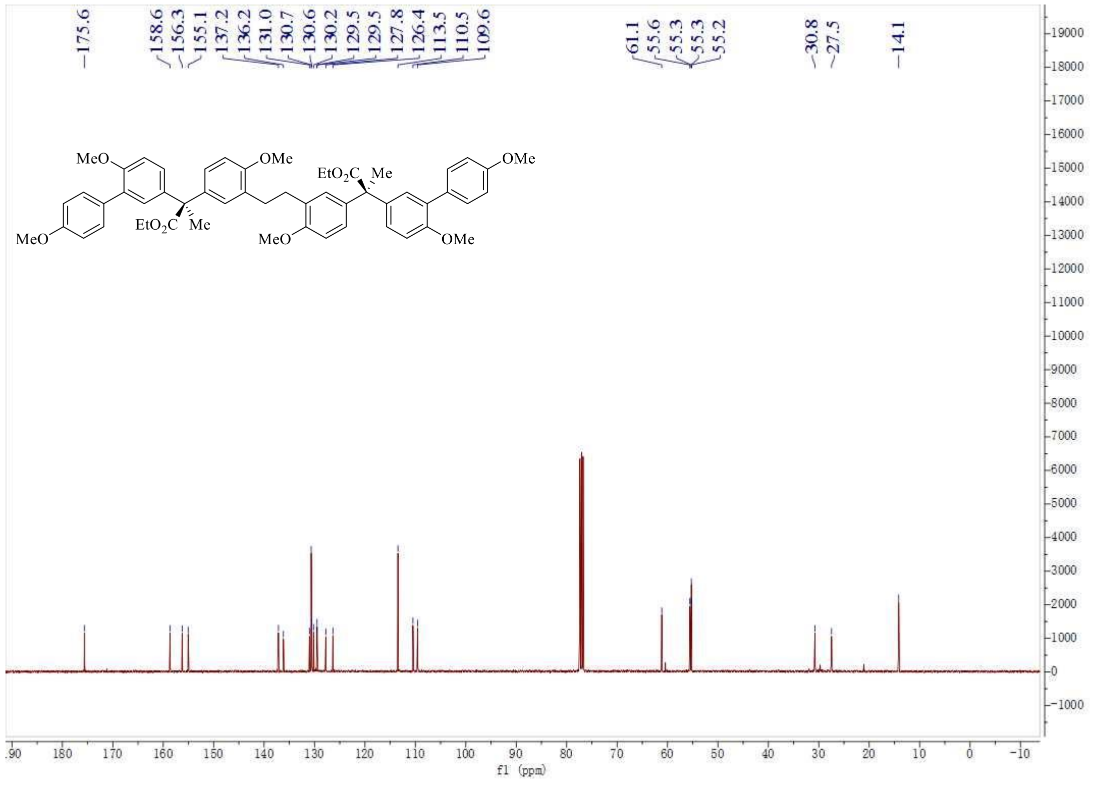

88

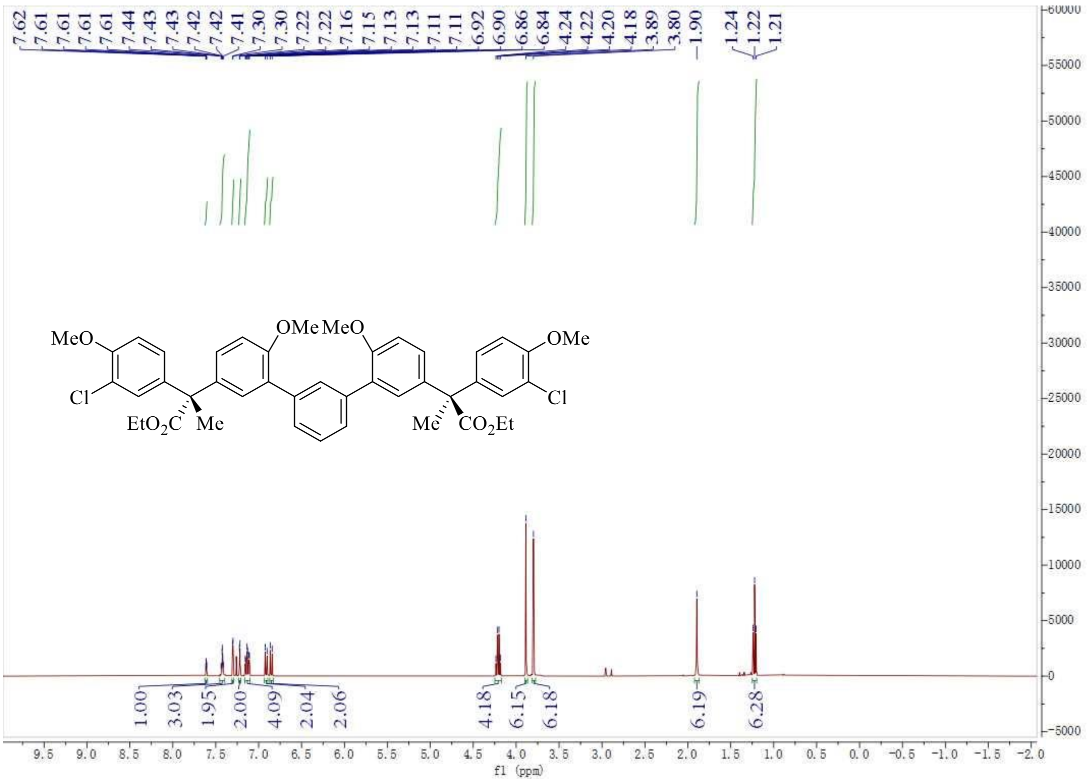

MeO
OMe MeO
OMe
Cl
Cl
EtO2C
Me
Me
CO2Et
f1 (ppm)

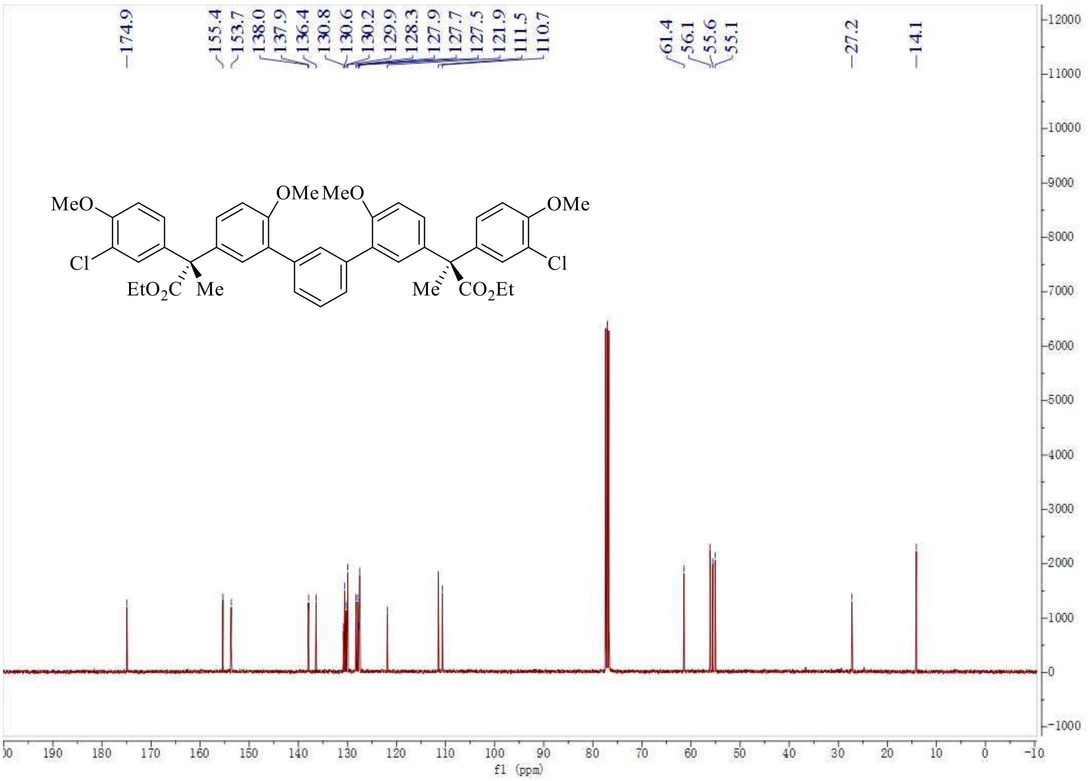

MeO
OMe MeO
OMe
Cl
Cl
EtO2C
Me
Me
CO2Et
f1 (ppm)

90

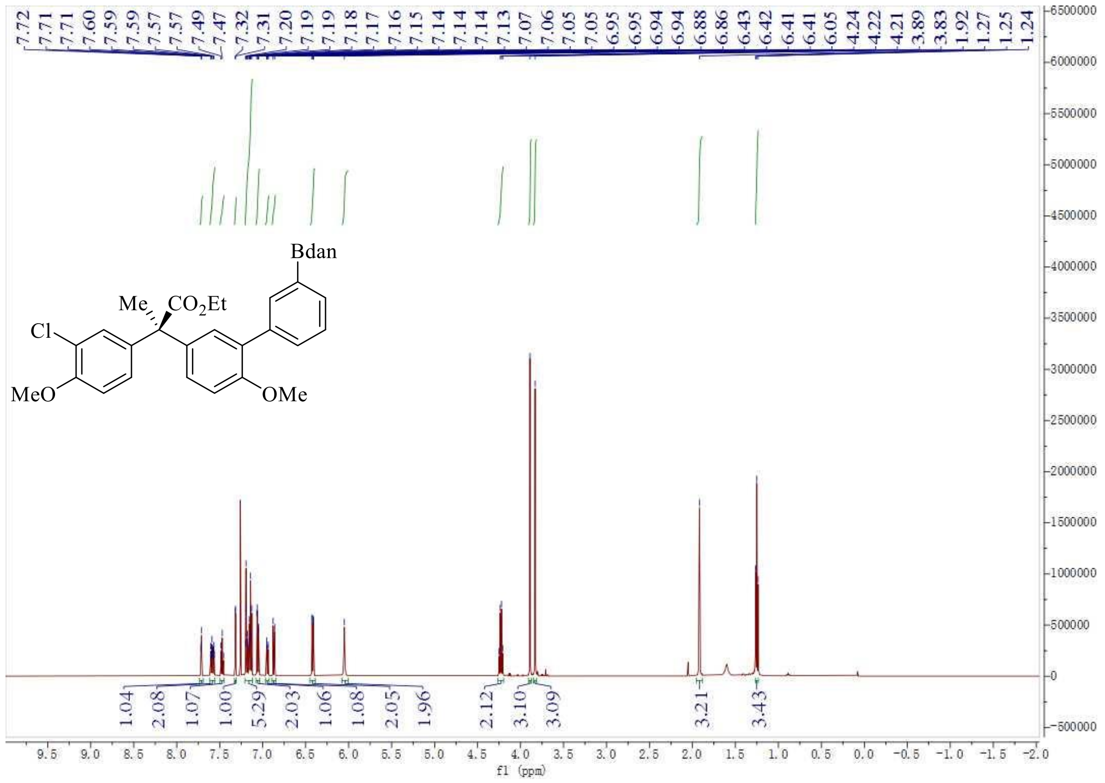

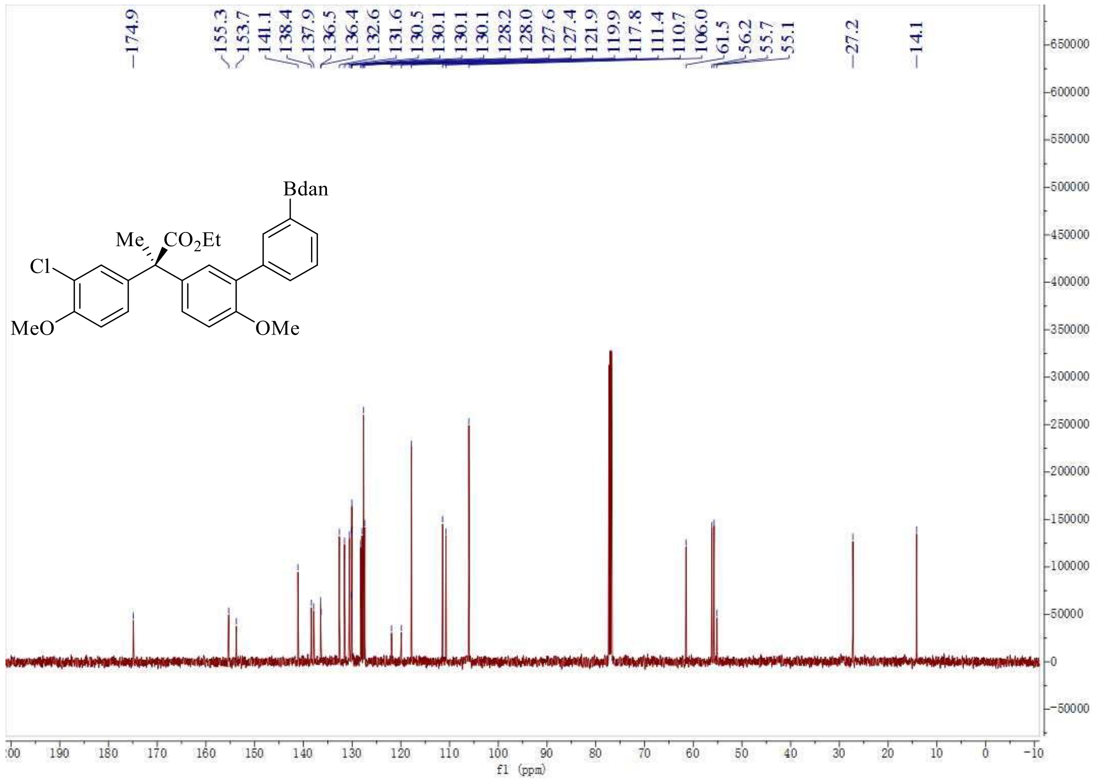

91

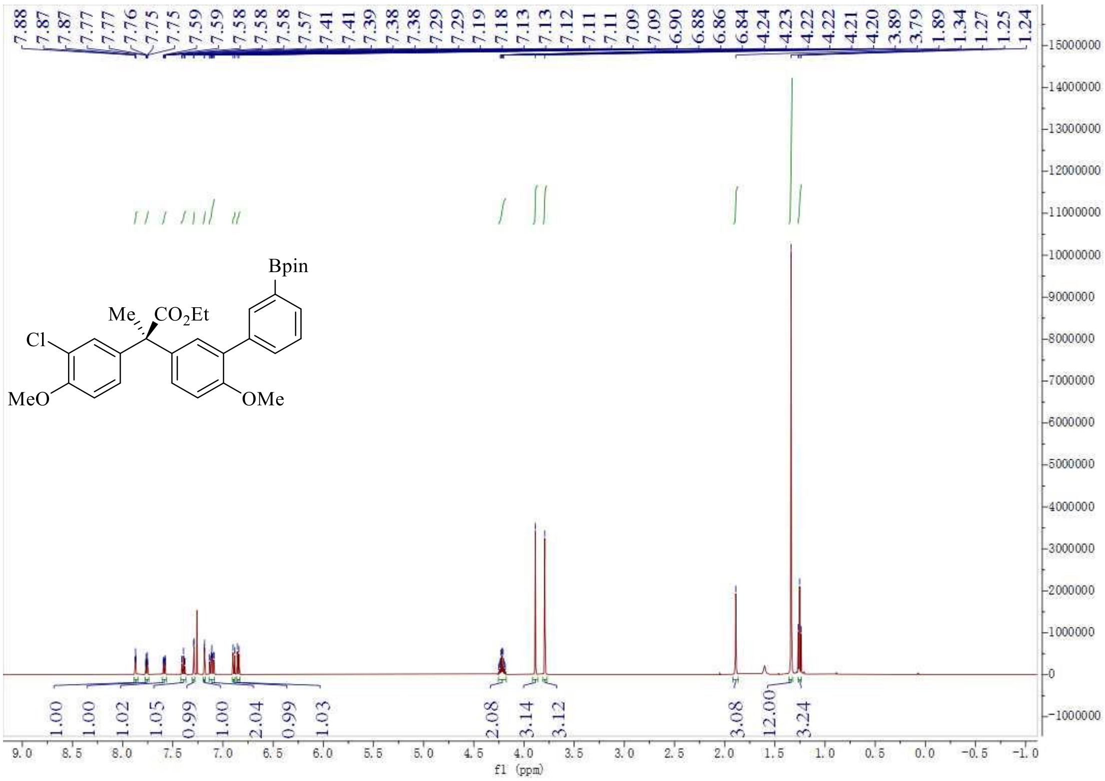

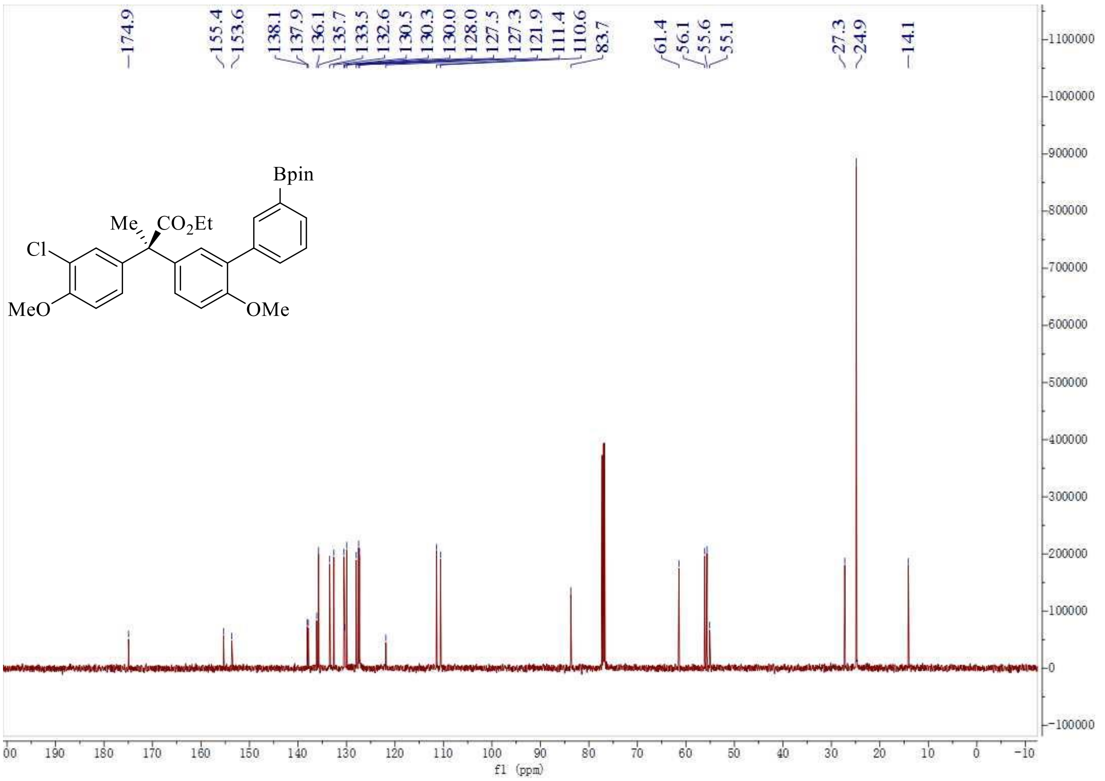

93

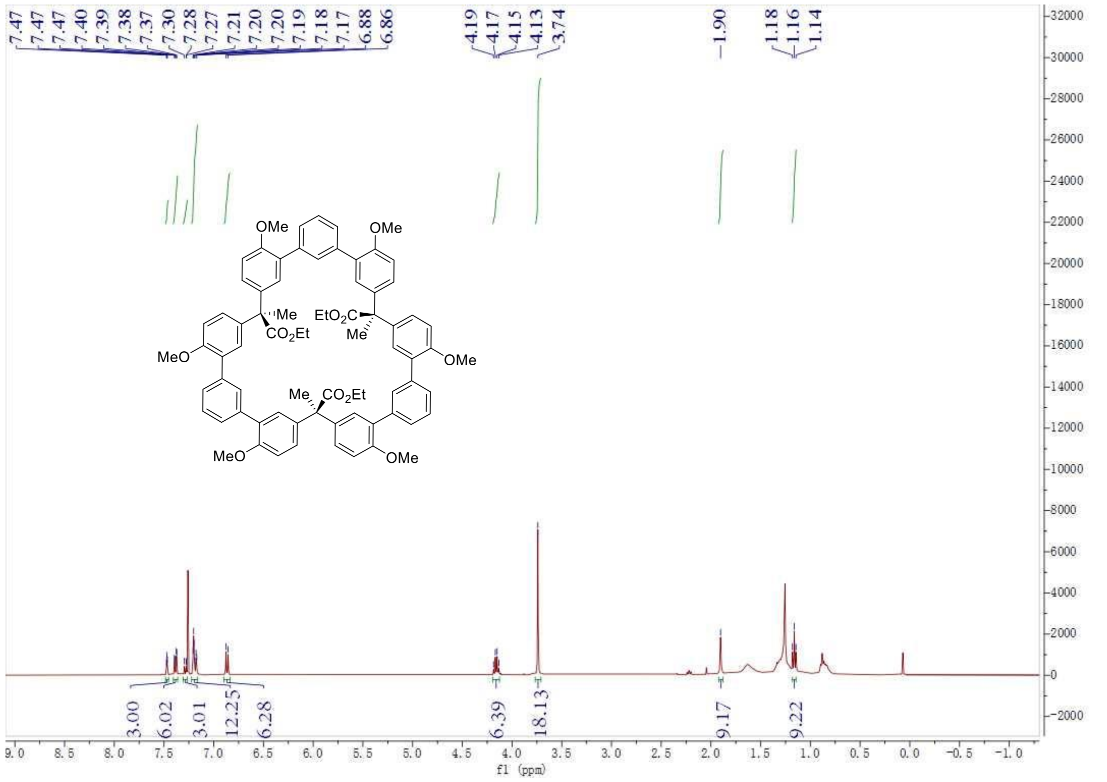

7.47 7.47 7.47 7.40 7.39 7.38 7.37 7.30 7.28 7.27 7.21 7.20 7.19 7.18 7.17 6.88 6.86
4.19 4.17 4.15 4.13 3.74
1.90
1.18 1.16 1.14
OMe
MeO
Me
CO2Et
EtO2C
3.00 6.02 3.01 12.25 6.28
6.39 18.13
9.17 9.22
f1 (ppm)

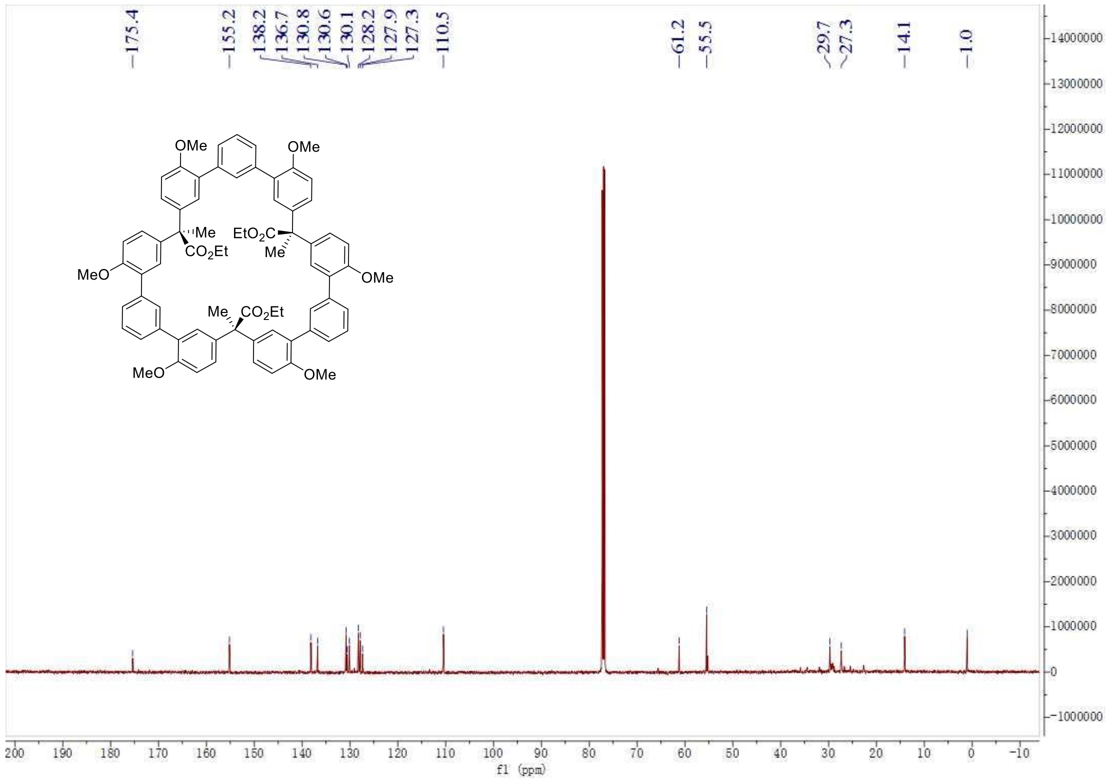

175.4
155.2
138.2 136.7 130.8 130.6 130.1 128.2 127.9 127.3
110.5
61.2 55.5
29.7 27.3
14.1
1.0
OMe
MeO
Me
CO2Et
EtO2C
f1 (ppm)

95

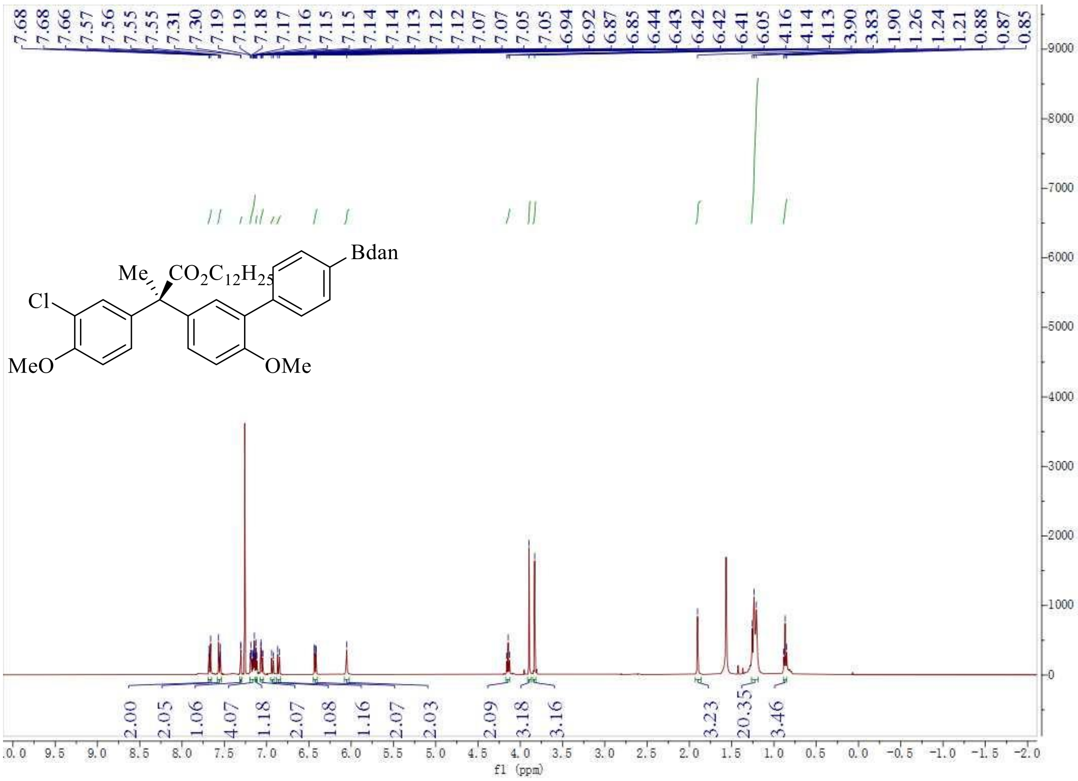

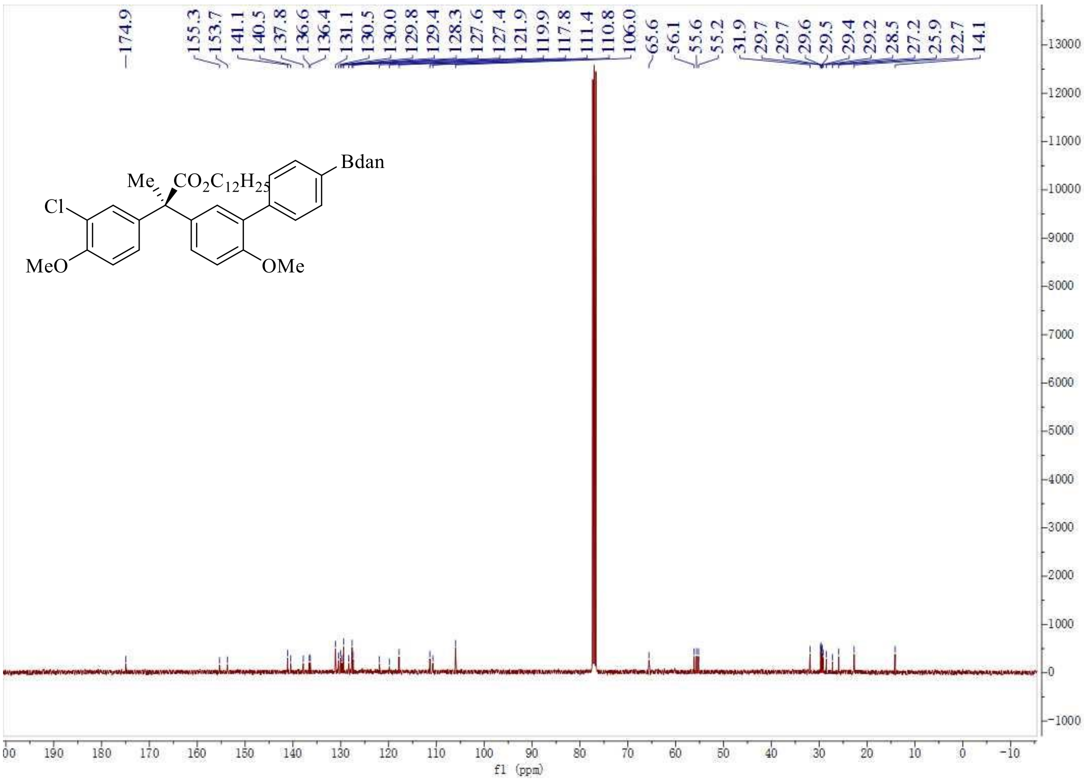

96

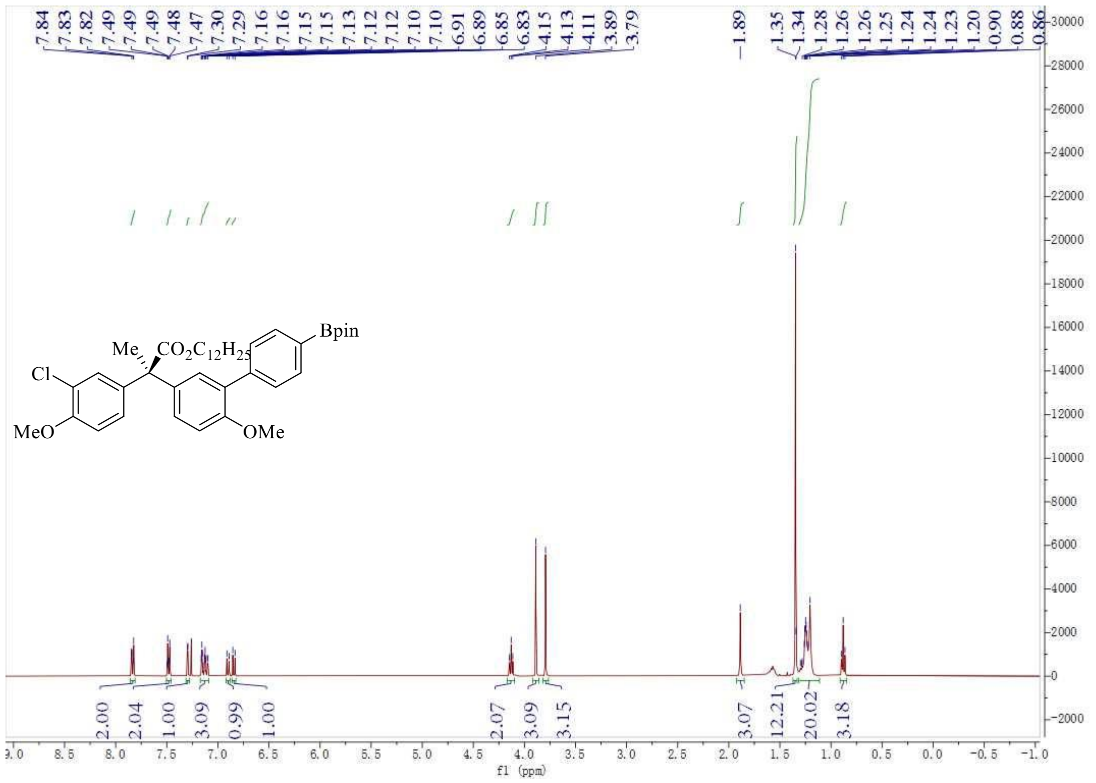

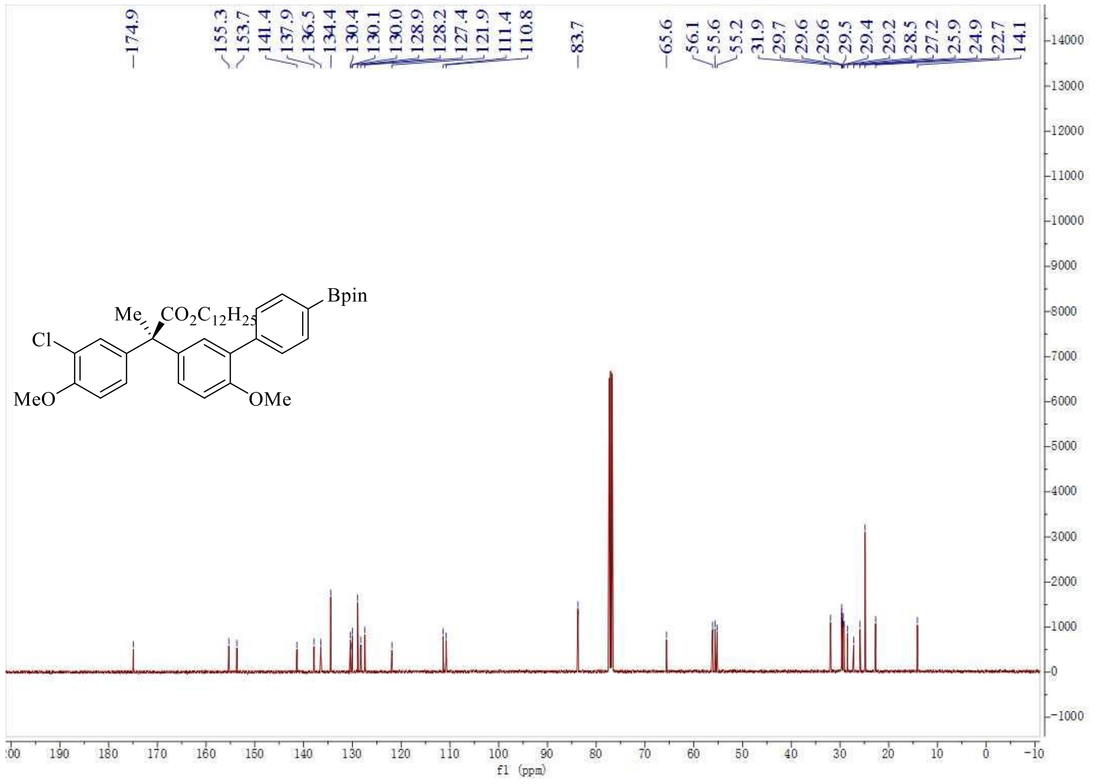

92

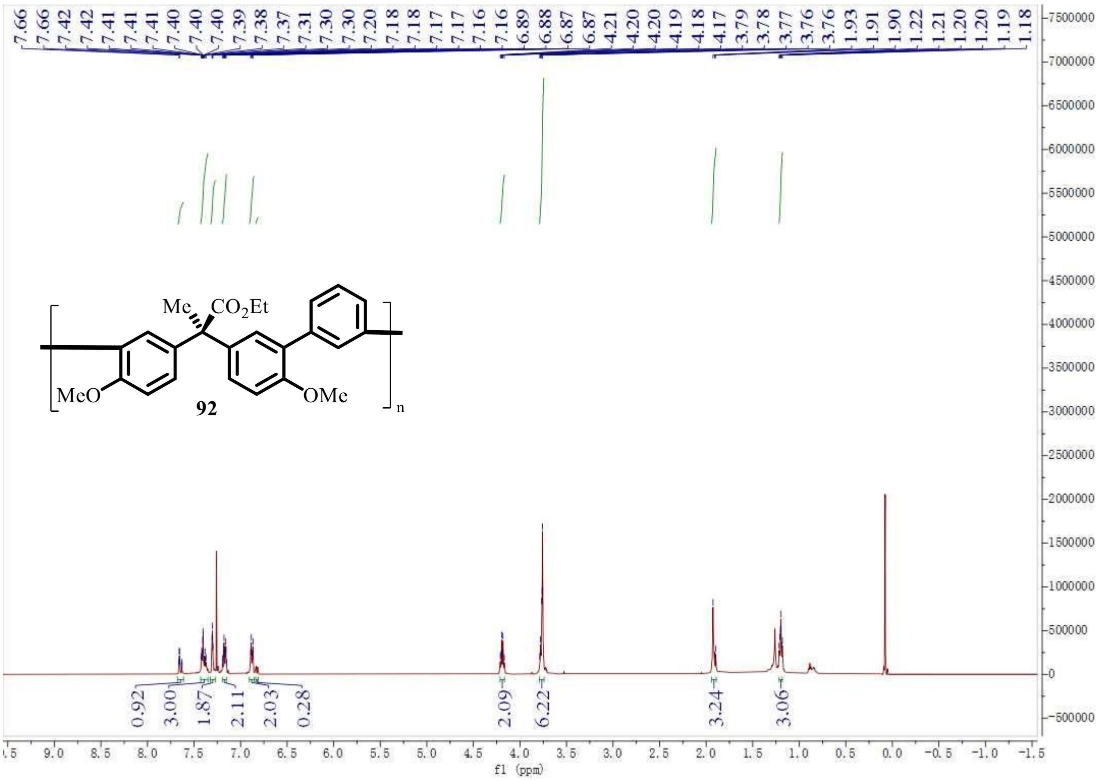

97

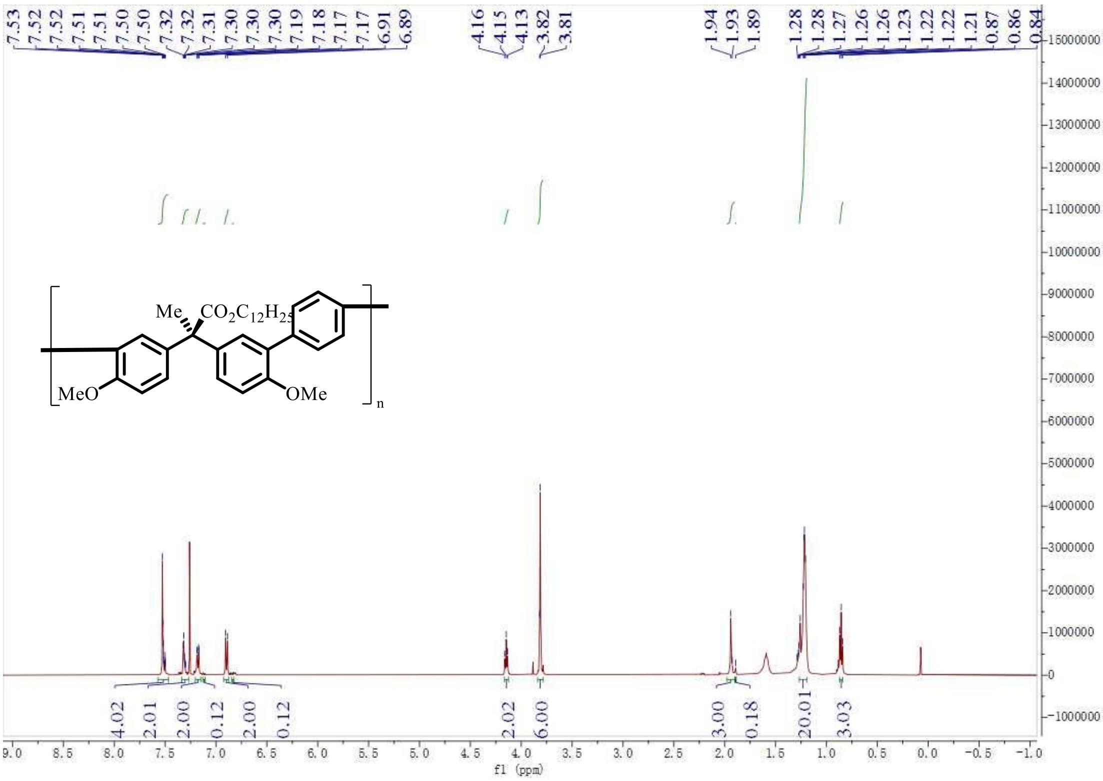

HPLC traces

1 (Me ester)

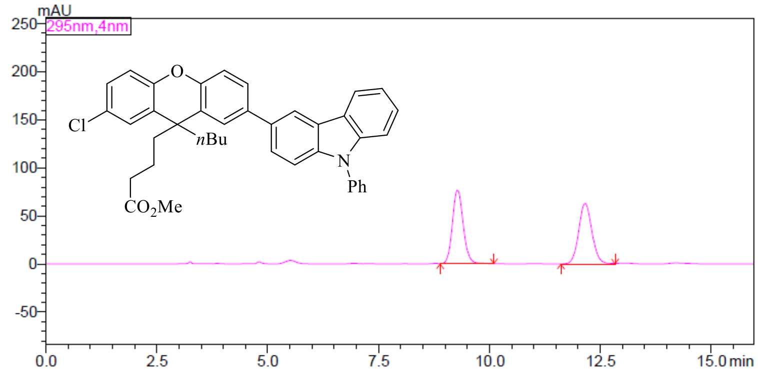

:Peak Table>

'DA Ch1 295nm

'eak#	Ret. Time	Area	Height	Area%	Peak Start	Peak End
1	9.276	1308469	76580	49.955	8.885	10.101
2	12.155	1310834	63049	50.045	11.611	12.832
Total		2619304	139629	100.000		

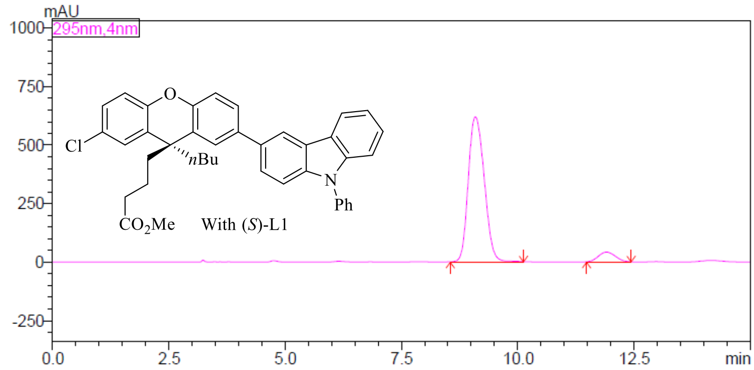

<Peak Table>

PDA Ch1 295nm

Peak#	Ret. Time	Area	Height	Area%	Peak Start	Peak End
1	9.088	14901541	620909	94.417	8.549	10.123
2	11.907	881067	38352	5.583	11.483	12.437
Total		15782608	659261	100.000		

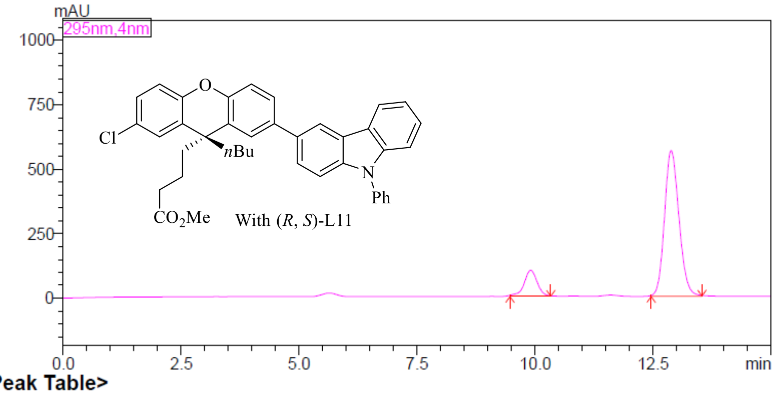

<Peak Table>

PDA Ch1 295nm

Peak#	Ret. Time	Area	Height	Area%	Peak Start	Peak End
1	9.913	1972647	101315	14.026	9.477	10.341
2	12.891	12091554	565671	85.974	12.469	13.547
Total		14064201	666986	100.000		

The major enantiomers of the two reactions are opposite.

2

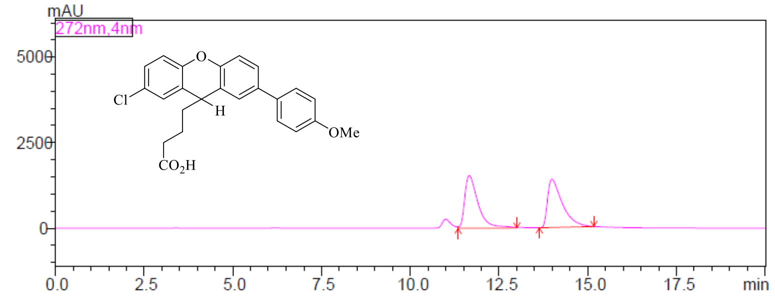

<Peak Table>

PDA Ch1 272nm

Peak#	Ret. Time	Area	Height	Area%	Peak Start	Peak End
1	11.663	40031012	1535173	49.293	11.344	13.003
2	13.993	41179467	1413943	50.707	13.637	15.173
Total		81210479	2949116	100.000		

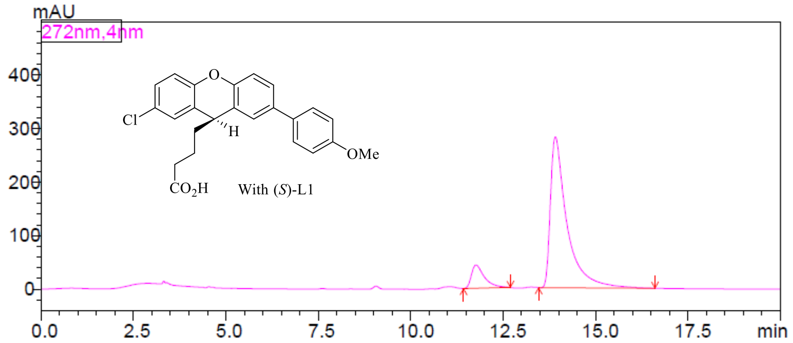

<Peak Table>

PDA Ch1 272nm

Peak#	Ret. Time	Area	Height	Area%	Peak Start	Peak End
1	11.768	1052595	43412	10.692	11.429	12.699
2	13.916	8791687	281954	89.308	13.461	16.613
Total		9844282	325366	100.000		

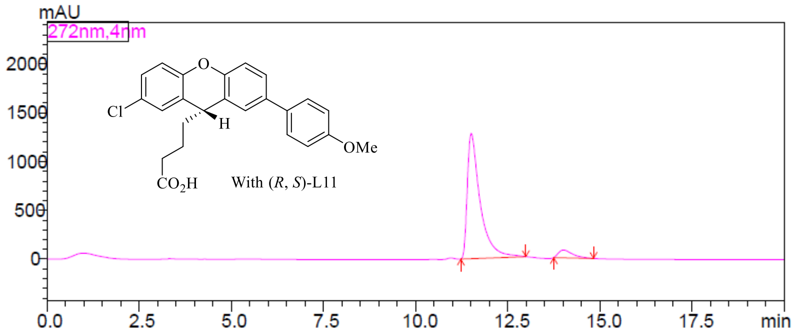

<Peak Table>

PDA Ch1 272nm

Peak#	Ret. Time	Area	Height	Area%	Peak Start	Peak End
1	11.512	31142207	1282934	93.724	11.227	12.992
2	14.016	2085388	78865	6.276	13.749	14.832
Total		33227595	1361799	100.000		

The major enantiomers of the two reactions are opposite.

4 (as ethyl ester)

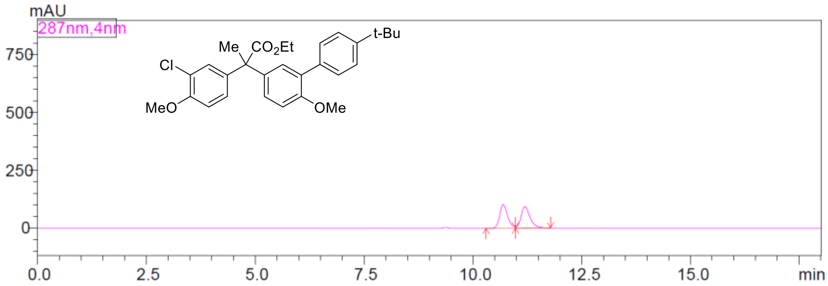

<Peak Table>

PDA Ch1 287nm

Peak#	Ret. Time	Area	Height	Area%	Peak Start	Peak End
1	10.700	1296045	102430	49.090	10.304	10.981
2	11.195	1344111	93746	50.910	10.981	11.787
Total		2640155	196175	100.000		

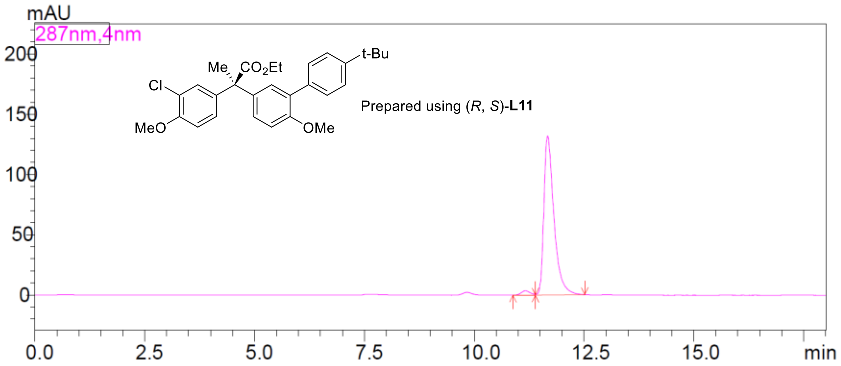

<Peak Table>

PDA Ch1 287nm

Peak#	Ret. Time	Area	Height	Area%	Peak Start	Peak End
1	11.165	48556	3487	2.258	10.880	11.392
2	11.671	2102282	131956	97.742	11.392	12.517
Total		2150838	135443	100.000		

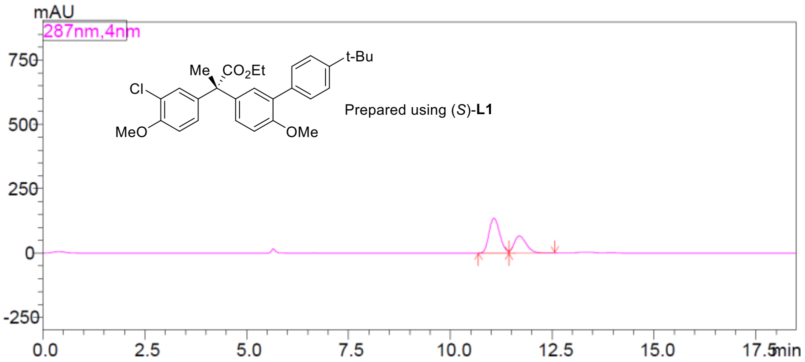

<Peak Table>

PDA Ch1 287nm

Peak#	Ret. Time	Area	Height	Area%	Peak Start	Peak End
1	11.069	2490025	135237	64.490	10.688	11.435
2	11.692	1371075	66354	35.510	11.435	12.565
Total		3861100	201591	100.000		

The major enantiomers of the two reactions are opposite.

4 (as carboxylic acid) The HPLC traces show that the elution order of enantiomeric peaks of **4**-COOH is different from **4**-ethyl ester.

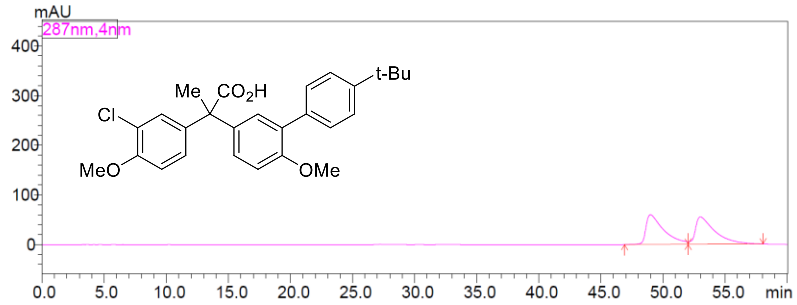

<Peak Table>

PDA Ch1 287nm

Peak#	Ret. Time	Area	Height	Area%	Peak Start	Peak End
1	48.988	5998383	59898	49.834	46.896	52.016
2	53.007	6038351	54963	50.166	52.016	58.053
Total		12036733	114861	100.000		

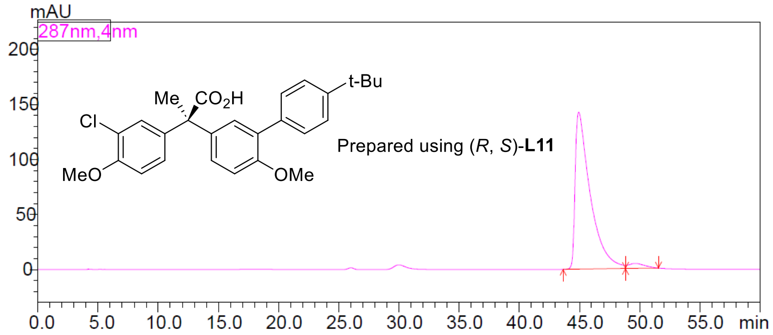

<Peak Table>

PDA Ch1 287nm

Peak#	Ret. Time	Area	Height	Area%	Peak Start	Peak End
1	44.935	12454512	142492	96.641	43.637	48.811
2	49.622	432852	4526	3.359	48.811	51.557
Total		12887364	147018	100.000		

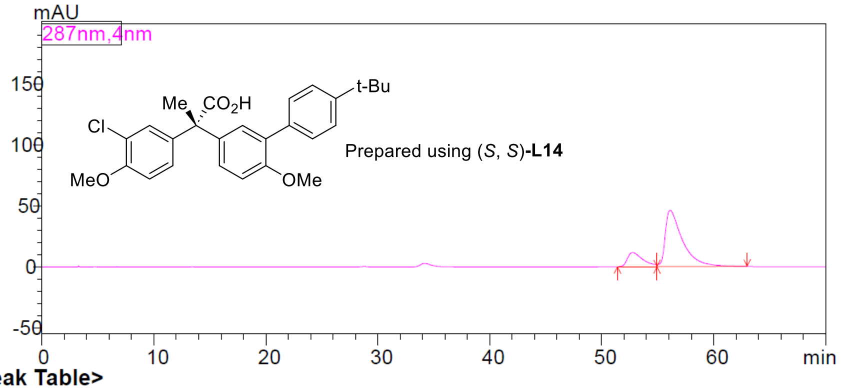

<Peak Table>

PDA Ch1 287nm

Peak#	Ret. Time	Area%	Peak Start	Peak End	Height%
1	52.756	18.178	51.392	54.928	20.098
2	56.106	81.822	54.928	62.976	79.902
Total		100.000			100.000

The major enantiomers of the two reactions are opposite.

7

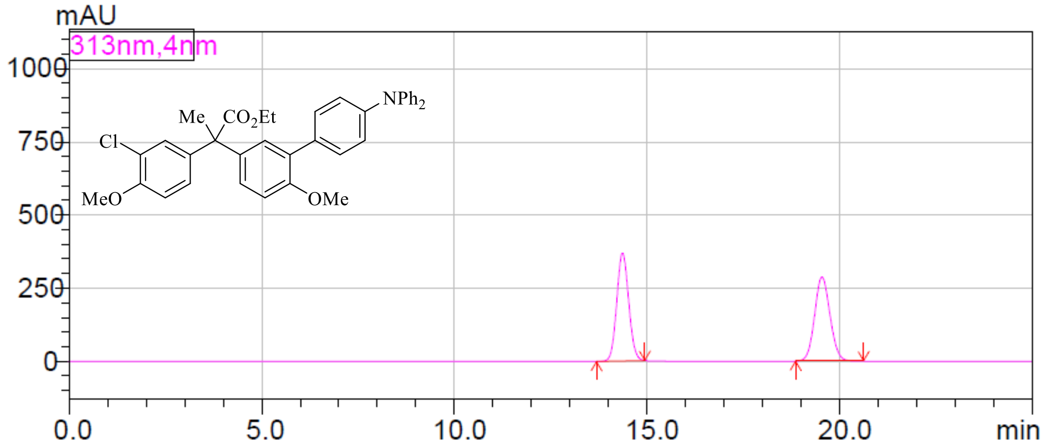

<Peak Table>

PDA Ch1 313nm

Peak#	Ret. Time	Area	Height	Area%	Peak Start	Peak End
1	14.368	7935780	368288	49.747	13.691	14.944
2	19.546	8016408	287621	50.253	18.864	20.624
Total		15952188	655909	100.000		

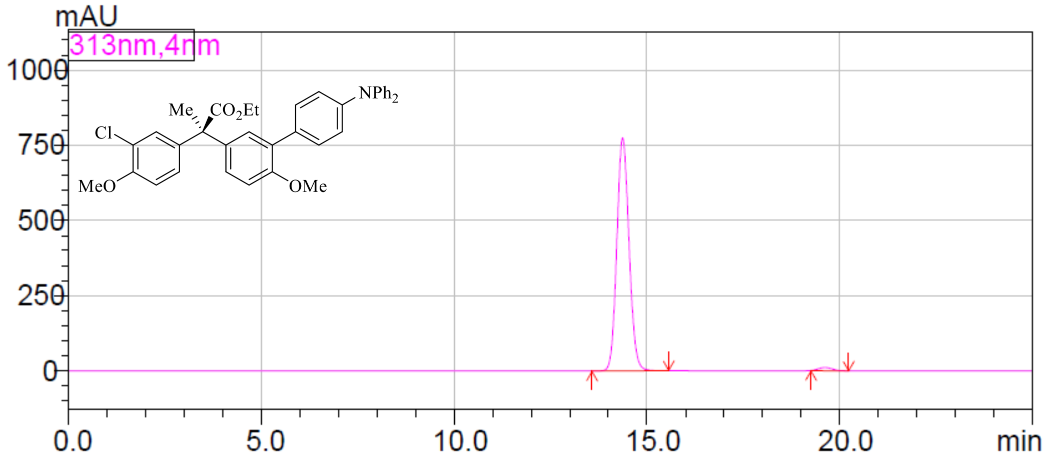

<Peak Table>

PDA Ch1 313nm

Peak#	Ret. Time	Area	Height	Area%	Peak Start	Peak End
1	14.370	17069159	774186	98.523	13.579	15.568
2	19.619	255816	10190	1.477	19.259	20.229
Total		17324975	784376	100.000		

8

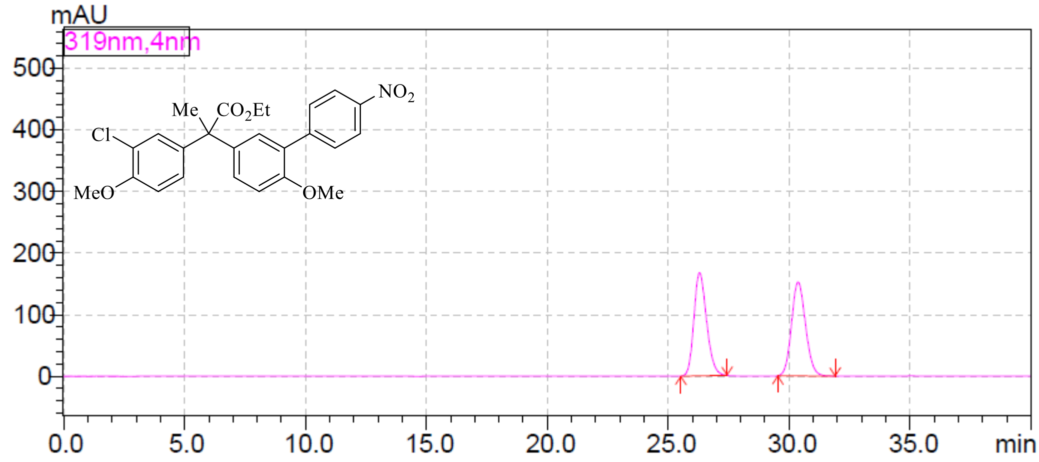

<Peak Table>

PDA Ch1 319nm

Peak#	Ret. Time	Area	Height	Area%	Peak Start	Peak End
1	26.293	6115989	167152	49.827	25.515	27.424
2	30.367	6158462	152341	50.173	29.520	31.893
Total		12274451	319492	100.000		

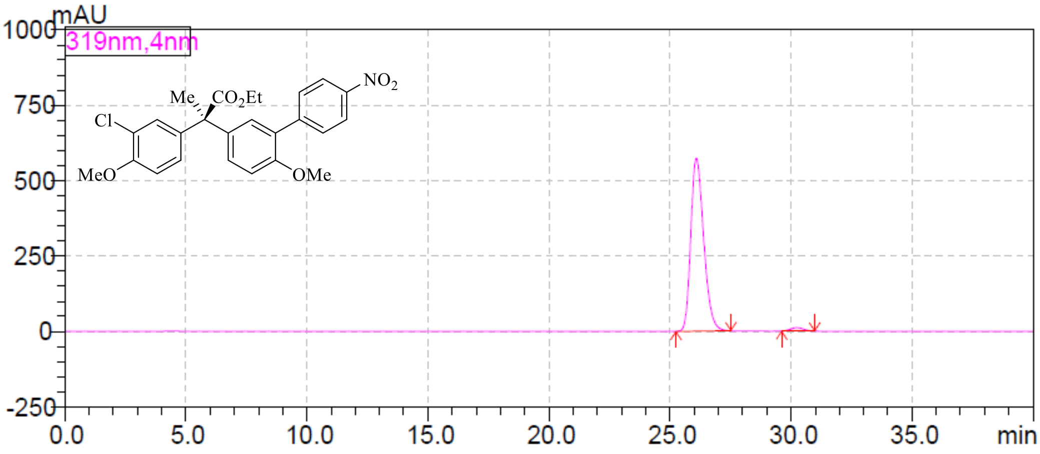

<Peak Table>

PDA Ch1 319nm

Peak#	Ret. Time	Area	Height	Area%	Peak Start	Peak End
1	26.089	21312905	573678	98.023	25.237	27.515
2	30.246	429857	11397	1.977	29.611	30.981
Total		21742761	585075	100.000		

9

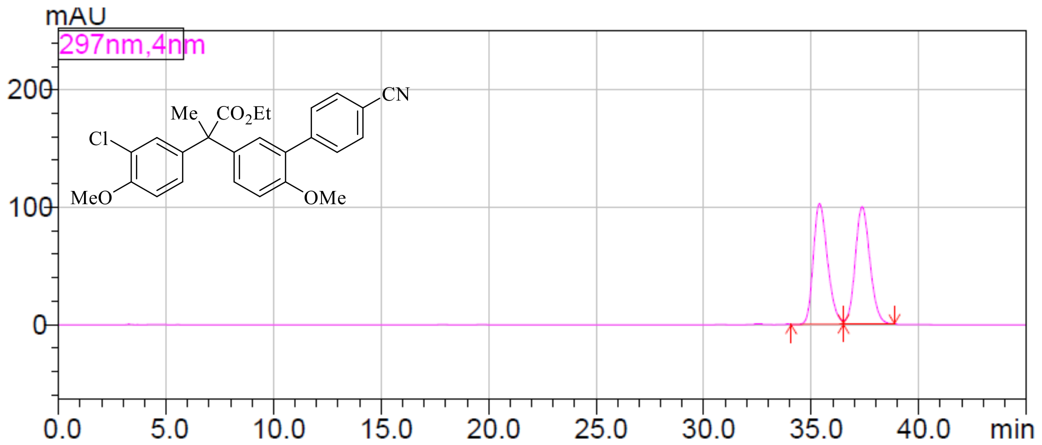

<Peak Table>

PDA Ch1 297nm

Peak#	Ret. Time	Area	Height	Mark	Area%	Peak Start	Peak End
1	35.388	4708974	102657	M	49.708	34.059	36.501
2	37.368	4764353	99862	V M	50.292	36.501	38.864
Total		9473328	202520		100.000		

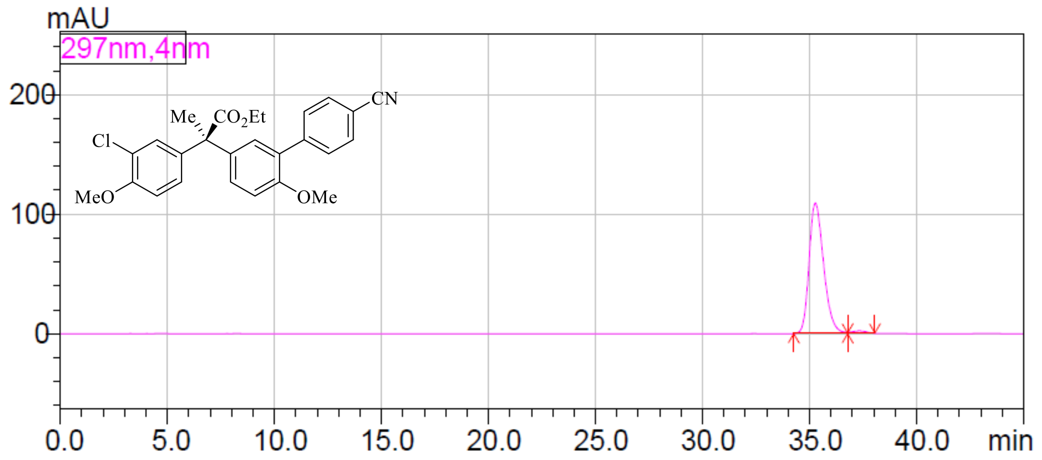

<Peak Table>

PDA Ch1 297nm

Peak#	Ret. Time	Area	Height	Area%	Peak Start	Peak End
1	35.268	5141247	109218	98.244	34.261	36.768
2	37.312	91901	2026	1.756	36.768	38.043
Total		5233148	111244	100.000		

10

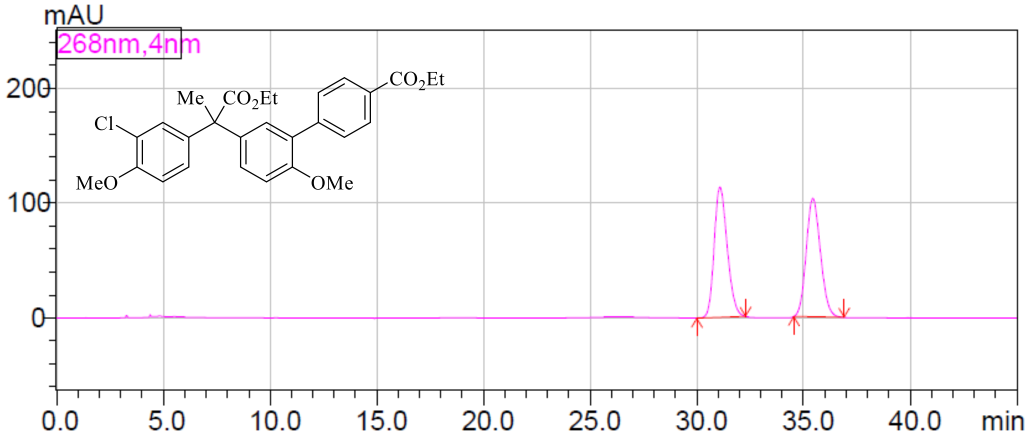

<Peak Table>

PDA Ch1 268nm

Peak#	Ret. Time	Area	Height	Area%	Peak Start	Peak End
1	31.082	4815224	113351	50.074	30.000	32.272
2	35.436	4801010	103184	49.926	34.544	36.907
Total		9616234	216536	100.000		

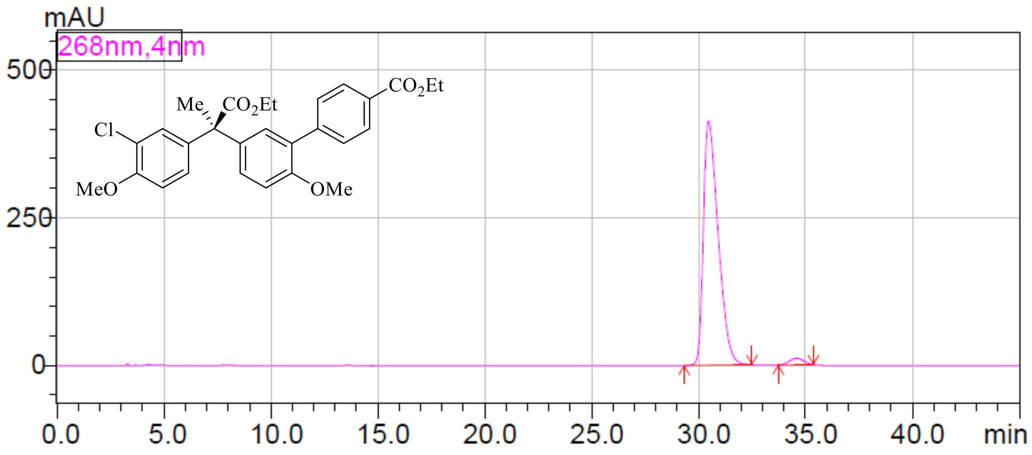

<Peak Table>

PDA Ch1 268nm

Peak#	Ret. Time	Area	Height	Area%	Peak Start	Peak End
1	30.468	19847507	413596	97.487	29.317	32.496
2	34.580	511594	11048	2.513	33.723	35.365
Total		20359101	424644	100.000		

11

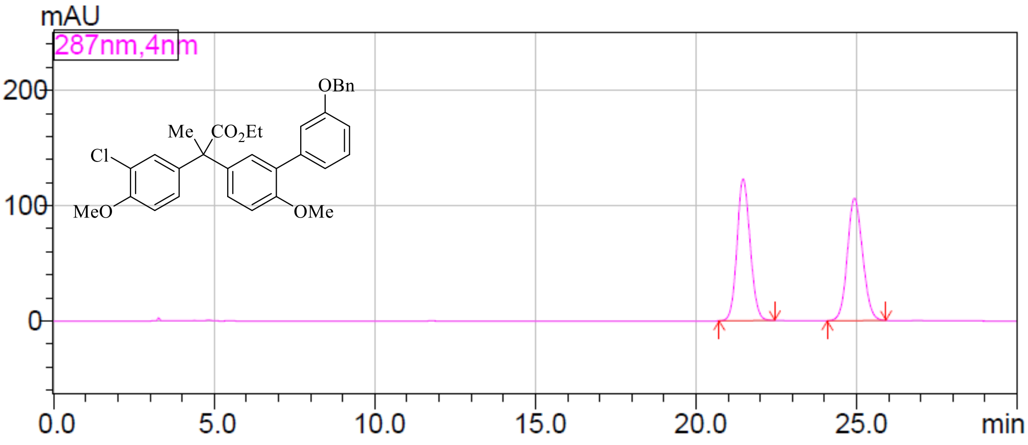

<Peak Table>

PDA Ch1 287nm

Peak#	Ret. Time	Area	Height	Area%	Peak Start	Peak End
1	21.468	3494414	122716	49.991	20.725	22.453
2	24.933	3495621	105998	50.009	24.091	25.909
Total		6990035	228714	100.000		

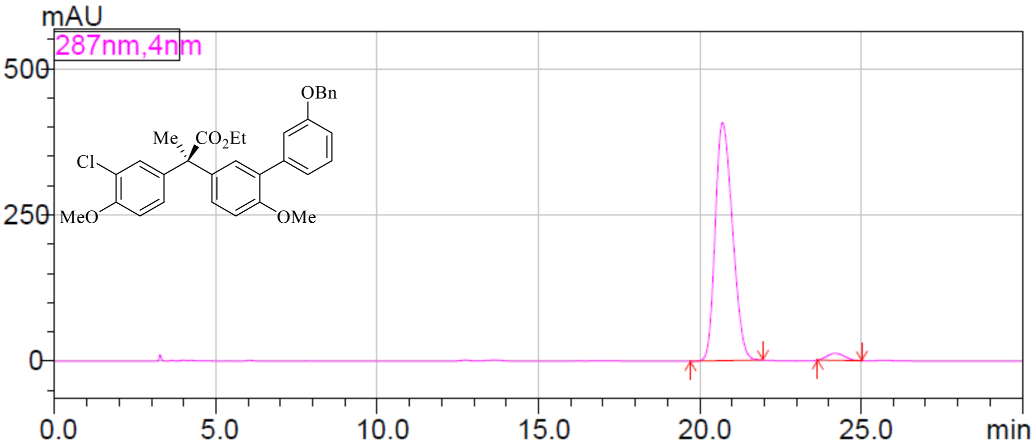

<Peak Table>

PDA Ch1 287nm

Peak#	Ret. Time	Area	Height	Area%	Peak Start	Peak End
1	20.707	15164948	407169	97.131	19.707	21.957
2	24.201	447937	11922	2.869	23.659	25.024
Total		15612885	419091	100.000		

12

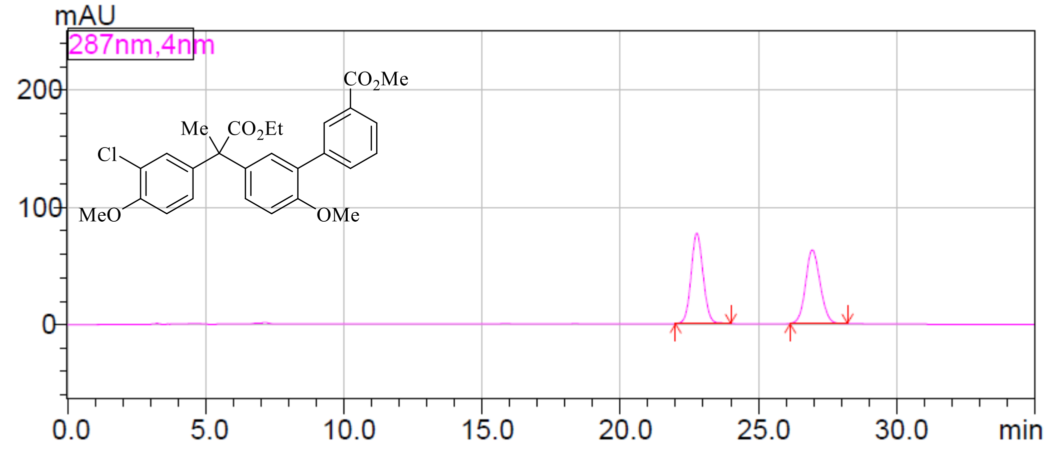

<Peak Table>

PDA Ch1 287nm

Peak#	Ret. Time	Area	Height	Area%	Peak Start	Peak End
1	22.770	2345038	77111	50.547	22.005	24.000
2	26.951	2294279	62779	49.453	26.149	28.224
Total		4639316	139889	100.000		

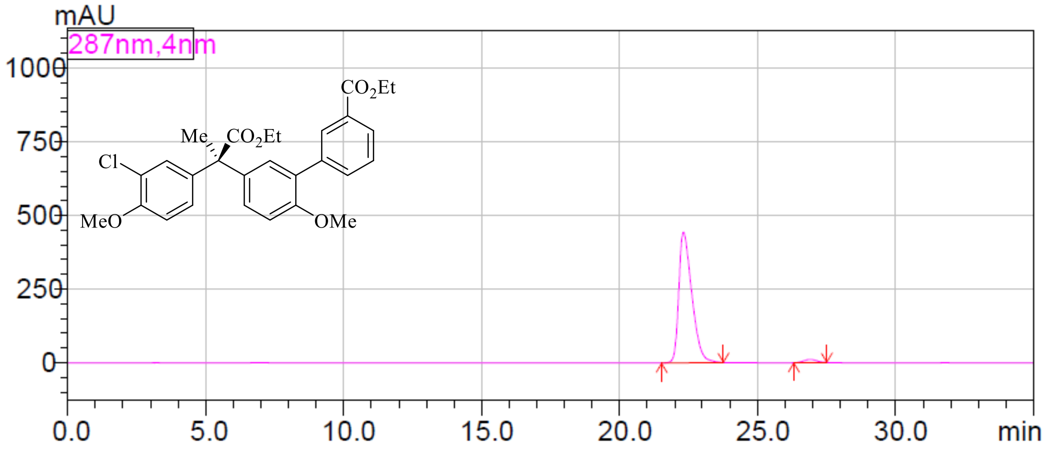

<Peak Table>

PDA Ch1 287nm

Peak#	Ret. Time	Area	Height	Area%	Peak Start	Peak End
1	22.316	14538369	442768	97.654	21.525	23.760
2	26.909	349256	10516	2.346	26.309	27.504
Total		14887625	453284	100.000		

13

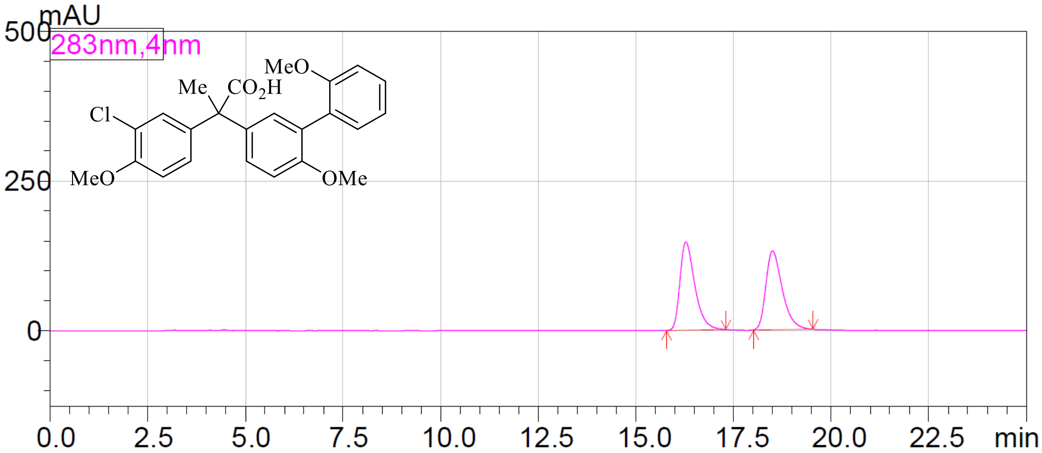

<Peak Table>

PDA Ch1 283nm

Peak#	Ret. Time	Area	Height	Area%	Peak Start	Peak End
1	16.280	5228585	197469	50.385	15.787	17.307
2	18.498	5148665	176332	49.615	18.016	19.536
Total		10377250	373801	100.000		

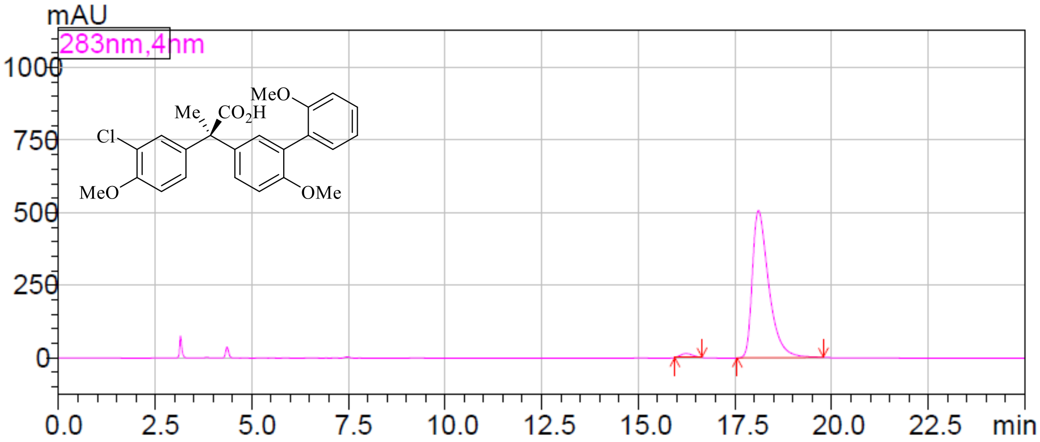

<Peak Table>

PDA Ch1 283nm

Peak#	Ret. Time	Area	Height	Area%	Peak Start	Peak End
1	16.237	291186	13506	1.909	15.941	16.651
2	18.111	14959859	507251	98.091	17.563	19.787
Total		15251045	520757	100.000		

14

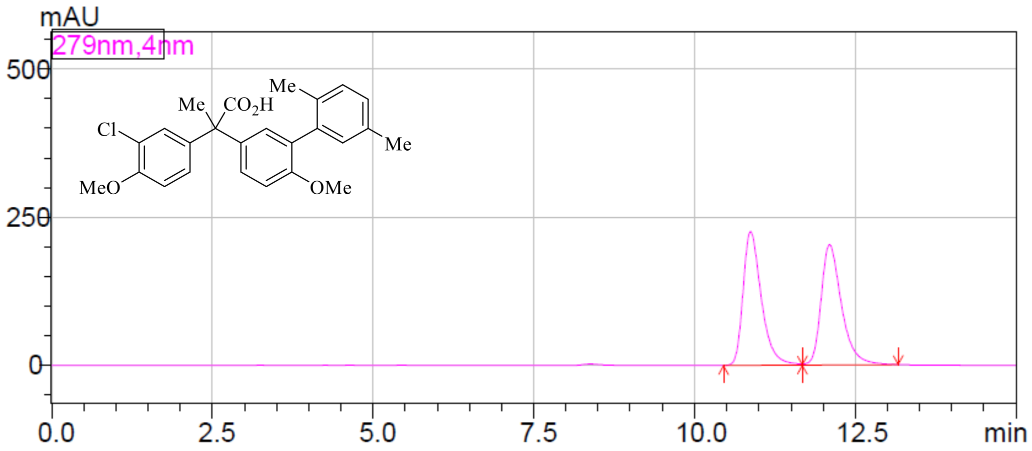

<Peak Table>

PDA Ch1 279nm

Peak#	Ret. Time	Area	Height	Area%	Peak Start	Peak End
1	10.872	4473553	225843	49.969	10.448	11.675
2	12.100	4479051	203077	50.031	11.675	13.168
Total		8952604	428920	100.000		

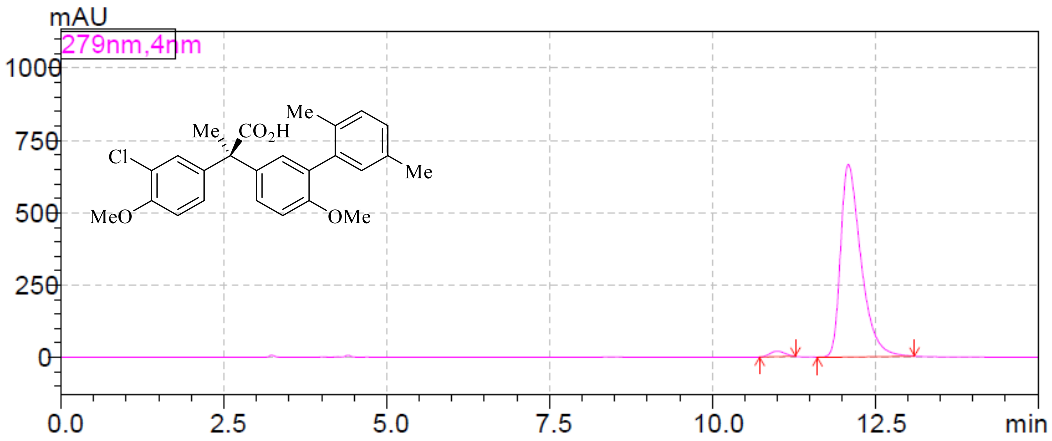

<Peak Table>

PDA Ch1 279nm

Peak#	Ret. Time	Area	Height	Area%	Peak Start	Peak End
1	10.996	309414	19248	2.089	10.725	11.275
2	12.088	14501255	664720	97.911	11.611	13.104
Total		14810669	683968	100.000		

S-15

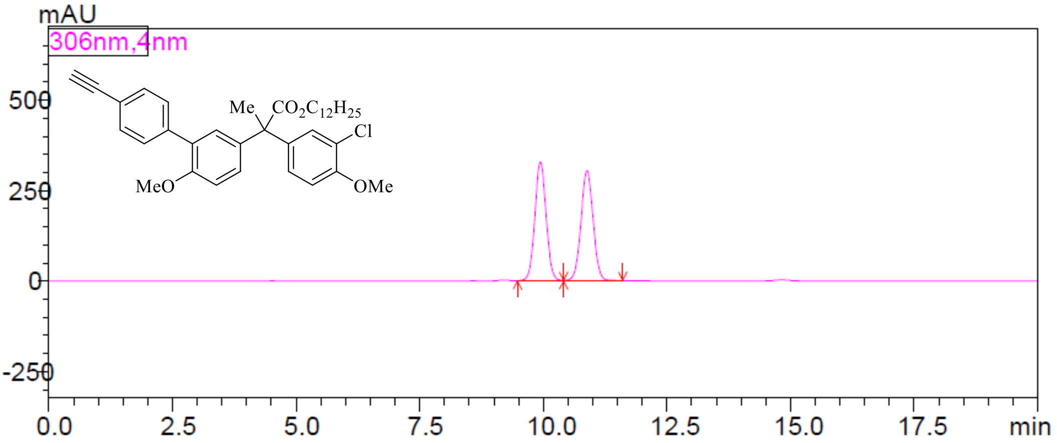

<Peak Table>

PDA Ch1 306nm

Peak#	Ret. Time	Area	Height	Area%	Peak Start	Peak End
1	9.939	5192577	327625	49.886	9.477	10.411
2	10.882	5216254	304031	50.114	10.411	11.605
Total		10408831	631657	100.000		

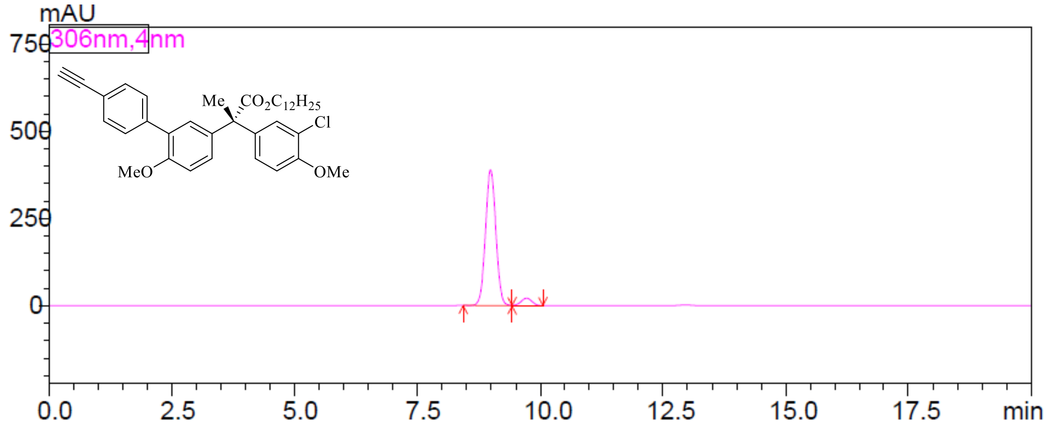

<Peak Table>

PDA Ch1 306nm

Peak#	Ret. Time	Area	Height	Area%	Peak Start	Peak End
1	8.987	5713231	388844	94.546	8.432	9.424
2	9.721	329564	21420	5.454	9.424	10.053
Total		6042796	410264	100.000		

16

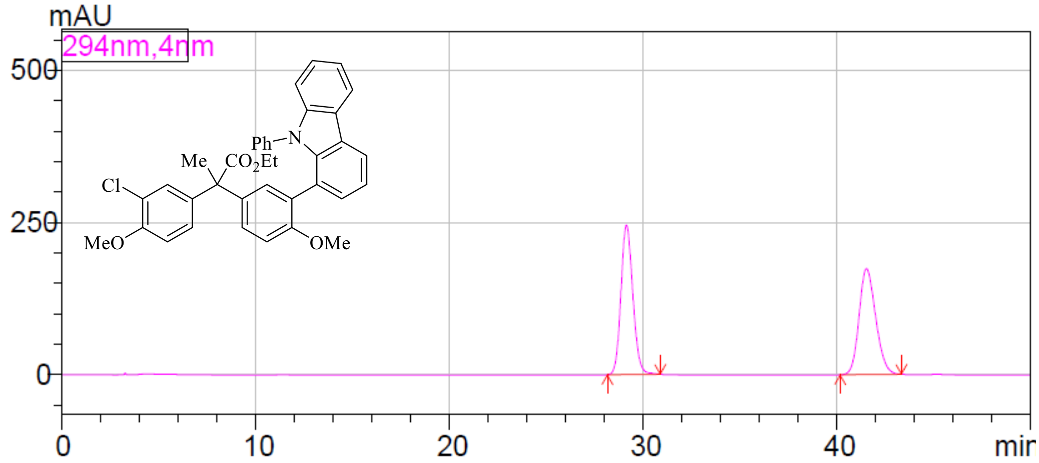

<Peak Table>

PDA Ch1 294nm

Peak#	Ret. Time	Area	Height	Area%	Peak Start	Peak End
1	29.149	10524759	245782	50.245	28.155	30.891
2	41.537	10422101	174127	49.755	40.181	43.328
Total		20946860	419909	100.000		

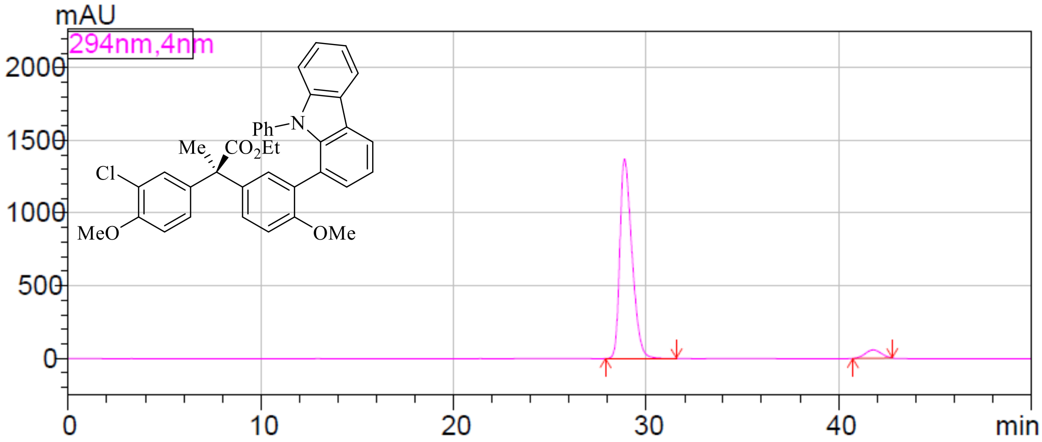

<Peak Table>

PDA Ch1 294nm

Peak#	Ret. Time	Area	Height	Area%	Peak Start	Peak End
1	28.882	60150957	1373169	94.924	27.883	31.573
2	41.792	3216207	56565	5.076	40.731	42.779
Total		63367164	1429733	100.000		

17

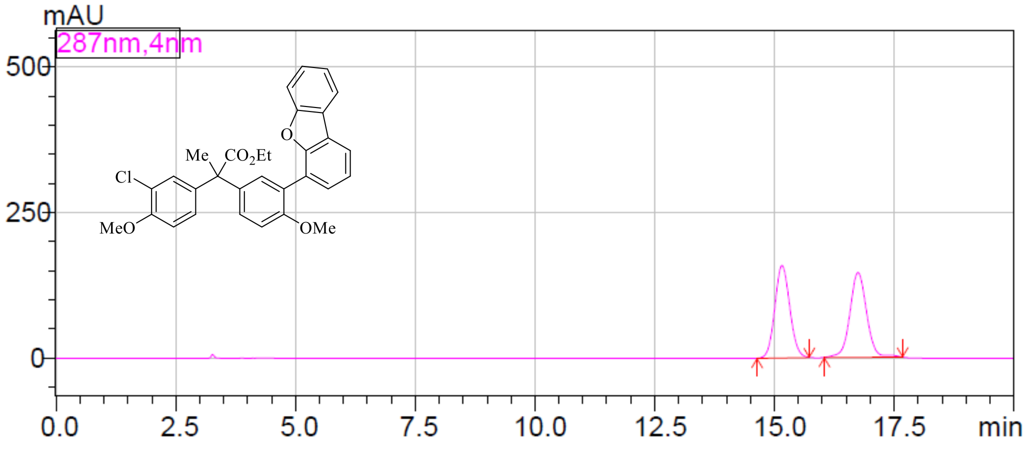

<Peak Table>

PDA Ch1 287nm

Peak#	Ret. Time	Area	Height	Area%	Peak Start	Peak End
1	15.160	3438612	158265	49.331	14.635	15.728
2	16.747	3531885	145421	50.669	16.043	17.680
Total		6970497	303687	100.000		

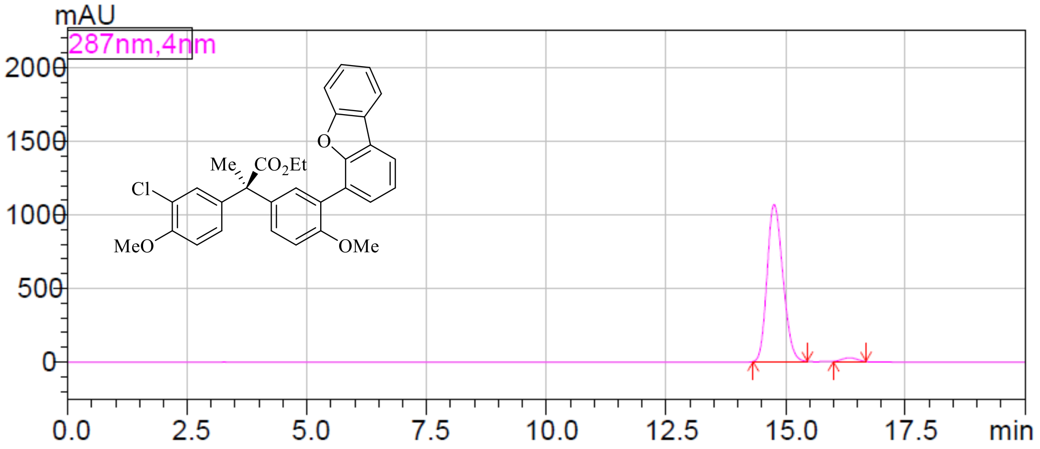

<Peak Table>

PDA Ch1 287nm

Peak#	Ret. Time	Area	Height	Area%	Peak Start	Peak End
1	14.762	24475957	1065229	97.911	14.320	15.456
2	16.336	522155	24706	2.089	16.000	16.683
Total		24998112	1089935	100.000		

18

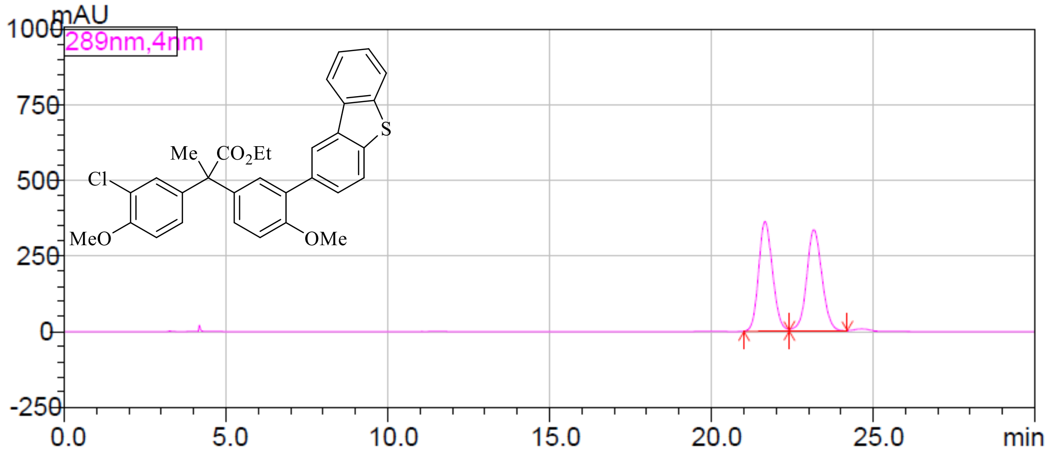

<Peak Table>

PDA Ch1 289nm

Peak#	Ret. Time	Area	Height	Area%	Peak Start	Peak End
1	21.659	10823553	362410	49.514	21.003	22.405
2	23.168	11035900	334685	50.486	22.405	24.203
Total		21859452	697094	100.000		

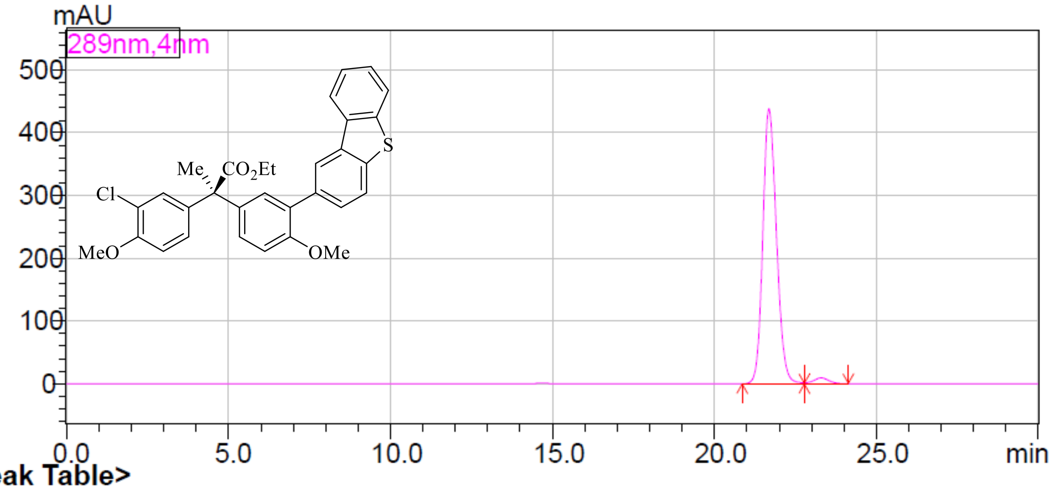

<Peak Table>

PDA Ch1 289nm

Peak#	Ret. Time	Area	Height	Area%	Peak Start	Peak End
1	21.688	12793297	438207	97.672	20.864	22.773
2	23.301	304904	9307	2.328	22.773	24.139
Total		13098201	447514	100.000		

19

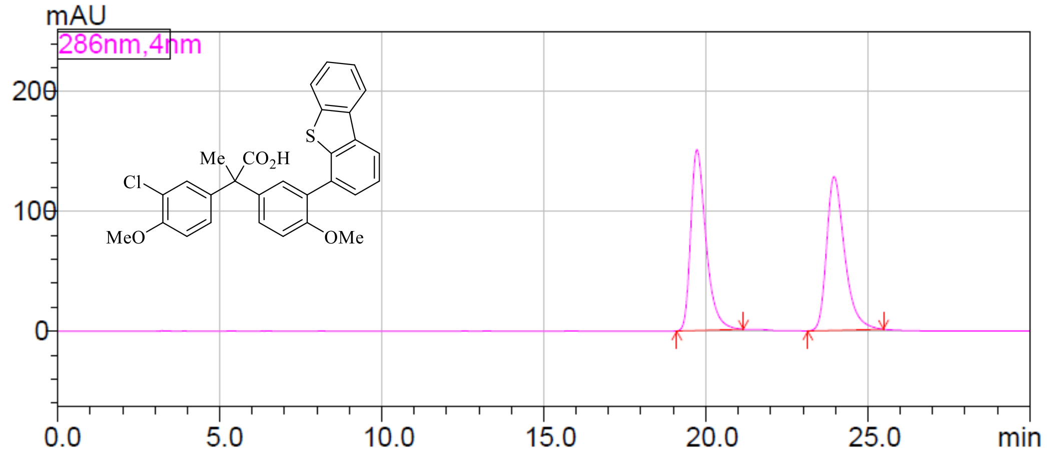

<Peak Table>

PDA Ch1 286nm

Peak#	Ret. Time	Area	Height	Area%	Peak Start	Peak End
1	19.729	4943051	150812	49.821	19.099	21.157
2	23.961	4978579	128226	50.179	23.147	25.488
Total		9921630	279038	100.000		

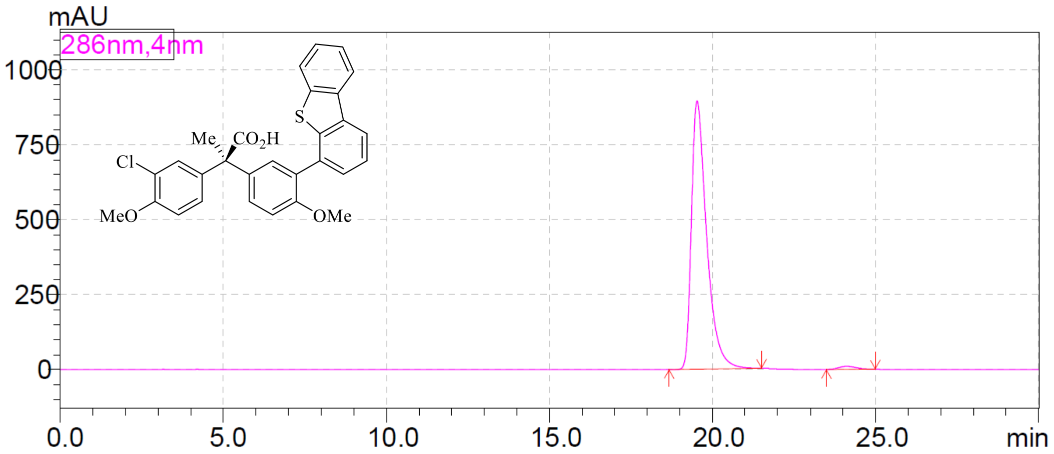

<Peak Table>

PDA Ch1 286nm

Peak#	Ret. Time	Area	Height	Area%	Peak Start	Peak End
1	19.530	29060066	895145	98.706	18.672	21.509
2	24.120	381062	10341	1.294	23.499	24.992
Total		29441128	905486	100.000		

20

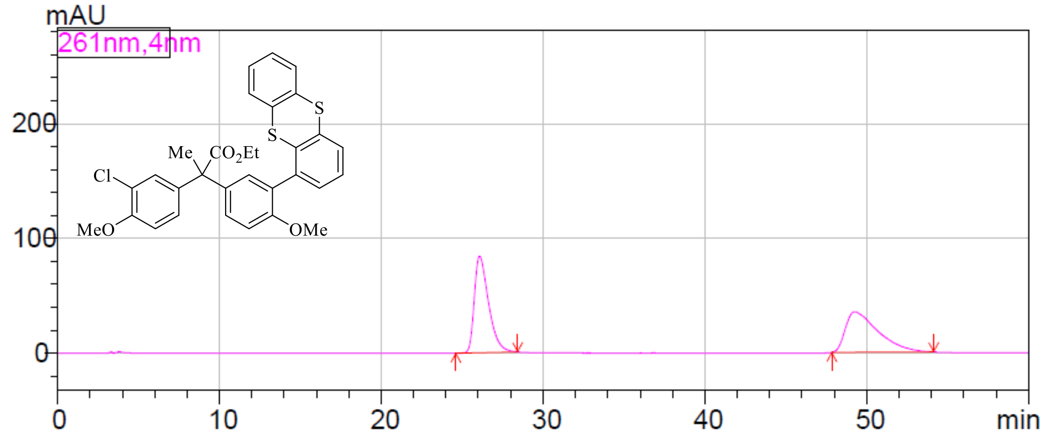

<Peak Table>

PDA Ch1 261nm

Peak#	Ret. Time	Area	Height	Area%	Peak Start	Peak End
1	26.077	5156676	84238	50.950	24.603	28.427
2	49.259	4964390	35441	49.050	47.835	54.123
Total		10121067	119679	100.000		

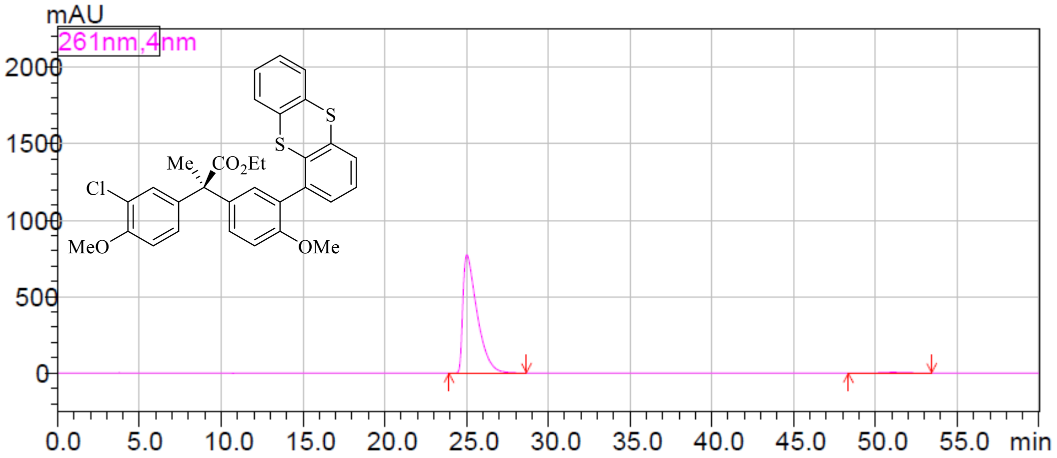

<Peak Table>

PDA Ch1 261nm

Peak#	Ret. Time	Area	Height	Area%	Peak Start	Peak End
1	25.024	47158016	772854	98.782	23.925	28.661
2	51.094	581408	5090	1.218	48.341	53.440
Total		47739424	777944	100.000		

21

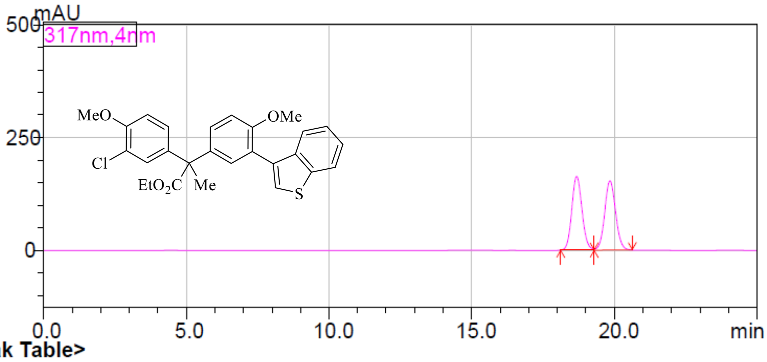

<Peak Table>

PDA Ch1 317nm

Peak#	Ret. Time	Area	Height	Area%	Peak Start	Peak End
1	18.679	4065281	163493	49.918	18.123	19.291
2	19.847	4078587	153441	50.082	19.291	20.624
Total		8143868	316934	100.000		

1.2 eq. boronic acid

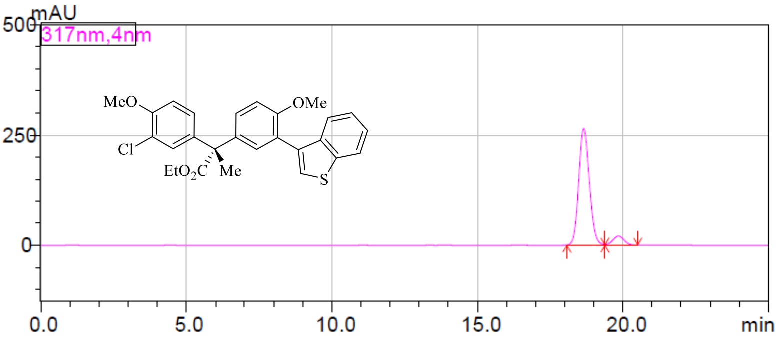

<Peak Table>

PDA Ch1 317nm

Peak#	Ret. Time	Area	Height	Area%	Peak Start	Peak End
1	18.654	6730558	264538	92.322	18.069	19.381
2	19.847	559756	20617	7.678	19.381	20.512
Total		7290313	285155	100.000		

3.6 eq. boronic acid

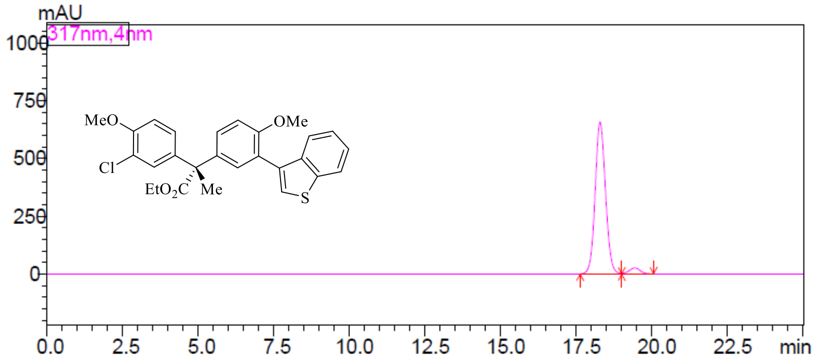

<Peak Table>

PDA Ch1 317nm

Peak#	Ret. Time	Area	Height	Area%	Peak Start	Peak End
1	18.290	15636672	657540	95.918	17.632	19.008
2	19.437	665428	26065	4.082	19.008	20.059
Total		16302100	683605	100.000		

22

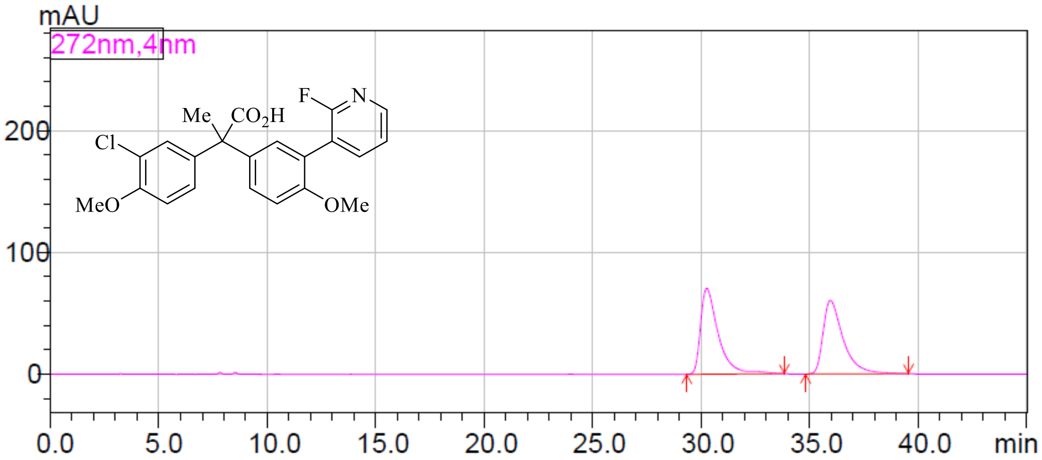

<Peak Table>

PDA Ch1 272nm

Peak#	Ret. Time	Area	Height	Area%	Peak Start	Peak End
1	30.259	4010460	70457	50.842	29.333	33.835
2	35.957	3877552	60375	49.158	34.805	39.552
Total		7888011	130832	100.000		

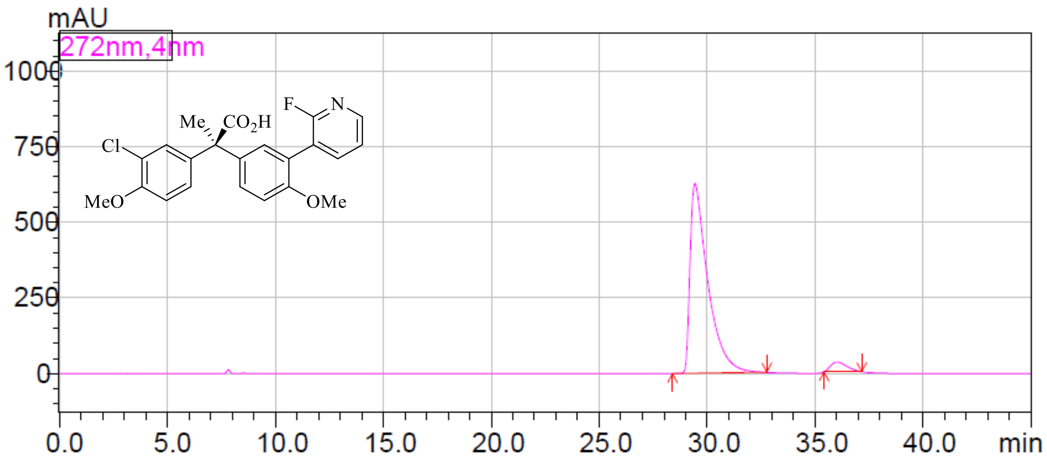

<Peak Table>

PDA Ch1 272nm

Peak#	Ret. Time	Area	Height	Area%	Peak Start	Peak End
1	29.438	35976229	627155	95.544	28.389	32.768
2	36.021	1677741	32179	4.456	35.419	37.152
Total		37653970	659334	100.000		

23

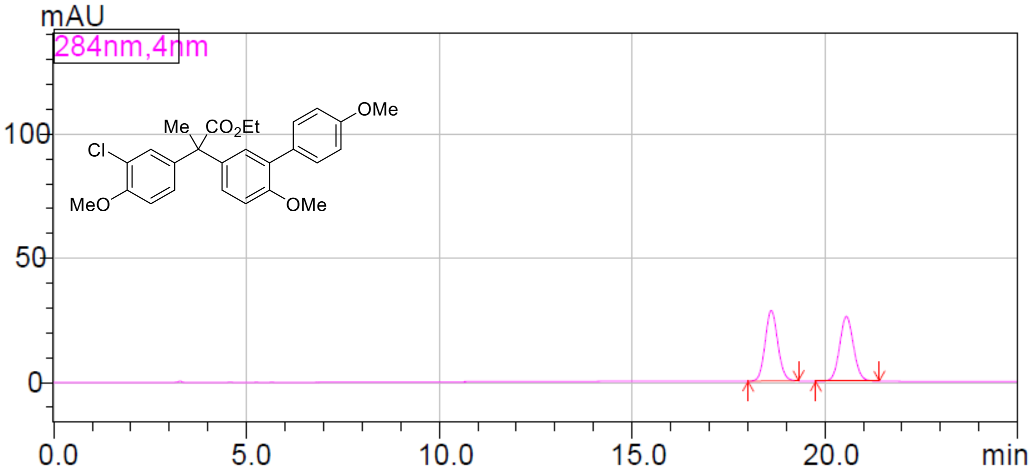

<Peak Table>

PDA Ch1 284nm

Peak#	Ret. Time	Area	Height	Mark	Area%	Peak Start	Peak End
1	18.613	650742	28409	M	49.885	18.011	19.317
2	20.562	653742	26093	M	50.115	19.771	21.419
Total		1304484	54502		100.000		

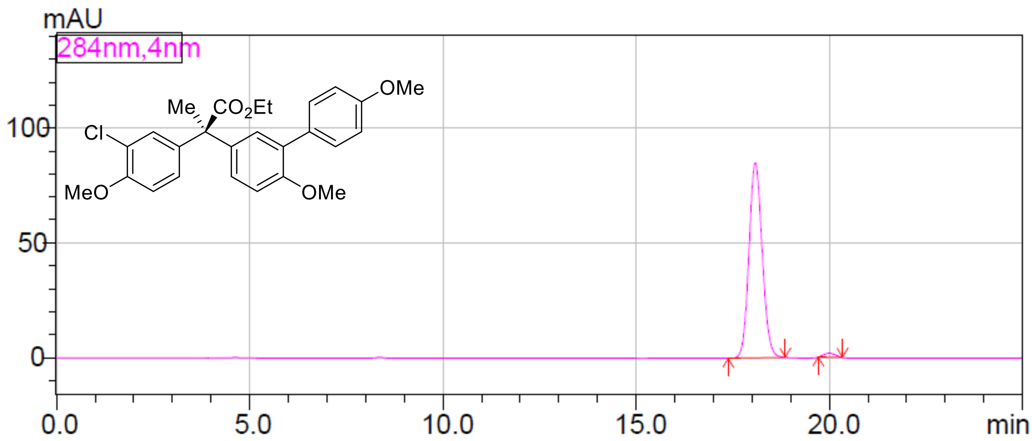

<Peak Table>

PDA Ch1 284nm

Peak#	Ret. Time	Area	Height	Mark	Area%	Peak End	Peak Start
1	18.085	2017482	85068	M	98.269	18.864	17.387
2	20.003	35539	1730	M	1.731	20.341	19.717
Total		2053021	86797		100.000		

24

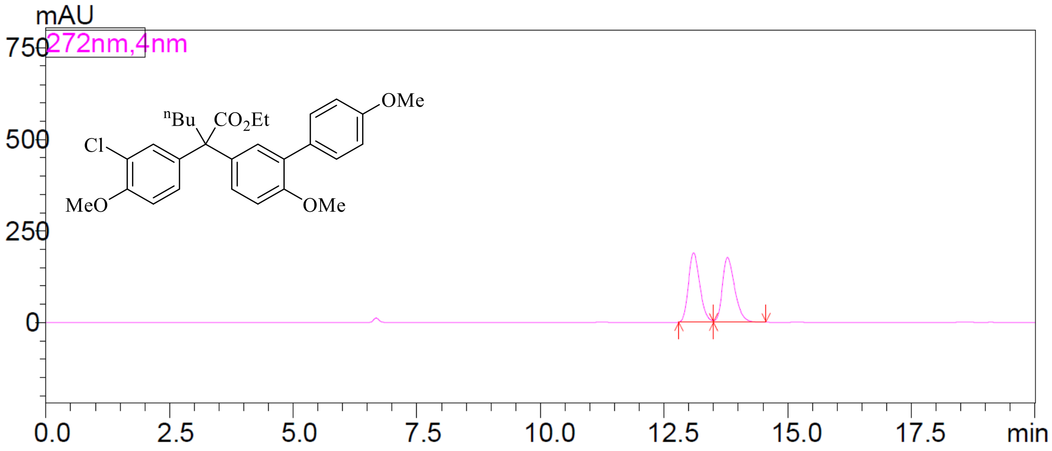

<Peak Table>

PDA Ch1 272nm

Peak#	Ret. Time	Area	Height	Area%	Peak Start	Peak End
1	13.098	3027563	189122	49.653	12.800	13.499
2	13.783	3069826	177020	50.347	13.499	14.560
Total		6097389	366142	100.000		

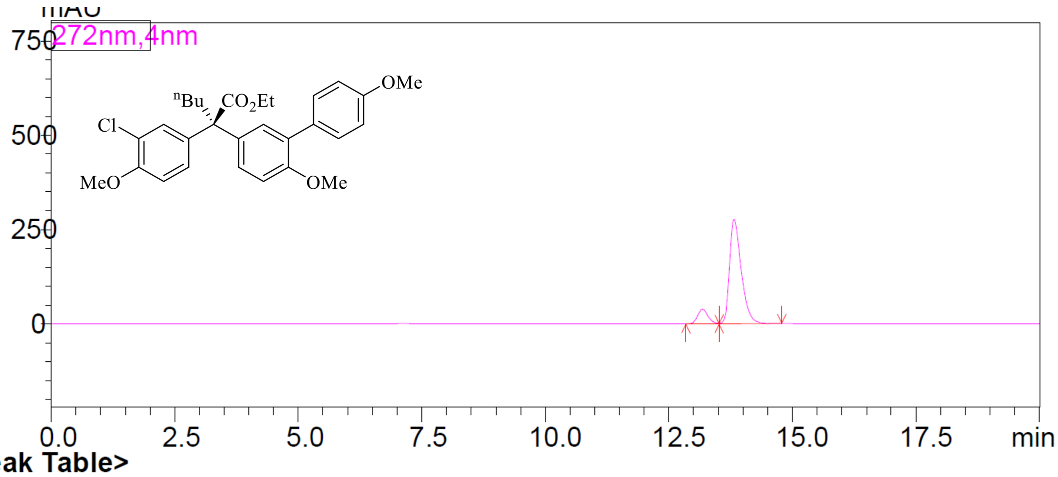

<Peak Table>

PDA Ch1 272nm

Peak#	Ret. Time	Area	Height	Area%	Peak Start	Peak End
1	13.180	575020	38807	11.156	12.843	13.515
2	13.819	4579442	276852	88.844	13.515	14.784
Total		5154461	315659	100.000		

25

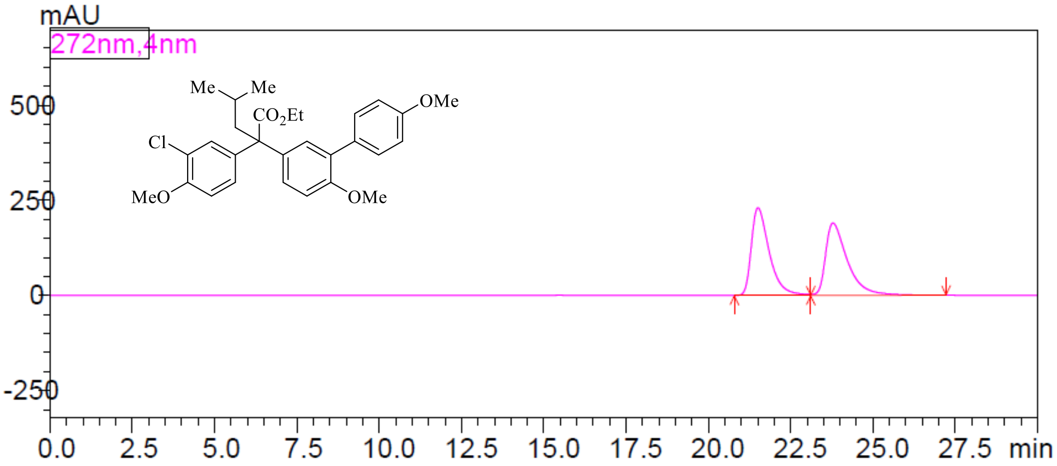

<Peak Table>

PDA Ch1 272nm

Peak#	Ret. Time	Area	Height	Area%	Peak Start	Peak End
1	21.509	8770265	230518	49.824	20.789	23.109
2	23.787	8832127	190286	50.176	23.109	27.221
Total		17602392	420805	100.000		

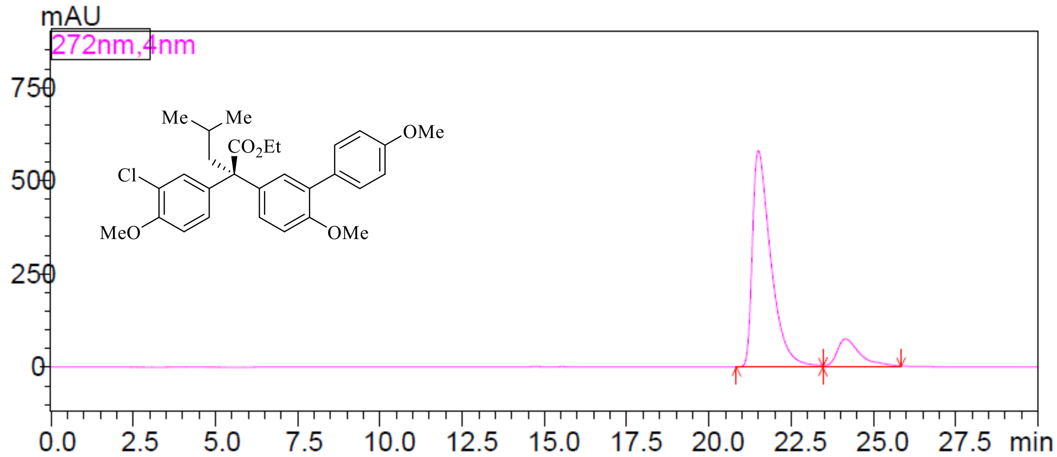

<Peak Table>

PDA Ch1 272nm

Peak#	Ret. Time	Area	Height	Area%	Peak Start	Peak End
1	21.500	22694431	580277	85.685	20.832	23.461
2	24.148	3791320	75146	14.315	23.461	25.835
Total		26485750	655423	100.000		

26

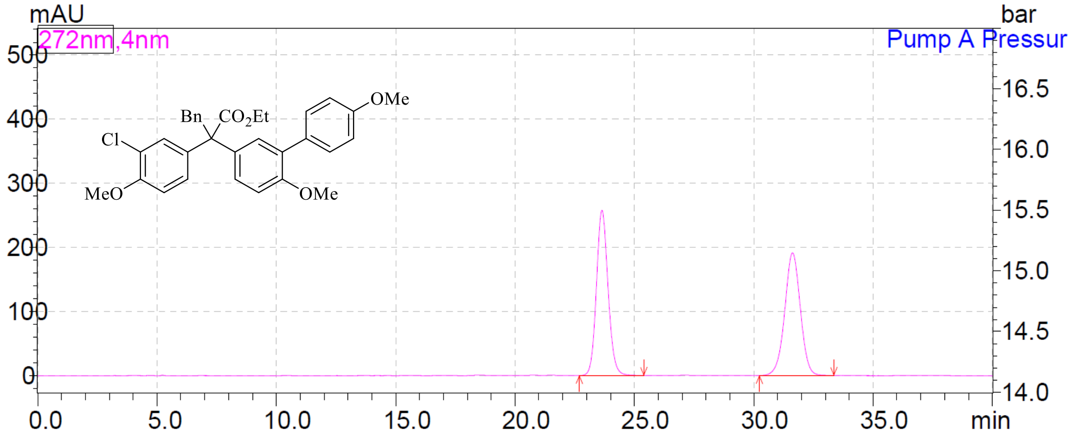

<Peak Table>

PDA Ch1 272nm

Peak#	Ret. Time	Area	Height	Area%	Peak Start	Peak End
1	23.638	8733153	257776	50.052	22.688	25.392
2	31.622	8714890	191258	49.948	30.235	33.355
Total		17448043	449034	100.000		

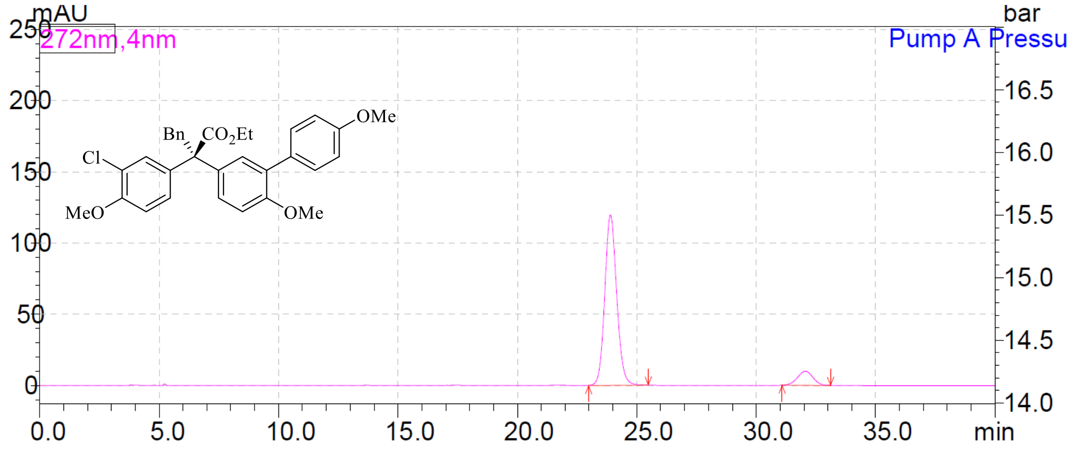

<Peak Table>

PDA Ch1 272nm

Peak#	Ret. Time	Area	Height	Area%	Peak Start	Peak End
1	23.894	4154761	119645	90.319	22.981	25.483
2	32.051	445325	9928	9.681	31.088	33.109
Total		4600085	129574	100.000		

27

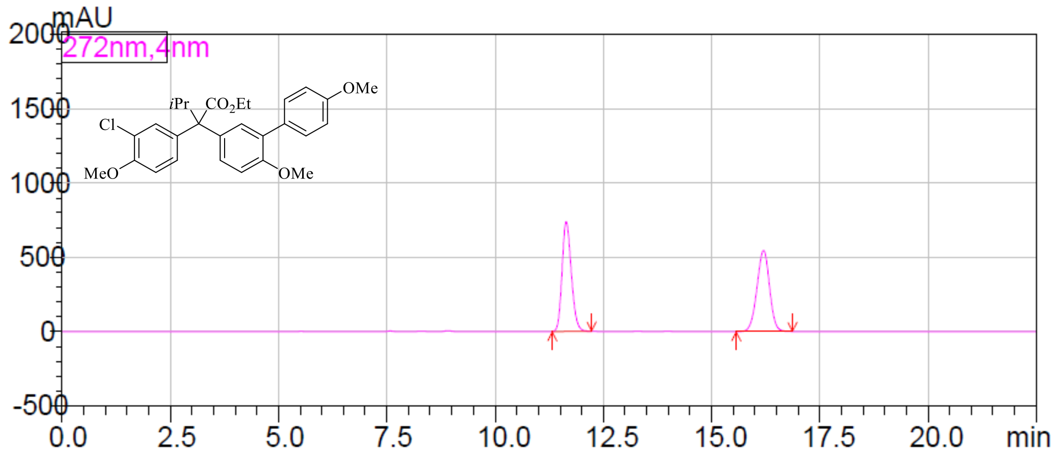

<Peak Table>

PDA Ch1 272nm

Peak#	Ret. Time	Area	Height	Area%	Peak Start	Peak End
1	11.643	11001078	739355	49.891	11.323	12.229
2	16.201	11049140	546352	50.109	15.568	16.869
Total		22050218	1285707	100.000		

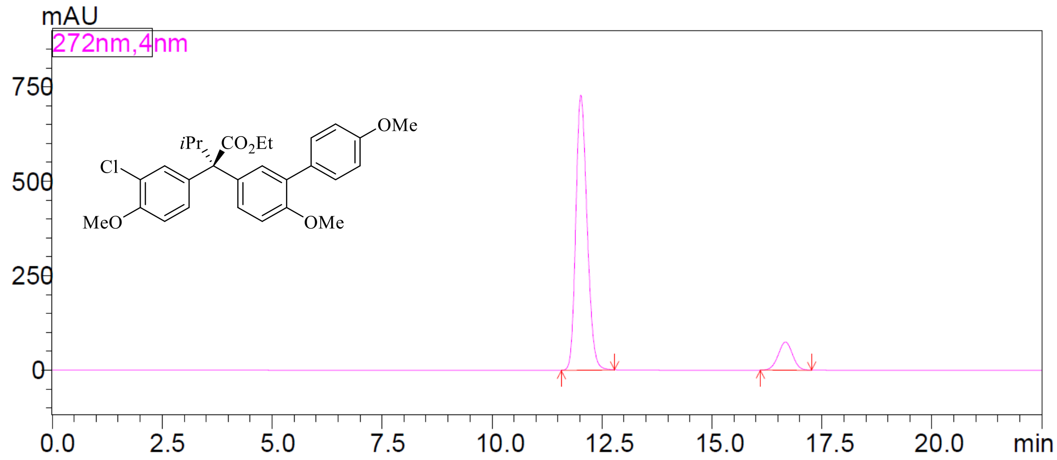

<Peak Table>

PDA Ch1 272nm

Peak#	Ret. Time	Area	Height	Area%	Peak Start	Peak End
1	12.020	12978908	728767	88.391	11.573	12.784
2	16.673	1704649	74543	11.609	16.101	17.269
Total		14683557	803310	100.000		

28

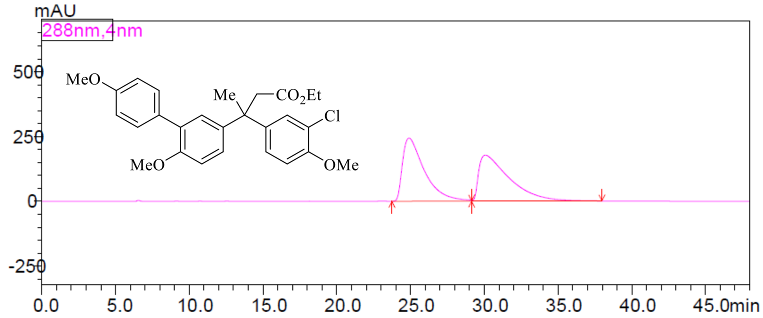

<Peak Table>

PDA Ch1 288nm

Peak#	Ret. Time	Area	Height	Area%	Peak Start	Peak End
1	24.896	25212477	243634	49.534	23.744	29.147
2	30.072	25686771	177254	50.466	29.147	37.973
Total		50899248	420888	100.000		

Reaction in THF:

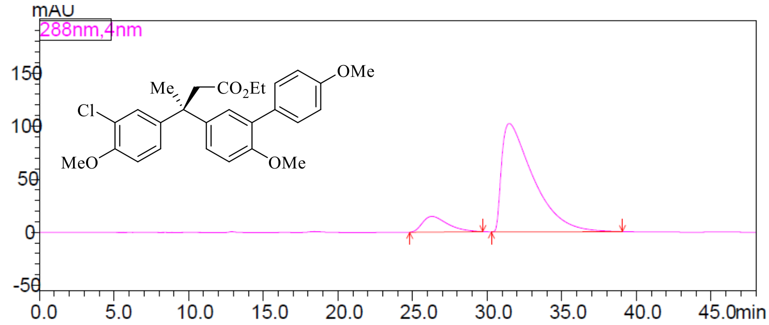

<Peak Table>

PDA Ch1 288nm

Peak#	Ret. Time	Area	Height	Area%	Peak Start	Peak End
1	26.277	1720381	14724	10.248	24.811	29.707
2	31.474	15066695	102149	89.752	30.283	39.056
Total		16787076	116873	100.000		

Reaction in 2-MeTHF:

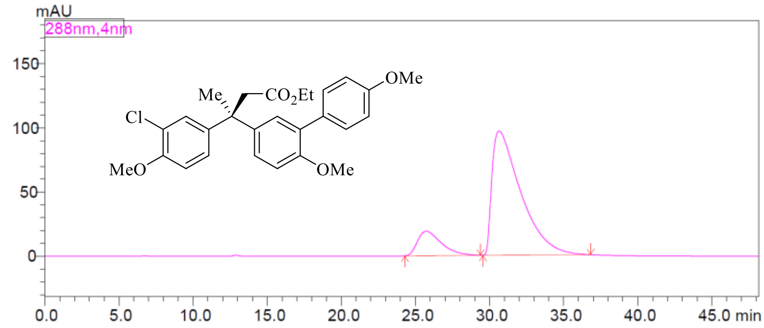

<Peak Table>

PDA Ch1 288nm

Peak#	Ret. Time	Area	Height	Area%	Peak Start	Peak End
1	25.730	2137372	19044	13.983	24.293	29.387
2	30.644	13148137	96869	86.017	29.547	36.821
Total		15285509	115912	100.000		

29

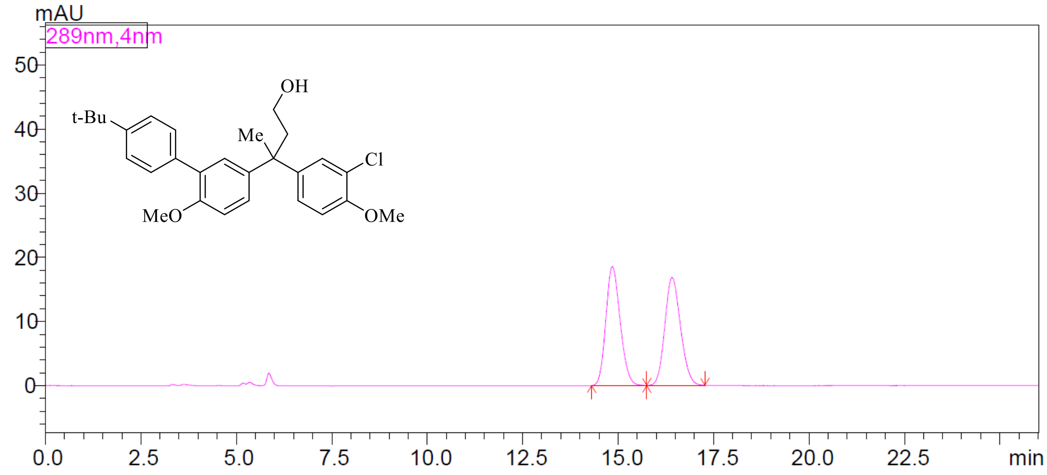

<Peak Table>

PDA Ch1 289nm

Peak#	Ret. Time	Area	Height	Area%	Peak Start	Peak End
1	14.850	484493	18566	50.075	14.304	15.749
2	16.412	483039	16836	49.925	15.749	17.275
Total		967532	35402	100.000		

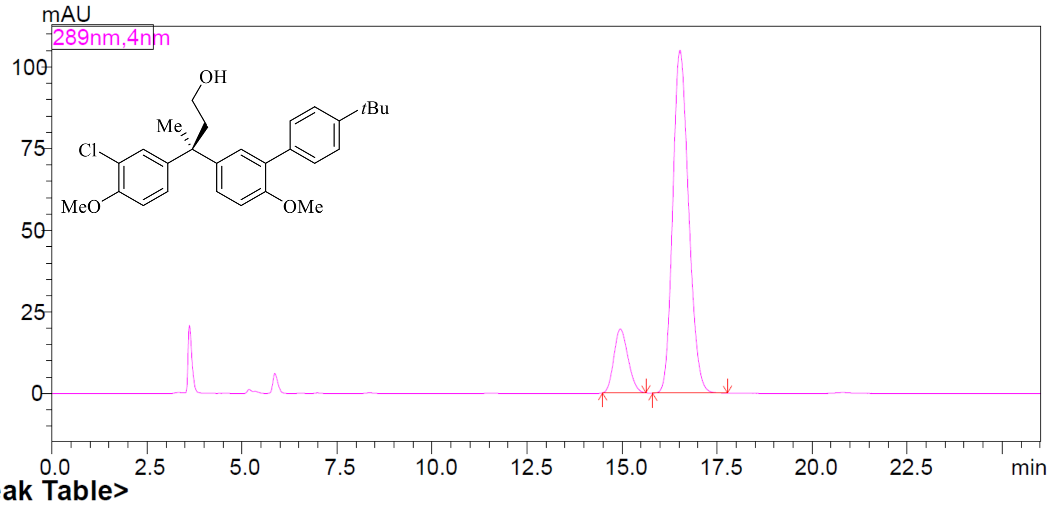

<Peak Table>

PDA Ch1 289nm

Peak#	Ret. Time	Area	Height	Area%	Peak Start	Peak End
1	14.956	508631	19552	14.238	14.485	15.627
2	16.524	3063709	104952	85.762	15.808	17.776
Total		3572340	124504	100.000		

3

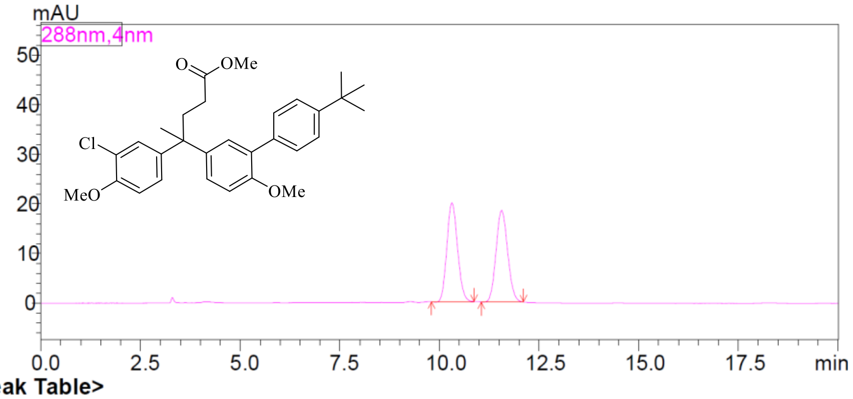

<Peak Table>

PDA Ch1 288nm

Peak#	Ret. Time	Area	Height	Area%	Peak Start	Peak End
1	10.314	370541	19946	49.696	9.803	10.869
2	11.561	375069	18483	50.304	11.051	12.112
Total		745610	38430	100.000		

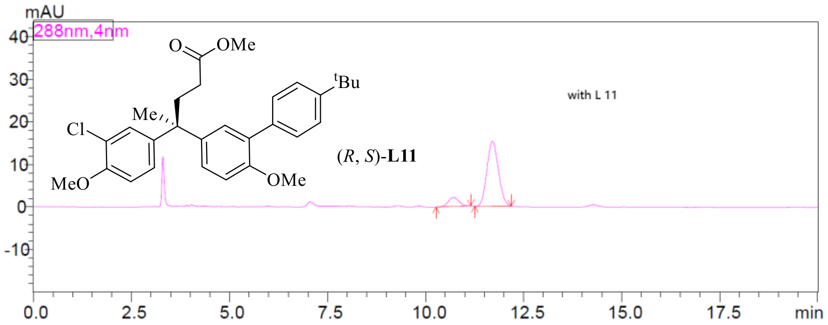

<Peak Table>

PDA Ch1 288nm

Peak#	Ret. Time	Area	Height	Area%	Peak Start	Peak End
1	10.717	39900	2155	11.344	10.267	11.157
2	11.705	311819	15248	88.656	11.259	12.187
Total		351718	17403	100.000		

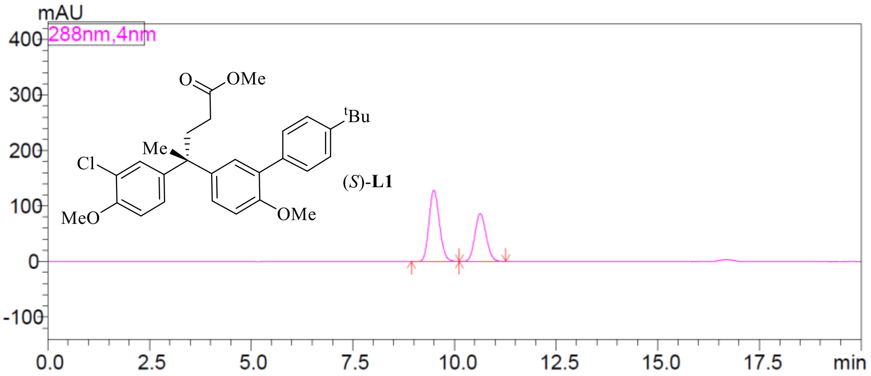

<Peak Table>

PDA Ch1 288nm

Peak#	Ret. Time	Area	Height	Area%	Peak Start	Peak End
1	9.493	2302225	128514	58.022	8.933	10.123
2	10.633	1665622	86087	41.978	10.123	11.264
Total		3967846	214601	100.000		

30

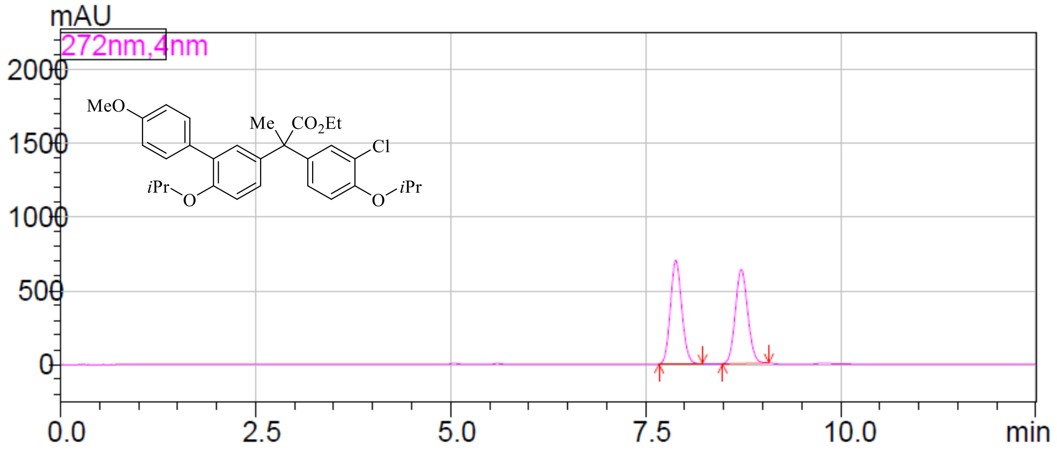

<Peak Table>

PDA Ch1 272nm

Peak#	Ret. Time	Area	Height	Area%	Peak Start	Peak End
1	7.886	7119009	702254	50.286	7.669	8.229
2	8.725	7037920	636038	49.714	8.485	9.077
Total		14156929	1338293	100.000		

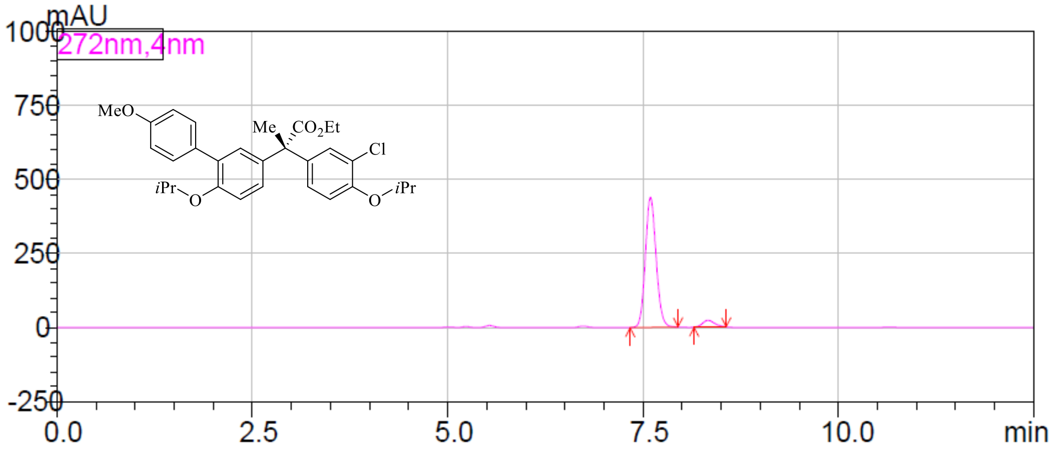

<Peak Table>

PDA Ch1 272nm

Peak#	Ret. Time	Area	Height	Area%	Peak Start	Peak End
1	7.595	4201072	439732	94.468	7.333	7.947
2	8.332	245997	23090	5.532	8.155	8.565
Total		4447068	462822	100.000		

31

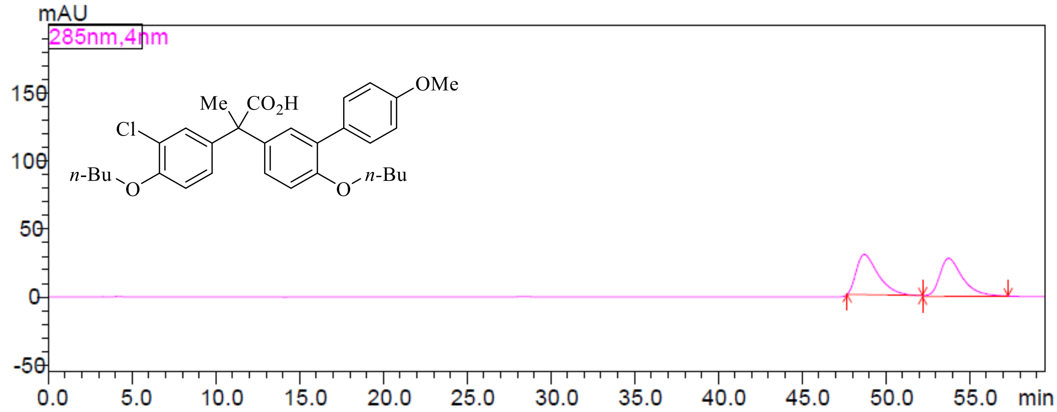

<Peak Table>

PDA Ch1 285nm

Peak#	Ret. Time	Area	Height	Area%	Peak Start	Peak End
1	48.721	2698438	29564	50.372	47.685	52.192
2	53.752	2658630	28021	49.628	52.192	57.307
Total		5357068	57585	100.000		

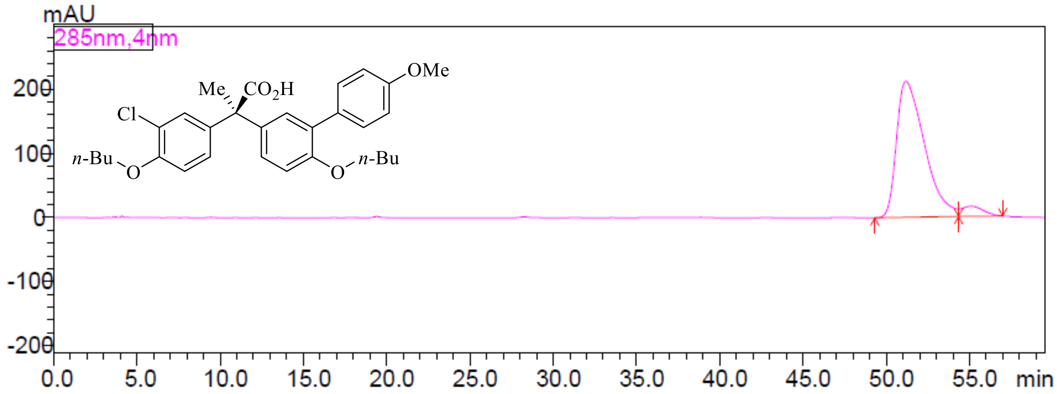

<Peak Table>

PDA Ch1 285nm

Peak#	Ret. Time	Area	Height	Area%	Peak Start	Peak End
1	51.183	24746043	212268	94.414	49.307	54.336
2	55.103	1464237	16080	5.586	54.336	56.981
Total		26210280	228349	100.000		

32

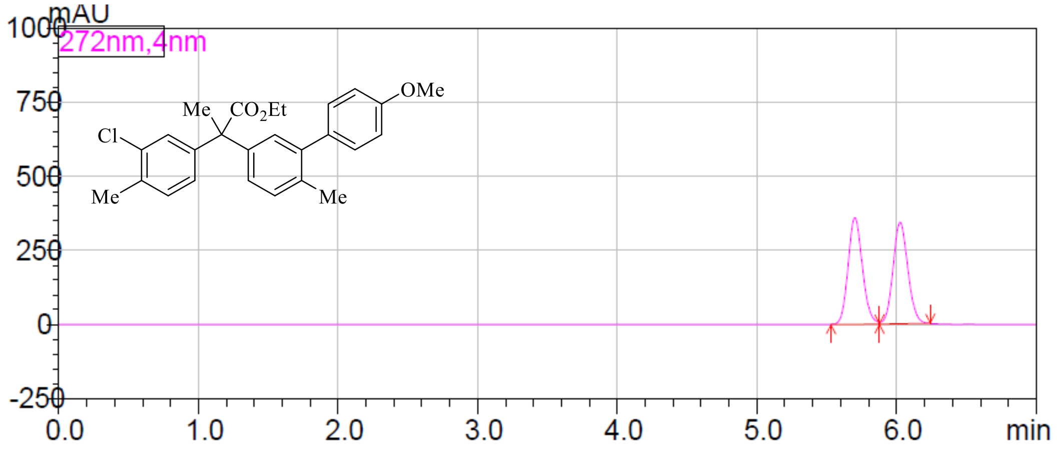

<Peak Table>

PDA Ch1 272nm

Peak#	Ret. Time	Area	Height	Area%	Peak Start	Peak End
1	5.701	2561367	359794	50.314	5.531	5.877
2	6.025	2529439	342694	49.686	5.877	6.240
Total		5090807	702489	100.000		

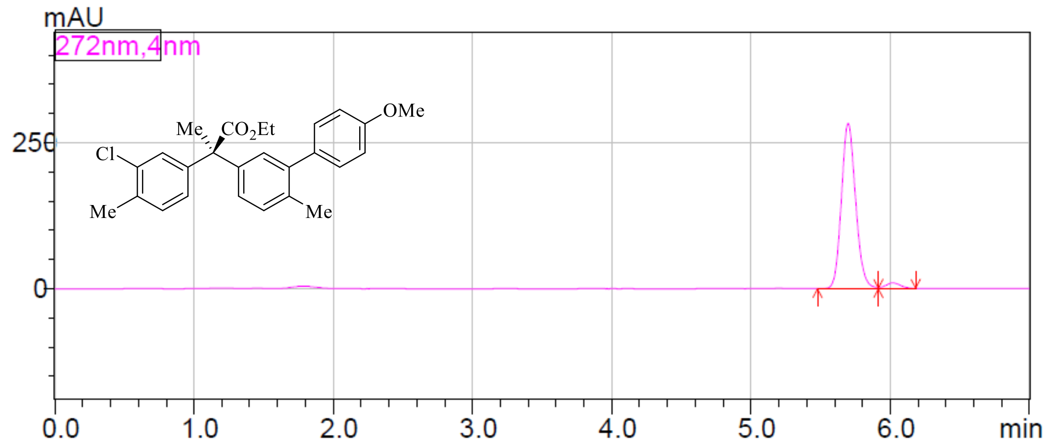

<Peak Table>

PDA Ch1 272nm

Peak#	Ret. Time	Area	Height	Area%	Peak Start	Peak End
1	5.697	2008058	283031	96.612	5.477	5.915
2	6.019	70425	9604	3.388	5.915	6.181
Total		2078483	292635	100.000		

33

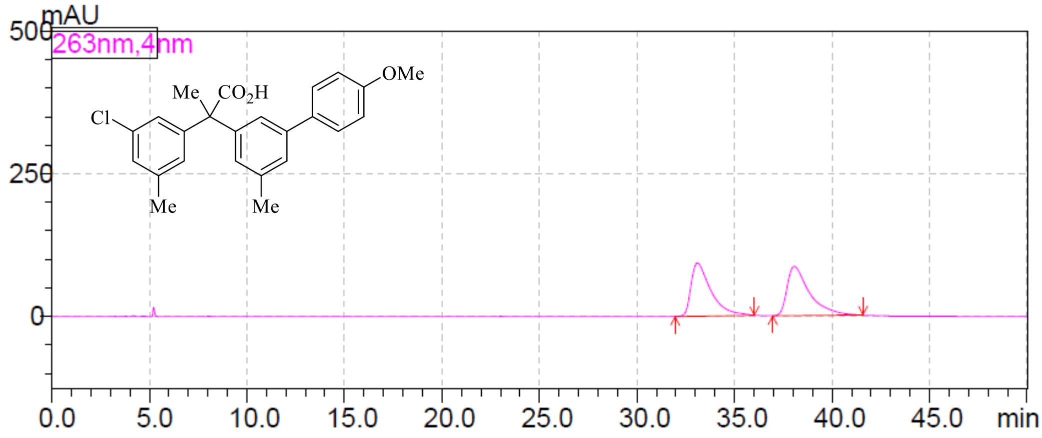

<Peak Table>

PDA Ch1 263nm

Peak#	Ret. Time	Area	Height	Area%	Peak Start	Peak End
1	33.098	6731533	93254	49.255	31.851	36.576
2	38.067	6935242	86301	50.745	36.955	41.589
Total		13666775	179555	100.000		

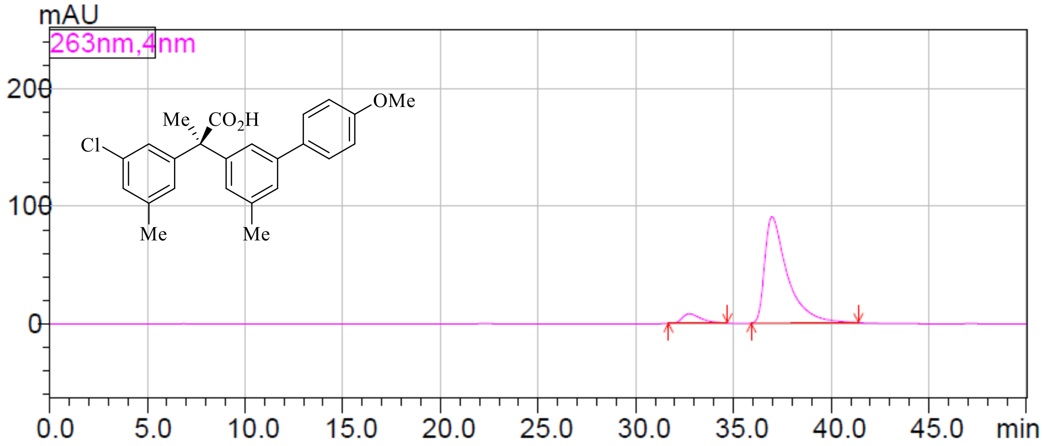

<Peak Table>

PDA Ch1 263nm

Peak#	Ret. Time	Area	Height	Area%	Peak Start	Peak End
1	32.741	524305	7953	6.717	31.664	34.688
2	36.978	7281746	90372	93.283	35.915	41.397
Total		7806050	98325	100.000		

34

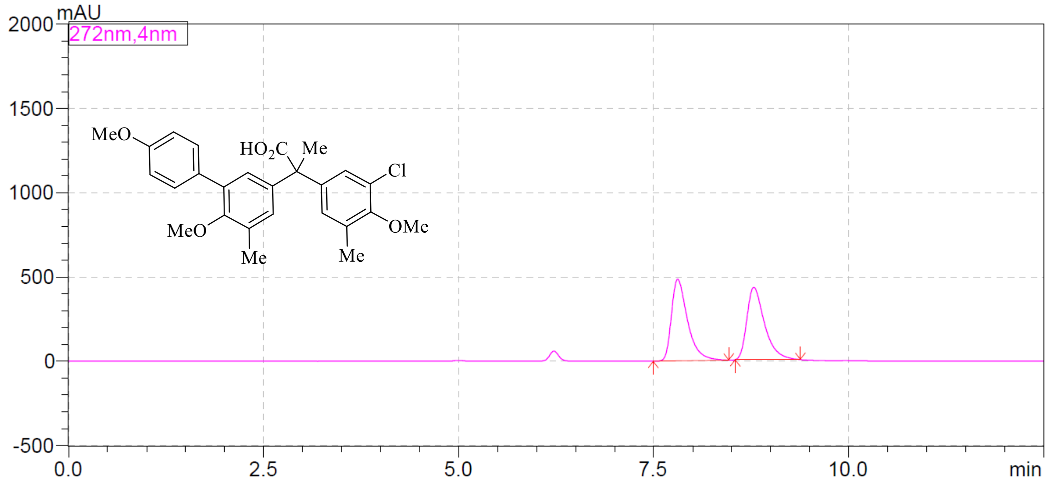

<Peak Table>

PDA Ch1 272nm

Peak#	Ret. Time	Area	Height	Area%	Peak Start	Peak End
1	7.808	6988250	484936	50.768	7.493	8.464
2	8.785	6776746	429830	49.232	8.544	9.381
Total		13764996	914766	100.000		

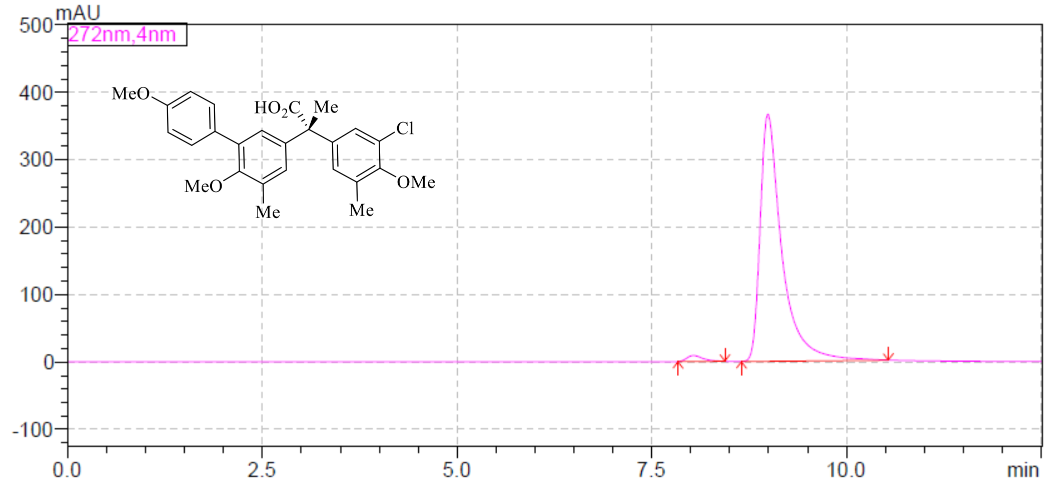

<Peak Table>

PDA Ch1 272nm

Peak#	Ret. Time	Area	Height	Area%	Peak Start	Peak End
1	8.034	127138	8832	1.754	7.835	8.437
2	8.990	7119757	366242	98.246	8.651	10.533
Total		7246896	375074	100.000		

35

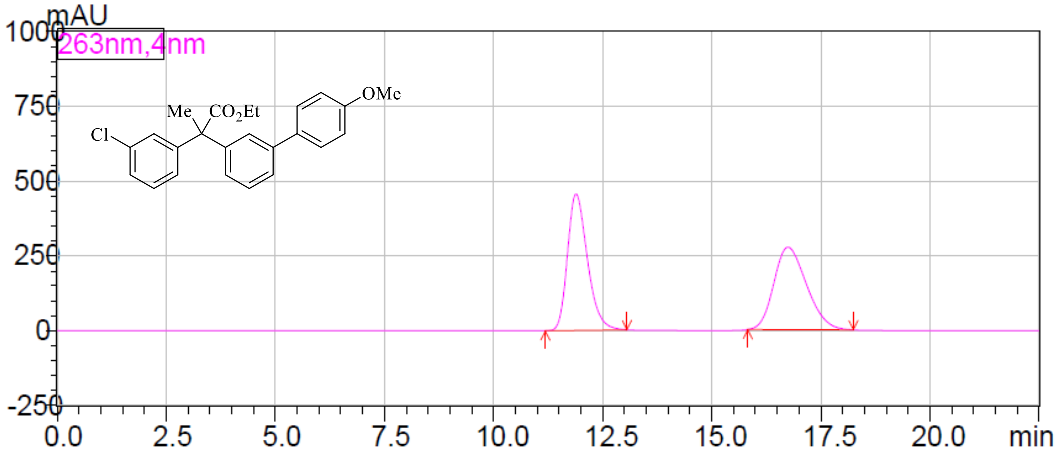

<Peak Table>

PDA Ch1 263nm

Peak#	Ret. Time	Area	Height	Area%	Peak Start	Peak End
1	11.891	14473554	455743	50.170	11.184	13.056
2	16.746	14375205	275516	49.830	15.824	18.245
Total		28848759	731259	100.000		

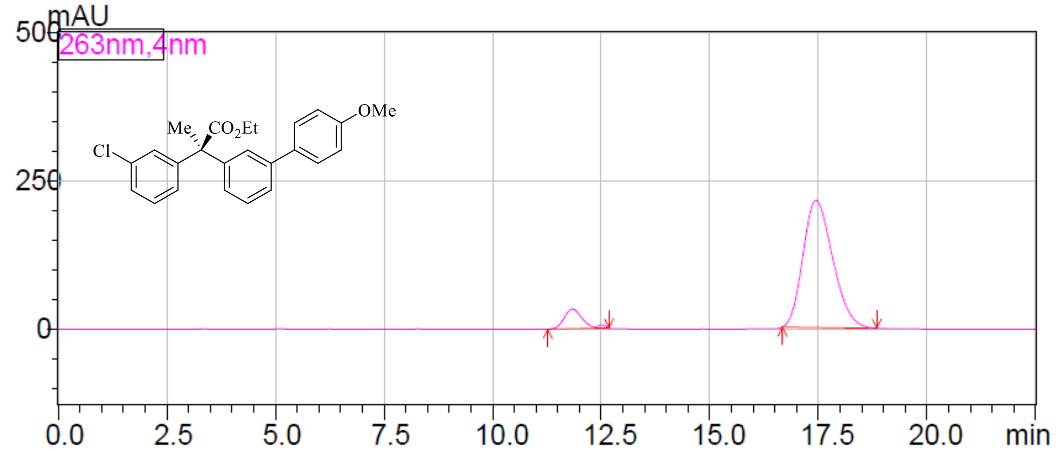

<Peak Table>

PDA Ch1 263nm

Peak#	Ret. Time	Area	Height	Area%	Peak Start	Peak End
1	11.838	948402	33424	8.528	11.312	12.352
2	17.444	10172966	213579	91.472	16.672	18.843
Total		11121368	247003	100.000		

36

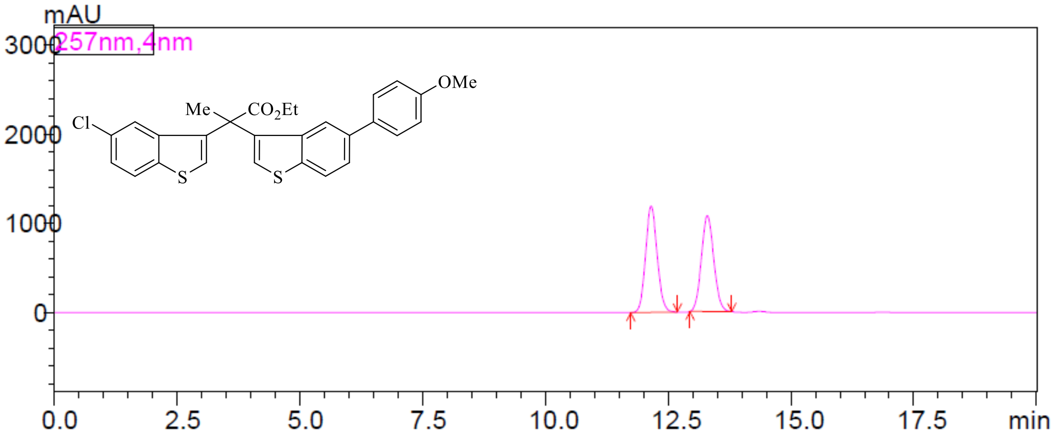

<Peak Table>

PDA Ch1 257nm

Peak#	Ret. Time	Area	Height	Area%	Peak Start	Peak End
1	12.154	19563612	1192110	50.786	11.739	12.688
2	13.299	18958271	1074699	49.214	12.944	13.797
Total		38521883	2266808	100.000		

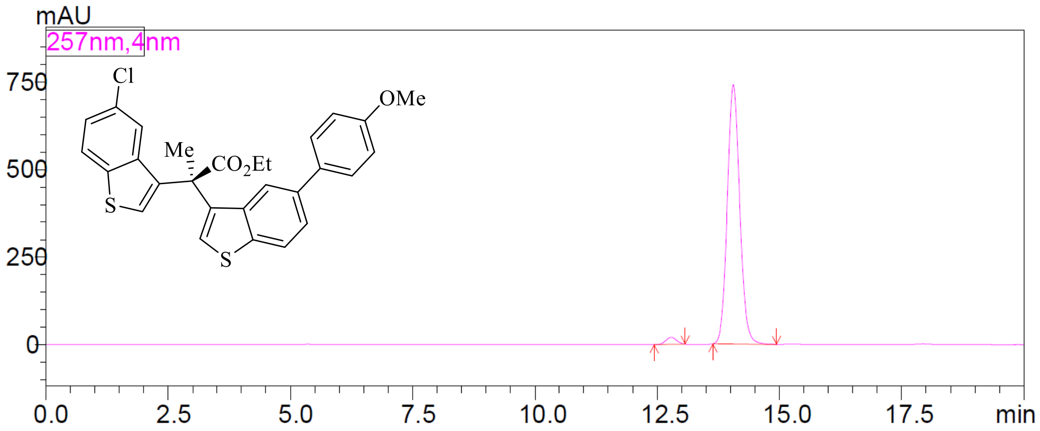

<Peak Table>

PDA Ch1 257nm

Peak#	Ret. Time	Area	Height	Area%	Peak Start	Peak End
1	12.786	314572	20396	2.336	12.437	13.072
2	14.057	13154420	741813	97.664	13.637	14.939
Total		13468992	762209	100.000		

37

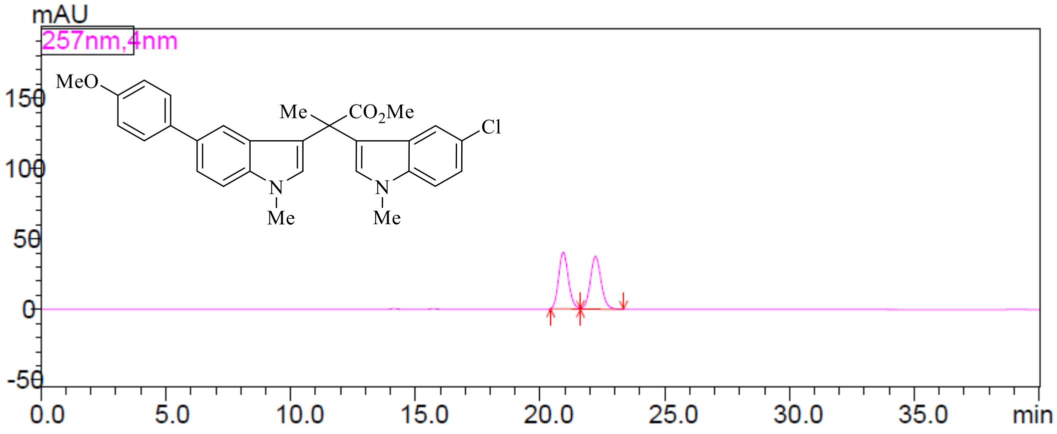

<Peak Table>

PDA Ch1 257nm

Peak#	Ret. Time	Area	Height	Area%	Peak Start	Peak End
1	20.928	1089673	40027	49.315	20.421	21.621
2	22.221	1119951	37389	50.685	21.621	23.349
Total		2209625	77416	100.000		

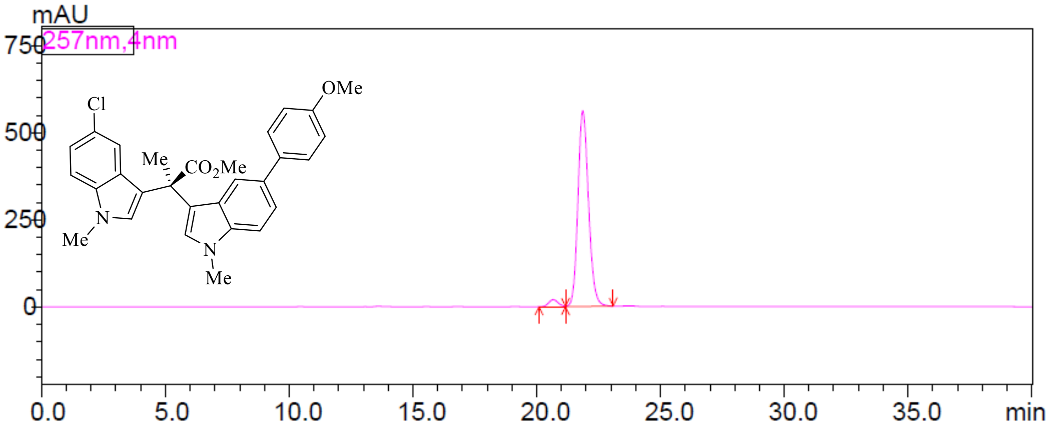

<Peak Table>

PDA Ch1 257nm

Peak#	Ret. Time	Area	Height	Area%	Peak Start	Peak End
1	20.671	533082	20061	3.095	20.096	21.163
2	21.864	16688395	560984	96.905	21.163	23.088
Total		17221477	581046	100.000		

38

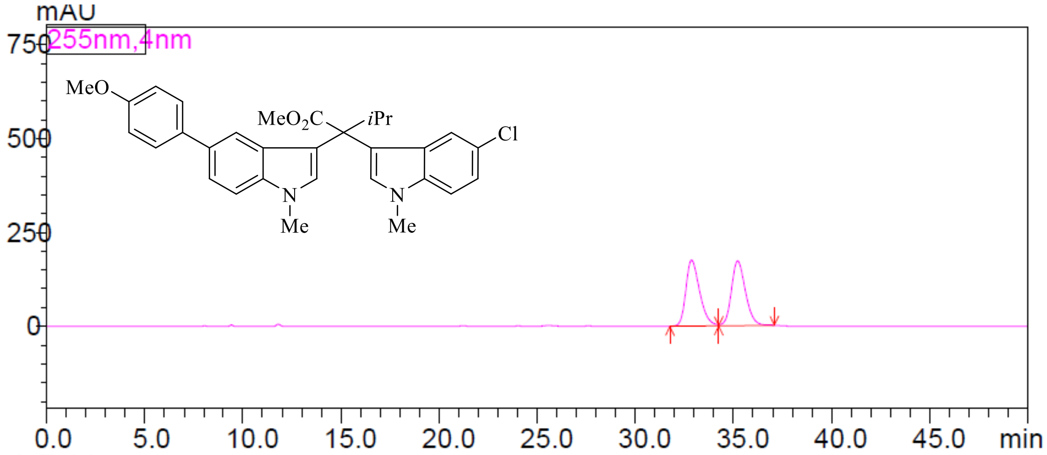

<Peak Table>

PDA Ch1 255nm

Peak#	Ret. Time	Area	Height	Area%	Peak Start	Peak End
1	32.869	8605428	175743	50.116	31.765	34.256
2	35.217	8565620	172485	49.884	34.256	37.088
Total		17171047	348228	100.000		

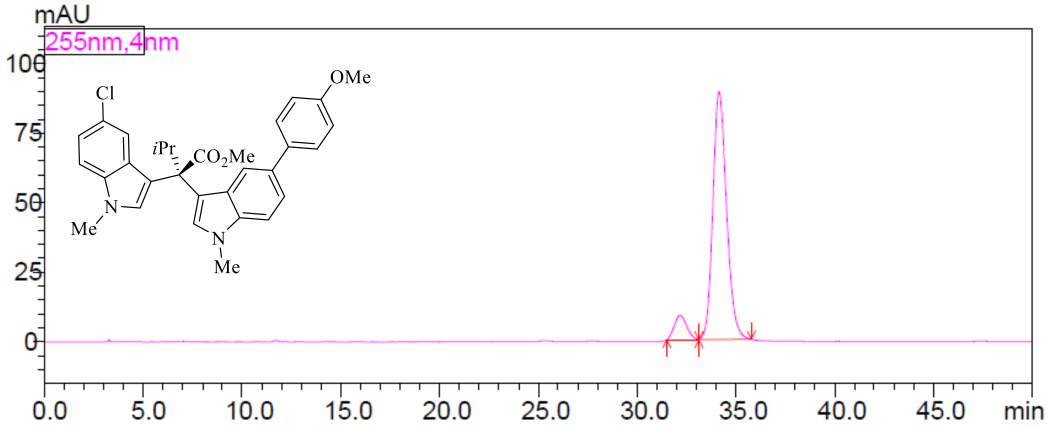

<Peak Table>

PDA Ch1 255nm

Peak#	Ret. Time	Area	Height	Area%	Peak Start	Peak End
1	32.171	380253	8760	8.134	31.499	33.120
2	34.148	4294536	89152	91.866	33.120	35.776
Total		4674789	97912	100.000		

39

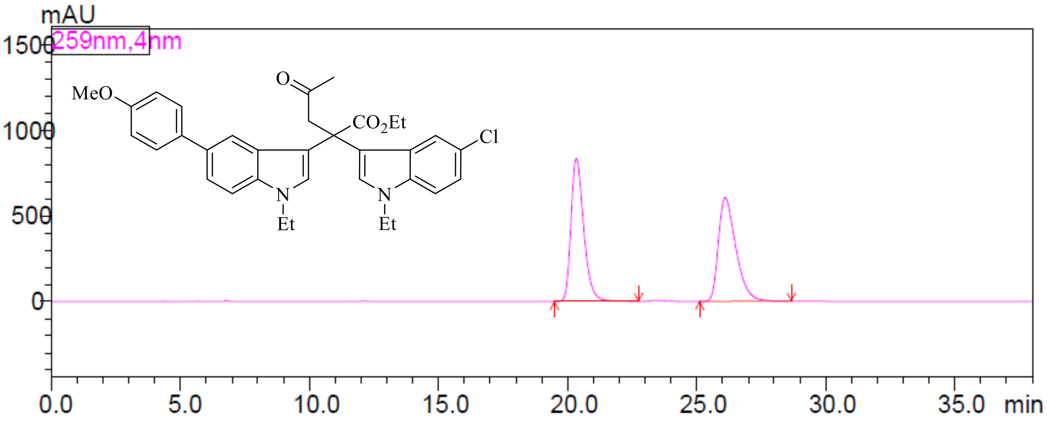

<Peak Table>

PDA Ch1 259nm

Peak#	Ret. Time	Area	Height	Area%	Peak Start	Peak End
1	20.334	28759351	836719	50.109	19.488	22.747
2	26.098	28634458	606790	49.891	25.115	28.661
Total		57393809	1443508	100.000		

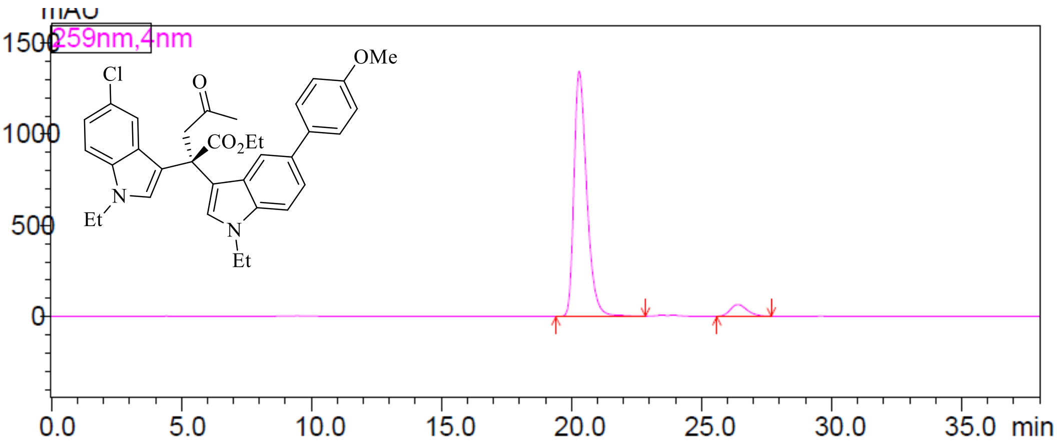

<Peak Table>

PDA Ch1 259nm

Peak#	Ret. Time	Area	Height	Area%	Peak Start	Peak End
1	20.294	45862225	1342786	93.707	19.397	22.837
2	26.391	3080056	65838	6.293	25.589	27.675
Total		48942280	1408625	100.000		

40

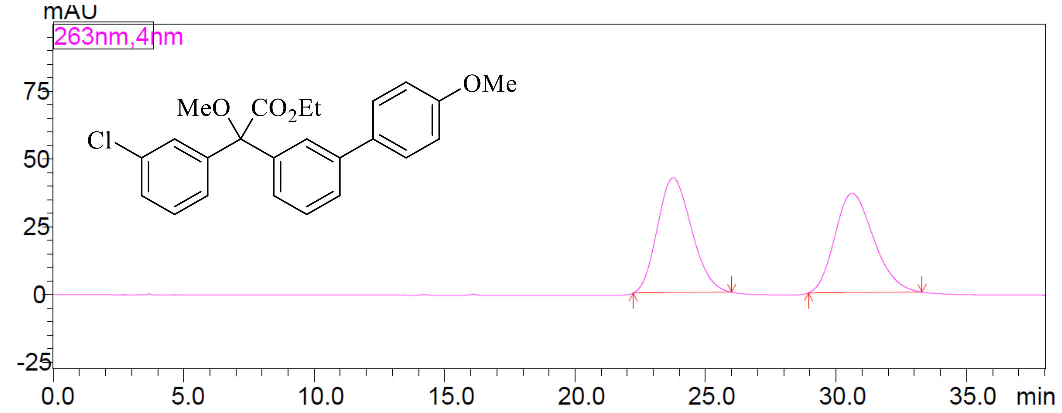

<Peak Table>

PDA Ch1 263nm

Peak#	Ret. Time	Area	Height	Area%	Peak Start	Peak End
1	23.758	3872820	42425	50.428	22.224	25.995
2	30.620	3807101	36616	49.572	28.944	33.301
Total		7679921	79041	100.000		

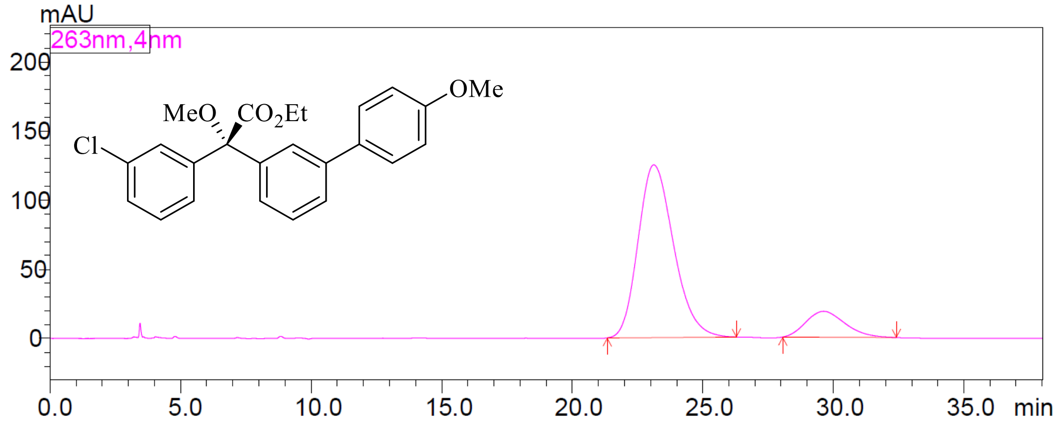

<Peak Table>

PDA Ch1 263nm

Peak#	Ret. Time	Area	Height	Area%	Peak Start	Peak End
1	23.123	11827836	124826	85.814	21.339	26.288
2	29.631	1955334	18742	14.186	28.059	32.416
Total		13783169	143569	100.000		

41

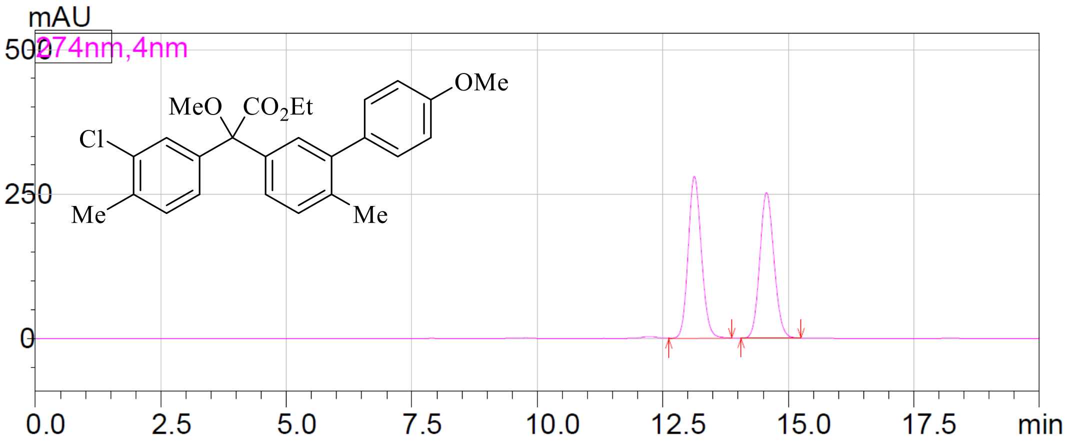

<Peak Table>

PDA Ch1 274nm

Peak#	Ret. Time	Area	Height	Area%	Peak Start	Peak End
1	13.126	4591772	250107	50.800	12.613	13.872
2	14.563	4447199	225100	49.200	14.059	15.253
Total		9038971	475207	100.000		

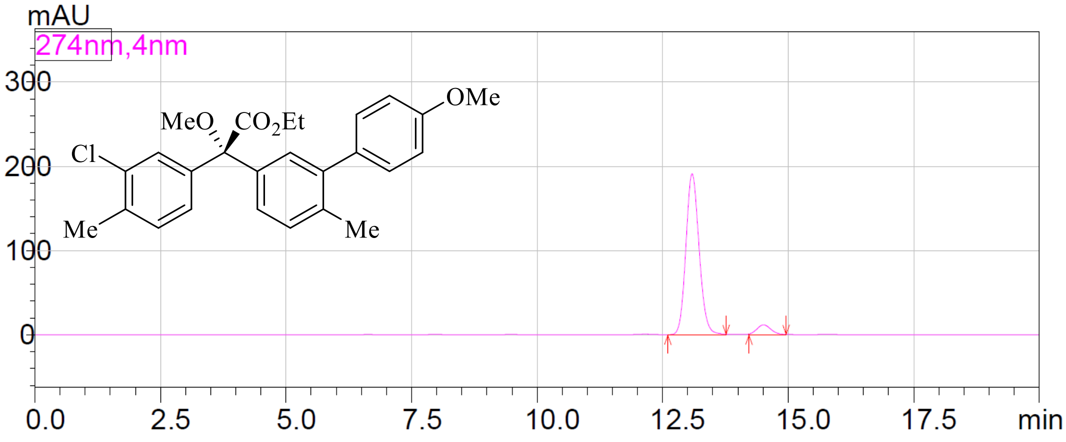

<Peak Table>

PDA Ch1 274nm

Peak#	Ret. Time	Area	Height	Area%	Peak Start	Peak End
1	13.084	3125875	170695	93.980	12.608	13.765
2	14.507	200225	10406	6.020	14.219	14.827
Total		3326100	181101	100.000		

42

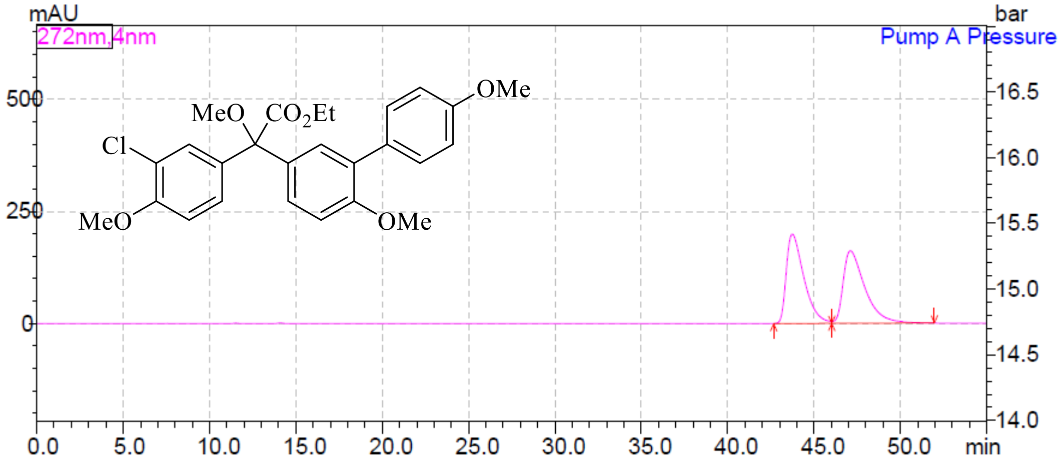

<Peak Table>

PDA Ch1 272nm

Peak#	Ret. Time	Area	Height	Area%	Peak Start	Peak End
1	43.739	14158052	199587	49.939	42.661	46.021
2	47.105	14192752	161779	50.061	46.021	51.968
Total		28350804	361367	100.000		

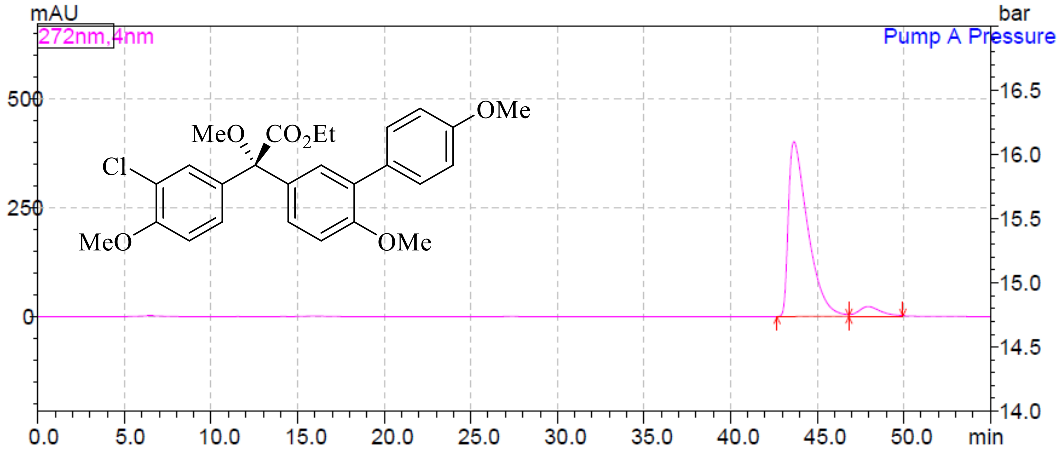

<Peak Table>

PDA Ch1 272nm

Peak#	Ret. Time	Area	Height	Area%	Peak Start	Peak End
1	43.664	31305715	401740	94.006	42.683	46.832
2	47.944	1996083	22230	5.994	46.832	49.957
Total		33301798	423970	100.000		

43

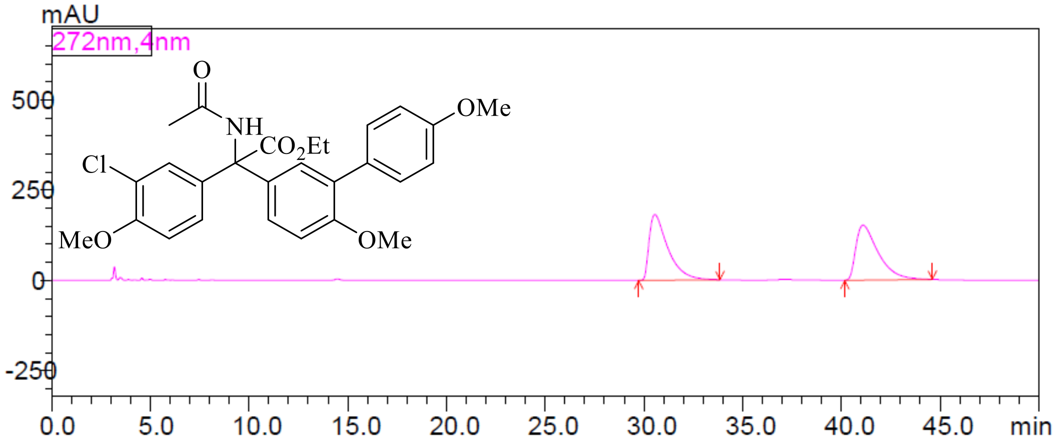

<Peak Table>

PDA Ch1 272nm

Peak#	Ret. Time	Area	Height	Area%	Peak Start	Peak End
1	30.551	12063966	181873	50.017	29.739	33.851
2	41.099	12055753	152055	49.983	40.176	44.603
Total		24119719	333929	100.000		

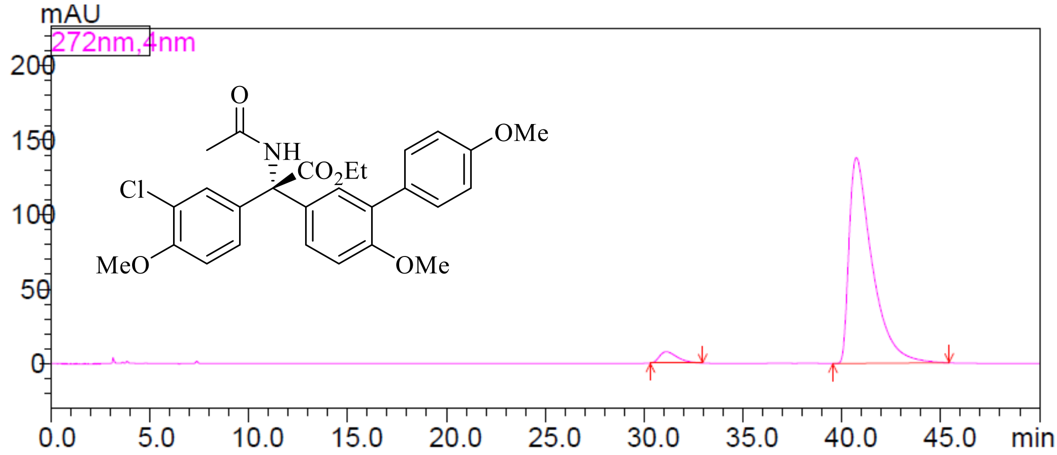

<Peak Table>

PDA Ch1 272nm

Peak#	Ret. Time	Area	Height	Area%	Peak Start	Peak End
1	31.113	477612	7635	4.065	30.315	32.955
2	40.735	11270641	138095	95.935	39.541	45.429
Total		11748252	145731	100.000		

44

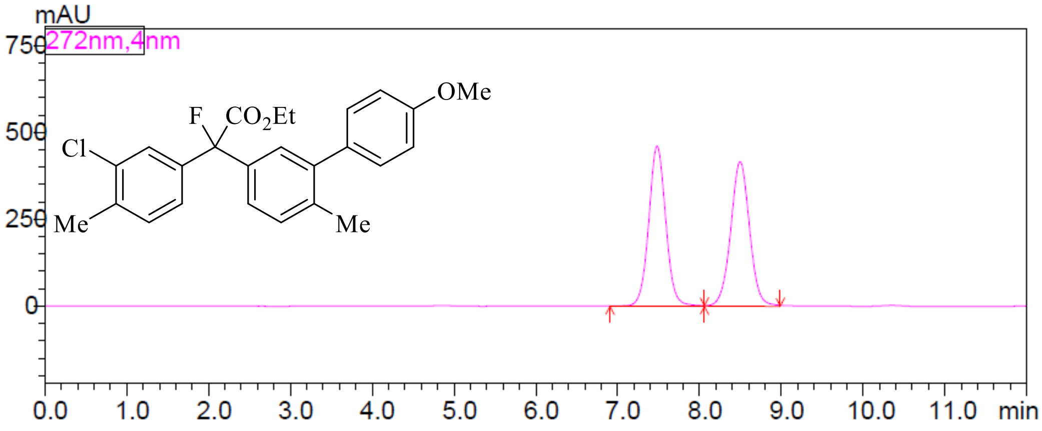

<Peak Table>

PDA Ch1 272nm

Peak#	Ret. Time	Area	Height	Area%	Peak Start	Peak End
1	7.479	6677461	460699	49.923	6.907	8.059
2	8.494	6697981	416214	50.077	8.059	8.987
Total		13375442	876913	100.000		

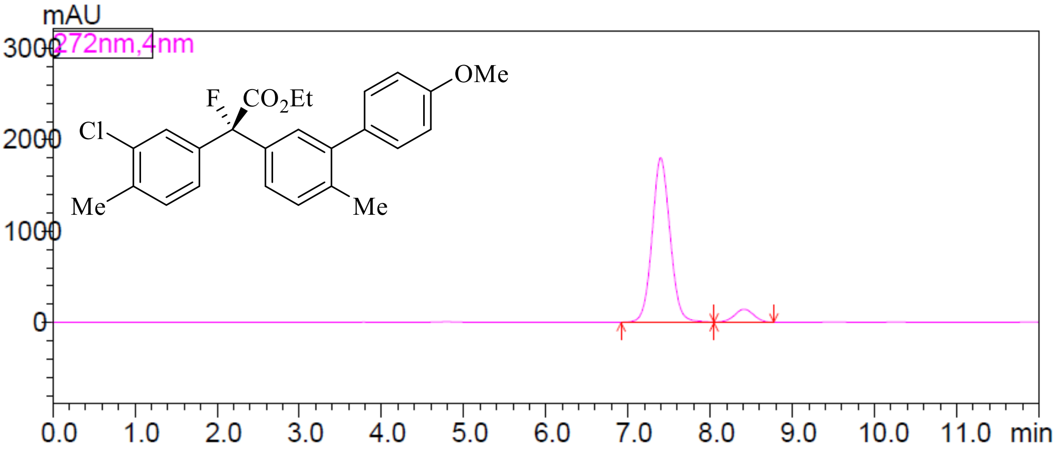

<Peak Table>

PDA Ch1 272nm

Peak#	Ret. Time	Area	Height	Area%	Peak Start	Peak End
1	7.395	27539848	1802771	91.683	6.917	8.048
2	8.410	2498377	146518	8.317	8.048	8.773
Total		30038224	1949289	100.000		

45

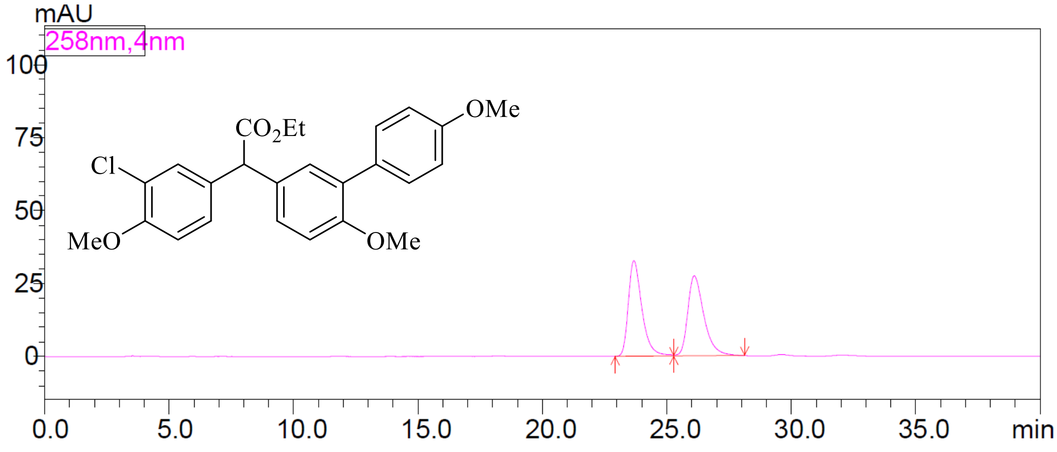

<Peak Table>

PDA Ch1 258nm

Peak#	Ret. Time	Area	Height	Area%	Peak Start	Peak End
1	23.675	1247344	32784	50.109	22.917	25.275
2	26.102	1241921	27443	49.891	25.275	28.123
Total		2489264	60227	100.000		

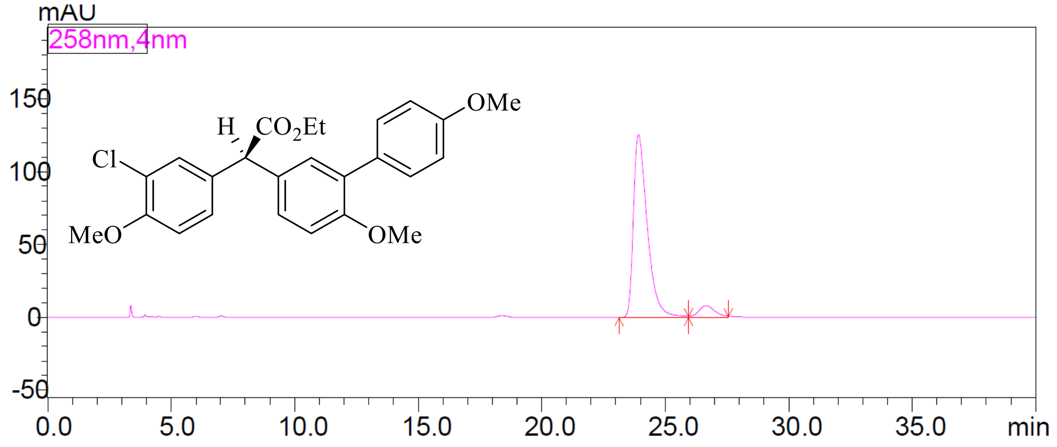

<Peak Table>

PDA Ch1 258nm

Peak#	Ret. Time	Area	Height	Area%	Peak Start	Peak End
1	23.919	4889290	125522	93.030	23.131	25.941
2	26.651	366331	7959	6.970	25.941	27.557
Total		5255621	133481	100.000		

46

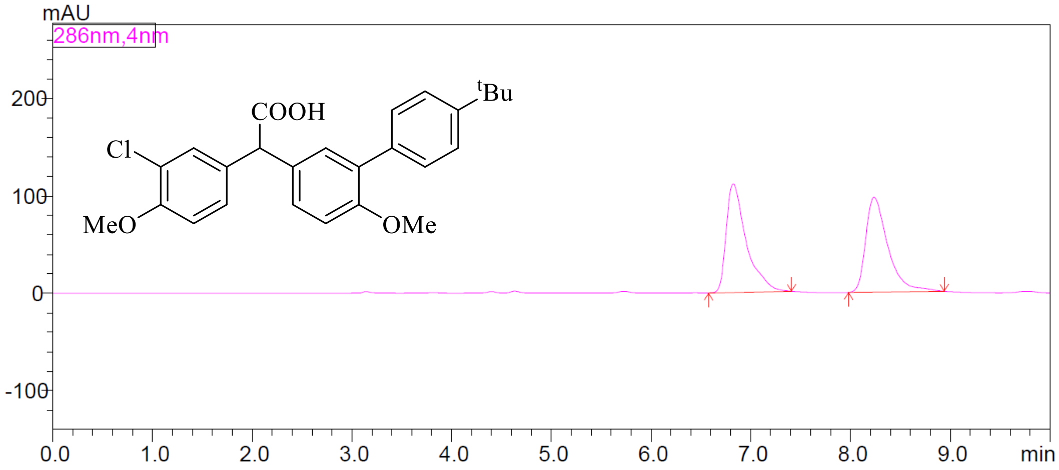

<Peak Table>

PDA Ch1 286nm

Peak#	Ret. Time	Area	Height	Area%	Peak Start	Peak End
1	6.824	1560392	111700	50.812	6.576	7.408
2	8.236	1510527	97564	49.188	7.979	8.939
Total		3070918	209264	100.000		

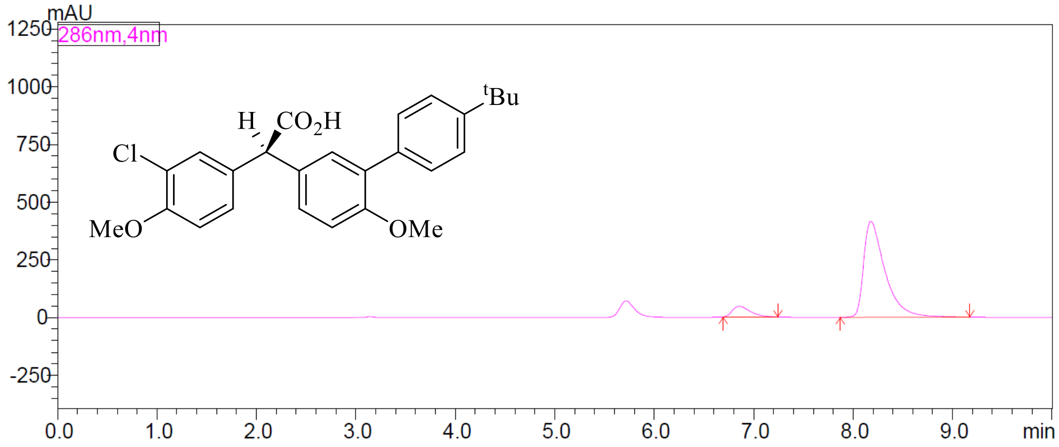

<Peak Table>

PDA Ch1 286nm

Peak#	Ret. Time	Area	Height	Area%	Peak Start	Peak End
1	6.855	584899	46399	8.587	6.693	7.243
2	8.179	6226907	416102	91.413	7.872	9.173
Total		6811806	462501	100.000		

47

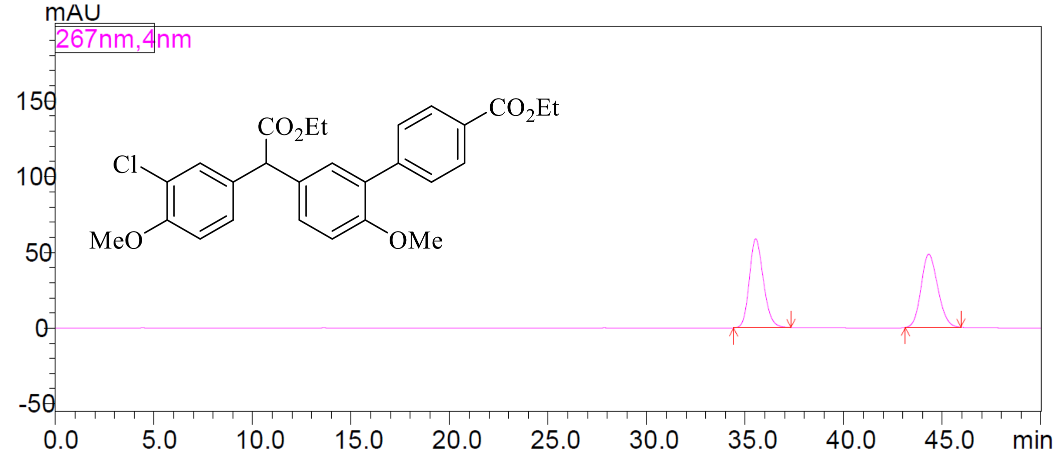

<Peak Table>

PDA Ch1 267nm

Peak#	Ret. Time	Area	Height	Area%	Peak Start	Peak End
1	35.541	2920989	58698	50.547	34.421	37.323
2	44.320	2857772	48401	49.453	43.136	45.952
Total		5778761	107100	100.000		

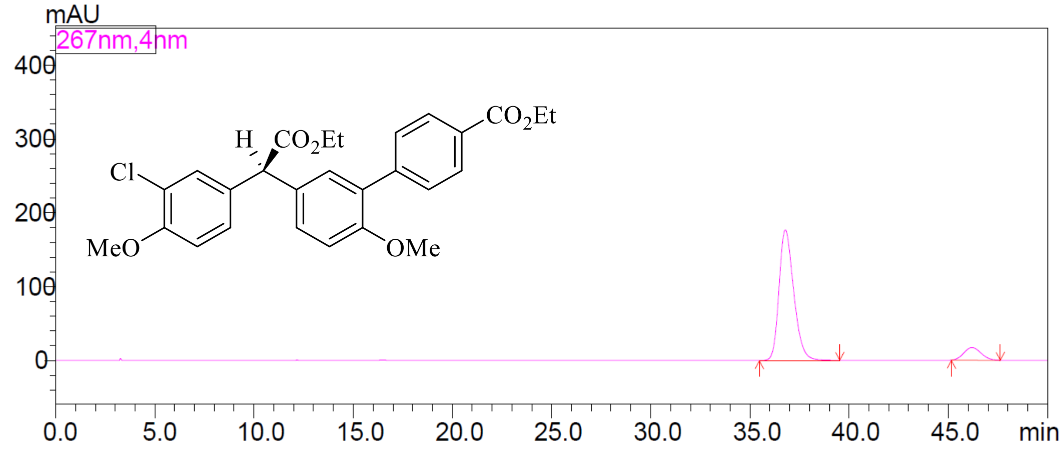

<Peak Table>

PDA Ch1 267nm

Peak#	Ret. Time	Area	Height	Area%	Peak Start	Peak End
1	36.783	9119939	176981	89.713	35.477	39.525
2	46.192	1045727	17271	10.287	45.157	47.621
Total		10165666	194252	100.000		

48

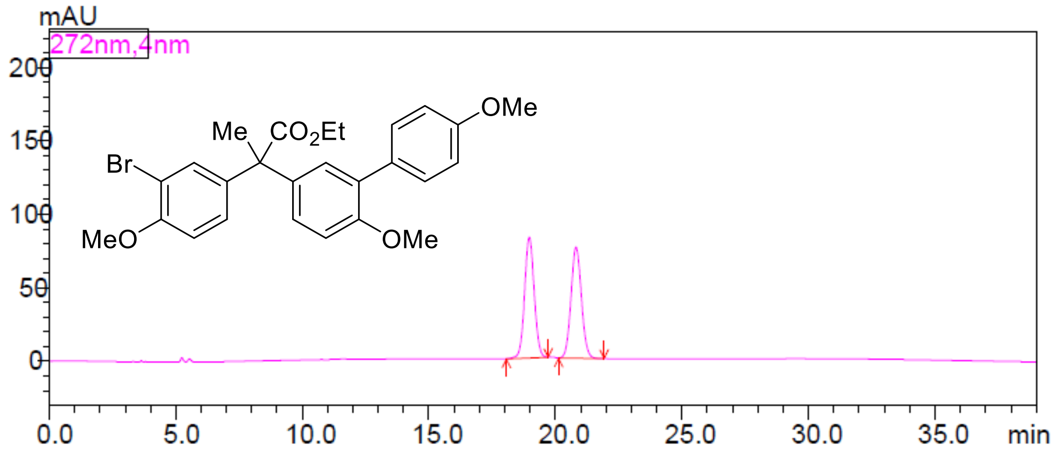

<Peak Table>

PDA Ch1 272nm

Peak#	Ret. Time	Area	Height	Area%	Peak Start	Peak End
1	18.965	2170477	82173	49.937	18.059	19.707
2	20.812	2175931	75639	50.063	20.128	21.920
Total		4346407	157811	100.000		

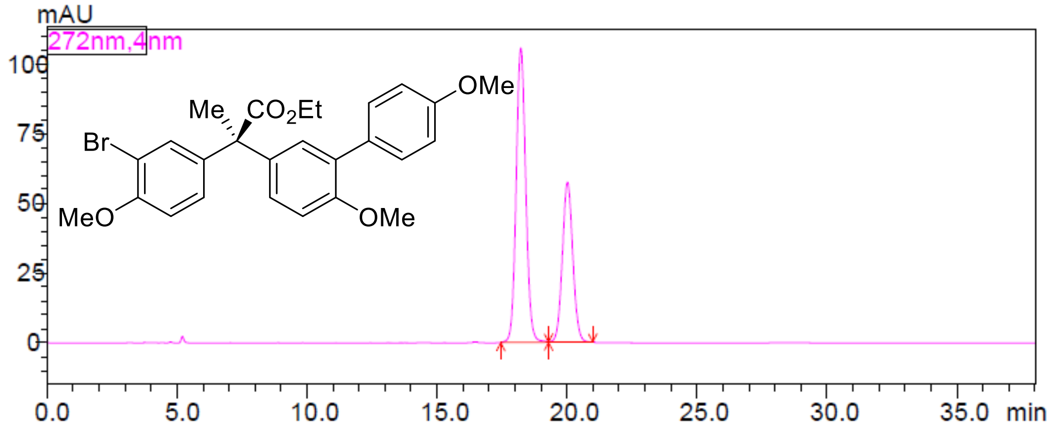

<Peak Table>

PDA Ch1 272nm

Peak#	Ret. Time	Area	Height	Area%	Peak Start	Peak End
1	18.221	2701863	105517	62.677	17.451	19.285
2	20.017	1608884	57381	37.323	19.285	20.987
Total		4310747	162898	100.000		

49

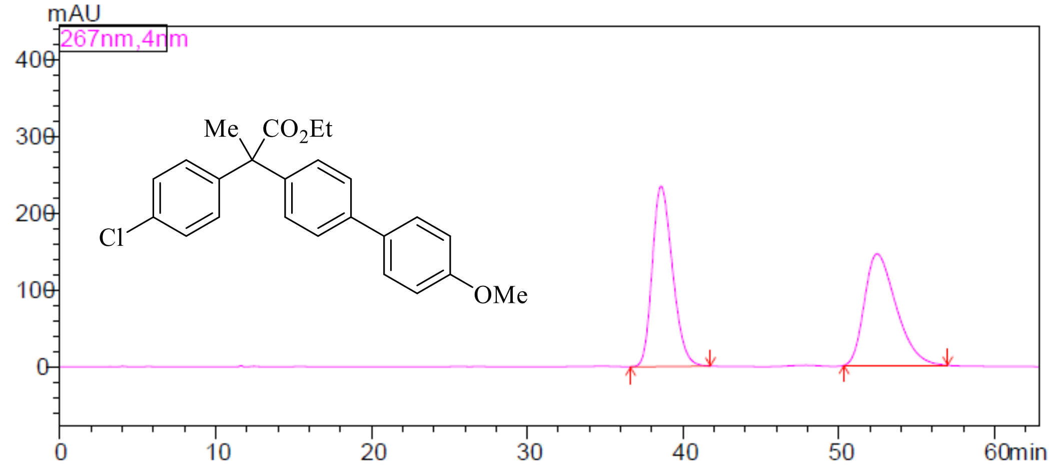

<Peak Table>

PDA Ch1 267nm

Peak#	Ret. Time	Area	Height	Area%	Peak Start	Peak End
1	38.592	21242099	234628	50.820	36.629	41.755
2	52.454	20556770	145039	49.180	50.331	56.992
Total		41798869	379668	100.000		

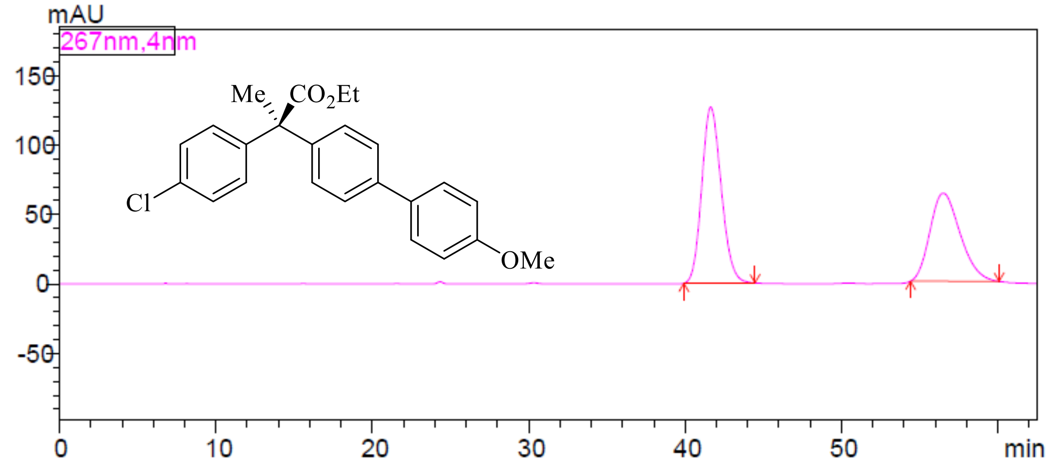

<Peak Table>

PDA Ch1 267nm

Peak#	Ret. Time	Area	Height	Area%	Peak Start	Peak End
1	41.647	10990163	126695	56.296	39.936	44.480
2	56.514	8531919	63386	43.704	54.432	60.085
Total		19522081	190082	100.000		

50

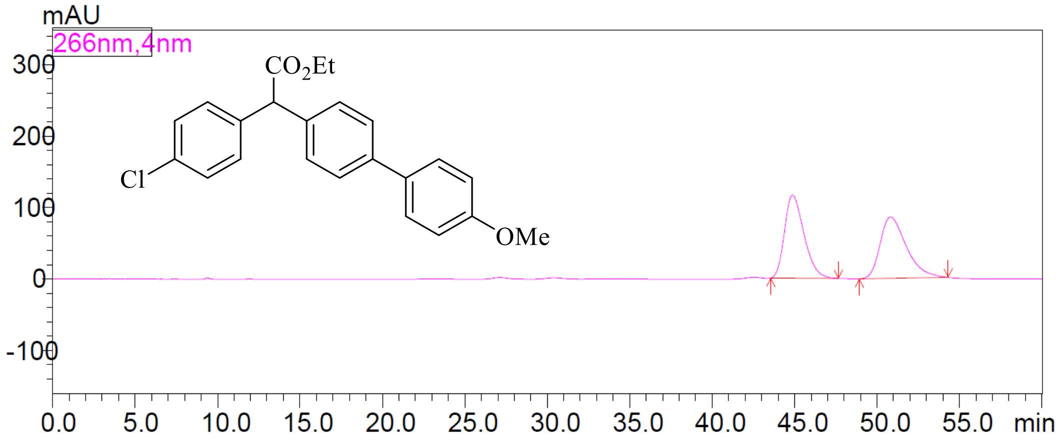

<Peak Table>

PDA Ch1 266nm

Peak#	Ret. Time	Area	Height	Area%	Peak Start	Peak End
1	44.879	9550916	116125	50.166	43.552	47.664
2	50.824	9487653	85814	49.834	48.928	54.304
Total		19038569	201939	100.000		

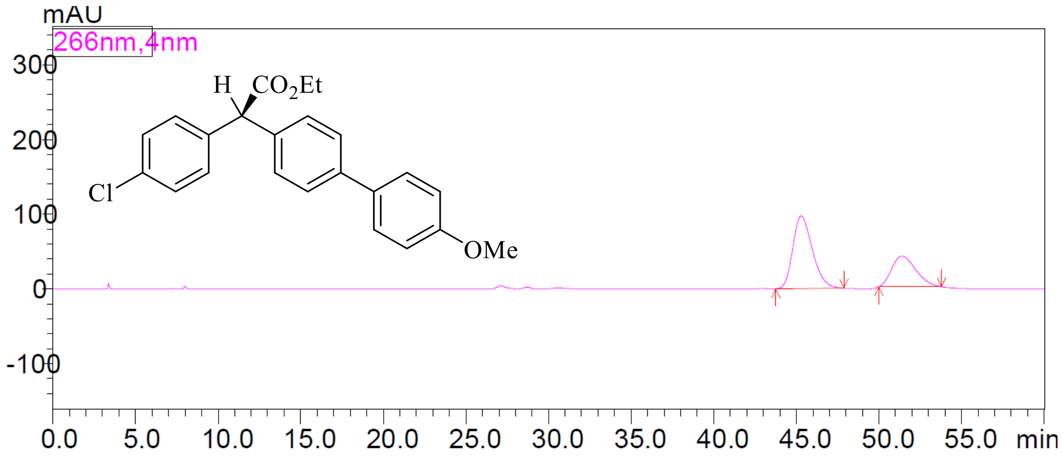

<Peak Table>

PDA Ch1 266nm

Peak#	Ret. Time	Area	Height	Area%	Peak Start	Peak End
1	45.290	8017139	97286	65.506	43.760	47.872
2	51.398	4221722	41420	34.494	49.984	53.781
Total		12238861	138706	100.000		

52

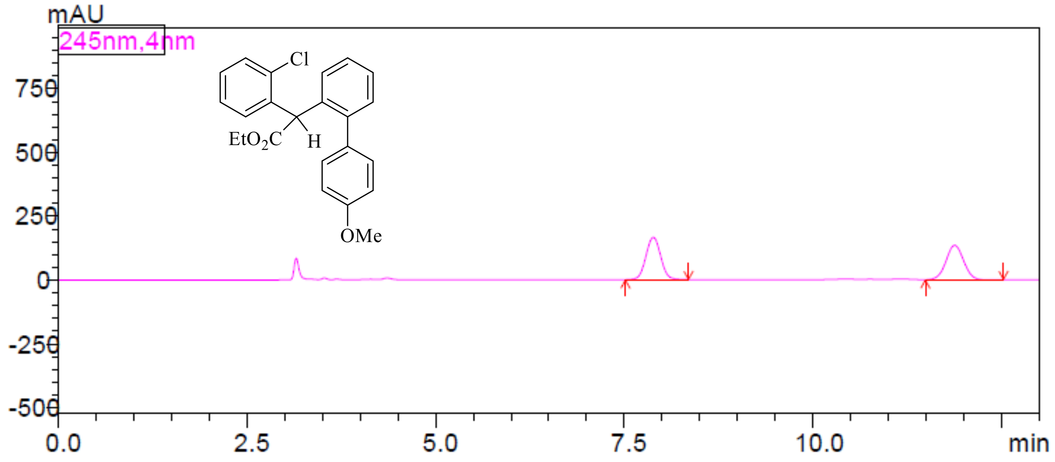

<Peak Table>

PDA Ch1 245nm

Peak#	Ret. Time	Area	Height	Area%	Peak Start	Peak End
1	7.885	2250082	165219	50.686	7.515	8.352
2	11.880	2189178	135161	49.314	11.493	12.528
Total		4439260	300380	100.000		

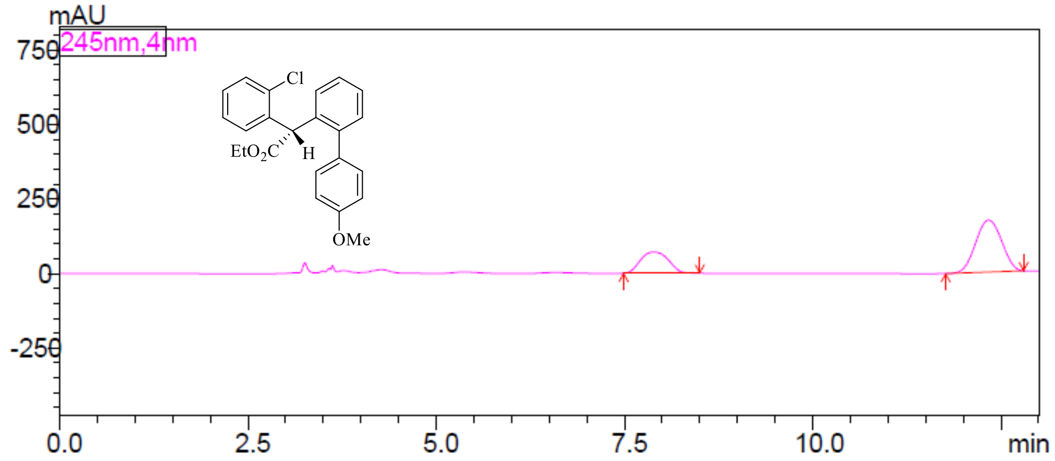

<Peak Table>

PDA Ch1 245nm

Peak#	Ret. Time	Area	Height	Area%	Peak Start	Peak End
1	7.889	1840433	71345	30.470	7.488	8.501
2	12.336	4199729	174583	69.530	11.765	12.800
Total		6040161	245928	100.000		

53

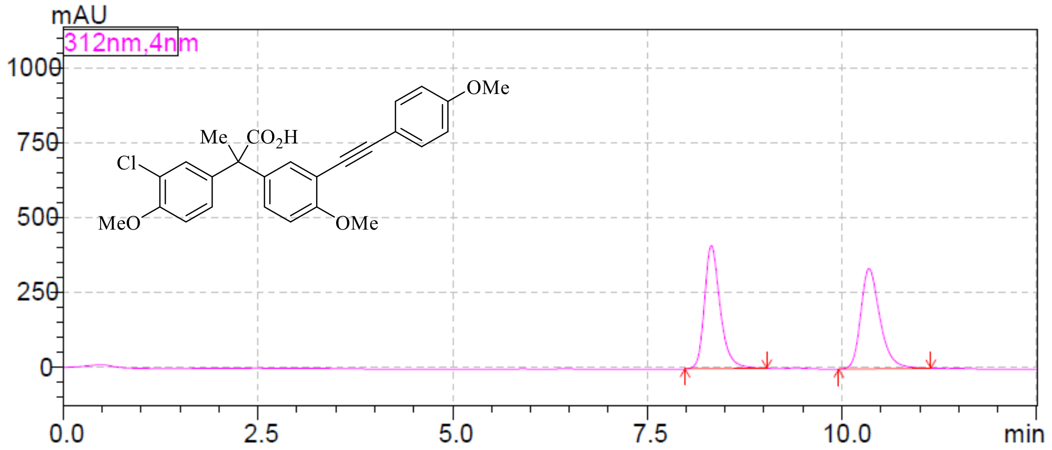

<Peak Table>

PDA Ch1 312nm

Peak#	Ret. Time	Area	Height	Peak Start	Peak End	Area%
1	8.323	5784629	410842	7.984	9.035	50.142
2	10.348	5751830	334127	9.957	11.141	49.858
Total		11536459	744969			100.000

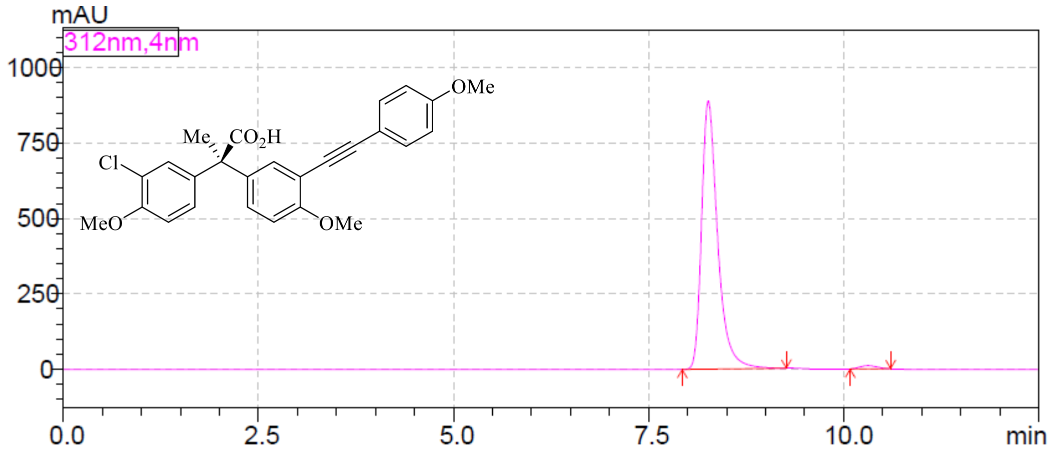

<Peak Table>

PDA Ch1 312nm

Peak#	Ret. Time	Area	Height	Peak Start	Peak End	Area%
1	8.266	12720584	889062	7.931	9.264	98.720
2	10.313	164941	10715	10.085	10.603	1.280
Total		12885525	899777			100.000

54

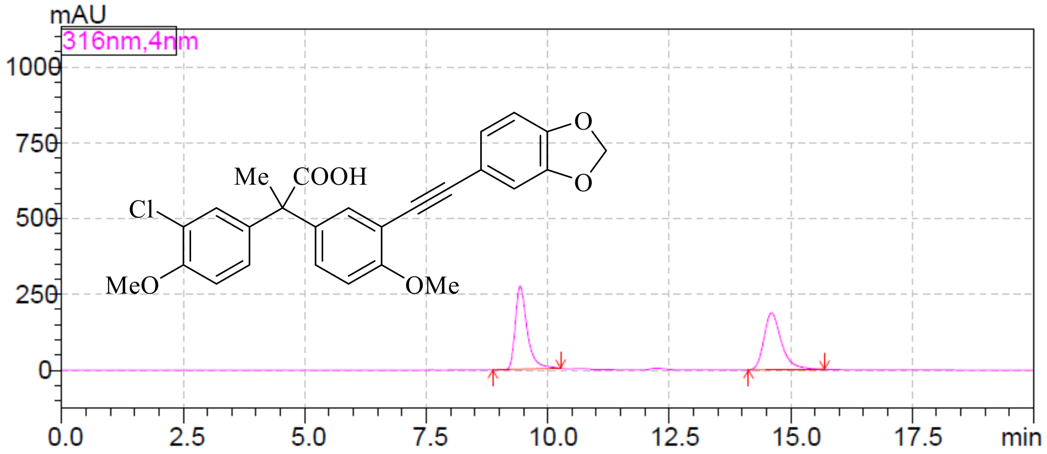

<Peak Table>

PDA Ch1 316nm

Peak#	Ret. Time	Area	Height	Area%	Peak Start	Peak End
1	9.434	4690328	274311	50.068	8.869	10.267
2	14.606	4677537	187227	49.932	14.128	15.691
Total		9367865	461538	100.000		

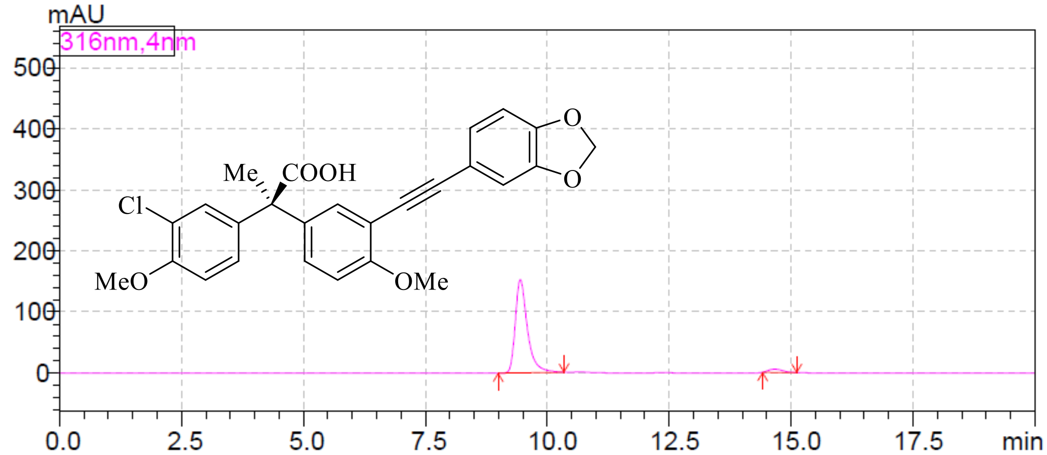

<Peak Table>

PDA Ch1 316nm

Peak#	Ret. Time	Area	Height	Area%	Peak Start	Peak End
1	9.450	2628052	152664	96.104	8.992	10.347
2	14.665	106550	5242	3.896	14.416	15.115
Total		2734601	157906	100.000		

55

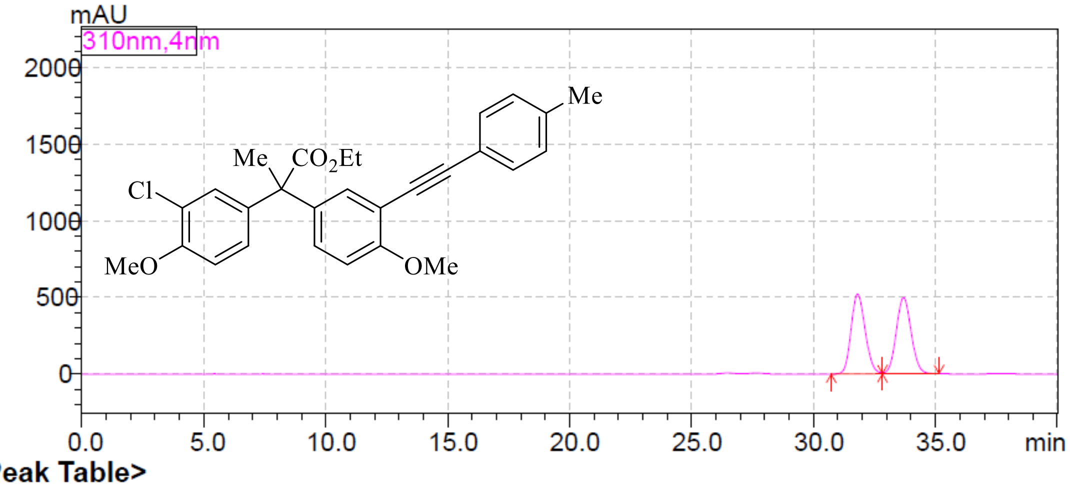

<Peak Table>

PDA Ch1 310nm

Peak#	Ret. Time	Area	Height	Area%	Peak Start	Peak End
1	31.808	20999977	520804	49.736	30.720	32.821
2	33.691	21223148	498274	50.264	32.821	35.152
Total		42223125	1019078	100.000		

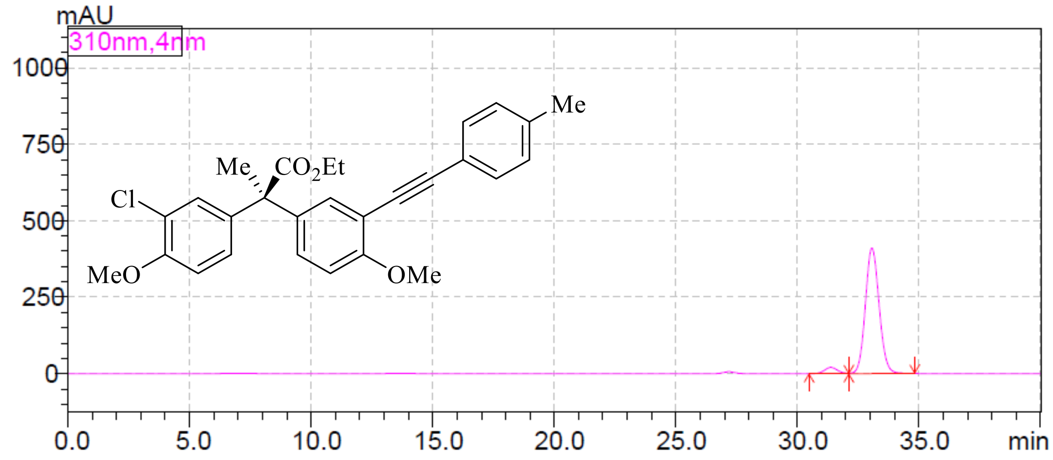

<Peak Table>

PDA Ch1 310nm

Peak#	Ret. Time	Area	Height	Peak Start	Peak End	Area%
1	31.378	751915	20340	30.475	32.117	4.447
2	33.068	16156044	410791	32.117	34.827	95.553
Total		16907959	431131			100.000

56

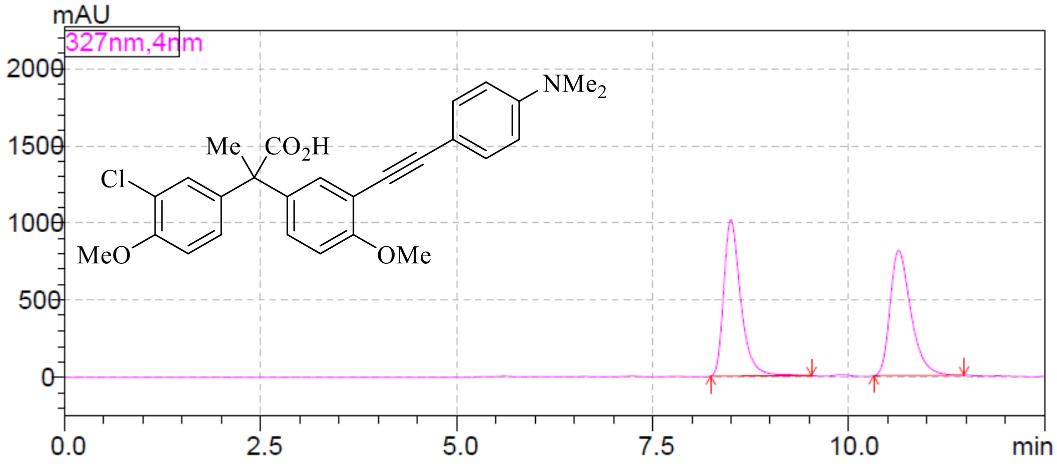

<Peak Table>

PDA Ch1 327nm

Peak#	Ret. Time	Area	Height	Peak Start	Peak End	Area%
1	8.497	15324236	1013893	8.240	9.525	50.477
2	10.637	15034649	812052	10.320	11.472	49.523
Total		30358885	1825945			100.000

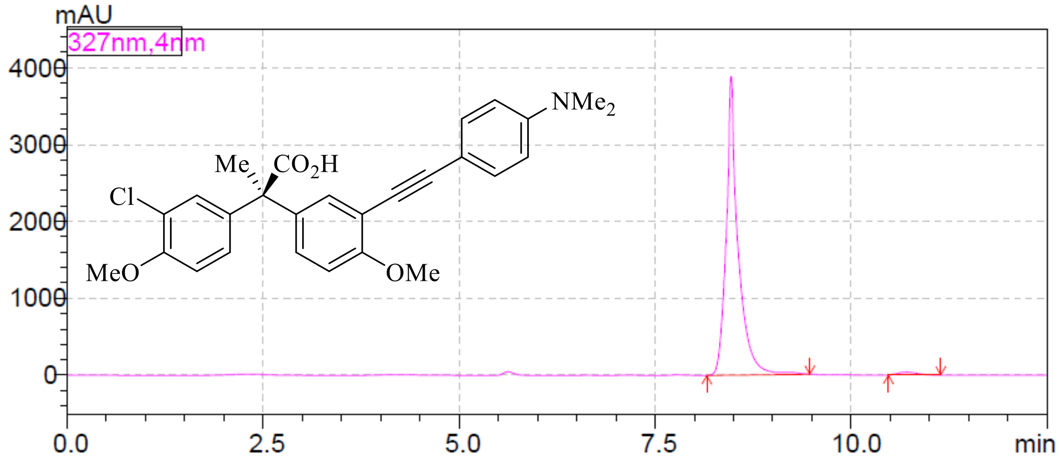

<Peak Table>

PDA Ch1 327nm

Peak#	Ret. Time	Area	Height	Peak Start	Peak End	Area%
1	8.468	41100597	3887425	8.160	9.472	98.434
2	10.708	654072	38538	10.475	11.141	1.566
Total		41754669	3925963			100.000

57

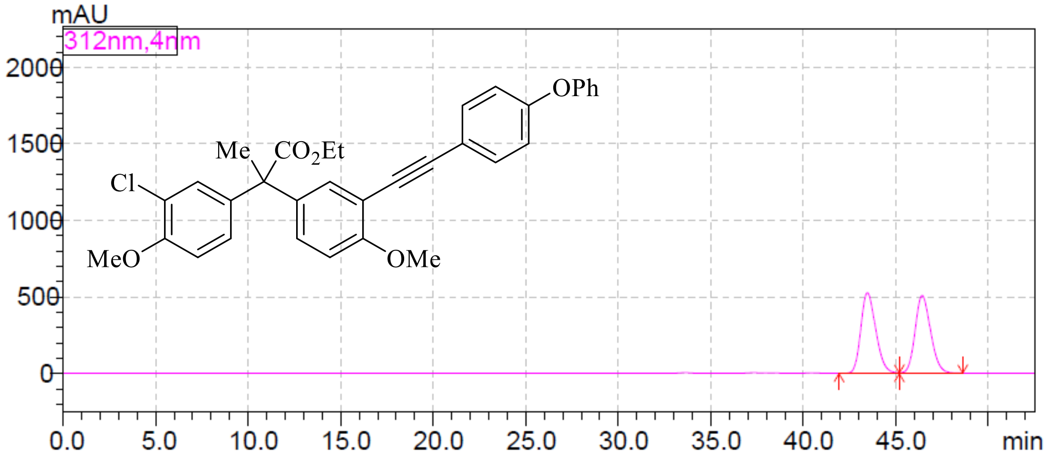

<Peak Table>

PDA Ch1 312nm

Peak#	Ret. Time	Area	Height	Area%	Peak Start	Peak End
1	43.460	29793620	526980	50.100	41.931	45.184
2	46.420	29675085	507733	49.900	45.184	48.608
Total		59468705	1034713	100.000		

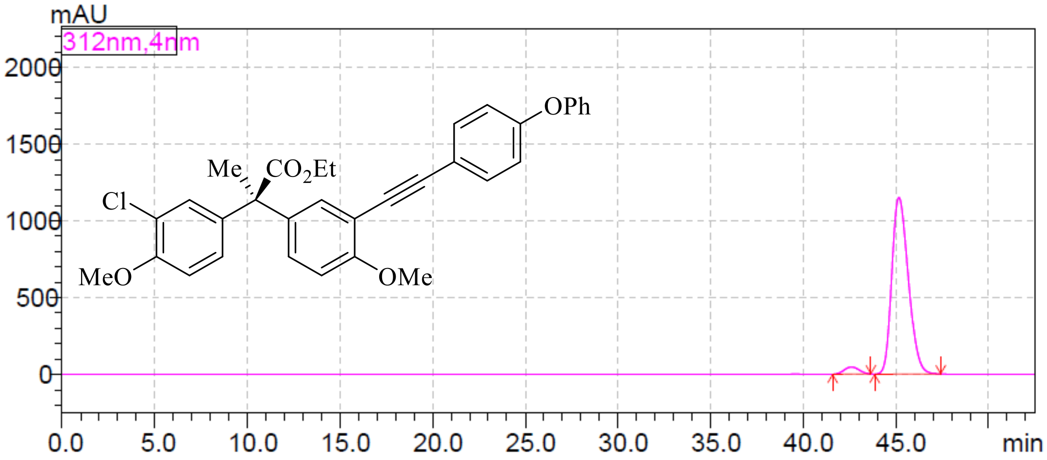

<Peak Table>

PDA Ch1 312nm

Peak#	Ret. Time	Area	Height	Area%	Peak Start	Peak End
1	42.604	2593568	46523	3.507	41.589	43.643
2	45.164	71365280	1150082	96.493	43.856	47.424
Total		73958848	1196605	100.000		

58

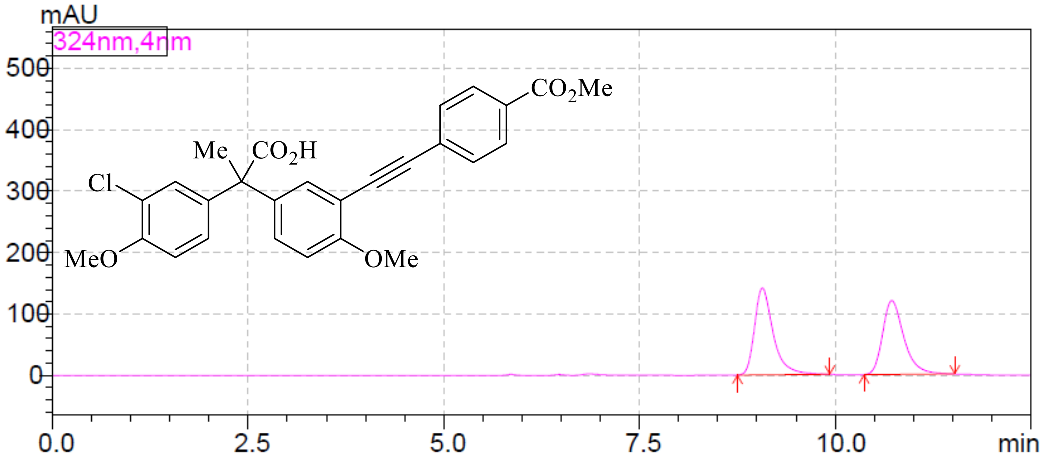

<Peak Table>

PDA Ch1 324nm

Peak#	Ret. Time	Area	Height	Peak Start	Peak End	Area%
1	9.070	2350219	141286	8.752	9.931	50.761
2	10.723	2279760	120233	10.368	11.525	49.239
Total		4629979	261519			100.000

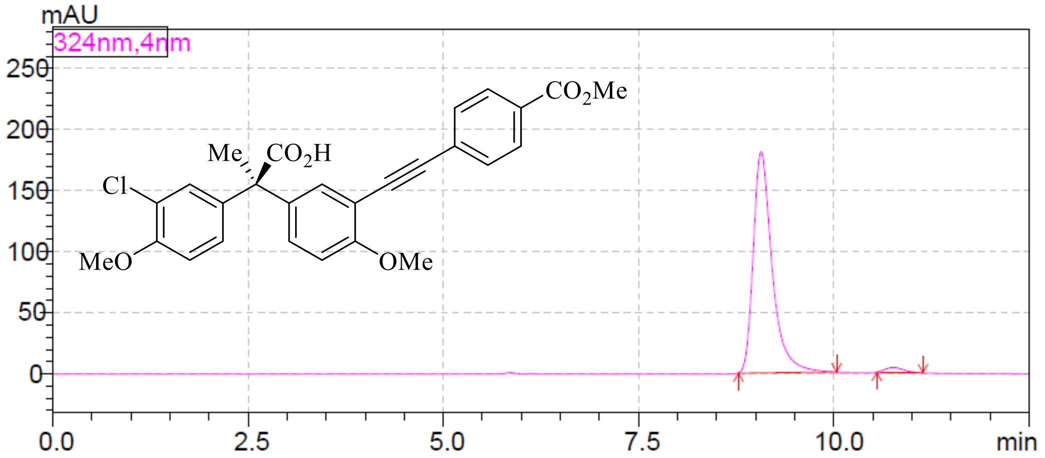

<Peak Table>

PDA Ch1 324nm

Peak#	Ret. Time	Area	Height	Peak Start	Peak End	Area%
1	9.069	3041052	180824	8.779	10.037	97.813
2	10.761	67987	4102	10.549	11.141	2.187
Total		3109039	184926			100.000

59

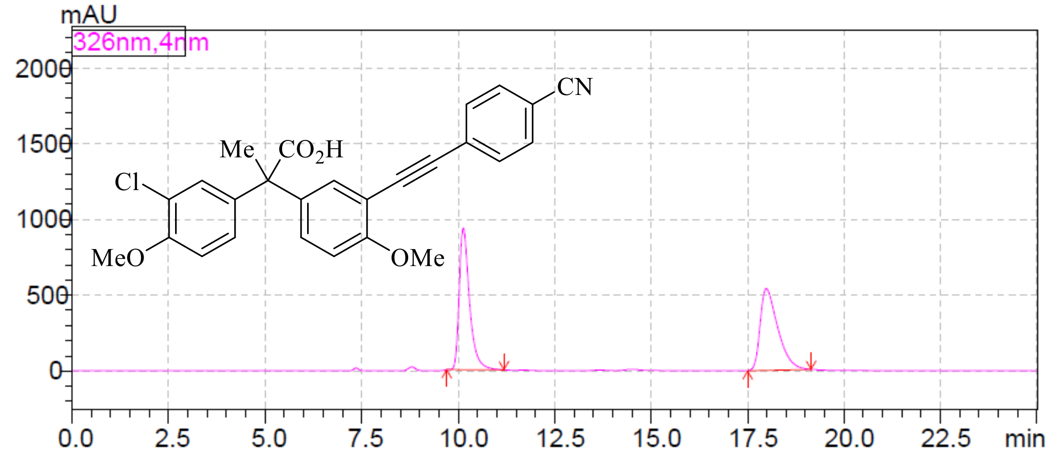

<Peak Table>

PDA Ch1 326nm

Peak#	Ret. Time	Area	Peak Start	Peak End	Area%
1	10.135	17355350	9.701	11.189	50.337
2	17.987	17122726	17.504	19.147	49.663
Total		34478076			100.000

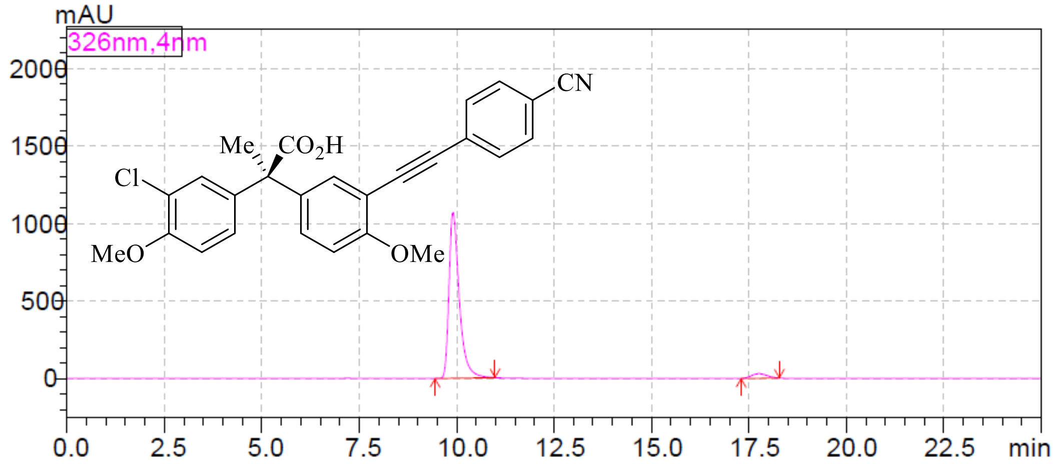

<Peak Table>

PDA Ch1 326nm

Peak#	Ret. Time	Area	Peak Start	Peak End	Area%	Height
1	9.907	19621525	9.445	10.987	96.038	1070278
2	17.757	809518	17.301	18.277	3.962	29438
Total		20431043			100.000	1099716

60

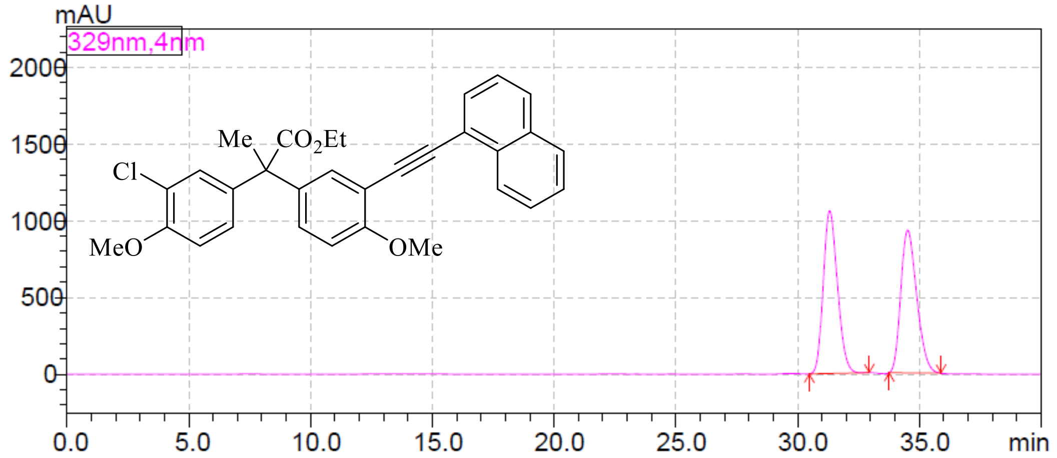

<Peak Table>

PDA Ch1 329nm

Peak#	Ret. Time	Area	Height	Peak Start	Peak End	Area%
1	31.319	43115639	1060072	30.475	32.939	50.372
2	34.519	42479311	930549	33.760	35.893	49.628
Total		85594950	1990622			100.000

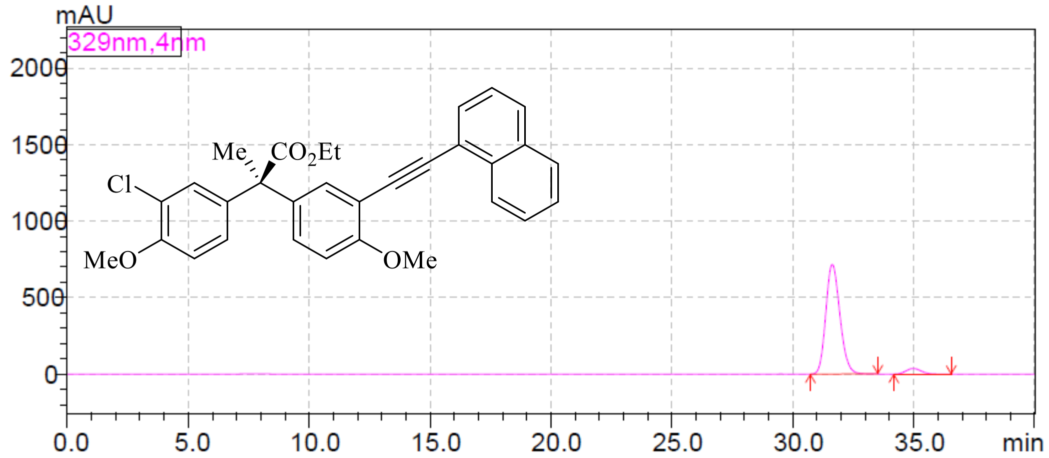

<Peak Table>

PDA Ch1 329nm

Peak#	Ret. Time	Area	Height	Area%	Peak Start	Peak End
1	31.633	29151306	716881	94.831	30.720	33.509
2	34.983	1588859	36507	5.169	34.171	36.549
Total		30740165	753387	100.000		

61

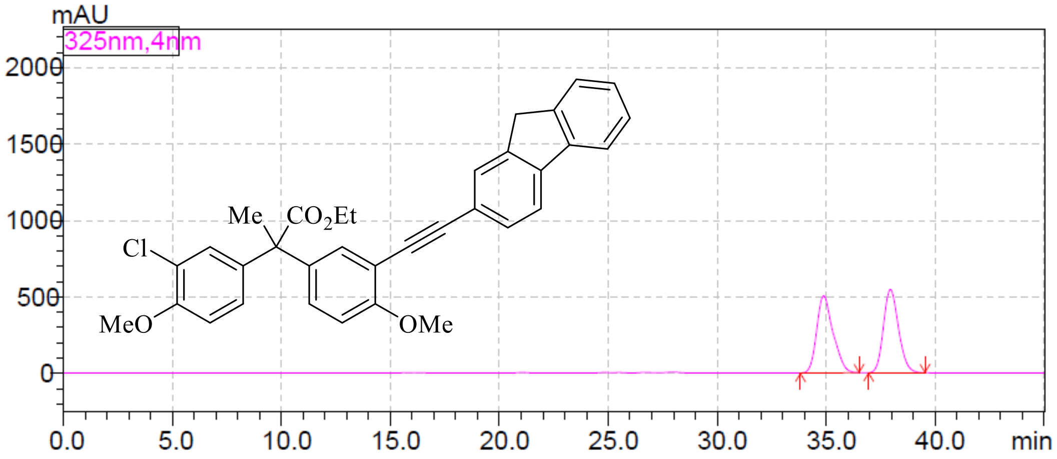

<Peak Table>

PDA Ch1 325nm

Peak#	Ret. Time	Area	Height	Area%	Peak Start	Peak End
1	34.885	26353487	504003	50.055	33.813	36.512
2	37.944	26295830	545425	49.945	36.944	39.536
Total		52649317	1049428	100.000		

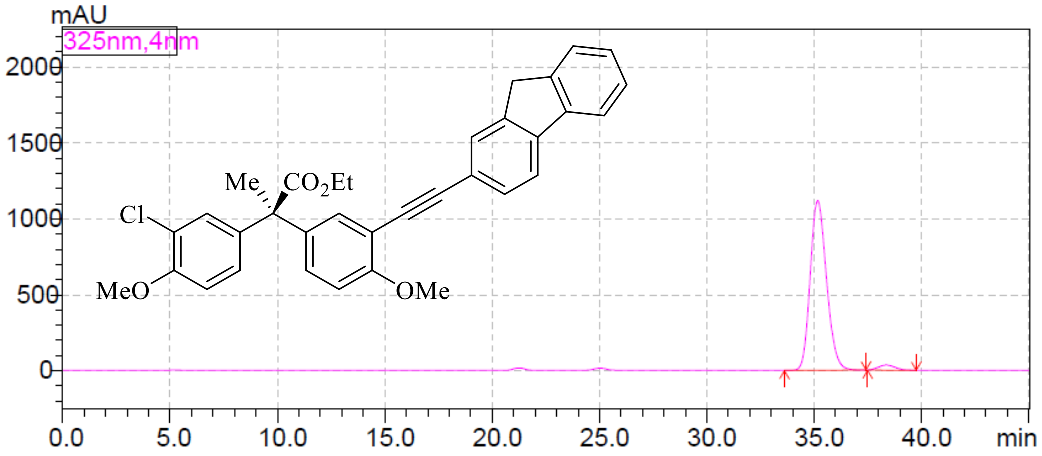

<Peak Table>

PDA Ch1 325nm

Peak#	Ret. Time	Area	Height	Area%	Peak Start	Peak End
1	35.160	59181276	1118487	96.985	33.611	37.408
2	38.354	1839519	33749	3.015	37.483	39.755
Total		61020795	1152235	100.000		

62

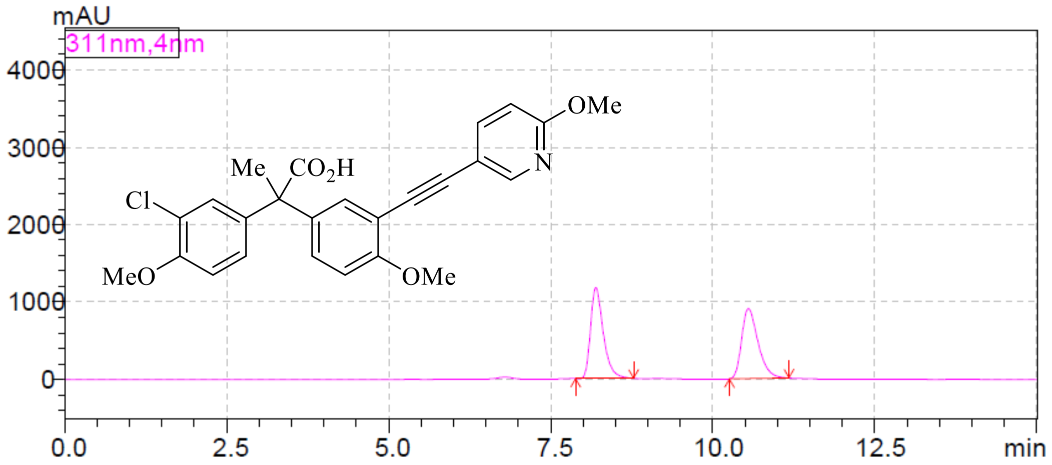

<Peak Table>

PDA Ch1 311nm

Peak#	Ret. Time	Area	Height	Peak Start	Peak End	Area%
1	8.197	16078563	1172442	7.883	8.779	50.181
2	10.551	15962449	905216	10.256	11.179	49.819
Total		32041012	2077658			100.000

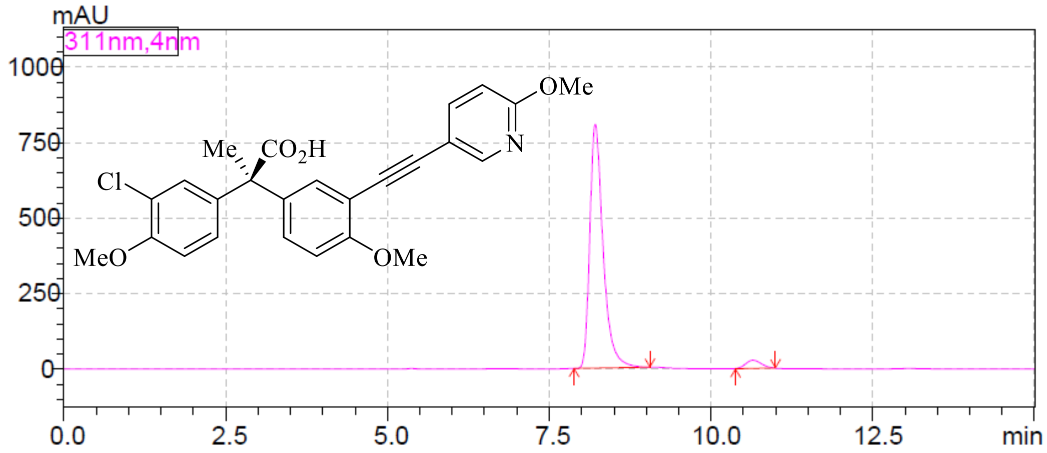

<Peak Table>

PDA Ch1 311nm

Peak#	Ret. Time	Area	Height	Peak Start	Peak End	Area%
1	8.212	11576223	807396	7.883	9.056	96.210
2	10.650	456015	27128	10.379	10.997	3.790
Total		12032238	834524			100.000

63

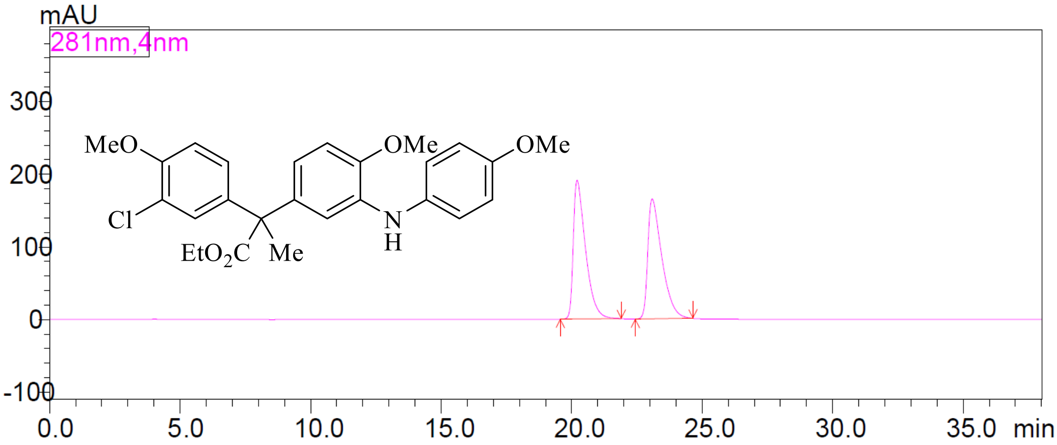

<Peak Table>

PDA Ch1 281nm

Peak#	Ret. Time	Area	Height	Area%	Peak Start	Peak End
1	20.212	6217387	191056	50.069	19.568	21.904
2	23.087	6200180	165079	49.931	22.437	24.645
Total		12417567	356134	100.000		

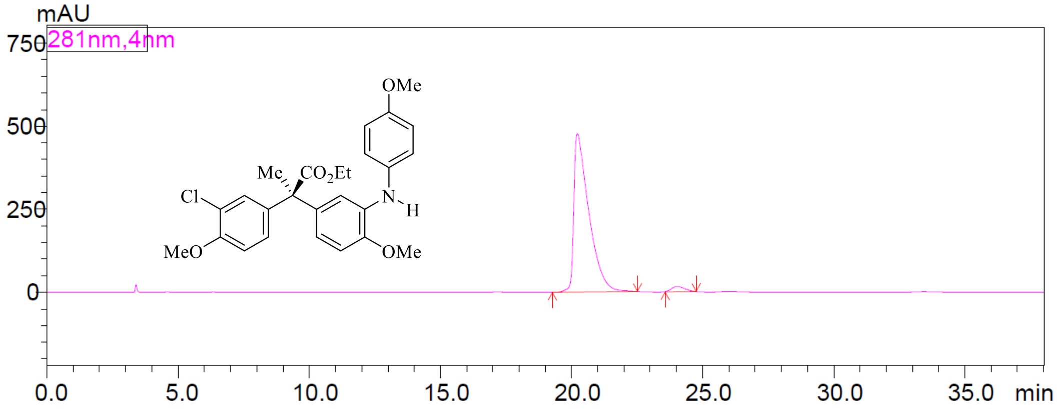

<Peak Table>

PDA Ch1 281nm

Peak#	Ret. Time	Area	Height	Area%	Peak Start	Peak End
1	20.225	18974263	476096	97.251	19.275	22.512
2	24.031	536255	15686	2.749	23.568	24.757
Total		19510518	491783	100.000		

64

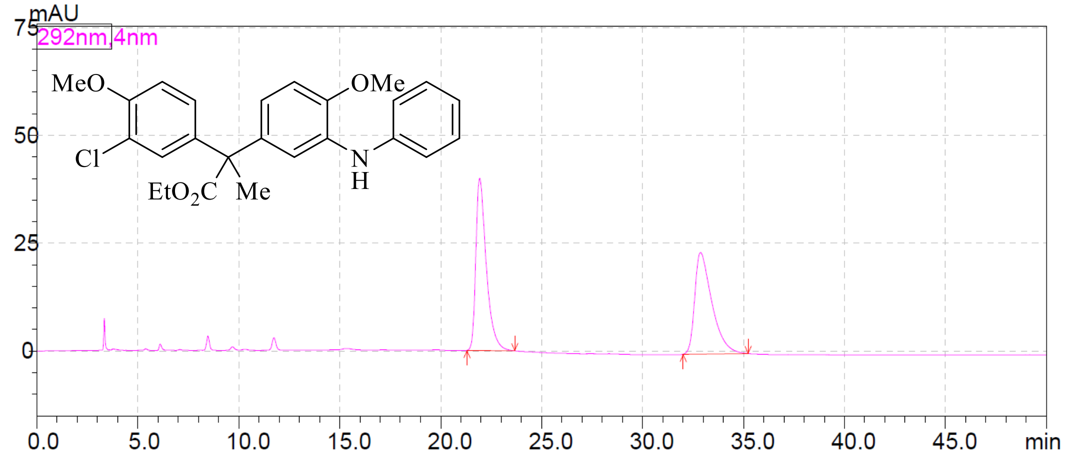

<Peak Table>

PDA Ch1 292nm

Peak#	Ret. Time	Area	Height	Area%	Peak Start	Peak End
1	21.931	1440849	39949	50.848	21.301	23.669
2	32.869	1392780	23545	49.152	32.016	35.227
Total		2833628	63494	100.000		

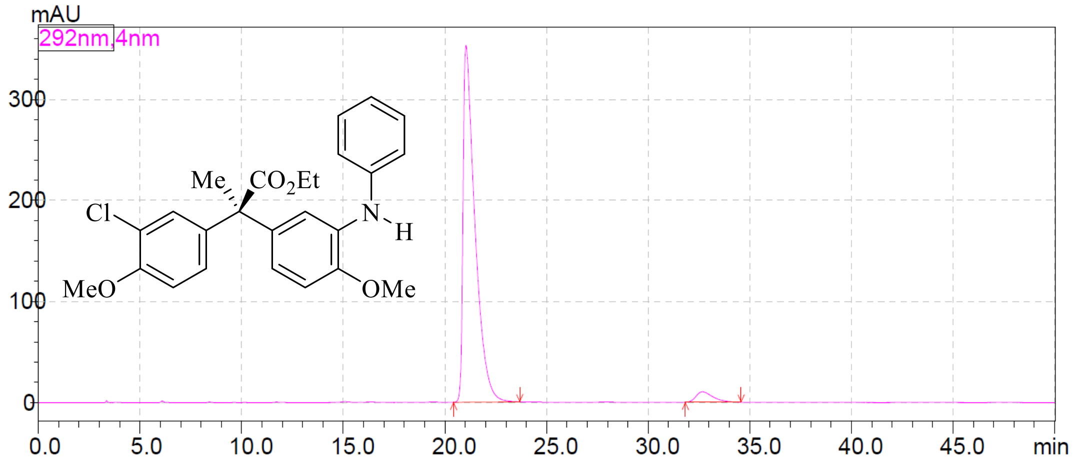

<Peak Table>

PDA Ch1 292nm

Peak#	Ret. Time	Area	Height	Area%	Peak Start	Peak End
1	21.037	14338316	353230	95.985	20.427	23.680
2	32.670	599772	10352	4.015	31.824	34.571
Total		14938088	363582	100.000		

65

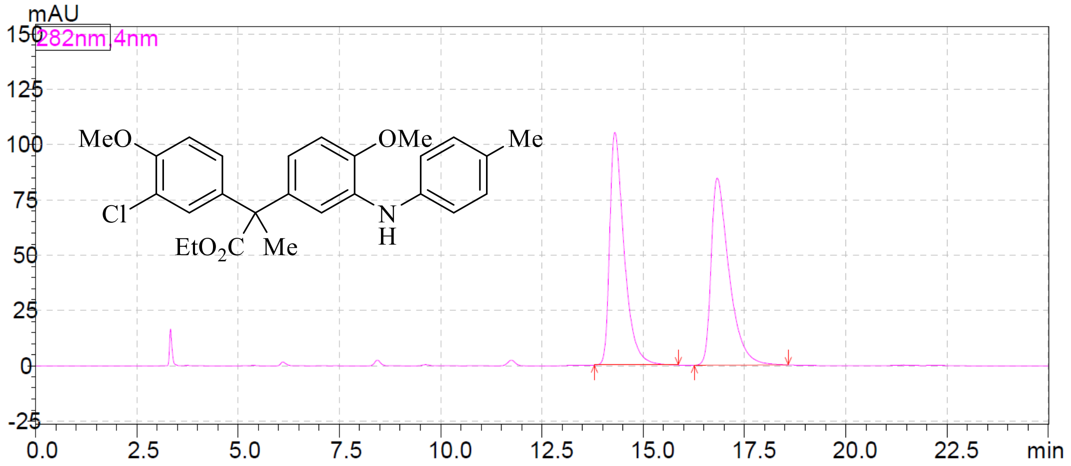

<Peak Table>

PDA Ch1 282nm

Peak#	Ret. Time	Area	Height	Area%	Peak Start	Peak End
1	14.299	2611967	105125	49.954	13.797	15.867
2	16.827	2616740	84543	50.046	16.261	18.581
Total		5228707	189668	100.000		

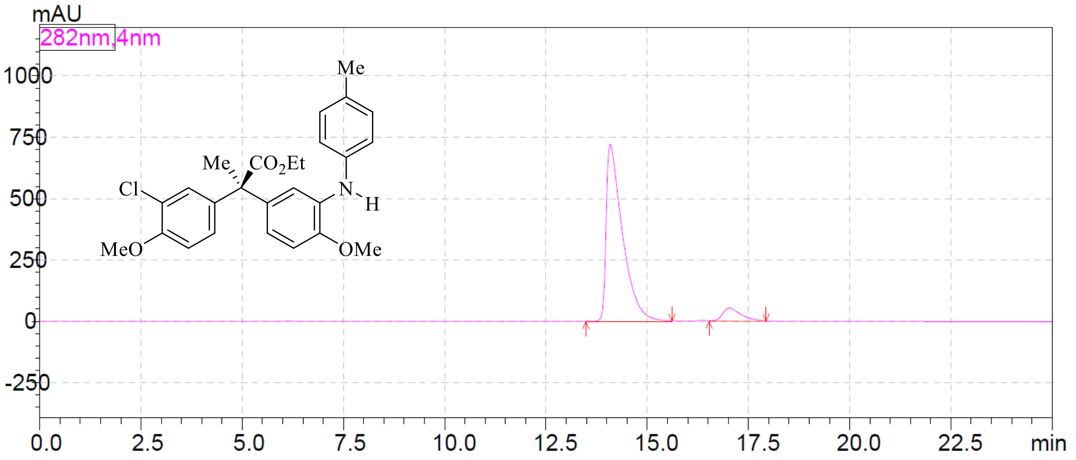

<Peak Table>

PDA Ch1 282nm

Peak#	Ret. Time	Area	Height	Area%	Peak Start	Peak End
1	14.087	20242595	721890	92.007	13.483	15.611
2	17.025	1758638	53680	7.993	16.528	17.936
Total		22001232	775569	100.000		

66

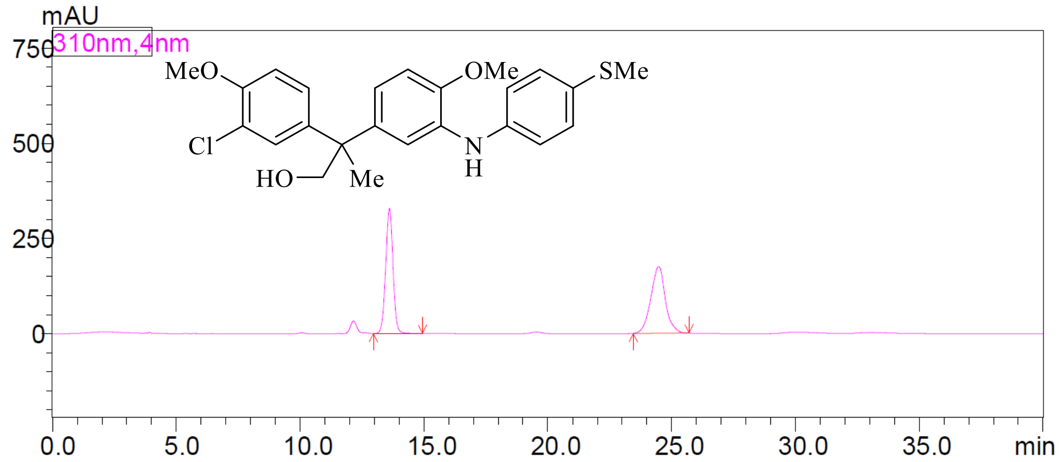

<Peak Table>

PDA Ch1 310nm

Peak#	Ret. Time	Area	Height	Area%	Peak Start	Peak End
1	13.606	6789036	329206	48.692	12.955	14.944
2	24.477	7153792	175781	51.308	23.451	25.707
Total		13942828	504986	100.000		

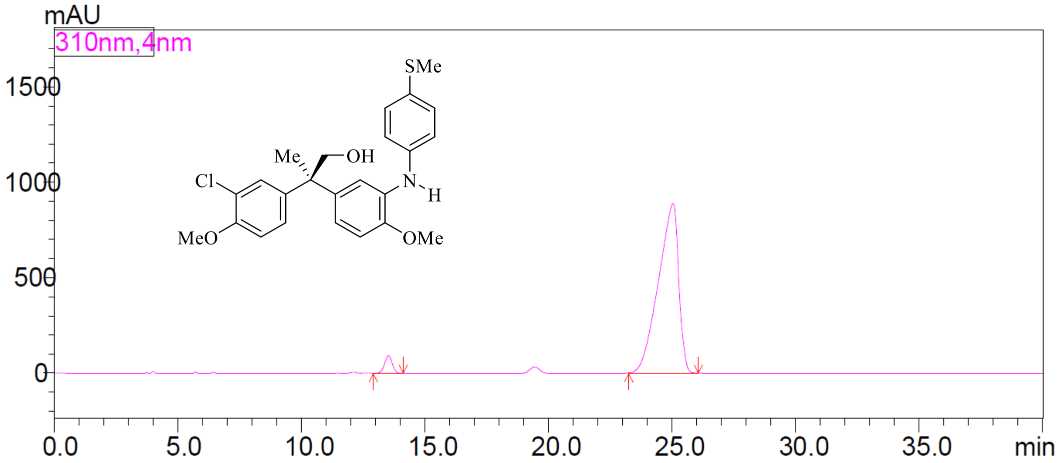

<Peak Table>

PDA Ch1 310nm

Peak#	Ret. Time	Area	Height	Area%	Peak Start	Peak End
1	13.516	2047641	92873	3.857	12.907	14.112
2	25.032	51035971	890654	96.143	23.237	26.059
Total		53083612	983527	100.000		

67

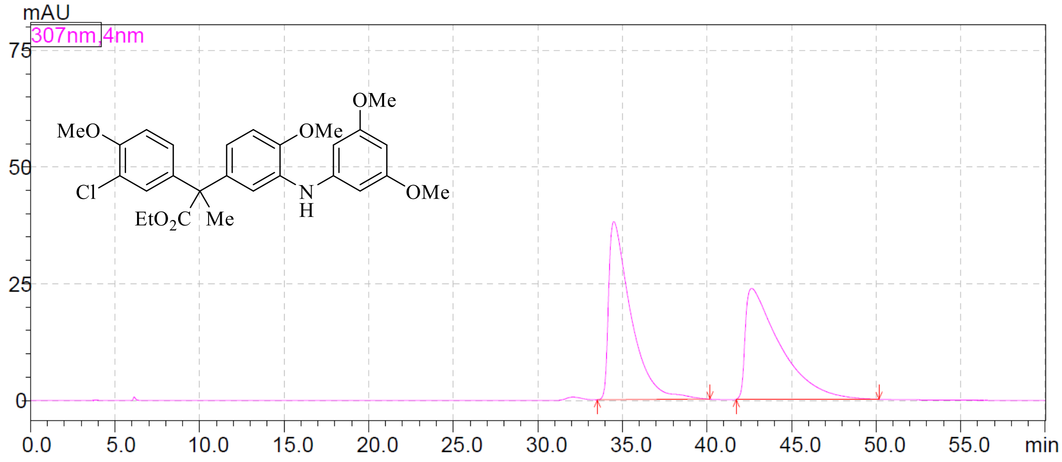

<Peak Table>

PDA Ch1 307nm

Peak#	Ret. Time	Area	Height	Area%	Peak Start	Peak End
1	34.495	3505015	38209	50.718	33.525	40.181
2	42.647	3405741	23779	49.282	41.728	50.171
Total		6910757	61988	100.000		

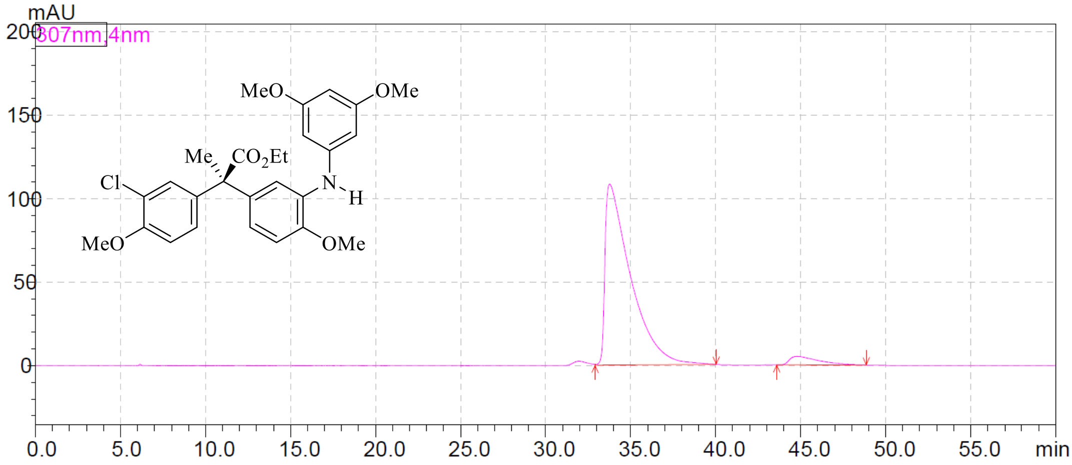

<Peak Table>

PDA Ch1 307nm

Peak#	Ret. Time	Area	Height	Area%	Peak Start	Peak End
1	33.766	11301503	108386	94.917	32.912	40.032
2	44.775	605206	5090	5.083	43.589	48.853
Total		11906709	113476	100.000		

68

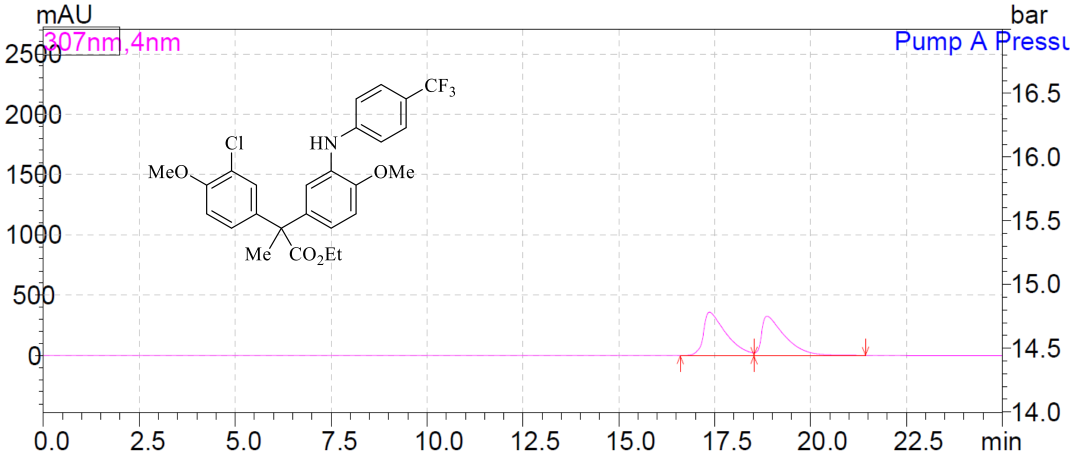

<Peak Table>

PDA Ch1 307nm

Peak#	Ret. Time	Area	Height	Area%	Peak Start	Peak End
1	17.362	14639000	359348	51.835	16.603	18.523
2	18.864	13602582	325584	48.165	18.523	21.429
Total		28241582	684931	100.000		

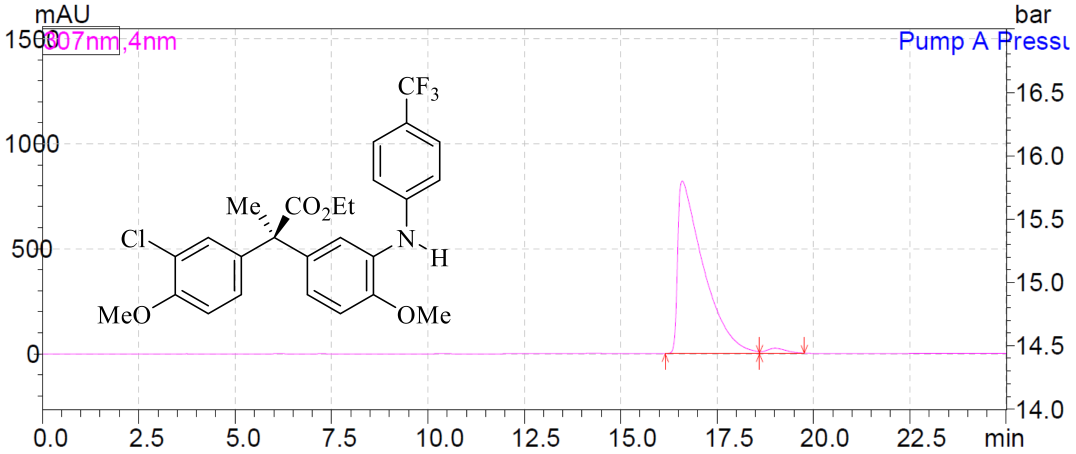

<Peak Table>

PDA Ch1 307nm

Peak#	Ret. Time	Area	Height	Peak Start	Peak End	Area%
1	16.596	38322858	821788	16.155	18.603	97.522
2	19.000	973745	25842	18.603	19.765	2.478
Total		39296604	847631			100.000

69

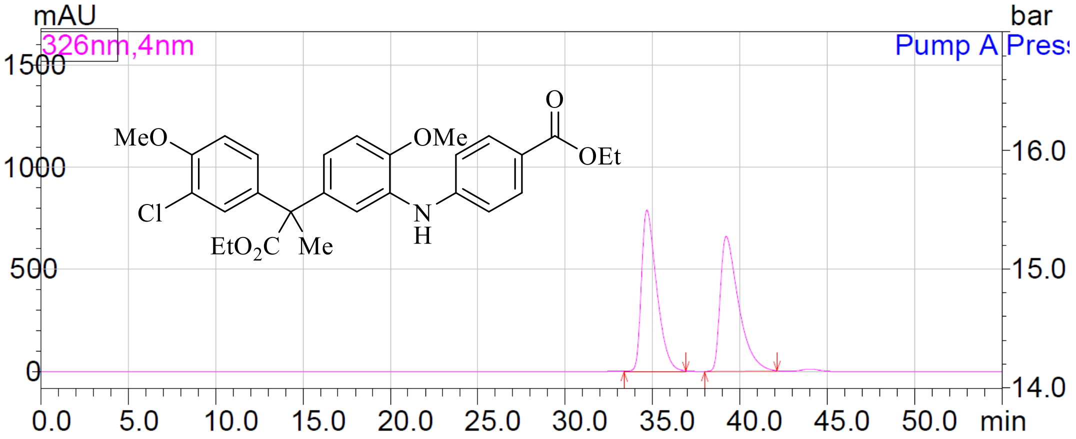

<Peak Table>

PDA Ch1 326nm

Peak#	Ret. Time	Area	Height	Area%	Peak Start	Peak End
1	34.676	48030474	790367	49.782	33.371	36.907
2	39.215	48450646	660629	50.218	37.979	42.112
Total		96481120	1450996	100.000		

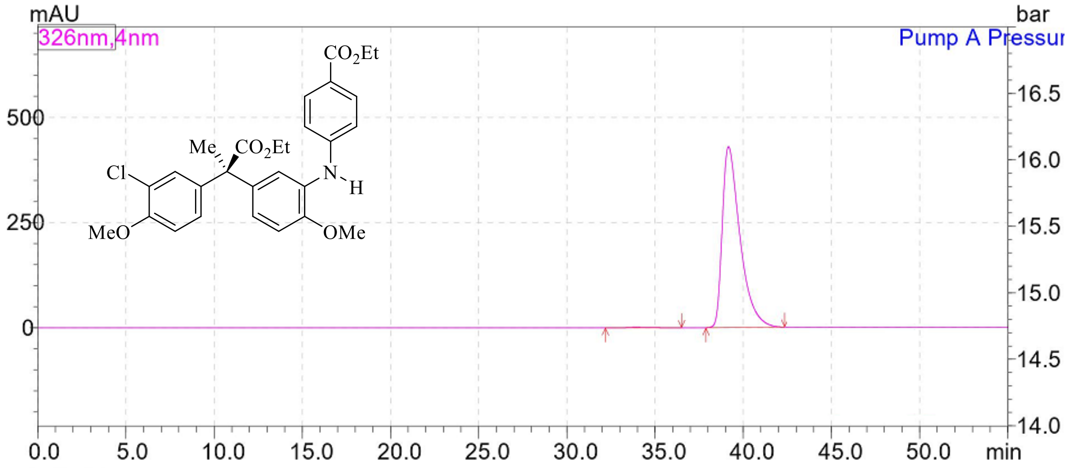

<Peak Table>

PDA Ch1 326nm

Peak#	Ret. Time	Area	Height	Area%	Peak Start	Peak End
1	33.913	124065	1430	0.412	32.181	36.507
2	39.164	29985769	430081	99.588	37.883	42.352
Total		30109834	431511	100.000		

70

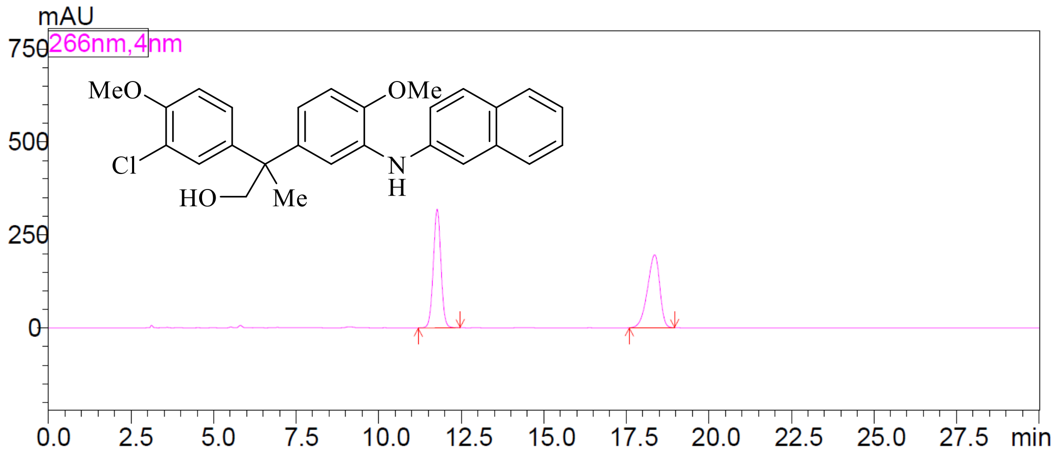

<Peak Table>

PDA Ch1 266nm

Peak#	Ret. Time	Area	Height	Area%	Peak Start	Peak End
1	11.773	5130436	318865	50.237	11.195	12.464
2	18.359	5082051	196270	49.763	17.589	18.960
Total		10212487	515135	100.000		

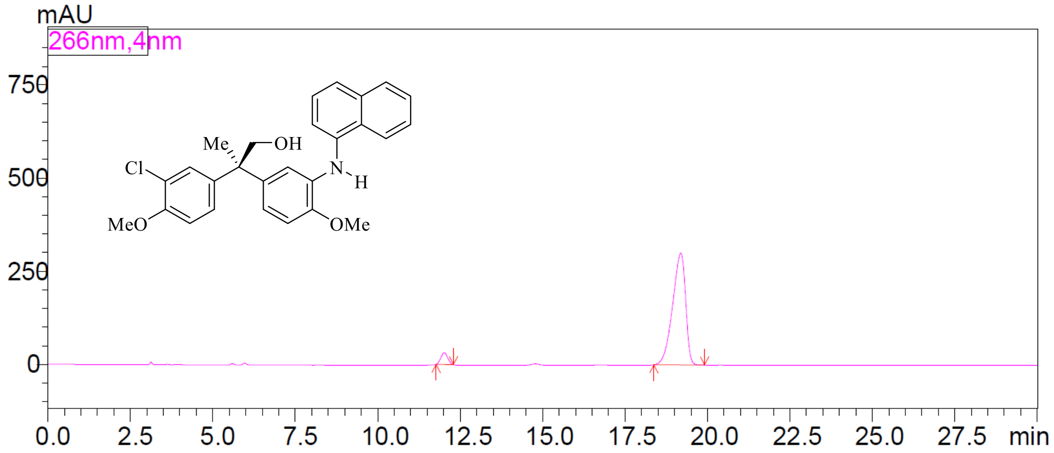

<Peak Table>

PDA Ch1 266nm

Peak#	Ret. Time	Area	Height	Area%	Peak Start	Peak End
1	12.020	468346	31696	5.371	11.776	12.304
2	19.193	8250923	300417	94.629	18.379	19.915
Total		8719268	332113	100.000		

71

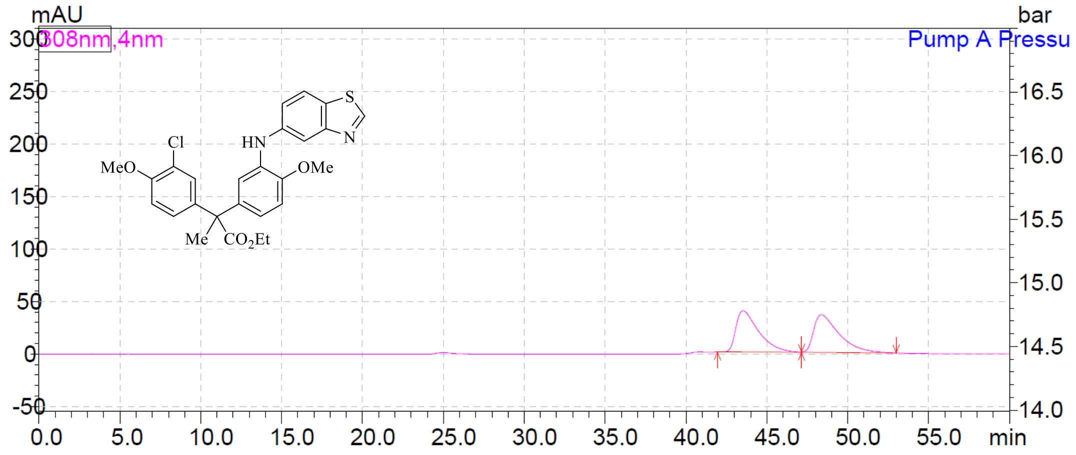

<Peak Table>

PDA Ch1 308nm

Peak#	Ret. Time	Area	Height	Area%	Peak Start	Peak End
1	43.525	3824794	39285	49.475	41.947	47.141
2	48.359	3905992	35718	50.525	47.141	52.992
Total		7730785	75003	100.000		

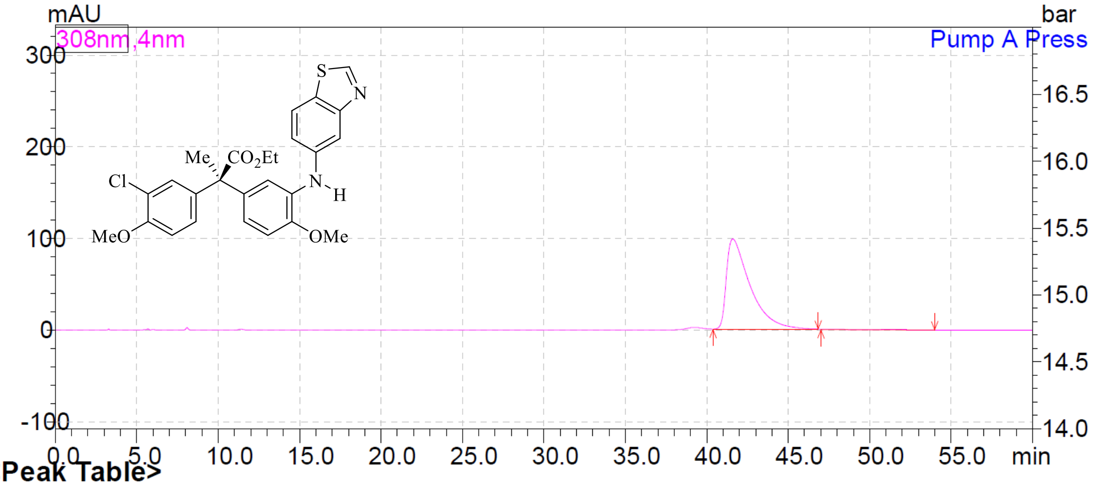

<Peak Table>

PDA Ch1 308nm

Peak#	Ret. Time	Area	Height	Area%	Peak Start	Peak End
1	41.596	9896487	98245	98.832	40.379	46.832
2	47.009	116978	697	1.168	47.008	53.984
Total		10013466	98942	100.000		

Control expermients

23

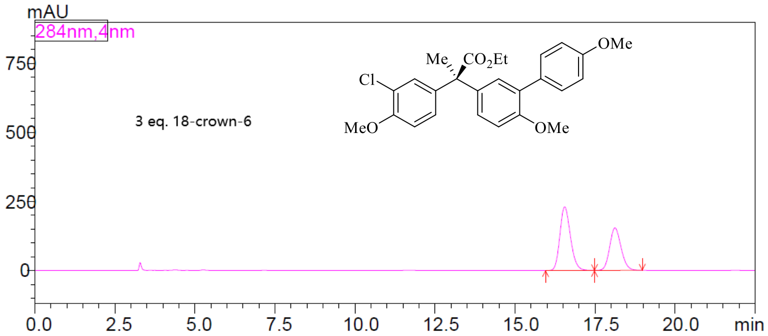

<Peak Table>

PDA Ch1 284nm

Peak#	Ret. Time	Area	Height	Area%	Peak Start	Peak End
1	16.549	5318583	230537	58.210	15.952	17.477
2	18.120	3818262	153786	41.790	17.477	18.971
Total		9136845	384323	100.000		

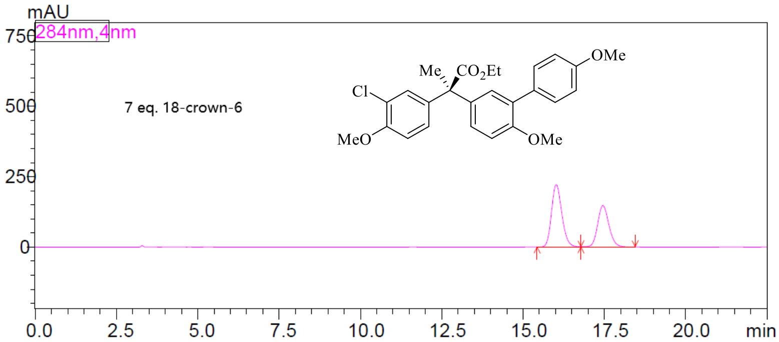

<Peak Table>

PDA Ch1 284nm

Peak#	Ret. Time	Area	Height	Area%	Peak Start	Peak End
1	16.019	4904008	221223	58.005	15.429	16.779
2	17.453	3550380	147042	41.995	16.779	18.448
Total		8454388	368264	100.000		

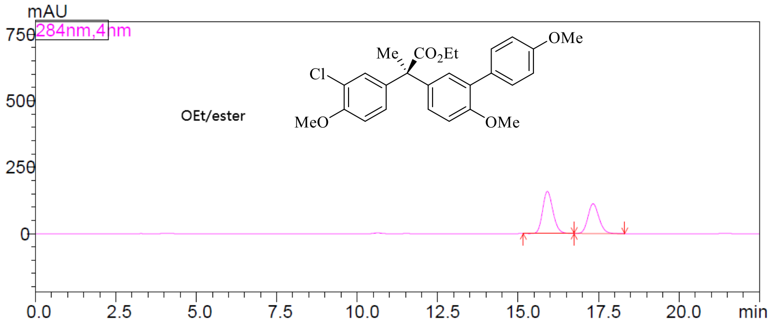

<Peak Table>

PDA Ch1 284nm

Peak#	Ret. Time	Area	Height	Area%	Peak Start	Peak End
1	15.910	3558766	158935	56.833	15.163	16.741
2	17.330	2703058	112512	43.167	16.741	18.315
Total		6261824	271447	100.000		

75

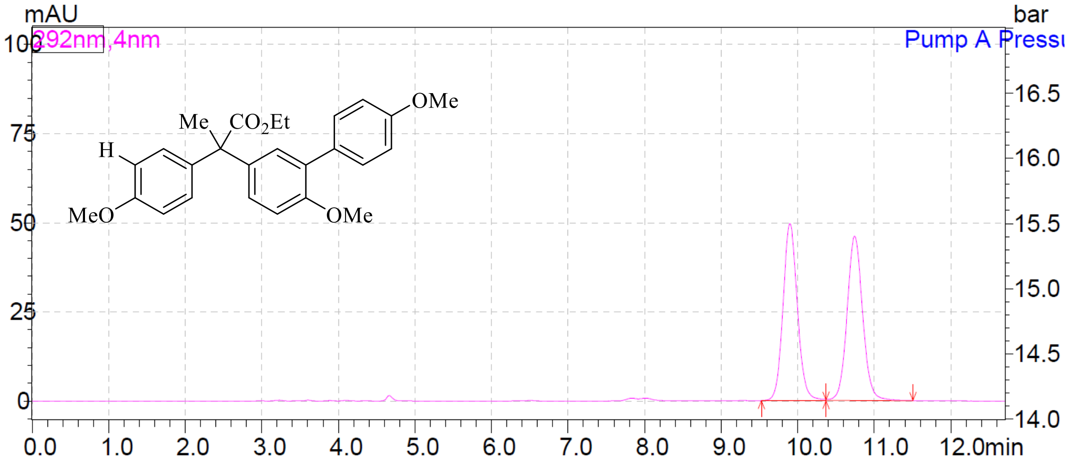

<Peak Table>

PDA Ch1 292nm

Peak#	Ret. Time	Area	Height	Area%	Peak Start	Peak End
1	9.895	647599	49665	49.738	9.525	10.368
2	10.741	654423	46101	50.262	10.368	11.504
Total		1302022	95766	100.000		

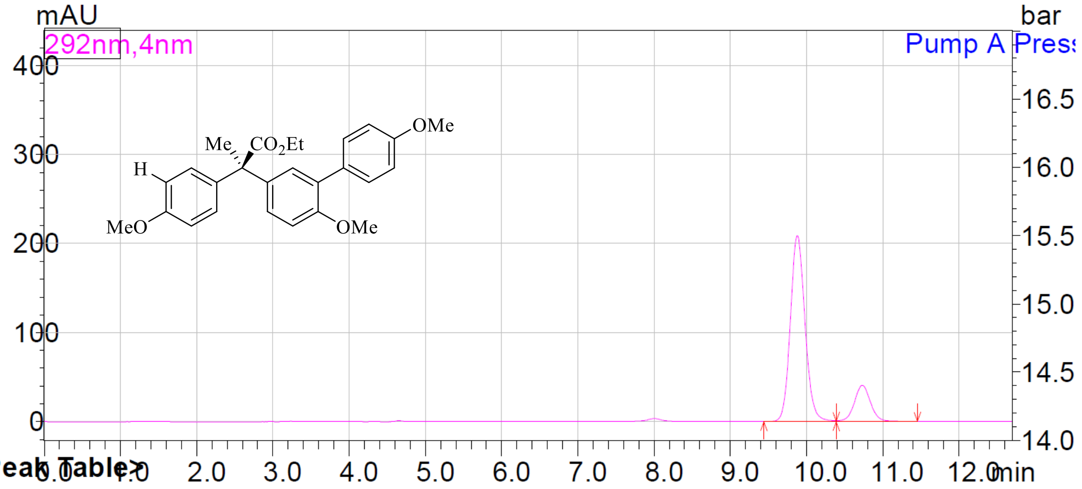

<Peak Table>

PDA Ch1 292nm

Peak#	Ret. Time	Area	Height	Area%	Peak Start	Peak End
1	9.877	2799188	208811	82.212	9.440	10.389
2	10.729	605635	40819	17.788	10.389	11.456
Total		3404824	249631	100.000		

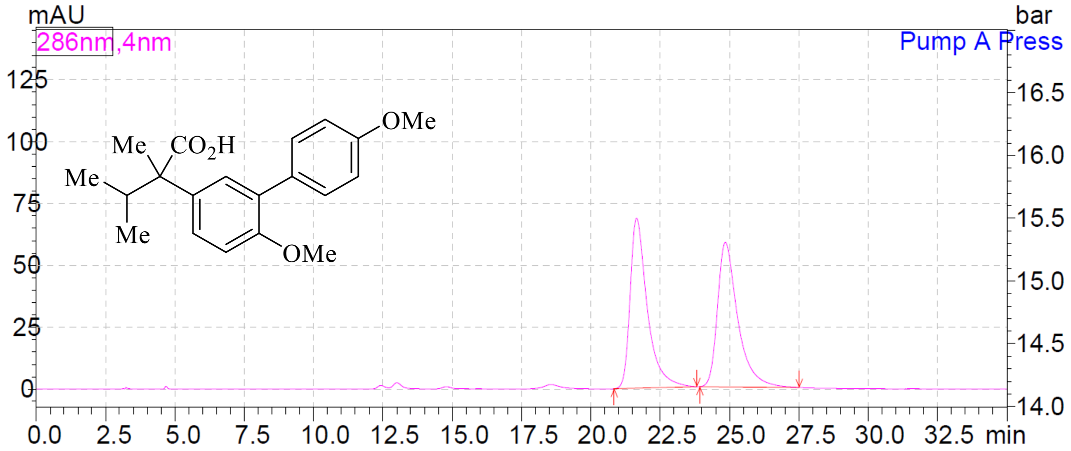

<Peak Table>

PDA Ch1 286nm

Peak#	Ret. Time	Area	Height	Area%	Peak Start	Peak End
1	21.646	2968640	68743	50.026	20.843	23.819
2	24.847	2965574	58401	49.974	23.925	27.509
Total		5934214	127143	100.000		

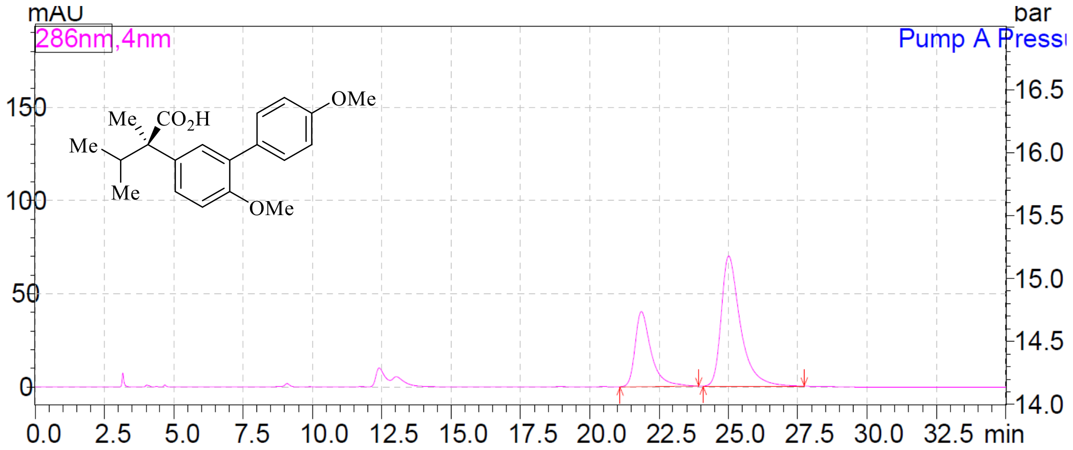

<Peak Table>

PDA Ch1 286nm

Peak#	Ret. Time	Area	Height	Area%	Peak Start	Peak End
1	21.860	1623341	40299	32.577	21.093	23.931
2	25.008	3359687	69807	67.423	24.091	27.733
Total		4983029	110106	100.000		

83

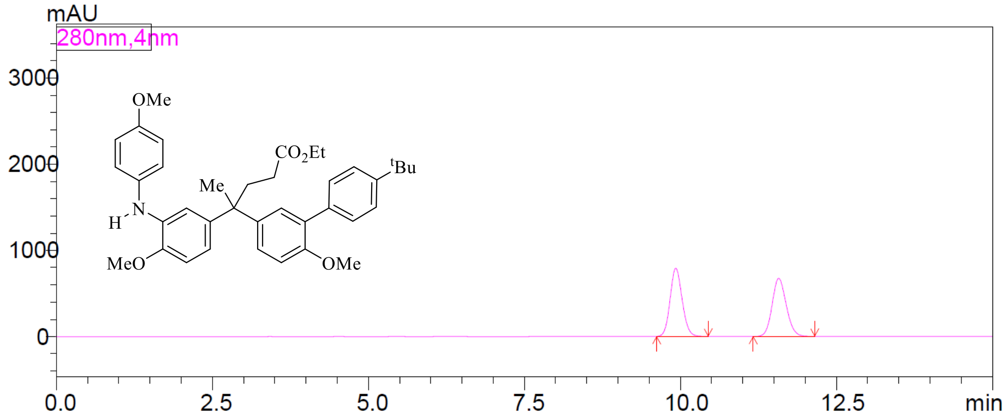

<Peak Table>

PDA Ch1 280nm

Peak#	Ret. Time	Area	Height	Area%	Peak Start	Peak End
1	9.925	10627982	790657	49.950	9.616	10.443
2	11.574	10649171	673841	50.050	11.163	12.149
Total		21277153	1464498	100.000		

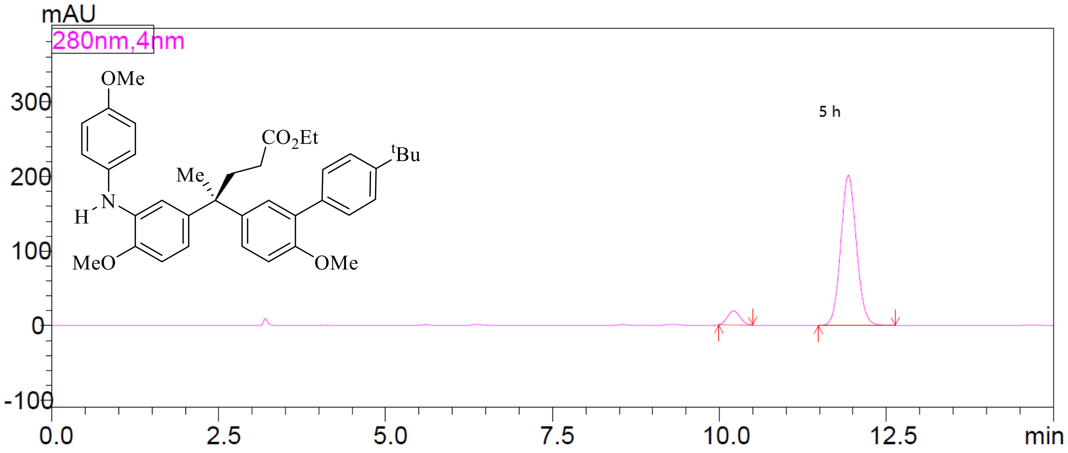

<Peak Table>

PDA Ch1 280nm

Peak#	Ret. Time	Area	Height	Area%	Peak Start	Peak End
1	10.210	248784	18878	7.006	9.989	10.496
2	11.929	3302212	200924	92.994	11.483	12.629
Total		3550995	219802	100.000		

86

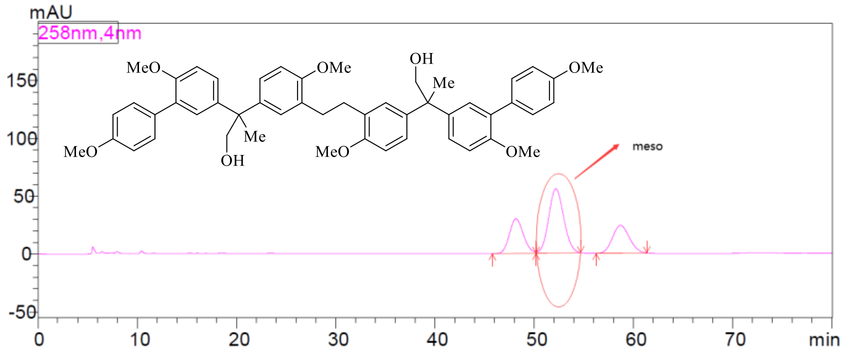

<Peak Table>

PDA Ch1 258nm

Peak#	Ret. Time	Area	Height	Area%	Peak Start	Peak End
1	48.177	2935810	30046	25.219	45.797	50.203
2	52.191	5844739	55547	50.207	50.203	54.699
3	58.694	2860682	24157	24.574	56.256	61.344
Total		11641231	109750	100.000		

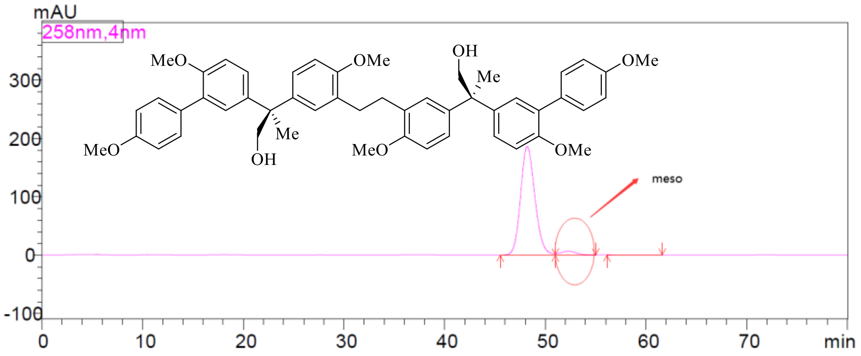

<Peak Table>

PDA Ch1 258nm

Peak#	Ret. Time	Area	Height	Area%	Peak Start	Peak End
1	48.205	18438877	186161	95.976	45.515	51.003
2	52.268	743927	6595	3.872	51.003	54.992
3	56.125	29182	356	0.152	56.123	61.627
Total		19211986	193112	100.000		

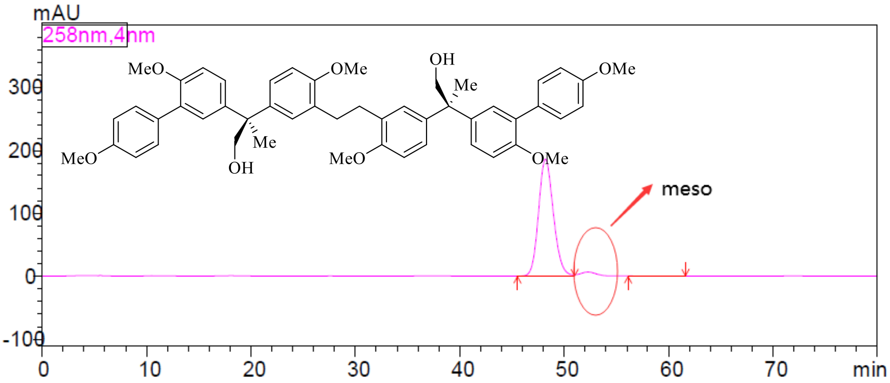

<Peak Table>

PDA Ch1 258nm

Peak#	Ret. Time	Area	Height	Area%	Peak Start	Peak End
1	48.205	18438877	186161	99.842	45.515	51.003
2	56.125	29182	356	0.158	56.123	61.627
Total		18468059	186517	100.000		

88

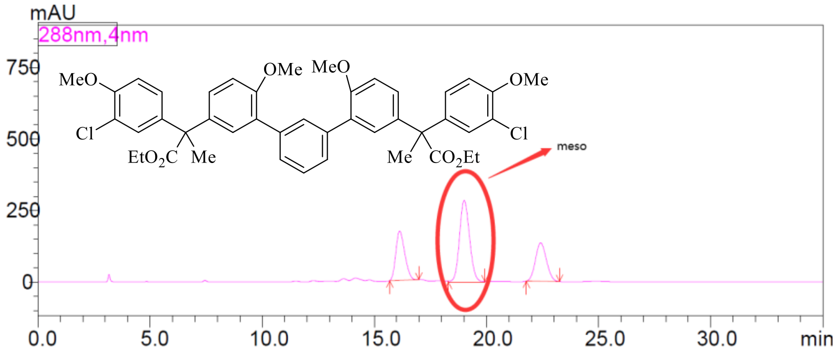

<Peak Table>

PDA Ch1 288nm

Peak#	Ret. Time	Area	Height	Area%	Peak Start	Peak End
1	16.127	4725792	172096	25.797	15.685	16.976
2	19.003	8985751	285455	49.052	18.304	19.931
3	22.414	4607454	134740	25.151	21.776	23.253
Total		18318996	592291	100.000		

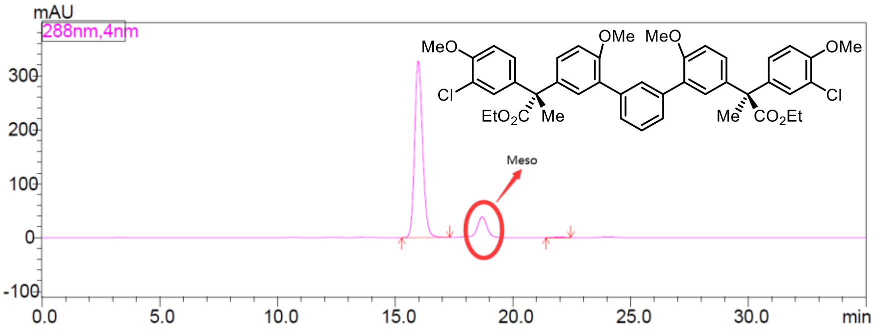

<Peak Table>

PDA Ch1 288nm

Peak#	Ret. Time	Area	Height	Area%	Peak Start	Peak End
1	15.981	7783116	327558	99.600	15.280	17.323
2	21.975	31264	1075	0.400	21.408	22.464
Total		7814380	328633	100.000		

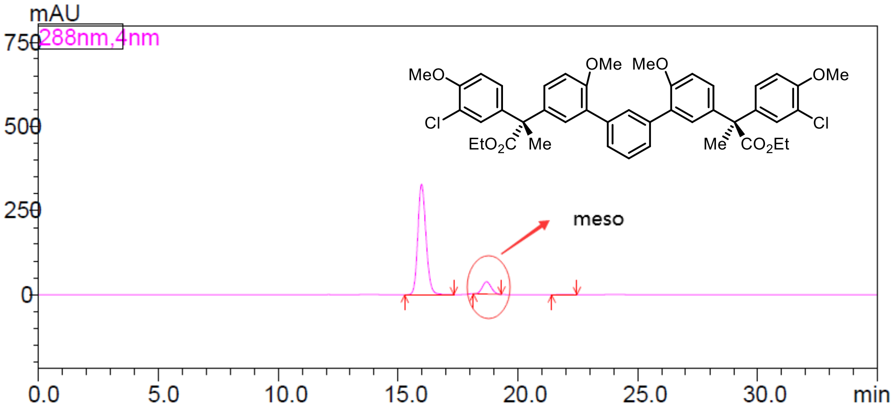

<Peak Table>

PDA Ch1 288nm

Peak#	Ret. Time	Area	Height	Area%	Peak Start	Peak End
1	15.981	7783116	327558	88.729	15.280	17.323
2	18.694	957451	36680	10.915	18.144	19.317
3	21.975	31264	1075	0.356	21.408	22.464
Total		8771831	365313	100.000		

90

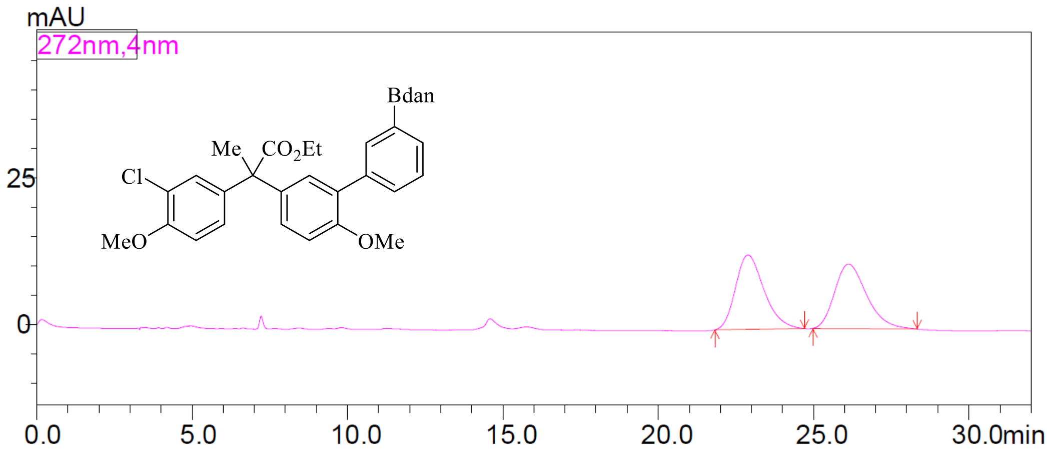

<Peak Table>

PDA Ch1 272nm

Peak#	Ret. Time	Area	Height	Area%	Peak Start	Peak End
1	22.885	811871	12702	50.708	21.819	24.704
2	26.122	789211	11007	49.292	24.981	28.336
Total		1601082	23709	100.000		

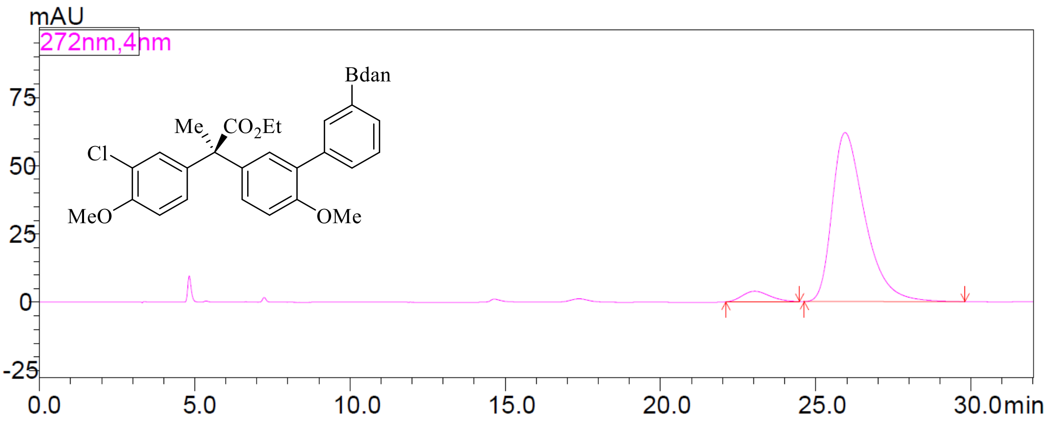

<Peak Table>

PDA Ch1 272nm

Peak#	Ret. Time	Area	Height	Area%	Peak Start	Peak End
1	23.032	240068	3871	5.011	22.107	24.485
2	25.946	4551106	61955	94.989	24.635	29.808
Total		4791174	65826	100.000		

91

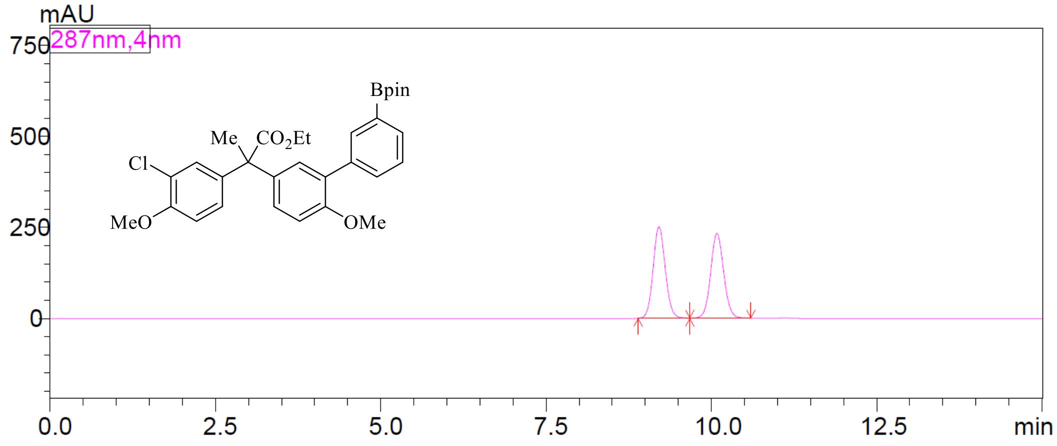

<Peak Table>

PDA Ch1 287nm

Peak#	Ret. Time	Area	Height	Area%	Peak Start	Peak End
1	9.206	3212124	251336	50.140	8.891	9.675
2	10.083	3194219	232645	49.860	9.675	10.597
Total		6406342	483982	100.000		

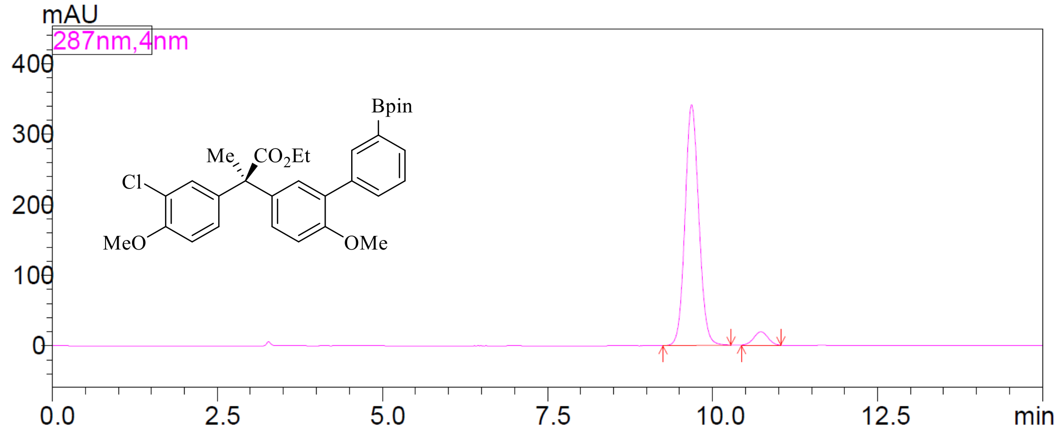

<Peak Table>

PDA Ch1 287nm

Peak#	Ret. Time	Area	Height	Area%	Peak Start	Peak End
1	9.684	4967474	341644	94.591	9.248	10.277
2	10.733	284070	19129	5.409	10.437	11.035
Total		5251544	360774	100.000		

95

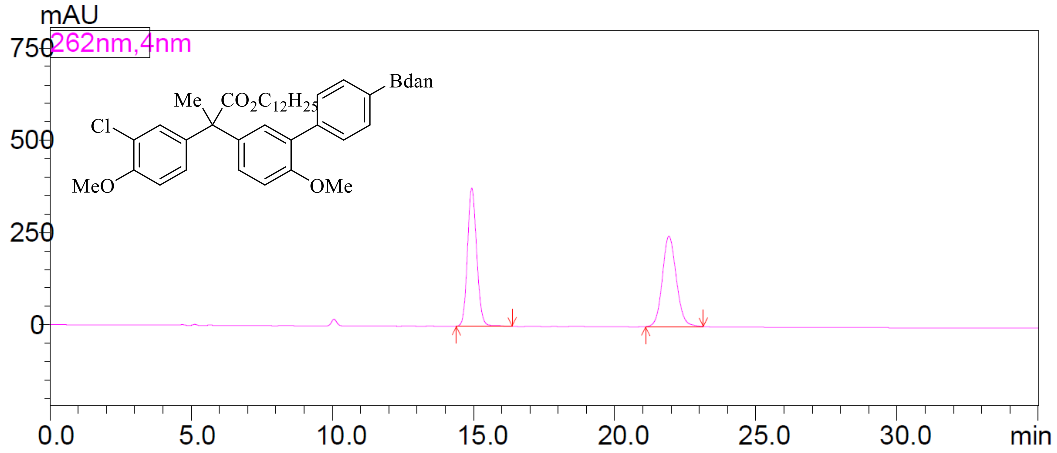

<Peak Table>

PDA Ch1 262nm

Peak#	Ret. Time	Area	Height	Area%	Peak Start	Peak End
1	14.939	8543763	374330	50.275	14.395	16.363
2	21.922	8450330	244992	49.725	21.099	23.136
Total		16994093	619322	100.000		

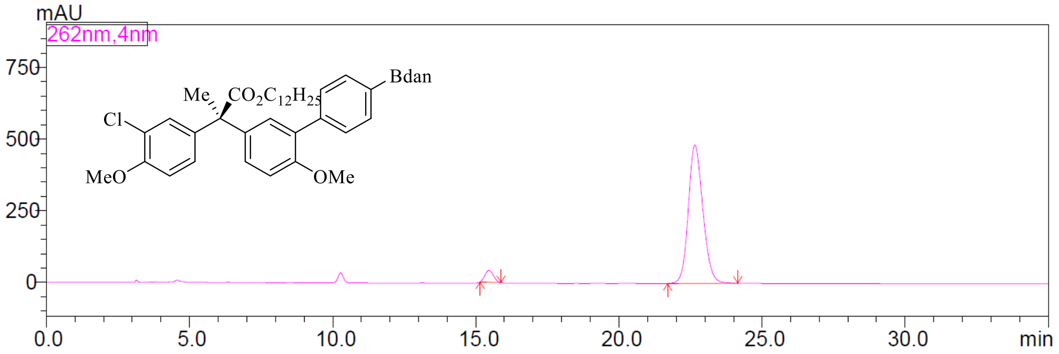

<Peak Table>

PDA Ch1 262nm

Peak#	Ret. Time	Area	Height	Area%	Peak Start	Peak End
1	15.460	849927	41063	4.741	15.152	15.877
2	22.651	17077295	482713	95.259	21.701	24.149
Total		17927222	523776	100.000		

96

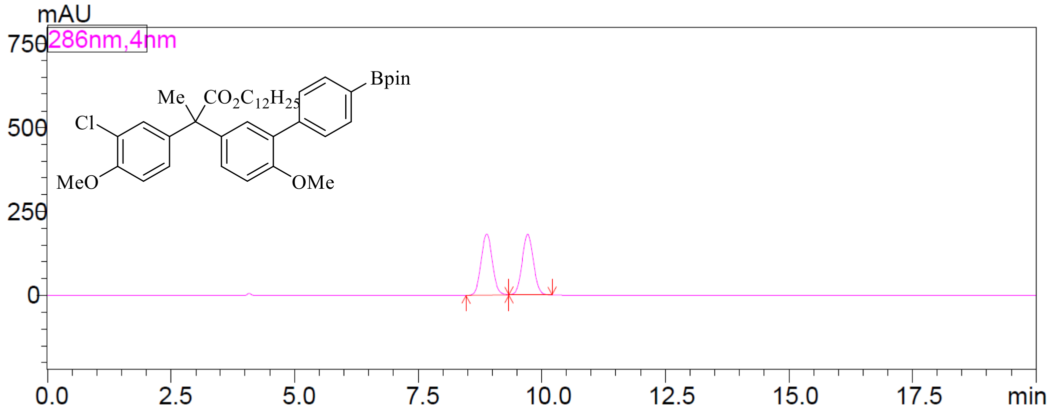

<Peak Table>

PDA Ch1 286nm

Peak#	Ret. Time	Area	Height	Area%	Peak Start	Peak End
1	8.888	2900706	182211	50.056	8.469	9.333
2	9.716	2894245	181034	49.944	9.333	10.208
Total		5794952	363245	100.000		

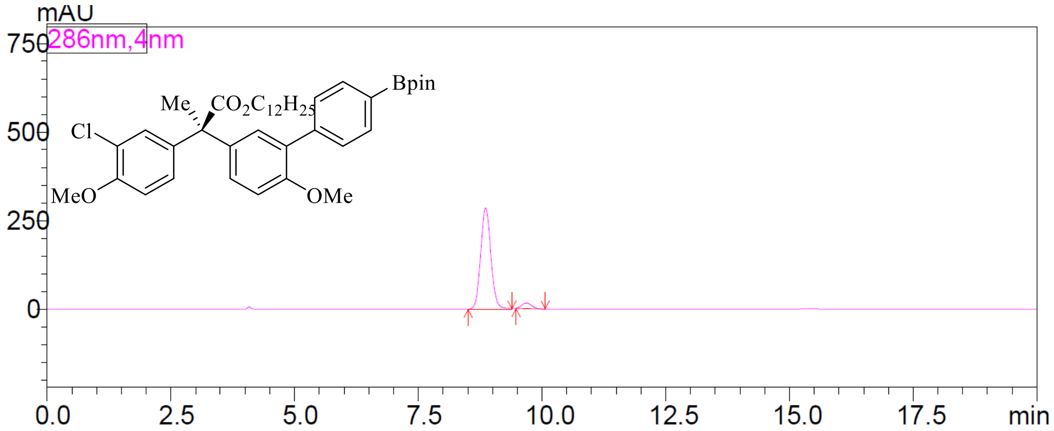

<Peak Table>

PDA Ch1 286nm

Peak#	Ret. Time	Area	Height	Area%	Peak Start	Peak End
1	8.860	4088209	284937	95.041	8.507	9.392
2	9.688	213296	15796	4.959	9.472	10.064
Total		4301505	300734	100.000		

X-ray crystallography data

(S_S, S)-**L3**

The .cif data file is attached as a separate document. CCDC Deposition Number **2054850**

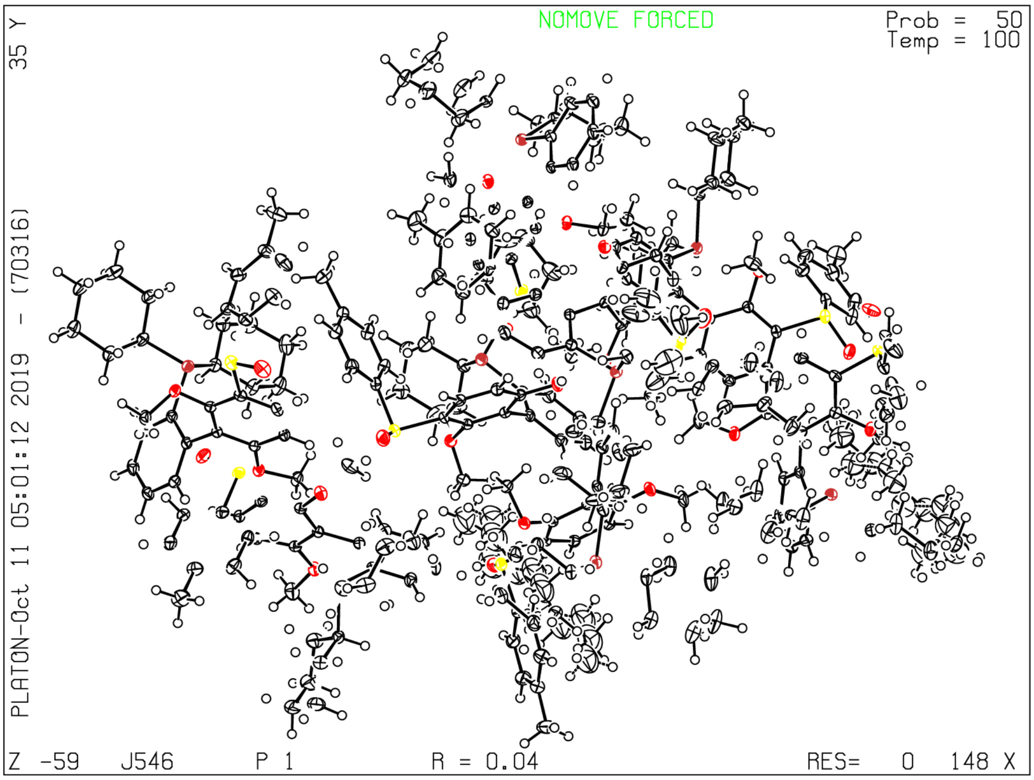

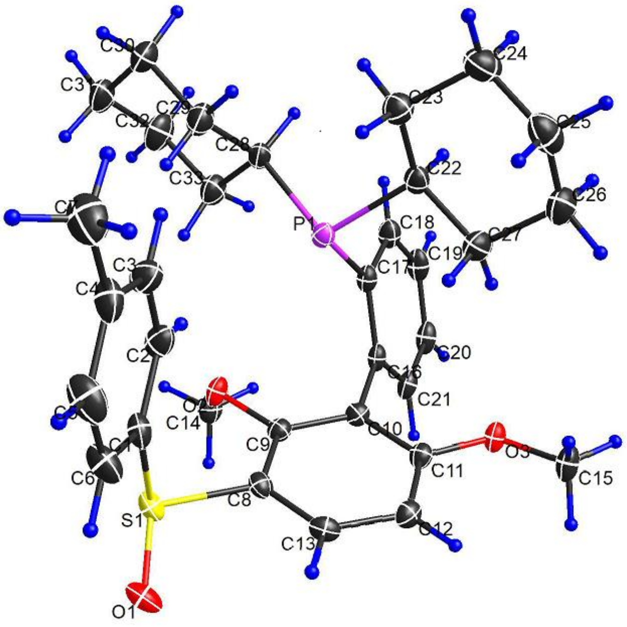

39

The .cif data file is attached as a separate document. CCDC Deposition Number **2257332**

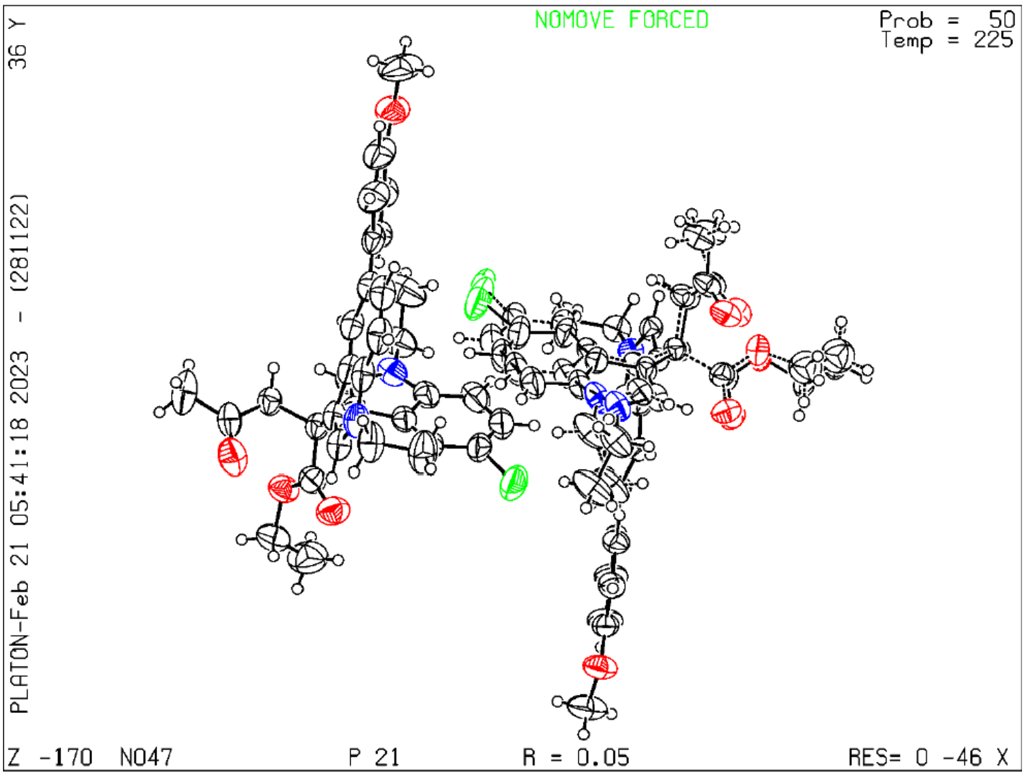

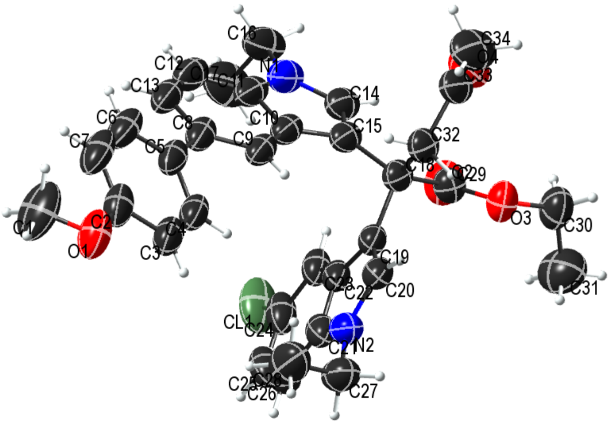

70

The .cif data file is attached as a separate document. CCDC Deposition Number **2257333**

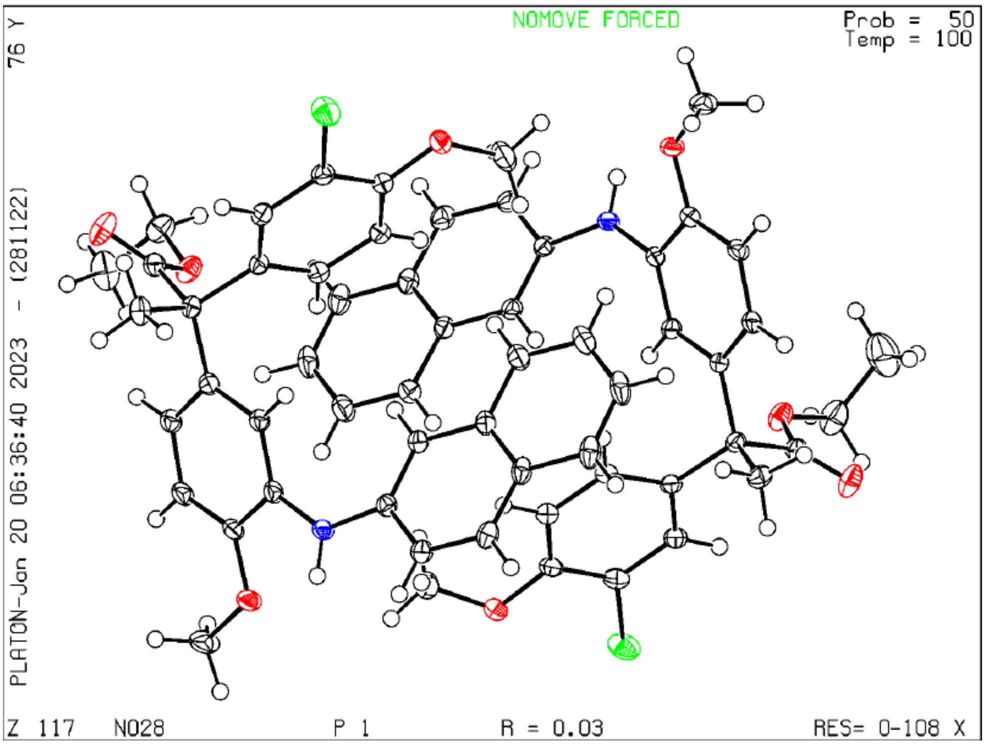

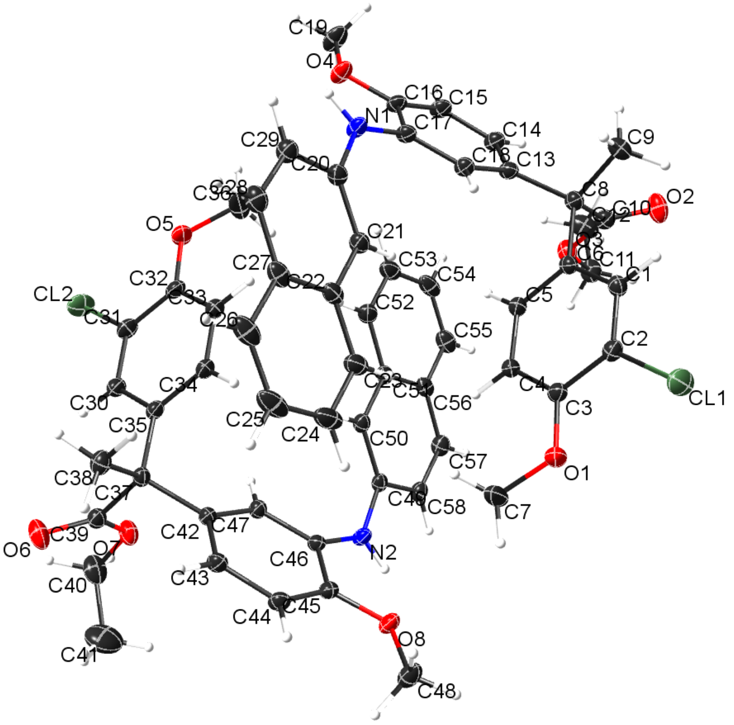

Supplement: Supplementary file 1 — ja3c04877_si_001.pdf [file ja3c04877_si_001.pdf]
